# Effects of exercise on multiple health outcomes in children and adolescents with overweight or obesity: a meta-analysis of 176 randomized controlled trials and its implications for global obesity prevention

**DOI:** 10.1186/s12966-026-01913-0

**Published:** 2026-04-17

**Authors:** Jie Men, Pengbo Wang, Jingwen Wang, Guoyu Zhu, Zhengyang Yu, Simin Wu, Yuxi Zhang, Weiqi An, Zhaowei Li, Rui Ma, Ruiqi Zhang, Shufeng Li, Yaoyong Wang, Penghong Liu

**Affiliations:** 1 Shanxi University of Medicine, Fenyang, 032200 China; 2 Department of Respiratory and Critical Care Medicine, Fenyang Hospital of Shanxi Province, Fenyang, 032200 China; 3 First Hospital of Shanxi Medical University, Taiyuan , 030001 China; 4 Shanxi Provincial Clinical Medical Research Center for Mental and Psychological Disorders (Depression), Taiyuan, China

**Keywords:** Obesity, Ooverweight, Children, Adolescents, Exercise, Holistic health, Policy

## Abstract

**Background:**

The continuing rise in overweight and obesity among children and adolescents portends a life-course public-health crisis. Although evidence has been accumulating that exercise interventions improve health in those with overweight or obesity, few studies have integrated multidimensional health outcomes within a unified analytical framework. To systematically evaluate the effects of exercise interventions on multidimensional health outcomes—including anthropometric, cardiometabolic, cardiorespiratory, and mental health indicators—among children and adolescents with overweight or obesity, and to explore implications for global obesity prevention and control.

**Methods:**

Six databases (PubMed, Embase, Cochrane Library, Web of Science, CNKI, and Wanfang Data) were searched from inception to January 10, 2025, for randomized controlled trials of exercise interventions in children and adolescents with overweight or obesity. Data were pooled using random- or fixed-effects models, as appropriate. Risk of bias was assessed using the Cochrane Risk of Bias Tool (RoB 2.0), and the certainty of evidence was rated with the Grading of Recommendations, Assessment, Development and Evaluations (GRADE) approach. The protocol was registered with PROSPERO (CRD420251169879).

**Results:**

A total of 176 trials (11,696 participants from 32 countries) were analyzed. Exercise interventions significantly reduced body mass index (MD: −1.25 kg m^−2^, 95% CI: −1.51 to −0.98), waist circumference (MD: −2.86 cm, 95% CI: −3.52 to −2.20), and body fat percentage (MD: −2.39, 95% CI: −2.81 to −1.96); improved cardiorespiratory fitness (maximal oxygen consumption MD: 2.83 mL kg^−1^ min^−1^, 95% CI: 2.17 to 3.50; peak oxygen uptake MD: 2.56 mL kg^−1^ min^−1^, 95% CI: 1.86 to 3.27); and lowered resting heart rate (MD: −2.89 bpm, 95% CI: −3.82 to −1.97). Cardiometabolic indicators also improved, with reductions in systolic blood pressure (MD: −3.90 mmHg, 95% CI: −5.34 to −2.46) and diastolic blood pressure (MD: −1.70 mmHg, 95% CI: −2.68 to −0.71); total cholesterol (MD: −0.35 mmol/l, 95% CI: −0.43 to −0.27), triglycerides (MD: −0.23 mmol/l, 95% CI: −0.28 to −0.17), and low-density lipoprotein cholesterol (MD: −0.28 mmol/l, 95% CI: −0.35 to −0.20) decreased, accompanied by an increase in high-density lipoprotein cholesterol (MD: 0.08 mmol/l, 95% CI: 0.05 to 0.10). Fasting glucose (MD: −0.20 mmol/l, 95% CI: −0.26 to −0.13) and fasting insulin (MD: −3.93 µU/mL, 95% CI: −4.80 to −3.06) also declined. Regarding mental health, exercise improved self-esteem (SMD: 0.18, 95% CI: 0.04 to 0.33) and self-worth (SMD: 0.25, 95% CI: 0.10 to 0.39), although the evidence for effects on depression and anxiety was limited. The observed effects varied across prespecified subgroups defined by exercise dose and type, region, country income level, sex, and obesity or metabolic phenotypes.

**Conclusions:**

Exercise interventions consistently improved multidimensional health outcomes in children and adolescents with overweight or obesity, showing dose- and type-dependent effects with greater benefits in higher-risk groups. These findings underscore the value of exercise as a key strategy to address obesity, while recognizing that political and economic barriers may hinder policy translation. Future research should refine intervention approaches and strengthen pathways from evidence to policy implementation.

**Supplementary Information:**

The online version contains supplementary material available at 10.1186/s12966-026-01913-0.

## Background

Predictive modeling studies estimate that by 2050, the global population of children and adolescents with overweight or obesity will reach 746 million—an increase of 150% compared with 2025 [1]. This life-course public health crisis not only undermines health during childhood and adolescence but also substantially increases the risk of developing non-communicable diseases (NCDs) in adulthood, including cancer, heart disease, stroke, type 2 diabetes, and mental disorders [2]. Data from the Global Burden of Disease, Injuries, and Risk Factors Study (GBD 2021) further indicate that obesity accounts for 4.8 million deaths and 128.5 million disability-adjusted life years worldwide, representing 8.4% of total global deaths and 5.4% of the overall disease burden [3]. If current trends continue, the gains in global life expectancy achieved to date may be offset—or even reversed—by 2050 [3].

Beyond the substantial health risks, the economic burden attributable to overweight and obesity has already exceeded 2.19% of the global gross domestic product (GDP), equivalent to US$1.33 trillion [4], and is projected to reach 6.4% (US$4.7 trillion) by 2050—surpassing the economic losses caused by climate change [5]. Unfortunately, no country or region has yet succeeded in curbing the rising trend of childhood and adolescent obesity [1]. This shortfall signifies not only the loss of a potential intergenerational health dividend but also the onset of multiple crises, including multisystem health deterioration, socioeconomic stagnation, and widening health inequities [6]. Therefore, effective interventions and policies are urgently needed to curb the rapid increase in obesity, which is essential for safeguarding future population health and ensuring sustainable economic development.

As a key behavioural intervention, exercise is explicitly recommended by both the World Health Organization (WHO) and the American Academy of Pediatrics for the prevention and management of overweight and obesity [7, 8]. Compared with pharmacological or surgical interventions, exercise does not cause gastrointestinal adverse reactions, hypertension, mental disorders, or a reoperation risk as high as 25% [8]; moreover, medium- to long-term follow-up studies have confirmed its sustained health benefits [9]. On the basis of extensive evidence linking physical activity with diverse health outcomes, the WHO updated its Guidelines on Physical Activity and Sedentary Behaviour in 2020. Due to the limited evidence on obesity phenotypes [10], the guideline provides a general recommendation for children and adolescents to engage in at least 60 min of moderate-to-vigorous physical activity daily [11]. Notably, this recommendation differs in its emphasis on exercise dose from previous meta-analyses focusing on children and adolescents with overweight or obesity. For example, the study by Menjie et al. [12] and colleagues suggested that moderate-to-low intensity exercise sessions lasting at least 50 min may be associated with more favourable health outcomes. This discrepancy indicates that guidelines developed for the general population may not fully capture the optimal exercise dose characteristics for specific high-risk groups, underscoring the need for further targeted research.

Evidence has shown that exercise improves body composition, cardiorespiratory fitness, cardiometabolic health, and mental well-being in children and adolescents with overweight or obesity [13, 14]. However, studies that integrate these multidimensional health outcomes within a unified analytical framework remain scarce. Given that a single outcome cannot comprehensively capture overall health effects, multi-outcome analyses can provide a more holistic understanding and yield more reliable estimates of intervention effectiveness. Existing research has predominantly focused on single outcomes or specific exercise modalities, and is often limited by small sample sizes, publication bias, and low-quality evidence [14, 15]—factors that undermine both the generalisability of findings and their persuasiveness for policy translation. Bridging the gap between evidence and policy represents another major challenge. Evidence-based policymaking frameworks that incorporate graded systems of evidence quality, such as GRADE (Grading of Recommendations, Assessment, Development and Evaluation), have been validated in areas like cancer prevention [16] and COVID-19 vaccination [17]. However, in the field of exercise intervention, effective policy translation remains constrained. These barriers are closely related to the strength and hierarchy of available evidence as well as socioeconomic factors, yet few studies have examined the relationship between evidence generation and policy implementation in this domain.

We conducted a meta-analysis to evaluate the effects of exercise interventions on multidimensional health outcomes in children and adolescents with overweight or obesity. The study explored how exercise dose and type, region, country income level, sex, and obesity/metabolic phenotypes may modify the health benefits of exercise, as well as the barriers to translating evidence into policy. The aim was to provide scientific evidence to inform more targeted intervention strategies and to support effective obesity control and improved health equity.

## Methods

This systematic review and meta-analysis was conducted in accordance with the Preferred Reporting Items for Systematic Reviews and Meta-Analysis (PRISMA) guidelines [18] and the Cochrane Handbook of Systematic Reviews of Interventions for systematic reviews [19]. This review protocol was registered with the PROSPERO international database (protocol number: CRD420251169879).

### Search strategy

The literature search was conducted across six databases—PubMed, Embase, the Cochrane Library, Web of Science, CNKI (China National Knowledge Infrastructure), and Wanfang Data—from database inception to January 10, 2025. The search strategy was developed based on the PICOS framework, incorporating the following key terms: (P) Population—children and adolescents with overweight or obesity; (I) Intervention—exercise or regular physical activity; (C) Comparator—no exercise intervention or maintenance of usual lifestyle; (O) Outcomes—indicators related to body composition, cardiorespiratory fitness, cardiometabolic health, and mental health; and (S) Study type—randomized controlled trials (RCTs). Detailed search strategies for each database are provided in Appendix pages 4–5. In addition, we manually screened relevant reviews and the reference lists of included studies to supplement the electronic search and ensure completeness and comprehensiveness of the literature coverage.

### Eligibility criteria

In this meta-analysis, “article” and “study” were used interchangeably, whereas “trial” referred to the basic unit of analysis. Accordingly, when an article included multiple eligible intervention groups, it was allowed to contribute more than one trial to the meta-analysis. When a study included multiple eligible intervention arms that shared a single comparison group, the number of participants in the shared comparator group was divided equally across the corresponding intervention–control comparisons to avoid unit-of-analysis errors. The inclusion criteria were as follows: (1) study design: RCTs; (2) participants: children and adolescents aged 5–19 years with overweight or obesity. Participants were not restricted by country, ethnicity, or sex, but professional athletes were excluded; (3) intervention: structured exercise programs, conducted alone or in combination with dietary or behavioral interventions; (4) control: no organized exercise or behavioral programs aimed at increasing physical activity; and (5) language: articles published in English or Chinese.

### Study selection

**Fig. 1:**
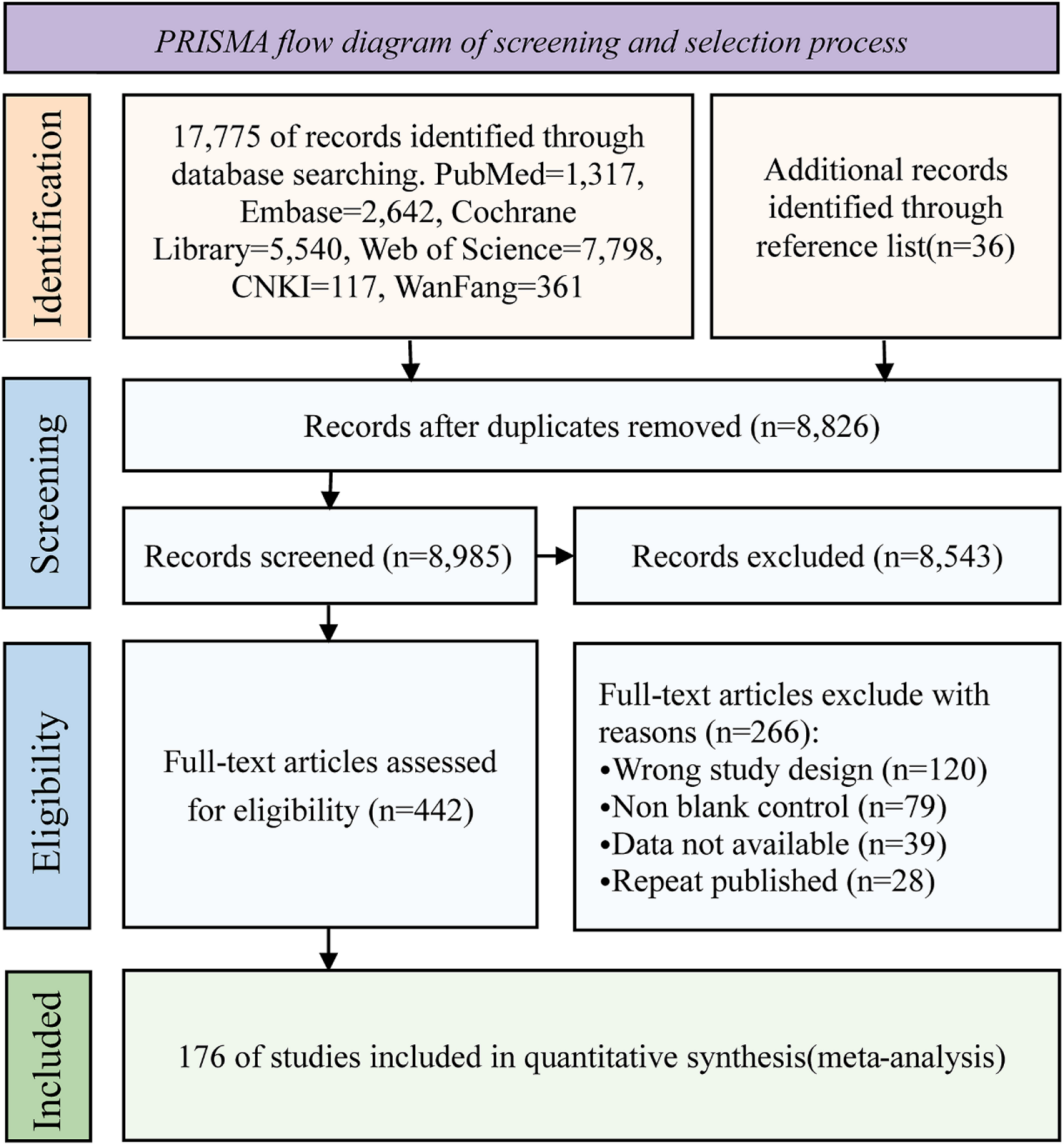
Preferred Reporting Items for Meta-Analyses flow diagram depicting the study selection process

Two independent reviewers (PW and JW) initially screened all retrieved records by titles and abstracts to remove duplicates and clearly irrelevant studies. Subsequently, two reviewers (PW and JW) independently assessed the full texts of studies that passed the initial screening to determine whether they met the predefined inclusion and exclusion criteria. Any disagreements arising during full-text eligibility assessment were resolved through discussion and consensus among all authors. The study selection process is illustrated in Fig. 1.

### Data extraction

Two researchers (PW and JW) independently extracted all data, which were subsequently verified by another team of investigators (JM and a co-investigator). The extracted information included: (1) study characteristics—title, first author, year of publication, and study country or region; (2) participant characteristics—sample size in the intervention and control groups, sex distribution, mean age, and number of participants; (3) intervention characteristics—type, intensity, duration per session, frequency, and total duration of exercise; and (4) post-intervention outcomes—means and standard deviations (SDs) for relevant indicators in both intervention and control groups. Any discrepancies identified during data extraction or verification were resolved through discussion until consensus was reached.

### Outcomes

The included studies were required to report at least one of the following outcome measures: body mass index (BMI), waist circumference (WC), percentage of body fat (BF%), maximal oxygen consumption (VO_2_max), peak oxygen uptake (VO_2_peak), maximal heart rate (HRmax), resting heart rate (HRrest), systolic blood pressure (SBP), diastolic blood pressure (DBP), total cholesterol (TC), triglycerides (TG), high-density lipoprotein cholesterol (HDL-C), low-density lipoprotein cholesterol (LDL-C), fasting plasma glucose (FPG), hemoglobin A1c (HbA1c), fasting insulin (FINS), depression, anxiety, self-esteem, or self-worth.

### Risk of bias

Two researchers (PW and JW) independently assessed the risk of bias for all included studies using the revised Cochrane Risk of Bias Tool for randomized controlled trials (RoB 2.0). Any disagreements were resolved by a third reviewer. The RoB 2.0 tool systematically evaluates bias across five domains: (1) bias arising from the randomization process; (2) bias due to deviations from intended interventions; (3) bias due to missing outcome data; (4) bias in measurement of the outcome; and (5) bias in selection of the reported result [20]. Based on judgments across these domains, each study was classified as having low risk of bias, some concerns, or high risk of bias.

### Certainty of evidence

Two researchers (PW and JW) independently assessed the certainty of evidence for each outcome using the GRADE approach. Any disagreements were resolved through discussion and consensus among all authors. The evaluation considered the following domains: study limitations (risk of bias at the study level), inconsistency (unexplained heterogeneity across studies), indirectness (differences between the study population, interventions, comparators, or outcomes and the research question of interest), imprecision (wide confidence intervals or small sample sizes), and publication bias (selective publication of studies with statistically significant results). The quality of evidence was categorized as high, moderate, low, or very low [19, 21]. Detailed quality assessment scales are provided in Appendix pages 6–7.

### Statistics

We extracted the mean and SD of relevant outcome measures for both intervention and control groups at the end of the intervention. For studies reporting multiple follow-up time points, only data from the final measurement were included in the analysis. When group means and SDs were not directly provided, they were estimated using the formulas recommended in the Cochrane Handbook [22–24]. All extracted data were converted into international standard units: TC, HDL-C, and LDL-C were converted from mg/dL to mmol/l by multiplying by 0.0259, and TG were converted from mg/dL to mmol/l by multiplying by 0.0113. For effect size calculations, mean difference (MD) was used for continuous outcomes measured on the same scale, while standardized mean difference (SMD) was applied for psychological outcomes assessed using different scales. All effect sizes were reported with their 95% confidence intervals (CIs).

We assessed heterogeneity using the *I*^2^ statistic, with thresholds of 25%, 50%, and 75% indicating low, moderate, and high heterogeneity, respectively [25]. Statistical significance was set at α = 0.05. A fixed-effects model was applied when *I*^2^ < 50%, and a random-effects model was used when *I*^2^ ≥ 50% [26]. To explore potential sources of heterogeneity, prespecified subgroup analyses were conducted based on exercise type (aerobic, anaerobic, resistance, and combined aerobic and resistance exercise), intensity (moderate-to-low intensity and high intensity), session duration (< 50 and ≥ 50 min/session), frequency (< 3 and ≥ 3 sessions/week), weekly total duration (< 150 and ≥ 150 min/week), intervention duration (< 12 and ≥ 12 weeks), region (Asia, Europe, Africa, North America, South America, and Oceania), country income level (lower-middle-income countries, upper-middle-income countries, and high-income countries), weight status (overweight and obesity), sex (boys and girls), and metabolic status (metabolically healthy obesity [MHO] and metabolically unhealthy obesity [MUO]). Subgroup classifications were primarily based on definitions reported in the original trials and authors’ descriptions. Tests for subgroup differences in the subgroup analyses were performed using the chi-square test (Cochran’s Q test); for multi-category stratified variables (e.g., exercise type, region, and country income level), no post hoc pairwise comparisons were conducted. Sensitivity analyses were performed using the leave-one-out method: if excluding a single study caused the pooled effect size to fall outside the 95%CI of the overall estimate, that study was considered to have a significant influence on the results [26]. Potential publication bias was evaluated using funnel plots and Egger’s regression test. In cases of asymmetry, the Duval and Tweedie trim-and-fill method (under a random-effects model) was applied to estimate and impute potentially missing studies, and the bias-adjusted pooled effect size was recalculated [27].

All analyses were conducted using Review Manager (RevMan) version 5.4 and R software version 4.5.1. A two-tailed *p* value of less than 0.05 was considered statistically significant.

## Results

### Search selection

As shown in Fig. 1, a total of 17,775 records were identified through database searches, and an additional 36 records were retrieved from the reference lists of relevant systematic reviews. After removing duplicates, 8,985 records were identified through database searches. Following title and abstract screening, 8,543 irrelevant records were excluded, leaving 442 records for full-text assessment. Ultimately, 176 studies were included in the meta-analysis.

### Study and participant characteristics

**Fig. 2:**
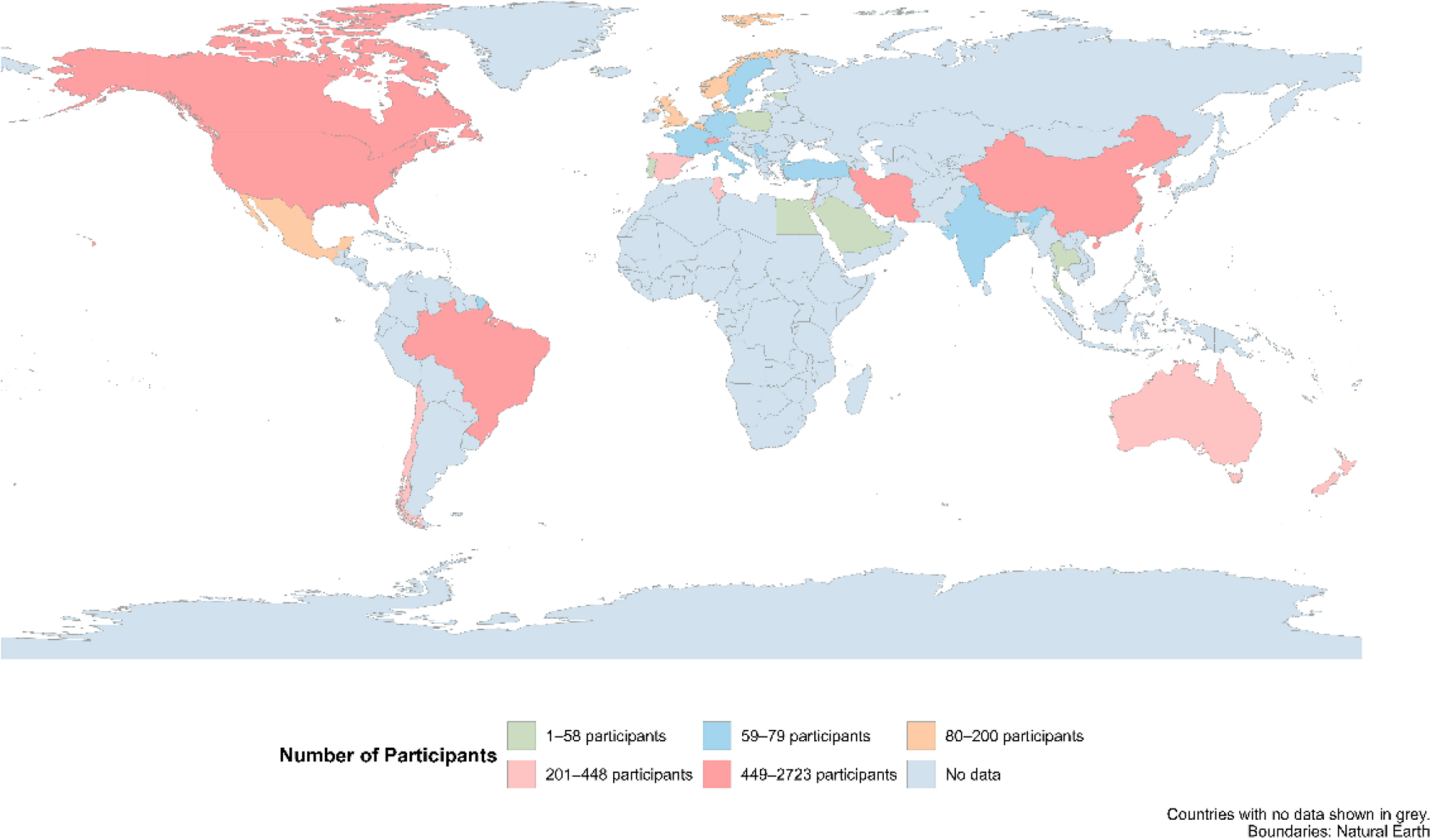
The geographic distribution of the included studies

A total of 176 RCTs involving 11,696 children and adolescents with overweight or obesity were included, comprising 6,778 participants in the intervention groups and 4,918 in the control groups. More than half of the studies (*n* = 89) were published after 2016. Twenty-seven studies included only boys (*n* = 963; 564 in intervention groups and 399 in control groups), and another 27 included only girls (*n* = 1,383; 789 in intervention groups and 594 in control groups). The mean age of participants ranged from 7.7 years (SD 1.2) to 19.8 years (SD 2.5). The duration of exercise interventions varied from 4 weeks to 12 months and primarily fell into four categories: aerobic exercise, anaerobic exercise, resistance training, and combined aerobic–resistance training. Most studies were conducted in high-income countries (*n* = 93), with samples distributed across 32 countries and regions (Fig. 2 and Appendix page 8). Because 34 studies included two intervention groups, eight included three, and one included four, a total of 229 intervention groups were obtained. Detailed study characteristics and raw data are provided in Appendix pages 9–31.

### Meta-analysis results

**Fig. 3:**
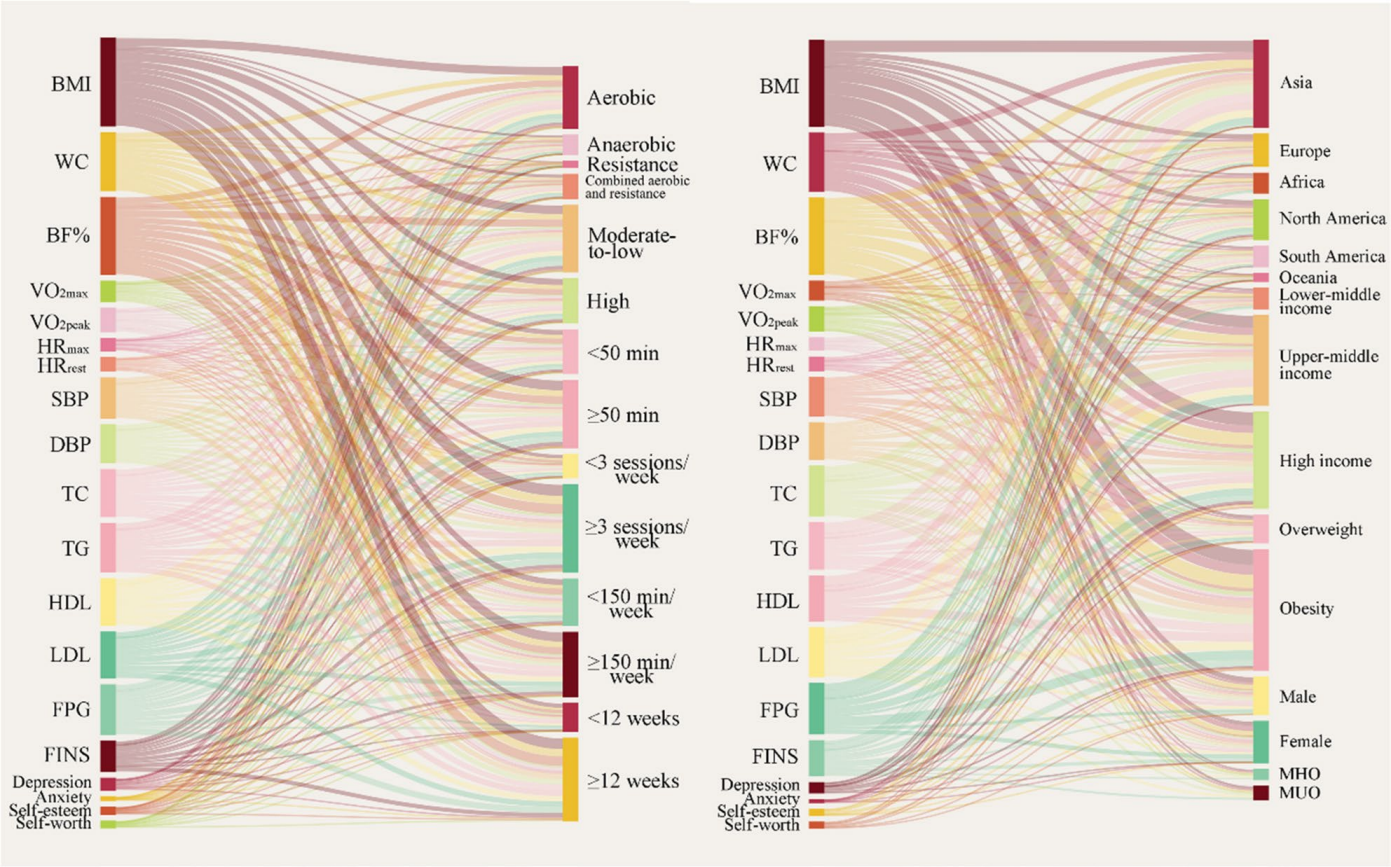
Sankey diagram of outcome measures and subgroup factors. Note: The Sankey diagram illustrates the relationships between outcome measures (left) and prespecified subgroup factors (right) in trials of exercise interventions among children and adolescents with overweight or obesity. Line thickness represents the number of participants contributing to each outcome–subgroup link and does not reflect effect size or statistical significance

The detailed overall and subgroup results of the effects of exercise interventions on body composition, cardiorespiratory fitness, cardiometabolic health, and mental health outcomes among children and adolescents with overweight or obesity, as well as the certainty of evidence, are presented in Table 1 and in Appendix pages 32–56 and 57–146. A Sankey diagram was constructed to illustrate the relationships between outcome indicators and subgroup factors, with the width of each connecting line representing the corresponding sample size (Fig. 3).

**Table 1:** Summary of meta-analysis results of exercise interventions in children and adolescents with overweight or obesity

Outcomes	The number of studies (trials)	The number of participants	Test for overall effect *P*	Heterogeneity I² (%)	Effect model	Effect Size Type	Effect Size [95%Cl]	Egger’s test *P*
BMI (kg m^−2^)	133 (167)	7753	<0.00001**	73	RE	MD	−1.25 [−1.51, −0.98]	0.44
WC (cm)	81 (110)	5442	< 0.00001**	64	RE	MD	−2.86 [−3.52, −2.20]	0.71
BF (%)	112 (146)	6468	< 0.00001**	70	RE	MD	−2.39 [−2.81, −1.96]	0.88
VO_2_max (mL·kg^−1^·min^−1^)	28 (38)	1411	< 0.00001**	77	RE	MD	2.83 [2.17, 3.50]	0.04*
VO_2_peak (mL·kg^−1^·min^−1^)	32 (44)	1862	< 0.00001**	65	RE	MD	2.56 [1.86, 3.27]	0.0002**
HRmax (bpm)	17 (23)	596	0.36	62	RE	MD	−0.52 [−1.63, 0.60]	0.23
HRrest (bpm)	19 (25)	902	< 0.00001**	54	RE	MD	−2.89 [−3.82, −1.97]	0.19
SBP (mmHg)	56 (76)	3823	< 0.00001**	91	RE	MD	−3.90 [−5.34, −2.46]	< 0.00001**
DBP (mmHg)	52 (71)	3525	0.0007**	86	RE	MD	−1.70 [−2.68, −0.71]	0.02*
TC (mmol/l)	73 (89)	4252	< 0.00001**	81	RE	MD	−0.35 [−0.43, −0.27]	0.05
TG (mmol/l)	76 (92)	4865	< 0.00001**	91	RE	MD	−0.23 [−0.28, −0.17]	< 0.00001**
HDL-C (mmol/l)	72 (88)	4119	< 0.00001**	72	RE	MD	0.08 [0.05, 0.10]	0.16
LDL-C (mmol/l)	71 (87)	3940	< 0.00001**	78	RE	MD	−0.28 [−0.35, −0.20]	0.13
FPG (mmol/l)	76 (94)	4642	< 0.00001**	85	RE	MD	−0.20 [−0.26, −0.13]	0.31
HbA1c (%)	12 (14)	588	0.05	15	FE	MD	−0.04 [−0.09, 0.00]	0.48
FINS (µU/mL)	46 (57)	2471	< 0.00001**	76	RE	MD	−3.93 [−4.80, −3.06]	0.24
Depression	16 (22)	1535	0.39	7	FE	SMD	−0.05 [−0.15, 0.06]	0.08
Anxiety	6 (6)	485	0.23	86	RE	SMD	−0.33 [−0.87, 0.21]	0.13
Self-esteem	10 (13)	857	0.01*	19	FE	SMD	0.18 [0.04, 0.33]	0.08
Self-worth	6 (12)	822	0.001**	0	FE	SMD	0.25 [0.10, 0.39]	0.09

*BMI* body mass index, *WC* waist circumference, *BF%* percentage of body fat, *VO*_2_*max* maximal oxygen consumption, *VO*_2_*peak* peak oxygen uptake, *HRmax* max heart rate, *HRrest* resting heart rate, *SBP* systolic blood pressure, *DBP* diastolic blood pressure, *TC* total cholesterol, *TG* triglyceride, *HDL-C* high-density lipoprotein cholesterol, *LDL-C* low-density lipoprotein cholesterol, *FPG* fasting plasma glucose, *HbA1c* hemoglobin A1c, *FINS* fasting insulin, *RE* Random-Effects Model, *FE* Fixed-Effects Model, *MD* Mean Difference, *SMD* Standardized Mean Difference

*P<0.05, **P<0.01

### Body composition

#### BMI

Across 167 RCTs including 7,753 participants, exercise interventions significantly reduced BMI (MD: −1.25 kg m^−2^, 95% CI: −1.51 to −0.98; *P* < 0.00001), with moderate heterogeneity (*I*^2^ = 73%) and moderate certainty of evidence. Subgroup analyses showed statistically significant between-group differences for exercise intensity (*P* = 0.02), exercise frequency (*P* = 0.01), weight status (*P* = 0.02), and metabolic status (*P* = 0.004). Descriptive comparisons suggested that moderate-to-low intensity (compared with high intensity), ≥ 3 sessions per week (compared with < 3 sessions per week), and participants with obesity (compared with those with overweight) or MUO (compared with MHO) had larger pooled point estimates indicating greater improvement. For multi-category stratified variables—including exercise type (*P* = 0.04), region (*P* = 0.004), and country income level (*P* = 0.0004)—tests for subgroup differences likewise suggested overall heterogeneity across categories; however, these tests did not allow identification of the specific categories contributing to the observed differences.

#### WC

Across 110 RCTs including 5,442 participants, exercise interventions significantly reduced WC (MD: −2.86 cm, 95% CI: −3.52 to −2.20; *P* < 0.00001), with moderate heterogeneity (*I*^2^ = 64%) and moderate certainty of evidence. Subgroup analyses showed statistically significant between-group differences for session duration (*P* = 0.02), weekly exercise duration (*P* = 0.006), and weight status (*P* = 0.002). Descriptive comparisons suggested that sessions lasting ≥ 50 min (compared with < 50 min), a weekly total duration of ≥ 150 min (compared with < 150 min per week), and participants with obesity had larger pooled point estimates indicating greater improvement. The test for subgroup differences by country income level was also statistically significant (*P* = 0.01), suggesting overall heterogeneity across income categories.

#### BF%

Across 146 RCTs including 6,468 participants, exercise interventions produced a significant reduction in BF% (MD: −2.39, 95% CI: −2.81 to −1.96; *P* < 0.00001), with moderate heterogeneity (*I*^2^ = 70%) and moderate certainty of evidence. Subgroup analyses showed statistically significant between-group differences for session duration (*P* = 0.0008), weekly exercise duration (*P* = 0.01), and metabolic status (*P* = 0.002). Descriptive comparisons suggested that sessions lasting ≥ 50 min, a weekly total duration of ≥ 150 min, and participants with MUO had larger pooled point estimates indicating greater improvement. Tests for subgroup differences were also statistically significant for region (*P* = 0.0005) and country income level (*P* = 0.0003).

### Cardiorespiratory fitness

#### VO_2_max

Across 38 RCTs including 1,411 participants, exercise interventions significantly improved VO_2_max (MD: 2.83 mL·kg^−1^·min^−1^, 95% CI: 2.17 to 3.50; *P* < 0.00001), with high heterogeneity (*I*^2^ = 77%) and moderate certainty of evidence. Subgroup analyses showed statistically significant between-group differences for region (*P* = 0.01) and country income level (*P* < 0.0001).

#### VO_2_peak

Across 44 RCTs including 1,862 participants, exercise interventions significantly improved VO_2_peak (MD: 2.56 mL·kg^−1^·min^−1^, 95% CI: 1.86 to 3.27; *P* < 0.00001), with moderate heterogeneity (*I*^2^ = 65%) and moderate certainty of evidence. Subgroup analyses showed statistically significant between-group differences for exercise frequency (*P* = 0.03), sex (*P* < 0.00001), and metabolic status (*P* = 0.002). Descriptive comparisons suggested that ≥ 3 sessions per week, boys (compared with girls), and participants with MUO had larger pooled point estimates indicating greater improvement. Tests for subgroup differences were also statistically significant for region (*P* < 0.0001) and country income level (*P* < 0.00001).

#### HRmax

Across 23 RCTs including 596 participants, exercise interventions showed no evidence of an effect on HRmax (MD: −0.52 bpm, 95% CI: −1.63 to 0.60; *P* = 0.36), with moderate heterogeneity (*I*^2^ = 62%) and low certainty of evidence. Subgroup analyses showed statistically significant between-group differences for exercise type (*P* < 0.0001) and country income level (*P* = 0.03).

#### HRrest

Across 25 RCTs including 902 participants, exercise interventions significantly reduced HRrest (MD: −2.89 bpm, 95% CI: −3.82 to −1.97; *P* < 0.00001), with moderate heterogeneity (*I*^2^ = 54%) and high certainty of evidence. Subgroup analyses showed statistically significant between-group differences for session duration (*P* = 0.02) and exercise frequency (*P* = 0.02). Descriptive comparisons suggested that sessions lasting ≥ 50 min or < 3 sessions per week had larger pooled point estimates indicating greater improvement.

### Cardiometabolic health

#### SBP

Across 76 RCTs including 3,823 participants, exercise interventions significantly reduced SBP (MD: −3.90 mmHg, 95% CI: −5.34 to −2.46; *P* < 0.00001), with high heterogeneity (*I*^2^ = 91%) and high certainty of evidence. Subgroup analyses showed statistically significant between-group differences for exercise intensity (*P* = 0.007), session duration (*P* = 0.008), exercise frequency (*P* = 0.01), and weekly exercise duration (*P* = 0.006). Descriptive comparisons suggested that moderate-to-low intensity, sessions lasting ≥ 50 min, ≥ 3 sessions per week, or a weekly total duration of ≥ 150 min had larger pooled point estimates indicating greater improvement. The test for subgroup differences by region was also statistically significant (*P* = 0.01).

#### DBP

Across 71 RCTs including 3,525 participants, exercise interventions significantly reduced DBP (MD: −1.70 mmHg, 95% CI: −2.68 to −0.71; *P* = 0.0007), with high heterogeneity (*I*^2^ = 86%) and moderate certainty of evidence. Subgroup analyses showed that none of the tests for between-group differences in DBP reached statistical significance.

#### TC

Across 89 RCTs including 4,252 participants, exercise interventions significantly reduced TC (MD: −0.35 mmol/l, 95% CI: −0.43 to −0.27; *P* < 0.00001), with high heterogeneity (*I*^2^ = 81%) and moderate certainty of evidence. Subgroup analyses showed statistically significant between-group differences for exercise frequency (*P* = 0.0007), weekly exercise duration (*P* = 0.001), and sex (*P* = 0.01). Descriptive comparisons suggested that ≥ 3 sessions per week, a weekly total duration of ≥ 150 minutes, or boys had larger pooled point estimates indicating greater improvement. Tests for subgroup differences were also statistically significant for region (*P* < 0.0001) and country income level (*P* < 0.0001).

#### TG

Across 92 RCTs including 4,865 participants, exercise interventions significantly reduced TG levels (MD: −0.23 mmol/l, 95% CI: −0.28 to −0.17; *P* < 0.00001), with high heterogeneity (*I*^2^ = 91%) and moderate certainty of evidence. Subgroup analyses showed statistically significant between-group differences for session duration (*P* = 0.002), intervention duration (*P* = 0.04), and sex (*P* = 0.02). Descriptive comparisons suggested that sessions lasting ≥ 50 min, interventions lasting < 12 weeks (compared with ≥ 12 weeks), or boys had larger pooled point estimates indicating greater improvement. Tests for subgroup differences were also statistically significant for region (*P* < 0.0001) and country income level (*P* = 0.0002).

#### HDL-C

Across 88 RCTs including 4,119 participants, exercise interventions significantly increased HDL-C levels (MD: 0.08 mmol/l, 95% CI: 0.05 to 0.10; *P* < 0.00001), with moderate heterogeneity (*I*^2^ = 72%) and low certainty of evidence. Subgroup analyses showed that none of the tests for between-group differences in HDL-C reached statistical significance.

#### LDL-C

Across 87 RCTs including 3,940 participants, exercise interventions significantly reduced LDL-C levels (MD: −0.28 mmol/l, 95% CI: −0.35 to −0.20; *P* < 0.00001), with high heterogeneity (*I*^2^ = 78%) and moderate certainty of evidence. Subgroup analyses showed statistically significant between-group differences for exercise frequency (*P* = 0.0003), weekly exercise duration (*P* = 0.04), and sex (*P* = 0.002). Descriptive comparisons suggested that ≥ 3 sessions per week, a weekly total duration of ≥ 150 min, or boys had larger pooled point estimates indicating greater improvement. Tests for subgroup differences were also statistically significant for region (*P* = 0.01) and country income level (*P* = 0.0008).

#### FPG

Across 94 RCTs including 4,642 participants, exercise interventions significantly reduced FPG (MD: −0.20 mmol/l, 95% CI: −0.26 to −0.13; *P* < 0.00001), with high heterogeneity (*I*^2^ = 85%) and moderate certainty of evidence. Subgroup analyses showed statistically significant between-group differences for exercise frequency (*P* = 0.0007), weekly exercise duration (*P* = 0.001), and weight status (*P* = 0.04). Descriptive comparisons suggested that ≥ 3 sessions per week, a weekly total duration of ≥ 150 min, or participants with obesity had larger pooled point estimates indicating greater improvement. Tests for subgroup differences were also statistically significant for region (*P* < 0.00001) and country income level (*P* = 0.02).

#### HbA1c

Across 14 RCTs including 588 participants, exercise interventions were associated with a small reduction in HbA1c (MD: −0.04, 95% CI: −0.09 to 0.00; *P* = 0.05), with low heterogeneity (*I*^2^ = 15%) and moderate certainty of evidence. Subgroup analyses showed that none of the tests for between-group differences in HbA1c reached statistical significance.

#### FINS

Across 57 RCTs including 2,471 participants, exercise interventions significantly reduced FINS levels (MD: −3.93 µU/mL, 95% CI: −4.80 to −3.06; *P* < 0.00001), with high heterogeneity (*I*^2^ = 76%) and moderate certainty of evidence. Subgroup analyses showed statistically significant between-group differences for exercise intensity (*P* = 0.02), session duration (*P* = 0.02), weight status (*P* = 0.0007), and metabolic status (*P* = 0.004). Descriptive comparisons suggested that moderate-to-low intensity, sessions lasting ≥ 50 min, and participants with obesity or MUO had larger pooled point estimates indicating greater improvement. Tests for subgroup differences were also statistically significant for region (*P* = 0.03) and country income level (*P* = 0.04).

### Mental health

#### Depression

Across 22 RCTs including 1,535 participants, exercise interventions showed no evidence of an effect on depression (SMD: −0.05, 95% CI: −0.15 to 0.06; *P* = 0.39), with low heterogeneity (*I*^2^ = 7%) and moderate certainty of evidence. Subgroup analyses showed a statistically significant between-group difference for session duration (*P* = 0.04). Descriptive comparisons suggested that sessions lasting < 50 min had larger pooled point estimates indicating greater improvement.

#### Anxiety

Across 6 RCTs including 485 participants, exercise interventions showed no evidence of an effect on anxiety (SMD: −0.33, 95% CI: −0.87 to 0.21; *P* = 0.23), with high heterogeneity (*I*^2^ = 86%) and very low certainty of evidence. Subgroup analyses showed that none of the tests for between-group differences in anxiety reached statistical significance.

#### Self-esteem

Across 13 RCTs including 857 participants, exercise interventions significantly improved self-esteem (SMD: 0.18, 95% CI: 0.04 to 0.33; *P* = 0.01), with low heterogeneity (*I*^2^ = 19%) and moderate certainty of evidence. Subgroup analyses showed a statistically significant between-group difference for sex (*P* = 0.01). Descriptive comparisons suggested that boys had larger pooled point estimates indicating greater improvement; however, this finding was based on only two trials [28, 29] and should therefore be interpreted with caution.

#### Self-worth

Across 12 RCTs including 822 participants, exercise interventions significantly improved self-worth (SMD: 0.25, 95% CI: 0.10 to 0.39; *P* = 0.001), with low heterogeneity (*I*^2^ = 0%) and high certainty of evidence. Subgroup analyses showed that none of the tests for between-group differences in self-worth reached statistical significance.

### Risk of bias

Across 229 RCTs, overall risk-of-bias assessment indicated that 50 trials (21.8%) were rated as having a low risk of bias, 168 (73.4%) as having some concerns, and 11 (4.8%) as having a high risk of bias. By domain, 148 trials (64.6%) incurred bias arising from the randomization process; 29 (12.7%) from deviations from intended interventions; 7 (3.1%) from missing data; 116 (50.7%) from outcome measurement; and 122 (53.3%) from selective reporting of results. Detailed domain-level assessments are presented in Appendix pages 148–149.

### Sensitive analysis and publication bias

Sensitivity analyses, performed by systematically excluding one study at a time, indicated that no single study had a substantial influence on the pooled effect size for most outcomes. For HbA1c, the pooled estimate was sensitive to the exclusion of individual studies, with the overall effect reaching statistical significance after omitting either Meyer et al. (2006) [30] or Tjønna et al. (2009) [31], suggesting limited robustness of the pooled result for this outcome. No obvious publication bias was detected for most outcomes, and all estimates remained robust after imputing potentially missing studies. Because only six studies were included for the anxiety outcome, the statistical power of the Egger’s test was limited, and the results should be interpreted with caution. Funnel plots, along with the results of Egger’s regression tests and the trim-and-fill analyses (including adjusted funnel plots), are presented in Appendix pages 142–146 and 150–156.

### Certainty of evidence (GRADE assessment)

Quality of evidence for all outcomes was assessed using the GRADE approach. Overall, the certainty of evidence across outcomes ranged from high to very low. The certainty of evidence was rated as high for HRrest, SBP, and self-worth; low for HRmax and HDL-C; and very low for anxiety-related outcomes, while the remaining outcomes were rated as moderate. Downgrading of the evidence was mainly driven by risk of bias, inconsistency, and imprecision, and some outcomes were also affected by potential publication bias. Detailed GRADE assessments for each outcome are provided in Appendix page 147.

## Discussion

A total of 176 RCTs involving 11,696 children and adolescents with overweight or obesity across 32 countries and regions were included in this study. To our knowledge, this is the largest and most comprehensive meta-analysis to date systematically evaluating the effects of exercise interventions on 20 multidimensional health outcomes in this population, encompassing body composition, cardiorespiratory fitness, cardiometabolic health, and mental health outcomes. The results demonstrated that exercise interventions significantly improved body composition (BMI, WC, BF%), enhanced cardiorespiratory fitness (VO_2_max, VO_2_peak, HRrest), and benefited cardiometabolic (SBP, DBP, TC, TG, HDL-C, LDL-C, FPG, FINS) and mental outcomes (self-esteem, self-worth). However, the evidence for improvements in HRmax, HbA1c, depression, and anxiety was inconclusive. Although clinical heterogeneity was relatively high, sensitivity analyses confirmed the robustness of the findings, and most indicators showed no significant publication bias. The certainty of evidence ranged from moderate to high. Subgroup analyses suggested that the magnitude of improvement may have varied across categories of exercise dose and type, region, country income level, sex, and obesity/metabolic phenotype. Given that real-world complexity extends beyond our subgroup classifications, these findings should be interpreted cautiously within the clinical context. Specifically, exercise programmes characterised by sessions lasting ≥ 50 min, performed ≥ 3 times per week or accumulating ≥ 150 min per week, and primarily delivered at moderate-to-low intensity showed overall more favourable pooled point estimates across most health outcomes. In addition, participants with obesity—particularly those with MUO—and boys demonstrated greater improvements in some outcomes. Notably, geographic region and country income level, as multi-category stratified variables, yielded statistically significant tests for subgroup differences across the majority of outcomes. Although the specific sources of these differences could not be identified, both factors may reflect important macro-level contextual effect modifiers and provide potential insight into between-study heterogeneity. These findings provide robust evidence to inform the design of exercise interventions and the development of public health policies aimed at curbing obesity and promoting health equity among children and adolescents with overweight or obesity.

Consistent with the findings of Stoner et al. [32], our study confirmed that exercise interventions effectively improve body composition in children and adolescents with overweight or obesity. However, unlike Stoner et al., who did not observe significant changes in cardiometabolic markers, the present study further demonstrated clear benefits of exercise in lowering blood pressure and improving glucose and lipid metabolism, providing more compelling evidence for disease prevention and risk management. Similarly, regarding improvements in cardiorespiratory fitness, our conclusions are consistent with those of Wang et al. [33], reinforcing the central role of exercise interventions in enhancing cardiopulmonary health. It is noteworthy that our study found no significant effect of exercise on HRmax, which is consistent with previous evidence indicating that HRmax is primarily determined by age and genetic factors and represents a relatively stable physiological upper limit that is difficult to modify through training [34–36]. Based on current evidence, HRmax may therefore serve better as a reference for exercise prescription intensity rather than as an indicator of cardiorespiratory fitness. In terms of psychological and mental health, our findings were not entirely consistent with those of Zhou et al. [14], which may be attributed to the complexity of psychological outcomes and variations in assessment instruments. Beyond the evidence level, policy remains another crucial determinant in achieving global obesity control targets. Given that policy implementation is closely linked to national and economic contexts, we conducted subgroup analyses by region and income level to provide an evidence base for developing more targeted public health strategies across different settings.

Subgroup analyses suggested that the effects of exercise interventions may vary according to exercise dose–related characteristics. Specifically, exercise regimens consisting of sessions lasting 50 min or longer, performed at least three times per week, or accumulating at least 150 min per week demonstrated more pronounced improvements in body composition, blood pressure, and glucose–lipid metabolism. Although these findings are not entirely consistent with WHO guidelines, both emphasize the importance of “adequate exercise dose” in achieving health benefits. It is worth noting that the WHO recommendations target the general pediatric population, whereas the present study focuses on children and adolescents with overweight or obesity, which may explain the differences in recommended frameworks. With respect to exercise type, this multi-category stratified variable suggested overall subgroup differences for some outcomes; however, the test for subgroup differences indicates only that at least one category differs from another. As no pairwise comparisons were performed, it is not possible to determine whether any specific exercise modality is superior to others. Accordingly, our interpretation focuses primarily on biological plausibility. Combined aerobic and resistance exercise may exert complementary effects across multiple physiological pathways. Specifically, aerobic exercise may promote fat oxidation and enhance mitochondrial function [37], whereas resistance training may increase skeletal muscle mass and upregulate GLUT4 expression, thereby improving glucose uptake and insulin sensitivity [38–40]. Together, these mechanisms may confer more favourable effects on body composition and a range of cardiometabolic risk markers. In contrast, anaerobic exercise appeared to be associated with greater improvements in VO_2_max and VO_2_peak, which may be attributable to both central adaptations (enhanced cardiac pump function and autonomic regulation [41, 42]) and peripheral adaptations (skeletal muscle mitochondrial remodeling and endothelial function improvement [43–45]). For mental health outcomes, short-duration and high-frequency exercise patterns may help enhance self-esteem and self-worth by repeatedly fostering manageable success experiences and positive emotional feedback. However, evidence regarding the effects of exercise on core mental health indicators such as depression and anxiety remains inconsistent, highlighting the need for future studies with longer intervention periods and standardized assessment tools.

This study found that intervention effects may be influenced by baseline weight status and metabolic phenotype. When stratified by weight status, tests for subgroup differences were statistically significant for BMI, WC, FPG, and FINS, and descriptive comparisons suggested that participants with obesity had larger pooled point estimates indicating greater improvement. Further stratification by metabolic status showed that participants with MUO had larger pooled point estimates indicating greater improvement in BMI, BF%, VO_2_peak, and FINS. In contrast, participants with MHO did not demonstrate a consistent pattern of improvement across these outcomes. These findings suggest that exercise may hold greater clinical value for high-risk subgroups, with effect differences potentially related to metabolic status and baseline risk characteristics. However, the long-term preventive and health-promoting effects of exercise among low-risk populations should not be overlooked [46, 47]. Meanwhile, a modest reduction in HbA1c—reflecting long-term glycaemic control—was also observed, approaching statistical significance. However, sensitivity analyses indicated that the pooled estimate was influenced by individual studies, suggesting limited robustness of the current evidence. These findings should therefore be interpreted with caution and warrant confirmation in further trials. Sex-stratified analyses showed statistically significant subgroup differences for VO_2_peak, TC, LDL-C, and some psychological outcomes. Overall, descriptive comparisons suggested that boys had larger pooled point estimates indicating greater improvement in cardiorespiratory fitness and lipid-related outcomes. These differences are likely due to physiological characteristics during puberty: increases in muscle mass and cardiac pump function in boys lead to higher maximal oxygen uptake [48], while reductions in blood lipid levels—particularly TG and LDL-C—occur predominantly in boys [49, 50], thereby reinforcing their advantage in cardiorespiratory and lipid improvements. With respect to mental outcomes, although the overall evidence for the effects of exercise on depression and anxiety remains limited, a potential improvement in depression was observed in the subgroup with session durations of < 50 min. In addition, some subgroup findings (e.g., improvement in anxiety among girls) may have been driven by single trials [51] and therefore require further confirmation. Taken together, future research and clinical practice should more fully consider obesity phenotypes, metabolic status, and sex differences to inform the development of more precise exercise prescriptions.

This study further suggests that social and geographic contexts may be associated with the effects of exercise interventions. As multi-category stratified variables, region and country income level yielded statistically significant tests for subgroup differences across most outcomes, indicating overall effect heterogeneity. Such heterogeneity may be related to macro-level contextual factors, including baseline risk profiles, accessibility and adherence to interventions, lifestyle environments, and the allocation of healthcare and public resources. The underlying mechanisms require further investigation through prespecified interaction analyses and contextual factor assessments in future research. Future studies should also seek to identify the macro-level conditions under which exercise interventions are most effective, in order to better inform context-specific intervention design and implementation. Although exercise interventions may offer a potentially scalable approach, their costs and feasibility are likely to vary across settings and modes of delivery. These findings underscore the need for more targeted implementation research across diverse geographic and socioeconomic settings to support the systematic integration of structured exercise into strategies for the prevention and management of overweight and obesity in children and adolescents.

Over the past several decades, the world has faced a paradox in public health: despite the continuous accumulation of scientific evidence supporting the effectiveness of obesity interventions, obesity rates have continued to rise globally. This contradiction underscores the persistent barriers in translating evidence into policy. These challenges are partly linked to methodological limitations—such as risk of bias, heterogeneity, and publication bias—that lead to downgrading in the GRADE system. Our findings corroborate this, with the quality of evidence for 13 outcome indicators rated as moderate owing to methodological constraints. However, methodological limitations alone do not fully explain the stagnation in policy translation. The deeper driving forces lie within the realm of political economy, as illustrated by the pervasive phenomenon of policy inertia [52]. Although global policies to combat the obesity epidemic have been established, lobbying by interest groups—particularly those opposing taxation on sugar-sweetened beverages and high-energy foods—has influenced legislation and weakened regulatory frameworks, substantially impeding policy implementation [53]. At the same time, the public has not fully recognized that obesity, while classified as a chronic non-communicable disease, should also be regarded as an urgent public-health concern—further undermining policy demand and political will. Consequently, many policies remain largely declarative, in stark contrast to the decisive global response once seen in addressing Acquired Immune Deficiency Syndrome [54]. Moreover, focusing solely on treatment approaches targeting individuals or groups with obesity may inadvertently reinforce assumptions of personal responsibility, prejudice, and stigma [55]. Combined with the inevitable shift of the obesity burden toward populations with lower socioeconomic status [56], this dynamic risks deepening existing health inequities. Given the critical role of policy in shaping global epidemics, these findings carry profound implications for health equity and further substantiate the rationale for prioritizing exercise interventions within public-health strategies.

### Strengths and limitations

This study provides the most comprehensive evidence to date on the effects of exercise interventions in improving outcomes among children and adolescents with overweight or obesity, adhering rigorously to the PRISMA guidelines to ensure the validity and reliability of the results. Nevertheless, several limitations should be acknowledged. Considerable heterogeneity existed across studies due to variations in participant characteristics (e.g., baseline status, age), intervention formats, and definitions of overweight and obesity. To address this, subgroup analyses were conducted based on exercise dose and type, region, country income level, sex, and obesity/metabolic phenotype. As subgroup comparisons are inherently observational, they are susceptible to confounding by other study-level characteristics (such as intervention duration, exercise intensity, and length of follow-up). Therefore, subgroup findings should be interpreted with caution and regarded as exploratory rather than causal or definitive conclusions. We selected fixed-effect or random-effects models based on an *I*² threshold. Although this approach is commonly used, the choice of an *I*² cut-off is inherently empirical and subject to methodological limitations; thus, the results should also be interpreted cautiously. Moreover, the relatively short intervention duration and lack of follow-up assessments precluded evaluation of the long-term effects of exercise. In addition, most included studies were conducted in middle- and high-income countries, with limited representation from low-income settings, where cautious extrapolation is warranted. Although the review incorporated data from 32 countries and regions worldwide, the inclusion was restricted to studies published in English and Chinese, which may introduce language bias. Furthermore, the number of studies examining mental outcomes was limited, and the measurement tools varied substantially, warranting cautious interpretation. Finally, due to data access and resource constraints, we were unable to perform computational modeling to explore policy-related determinants and identify key barriers to policy formulation and implementation.

## Conclusions

In conclusion, exercise interventions produce significant improvements across multiple dimensions of physical and mental health among children and adolescents with overweight or obesity. These findings are supported by sensitivity analyses, publication bias assessments, and subgroup analyses. However, the effects of exercise are substantially influenced by intervention characteristics—such as exercise types and dosage parameters, and by population factors including region, sex, and obesity/metabolic phenotype, with particularly greater benefits observed among high-risk subgroups. Nevertheless, the continued global rise in obesity rates does not stem from a lack of effective interventions. On the contrary, both previous evidence and our findings confirm that exercise interventions are effective, safe, low-cost, and broadly applicable. The challenge lies in the limited translation of evidence into tangible public health outcomes—a pressing global issue. Future efforts should operate on two fronts. First, societies, schools, and communities should be further encouraged to implement exercise-based intervention programs to achieve broader health gains. Second, it is essential to move beyond evidence synthesis and address the upstream determinants of policy-making—particularly those rooted in political economy—to establish robust global governance strategies capable of overcoming policy inertia and translating scientific evidence into actionable public health policies.

## Supplementary Information

Supplementary Material 1.

Supplementary Material 2.

## Data Availability

All data generated or analysed during this study are included in this published article [and its supplementary information files].

## Abbreviations

WHO: World Health Organization
NCDs: Non-communicable diseases
COVID-19: Coronavirus disease 2019
BMI: Body mass index
WC: Waist circumference
BF%: Body fat percentage
VO_2_max: Maximal oxygen consumption
VO_2_peak: Peak oxygen uptake
HRmax: Maximal heart rate
HRrest: Resting heart rate
SBP: Systolic blood pressure
DBP: Diastolic blood pressure
TC: Total cholesterol
TG: Triglycerides
HDL-C: High-density lipoprotein cholesterol
LDL-C: Low-density lipoprotein cholesterol
FPG: Fasting plasma glucose
HbA1c: Hemoglobin A1c
FINS: Fasting insulin
GRADE: Grading of Recommendations, Assessment, Development and Evaluation
RCTs: Randomized controlled trials
CNKI: China National Knowledge Infrastructure
PRISMA: Preferred Reporting Items for Systematic Reviews and Meta-Analyses
PROSPERO: Prospective Register of Systematic Reviews
MD: Mean difference
CI: Confidence interval
I^2^: I-squared statistic
RE: Random-effects model
FE: Fixed-effects model
ROB: Risk of bias
RevMan: Review Manager
MHO: Metabolically healthy obesity
MUO: Metabolically unhealthy obesity
GLUT4: Glucose transporter type 4
