# Supplementary material for: Effects of exercise on multiple health outcomes in children and adolescents with overweight or obesity: a meta-analysis of 176 randomized controlled trials and its implications for global obesity prevention
Source: Int J Behav Nutr Phys Act. 2026 Apr 17;23:55. doi: 10.1186/s12966-026-01913-0 (PMC13224709; doi:10.1186/s12966-026-01913-0)
Supplement: Supplementary file 1 — Supplementary Material 1. [file 12966_2026_1913_MOESM1_ESM.pdf]

Supplementary Appendix

Effects of exercise on multiple health outcomes in children and adolescents with overweight or obesity: a meta-analysis of 176 randomized controlled trials and its implications for global obesity prevention

Jie Men[a, d, *], Pengbo Wang[a, d], Jingwen Wang[a, d], Guoyu Zhu[a], Zhengyang Yu[a], Simin Wu[a], Yuxi Zhang[a], Weiqi An[a], Zhaowei Li[a], Rui Ma[a], Ruiqi Zhang[a], Shufeng Li[a], Yaoyong Wang[b], Penghong Liu[c]

[a] Shanxi University of Medicine, Fenyang 032200, China

[b] Department of Respiratory and Critical Care Medicine, Fenyang Hospital of Shanxi Province, Fenyang 032200, China

[c] First Hospital of Shanxi Medical University, Taiyuan 030001, China

Contents

Appendix 1. Search strategy used in each database **4**

Appendix 2. Methodological details for risk of bias assessment and certainty assessment **6**

Appendix 3. Distribution of included studies and participants by country (32 countries, 176 studies, 11 696 participants) **8**

Appendix 4. Supplementary tables and figures **9**

Table S1. The detailed basic characteristics of the included studies(N=176) ***9***

Table S2. Summary of subgroup analyses of meta-analysis results of exercise interventions in children and adolescents with overweight or obesity ***32***

Figure S1. Forest plots of meta-analysis results for 20 outcome indicators in children and adolescents with overweight or obesity ***57***

Figure S2. Forest plots of subgroup analyses for BMI in children and adolescents with overweight or obesity . ***64***

Figure S3. Forest plots of subgroup analyses for WC in children and adolescents with overweight or obesity ... ***69***

Figure S4. Forest plots of subgroup analyses for BF% in children and adolescents with overweight or obesity ***74***

Figure S5. Forest plots of subgroup analyses for VO_2max in children and adolescents with overweight or obesity ***79***

Figure S6. Forest plots of subgroup analyses for VO_2peak in children and adolescents with overweight or obesity ***82***

Figure S7. Forest plots of subgroup analyses for HRmax in children and adolescents with overweight or obesity ***85***

Figure S8. Forest plots of subgroup analyses for HRrest in children and adolescents with overweight or obesity ***88***

Figure S9. Forest plots of subgroup analyses for SBP in children and adolescents with overweight or obesity .. ***91***

Figure S10. Forest plots of subgroup analyses for DBP in children and adolescents with overweight or obesity ***96***

Figure S11. Forest plots of subgroup analyses for TC in children and adolescents with overweight or obesity ***101***

Figure S12. Forest plots of subgroup analyses for TG in children and adolescents with overweight or obesity ***106***

Figure S13. Forest plots of subgroup analyses for HDL-C in children and adolescents with overweight or obesity ***111***

Figure S14. Forest plots of subgroup analyses for LDL-C in children and adolescents with overweight or obesity ***116***

Figure S15. Forest plots of subgroup analyses for FPG in children and adolescents with overweight or obesity ***121***

Figure S16. Forest plots of subgroup analyses for HbA1c in children and adolescents with overweight or obesity ***126***

Figure S17. Forest plots of subgroup analyses for FINS in children and adolescents with overweight or obesity ***128***

Figure S18. Forest plots of subgroup analyses for Depression in children and adolescents with overweight or obesity ***133***

Figure S19. Forest plots of subgroup analyses for Anxiety in children and adolescents with overweight or obesity ***136***

Figure S20. Forest plots of subgroup analyses for Self-esteem in children and adolescents with overweight or obesity ***138***

Figure S21. Forest plots of subgroup analyses for Self-worth in children and adolescents with overweight or obesity ***140***

Figure S22. Funnel plots of meta-analysis results for 20 outcome indicators in children and adolescents with overweight or obesity *142*
Table S3. Certainty of assessment using GRADE for RCTs *147*
Figure S23. Revised Cochrane Risk-of-Bias Tool for Randomised Trials summary and author judgments of low, some concerns, and high risk of bias across all included RCTs (K=229) *148*
Figure S24. Review judgment of risk bias for each domain: percentages across all included RCTs (K=229) *149*
Table S4. Egger's test and Trim-and-Fill correction analyses for publication bias across outcomes *150*
Figure S25. Trim-and-fill funnel plots of meta-analysis results for 20 outcome indicators in children and adolescents with overweight or obesity *152*
Appendix 5. List of all included studies in the meta-analysis **157**

Appendix 1. Search strategy used in each database

Database	Search terms	
**PubMed**	#1	search ("adolescent"[MeSH Terms] OR "children"[Title/Abstract] OR "youngster"[Title/Abstract] OR "teenager"[Title/Abstract] OR "kid"[Title/Abstract] OR "youth"[Title/Abstract] OR "student"[Title/Abstract])
	#2	search ("obesity"[MeSH Terms] OR "adiposity"[Title/Abstract] OR "overweight"[Title/Abstract] OR "fat"[Title/Abstract] OR "body mass index"[Title/Abstract])
	#3	search ("exercise"[MeSH Terms] OR "sport"[Title/Abstract] OR "physical activity"[Title/Abstract] OR "physical training"[Title/Abstract] OR "exercise training"[Title/Abstract] OR "aerobic exercise"[Title/Abstract] OR "resistance training"[Title/Abstract] OR "acute exercise"[Title/Abstract] OR "endurance exercise"[Title/Abstract])
	#4	search ("body mass index"[MeSH Terms] OR "waist circumference"[MeSH Terms] OR "body composition"[MeSH Terms] OR "oxygen consumption"[MeSH Terms] OR "heart rate"[MeSH Terms] OR "blood pressure"[MeSH Terms] OR "blood pressure determination"[MeSH Terms] OR "arterial pressure"[MeSH Terms] OR "cholesterol"[MeSH Terms] OR "triglycerides"[MeSH Terms] OR "glucose"[MeSH Terms] OR "insulin"[MeSH Terms] OR "depressive disorder"[MeSH Terms] OR "depression"[MeSH Terms] OR "anxiety"[MeSH Terms] OR "self concept"[MeSH Terms] OR "self-esteem"[MeSH Terms] OR "self-worth"[Title/Abstract])
	#5	Search (#1 AND #2 AND #3 AND #4)
**Embase**	#1	'adolescent'/exp OR 'adolescent':ab,ti OR 'children':ab,ti OR 'youngster':ab,ti OR 'teenager':ab,ti OR 'kid':ab,ti OR 'youth':ab,ti OR 'student':ab,ti
	#2	'obesity'/exp OR 'obesity':ab,ti OR 'adiposity':ab,ti OR 'overweight':ab,ti OR 'fat':ab,ti OR 'body mass index':ab,ti
	#3	'exercise'/exp OR 'exercise':ab,ti OR 'sport':ab,ti OR 'physical activity':ab,ti OR 'physical training':ab,ti OR 'exercise training':ab,ti OR 'aerobic exercise':ab,ti OR 'resistance training':ab,ti OR 'acute exercise':ab,ti OR 'endurance exercise':ab,ti
	#4	'body mass index'/exp OR 'body mass index':ab,ti OR 'waist circumference'/exp OR 'waist circumference':ab,ti OR 'body composition'/exp OR 'body composition':ab,ti OR 'oxygen consumption'/exp OR 'oxygen consumption':ab,ti OR 'heart rate'/exp OR 'heart rate':ab,ti OR 'blood pressure'/exp OR 'blood pressure':ab,ti OR 'blood pressure determination'/exp OR 'blood pressure determination':ab,ti OR 'arterial pressure'/exp OR 'arterial pressure':ab,ti OR 'cholesterol'/exp OR 'cholesterol':ab,ti OR 'triglycerides'/exp OR 'triglycerides':ab,ti OR 'glucose'/exp OR 'glucose':ab,ti OR 'insulin'/exp OR 'insulin':ab,ti OR 'depressive disorder'/exp OR 'depressive disorder':ab,ti OR 'depression'/exp OR 'depression':ab,ti OR 'anxiety'/exp OR 'anxiety':ab,ti OR 'self concept'/exp OR 'self concept':ab,ti OR 'self esteem'/exp OR 'self esteem':ab,ti OR 'self worth'/exp OR 'self worth':ab,ti
	#5	#1 AND #2 AND #3 AND #4
**Cochrane**	#1	(Adolescent):MeSH OR (adolescent):ti,ab,kw OR (children):ti,ab,kw OR (youngster):ti,ab,kw OR (teenager):ti,ab,kw OR (kid):ti,ab,kw OR (youth):ti,ab,kw OR (student):ti,ab,kw
	#2	(Obesity):MeSH OR (obesity):ti,ab,kw OR (adiposity):ti,ab,kw OR (overweight):ti,ab,kw OR (fat):ti,ab,kw OR (body mass index):ti,ab,kw
	#3	(Exercise):MeSH OR (exercise):ti,ab,kw OR (sport):ti,ab,kw OR (physical activity):ti,ab,kw OR (physical training):ti,ab,kw OR (exercise training):ti,ab,kw OR (aerobic exercise):ti,ab,kw OR (resistance training):ti,ab,kw OR (acute exercise):ti,ab,kw OR (endurance exercise):ti,ab,kw
	#4	(Body mass index):MeSH OR (body mass index):ti,ab,kw OR (Waist circumference):MeSH OR (waist circumference):ti,ab,kw OR (Body composition):MeSH OR (body composition):ti,ab,kw OR (Oxygen consumption):MeSH OR (oxygen consumption):ti,ab,kw OR (Heart rate):MeSH OR (heart rate):ti,ab,kw OR (Blood pressure):MeSH OR (blood pressure):ti,ab,kw OR (Blood pressure determination):MeSH OR (blood pressure determination):ti,ab,kw OR (Arterial pressure):MeSH OR (arterial pressure):ti,ab,kw OR (Cholesterol):MeSH OR (cholesterol):ti,ab,kw OR (Triglycerides):MeSH OR (triglycerides):ti,ab,kw OR (Glucose):MeSH OR (glucose):ti,ab,kw OR (Insulin):MeSH OR (insulin):ti,ab,kw OR (Depressive disorder):MeSH OR (depressive disorder):ti,ab,kw OR (Depression):MeSH OR (depression):ti,ab,kw OR (Anxiety):MeSH OR (anxiety):ti,ab,kw OR (Self concept):MeSH OR (self concept):ti,ab,kw OR (Self esteem):MeSH OR (self esteem):ti,ab,kw OR (Self worth):MeSH OR (self worth):ti,ab,kw
	#5	#1 AND #2 AND #3 AND #4

Database		Search terms
**Web of Science**	#1	(TS=(adolescent) OR TS=(children) OR TS=(youngster) OR TS=(teenager) OR TS=(kid) OR TS=(youth) OR TS=(student)) NOT (SILOID==("PPRN") OR SILOID==("RC"))
	#2	(TS=(obesity) OR TS=(adiposity) OR TS=(overweight) OR TS=(fat) OR TS=(body mass index)) NOT (SILOID==("PPRN") OR SILOID==("RC"))
	#3	(TS=(exercise) OR TS=(sport) OR TS=(physical activity) OR TS=(physical training) OR TS=(exercise training) OR TS=(aerobic exercise) OR TS=(resistance training) OR TS=(acute exercise) OR TS=(endurance exercise)) NOT (SILOID==("PPRN") OR SILOID==("RC"))
	#4	(TS=(body mass index) OR TS=(waist circumference) OR TS=(body composition) OR TS=(oxygen consumption) OR TS=(heart rate) OR TS=(blood pressure) OR TS=(blood pressure determination) OR TS=(arterial pressure) OR TS=(cholesterol) OR TS=(triglycerides) OR TS=(glucose) OR TS=(insulin) OR TS=(depressive disorder) OR TS=(depression) OR TS=(anxiety) OR TS=(self concept) OR TS=(self-esteem) OR TS=(self-worth)) NOT (SILOID==("PPRN") OR SILOID==("RC"))
	#5	(TS=(clinical trials) OR TS=(randomized controlled trial) OR TS=(controlled clinical trial) OR TS=(randomized) OR TS=(placebo) OR TS=(randomly)) NOT (SILOID==("PPRN") OR SILOID==("RC"))
	#6	#1 AND #2 AND #3 AND #4 AND #5
**CNKI**		(SU="儿童青少年" OR SU="儿童" OR SU="青少年") AND (SU="运动" OR SU="有氧运动" OR SU="体育运动" OR SU="身心锻炼" OR SU="力量训练" OR SU="抗阻运动" OR SU="急性运动" OR SU="体育锻炼") AND (SU="肥胖" OR SU="超重") AND (SU="身体形态" OR SU="身体成分" OR SU="心肺机能" OR SU="心肺适能" OR SU="心肺功能" OR SU="有氧能力" OR SU="心肺耐力" OR SU="生化指标" OR SU="血脂代谢" OR SU="代谢" OR SU="脂蛋白" OR SU="胆固醇" OR SU="高密度脂蛋白" OR SU="低密度脂蛋白" OR SU="空腹血糖" OR SU="心理健康" OR SU="心理亚健康" OR SU="身心健康" OR SU="焦虑" OR SU="抑郁" OR SU="情绪" OR SU="自尊" OR SU="自我价值")
**Wanfang Data**		(主题:"儿童青少年" or 主题:"儿童" or 主题:"青少年") and (主题:"运动" or 主题:"有氧运动" or 主题:"体育运动" or 主题:"身心锻炼" or 主题:"力量训练" or 主题:"抗阻运动" or 主题:"急性运动" or 主题:"体育锻炼") and (主题:"肥胖" or 主题:"超重") and (主题:"身体形态" or 主题:"身体成分" or 主题:"心肺机能" or 主题:"心肺适能" or 主题:"心肺功能" or 主题:"有氧能力" or 主题:"心肺耐力" or 主题:"生化指标" or 主题:"血脂代谢" or 主题:"代谢" or 主题:"脂蛋白" or 主题:"胆固醇" or 主题:"高密度脂蛋白" or 主题:"低密度脂蛋白" or 主题:"空腹血糖" or 主题:"心理健康" or 主题:"心理亚健康" or 主题:"身心健康" or 主题:"焦虑" or 主题:"抑郁" or 主题:"情绪" or 主题:"自尊" or 主题:"自我价值")

Access Date: 10 January 2025

Appendix 2. Methodological details for risk of bias assessment and certainty assessment

Risk of bias assessment

We adhered to the comprehensive guidelines provided by the Cochrane Risk of Bias Tool for Randomised Trials (RoB 2) to evaluate the risk of bias across each outcome in the included studies. Utilizing response options: Yes (Y), Probably yes (PY), Probably no (PN), No (N), and No information (NI), we responded to the signalling questions within each domain of risk of bias to reach an overall risk-of-bias judgment for each domain (see below) The responses to the signalling questions feed into algorithms that were developed to guide users of the tools to proposed judgments about the overall risk-of-bias judgment for each domain. The risk-of-bias assessments were categorized into the following levels: low risk, some concerns, and high risk.

Risk of bias domains and signalling questions according to RoB 2:

(1) Randomisation process

(1.1) Was the allocation sequence random?

(1.2) Was the allocation sequence concealed until participants were enrolled and assigned to interventions?

(1.3) Did baseline differences between intervention groups suggest a problem with the randomisation process?

(2) Deviations from intended interventions (effect of assignment to intervention/ITT)

(2.1) Were participants aware of their assigned intervention during the trial?

(2.2) Were carers and people delivering the interventions aware of participants' assigned intervention during the trial?

(2.3) If Y/PY/NI to 2.1 or 2.2: Were there deviations from the intended intervention that arose because of the trial context?

(2.4) If Y/PY to 2.3: Were these deviations likely to have affected the outcome?

(2.5) If Y/PY/NI to 2.4: Were these deviations from intended intervention balanced between groups? (2.6) Was an appropriate analysis used to estimate the effect of assignment to intervention?

(2.7) If N/PN/NI to 2.6: Was there potential for a substantial impact (on the result) of the failure to analyse participants in the group to which they were randomised?

(2) Deviations from intended interventions (effect of adhering to intervention/PP)

(2.1) Were participants aware of their assigned intervention during the trial?

(2.2) Were carers and people delivering the interventions aware of participants' assigned intervention during the trial?

(2.3) Were there failures in implementing the intervention that could have affected the outcome?

(2.4) Y/PY/NI to 2.3: Was an appropriate analysis used to estimate the effect of adhering to intervention?

(3) Missing outcome data

(3.1) Were data for this outcome available for all, or nearly all, participants randomised?

(3.2) If N/PN/NI to 3.1: Is there evidence that the result was not biased by missing outcome data? (3.3) If N/PN to 3.2: Could missingness in the outcome depend on its true value?

(3.4) If Y/PY/NI to 3.3: Is it likely that missingness in the outcome depended on its true value?

(4) Measurement of the outcome

(4.1) Was the method of measuring the outcome inappropriate?
(4.2) Could measurement or ascertainment of the outcome have differed between intervention groups?
(4.3) If N/PN/NI to 4.1 and 4.2: Were outcome assessors aware of the intervention received by study participants?
(4.4) If Y/PY/NI to 4.3: Could assessment of the outcome have been influenced by knowledge of intervention received?
(4.5) If Y/PY/NI to 4.4: Is it likely that assessment of the outcome was influenced by knowledge of intervention received?

(5) Selection of the reported result

(5.1) Were the data that produced this result analysed in accordance with a pre-specified analysis plan that was finalised before unblinded outcome data were available for analysis?
(5.2) Is the numerical result being assessed likely to have been selected, on the basis of the results, frommultiple eligible outcome measurements (e.g., scales, definitions, time points) within the outcome domain?
(5.3) Is the numerical result being assessed likely to have been selected, on the basis of the results, from multiple eligible analyses of the data?

Certainty assessment:

We followed the detailed guidance given by the Grading of Recommendations, Assessment, Development and Evaluations (GRADE) Handbook to assess the certainty in the body of evidence for each outcome. The certainty of the evidence, as defined by the GRADE Working Group, reflects the extent to which we are confident that an effect estimate is correct and refers to an assessment of how good an indication the research provides of the likely effect. Regarding to randomized controlled trials, we initiated our assessment with the assumption of high certainty, which we then adjusted based on five GRADE criteria: study limitations, inconsistency, indirectness, imprecision, and publication bias, each of which could potentially downgrade the evidence's certainty. The intended interpretation of each level of certainty is provided in the subsequent sections.

Factors/reasons for downgrading the certainty of the evidence included risk of bias, inconsistency, indirectness, imprecision, and publication bias.

The four grades of certainty of the evidence were:

(1) High: further research is unlikely to change the confidence in the effect estimate.
(2) Moderate: further research is likely to have an important impact on the confidence in the effect estimate and may change the estimate.
(3) Low: further research is very likely to have an important impact on the confidence in the effect estimate and is likely to change the estimate.
(4) Very low: any effect estimate is very uncertain.

Appendix 3. Distribution of included studies and participants by country (32 countries, 176 studies, 11 696 participants)

A total of 176 studies comprising 11 696 participants from 32 countries were included in this systematic review and meta-analysis. The number of included studies and participants per country were as follows: China (39 studies, 2 723 participants), United States (34, 2 420), Brazil (14, 657), South Korea (13, 468), Iran (12, 657), Tunisia (10, 371), Spain (6, 359), Australia (5, 206), Canada (4, 1 015), United Kingdom (4, 193), Belgium (3, 124), New Zealand (3, 322), Switzerland (3, 609), Denmark (2, 141), Estonia (2, 56), France (2, 72), Israel (2, 220), Norway (2, 84), Serbia (2, 79), Turkey (2, 79), Chile (1, 274), Egypt (1, 30), Germany (1, 67), India (1, 69), Italy (1, 70), Mexico (1, 105), Poland (1, 22), Portugal (1,40), Saudi Arabia (1, 27), Singapore (1,24), Sweden (1, 76), Thailand (1, 37).

Appendix 4. Supplementary tables and figures

Table S1. The detailed basic characteristics of the included studies(N=176)

Author	Year	Country	Weight status	Age		Sample		Intervention measure		Intervention duration		Outcome indicator
				experimental group	control group	experimental group (boys/girls)	control group (boys/girls)	experimental group	control group			
Abassi et al.	2020	Tunisia	Overweight and obesity	15.11 to 17.57	16.9±1.64	16(0/16)	8(0/8)	Mode of motion: running<br>Exercise time:55 min<br>Exercise frequency: three times per week<br>Exercise intensity:70-80% or 100-110% of MAS	Mode of motion: daily activities	12 weeks	12 weeks	1,2,3,8,14,15
Abassi et al.	2022	Tunisia	Overweight and obesity	16.7±0.20	16.7±0.20	26(0/26)	12(0/12)	Mode of motion: running<br>Exercise time:40 min<br>Exercise frequency: three times per week<br>Exercise intensity:100-110% or 70-80% of MAS	Mode of motion: daily activities	12 weeks	12 weeks	1,2,3,6,7,8
Abassi et al.	2023	Tunisia	Overweight and Obesity	16.4±1.2	16.4±1.2	26(0/26)	12(0/12)	Mode of motion: HIIT and MIIT<br>Exercise time:42 min<br>Exercise frequency: three times per week<br>Exercise intensity:100-110% or 70-80% MAS	Mode of motion: daily activities	12 weeks	12 weeks	1,2,3
Aguilar-Cordero et al.	2020	Spain	Overweight and obesity	10.43±1.35	10.43±1.35	49	49	Mode of motion: aerobic exercise<br>Exercise time:90 min<br>Exercise frequency: four times per week<br>Exercise intensity: medium and low intensity	Mode of motion: daily activities	36 weeks	36 weeks	3,6,7
Ahmadi et al.	2020	Iran	Overweight and obesity	11.09-17.10	12.70±2.64	86(31/55)	27(14/13)	Mode of motion: HIIT and resistance training<br>Exercise time:50 min<br>Exercise frequency: three times per week<br>Exercise intensity: medium or high intensity	Mode of motion: daily activities	8 weeks	8 weeks	1,2,10,11,12,13
Alberga et al.	2016	Canada	Obesity	14.1 to 17.4	15.6±1.3	228	76	Mode of motion: cycling, running and strength training<br>Exercise time:20-45/ 30-40 min<br>Exercise frequency: four times per week<br>Exercise intensity:70-85% HRmax	Mode of motion: daily activities	22 weeks	22 weeks	5
Alves et al.	2019	Portugal	Overweight and obesity	14.77±1.49	14.77±1.49	30	10	Mode of motion: aerobic and strength training<br>Exercise time:60 min<br>Exercise frequency: two or three times per week<br>Exercise intensity:75%VO_2max	Mode of motion: daily activities	10 weeks	10 weeks	1,3,4

Author	Year	Country	Weight status	Age		Sample		Intervention measure		Intervention duration		Outcome indicator
				experimental group	control group	experimental group (boys/girls)	control group (boys/girls)	experimental group	control group			
Azad et al.	2011	Iran	Overweight and obesity	16.53±0.83	16.6±0.81	15(15/0)	15(15/0)	Mode of motion: running<br>Exercise time:30 min<br>Exercise frequency: three times per week<br>Exercise intensity:75-85%VO_2max	Mode of motion: daily activities	24 weeks	24 weeks	1
Barbeau et al.	2007	American	Overweight	8-12	8-12	118(0/118)	83(0/83)	Mode of motion: physical activity<br>Exercise time:80 min<br>Exercise frequency: five times per week<br>Exercise intensity: above 150 bpm HR	Mode of motion: daily activities	40 weeks	40 weeks	1,2,3
Ben et al.	2010	Tunisia	Obesity	13.1±0.8	13.3±0.6	14(7/7)	14(7/7)	Mode of motion: aerobic exercise<br>Exercise time:90 min<br>Exercise frequency: four times per week<br>Exercise intensity: medium and low intensity	Mode of motion: daily activities	8 weeks	8 weeks	1,3
Benson et al.	2008	New Zealand	Overweight and Obesity	12.3±1.3	12.2±1.3	32	38	Mode of motion: resistance training<br>Exercise time:60 min<br>Exercise frequency: two times per week<br>Exercise intensity: high intensity	mode of motion: daily activities	8 weeks	8 weeks	1,2,5,10,12,13,14,15
Bezerra et al.	2023	Brazil	Overweight	7.9±1.0	7.9±1.0	20	21	Mode of motion: physical activity<br>Exercise time:60 min<br>Exercise frequency: three times per week<br>Exercise intensity: medium and high intensity	Mode of motion: daily activities	10 weeks	10 weeks	1,2,6,7,10,11,12,13,14
Bharath et al.	2018	America	Obesity	14.6±1.0	14.8±1.0	20(0/20)	20(0/20)	Mode of motion: resistance and aerobic exercise<br>Exercise time:60 min<br>Exercise frequency: five times per week<br>Exercise intensity:40-70%HRmax	Mode of motion: daily activities	12 weeks	12 weeks	1,2,3,4,6,7,9,14,15
Boer et al.	2014	Belgium	Overweight	13.1-21.2	17.4±2.4	32(21/11)	14(9/5)	Mode of motion: cycling<br>Exercise time:40 min<br>Exercise frequency: two times per week<br>Exercise intensity: medium and high intensity	Mode of motion: daily activities	15 weeks	15 weeks	1,2,3,6,7,10,11,12,13, 14
Boff et al.	2020	Brazil	Overweight and Obesity	16.42±1.17	16.46±1.01	34	31	Mode of motion: physical exercise<br>Exercise time:30 min<br>Exercise frequency: one time per week<br>Exercise intensity: medium and low intensity	Mode of motion: daily activities	12 weeks	12 weeks	1,2,6,7,11,12,13,20

Author	Year	Country	Weight status	Age		Sample		Intervention measure		Intervention duration		Outcome indicator
				experimental group	control group	experimental group (boys/girls)	control group (boys/girls)	experimental group	control group			
Bowen et al.	2010	China	Overweight and Obesity	12-16	12-16	22	18	Mode of motion: aerobic exercise<br>Exercise time:60 min<br>Exercise frequency: above three times per week<br>Exercise intensity:50-60%VO_2max	Mode of motion: daily activities	10 weeks	10 weeks	1,3,10,11,12,13,14,15
Brennan et al.	2012	Australia	Overweight and Obese	14.3±1.9	14.3±1.9	42	21	Mode of motion: aerobic exercise<br>Exercise time:60 min<br>Exercise frequency: once a week<br>Exercise intensity: medium and low intensity	Mode of motion: daily activities	24 weeks	24 weeks	16,17,19
Bruyndonckx et al.	2015	Belgium	Obesity	15.4±1.5	15.1±1.2	27	21	Mode of motion: aerobic exercise and resistance training<br>Exercise time:40 min<br>Exercise frequency: three times per week<br>Exercise intensity: medium and low intensity	Mode of motion: daily activities	40 weeks	40 weeks	1,3,6,7,10,12,13
Cao et al.	2022	China	Obesity	11.2±0.7	10.9±0.4	20(10/10)	20(10/10)	Mode of motion: running<br>Exercise time:38 min<br>Exercise frequency: three times per week<br>Exercise intensity:100% of MAS	Mode of motion: daily activities	12 weeks	12 weeks	1,3,4,8,9
Carrel et al.	2005	American	Obesity	12.5±0.5	12.5±0.7	27(13/14)	23(13/10)	Mode of motion: aerobic exercise<br>Exercise time:45 min<br>Exercise frequency: five times every two weeks<br>Exercise intensity: medium and low intensity	Mode of motion: daily activities	36 weeks	36 weeks	1,3,4,14,15
Chae et al.	2010	Korea	Obesity	10.4±3.1	10.6±3.8	19(11/8)	19(10/9)	Mode of motion: aerobic exercise and resistance training<br>Exercise time:90 min<br>Exercise frequency: two times per week<br>Exercise intensity: medium and high intensity	Mode of motion: daily activities	12 weeks	12 weeks	1,3,4,10,11,12,13
Chuensiri et al.	2018	Thailand	Obesity	10.7-11.3	10.6±0.3	26(26/0)	11(11/0)	Mode of motion: cycling<br>Exercise time:23 min<br>Exercise frequency: three times per week<br>Exercise intensity:90% or 170% of peak power output	Mode of motion: daily activities	12 weeks	12 weeks	1,2,3,5,6,7,10,11,12, 13
CROKER et al.	2012	UK	Overweight and Obesity	10.8±1.6	9.8±1.4	33	30	Mode of motion: physical activity<br>Exercise time:90 min<br>Exercise frequency: everyday<br>Exercise intensity: medium and low intensity	Mode of motion: daily activities	24 weeks	24 weeks	1,2,16,17

Author	Year	Country	Weight status	Age		Sample		Intervention measure		Intervention duration		Outcome indicator
				experimental group	control group	experimental group (boys/girls)	control group (boys/girls)	experimental group	control group			
Crova et al.	2014	Italy	Overweight	9.6±0.5	9.6±0.5	37(20/17)	33(15/18)	Mode of motion: tennis-specific training<br>Exercise time:120 min<br>Exercise frequency: one time per week<br>Exercise intensity: average heart rate 150.5±6.4	Mode of motion: daily activities	21 weeks	21 weeks	1,4,8
Cvetković et al.	2018	Serbia	Overweight and obesity	11-13	11-13	21(21/0)	14(14/0)	Mode of motion: football training and running<br>Exercise time:60 or 38 min<br>Exercise frequency: three times per week<br>Exercise intensity:100% of MAS	Mode of motion: daily activities	12 weeks	12 weeks	1,3,6,7,8,9
Daley et al.	2006	UK	Obesity	11-16	11-16	49	26	Mode of motion: aerobic exercise and light body-conditioning/stretching exercises<br>Exercise time:30 min<br>Exercise frequency: three times per week<br>Exercise intensity: < 59% of HR reserve	Mode of motion: daily activities	8 weeks	8 weeks	9,16,18
Danielsen et al.	2013	Norway	Obesity	10.68±1.24	10.68±1.24	22	20	Mode of motion: body activity<br>Exercise time:30 min<br>Exercise frequency: everyday<br>Exercise intensity: medium and low intensity	Mode of motion: daily activities	12 weeks	12 weeks	16,18
Davis et al.(1)	2009	America	Overweight	15.5±1.0	15.5±1.0	17(8/9)	21(11/10)	Mode of motion: strength training<br>Exercise time:60 min<br>Exercise frequency: two times per week<br>Exercise intensity: medium and high intensity	Mode of motion: daily activities	16 weeks	16 weeks	1,14,15
DAVIS et al.(2)	2009	America	Overweight	13.8-16.9	15.3±1.1	24(0/24)	10(0/10)	Mode of motion: cardiovascular activity and strength training<br>Exercise time:60 min<br>Exercise frequency: two times per week<br>Exercise intensity: medium and high intensity	Mode of motion: daily activities	16 weeks	16 weeks	1,14,15
Davis et al.	2020	America	Overweight and obesity	9.6±0.73	9.7±0.94	90(23/67)	85(30/55)	Mode of motion: aerobic exercise<br>Exercise time:40 min<br>Exercise frequency: five times per week<br>Exercise intensity:>140 beats per minute	Mode of motion: sedentary activities	32 weeks	32 weeks	1,2,3,5,6,7,10,11,12,13,14,15
DeBar et al.	2012	America	Overweight	14.12±1.48	14.03±1.50	100(0/100)	95(0/95)	Mode of motion: Yoga, Dance Video Games and aerobic training<br>Exercise time:30-60 min<br>Exercise frequency: five times per week<br>Exercise intensity: medium and low intensity	Mode of motion: daily activities	24 weeks	24 weeks	10,11,12,13,14,17

Author	Year	Country	Weight status	Age		Sample		Intervention measure		Intervention duration		Outcome indicator
				experimental group	control group	experimental group (boys/girls)	control group (boys/girls)	experimental group	control group			
Dennis et al.	2013	America	Overweight and obesity	9.3±1.1	9.3±1.1	70	42	Mode of motion: aerobic exercise<br>Exercise time:20 or 40 min<br>Exercise frequency: five times per week<br>Exercise intensity:167±7 beats per minute	Mode of motion: daily activities	13 weeks	13 weeks	1,2,3,5
Dias et al.	2018	Australia	Obesity	9.5-14.3	11.8±2.4	41	21	Mode of motion: running<br>Exercise time:25 or 44 min<br>Exercise frequency: three times per week<br>Exercise intensity:60%-95%HRmax	Mode of motion: daily activities	12 weeks	12 weeks	1,3,5,10,11,12,13,14,20
Duft et al.	2020	Brazil	Overweight and obesity	14.44±1.04	14.72±1.07	18(9/9)	19(9/10)	Mode of motion: resistance training and aerobic training<br>Exercise time:60 min<br>Exercise frequency: three times per week<br>Exercise intensity:50-85%VO_2peak	Mode of motion: daily activities	12 weeks	12 weeks	1,2,3,5,10,14
Elmahgoub et al.	2009	Belgium	Overweight and obesity	14-22	14-22	15	15	Mode of motion: strength and endurance exercises<br>Exercise time:50 min<br>Exercise frequency: three times per week<br>Exercise intensity:60-75%HRmax	Mode of motion: daily activities	10 weeks	10 weeks	1,2,5,10,11,12,13
Elnaggar et al.	2021	Saudi Arabia	Obesity	12.69±2.53	13.36±2.17	13	14	Mode of motion: aerobic exercise<br>Exercise time:25-60 min<br>Exercise frequency: three times per week<br>Exercise intensity: 40-80% of HRmax	Mode of motion: daily activities	8 weeks	8weeks	5,8
Eskandari et al.	2020	Iran	Overweight and obesity	15.4±1.1	15.4±1.1	24(24/0)	12(12/0)	Mode of motion: jump rope exercise<br>Exercise time:40 min<br>Exercise frequency: five times per week<br>Exercise intensity:60-90 jumps/min	Mode of motion: daily activities	6 weeks	6 weeks	1
Espinoza-Silva et al.	2019	Chile	Overweight and obesity	8.12±1.50	8.12±1.50	210	64	Mode of motion: HIIT<br>Exercise time:40-50 min<br>Exercise frequency: two times per week<br>Exercise intensity: high intensity	Mode of motion: daily activities	28 weeks	28 weeks	1,2,3,6,7
Faria et al.	2020	Brazil	Overweight	14.6-17.1	16.5±1.0	50(21/29)	26(11/15)	Mode of motion: running and resistance training<br>Exercise time:53 or 47 min<br>Exercise frequency: two times per week<br>Exercise intensity:55%-65% or 90%VO_2max	Mode of motion: daily activities	12 weeks	12 weeks	2,3,5,6,7,10,11,12,13,14,15,20

Author	Year	Country	Weight status	Age		Sample		Intervention measure		Intervention duration		Outcome indicator
				experimental group	control group	experimental group (boys/girls)	control group (boys/girls)	experimental group	control group			
Farpour-Lambert et al.	2009	Switzerland	Obesity	9.1±1.4	8.8±1.6	22(9/13)	22(7/15)	Mode of motion: aerobic exercises and strengthening exercises<br>Exercise time:60 min<br>Exercise frequency: three times per week<br>Exercise intensity:55-65%VO_2max	Mode of motion: daily activities	12 weeks	12 weeks	1,3,4,6,7,10,11,12,13,14,15
Farpour-Lambert et al.	2019	Switzerland	Obesity	8.3-10.8	9.7±1.0	47	19	Mode of motion: aerobic exercises and resistance training<br>Exercise time:60 min<br>Exercise frequency: two times per week<br>Exercise intensity: medium or high intensity	Mode of motion: daily activities	24 weeks	24 weeks	1,2,3
Ghorbanian et al.	2013	Iran	Overweight and obesity	17.35±1.07	16.90±1.15	15(15/0)	15(15/0)	Mode of motion: endurance rope training<br>Exercise time:40 min<br>Exercise frequency: four times per week<br>Exercise intensity: medium and low intensity	Mode of motion: daily activities	8 weeks	8 weeks	1,3,4,10,11,12,13,14
Goldfield et al.	2015	Canada	Obesity	14-17	15.6±1.3	228(67/161)	76(24/52)	Mode of motion: aerobic exercise and resistance training<br>Exercise time:20-45 min<br>Exercise frequency: four times per week<br>Exercise intensity:65-85%HRmax	Mode of motion: daily activities	22 weeks	22 weeks	16,17,18
Gonzalez-Galvez et al.	2024	Spain	Overweight and Obesity	12.51±0.75	12.51±0.75	20	12	Mode of motion: sprint interval training<br>Exercise time:12 min<br>Exercise frequency: twice per week<br>Exercise intensity:80%-85%/90%-95%HRmax	Mode of motion: daily activities	8 weeks	8 weeks	1,3,4,6,7
Guijun	2009	China	Obesity	11.76-12.67	12.56±0.25	16	10	Mode of motion: aerobic exercise<br>Exercise time:70-90 min<br>Exercise frequency: three-four times per week<br>Exercise intensity:50-70%HRmax	Mode of motion: daily activities	24 weeks	24 weeks	1,6,7,10,11,12,13
Gulijianati et al.	2022	China	Overweight	10.76±1.35	10.35±1.69	47(29/18)	46(27/19)	Mode of motion: aerobic exercise<br>Exercise time:40 min<br>Exercise frequency: four times per week<br>Exercise intensity:140-160 beats per minute	Mode of motion: daily activities	12 weeks	12 weeks	1,3,10,11,12,13,14,15
Guo et al.	2015	China	Overweight and obesity	8-10	8-10	23	12	Mode of motion: aerobic exercise<br>Exercise time:60 min<br>Exercise frequency: unclear<br>Exercise intensity:70-80%HRmax	Mode of motion: daily activities	48 weeks	48 weeks	1,2,10,11,12,13,14

Author	Year	Country	Weight status	Age		Sample		Intervention measure		Intervention duration		Outcome indicator
				experimental group	control group	experimental group (boys/girls)	control group (boys/girls)	experimental group	control group			
Haiying et al.	2023	China	Obesity	15±1.08	15±1.12	20	20	Mode of motion: aerobic exercise<br>Exercise time:110 min<br>Exercise frequency: everyday<br>Exercise intensity: 60% to 80% VO_2max	Mode of motion: daily activities	4 weeks	4 weeks	3
Hamila et al.	2018	Tunisia	Overweight and obesity	13.3-15.5	14.5±0.9	23(9/14)	8(3/5)	Mode of motion: walking<br>Exercise time:60 min<br>Exercise frequency: three times per week<br>Exercise intensity:95-159 beats per minute	Mode of motion: daily activities	8 weeks	8 weeks	1,2,3,4,6,7,9
Harder-Lauridsen et al.	2014	Denmark	Overweight	8.8±0.9	8.7±0.9	18	17	Mode of motion: aerobic and resistance exercise<br>Exercise time:105 min<br>Exercise frequency: one time per week<br>Exercise intensity: medium and low intensity	Mode of motion: daily activities	20 weeks	20 weeks	1,2,3,6,7,9,10,11,12, 13,14,20
Hay et al.	2016	Canada	Overweight and obesity	13.3-17.0	15.2±1.7	70(12/58)	33	Mode of motion: running<br>Exercise time:30-45 min<br>Exercise frequency: three times per week<br>Exercise intensity:40-55% or 70-85%HRmax	Mode of motion: daily activities	24 weeks	24 weeks	2,3,5
Heidarianpour et al.	2023	Iran	Overweight and obesity	8.51±0.44	8.35±0.59	15(0/15)	15(0/15)	Mode of motion: aerobic and resistance training<br>Exercise time:60 min<br>Exercise frequency: three times per week<br>Exercise intensity:55-65% of individual maximal cardiorespiratory fitness	Mode of motion: daily activities	12 weeks	12 weeks	2,5,6,7,9,16,19
Hong et al.	2016	China	Obesity	8.3±5.4	8.4±5.9	55	55	Mode of motion: running, skipping rope and ball sports<br>Exercise time:60 min<br>Exercise frequency: everyday<br>Exercise intensity: medium and low intensity	Mode of motion: daily activities	unclear	unclear	1,10,11
Hongling et al.	2012	China	Obesity	13-14	13-14	30(30/0)	30(30/0)	Mode of motion: aerobic and resistance exercise<br>Exercise time:60-70 min<br>Exercise frequency: two times per week<br>Exercise intensity: medium and low intensity	Mode of motion: daily activities	8 weeks	8 weeks	1,3,4,6,7,9,10,11,12, 13,14
Horner et al.	2015	America	Obesity	12.7-16.5	14.9±1.8	50	16	Mode of motion: aerobic or resistance exercise<br>Exercise time:60 min<br>Exercise frequency: three times per week<br>Exercise intensity: 60-75%VO_2max or 8-12RM	Mode of motion: daily activities	12 weeks	12 weeks	6,7,10,11,12,13

Author	Year	Country	Weight status	Age		Sample		Intervention measure		Intervention duration		Outcome indicator
				experimental group	control group	experimental group (boys/girls)	control group (boys/girls)	experimental group	control group			
Jain et al.	2022	Indian	Overweight and obesity	11.7±1.7	11.4±1.8	41	28	Mode of motion: yoga<br>Exercise time:60 min<br>Exercise frequency: five times per week<br>Exercise intensity: medium and low intensity	Mode of motion: daily activities	18 weeks	18 weeks	1,2,6,7,9
Jelalian et al.	2011	America	Obesity	13-16	13-16	44	45	Mode of motion: aerobic exercise<br>Exercise time:60 min<br>Exercise frequency: one time per week<br>Exercise intensity: medium and low intensity	Mode of motion: daily activities	16 weeks	16 weeks	1
Jelalian et al.	2019	American	Overweight and obesity	15.25±1.51	14.44±1.67	24(7/17)	9(2/7)	Mode of motion: aerobic exercise<br>Exercise time:60 min<br>Exercise frequency: once a week<br>Exercise intensity: medium and low intensity	Mode of motion: Cognitive Behavioral Therapy	24 weeks	24 weeks	1,16
Jiafen et al.	2017	China	Obesity	6.0-13.0	6.0-12.0	78(44/34)	78(46/32)	Mode of motion: aerobic exercise<br>Exercise time:60-90 min<br>Exercise frequency: six-twelve times per week<br>Exercise intensity:60-70%HRmax	Mode of motion: daily activities	10 weeks	10 weeks	14
Jian et al.	2014	China	Obesity	19.8±2.5	19.8±2.5	44(0/44)	44(0/44)	Mode of motion: belly dance<br>Exercise time:60 min<br>Exercise frequency: five times per week<br>Exercise intensity:60-80%HRmax	Mode of motion: daily activities	16 weeks	16 weeks	1,2,6,7,9,10,11,12,13
Jianjun et al.	2012	China	Obesity	18.24±1.57	18.28±1.02	20(20/0)	15(15/0)	Mode of motion: swimming and brisk walking and running<br>Exercise time:120-130 min<br>Exercise frequency: six times per week<br>Exercise intensity:3-7MET	Mode of motion: daily activities	12 weeks	12 weeks	1,2,3,10,11,12,13,14,15
Julian et al.	2022	France	Obesity	11.9-14.1	13.2±1.0	38	11	Mode of motion: cycling training<br>Exercise time:15 or 45 min<br>Exercise frequency: two times per week<br>Exercise intensity:60% or 75-90%VO_2max	Mode of motion: daily activities	16 weeks	16 weeks	1,3
Jun et al.	2006	China	Obesity	8-12	8-12	68	63	Mode of motion: aerobic exercise<br>Exercise time:60 min<br>Exercise frequency: five time per week<br>Exercise intensity: 65%HRmax	Mode of motion: daily activities	12 weeks	12 weeks	3,10,11,12,13,14

Author	Year	Country	Weight status	Age		Sample		Intervention measure		Intervention duration		Outcome indicator
				experimental group	control group	experimental group (boys/girls)	control group (boys/girls)	experimental group	control group			
Kai et al.	2024	China	Obesity	14.8±0.8	15.0±0.8	20(0/20)	24(0/24)	Mode of motion: jump rope and taekwondo<br>Exercise time:50 min<br>Exercise frequency: five times per week<br>Exercise intensity:40%~70%HRR	Mode of motion: daily activities	12 weeks	12 weeks	1,2,3,4,6,7
Karacabey	2009	Turkey	Obesity	11.8±0.5	11.2±0.8	20(20/0)	20(20/0)	Mode of motion: walking and jogging<br>Exercise time:30-65 min<br>Exercise frequency: three times per week<br>Exercise intensity:60-65%HRR	Mode of motion: daily activities	12 weeks	12 weeks	1,12,13
Ke et al.	2024	China	Obesity	16.7±0.7	16.6±0.8	10	10	Mode of motion: HIIT<br>Exercise time:60 min<br>Exercise frequency: three times per week<br>Exercise intensity: 65%-80%HRmax	Mode of motion: daily activities	12 weeks	12 weeks	1,2,3,10,11,12,13,14
Kelishadi et al.	2008	Iran	Obesity	7.7±1.2	7.7±1.2	45	47	Mode of motion: physical training<br>Exercise time:40 min<br>Exercise frequency: five times per week<br>Exercise intensity: medium and low intensity	Mode of motion: daily activities	24 weeks	24 weeks	1,2,6,7,10,11,12,13,14
Kelly et al.	2004	America	Overweight	11.0±0.63	11.0±0.71	10	10	Mode of motion: aerobic exercise<br>Exercise time:40 min<br>Exercise frequency: four times per week<br>Exercise intensity:50-80% of VO_2peak	Mode of motion: daily activities	8 weeks	8 weeks	1,3,5,6,7,10,11,12,13,14,15
Kelly et al.	2019	America	Obesity	15.29±0.95	15.58±0.99	13(13/0)	13(13/0)	Mode of motion: resistance exercise<br>Exercise time: ≤60 min<br>Exercise frequency: two times per week<br>Exercise intensity:62-97%1RM	Mode of motion: daily activities	16 weeks	16 weeks	14
Khan et al.	2014	America	Overweight and obesity	8.8±0.5	8.8±0.6	46	42	Mode of motion: aerobic exercise<br>Exercise time:70 min<br>Exercise frequency: five times per week<br>Exercise intensity: medium and high intensity	Mode of motion: daily activities	36weeks	36weeks	1,3,4
Kim et al.	2007	Korea	Obesity	17±0.11	17±0.11	14(14/0)	12(12/0)	Mode of motion: jump rope exercise training<br>Exercise time:40 min<br>Exercise frequency: five times per week<br>Exercise intensity: medium and low intensity	Mode of motion: daily activities	6 weeks	6 weeks	1,2,3,6,7,9,10,11,12,13,14,15

Author	Year	Country	Weight status	Age		Sample		Intervention measure		Intervention duration		Outcome indicator
				experimental group	control group	experimental group (boys/girls)	control group (boys/girls)	experimental group	control group			
Kim et al.	2011	Korea	Obesity	12.7±0.2	12.6±0.2	12(12/0)	10(10/0)	Mode of motion: aerobic exercise<br>Exercise time:50 min<br>Exercise frequency: three times per week<br>Exercise intensity:60-70%HRmax	Mode of motion: daily activities	12 weeks	12 weeks	1,2,3,6,7
Kim et al.	2020	America	Obesity	15±1	15±1	24(0/24)	24(0/24)	Mode of motion: jump rope exercise<br>Exercise time:50 min<br>Exercise frequency: five times per week<br>Exercise intensity:40-70%HRR	Mode of motion: daily activities	12 weeks	12 weeks	1,2,3,6,7,14,15
Kim et al.	2022	Korea	Overweight and obesity	16-18	16-18	13(0/13)	13(0/13)	Mode of motion: aerobic and resistance exercise<br>Exercise time:50 min<br>Exercise frequency: three times per week<br>Exercise intensity:55-75%HRmax	Mode of motion: daily activities	12 weeks	12 weeks	1,2,3,10,11,12,13,14
Lambrick et al.	2016	UK	Obesity	9.3±0.8	9.4±0.8	15(10/5)	14(7/7)	Mode of motion: active games<br>Exercise time:40 min<br>Exercise frequency: two times per week<br>Exercise intensity: high intensity	Mode of motion: daily activities	6 weeks	6 weeks	1,2,3,4,5,8
Larsen et al.	2016	Denmark	Overweight and obesity	12±0.4	12±0.4	55	51	Mode of motion: Fun Sports<br>Exercise time:180 min<br>Exercise frequency: seven times per week<br>Exercise intensity: medium and low intensity	Mode of motion: daily activities	6 weeks	6 weeks	1,2,3,6,11
Lee et al.	2010	Korea	Obesity	12-14	12-14	36	18	Mode of motion: aerobic and resistance training exercise<br>Exercise time:60 min<br>Exercise frequency: three times per week<br>Exercise intensity:60-80%VO_2max	Mode of motion: daily activities	10 weeks	10 weeks	1,2,4,6,10,11,12,13,14
Lee et al.	2012	America	Obesity	13.1-17.1	14.8±1.4	31(31/0)	11(11/0)	Mode of motion: aerobic and resistance exercise<br>Exercise time:60 min<br>Exercise frequency: three times per week<br>Exercise intensity:50-75%VO_2peak or 60% of baseline repetition maximum	Mode of motion: daily activities	12 weeks	12 weeks	1,2,5,14,15
Lee et al.	2013	America	Obesity	12.7-16.7	15.0±2.2	28(0/28)	8(0/8)	Mode of motion: running and body exercises<br>Exercise time:60 min<br>Exercise frequency: three times per week<br>Exercise intensity:60-75%VO_2peak	Mode of motion: daily activities	12 weeks	12 weeks	1,2,3,5,14,15

Author	Year	Country	Weight status	Age		Sample		Intervention measure		Intervention duration		Outcome indicator
				experimental group	control group	experimental group (boys/girls)	control group (boys/girls)	experimental group	control group			
Lee et al.	2016	Korea	Obesity	9-12	9-12	15(4/11)	15(12/3)	Mode of motion: fun health sports<br>Exercise time: unclear<br>Exercise frequency: unclear<br>Exercise intensity: medium and low intensity	Mode of motion: daily activities	10 weeks	10 weeks	1,17
Liangmei et al.	2015	China	Obesity	13-14	13-14	12(6/6)	12(6/6)	Mode of motion: aerobic exercise<br>Exercise time:50 min<br>Exercise frequency: three times per week<br>Exercise intensity:60%-70%VO_2peak	Mode of motion: daily activities	24 weeks	24 weeks	1,2,10,11,12,13
Lidan et al.	2012	China	Obesity	10±2.4	10±2.4	30	30	Mode of motion: aerobic exercise<br>Exercise time: 60 min<br>Exercise frequency: five times per week<br>Exercise intensity: 140-160 beats/min of HR	Mode of motion: daily activities	12 weeks	12 weeks	10,11,14
Lima et al.	2020	Switzerland	Overweight and obesity	9.01±0.45	9.01±0.45	293	206	Mode of motion: aerobic and strength exercises<br>Exercise time:45 min<br>Exercise frequency: two times per week<br>Exercise intensity: medium and low intensity	Mode of motion: daily activities	36 weeks	36 weeks	11,14
Lingling	2021	China	Overweight and obesity	16.1±1.2	15.9±1.2	20(20/0)	20(20/0)	Mode of motion: running<br>Exercise time:33-78 min<br>Exercise frequency: three times per week<br>Exercise intensity:100-110%MAP	Mode of motion: daily activities	12 weeks	12 weeks	1,2,3,4,8,10,11,12,13
Liqun et al.	2014	China	Overweight and obesity	7-11	7-11	76(40/36)	97(53/44)	Mode of motion: unclear<br>Exercise time:10 min<br>Exercise frequency: ten times per week<br>Exercise intensity: medium and low intensity	Mode of motion: daily activities	48 weeks	48 weeks	1,2,6,7,10,11,12,13
Lira et al.	2017	Brazil	Obesity	13.6-16.30	14.72±1.35	51	33	Mode of motion: running<br>Exercise time:34-58 min<br>Exercise frequency: three times per week<br>Exercise intensity: medium or high intensity	Mode of motion: daily activities	12 weeks	12 weeks	1,10,11,12,13,14,15
Liu et al.	2018	China	Obesity	14.6±0.7	14.7±0.8	30(0/30)	20(0/20)	Mode of motion: aerobic exercises<br>Exercise time:120 min<br>Exercise frequency: twelve times per week<br>Exercise intensity:100-140 beats per minute	Mode of motion: daily activities	4 weeks	4 weeks	1,2,10,11,12,13,14,15

Author	Year	Country	Weight status	Age		Sample		Intervention measure		Intervention duration		Outcome indicator
				experimental group	control group	experimental group (boys/girls)	control group (boys/girls)	experimental group	control group			
Lofrano-Prado et al.	2022	Brazil	Obesity	15±1	15±1	37(17/20)	37(17/20)	Mode of motion: recreational physical activity<br>Exercise time:60 min<br>Exercise frequency: two times per week<br>Exercise intensity: medium and low intensity	Mode of motion: daily activities	12 weeks	12 weeks	2,3,16
Lopes et al.	2016	Brazil	Overweight	14.6±1.15	14.4±1.16	17(0/17)	16(0/16)	Mode of motion: resistance and aerobic training<br>Exercise time:60 min<br>Exercise frequency: three times per week<br>Exercise intensity:50-85%VO_2peak	Mode of motion: daily activities	12 weeks	12 weeks	1,3,5,10,11,12,13,14,15
Luo et al.	2013	China	Obesity	11.11-12.93	11.22-13.26	95(52/43)	72(38/34)	Mode of motion: aerobic exercise<br>Exercise time:180 min<br>Exercise frequency: twelve times per week<br>Exercise intensity:120-150 beats per minute	Mode of motion: daily activities	6 weeks	6 weeks	1,2,3,6,7,10,11,12,13,14,15
Machado et al.	2022	Brazil	Overweight and Obesity	10.3±1.8	10.9±2.1	20(9/11)	15(5/10)	Mode of motion: aerobic exercise<br>Exercise time:50 min<br>Exercise frequency: two times per week<br>Exercise intensity: medium and low intensity	Mode of motion: daily activities	24 weeks	24 weeks	6,7,11,12,13,14,15
Maddison et al.	2011	New Zealand	Overweight and obesity	11.6±1.1	11.6±1.1	115	116	Mode of motion: active video games<br>Exercise time: 80 min<br>Exercise frequency: three times per week<br>Exercise intensity: medium and high intensity	Mode of motion: daily activities	24 weeks	24 weeks	1,2,3,4
McCormack et al.	2014	America	Obesity	13.8±2.2	12.1±1.2	10(8/2)	8(5/3)	Mode of motion: aerobic and resistance training<br>Exercise time:80 min<br>Exercise frequency: three times per week<br>Exercise intensity:60-80%HRR	Mode of motion: daily activities	8 weeks	8 weeks	1,3,14,15
McNarry et al.	2015	UK	Obesity	9.3±0.9	9.3±0.9	13	13	Mode of motion: high-intensity games<br>Exercise time:60 min<br>Exercise frequency: two times per week<br>Exercise intensity: high intensity	Mode of motion: daily activities	6 weeks	6 weeks	1
Medrano et al.	2019	Spain	Overweight and obesity	10.9±1.1	11.0±1.1	26	17	Mode of motion: aerobic and strength exercises<br>Exercise time:90 min<br>Exercise frequency: three times per week<br>Exercise intensity:76%HRmax	Mode of motion: daily activities	22 weeks	22 weeks	1,3

Author	Year	Country	Weight status	Age		Sample		Intervention measure		Intervention duration		Outcome indicator
				experimental group	control group	experimental group （boys/girls）	control group （boys/girls）	experimental group	control group			
Meng et al.	2022	China	Obesity	10.5-12.2	11.0±0.7	23(23/0)	13(13/0)	Mode of motion: running<br>Exercise time:11-30mins<br>Exercise frequency: three times per week<br>Exercise intensity:60-70% or 90-100% of MAS	Mode of motion: daily activities	12 weeks	12 weeks	1,2,3,5,6,7,10,11,12,13,14,15
Meyer et al.	2006	Germany	Obesity	13.7±2.1	14.1±2.4	33(17/16)	34(17/17)	Mode of motion: aerobic training<br>Exercise time:60-90 min<br>Exercise frequency: three times per week<br>Exercise intensity: medium and low intensity	mode of motion: daily activities	24 weeks	24 weeks	1,3,6,11,12,13,15,20
Migueles et al.	2023	Spain	Overweight and obesity	10±1.1	10±1.1	47	45	Mode of motion: Aerobic and resistance exercise<br>Exercise time:90 min<br>Exercise frequency: three to five times per week<br>Exercise intensity:80% HRmax(38 min above 80%)	Mode of motion: daily activities	20 weeks	20 weeks	1,2,4,11,12,13,14,15,16,17,19
Mohammadi et al.	2019	Iran	Overweight	12.40±1.71	11.80±2.20	10(0/10)	10(0/10)	Mode of motion: aerobic and resistance training<br>Exercise time:75 min<br>Exercise frequency: three time per week<br>Exercise intensity:60-80%HHR	Mode of motion: daily activities	8 weeks	8 weeks	1,2,3,10,11,12,13,14
Monteiro et al.	2015	Brazil	Obesity	10.08-12.76	11.32±1.90	32	16	Mode of motion: aerobic and resistance training<br>Exercise time:50 min<br>Exercise frequency: three times per week<br>Exercise intensity:65%-85%VO_2peak	Mode of motion: daily activities	20 weeks	20 weeks	1,2,3,10,11,12,13
Moon et al.	2004	Korea	Obesity	10-12	10-12	34(22/12)	28(16/12)	Mode of motion: behavior modification<br>Exercise time:60-70 min<br>Exercise frequency: once a week<br>Exercise intensity: medium and low intensity	mode of motion: daily activities	8 weeks	8 weeks	3,16
Morgan et al.	2021	Egypt	Obesity	14.10±1.25	14.20±1.196	15	15	Mode of motion: aerobic exercise<br>Exercise time:50 min<br>Exercise frequency: three times per week<br>Exercise intensity: medium and low intensity	Mode of motion: routine medical treatment	12 weeks	12 weeks	1,10,11,12,13
Moslehi et al.	2019	Iran	Obesity	10.33-11.63	10.93±0.53	20(20/0)	10(10/0)	Mode of motion: running<br>Exercise time:25-40 min<br>Exercise frequency: three times per week<br>Exercise intensity:65-85%HRmax	Mode of motion: daily activities	8 weeks	8 weeks	1,3,4

Author	Year	Country	Weight status	Age		Sample		Intervention measure		Intervention duration		Outcome indicator
				experimental group	control group	experimental group (boys/girls)	control group (boys/girls)	experimental group	control group			
Murphy et al.	2009	America	Overweight	10.21±1.67	10.21±1.67	23	12	Mode of motion: an active vedio game(Dance Dance Revolution)<br>Exercise time:10-30 min<br>Exercise frequency: five times per week<br>Exercise intensity: medium and low intensity	Mode of motion: daily activities	12 weeks	12 weeks	1,5,6,7,10,11,12,13,14
Naczk et al.	2021	Poland	Overweight	14.9±2.35	14.4±1.97	11(7/4)	11(7/4)	Mode of motion: swimming<br>Exercise time:70-90 min<br>Exercise frequency: three times per week<br>Exercise intensity: medium and low intensity	Mode of motion: daily activities	33 weeks	33 weeks	1,3,4,8
Naylor et al.	2016	Australia	Obesity	17.3±0.8	15.3±0.8	8(2/6)	5(1/4)	Mode of motion: aerobic and resistance exercise<br>Exercise time:60 min<br>Exercise frequency: three times per week<br>Exercise intensity:65-85%HRmax	Mode of motion: daily activities	12 weeks	12 weeks	1,3,5,20
Nobre et al.	2017	Brazil	Overweight and obesity	9.8±0.9	9.9±1.1	40（40/0）	19（19/0）	Mode of motion: plyometric training<br>Exercise time:20 min<br>Exercise frequency: two times per week<br>Exercise intensity: medium and low and high intensity	Mode of motion: daily activities	12 weeks	12 weeks	1,3
Nowicka et al.	2009	Sweden	Obesity	11.8±0.9	11.6±0.9	38	38	Mode of motion: aerobic exercise<br>Exercise time: 30 min<br>Exercise frequency: one-two times per week<br>Exercise intensity: medium and low intensity	Mode of motion: daily activities	24 weeks	24 weeks	1,3
Park et al.	2007	Korea	Obesity	14.2±0.5	14.1±0.5	19(0/19)	21(0/21)	Mode of motion: walking<br>Exercise time:10-50 min<br>Exercise frequency: six times per week<br>Exercise intensity:55–75% HRmax	Mode of motion: daily activities	12 weeks	12 weeks	1,2,3,6,7,10,12,13,14,15,20
Pena et al.	2022	America	Obesity	14±1	14±2	79(46/33)	38(24/14)	Mode of motion: physical activity<br>Exercise time:60 min<br>Exercise frequency: twice times per week<br>Exercise intensity: above 150 bpm HR	Mode of motion: daily activities	24 weeks	24 weeks	1,2,3,9,14,15,20
Pena et al.	2023	America	Obesity	13.4±1.4	13.2±1.4	40(24/16)	24(15/9)	Mode of motion: physical activity<br>Exercise time:60 min<br>Exercise frequency: twice times per week<br>Exercise intensity: above 150 bpm	Mode of motion: daily activities	24 weeks	24 weeks	1,3

Author	Year	Country	Weight status	Age		Sample		Intervention measure		Intervention duration		Outcome indicator
				experimental group	control group	experimental group (boys/girls)	control group (boys/girls)	experimental group	control group			
Petty et al.	2009	America	Overweight	8.3-10.9	8.2-10.6	125	59	Mode of motion: low/high aerobic exercise<br>Exercise time:20-40 min<br>Exercise frequency: seven times per week<br>Exercise intensity: low/high intensity	mode of motion: daily activities	10-15 weeks	10-15 weeks	16,18
Plavsic et al.	2020	Serbia	Obesity	16.6±1.3	15.8±1.5	22(0/22)	22(0/22)	Mode of motion: aerobic exercise<br>Exercise time:43 min<br>Exercise frequency: two times per week<br>Exercise intensity:60-90%HRmax	Mode of motion: daily activities	12 weeks	12 weeks	1,2,3,5,6,7,8,9,10,11, 12,13,20
Prado et al.	2009	Brazil	Obesity	10.6±0.2	10.6±0.3	21	17	Mode of motion: aerobic and recreational exercises<br>Exercise time:60 min<br>Exercise frequency: three times per week<br>Exercise intensity: medium and low intensity	Mode of motion: daily activities	16 weeks	16 weeks	1,3,5
Prado et al.	2010	Brazil	Obesity	10.6±0.2	10.4±0.3	18	15	Mode of motion: aerobic exercise<br>Exercise time:60 min<br>Exercise frequency: three times per week<br>Exercise intensity: medium and low intensity	Mode of motion: daily activities	16 weeks	16 weeks	1,3,5
Qiong et al.	2015	China	Obesity	10.4-18.0	14.4±3.2	45(45/0)	15(15/0)	Mode of motion: aerobic exercise and resistance training<br>Exercise time:55-60 min<br>Exercise frequency: three times per week<br>Exercise intensity:60%VO_2max	Mode of motion: daily activities	8 weeks	8 weeks	1,3,4
Racil et al.(1)	2016	Tunisia	Obesity	14.2±1.2	14.2±1.2	33(0/33)	14(0/14)	Mode of motion: HIIT/MIIT<br>Exercise time:36-40 min<br>Exercise frequency: three times per week<br>Exercise intensity:100%/50% or 80%/50% MAS	Mode of motion: daily activities	12 weeks	12 weeks	2,3,6,7,8,9,14,15
Racil et al.(2)	2016	Tunisia	Obesity	15.3-17.7	16.9±1.0	49(0/49)	19(0/19)	Mode of motion: plyometric exercise + HIIT<br>Exercise time:30-34 min<br>Exercise frequency: three times per week<br>Exercise intensity:50-100%VO_2peak	Mode of motion: daily activities	12 weeks	12 weeks	2,3,5,14,15
Racil et al.	2013	Tunisia	Obesity	14.9-16.82	15.9±1.2	22(0/22)	12(0/12)	Mode of motion: HIIT/MIIT<br>Exercise time:40 min<br>Exercise frequency: three times per week<br>Exercise intensity:70-80% or 100-110%MAS	Mode of motion: daily activities	12 weeks	12 weeks	2,3,5,10,11,12,13,14, 15

Author	Year	Country	Weight status	Age		Sample		Intervention measure		Intervention duration		Outcome indicator
				experimental group	control group	experimental group (boys/girls)	control group (boys/girls)	experimental group	control group			
Racil et al.	2024	Tunisia	Obesity	13.22-15.46	14.50±1.22	24(0/24)	11(0/11)	Mode of motion: HIIT/MIIT<br>Exercise time:28 min<br>Exercise frequency: three times per week<br>Exercise intensity: 50%-1000%MAS	Mode of motion: daily activities	8 weeks	8 weeks	2,3,4,6,7,8,14
Ramezani et al.	2017	Iran	Obesity	10.05±1.41	10.05±1.41	45(45/0)	15(15/0)	Mode of motion: endurance and resistance exercise<br>Exercise time:50-65 min<br>Exercise frequency: four times per week<br>Exercise intensity: 50%-75%HRmax	Mode of motion: daily activities	8 weeks	8 weeks	10,11,12,13,14
Regaieg et al.	2013	Tunisia	Obesity	10.92±0.61	10.64±0.72	14(8/6)	14(8/6)	Mode of motion: Aerobic Exercise<br>Exercise time:60 min<br>Exercise frequency: four times per week<br>Exercise intensity:70-85%HRmax	Mode of motion: daily activities	16 weeks	16 weeks	1,2,3,8
Roh et al.	2020	Korea	Overweight and obesity	12.60±0.52	12.50±0.53	10(7/3)	10(7/3)	Mode of motion: taekwondo training<br>Exercise time:60 min<br>Exercise frequency: five times per week<br>Exercise intensity: medium and low intensity	Mode of motion: daily activities	16 weeks	16 weeks	1,4
Romero-Perez et al.	2020	Mexico	Obesity	10.02±0.79	10.02±0.79	54(24/30)	51(21/30)	Mode of motion: aerobic exercise + playful activities<br>Exercise time:50 min<br>Exercise frequency: two times per week<br>Exercise intensity: medium and low intensity	Mode of motion: daily activities	20 weeks	20 weeks	16,19
Salus et al.(1)	2022	Estonia	Obesity	13.1±0.3	13.7±0.4	14(14/0)	14(14/0)	Mode of motion: cycling<br>Exercise time:29-30 min<br>Exercise frequency: three times per week<br>Exercise intensity: high intensity	Mode of motion: daily activities	12 weeks	12 weeks	2,3,5,6,7,8,9
Salus et al.(2)	2022	Estonia	Obesity	13.1±1.3	13.7±1.6	14(14/0)	14(14/0)	Mode of motion: cycling<br>Exercise time:29-30 min<br>Exercise frequency: three times per week<br>Exercise intensity: high intensity	mode of motion: daily activities	12 weeks	12 weeks	5,10,11,12,13,14,15
Savoye et al.	2007	America	Obesity	12.0±2.4	12.2±2.1	75(33/42)	44(17/27)	Mode of motion: aerobic exercise<br>Exercise time:50 min<br>Exercise frequency: two times per week/mouth<br>Exercise intensity:65-80%HRmax	Mode of motion: daily activities	48 weeks	48 weeks	1,3,6,7,10,11,12,13,14,15

Author	Year	Country	Weight status	Age		Sample		Intervention measure		Intervention duration		Outcome indicator
				experimental group	control group	experimental group (boys/girls)	control group (boys/girls)	experimental group	control group			
Savoye et al.	2011	America	Obesity	12.0±2.5	12.5±2.3	75	44	Mode of motion: aerobic exercise<br>Exercise time:50-100 min<br>Exercise frequency: twice a week for 6 months and then twice a month<br>Exercise intensity:65-80%HRmax	Mode of motion: daily activities	48 weeks	48 weeks	1,3,6,7,10,11,12,13,14,15
Saygın et al.	2011	Turkey	Obesity	10-12	10-12	20(0/20)	19(0/19)	Mode of motion: aerobic exercise<br>Exercise time:60-90 min<br>Exercise frequency: three times per week<br>Exercise intensity:50-60%HRmax	Mode of motion: daily activities	12 weeks	12 weeks	1,4,6,9,10,11,12,13
Schranz et al.	2014	Australia	Overweight and obesity	14.9±1.4	15.1±1.6	26(26/0)	23(23/0)	Mode of motion: resistance exercise<br>Exercise time:75 min<br>Exercise frequency: three times per week<br>Exercise intensity: medium and low intensity	mode of motion: daily activities	24 weeks	24 weeks	1,3,17,18
Seo et al.	2012	Korea	Obesity	14.70±0.48	14.60±0.96	10(10/0)	10(10/0)	Mode of motion: yoga(asana)<br>Exercise time:60 min<br>Exercise frequency: three times per week<br>Exercise intensity:40-60%HRR	Mode of motion: daily activities	8 weeks	8 weeks	1,3,10,11,12,13,14,15
Seo et al.	2019	Korea	Obesity	12.80±1.72	12.09±2.20	26(17/9)	44(28/16)	Mode of motion: aerobic exercise<br>Exercise time:60 min<br>Exercise frequency: three times per week<br>Exercise intensity:60-90%HRmax	Mode of motion: daily activities	16 weeks	16 weeks	2,3,6,7,11,12,13
Shaibi et al.	2006	America	Overweight	15.1±0.5	15.6±0.5	11(11/0)	11(11/0)	Mode of motion: resistance exercise<br>Exercise time: not exceed 60 min in duration<br>Exercise frequency: twice times per week<br>Exercise intensity: medium and low intensity	Mode of motion: daily activities	16 weeks	16 weeks	1,3,14,15
Shalitin et al.	2009	Israel	Obesity	8.2±1.56	8.51±1.52	55(32/23)	55(26/29)	Mode of motion: aerobic and resistance exercise<br>Exercise time:90 min<br>Exercise frequency: three times per week<br>Exercise intensity: medium and low intensity	Mode of motion: daily activities	12 weeks	12 weeks	1,2,3,6,7,10,11,12,13,14,15
Shu et al.	2023	China	Obesity	11.0±0.8	11.0±0.8	31	16	Mode of motion: running<br>Exercise time: 20-40 min<br>Exercise frequency: three times per week<br>Exercise intensity: 60-80%/100-120% MAS(70%~80% />80% HRmax)	Mode of motion: daily activities	12weeks	12weeks	1,2,3,4,6,7,8,9

Author	Year	Country	Weight status	Age		Sample		Intervention measure		Intervention duration		Outcome indicator
				experimental group	control group	experimental group (boys/girls)	control group (boys/girls)	experimental group	control group			
Sigal et al.	2014	Canada	Overweight and obesity	14.1-17.4	15.6±1.3	228(67/161)	76(24/52)	Mode of motion: aerobic and resistance exercise<br>Exercise time:20-90 min<br>Exercise frequency: four times per week<br>Exercise intensity:65-85%HRmax	Mode of motion: daily activities	22 weeks	22 weeks	1,2,3,6,7
Silva et al.	2012	Brazil	Overweight	13-17	13-17	9	5	Mode of motion: aerobic exercise<br>Exercise time:30-40 min<br>Exercise frequency: three times per week<br>Exercise intensity:85%HRmax	Mode of motion: daily activities	12 weeks	12 weeks	1,3,8
Son et al.	2017	Korea	Obesity	15±1	15±1	20(0/20)	20(0/20)	Mode of motion: aerobic and resistance exercise<br>Exercise time:60 min<br>Exercise frequency: three times per week<br>Exercise intensity:40-70%HRR	Mode of motion: daily activities	12 weeks	12 weeks	1,2,3,6,7,14,15
Staiano et al.	2013	America	Overweight and obesity	15-19	15-19	19	16	Mode of motion: exergame<br>Exercise time: 30-60 min<br>Exercise frequency: five times per week<br>Exercise intensity: medium and low intensity	mode of motion: daily activities	10-20 weeks	10-20 weeks	17
Staiano et al.	2017	America	Overweight and obesity	15.3±1.2	16.1±1.4	20(0/20)	18(0/18)	Mode of motion: dance exergaming<br>Exercise time:60 min<br>Exercise frequency: three times per week<br>Exercise intensity:60-75%HRmax	Mode of motion: daily activities	12 weeks	12 weeks	2,3,10,11,12,13,14,15
Staiano et al.	2018	America	Overweight and obesity	11.2±0.8	11.2±0.8	22	23	Mode of motion: gaming console<br>Exercise time:10-60 min<br>Exercise frequency: three times per week<br>Exercise intensity: medium and low intensity	Mode of motion: daily activities	24 weeks	24 weeks	3,10,11,12,13,14
Suarez-Villadat et al.	2020	Spain	Obesity	14.3±1.25	14.11±1.21	15(8/7)	30(17/13)	Mode of motion: swimming<br>Exercise time:50 min<br>Exercise frequency: three times per week<br>Exercise intensity:110-180 beats per minute	Mode of motion: daily activities	36 weeks	36 weeks	1,2,3
Suarez-Villadat et al.	2023	Spanish	Overweight and obesity	14.83±1.17	14.98±0.95	24(14/10)	25(16/9)	Mode of motion: exergames<br>Exercise time:60 min<br>Exercise frequency: three times per week<br>Exercise intensity: medium and low intensity	Mode of motion: daily activities	20 weeks	20 weeks	1,2,3

Author	Year	Country	Weight status	Age		Sample		Intervention measure		Intervention duration		Outcome indicator
				experimental group	control group	experimental group (boys/girls)	control group (boys/girls)	experimental group	control group			
Sun et al.	2011	China	Overweight	13.6±0.7	13.6±0.7	25(18/7)	17(7/10)	Mode of motion: aerobic exercise<br>Exercise time:60 min<br>Exercise frequency: four times per week<br>Exercise intensity:40-60%VO_2max	Mode of motion: daily activities	10 weeks	10 weeks	1,2,3,4,10,11,12,13
Tan et al.	2010	China	Obesity	9.4±0.5	9.5±0.5	30(17/13)	30(17/13)	Mode of motion: aerobic exercise<br>Exercise time:50 min<br>Exercise frequency: five times per week<br>Exercise intensity:average165beat/min	Mode of motion: daily activities	8 weeks	8 weeks	1,2
Tan et al.	2016	China	Obesity	9.0±0.9	9.4±1.3	11(11/0)	13(13/0)	Mode of motion: aerobic exercise<br>Exercise time:60 min<br>Exercise frequency: five times per week<br>Exercise intensity: individualized HR of FAT max intensity(average140±6beats/min)	Mode of motion: daily activities	10weeks	10weeks	1,3,4
Tas et al.	2023	America	Obesity	15.2±1.5	15.4±1.0	31(14/17)	6(2/4)	Mode of motion: running or biking(HIIT)<br>Exercise time:45 min<br>Exercise frequency: three times per week<br>Exercise intensity:80-90%HRmax	Mode of motion: daily activities	4 weeks	4 weeks	3,5,10,11,12,13,14,15
Tjonna et al.	2009	Norway	Overweight and obesity	13.9±0.3	14.2±0.3	20	22	Mode of motion: aerobic exercise<br>Exercise time:40 min<br>Exercise frequency: two times per week<br>Exercise intensity:70-95%Hfmax	Mode of motion: daily activities	12 weeks	12 weeks	1,2,3,4,6,7,11,12,14, 15,20
Toulabi et al.	2012	Iran	Obesity	15.87±1.0	15.87±1.0	76	76	Mode of motion: aerobic exercise<br>Exercise time:60 min<br>Exercise frequency: three times per week<br>Exercise intensity: medium and low intensity	Mode of motion: daily activities	6 weeks	6 weeks	1,2
Tsang et al.	2009	Australia	Overweight and Obesity	13.1±2.1	13.1±2.1	11(5/6)	8(3/5)	Mode of motion: kung fu<br>Exercise time:60 min<br>Exercise frequency: three times per week<br>Exercise intensity: medium and low intensity	Mode of motion: Tai Chi	24 weeks	24 weeks	10,11,12,13,14,15,20
Vasconcellos et al.	2016	Brazil	Obesity	14.1±1.1	14.8±1.4	10(8/2)	10(6/4)	Mode of motion: soccer program<br>Exercise time:60 min<br>Exercise frequency: three times per week<br>Exercise intensity: high intensity	Mode of motion: daily activities	12 weeks	12 weeks	1,2,3,5,6,7,10,11,12, 13,14,15

Author	Year	Country	Weight status	Age		Sample		Intervention measure		Intervention duration		Outcome indicator
				experimental group	control group	experimental group (boys/girls)	control group (boys/girls)	experimental group	control group			
Velez et al.	2010	America	Overweight and obesity	16.14±0.19	16.14±0.19	13(8/5)	15(8/7)	Mode of motion: resistance training<br>Exercise time:35-40 min<br>Exercise frequency: three times per week<br>Exercise intensity: medium intensity	Mode of motion: daily activities	12 weeks	12 weeks	1,3
Wagener et al.	2012	America	Obesity	14±1.66	14±1.66	20	20	Mode of motion: exergame exercise (Dance Dance Revolution)<br>Exercise time: 40-75 min<br>Exercise frequency: three times per week<br>Exercise intensity: medium and low intensity	mode of motion: daily activities	10 weeks	10 weeks	16,17,19
Weintraub et al.	2008	America	Overweight	10-11	10-11	9	12	Mode of motion: after-school soccer program<br>Exercise time:75 min<br>Exercise frequency: three to four times per week<br>Exercise intensity: medium and low intensity	mode of motion: daily activities	24 weeks	24 weeks	1,16,17
Wen et al.	2008	China	Obesity	12.6±0.8	12.2±0.1	24	13	Mode of motion: aerobic and resistance exercise<br>Exercise time:120-150/50-60mins<br>Exercise frequency: four to five times per week<br>Exercise intensity:3-7MET	Mode of motion: daily activities	36 weeks	36 weeks	10,11,12,13,14,15
Williams et al.	2019	America	Overweight and Obesity	10.4±0.87	10.4±0.89	90(30/60)	85(38/47)	Mode of motion: aerobic activities and games<br>Exercise time:40 min<br>Exercise frequency: seven times per week<br>Exercise intensity: high intensity	Mode of motion: daily activities	32 weeks	32 weeks	3,5,16,18
Williams et al.	2024	New Zealand	Overweight and Obesity	14.8±1.6	14.9±1.4	10	11	Mode of motion: running<br>Exercise time: 49.1±10.6 min<br>Exercise frequency: 6-10 times per 10 weeks<br>Exercise intensity: unclear	Mode of motion: daily activities	10 weeks	10 weeks	1,3,20
Wong et al.	2008	Singapore	Obesity	13.75±1.06	14.25±1.54	12(12/0)	12(12/0)	Mode of motion: aerobic exercises, resistance training and game activities<br>Exercise time:45-60 min<br>Exercise frequency: two times per week<br>Exercise intensity:65-85%HRmax	Mode of motion: daily activities	12 weeks	12 weeks	1,3,6,7,9,10,11,12,13, 14
Wong et al.	2018	America	Obesity	15.2±1.2	15.3±1.1	15(0/15)	15(0/15)	Mode of motion: aerobic and resistance exercise<br>Exercise time:60 min<br>Exercise frequency: three times per week<br>Exercise intensity:40-70%HRR	Mode of motion: daily activities	12 weeks	12 weeks	1,2,3,9,14,15

Author	Year	Country	Weight status	Age		Sample		Intervention measure		Intervention duration		Outcome indicator
				experimental group	control group	experimental group (boys/girls)	control group (boys/girls)	experimental group	control group			
Woo et al.	2004	China	Overweight and obesity	10.1±1.0	10.0±0.9	41(27/14)	41(27/14)	Mode of motion: Aerobic training<br>Exercise time:75 min<br>Exercise frequency: two times per week<br>Exercise intensity:60-70%HRmax	Mode of motion: daily activities	6 weeks	6 weeks	1,3,10,11,12,13,14
Woo et al.	2012	Korean	Overweight	11.30±1.17	11.30±1.17	10	10	Mode of motion: aerobic exercise<br>Exercise time:60 min<br>Exercise frequency: three times per week<br>Exercise intensity:45-65%HRR	mode of motion: daily activities	12weeks	12weeks	3,5,6,7,10,11
Wu et al.	2017	China	Overweight and obesity	16.22±1.47	17.79±0.37	14(6/8)	14(5/9)	Mode of motion: aerobic and resistance exercise<br>Exercise time:50 min<br>Exercise frequency: five times per week<br>Exercise intensity:84%HRmax	Mode of motion: daily activities	12 weeks	12 weeks	1,3,8
Xianbo et al.	2011	China	Overweight and Obesity	13.6±0.7	13.6±0.7	25(18/7)	17(7/10)	Mode of motion: aerobic activities<br>Exercise time:60 min<br>Exercise frequency: four times per week<br>Exercise intensity: medium and low intensity	Mode of motion: daily activities	10 weeks	10 weeks	1,2,3,10,13,14
Xinhui et al.	2016	China	Obesity	13.12±1.32	13.12±1.32	14(7/7)	14(7/7)	Mode of motion: aerobic exercise<br>Exercise time:50-100 min<br>Exercise frequency: three times per week<br>Exercise intensity:60-70%VO_2peak	Mode of motion: daily activities	24 weeks	24 weeks	1,2,10,11,12,13
Xishao et al.	2009	China	Obesity	8.3±1.2	8.3±1.2	34	33	Mode of motion: aerobic exercise<br>Exercise time:60 min<br>Exercise frequency: five times per week<br>Exercise intensity: 140-160 bpm HR	Mode of motion: daily activities	12 weeks	12 weeks	10,11,14
Xishao et al.	2010	China	Obesity	8.3±1.2	8.3±1.2	100	100	Mode of motion: aerobic exercise<br>Exercise time:60 min<br>Exercise frequency: five times per week<br>Exercise intensity:50% of VO_2peak (65% of HRpeak)	Mode of motion: daily activities	24 weeks	24 weeks	10,11,14
Yackobovitch-Gavan et al.	2009	Israel	Obesity	8.2±0.2	8.5±0.2	55(32/23)	55(26/29)	Mode of motion: aerobic and resistance training exercises<br>Exercise time:90 min<br>Exercise frequency: three times per week<br>Exercise intensity:75%HRmax	Mode of motion: daily activities	12 weeks	12 weeks	1,3

Author	Year	Country	Weight status	Age		Sample		Intervention measure		Intervention duration		Outcome indicator
				experimental group	control group	experimental group (boys/girls)	control group (boys/girls)	experimental group	control group			
Yanqin et al.	2017	China	Obesity	10.3±1.21	10.7±1.34	35(18/17)	33(17/16)	Mode of motion: aerobic exercise<br>Exercise time:30-60 min<br>Exercise frequency: three-five times per week<br>Exercise intensity:75%HRmax	Mode of motion: daily activities	48 weeks	48 weeks	1,2,10,11
Youssef et al.	2015	France	Overweight and Obesity	16.1±0.3	16.3 ± 0.5	14(0/14)	9(0/9)	Mode of motion: aerobic exercises<br>Exercise time:40-90 min<br>Exercise frequency: three times per week<br>Exercise intensity:70-100% HRrest	Mode of motion: daily activities	12 weeks	12 weeks	1,3,5
Yu et al.	2008	China	Overweight and Obesity	10.5±1.0	10.5±1.0	41	41	Mode of motion: aerobic and resistance training<br>Exercise time:75 min<br>Exercise frequency: three times per week<br>Exercise intensity: 60%-70%HRmax	Mode of motion: daily activities	6weeks	6weeks	1,3
Yu et al.	2020	China	Obesity	9.9±0.7	9.7±0.6	99(82/17)	72(54/18)	Mode of motion: aerobic activities<br>Exercise time:60 min<br>Exercise frequency: five times per week<br>Exercise intensity: medium and low intensity	Mode of motion: daily activities	32 weeks	32 weeks	1,2,6,7,11,12,14,16,19
Yuanyuan et al.	2019	China	Obesity	11.13±1.26	11.14±1.28	29(19/10)	10(6/4)	Mode of motion: football exercise<br>Exercise time:50 min<br>Exercise frequency: three times per week<br>Exercise intensity:54-85%HRmax	Mode of motion: daily activities	16 weeks	16 weeks	1,2,3,6,7
Zehsaz et al.	2016	Iran	Obesity	10.8±0.9	10.3±0.9	16(16/0)	16(16/0)	Mode of motion: walking and band exercises<br>Exercise time:85-90 min<br>Exercise frequency: two times per week<br>Exercise intensity:55-75%HRmax	Mode of motion: daily activities	16 weeks	16 weeks	1,2,3,10,11,12,13,14, 15
Zehsaz et al.	2017	Iran	Overweight and obesity	10.8±0.9	10.3±0.9	16(16/0)	16(16/0)	Mode of motion: aerobic and resistance exercises<br>Exercise time:60 min<br>Exercise frequency: two times per week<br>Exercise intensity:65-85%HRmax	Mode of motion: daily activities	16 weeks	16 weeks	1,2,3,10,11,12,13,14
Zhaogang et al.	2021	China	Obesity	16.18±0.79	15.36±0.68	49(19/30)	49(26/23)	Mode of motion: aerobic exercise<br>Exercise time: 30 min<br>Exercise frequency: 7 times per week<br>Exercise intensity: medium and low intensity	Mode of motion: daily activities	48 weeks	48 weeks	6,7,10,14

Author	Year	Country	Weight status	Age		Sample		Intervention measure		Intervention duration		Outcome indicator
				experimental group	control group	experimental group (boys/girls)	control group (boys/girls)	experimental group	control group			
Zhihui	2017	China	Obesity	16.28±3.03	16.83±3.52	40	40	Mode of motion: core strength training<br>Exercise time: 150 min<br>Exercise frequency: once a day<br>Exercise intensity: high intensity	Mode of motion: daily activities	4 weeks	4 weeks	1,2,3

Notes: 1. BMI, 2. WC, 3. BF%, 4. VO_2max, 5. VO_2peak, 6. SBP, 7. DBP, 8. HRmax, 9. HRrest, 10. TC, 11. TG, 12. HDL-C, 13. LDL-C, 14. FPG, 15. FINS, 16. Depression, 17. Self-esteem, 18. Self-worth, 19. Anxiety, 20. HbA1c

BMI, body mass index; WC, waist circumference; BF%, percentage of body fat; VO_2max, maximal oxygen consumption; VO_2peak, peak oxygen uptake; HRmax, max heart rate; HRrest, resting heart rate; SBP, systolic blood pressure; DBP, diastolic blood pressure; TC, total cholesterol; TG, triglyceride; HDL-C, high-density lipoprotein cholesterol; LDL-C, low-density lipoprotein cholesterol; FPG, fasting plasma glucose; HbA1c, hemoglobin A1c; FINS, fasting insulin; MAS, maximal aerobic speed; MET, metabolic equivalent; HRR, heart rate reserve; MAP, heart rate reserve; Hfmax, maximal heart frequency; RM, repetition maximum; HIIT, High-Intensity Interval Training; MICT, Moderate-Intensity Continuous Training.

Table S2. Summary of subgroup analyses of meta-analysis results of exercise interventions in children and adolescents with overweight or obesity

Health outcomes	k	MD[95%CI]	I^2(%)	P-value of Q test	Subgroup Differences
**BMI**					
Type of exercise					
Aerobic	93	-1.49 [-1.82, -1.17]	76	＜0.00001**	0.04*
Anaerobic	25	-1.20 [-1.91, -0.50]	63	0.0008**	
Resistance	12	0.18 [-1.10, 1.45]	44	0.79	
Combined aerobic and resistance	37	-0.83 [-1.47, -0.19]	69	0.01*	
Exercise intensity					
Moderate-to-low	97	-1.50 [-1.89, -1.11]	78	＜0.00001**	0.02*
High	70	-0.90 [-1.24, -0.55]	58	＜0.00001**	
Duration per session					
<50 min	56	-1.17 [-1.58, -0.76]	75	＜0.00001**	0.70
⩾50 min	111	-1.28 [-1.63, -0.93]	72	＜0.00001**	
Frequency					
<3 sessions/week	40	-0.70 [-1.15, -0.24]	37	0.003**	0.01*
⩾3 sessions/week	127	-1.41 [-1.71, -1.10]	76	＜0.00001**	
Weekly duration					
<150 min/week	65	-1.03 [-1.47, -0.58]	69	＜0.00001**	0.22
⩾150 min/week	102	-1.37 [-1.70, -1.05]	74	＜0.00001**	
Intervention duration					
<12 weeks	47	-1.03 [-1.62, -0.44]	71	0.0007**	0.40
⩾12 weeks	120	-1.31 [-1.61, -1.02]	73	＜0.00001**	
Region					
Asia	71	-1.52 [-1.86, -1.17]	76	＜0.00001**	0.004**
Europe	31	-0.75 [-1.44, -0.05]	77	0.04*	
Africa	12	-3.01 [-4.41, -1.61]	53	＜0.0001**	
North America	31	-0.84 [-1.46, -0.23]	54	0.007**	
South America	15	-0.98 [-1.71, -0.25]	30	0.009**	
Oceania	7	-0.07 [-1.11, 0.98]	18	0.90	
Economic status					
Lower-middle income	13	-2.82 [-4.14, -1.50]	56	＜0.0001**	0.0004**

Health outcomes	k	MD[95%CI]	I^2(%)	P-value of Q test	Subgroup Differences
Upper-middle income	72	-1.55 [-1.91, -1.19]	75	＜0.00001**	
High income	82	-0.74 [-1.10, -0.38]	62	＜0.0001**	
Weight status					
Overweight	20	-0.69 [-1.42, 0.03]	53	0.06	0.02*
Obesity	91	-1.70 [-2.11, -1.29]	77	＜0.00001**	
Sex					
Boys	31	-1.61 [-2.27, -0.95]	67	＜0.00001**	0.83
Girls	26	-1.71 [-2.35, -1.08]	49	＜0.00001**	
Metabolic status					
MHO	9	-0.19 [-0.71, 0.34]	0	0.49	0.004**
MUO	15	-1.80 [-2.78, -0.83]	81	0.0003**	
**WC**					
Type of exercise					
Aerobic	58	-2.93 [-3.87, -1.99]	74	＜0.00001**	0.72
Anaerobic	24	-3.47 [-4.89, -2.05]	37	＜0.00001**	
Resistance	6	-0.93 [-6.78, 4.92]	76	0.76	
Combined aerobic and resistance	22	-2.64 [-3.62, -1.67]	25	＜0.00001**	
Exercise intensity					
Moderate-to-low	62	-3.14 [-4.03, -2.24]	72	＜0.00001**	0.24
High	48	-2.35 [-3.29, -1.42]	41	＜0.00001**	
Duration per session					
<50 min	41	-1.99 [-2.62, -1.36]	0	＜0.00001**	0.02*
⩾50 min	69	-3.34 [-4.28, -2.39]	74	＜0.00001**	
Frequency					
<3 sessions/week	18	-1.99 [-3.17, -0.81]	13	0.0010**	0.14
⩾3 sessions/week	92	-3.04 [-3.79, -2.30]	68	＜0.00001**	
Weekly duration					
<150 min/week	43	-1.83 [-2.52, -1.13]	0	＜0.00001**	0.006**
⩾150 min/week	67	-3.43 [-4.34, -2.52]	75	＜0.00001**	
Intervention duration					
<12 weeks	25	-3.35 [-5.72, -0.99]	79	0.005**	0.58
⩾12 weeks	85	-2.66 [-3.28, -2.03]	53	＜0.00001**	

Health outcomes	k	MD[95%CI]	I^2(%)	P-value of Q test	Subgroup Differences
Region					
Asia	46	-3.74 [-4.96, -2.51]	80	＜0.00001**	0.12
Europe	15	-1.48 [-2.41, -0.55]	0	0.002**	
Africa	18	-2.18 [-3.33, -1.04]	0	0.0002**	
North America	18	-2.33 [-3.32, -1.33]	25	＜0.00001**	
South America	11	-2.08 [-3.77, -0.39]	25	0.02*	
Oceania	2	0.53 [-7.98, 9.05]	90	0.90	
Economic status					
Lower-middle income	19	-2.21 [-3.32, -1.09]	0	0.0001**	0.01*
Upper-middle income	46	-4.05 [-5.30, -2.79]	80	＜0.00001**	
High income	45	-1.92 [-2.53, -1.30]	14	＜0.00001**	
Weight status					
Overweight	10	-1.16 [-2.67, 0.35]	10	0.13	0.002**
Obesity	61	-3.93 [-4.88, -2.98]	74	＜0.00001**	
Sex					
Boys	14	-4.39 [-7.42, -1.35]	79	0.005**	0.47
Girls	31	-3.18 [-4.37, -2.00]	72	＜0.00001**	
Metabolic status					
MHO	9	-0.87 [-2.17, 0.42]	0	0.18	0.05
MUO	10	-5.51 [-9.95, -1.08]	90	0.01*	
**BF%**					
Type of exercise					
Aerobic	74	-2.59 [-3.12, -2.05]	70	＜0.00001**	0.12
Anaerobic	32	-2.27 [-3.36, -1.17]	77	＜0.0001**	
Resistance	7	-0.75 [-2.15, 0.66]	0	0.30	
Combined aerobic and resistance	33	-2.32 [-3.36, -1.29]	65	＜0.0001**	
Exercise intensity					
Moderate-to-low	82	-2.61 [-3.18, -2.03]	72	＜0.00001**	0.26
High	64	-2.11 [-2.75, -1.47]	67	＜0.00001**	
Duration per session					
<50 min	60	-1.60 [-2.14, -1.07]	60	＜0.00001**	0.0008**
⩾50 min	86	-2.99 [-3.60, -2.38]	71	＜0.00001**	

Health outcomes	k	MD[95%CI]	I^2 (%)	P-value of Q test	Subgroup Differences
Frequency					
<3 sessions/week	34	-2.45 [-3.16, -1.73]	33	＜0.00001**	0.88
⩾3 sessions/week	112	-2.38 [-2.87, -1.88]	75	＜0.00001**	
Weekly duration					
<150 min/week	67	-1.83 [-2.36, -1.30]	56	＜0.00001**	0.01*
⩾150 min/week	79	-2.86 [-3.49, -2.24]	75	＜0.00001**	
Intervention duration					
<12 weeks	37	-2.54 [-3.41, -1.66]	62	＜0.00001**	0.70
⩾12 weeks	109	-2.34 [-2.83, -1.85]	72	＜0.00001**	
Region					
Asia	51	-3.46 [-4.25, -2.68]	80	＜0.00001**	0.0005**
Europe	28	-2.32 [-3.48, -1.15]	56	0.0001**	
Africa	19	-1.19 [-2.02, -0.37]	62	0.004**	
North America	28	-1.69 [-2.42, -0.96]	45	＜0.00001**	
South America	14	-2.47 [-3.51, -1.43]	15	＜0.00001**	
Oceania	6	-0.63 [-2.05, 0.79]	0	0.39	
Economic status					
Lower-middle income	19	-1.19 [-2.02, -0.37]	62	0.004**	0.0003**
Upper-middle income	53	-3.46 [-4.26, -2.66]	80	＜0.00001**	
High income	74	-1.90 [-2.40, -1.40]	41	＜0.00001**	
Weight status					
Overweight	16	-1.94 [-3.04, -0.85]	52	0.0005**	0.08
Obesity	82	-3.06 [-3.67, -2.46]	76	＜0.00001**	
Sex					
Boys	27	-2.56 [-3.76, -1.36]	67	＜0.0001**	0.38
Girls	30	-1.94 [-2.66, -1.22]	75	＜0.00001**	
Metabolic status					
MHO	10	-1.18 [-2.38, 0.02]	0	0.05	0.002**
MUO	10	-4.03 [-5.39, -2.68]	63	＜0.00001**	
**VO_2max**					
Type of exercise					
Aerobic	21	2.93 [2.08, 3.78]	77	＜0.00001**	0.64

Health outcomes	k	MD[95%CI]	I^2 (%)	P-value of Q test	Subgroup Differences
Anaerobic	7	3.42 [1.76, 5.08]	67	＜0.0001**	
Resistance	1	1.80 [-2.17, 5.77]	-	0.37	
Combined aerobic and resistance	9	2.11 [0.62, 3.61]	71	0.006**	
Exercise intensity					
Moderate-to-low	25	2.63 [1.79, 3.47]	78	＜0.00001**	0.46
High	13	3.20 [1.97, 4.42]	75	＜0.00001**	
Duration per session					
<50 min	14	3.30 [2.42, 4.18]	50	＜0.00001**	0.26
⩾50 min	24	2.60 [1.75, 3.45]	79	＜0.00001**	
Frequency					
<3 sessions/week	9	2.25 [0.36, 4.15]	71	0.02*	0.50
⩾3 sessions/week	29	2.96 [2.22, 3.69]	79	＜0.00001**	
Weekly duration					
<150 min/week	16	2.96 [1.98, 3.93]	61	＜0.00001**	0.77
⩾150 min/week	22	2.76 [1.87, 3.65]	81	＜0.00001**	
Intervention duration					
<12 weeks	21	3.04 [1.88, 4.21]	79	＜0.00001**	0.57
⩾12 weeks	17	2.62 [1.77, 3.47]	75	＜0.00001**	
Region					
Asia	19	3.04 [2.11, 3.97]	81	＜0.00001**	0.01*
Europe	10	1.82 [0.35, 3.28]	65	0.02*	
Africa	5	4.84 [2.94, 6.73]	68	＜0.00001**	
North America	3	1.43 [-0.47, 3.32]	0	0.14	
South America	0	-	-	-	
Oceania	1	1.10 [-0.32, 2.52]	-	0.13	
Economic status					
Lower-middle income	5	4.84 [2.94, 6.73]	68	＜0.00001**	＜0.0001**
Upper-middle income	15	3.63 [2.63, 4.63]	77	＜0.00001**	
High income	18	1.31 [0.49, 2.14]	54	0.002**	
Weight status					
Overweight	3	2.44 [-1.10, 5.98]	81	0.18	0.90
Obesity	21	2.67 [1.76, 3.59]	81	＜0.00001**	

Health outcomes	k	MD[95%CI]	I^2(%)	P-value of Q test	Subgroup Differences
Sex					
Boys	9	3.69 [2.76, 4.61]	20	＜0.00001**	0.93
Girls	5	3.57 [1.33, 5.81]	91	0.002**	
**VO_2peak**					
Type of exercise					
Aerobic	19	2.61 [1.58, 3.65]	67	＜0.00001**	0.77
Anaerobic	13	3.02 [1.54, 4.51]	76	＜0.0001**	
Resistance	4	2.59 [-1.63, 6.81]	80	0.23	
Combined aerobic and resistance	8	2.03 [0.85, 3.21]	0	0.0008**	
Exercise intensity					
Moderate-to-low	16	2.91 [1.66, 4.15]	69	＜0.00001**	0.49
High	28	2.37 [1.49, 3.25]	62	＜0.00001**	
Duration per session					
<50 min	28	2.12 [1.39, 2.86]	59	＜0.00001**	0.13
⩾50 min	16	3.46 [1.89, 5.03]	66	＜0.0001**	
Frequency					
<3 sessions/week	5	0.92 [-0.65, 2.48]	11	0.25	0.03*
⩾3 sessions/week	39	2.79 [2.03, 3.55]	67	＜0.00001**	
Weekly duration					
<150 min/week	26	2.21 [1.41, 3.02]	59	＜0.00001**	0.21
⩾150 min/week	18	3.25 [1.85, 4.65]	72	＜0.00001**	
Intervention duration					
<12 weeks	6	0.33 [-2.16, 2.81]	57	0.80	0.06
⩾12 weeks	38	2.81 [2.06, 3.56]	66	＜0.00001**	
Region					
Asia	7	3.72 [2.08, 5.36]	54	＜0.00001**	＜0.0001**
Europe	6	1.65 [-0.47, 3.77]	57	0.13	
Africa	4	0.57 [-0.08, 1.22]	0	0.08	
North America	16	2.98 [1.58, 4.38]	72	＜0.0001**	
South America	7	3.62 [2.40, 4.85]	3	＜0.00001**	
Oceania	4	1.11 [-1.41, 3.63]	19	0.39	
Economic status					

Health outcomes	k	MD[95%CI]	I^2(%)	P-value of Q test	Subgroup Differences
Lower-middle income	4	0.57 [-0.08, 1.22]	0	0.08	＜0.00001**
Upper-middle income	13	3.71 [2.56, 4.86]	39	＜0.00001**	
High income	27	2.48 [1.48, 3.48]	64	＜0.00001**	
Weight status					
Overweight	6	2.57 [1.33, 3.80]	0	＜0.0001**	0.49
Obesity	27	3.14 [2.09, 4.18]	74	＜0.00001**	
Sex					
Boys	8	5.70 [4.22, 7.19]	37	＜0.00001**	＜0.00001**
Girls	10	1.14 [0.26, 2.03]	43	0.01*	
Metabolic status					
MHO	3	1.11 [-0.30, 2.51]	24	0.12	0.002**
MUO	5	4.48 [2.82, 6.15]	15	＜0.00001**	
**HRmax**					
Type of exercise					
Aerobic	12	-0.25 [-1.63, 1.13]	51	0.73	＜0.0001**
Anaerobic	10	-1.57 [-2.82, -0.33]	39	0.01*	
Resistance	0	-	-	-	
Combined aerobic and resistance	1	11.50 [5.79, 17.21]	-	＜0.0001**	
Exercise intensity					
Moderate-to-low	9	0.18 [-1.60, 1.96]	59	0.84	0.33
High	14	-0.96 [-2.45, 0.53]	66	0.21	
Duration per session					
<50 min	16	-0.79 [-1.92, 0.33]	49	0.17	0.48
⩾50 min	7	0.36 [-2.60, 3.33]	79	0.81	
Frequency					
<3 sessions/week	3	0.45 [-4.12, 5.02]	0	0.85	0.68
⩾3 sessions/week	20	-0.55 [-1.72, 0.62]	67	0.36	
Weekly duration					
<150 min/week	17	-0.77 [-1.86, 0.31]	46	0.16	0.50
⩾150 min/week	6	0.42 [-2.87, 3.71]	83	0.80	
Intervention duration					
<12 weeks	4	0.92 [-5.13, 6.97]	51	0.77	0.62

Health outcomes	k	MD[95%CI]	I^2(%)	P-value of Q test	Subgroup Differences
⩾12 weeks	19	-0.64 [-1.76, 0.48]	65	0.26	
Region					
Asia	6	2.10 [-1.93, 6.12]	84	0.31	0.08
Europe	7	0.34 [-1.60, 2.28]	0	0.73	
Africa	9	-1.67 [-2.53, -0.81]	17	0.0001**	
North America	0	-	-	-	
South America	1	-0.50 [-1.96, 0.96]	-	0.50	
Oceania	0	-	-	-	
Economic status					
Lower-middle income	9	-1.67 [-2.53, -0.81]	17	0.0001**	0.03*
Upper-middle income	9	0.61 [-1.68, 2.90]	76	0.60	
High income	5	1.97 [-1.31, 5.25]	12	0.24	
Weight status					
Overweight	3	-0.49 [-1.87, 0.88]	0	0.48	0.61
Obesity	12	0.04 [-1.49, 1.57]	38	0.96	
Sex					
Boys	4	-0.90 [-5.36, 3.57]	77	0.69	0.77
Girls	9	-1.58 [-2.33, -0.82]	1	＜0.0001**	
**HRrest**					
Type of exercise					
Aerobic	13	-2.19 [-3.20, -1.17]	29	＜0.0001**	0.07
Anaerobic	6	-2.82 [-4.74, -0.91]	62	0.004**	
Resistance	0	-	-	-	
Combined aerobic and resistance	6	-5.51 [-8.19, -2.83]	59	＜0.0001**	
Exercise intensity					
Moderate-to-low	16	-2.69 [-3.97, -1.40]	53	＜0.0001**	0.55
High	9	-3.29 [-4.79, -1.80]	59	＜0.0001**	
Duration per session					
<50 min	11	-1.85 [-2.92, -0.78]	38	0.0007**	0.02*
⩾50 min	14	-3.88 [-5.20, -2.55]	47	＜0.00001**	
Frequency					
<3 sessions/week	5	-4.49 [-5.85, -3.13]	0	＜0.00001**	0.02*

Health outcomes	k	MD[95%CI]	I^2(%)	P-value of Q test	Subgroup Differences
⩾3 sessions/week	20	-2.48 [-3.52, -1.44]	56	＜0.00001**	
Weekly duration					
<150 min/week	14	-2.59 [-3.69, -1.49]	54	＜0.00001**	0.34
⩾150 min/week	11	-3.66 [-5.57, -1.74]	52	0.0002**	
Intervention duration					
<12 weeks	7	-2.44 [-4.10, -0.77]	36	0.004**	0.50
⩾12 weeks	18	-3.14 [-4.29, -1.99]	60	＜0.00001**	
Region					
Asia	10	-3.03 [-4.47, -1.58]	74	＜0.0001**	0.80
Europe	7	-4.09 [-7.69, -0.48]	58	0.03*	
Africa	5	-2.58 [-3.69, -1.46]	0	＜0.00001**	
North America	3	-3.64 [-6.59, -0.68]	0	0.02*	
South America	0	-	-	-	
Oceania	0	-	-	-	
Economic status					
Lower-middle income	6	-2.25 [-3.40, -1.10]	7	0.0001**	0.42
Upper-middle income	10	-3.15 [-4.62, -1.68]	67	＜0.0001**	
High income	9	-3.77 [-6.08, -1.46]	56	0.001**	
Weight status					
Overweight	1	-7.40 [-13.97, -0.83]	-	0.03*	0.16
Obesity	17	-2.63 [-3.50, -1.77]	47	＜0.00001**	
Sex					
Boys	6	-4.73 [-6.12, -3.35]	0	＜0.00001**	0.23
Girls	8	-3.47 [-5.00, -1.95]	54	＜0.00001**	
**SBP**					
Type of exercise					
Aerobic	45	-4.26 [-6.13, -2.39]	94	＜0.00001**	0.66
Anaerobic	16	-2.88 [-4.52, -1.24]	18	0.0006**	
Resistance	2	-1.10 [-8.54, 6.33]	67	0.77	
Combined aerobic and resistance	13	-3.62 [-5.56, -1.69]	44	0.0002**	
Exercise intensity					
Moderate-to-low	48	-4.79 [-6.57, -3.00]	93	＜0.00001**	0.007**

Health outcomes	k	MD[95%CI]	I^2(%)	P-value of Q test	Subgroup Differences
High	28	-1.96 [-3.01, -0.92]	16	0.0002**	
Duration per session					
<50 min	35	-2.25 [-3.49, -1.02]	52	0.0003**	0.008**
≥50 min	41	-5.21 [-7.02, -3.40]	92	＜0.00001**	
Frequency					
<3 sessions/week	17	-1.72 [-3.01, -0.42]	10	0.009**	0.01*
≥3 sessions/week	59	-4.43 [-6.05, -2.80]	92	＜0.00001**	
Weekly duration					
<150 min/week	36	-2.24 [-3.25, -1.23]	32	＜0.0001**	0.006**
≥150 min/week	40	-5.38 [-7.35, -3.40]	93	＜0.00001**	
Intervention duration					
<12 weeks	16	-4.26 [-6.84, -1.67]	65	0.001**	0.78
≥12 weeks	60	-3.83 [-5.45, -2.21]	93	＜0.00001**	
Region					
Asia	32	-5.42 [-7.39, -3.46]	92	＜0.00001**	0.010*
Europe	15	-4.82 [-7.11, -2.54]	45	＜0.0001**	
Africa	9	-2.19 [-3.83, -0.55]	0	0.009**	
North America	12	-1.28 [-3.61, 1.05]	78	0.28	
South America	8	-1.36 [-3.61, 0.88]	29	0.23	
Oceania	0	-	-	-	
Economic status					
Lower-middle income	10	-2.16 [-3.68, -0.65]	0	0.005**	0.05
Upper-middle income	30	-5.29 [-7.34, -3.24]	93	＜0.00001**	
High income	36	-2.96 [-4.44, -1.47]	68	＜0.0001**	
Weight status					
Overweight	10	-2.43 [-5.04, 0.18]	43	0.07	0.18
Obesity	45	-4.56 [-6.28, -2.85]	92	＜0.00001**	
Sex					
Boys	12	-4.21 [-6.74, -1.67]	48	0.001**	0.46
Girls	16	-5.54 [-8.06, -3.02]	96	＜0.0001**	
**DBP**					
Type of exercise					

Health outcomes	k	MD[95%CI]	I^2(%)	P-value of Q test	Subgroup Differences
Aerobic	41	-2.04 [-3.39, -0.69]	91	0.003**	0.66
Anaerobic	16	-1.01 [-2.78, 0.75]	53	0.26	
Resistance	2	-0.16 [-3.34, 3.02]	0	0.92	
Combined aerobic and resistance	12	-1.52 [-2.68, -0.35]	0	0.01*	
Exercise intensity					
Moderate-to-low	43	-2.07 [-3.38, -0.77]	90	0.002**	0.19
High	28	-0.98 [-1.98, 0.02]	29	0.05	
Duration per session					
<50 min	35	-0.85 [-1.62, -0.09]	25	0.03*	0.07
⩾50 min	36	-2.38 [-3.87, -0.89]	91	0.002**	
Frequency					
<3 sessions/week	17	-0.57 [-2.00, 0.85]	33	0.43	0.13
⩾3 sessions/week	54	-1.98 [-3.12, -0.85]	89	0.0006**	
Weekly duration					
<150 min/week	36	-0.99 [-1.75, -0.24]	18	0.010*	0.12
⩾150 min/week	35	-2.32 [-3.84, -0.80]	92	0.003**	
Intervention duration					
<12 weeks	13	-2.99 [-4.33, -1.66]	8	＜0.0001**	0.09
⩾12 weeks	58	-1.47 [-2.58, -0.37]	88	0.009**	
Region					
Asia	29	-2.46 [-4.09, -0.83]	92	0.003**	0.08
Europe	13	-2.10 [-3.81, -0.39]	34	0.02*	
Africa	9	-0.36 [-1.47, 0.76]	0	0.53	
North America	12	-0.35 [-1.17, 0.46]	0	0.39	
South America	8	-0.00 [-2.42, 2.42]	41	1.00	
Oceania	0	-	-	-	
Economic status					
Lower-middle income	10	-0.61 [-1.69, 0.46]	0	0.26	0.11
Upper-middle income	29	-2.64 [-4.29, -0.99]	92	0.002**	
High income	32	-0.78 [-1.72, 0.16]	37	0.10	
Weight status					
Overweight	10	0.00 [-1.76, 1.77]	0	1.00	0.09

Health outcomes	k	MD[95%CI]	I^2(%)	P-value of Q test	Subgroup Differences
Obesity	41	-1.92 [-3.25, -0.60]	91	0.004**	
Sex					
Boys	12	-1.88 [-3.36, -0.39]	16	0.01*	0.78
Girls	15	-2.28 [-4.69, 0.12]	96	0.06	
**TC**					
Type of exercise					
Aerobic	52	-0.38 [-0.48, -0.28]	86	＜0.00001**	0.07
Anaerobic	11	-0.45 [-0.59, -0.30]	0	＜0.00001**	
Resistance	4	-0.41 [-0.98, 0.17]	70	0.17	
Combined aerobic and resistance	22	-0.20 [-0.33, -0.07]	42	0.003**	
Exercise intensity					
Moderate-to-low	58	-0.35 [-0.44, -0.27]	82	＜0.00001**	0.83
High	31	-0.33 [-0.53, -0.13]	79	0.0009**	
Duration per session					
<50 min	30	-0.36 [-0.51, -0.20]	86	＜0.0001**	0.95
⩾50 min	59	-0.35 [-0.44, -0.26]	76	＜0.00001**	
Frequency					
<3 sessions/week	15	-0.17 [-0.27, -0.08]	0	0.0003**	0.0007**
⩾3 sessions/week	74	-0.39 [-0.48, -0.31]	82	＜0.00001**	
Weekly duration					
<150 min/week	28	-0.19 [-0.28, -0.10]	18	＜0.0001**	0.0010**
⩾150 min/week	61	-0.41 [-0.51, -0.32]	84	＜0.00001**	
Intervention duration					
<12 weeks	25	-0.34 [-0.52, -0.15]	67	0.0003**	0.83
⩾12 weeks	64	-0.36 [-0.44, -0.27]	83	＜0.00001**	
Region					
Asia	53	-0.45 [-0.55, -0.35]	84	＜0.00001**	＜0.0001**
Europe	8	-0.26 [-0.45, -0.07]	31	0.007**	
Africa	3	-0.45 [-1.03, 0.13]	94	0.13	
North America	11	-0.09 [-0.20, 0.03]	12	0.14	
South America	10	-0.23 [-0.41, -0.05]	0	0.01*	
Oceania	4	-0.02 [-0.31, 0.27]	0	0.89	

Health outcomes	k	MD[95%CI]	I^2(%)	P-value of Q test	Subgroup Differences
Economic status					
Lower-middle income	3	-0.45 [-1.03, 0.13]	94	0.13	＜0.0001**
Upper-middle income	54	-0.46 [-0.55, -0.36]	83	＜0.00001**	
High income	32	-0.14 [-0.24, -0.05]	27	0.003**	
Weight status					
Overweight	14	-0.34 [-0.71, 0.02]	89	0.07	0.74
Obesity	56	-0.41 [-0.49, -0.32]	79	＜0.00001**	
Sex					
Boys	18	-0.71 [-1.01, -0.41]	83	＜0.00001**	0.01*
Girls	13	-0.25 [-0.44, -0.06]	84	0.01*	
Metabolic status					
MHO	10	-0.12 [-0.27, 0.04]	17	0.14	0.35
MUO	14	-0.24 [-0.44, -0.03]	72	0.02*	
**TG**					
Type of exercise					
Aerobic	56	-0.23 [-0.30, -0.16]	94	＜0.00001**	0.36
Anaerobic	11	-0.30 [-0.52, -0.07]	82	0.01*	
Resistance	3	-0.35 [-0.85, 0.15]	67	0.17	
Combined aerobic and resistance	22	-0.16 [-0.22, -0.09]	58	＜0.0001**	
Exercise intensity					
Moderate-to-low	61	-0.24 [-0.31, -0.18]	93	＜0.00001**	0.27
High	31	-0.19 [-0.27, -0.10]	69	＜0.00001**	
Duration per session					
<50 min	30	-0.11 [-0.17, -0.05]	74	0.0003**	0.002**
⩾50 min	62	-0.27 [-0.35, -0.19]	93	＜0.00001**	
Frequency					
<3 sessions/week	18	-0.18 [-0.27, -0.08]	80	0.0004**	0.27
⩾3 sessions/week	74	-0.24 [-0.30, -0.18]	92	＜0.00001**	
Weekly duration					
<150 min/week	30	-0.15 [-0.23, -0.07]	78	0.0002**	0.05
⩾150 min/week	62	-0.26 [-0.33, -0.19]	93	＜0.00001**	
Intervention duration					

Health outcomes	k	MD[95%CI]	I^2 (%)	P-value of Q test	Subgroup Differences
<12 weeks	24	-0.38 [-0.55, -0.20]	84	＜0.0001**	0.04*
⩾12 weeks	68	-0.18 [-0.24, -0.13]	91	＜0.00001**	
Region					
Asia	52	-0.29 [-0.36, -0.22]	94	＜0.00001**	＜0.0001**
Europe	12	-0.08 [-0.13, -0.03]	0	0.002**	
Africa	3	-0.13 [-0.54, 0.27]	98	0.51	
North America	11	-0.06 [-0.13, 0.01]	0	0.09	
South America	11	-0.23 [-0.48, 0.02]	83	0.07	
Oceania	3	-0.26 [-0.54, 0.02]	0	0.07	
Economic status					
Lower-middle income	3	-0.13 [-0.54, 0.27]	98	0.51	0.0002**
Upper-middle income	54	-0.30 [-0.37, -0.23]	88	＜0.00001**	
High income	35	-0.10 [-0.16, -0.04]	68	0.002**	
Weight status					
Overweight	14	-0.21 [-0.30, -0.13]	19	＜0.00001**	0.33
Obesity	56	-0.27 [-0.34, -0.20]	93	＜0.00001**	
Sex					
Boys	18	-0.44 [-0.61, -0.27]	86	＜0.00001**	0.02*
Girls	12	-0.16 [-0.33, 0.01]	94	0.06	
**HDL-C**					
Type of exercise					
Aerobic	51	0.08 [0.05, 0.12]	80	＜0.00001**	0.79
Anaerobic	11	0.06 [-0.01, 0.12]	31	0.07	
Resistance	4	0.10 [0.02, 0.17]	0	0.02*	
Combined aerobic and resistance	22	0.07 [0.02, 0.11]	38	0.003**	
Exercise intensity					
Moderate-to-low	56	0.08 [0.05, 0.12]	79	＜0.00001**	0.65
High	32	0.07 [0.04, 0.10]	18	＜0.00001**	
Duration per session					
<50 min	31	0.06 [0.02, 0.11]	79	0.004**	0.43
⩾50 min	57	0.09 [0.05, 0.12]	65	＜0.00001**	
Frequency					

Health outcomes	k	MD[95%CI]	I^2(%)	P-value of Q test	Subgroup Differences
<3 sessions/week	18	0.05 [0.01, 0.10]	28	0.03*	0.25
⩾3 sessions/week	70	0.08 [0.06, 0.11]	76	＜0.00001**	
Weekly duration					
<150 min/week	32	0.06 [0.02, 0.10]	64	0.005**	0.26
⩾150 min/week	56	0.09 [0.06, 0.12]	75	＜0.00001**	
Intervention duration					
<12 weeks	24	0.04 [0.00, 0.08]	43	0.03*	0.06
⩾12 weeks	64	0.09 [0.06, 0.12]	75	＜0.00001**	
Region					
Asia	48	0.09 [0.05, 0.13]	77	＜0.00001**	0.67
Europe	11	0.07 [0.02, 0.12]	10	0.004**	
Africa	3	0.11 [0.06, 0.17]	65	＜0.0001**	
North America	11	0.04 [-0.05, 0.14]	81	0.34	
South America	11	0.07 [-0.01, 0.15]	45	0.07	
Oceania	4	0.03 [-0.07, 0.13]	0	0.52	
Economic status					
Lower-middle income	3	0.11 [0.06, 0.17]	65	＜0.0001**	0.13
Upper-middle income	50	0.09 [0.05, 0.13]	78	＜0.00001**	
High income	35	0.05 [0.01, 0.09]	53	0.01*	
Weight status					
Overweight	13	0.03 [-0.04, 0.09]	60	0.42	0.12
Obesity	54	0.09 [0.05, 0.12]	74	＜0.00001**	
Sex					
Boys	19	0.07 [-0.01, 0.14]	79	0.07	0.63
Girls	13	0.04 [-0.01, 0.10]	70	0.10	
**LDL-C**					
Type of exercise					
Aerobic	50	-0.28 [-0.38, -0.19]	84	＜0.00001**	0.12
Anaerobic	11	-0.40 [-0.53, -0.27]	3	＜0.00001**	
Resistance	4	-0.35 [-0.68, -0.01]	52	0.04*	
Combined aerobic and resistance	22	-0.19 [-0.31, -0.07]	56	0.002**	
Exercise intensity					

Health outcomes	k	MD[95%CI]	I^2(%)	P-value of Q test	Subgroup Differences
Moderate-to-low	56	-0.28 [-0.37, -0.20]	80	＜0.00001**	0.74
High	31	-0.26 [-0.38, -0.13]	71	＜0.0001**	
Duration per session					
<50 min	30	-0.24 [-0.36, -0.12]	80	＜0.0001**	0.52
⩾50 min	57	-0.29 [-0.38, -0.20]	76	＜0.00001**	
Frequency					
<3 sessions/week	17	-0.10 [-0.19, -0.01]	15	0.03*	0.0003**
⩾3 sessions/week	70	-0.33 [-0.41, -0.25]	80	＜0.00001**	
Weekly duration					
<150 min/week	31	-0.19 [-0.28, -0.10]	53	＜0.0001**	0.04*
⩾150 min/week	56	-0.33 [-0.42, -0.23]	81	＜0.00001**	
Intervention duration					
<12 weeks	25	-0.28 [-0.43, -0.13]	71	0.0002**	0.96
⩾12 weeks	62	-0.28 [-0.36, -0.19]	80	＜0.00001**	
Region					
Asia	48	-0.35 [-0.45, -0.25]	83	＜0.00001**	0.01*
Europe	10	-0.23 [-0.37, -0.09]	45	0.001**	
Africa	3	-0.42 [-0.84, -0.01]	89	0.04*	
North America	11	-0.10 [-0.20, 0.00]	13	0.06	
South America	11	-0.23 [-0.38, -0.07]	30	0.004**	
Oceania	4	-0.02 [-0.26, 0.22]	0	0.89	
Economic status					
Lower-middle income	3	-0.42 [-0.84, -0.01]	89	0.04*	0.0008**
Upper-middle income	50	-0.37 [-0.47, -0.27]	82	＜0.00001**	
High income	34	-0.14 [-0.21, -0.07]	27	0.0002**	
Weight status					
Overweight	13	-0.16 [-0.39, 0.07]	85	0.18	0.09
Obesity	53	-0.37 [-0.46, -0.28]	78	＜0.00001**	
Sex					
Boys	19	-0.52 [-0.67, -0.37]	63	＜0.00001**	0.002**
Girls	13	-0.19 [-0.33, -0.05]	76	0.008**	
Metabolic status					

**Health outcomes**	**k**	**MD[95%CI]**	***I*²(%)**	**P-value of Q test**	**Subgroup Differences**
MHO	10	-0.10 [-0.21, 0.02]	0	0.10	0.43
MUO	15	-0.18 [-0.35, -0.01]	80	0.04*	
**FPG**					
Type of exercise					
Aerobic	49	-0.21 [-0.31, -0.11]	89	＜0.0001**	0.46
Anaerobic	14	-0.19 [-0.33, -0.05]	56	0.006**	
Resistance	8	-0.06 [-0.23, 0.10]	60	0.46	
Combined aerobic and resistance	23	-0.21 [-0.34, -0.08]	82	0.001**	
Exercise intensity					
Moderate-to-low	60	-0.24 [-0.33, -0.15]	88	＜0.00001**	0.10
High	34	-0.13 [-0.22, -0.05]	73	0.003**	
Duration per session					
<50 min	32	-0.12 [-0.20, -0.05]	72	0.002**	0.06
⩾50 min	62	-0.24 [-0.34, -0.15]	88	＜0.00001**	
Frequency					
<3 sessions/week	23	-0.06 [-0.13, 0.01]	53	0.11	0.0007**
⩾3 sessions/week	71	-0.25 [-0.33, -0.16]	87	＜0.00001**	
Weekly duration					
<150 min/week	38	-0.08 [-0.15, -0.02]	60	0.009**	0.001**
⩾150 min/week	56	-0.28 [-0.38, -0.18]	89	＜0.00001**	
Intervention duration					
<12 weeks	24	-0.20 [-0.40, 0.01]	90	0.06	0.98
⩾12 weeks	70	-0.20 [-0.27, -0.13]	83	＜0.00001**	
Region					
Asia	39	-0.35 [-0.48, -0.21]	89	＜0.00001**	＜0.00001**
Europe	8	-0.02 [-0.10, 0.07]	41	0.72	
Africa	10	-0.19 [-0.35, -0.04]	51	0.01*	
North America	24	-0.17 [-0.28, -0.06]	84	0.003**	
South America	9	-0.07 [-0.21, 0.07]	61	0.33	
Oceania	4	0.16 [0.02, 0.31]	0	0.02*	
Economic status					
Lower-middle income	10	-0.19 [-0.35, -0.04]	51	0.01*	0.02*

Health outcomes	k	MD[95%CI]	I^2(%)	P-value of Q test	Subgroup Differences
Upper-middle income	39	-0.32 [-0.45, -0.19]	90	＜0.00001**	
High income	45	-0.10 [-0.17, -0.03]	78	0.008**	
Weight status					
Overweight	16	-0.13 [-0.25, -0.01]	78	0.04*	0.04*
Obesity	59	-0.29 [-0.39, -0.19]	88	＜0.00001**	
Sex					
Boys	19	-0.22 [-0.39, -0.05]	76	0.010*	0.45
Girls	26	-0.30 [-0.40, -0.20]	70	＜0.00001**	
**HbA1c**					
Type of exercise					
Aerobic	8	-0.03 [-0.09, 0.02]	0	0.20	0.94
Anaerobic	2	-0.06 [-0.20, 0.09]	0	0.42	
Resistance	0	-	-	-	
Combined aerobic and resistance	4	-0.05 [-0.24, 0.14]	62	0.59	
Exercise intensity					
Moderate-to-low	8	-0.07 [-0.13, 0.00]	0	0.05	0.59
High	6	-0.04 [-0.13, 0.06]	43	0.45	
Duration per session					
<50 min	8	-0.05 [-0.10, 0.01]	0	0.08	0.80
⩾50 min	6	-0.03 [-0.16, 0.11]	50	0.69	
Frequency					
<3 sessions/week	8	-0.04 [-0.09, 0.01]	0	0.15	0.92
⩾3 sessions/week	6	-0.05 [-0.20, 0.10]	60	0.55	
Weekly duration					
<150 min/week	10	-0.05 [-0.10, -0.00]	0	0.05	0.61
⩾150 min/week	4	0.01 [-0.22, 0.25]	68	0.91	
Intervention duration					
<12 weeks	1	-0.10 [-0.34, 0.14]	-	0.41	0.67
⩾12 weeks	13	-0.05 [-0.10, 0.01]	20	0.09	
Region					
Asia	1	-0.10 [-0.29, 0.09]	-	0.29	0.56
Europe	4	0.00 [-0.06, 0.06]	0	0.97	

Health outcomes	k	MD[95%CI]	I^2(%)	P-value of Q test	Subgroup Differences
Africa	0	-	-	-	
North America	1	-0.10 [-0.31, 0.11]	-	0.34	
South America	3	-0.07 [-0.17, 0.02]	0	0.14	
Oceania	5	-0.08 [-0.25, 0.09]	58	0.37	
Economic status					
Lower-middle income	0	-	-	-	0.85
Upper-middle income	4	-0.06 [-0.14, 0.02]	0	0.14	
High income	10	-0.05 [-0.12, 0.03]	37	0.23	
Weight status					
Overweight	3	-0.08 [-0.18, 0.02]	0	0.12	0.71
Obesity	7	-0.05 [-0.17, 0.06]	53	0.38	
Sex					
Boys	0	-	-	-	-
Girls	2	-0.05 [-0.16, 0.06]	0	0.33	
**FINS**					
Type of exercise					
Aerobic	28	-4.66 [-5.99, -3.33]	81	＜0.00001**	0.26
Anaerobic	10	-3.44 [-4.06, -2.83]	0	＜0.00001**	
Resistance	6	-0.28 [-6.03, 5.47]	36	0.92	
Combined aerobic and resistance	13	-4.03 [-6.77, -1.28]	84	0.004**	
Exercise intensity					
Moderate-to-low	32	-4.92 [-6.42, -3.42]	83	＜0.00001**	0.02*
High	25	-2.88 [-3.64, -2.12]	34	＜0.00001**	
Duration per session					
<50 min	19	-2.80 [-3.53, -2.07]	52	＜0.00001**	0.02*
⩾50 min	38	-4.94 [-6.54, -3.35]	78	＜0.00001**	
Frequency					
<3 sessions/week	14	-4.11 [-6.87, -1.35]	75	0.004**	0.89
⩾3 sessions/week	43	-3.89 [-4.82, -2.96]	77	＜0.00001**	
Weekly duration					
<150 min/week	24	-3.42 [-4.57, -2.26]	68	＜0.00001**	0.32
⩾150 min/week	33	-4.31 [-5.66, -2.96]	80	＜0.00001**	

Health outcomes	k	MD[95%CI]	I^2(%)	P-value of Q test	Subgroup Differences
Intervention duration					
<12 weeks	10	-4.73 [-6.89, -2.57]	47	＜0.0001**	0.44
⩾12 weeks	47	-3.79 [-4.73, -2.86]	77	＜0.00001**	
Region					
Asia	15	-5.66 [-7.38, -3.94]	84	＜0.00001**	0.03*
Europe	5	-1.11 [-4.05, 1.84]	54	0.46	
Africa	8	-2.81 [-3.57, -2.06]	35	＜0.00001**	
North America	20	-4.31 [-7.22, -1.40]	72	0.004**	
South America	7	-3.39 [-7.99, 1.20]	84	0.15	
Oceania	2	-0.81 [-6.41, 4.79]	0	0.78	
Economic status					
Lower-middle income	8	-2.81 [-3.57, -2.06]	35	＜0.00001**	0.04*
Upper-middle income	17	-5.53 [-7.48, -3.59]	87	＜0.00001**	
High income	32	-3.41 [-5.07, -1.75]	67	＜0.0001**	
Weight status					
Overweight	9	-1.68 [-3.16, -0.20]	29	0.03*	0.0007**
Obesity	38	-4.83 [-5.91, -3.76]	76	＜0.00001**	
Sex					
Boys	11	-6.64 [-9.16, -4.11]	72	＜0.00001**	0.07
Girls	21	-4.11 [-5.26, -2.96]	74	＜0.00001**	
Metabolic status					
MHO	8	-0.67 [-1.95, 0.60]	0	0.30	0.004**
MUO	13	-5.48 [-8.51, -2.45]	90	0.0004**	
**Depression**					
Type of exercise					
Aerobic	18	-0.03 [-0.16, 0.10]	13	0.66	0.55
Anaerobic	0	-	-	-	
Resistance	1	-0.28 [-0.73, 0.17]	-	0.22	
Combined aerobic and resistance	3	-0.09 [-0.38, 0.19]	0	0.52	
Exercise intensity					
Moderate-to-low	15	0.01 [-0.13, 0.16]	11	0.86	0.16
High	7	-0.15 [-0.31, 0.02]	0	0.08	

Health outcomes	k	MD[95%CI]	I^2(%)	P-value of Q test	Subgroup Differences
Duration per session					
<50 min	11	-0.16 [-0.31, -0.01]	0	0.04*	0.04*
⩾50 min	11	0.06 [-0.09, 0.21]	3	0.43	
Frequency					
<3 sessions/week	5	0.14 [-0.08, 0.36]	0	0.20	0.05
⩾3 sessions/week	17	-0.10 [-0.22, 0.02]	1	0.09	
Weekly duration					
<150 min/week	12	0.01 [-0.14, 0.15]	0	0.94	0.23
⩾150 min/week	10	-0.14 [-0.33, 0.05]	30	0.14	
Intervention duration					
<12 weeks	4	-0.12 [-0.43, 0.19]	0	0.46	0.67
⩾12 weeks	18	-0.05 [-0.17, 0.08]	19	0.48	
Region					
Asia	3	-0.01 [-0.36, 0.34]	39	0.96	0.14
Europe	5	-0.05 [-0.29, 0.19]	0	0.68	
Africa	0	-	-	-	
North America	12	-0.14 [-0.29, -0.00]	0	0.05	
South America	1	0.30 [-0.16, 0.76]	-	0.20	
Oceania	1	0.43 [-0.11, 0.96]	-	0.12	
Economic status					
Lower-middle income	0	-	-	-	0.29
Upper-middle income	4	0.06 [-0.20, 0.32]	33	0.64	
High income	18	-0.09 [-0.21, 0.03]	0	0.14	
Weight status					
Overweight	5	-0.23 [-0.58, 0.11]	32	0.18	0.30
Obesity	11	-0.04 [-0.18, 0.10]	0	0.61	
Sex					
Boys	0	-	-	-	-
Girls	1	-0.55 [-1.28, 0.18]	-	0.14	
**Anxiety**					
Type of exercise					
Aerobic	4	-0.03 [-0.23, 0.18]	0	0.80	0.41

Health outcomes	k	MD[95%CI]	I^2 (%)	P-value of Q test	Subgroup Differences
Anaerobic	0	-	-	-	
Resistance	0	-	-	-	
Combined aerobic and resistance	2	-1.68 [-5.62, 2.26]	97	0.40	
Exercise intensity					
Moderate-to-low	5	-0.50 [-1.17, 0.16]	88	0.14	0.05
High	1	0.29 [-0.14, 0.72]	-	0.19	
Duration per session					
<50 min	0	-	-	-	-
⩾50 min	6	-0.33 [-0.87, 0.21]	86	0.23	
Frequency					
<3 sessions/week	2	-0.07 [-0.38, 0.24]	0	0.65	0.27
⩾3 sessions/week	4	-0.62 [-1.56, 0.31]	92	0.19	
Weekly duration					
<150 min/week	2	-0.07 [-0.38, 0.24]	0	0.65	0.27
⩾150 min/week	4	-0.62 [-1.56, 0.31]	92	0.19	
Intervention duration					
<12 weeks	1	0.05 [-0.62, 0.73]	-	0.87	0.31
⩾12 weeks	5	-0.43 [-1.06, 0.21]	89	0.19	
Region					
Asia	2	-1.82 [-5.47, 1.84]	97	0.33	0.42
Europe	1	0.29 [-0.14, 0.72]	-	0.19	
Africa	0	-	-	-	
North America	2	-0.08 [-0.41, 0.25]	0	0.63	
South America	0	-	-	-	
Oceania	1	0.03 [-0.50, 0.56]	-	0.92	
Economic status					
Lower-middle income	0	-	-	-	0.05
Upper-middle income	3	-1.02 [-2.15, 0.10]	94	0.08	
High income	3	0.16 [-0.14, 0.46]	0	0.30	
Weight status					
Overweight	0	-	-	-	-
Obesity	3	-0.04 [-0.26, 0.19]	0	0.75	

Health outcomes	k	MD[95%CI]	I^2(%)	P-value of Q test	Subgroup Differences
Sex					
Boys	0	-	-	-	-
Girls	1	-3.73 [-4.97, -2.49]	-	＜0.00001**	
**Self-esteem**					
Type of exercise					
Aerobic	8	0.18 [-0.04, 0.41]	24	0.11	0.48
Anaerobic	0	-	-	-	
Resistance	2	0.51 [-0.09, 1.12]	61	0.09	
Combined aerobic and resistance	3	0.10 [-0.20, 0.40]	0	0.53	
Exercise intensity					
Moderate-to-low	9	0.30 [0.03, 0.58]	44	0.03*	0.38
High	4	0.14 [-0.09, 0.37]	0	0.23	
Duration per session					
<50 min	7	0.21 [-0.01, 0.43]	18	0.06	0.98
⩾50 min	6	0.21 [-0.08, 0.49]	33	0.16	
Frequency					
<3 sessions/week	2	0.59 [-0.19, 1.36]	65	0.14	0.28
⩾3 sessions/week	11	0.15 [-0.00, 0.30]	0	0.05	
Weekly duration					
<150 min/week	5	0.26 [0.01, 0.51]	16	0.04*	0.65
⩾150 min/week	8	0.18 [-0.05, 0.42]	27	0.13	
Intervention duration					
<12 weeks	2	0.68 [0.02, 1.34]	37	0.04*	0.12
⩾12 weeks	11	0.15 [-0.00, 0.29]	0	0.05	
Region					
Asia	1	1.04 [0.27, 1.81]	-	0.008**	0.06
Europe	2	0.01 [-0.31, 0.33]	0	0.94	
Africa	0	-	-	-	
North America	8	0.13 [-0.05, 0.30]	0	0.16	
South America	0	-	-	-	
Oceania	2	0.53 [-0.07, 1.13]	57	0.08	
Economic status					

Health outcomes	k	MD[95%CI]	I^2(%)	P-value of Q test	Subgroup Differences
Lower-middle income	0	-	-	-	-
Upper-middle income	0	-	-	-	
High income	13	0.21 [0.04, 0.37]	19	0.01*	
Weight status					
Overweight	2	0.02 [-0.25, 0.28]	0	0.91	0.16
Obesity	5	0.28 [0.01, 0.55]	17	0.04*	
Sex					
Boys	1	0.85 [0.26, 1.44]	-	0.005**	0.01*
Girls	1	0.04 [-0.23, 0.31]	-	0.77	
**Self-worth**					
Type of exercise					
Aerobic	9	0.27 [0.08, 0.45]	11	0.005**	0.85
Anaerobic	0	-	-	-	
Resistance	2	0.27 [-0.09, 0.63]	0	0.14	
Combined aerobic and resistance	1	0.12 [-0.35, 0.59]	-	0.61	
Exercise intensity					
Moderate-to-low	6	0.34 [0.09, 0.60]	0	0.007**	0.36
High	6	0.20 [0.00, 0.39]	9	0.04*	
Duration per session					
<50 min	11	0.24 [0.09, 0.39]	0	0.002**	0.71
⩾50 min	1	0.35 [-0.22, 0.92]	-	0.23	
Frequency					
<3 sessions/week	0	-	-	-	-
⩾3 sessions/week	12	0.25 [0.10, 0.39]	0	0.001**	
Weekly duration					
<150 min/week	7	0.21 [0.01, 0.42]	0	0.04*	0.55
⩾150 min/week	5	0.31 [0.05, 0.58]	28	0.02*	
Intervention duration					
<12 weeks	2	0.62 [0.13, 1.11]	0	0.01*	0.12
⩾12 weeks	10	0.21 [0.05, 0.36]	0	0.008**	
Region					
Asia	0	-	-	-	0.18

Health outcomes	k	MD[95%CI]	I^2(%)	P-value of Q test	Subgroup Differences
Europe	3	0.56 [0.18, 0.95]	0	0.004**	
Africa	0	-	-	-	
North America	8	0.18 [0.01, 0.34]	0	0.04*	
South America	0	-	-	-	
Oceania	1	0.35 [-0.22, 0.92]	-	0.23	
Economic status					
Lower-middle income	0	-	-	-	-
Upper-middle income	0	-	-	-	
High income	12	0.25 [0.10, 0.39]	0	0.001**	
Weight status					
Overweight	4	0.24 [-0.15, 0.64]	45	0.23	0.87
Obesity	6	0.28 [0.06, 0.50]	0	0.01*	
Sex					
Boys	1	0.35 [-0.22, 0.92]	-	0.23	-
Girls	0	-	-	-	

Notes: MD, mean difference; CI, confidence interval; I^2, I-square; P-value, probability value. N indicated the number of the included studies while k indicated the number of intervention groups incorporated into the meta-analysis. BMI, body mass index; WC, waist circumference; BF%, percentage of body fat; VO_2max, maximal oxygen consumption; VO_2peak, peak oxygen uptake; HRmax, max heart rate; HRrest, resting heart rate; SBP, systolic blood pressure; DBP, diastolic blood pressure; TC, total cholesterol; TG, triglyceride; HDL-C, high-density lipoprotein cholesterol; LDL-C, low-density lipoprotein cholesterol; FPG, fasting plasma glucose; HbA1c, hemoglobin A1c; FINS, fasting insulin; MHO, metabolically healthy obesity; MUO, metabolically unhealthy obesity.

$*P < 0.05$, $**P < 0.01$

Figure S1. Forest plots of meta-analysis results for 20 outcome indicators in children and adolescents with overweight or obesity

Figure S1.1. Forest plot of the effect of exercise on BMI

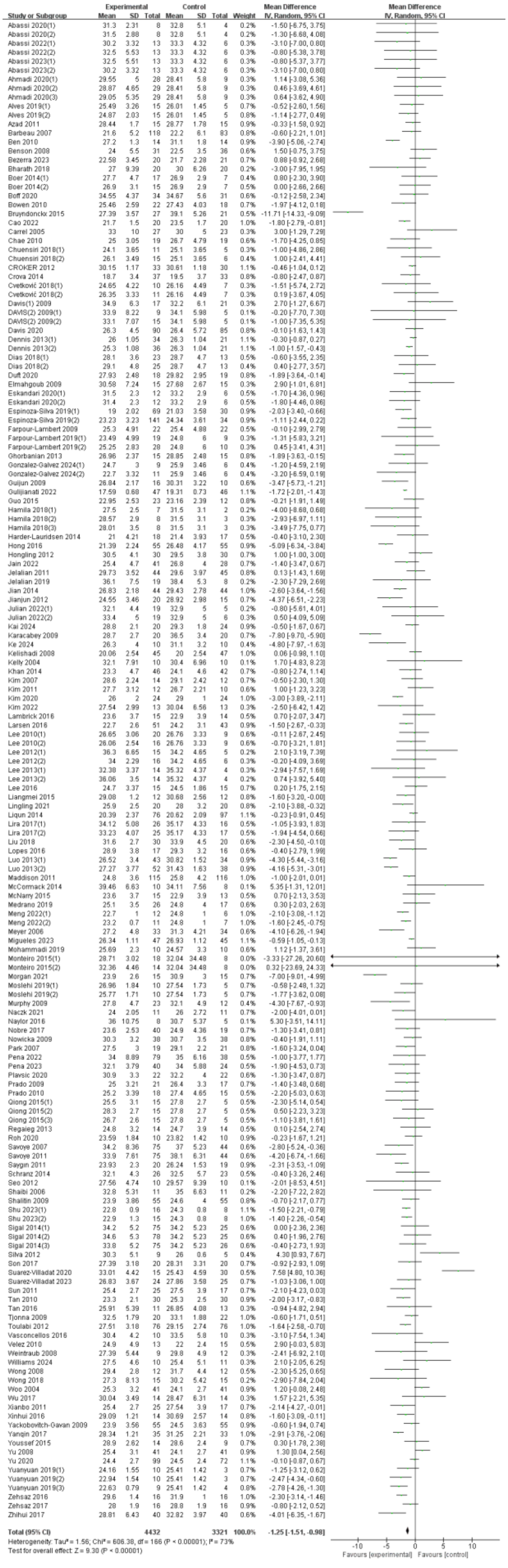

Figure S1.2. Forest plot of the effect of exercise on WC

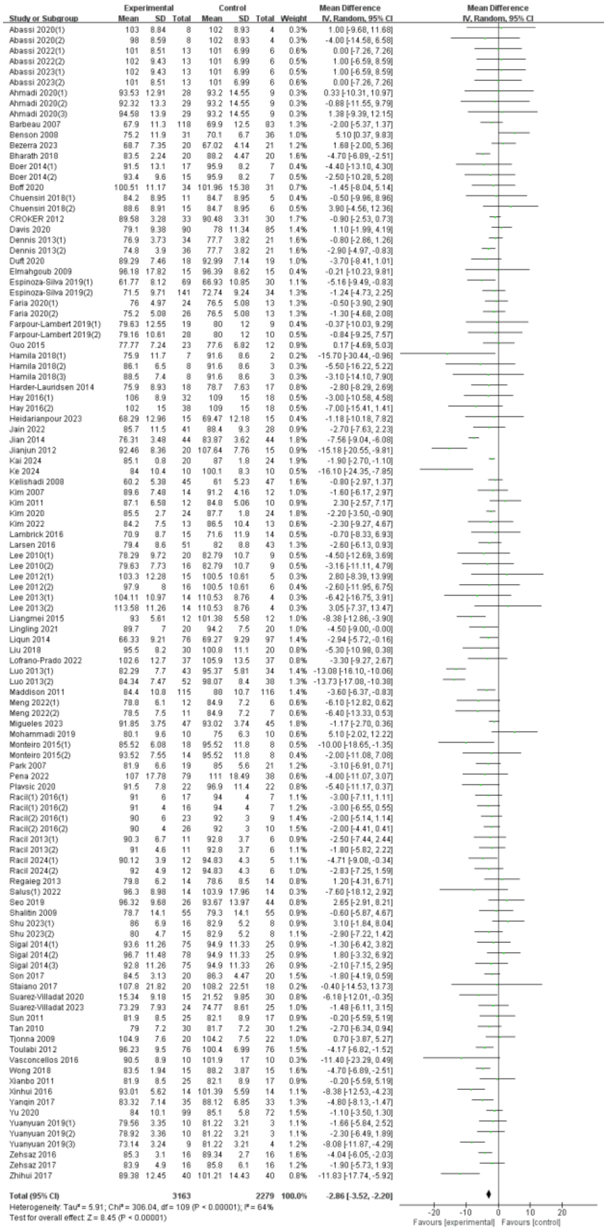

Figure S1.3. Forest plot of the effect of exercise on BF%

Study or Subgroup	Experimental Mean	Experimental SD	Experimental Total	Control Mean	Control SD	Control Total	Weight	Mean Difference IV, Random, 95% CI
Abassi 2020(1)	33.3	2.41	8	33.8	3.9	4	0.6%	-0.50 [-4.67, 3.67]
Abassi 2020(2)	31.7	2.41	8	33.8	3.9	4	0.6%	-2.10 [-6.27, 2.07]
Abassi 2022(1)	32.1	3.42	13	33.5	3.22	6	0.7%	-1.40 [-4.58, 1.78]
Abassi 2022(2)	32.9	2.99	13	33.5	3.22	6	0.8%	-0.60 [-3.65, 2.45]
Abassi 2023(1)	32.9	2.93	13	33.5	3.2	6	0.8%	-0.60 [-3.62, 2.42]
Abassi 2023(2)	32.1	3.42	13	33.5	3.2	6	0.7%	-1.40 [-4.56, 1.76]
Aguilar-Cordero 2020	38.5	6.62	49	41.25	4.93	49	0.9%	-2.75 [-5.06, -0.44]
Alves 2019(1)	26.24	4.97	15	26.02	5.06	5	0.4%	0.22 [-4.88, 5.32]
Alves 2019(2)	24.51	6.08	15	26.02	5.06	5	0.4%	-1.51 [-6.91, 3.89]
Barbeau 2007	29.1	11.8	118	31	12.2	83	0.7%	-1.90 [-5.28, 1.48]
Ben 2010	35.3	5.4	14	39.8	5.3	14	0.6%	-4.50 [-8.46, -0.54]
Bharath 2018	39.6	9.39	20	43	9.84	20	0.4%	-3.40 [-9.36, 2.56]
Boer 2014(1)	30.4	7	17	32	7	7	0.3%	-1.60 [-7.76, 4.56]
Boer 2014(2)	31.3	6.6	15	32	7	7	0.3%	-0.70 [-6.87, 5.47]
Bowen 2010	31.29	5.78	22	38.19	6.34	18	0.6%	-6.90 [-10.70, -3.10]
Bruyndonckx 2015	37.57	9.39	27	51.64	3.98	21	0.6%	-14.07 [-18.00, -10.14]
Cao 2022	40.1	7.8	20	44	7.5	20	0.5%	-3.90 [-8.64, 0.84]
Carrel 2005	32.6	6.4	27	34.5	5.8	23	0.7%	-1.90 [-5.28, 1.48]
Chae 2010	32.7	5.67	19	37.8	4.36	19	0.7%	-5.10 [-8.32, -1.88]
Chuensiri 2018(1)	21.5	5.31	11	24.4	5.31	5	0.4%	-2.90 [-8.51, 2.71]
Chuensiri 2018(2)	24.8	5.42	15	24.4	5.31	6	0.5%	0.40 [-4.66, 5.46]
Cvetković 2018(1)	33.47	6.99	10	29.6	7.68	7	0.3%	3.87 [-3.28, 11.02]
Cvetković 2018(2)	31.15	6.9	11	29.6	7.68	7	0.3%	1.55 [-5.45, 8.55]
Davis 2020	36.2	7.41	90	36.2	7.19	85	1.0%	0.00 [-2.16, 2.16]
Dennis 2013(1)	37.8	2.45	34	38.8	2.53	21	1.1%	-1.00 [-2.36, 0.36]
Dennis 2013(2)	36.5	2.58	36	38.8	2.53	21	1.1%	-2.30 [-3.67, -0.93]
Dias 2018(1)	43.4	7.1	18	42.9	6.5	8	0.4%	0.50 [-5.07, 6.07]
Dias 2018(2)	42.6	6	22	42.9	6.5	8	0.4%	-0.30 [-5.45, 4.85]
Duft 2020	37.78	5.55	18	42.99	5.78	19	0.7%	-5.21 [-8.86, -1.56]
Espinoza-Silva 2019(1)	19.55	4.81	69	22.78	6.15	30	0.9%	-3.23 [-5.71, -0.75]
Espinoza-Silva 2019(2)	26.81	6.8	141	28.17	6.45	34	0.9%	-1.36 [-3.80, 1.08]
Faria 2020(1)	26.1	5.57	24	27.3	5.82	13	0.6%	-1.20 [-5.07, 2.67]
Faria 2020(2)	26.5	5.82	26	27.3	5.82	13	0.6%	-0.80 [-4.67, 3.07]
Farpour-Lambert 2009	41.1	6.97	22	43.2	7.71	22	0.5%	-2.10 [-6.44, 2.24]
Farpour-Lambert 2019(1)	40.22	9.6	19	44	5.2	9	0.4%	-3.78 [-9.27, 1.71]
Farpour-Lambert 2019(2)	42.9	2.72	28	44	5.2	10	0.7%	-1.10 [-4.48, 2.28]
Ghorbanian 2013	27.43	1.3	15	29.11	2.13	15	1.2%	-1.68 [-2.94, -0.42]
Gonzalez-Galvez 2024(1)	26.7	7.8	9	31	9.01	6	0.2%	-4.30 [-13.13, 4.53]
Gonzalez-Galvez 2024(2)	24.3	7.96	11	31	9.01	6	0.2%	-6.70 [-15.31, 1.91]
Gulijianati 2022	38.03	3.16	47	41.98	3.87	46	1.1%	-3.95 [-5.39, -2.51]
Haiying 2023	35.8	5.16	20	39.8	6.15	20	0.7%	-4.00 [-7.52, -0.48]
Hamila 2018(1)	21.3	4.8	7	27.4	6.7	2	0.2%	-6.10 [-16.04, 3.84]
Hamila 2018(2)	23.4	3.6	8	27.4	6.7	3	0.2%	-4.00 [-11.98, 3.98]
Hamila 2018(3)	27.1	7	8	27.4	6.7	3	0.2%	-0.30 [-9.30, 8.70]
Harder-Lauridsen 2014	34.9	6.98	18	37.9	7.78	18	0.5%	-3.00 [-7.83, 1.83]
Hay 2016(1)	37.9	5.2	32	39.4	4.8	17	0.8%	-1.50 [-4.41, 1.41]
Hay 2016(2)	39.5	4.3	37	39.4	4.8	17	0.9%	0.10 [-2.57, 2.77]
Hongling 2012	27.1	4.6	30	31.3	8	30	0.7%	-4.20 [-7.50, -0.90]
Jianjun 2012	27.24	6.08	20	40.56	6.66	15	0.6%	-13.32 [-17.62, -9.02]
Julian 2022(1)	33.8	3.6	19	37.7	3.6	5	0.7%	-3.90 [-7.45, -0.35]
Julian 2022(2)	35.9	3.8	19	37.7	3.6	6	0.7%	-1.80 [-5.15, 1.55]
Jun 2006(1)	34.08	3.81	33	36.84	4.6	31	1.0%	-2.76 [-4.84, -0.68]
Jun 2006(2)	35.12	3.5	35	35.47	4.79	32	1.0%	-0.35 [-2.37, 1.67]
Kai 2024	30.52	1.01	20	34.16	0.72	24	1.3%	-3.64 [-4.17, -3.11]
Ke 2024	28.9	5.5	10	35.1	5.4	10	0.5%	-6.20 [-10.98, -1.42]
Kelly 2004	45.4	6.64	10	45.3	7.59	10	0.3%	0.10 [-6.15, 6.35]
Khan 2014	37	5.5	46	38.3	6.3	42	0.9%	-1.30 [-3.78, 1.18]
Kim 2007	29.3	3.74	14	29.3	6.58	12	0.6%	0.00 [-4.21, 4.21]
Kim 2011	34.6	5.2	12	34.1	3.48	10	0.7%	0.50 [-3.15, 4.15]
Kim 2020	30.8	2.6	24	34.9	2.5	24	1.1%	-4.10 [-5.54, -2.66]
Kim 2022	33.3	2.9	13	36.3	3.7	13	0.9%	-3.00 [-5.56, -0.44]
Lambrick 2016	32.7	7	15	29.5	8.4	14	0.4%	3.20 [-2.45, 8.85]
Larsen 2016	32.9	7.4	50	38.1	7	44	0.8%	-5.20 [-8.11, -2.29]
Lee 2013(1)	46.14	3.37	14	51.38	3.48	4	0.6%	-5.24 [-9.08, -1.40]
Lee 2013(2)	49.89	4.1	14	51.38	3.48	4	0.6%	-1.49 [-5.52, 2.54]
Lingling 2021	20.1	4.7	20	21.6	3.5	20	0.9%	-1.50 [-4.07, 1.07]
Lofrano-Prado 2022	45.6	10.8	37	49.4	11.7	37	0.4%	-3.80 [-8.93, 1.33]
Lopes 2016	43.5	4.6	17	46	6.3	16	0.6%	-2.50 [-6.28, 1.28]
Luo 2013(1)	32.24	5.62	43	38.08	3.97	34	1.0%	-5.84 [-7.99, -3.69]
Luo 2013(2)	29.46	5.9	52	35.92	5.24	38	0.9%	-6.46 [-8.77, -4.15]
Maddison 2011	29.8	7.2	115	31.1	6.3	116	1.1%	-1.30 [-3.05, 0.45]
McCormack 2014	43	3.93	10	41	4.01	8	0.7%	2.00 [-1.70, 5.70]
Medrano 2019	39.2	4.3	26	37.7	5.5	17	0.8%	1.50 [-1.59, 4.59]
Meng 2022(1)	36.2	3.9	12	39.5	2.1	6	0.8%	-3.30 [-6.07, -0.53]
Meng 2022(2)	34.4	1.5	11	39.5	2.1	7	1.0%	-5.10 [-6.89, -3.31]
Meyer 2006	34.9	9.71	33	37.2	8.8	34	0.5%	-2.30 [-6.74, 2.14]
Mohammadi 2019	23.06	1.4	10	23.75	1	10	1.2%	-0.69 [-1.76, 0.38]
Monteiro 2015(1)	43.12	3.74	18	47.98	3.94	8	0.7%	-4.86 [-8.09, -1.63]
Monteiro 2015(2)	45.21	4.53	14	47.98	3.94	8	0.7%	-2.77 [-6.39, 0.85]
Moon 2004	32.9	5.8	34	36	6.2	28	0.8%	-3.10 [-6.11, -0.09]
Moslehi 2019(1)	31.52	3.08	10	31.76	2.49	5	0.8%	-0.24 [-3.14, 2.66]
Moslehi 2019(2)	28.51	2.03	10	31.76	2.49	5	0.9%	-3.25 [-5.77, -0.73]
Naczk 2021	23.7	4.19	11	26.8	3.47	11	0.7%	-3.10 [-6.31, 0.11]
Naylor 2016	43.2	8.2	8	44	4.92	5	0.3%	-0.80 [-7.93, 6.33]
Nobre 2017	35.9	6.32	40	38.5	10.9	19	0.4%	-2.60 [-7.88, 2.68]
Nowicka 2009	46.6	5	19	47.2	4.5	13	0.7%	-0.60 [-3.92, 2.72]
Park 2007	41.8	4.6	19	44.9	3.6	21	0.9%	-3.10 [-5.68, -0.52]
Pena 2022	44	8.89	79	47	6.16	38	0.8%	-3.00 [-5.77, -0.23]
Pena 2023	42.7	4.43	40	46.3	4.9	24	0.9%	-3.60 [-5.99, -1.21]
Plavsic 2020	41.8	6.1	22	44	3.9	22	0.8%	-2.20 [-5.23, 0.83]
Prado 2009	30.2	5.5	21	32.5	3.3	17	0.8%	-2.30 [-5.13, 0.53]
Prado 2010	30.4	5.52	18	32.9	4.26	15	0.7%	-2.50 [-5.84, 0.84]
Qiong 2015(1)	23.1	4.3	15	26.9	5	5	0.5%	-3.80 [-8.69, 1.09]
Qiong 2015(2)	23.7	5.6	15	26.9	5	5	0.4%	-3.20 [-8.42, 2.02]
Qiong 2015(3)	22.4	3.7	15	26.9	5	5	0.5%	-4.50 [-9.27, 0.27]
Racil(1) 2016(1)	36.4	1.5	17	39.3	0.7	7	1.2%	-2.90 [-3.78, -2.02]
Racil(1) 2016(2)	37.2	1.2	16	39.3	0.7	7	1.2%	-2.10 [-2.88, -1.32]
Racil(2) 2016(1)	36.5	1.3	23	37	1.9	9	1.1%	-0.50 [-1.85, 0.85]
Racil(2) 2016(2)	38.8	3.7	26	37	1.9	10	1.0%	1.80 [-0.05, 3.65]
Racil 2013(1)	34.3	1.7	11	35.4	1.2	6	1.1%	-1.10 [-2.49, 0.29]
Racil 2013(2)	36.2	1	11	35.4	1.2	6	1.2%	0.80 [-0.33, 1.93]
Racil 2024(1)	35.41	1.6	12	37.6	2.3	5	1.0%	-2.19 [-4.40, 0.02]
Racil 2024(2)	35.93	2.5	12	37.6	2.3	6	0.9%	-1.67 [-3.99, 0.65]
Regaieg 2013	28.8	6.9	14	29.5	8.4	14	0.4%	-0.70 [-6.39, 4.99]
Salus(1) 2022	39.2	5.61	14	38.9	7.11	14	0.5%	0.30 [-4.44, 5.04]
Savoye 2007	43.6	8.2	75	48.2	7.16	44	0.8%	-4.60 [-7.41, -1.79]
Savoye 2011	43.1	7.59	75	47.9	5.56	44	0.9%	-4.80 [-7.18, -2.42]
Schranz 2014	41	6	26	39.5	7.6	23	0.6%	1.50 [-2.37, 5.37]
Seo 2012	35.66	19.13	10	36.15	20.71	10	0.1%	-0.49 [-17.96, 16.98]
Seo 2019	40.22	4.62	26	41.05	4.49	44	1.0%	-0.83 [-3.05, 1.39]
Shaibi 2006	32.8	6.96	11	30.7	4.64	11	0.5%	2.10 [-2.84, 7.04]
Shalitin 2009	37.6	10.98	55	38.3	11.79	55	0.6%	-0.70 [-4.96, 3.56]
Shu 2023(1)	34.2	5.1	16	37.9	4.9	8	0.6%	-3.70 [-7.92, 0.52]
Shu 2023(2)	33.9	5.7	15	37.9	4.9	8	0.5%	-4.00 [-8.46, 0.46]
Sigal 2014(1)	48	6.02	74	48.3	6.02	24	0.8%	-0.30 [-3.07, 2.47]
Sigal 2014(2)	48.1	6.06	75	48.3	6.02	25	0.8%	-0.20 [-2.93, 2.53]
Sigal 2014(3)	48.8	6.02	74	48.3	6.02	25	0.8%	0.50 [-2.23, 3.23]
Silva 2012	27.6	5.6	9	20.7	5.9	5	0.3%	6.90 [0.57, 13.23]
Son 2017	30.4	7.2	20	33.85	10.78	20	0.4%	-3.45 [-9.13, 2.23]
Staiano 2017	48.8	8.41	20	49	6.38	18	0.5%	-0.20 [-4.92, 4.52]
Staiano 2018	41.5	5.98	22	43.8	3.12	23	0.8%	-2.30 [-5.11, 0.51]
Suarez-Villadat 2020	23.14	9.28	15	29.19	9.52	30	0.4%	-6.05 [-11.85, -0.25]
Suarez-Villadat 2023	31.82	11.95	24	33.59	9.51	25	0.4%	-1.77 [-7.83, 4.29]
Sun 2011	30	4.8	25	35.6	5.1	17	0.8%	-5.60 [-8.67, -2.53]
Tan 2016	38.5	8.4	11	41.6	6.1	13	0.4%	-3.10 [-9.07, 2.87]
Tas 2023	45.19	6.18	31	44.05	6.18	6	0.4%	1.14 [-4.26, 6.54]
Tjonna 2009	39.3	2.24	20	40.8	2.35	22	1.1%	-1.50 [-2.89, -0.11]
Vasconcellos 2016	38.9	6.1	10	41.7	5.1	10	0.5%	-2.80 [-7.73, 2.13]
Velez 2010	18.9	10.7	13	20.5	7.4	15	0.3%	-1.60 [-8.52, 5.32]
Williams 2019	36.2	7.7	90	36.2	8	85	0.9%	0.00 [-2.33, 2.33]
Williams 2024	30.9	10.2	10	28.8	10.9	11	0.2%	2.10 [-6.93, 11.13]
Wong 2008	34.8	7	12	36.3	3.8	12	0.5%	-1.50 [-6.01, 3.01]
Wong 2018	39.6	8.13	15	43.4	8.52	15	0.4%	-3.80 [-9.76, 2.16]
Woo 2004	37.3	4	41	37	3.5	41	1.1%	0.30 [-1.33, 1.93]
Woo 2012	25.55	2.19	10	27.3	1.51	10	1.1%	-1.75 [-3.40, -0.10]
Wu 2017	39.24	7.45	14	37.58	9.49	14	0.3%	1.66 [-4.66, 7.98]
Xianbo 2011	30	4.8	25	35.6	5.1	17	0.8%	-5.60 [-8.67, -2.53]
Yackobovitch-Gavan 2009	37.6	8.16	36	38.6	8.73	40	0.6%	-1.00 [-4.80, 2.80]
Youssef 2015	35.2	6.88	14	38	5.67	9	0.4%	-2.80 [-7.97, 2.37]
Yu 2008	37.3	4.2	41	36.9	3.3	41	1.1%	0.40 [-1.23, 2.03]
Yuanyuan 2019(1)	42	3	10	48	2	3	0.8%	-6.00 [-8.93, -3.07]
Yuanyuan 2019(2)	38	3	10	48	2	3	0.8%	-10.00 [-12.93, -7.07]
Yuanyuan 2019(3)	35	2	9	48	2	4	0.9%	-13.00 [-15.36, -10.64]
Zehsaz 2016	28.4	3.9	16	34.9	3.4	16	0.9%	-6.50 [-9.04, -3.96]
Zehsaz 2017	33	7.4	16	34.1	6.5	16	0.5%	-1.10 [-5.93, 3.73]
Zhihui 2017	30.95	7.05	40	32.97	4.61	40	0.9%	-2.02 [-4.63, 0.59]
**Total (95% CI)**			**3761**			**2707**	**100.0%**	**-2.39 [-2.81, -1.96]**

Heterogeneity: $Tau^2 = 3.65$; $Chi^2 = 485.07$, $df = 145$ ($P < 0.00001$); $I^2 = 70\%$
Test for overall effect: $Z = 11.02$ ($P < 0.00001$)

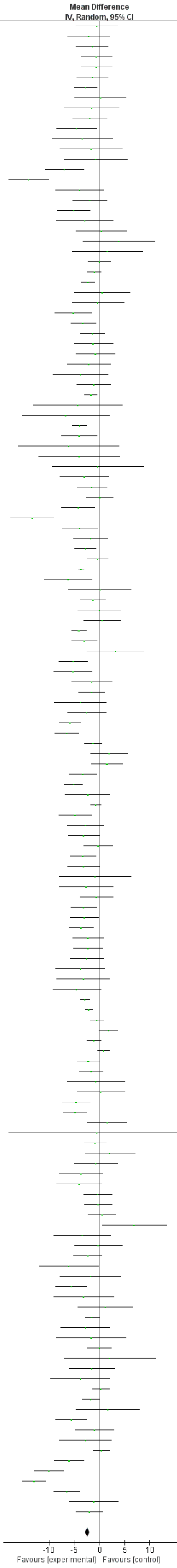

Figure S1.4. Forest plot of the effect of exercise on VO_2max

Study or Subgroup	Experimental Mean	Experimental SD	Experimental Total	Control Mean	Control SD	Control Total	Weight	Mean Difference IV, Random, 95% CI
Alves 2019(1)	40.66	4.03	15	37.94	4.97	5	1.3%	2.72 [-2.09, 7.53]
Alves 2019(2)	39.7	3.08	15	37.94	4.97	5	1.4%	1.76 [-2.87, 6.39]
Bharath 2018	27	5.37	20	26	9.39	20	1.4%	1.00 [-3.74, 5.74]
Cao 2022	43.8	2.4	20	39.6	3.2	20	3.4%	4.20 [2.45, 5.95]
Carrel 2005	34.5	6	27	32.5	6	23	2.1%	2.00 [-1.34, 5.34]
Chae 2010	35.7	4.79	19	32.2	5.67	19	2.1%	3.50 [0.16, 6.84]
Crova 2014	44.3	4	37	44.4	4.3	33	3.2%	-0.10 [-2.05, 1.85]
Farpour-Lambert 2009	36.3	4.06	22	35.3	6.27	22	2.2%	1.00 [-2.12, 4.12]
Ghorbanian 2013	38.6	2.16	15	33.7	2.4	15	3.5%	4.90 [3.27, 6.53]
Gonzalez-Galvez 2024(1)	24.2	3.6	9	20.6	3.81	6	1.8%	3.60 [-0.25, 7.45]
Gonzalez-Galvez 2024(2)	25	3.98	11	20.6	3.81	6	1.8%	4.40 [0.55, 8.25]
Hamila 2018(1)	41.68	2.17	7	36.68	2.06	2	2.1%	5.00 [1.72, 8.28]
Hamila 2018(2)	44.51	2.65	8	36.68	2.06	3	2.4%	7.83 [4.86, 10.80]
Hamila 2018(3)	41.53	2.06	8	36.68	2.06	3	2.5%	4.85 [2.12, 7.58]
Hongling 2012	32.7	4.6	30	27	4.5	30	2.9%	5.70 [3.40, 8.00]
Kai 2024	39	0.44	20	37.14	0.77	24	4.3%	1.86 [1.50, 2.22]
Khan 2014	35.6	6.5	46	34.4	6.1	42	2.6%	1.20 [-1.43, 3.83]
Lambrick 2016	31.6	5.9	15	36.8	6.4	14	1.5%	-5.20 [-9.69, -0.71]
Lee 2010(1)	57.91	1.13	20	58.28	1.52	9	3.9%	-0.37 [-1.48, 0.74]
Lee 2010(2)	58.45	1.8	16	58.28	1.52	9	3.7%	0.17 [-1.16, 1.50]
Lingling 2021	41.3	4	20	37.6	3.5	20	2.9%	3.70 [1.37, 6.03]
Maddison 2011	28.9	5.7	115	27.8	5.3	116	3.7%	1.10 [-0.32, 2.52]
Migueles 2023	40.78	2.09	46	39.84	2.08	43	4.1%	0.94 [0.07, 1.81]
Moslehi 2019(1)	33.13	2.19	10	30.16	2.38	5	2.7%	2.97 [0.48, 5.46]
Moslehi 2019(2)	32.79	1.73	10	30.16	2.38	5	2.8%	2.63 [0.28, 4.98]
Naczk 2021	31.7	4.05	11	25.9	3.08	11	2.3%	5.80 [2.79, 8.81]
Qiong 2015(1)	34.8	3.8	15	30.8	3.9	5	1.7%	4.00 [0.08, 7.92]
Qiong 2015(2)	32.6	4	15	30.8	3.9	5	1.7%	1.80 [-2.17, 5.77]
Qiong 2015(3)	35.5	3.6	15	30.8	3.9	5	1.8%	4.70 [0.83, 8.57]
Racil 2024(1)	38.35	1.7	12	33.1	1.8	5	3.3%	5.25 [3.40, 7.10]
Racil 2024(2)	35.22	1.9	12	33.1	1.8	6	3.3%	2.12 [0.32, 3.92]
Roh 2020	36.46	5.76	10	35.45	5.66	10	1.3%	1.01 [-4.00, 6.02]
Saygın 2011	34.44	3.15	20	27.76	2.03	19	3.5%	6.68 [5.03, 8.33]
Shu 2023(1)	45.4	2.6	16	41.4	2	8	3.3%	4.00 [2.12, 5.88]
Shu 2023(2)	44.2	2.7	15	41.4	2	8	3.2%	2.80 [0.85, 4.75]
Sun 2011	41.3	6.2	25	39.3	4.3	17	2.2%	2.00 [-1.18, 5.18]
Tan 2016	44.3	1.4	11	42.2	3.1	13	3.3%	2.10 [0.22, 3.98]
Tjonna 2009	35.3	3.58	20	32.3	3.75	22	3.0%	3.00 [0.78, 5.22]
**Total (95% CI)**			**778**			**633**	**100.0%**	**2.83 [2.17, 3.50]**

Heterogeneity: $Tau^2 = 2.65$; $Chi^2 = 160.57$, $df = 37$ ($P < 0.00001$); $I^2 = 77\%$
Test for overall effect: $Z = 8.32$ ($P < 0.00001$)

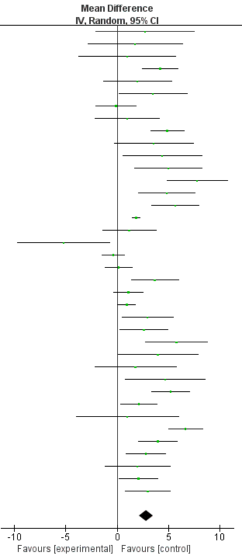

Figure S1.5. Forest plot of the effect of exercise on VO_2peak

Study or Subgroup	Experimental Mean	Experimental SD	Experimental Total	Control Mean	Control SD	Control Total	Weight	Mean Difference IV, Random, 95% CI
Alberga 2016(1)	33.4	6.06	75	30.9	6.1	25	2.6%	2.50 [-0.26, 5.26]
Alberga 2016(2)	30.9	6.18	78	30.9	6.1	25	2.6%	0.00 [-2.76, 2.76]
Alberga 2016(3)	32.2	5.2	75	30.9	6.1	26	2.7%	1.30 [-1.32, 3.92]
Benson 2008	41.4	6.8	29	42.4	7.6	34	2.0%	-1.00 [-4.56, 2.56]
Chuensiri 2018(1)	35.5	4.64	11	26.4	3.98	5	1.6%	9.10 [4.66, 13.54]
Chuensiri 2018(2)	30.8	3.87	15	26.4	3.98	6	1.9%	4.40 [0.66, 8.14]
Davis 2020	31.7	3.58	90	31.3	4.11	85	3.8%	0.40 [-0.74, 1.54]
Dennis 2013(1)	27.9	6.41	34	25.5	6.48	21	2.1%	2.40 [-1.11, 5.91]
Dennis 2013(2)	29.3	7.2	36	25.5	6.48	21	2.0%	3.80 [0.17, 7.43]
Dias 2018(1)	36.6	7.4	22	32.2	5.9	13	1.6%	4.40 [-0.06, 8.86]
Dias 2018(2)	33.6	6.3	25	32.2	5.9	13	1.8%	1.40 [-2.65, 5.45]
Duft 2020	37.66	6.19	18	35.06	5.5	19	1.9%	2.60 [-1.18, 6.38]
Elmahgoub 2009	27	6.15	15	27.31	4.6	15	1.9%	-0.31 [-4.20, 3.58]
Elnaggar 2021	30.87	2.78	13	27.45	1.84	14	3.3%	3.42 [1.63, 5.21]
Faria 2020(1)	38.4	4.5	24	36.3	4.58	13	2.4%	2.10 [-0.97, 5.17]
Faria 2020(2)	39	4.7	26	36.3	4.58	13	2.4%	2.70 [-0.38, 5.78]
Hay 2016(1)	26	5.9	32	23.3	4.8	18	2.4%	2.70 [-0.32, 5.72]
Hay 2016(2)	26.6	4	38	23.3	4.8	18	2.7%	3.30 [0.74, 5.86]
Heidarianpour 2023	40.62	6.48	15	38.38	6.55	15	1.5%	2.24 [-2.42, 6.90]
Kelly 2004	23.2	4.74	10	20.9	6.96	10	1.3%	2.30 [-2.92, 7.52]
Lambrick 2016	49.2	7.9	15	52.1	9	14	1.0%	-2.90 [-9.08, 3.28]
Lee 2012(1)	38	3.64	15	29.9	3.08	5	2.2%	8.10 [4.83, 11.37]
Lee 2012(2)	38	5.99	16	29.9	3.08	6	1.9%	8.10 [4.27, 11.93]
Lee 2013(1)	33.67	7.69	14	23.47	4.34	4	1.1%	10.20 [4.34, 16.06]
Lee 2013(2)	27.4	7.26	14	23.47	4.34	4	1.1%	3.93 [-1.78, 9.64]
Lopes 2016	35.2	2.4	17	32.1	4.5	16	2.8%	3.10 [0.62, 5.58]
Meng 2022(1)	47.9	2.6	12	42.6	2.9	6	2.6%	5.30 [2.55, 8.05]
Meng 2022(2)	45.6	2.1	11	42.6	2.9	7	2.8%	3.00 [0.52, 5.48]
Murphy 2009	29.5	4.5	23	24.3	4.8	12	2.2%	5.20 [1.92, 8.48]
Naylor 2016	26.4	9.9	8	27.4	6.04	5	0.6%	-1.00 [-9.67, 7.67]
Plavsic 2020	23.9	3.7	22	23.3	4.8	22	2.8%	0.60 [-1.93, 3.13]
Prado 2009	31.3	4.58	21	26	4.54	17	2.5%	5.30 [2.39, 8.21]
Prado 2010	30.3	4.24	18	26	4.65	15	2.4%	4.30 [1.24, 7.36]
Racil(2) 2016(1)	39.2	1	23	38.8	1.5	9	3.9%	0.40 [-0.66, 1.46]
Racil(2) 2016(2)	39.4	1.8	26	38.8	1.5	10	3.8%	0.60 [-0.56, 1.76]
Racil 2013(1)	39.7	1.8	11	38.6	1.4	6	3.5%	1.10 [-0.44, 2.64]
Racil 2013(2)	38.9	2.2	11	38.6	1.4	6	3.4%	0.30 [-1.42, 2.02]
Salus(1) 2022	33	4.49	14	28	4.86	14	2.1%	5.00 [1.53, 8.47]
Salus(2) 2022	33	4.49	14	28	4.86	14	2.1%	5.00 [1.53, 8.47]
Tas 2023	40.93	7.23	31	44.41	7.23	6	1.0%	-3.48 [-9.80, 2.84]
Vasconcellos 2016	33.1	9.2	10	24	3.9	10	1.0%	9.10 [2.91, 15.29]
Williams 2019	31.6	5.6	90	31.2	5.8	85	3.4%	0.40 [-1.29, 2.09]
Woo 2012	32.73	2.66	10	32.06	3.18	10	2.7%	0.67 [-1.90, 3.24]
Youssef 2015	28.2	4.12	14	27.6	2.1	9	2.7%	0.60 [-1.96, 3.16]
**Total (95% CI)**			**1141**			**721**	**100.0%**	**2.56 [1.86, 3.27]**

Heterogeneity: $Tau^2 = 3.06$; $Chi^2 = 121.87$, $df = 43$ ($P < 0.00001$); $I^2 = 65\%$
Test for overall effect: $Z = 7.10$ ($P < 0.00001$)

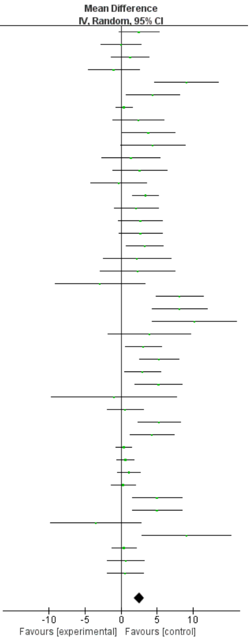

Figure S1.6. Forest plot of the effect of exercise on HRmax

Study or Subgroup	Experimental Mean	Experimental SD	Experimental Total	Control Mean	Control SD	Control Total	Weight	Mean Difference IV, Random, 95% CI
Abassi 2020(1)	198	3.02	8	200	1.2	4	6.4%	-2.00 [-4.40, 0.40]
Abassi 2020(2)	198	1.2	8	200	1.2	4	7.9%	-2.00 [-3.44, -0.56]
Abassi 2022(1)	196	2.6	13	199	1.64	6	7.2%	-3.00 [-4.93, -1.07]
Abassi 2022(2)	198	2.64	13	199	1.64	6	7.1%	-1.00 [-2.94, 0.94]
Cao 2022	198.4	5.3	20	199.2	4.4	20	5.5%	-0.80 [-3.82, 2.22]
Crova 2014	150.5	6.4	37	150.4	16	33	2.6%	0.10 [-5.74, 5.94]
Cvetković 2018(1)	203.9	3.07	10	200.72	3.89	7	4.9%	3.18 [-0.27, 6.63]
Cvetković 2018(2)	198.3	2.9	11	200.72	3.89	7	5.0%	-2.42 [-5.77, 0.93]
Elnaggar 2021	197	9	13	189	9	14	2.1%	8.00 [1.21, 14.79]
Lambrick 2016	203	14	15	202	13	14	1.1%	1.00 [-8.83, 10.83]
Lingling 2021	192	6	20	198	7	20	4.1%	-6.00 [-10.04, -1.96]
Naczk 2021	168	8.25	11	169	5.37	11	2.6%	-1.00 [-6.82, 4.82]
Plavsic 2020	183	9	22	182	25	22	0.9%	1.00 [-10.10, 12.10]
Racil(1) 2016(1)	202.8	2.1	17	202.3	2.2	7	7.2%	0.50 [-1.41, 2.41]
Racil(1) 2016(2)	200.6	1.9	16	202.3	2.2	7	7.2%	-1.70 [-3.58, 0.18]
Racil 2024(1)	195.4	8.4	12	198.31	9.1	5	1.2%	-2.91 [-12.19, 6.37]
Racil 2024(2)	194.2	6.9	12	198.31	9.1	6	1.5%	-4.11 [-12.37, 4.15]
Regaieg 2013	175.3	7.9	14	180.9	8.1	14	2.5%	-5.60 [-11.53, 0.33]
Salus(1) 2022	194.4	9.73	14	191.4	11.6	14	1.6%	3.00 [-4.93, 10.93]
Shu 2023(1)	194.4	3.4	16	194.3	3.9	8	5.2%	0.10 [-3.07, 3.27]
Shu 2023(2)	197.2	2.7	15	194.3	3.9	8	5.4%	2.90 [-0.13, 5.93]
Silva 2012	197.6	1.4	9	198.1	1.3	5	7.9%	-0.50 [-1.96, 0.96]
Wu 2017	180.14	6.56	14	168.64	8.71	14	2.7%	11.50 [5.79, 17.21]
**Total (95% CI)**			**340**			**256**	**100.0%**	**-0.52 [-1.63, 0.60]**

Heterogeneity: Tau² = 3.54; Chi² = 58.53, df = 22 (P < 0.0001); I² = 62%
Test for overall effect: Z = 0.91 (P = 0.36)

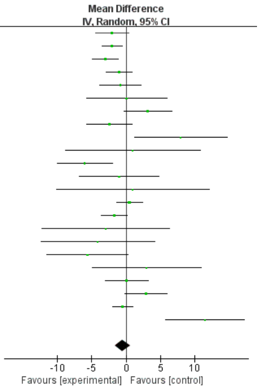

Figure S1.7. Forest plot of the effect of exercise on HRrest

Study or Subgroup	Experimental Mean	Experimental SD	Experimental Total	Control Mean	Control SD	Control Total	Weight	Mean Difference IV, Random, 95% CI
Bharath 2018	67	4.47	20	69	8.94	20	3.1%	-2.00 [-6.38, 2.38]
Cao 2022	74.3	3.3	20	74.9	2.1	20	7.7%	-0.60 [-2.31, 1.11]
Cvetković 2018(1)	77.5	9.5	10	84.15	9.83	7	0.9%	-6.65 [-16.01, 2.71]
Cvetković 2018(2)	76.9	11.11	11	84.15	9.83	7	0.8%	-7.25 [-17.05, 2.55]
Daley 2006(1)	80.42	4.99	27	81.29	4.9	13	4.5%	-0.87 [-4.13, 2.39]
Daley 2006(2)	82.16	4.32	22	81.29	4.9	13	4.6%	0.87 [-2.35, 4.09]
Hamila 2018(1)	85.7	2	7	89.8	3.3	2	2.7%	-4.10 [-8.91, 0.71]
Hamila 2018(2)	87	1	8	89.8	3.3	3	3.8%	-2.80 [-6.60, 1.00]
Hamila 2018(3)	87.9	3.2	8	89.8	3.3	3	3.2%	-1.90 [-6.24, 2.44]
Harder-Lauridsen 2014	75.5	7.4	18	82.9	12.15	18	1.7%	-7.40 [-13.97, -0.83]
Heidarianpour 2023	83.4	9.85	15	99.71	10.77	15	1.4%	-16.31 [-23.70, -8.92]
Hongling 2012	72	3	30	76	4	30	7.5%	-4.00 [-5.79, -2.21]
Jain 2022	81	8	40	79	9	27	3.3%	2.00 [-2.20, 6.20]
Jian 2014	75.71	2.01	44	78.91	1.98	44	9.7%	-3.20 [-4.03, -2.37]
Kim 2007	70.4	7.86	14	76.2	8.31	12	1.8%	-5.80 [-12.05, 0.45]
Pena 2022	77	8.83	78	82	12.17	37	3.1%	-5.00 [-9.38, -0.62]
Plavsic 2020	99	16	22	102	19	22	0.7%	-3.00 [-13.38, 7.38]
Racil(1) 2016(1)	67	2	17	70	2	7	7.6%	-3.00 [-4.76, -1.24]
Racil(1) 2016(2)	68	2	16	70	2	7	7.6%	-2.00 [-3.78, -0.22]
Salus(1) 2022	73.1	12.72	14	85.5	8.98	14	1.1%	-12.40 [-20.56, -4.24]
Saygın 2011	88.46	8.17	20	94.22	8.77	19	2.3%	-5.76 [-11.09, -0.43]
Shu 2023(1)	74	2.4	16	75.6	1.9	8	7.6%	-1.60 [-3.37, 0.17]
Shu 2023(2)	73.9	3.3	15	75.6	1.9	8	6.7%	-1.70 [-3.83, 0.43]
Wong 2008	71	2.5	12	76	3.9	12	5.7%	-5.00 [-7.62, -2.38]
Wong 2018	69	11.62	15	74	15.49	15	0.8%	-5.00 [-14.80, 4.80]
**Total (95% CI)**			**519**			**383**	**100.0%**	**-2.89 [-3.82, -1.97]**

Heterogeneity: Tau² = 2.11; Chi² = 51.89, df = 24 (P = 0.0008); I² = 54%
Test for overall effect: Z = 6.14 (P < 0.00001)

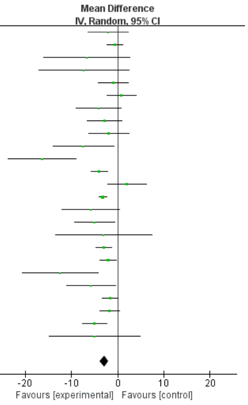

Figure S1.8. Forest plot of the effect of exercise on SBP

Study or Subgroup	Experimental Mean	Experimental SD	Experimental Total	Control Mean	Control SD	Control Total	Weight	Mean Difference IV, Random, 95% CI
Abassi 2022(1)	122	2.3	13	125	3.8	6	1.6%	-3.00 [-6.29, 0.29]
Abassi 2022(2)	123	3.8	13	125	3.8	6	1.6%	-2.00 [-5.68, 1.68]
Aguilar-Cordero 2020	124.69	5.72	49	131.16	12.24	49	1.6%	-6.47 [-10.25, -2.69]
Bezerra 2023	111.15	11.3	20	111.26	6.41	21	1.4%	-0.11 [-5.77, 5.55]
Bharath 2018	120	1.79	20	124	6.26	20	1.7%	-4.00 [-6.85, -1.15]
Boer 2014(1)	113	8	17	119	10	7	1.1%	-6.00 [-14.33, 2.33]
Boer 2014(2)	119	9	15	119	10	7	1.1%	0.00 [-8.70, 8.70]
Boff 2020	122.84	10.37	34	121	11.08	31	1.4%	1.84 [-3.39, 7.07]
Bruyndonckx 2015	108	6	27	120	11	21	1.4%	-12.00 [-17.22, -6.78]
Chuensiri 2018(1)	125	13.27	11	121	13.27	5	0.7%	4.00 [-10.03, 18.03]
Chuensiri 2018(2)	116	11.62	15	121	13.27	6	0.8%	-5.00 [-17.14, 7.14]
Cvetković 2018(1)	117.5	11.12	10	124.85	11.58	7	0.9%	-7.35 [-18.35, 3.65]
Cvetković 2018(2)	116.5	14.15	11	124.85	11.58	7	0.8%	-8.35 [-20.33, 3.63]
Davis 2020	105.1	6.88	90	102.3	6	85	1.7%	2.80 [0.89, 4.71]
Espinoza-Silva 2019(1)	103.06	13.53	69	102.6	10.14	30	1.5%	0.46 [-4.37, 5.29]
Espinoza-Silva 2019(2)	103.03	8.49	141	102.91	8.9	34	1.6%	0.12 [-3.18, 3.42]
Faria 2020(1)	115.7	8.05	24	117.3	8.29	13	1.4%	-1.60 [-7.14, 3.94]
Faria 2020(2)	114.5	8.29	26	117.3	8.29	13	1.4%	-2.80 [-8.32, 2.72]
Farpour-Lambert 2009	120.7	13.69	22	125.7	14.85	22	1.1%	-5.00 [-13.44, 3.44]
Gonzalez-Galvez 2024(1)	123	11.4	9	126.5	13.16	6	0.7%	-3.50 [-16.40, 9.40]
Gonzalez-Galvez 2024(2)	116.3	11.28	11	126.5	13.16	6	0.8%	-10.20 [-22.66, 2.26]
Guijun 2009	114.97	1.5	16	123.15	2.7	10	1.7%	-8.18 [-10.01, -6.35]
Hamila 2018(1)	116.4	7.1	7	125.5	11.2	2	0.5%	-9.10 [-25.49, 7.29]
Hamila 2018(2)	118.6	5.7	8	125.5	11.2	3	0.7%	-6.90 [-20.17, 6.37]
Hamila 2018(3)	121.8	6.2	8	125.5	11.2	3	0.7%	-3.70 [-17.08, 9.68]
Harder-Lauridsen 2014	104.5	9.38	18	102.3	9.98	18	1.3%	2.20 [-4.13, 8.53]
Heidarianpour 2023	108.22	12.39	15	118.2	13.47	15	1.0%	-9.98 [-19.24, -0.72]
Hongling 2012	113	5	30	116	6	30	1.7%	-3.00 [-5.79, -0.21]
Horner 2015(1)	109.58	19.56	27	116.63	10.23	8	0.9%	-7.05 [-17.28, 3.18]
Horner 2015(2)	110.89	8.04	23	116.63	10.23	8	1.2%	-5.74 [-13.55, 2.07]
Jain 2022	114	8	40	116	8	27	1.6%	-2.00 [-5.91, 1.91]
Jian 2014	120.55	2.56	44	129.72	2.24	44	1.8%	-9.17 [-10.18, -8.16]
Kai 2024	127	1	20	132	3	24	1.8%	-5.00 [-6.28, -3.72]
Kelishadi 2008	116.9	45.88	45	116.7	45.9	47	0.4%	0.20 [-18.56, 18.96]
Kelly 2004	120	6.32	10	122	15.81	10	0.9%	-2.00 [-12.55, 8.55]
Kim 2007	122.9	7.11	14	124.2	10.74	12	1.2%	-1.30 [-8.43, 5.83]
Kim 2011	114	10.39	12	118	15.81	10	0.8%	-4.00 [-15.43, 7.43]
Kim 2020	127.6	2.3	24	133.2	4.1	24	1.7%	-5.60 [-7.48, -3.72]
Larsen 2016	100.1	6.9	51	103.1	7.7	44	1.7%	-3.00 [-5.96, -0.04]
Lee 2010(1)	119.34	15.19	20	121.12	16.03	9	0.8%	-1.78 [-14.19, 10.63]
Lee 2010(2)	116.53	10.99	16	121.12	16.03	9	0.8%	-4.59 [-16.37, 7.19]
Liqun 2014	106.5	12.5	76	109.8	9.3	97	1.6%	-3.30 [-6.66, 0.06]
Luo 2013(1)	100.72	6.21	43	112.21	8.26	34	1.6%	-11.49 [-14.83, -8.15]
Luo 2013(2)	97.5	6.22	52	107.89	9.11	38	1.6%	-10.39 [-13.74, -7.04]
Machado 2022	100.3	4.8	20	110.2	13.3	15	1.3%	-9.90 [-16.95, -2.85]
Meng 2022(1)	110	5	12	116	4	6	1.5%	-6.00 [-10.27, -1.73]
Meng 2022(2)	114	7	11	116	4	7	1.5%	-2.00 [-7.09, 3.09]
Meyer 2006	120	13.3	33	133	19.6	34	1.2%	-13.00 [-21.00, -5.00]
Murphy 2009	111.4	8	23	114.2	5.7	12	1.5%	-2.80 [-7.39, 1.79]
Park 2007	112	7.6	19	119.3	7.5	21	1.5%	-7.30 [-11.99, -2.61]
Plavsic 2020	110.3	8.5	22	114.4	6.8	22	1.5%	-4.10 [-8.65, 0.45]
Racil(1) 2016(1)	115	5	17	118	5	7	1.5%	-3.00 [-7.40, 1.40]
Racil(1) 2016(2)	115	5	16	118	5	7	1.5%	-3.00 [-7.44, 1.44]
Racil 2024(1)	119.15	5	12	119.1	4.7	5	1.5%	0.05 [-4.95, 5.05]
Racil 2024(2)	119.33	4.7	12	119.1	4.7	6	1.5%	0.23 [-4.38, 4.84]
Salus(1) 2022	125.9	8.98	14	120.7	16.46	14	1.0%	5.20 [-4.62, 15.02]
Savoye 2007	121	11	75	120.4	12.54	44	1.5%	0.60 [-3.86, 5.06]
Savoye 2011	121.7	10.24	75	121.7	10.82	44	1.6%	0.00 [-3.95, 3.95]
Saygın 2011	121.6	1.03	20	135.1	0.84	19	1.8%	-13.50 [-14.09, -12.91]
Seo 2019	123.06	11.07	26	123.91	15.95	44	1.3%	-0.85 [-7.20, 5.50]
Shalitin 2009	111	17.8	55	112	18.54	55	1.3%	-1.00 [-7.79, 5.79]
Shu 2023(1)	111.9	4.7	16	117.1	5	8	1.6%	-5.20 [-9.36, -1.04]
Shu 2023(2)	113.2	6.3	15	117.1	5	8	1.5%	-3.90 [-8.61, 0.81]
Sigal 2014(1)	111	8.66	75	110	8.72	25	1.6%	1.00 [-2.94, 4.94]
Sigal 2014(2)	112	8.83	78	110	8.72	25	1.6%	2.00 [-1.94, 5.94]
Sigal 2014(3)	110	8.66	75	110	8.72	26	1.6%	0.00 [-3.88, 3.88]
Son 2017	123.7	9.53	20	130.6	6.22	20	1.5%	-6.90 [-11.89, -1.91]
Tjonna 2009	119.4	7.16	20	122.5	7.04	22	1.5%	-3.10 [-7.40, 1.20]
Vasconcellos 2016	123	13	10	129	6	10	1.1%	-6.00 [-14.87, 2.87]
Wong 2008	113.8	7.1	12	117	6.2	12	1.4%	-3.20 [-8.53, 2.13]
Woo 2012	103.05	7.75	10	116.3	7.18	10	1.3%	-13.25 [-19.80, -6.70]
Yu 2020	102.7	8.1	99	102.1	8.9	72	1.7%	0.60 [-2.00, 3.20]
Yuanyuan 2019(1)	105.47	7.21	10	106.57	7.62	3	1.0%	-1.10 [-10.81, 8.61]
Yuanyuan 2019(2)	99.01	7.24	10	106.57	7.62	3	1.0%	-7.56 [-17.28, 2.16]
Yuanyuan 2019(3)	97.65	6.62	9	106.57	7.62	4	1.1%	-8.92 [-17.55, -0.29]
Zhaogang 2021	98.24	9.64	49	101.24	10.04	49	1.6%	-3.00 [-6.90, 0.90]
**Total (95% CI)**			**2236**			**1587**	**100.0%**	**-3.90 [-5.34, -2.46]**

Heterogeneity: Tau² = 30.25; Chi² = 876.13, df = 75 (P < 0.00001); I² = 91%
Test for overall effect: Z = 5.31 (P < 0.00001)

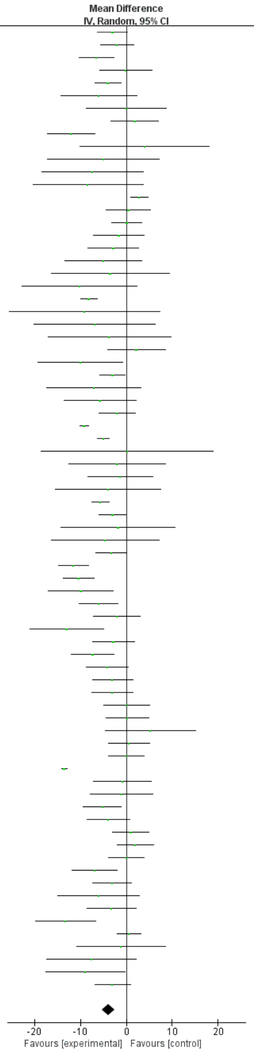

Figure S1.9. Forest plot of the effect of exercise on DBP

Study or Subgroup	Experimental Mean	Experimental SD	Experimental Total	Control Mean	Control SD	Control Total	Weight	Mean Difference IV, Random, 95% CI
Abassi 2022(1)	76.7	4.22	13	77.8	4	6	1.6%	-1.10 [-5.04, 2.84]
Abassi 2022(2)	77.4	4.42	13	77.8	4	6	1.6%	-0.40 [-4.40, 3.60]
Aguilar-Cordero 2020	72.77	4.97	49	78.41	8.7	49	1.8%	-5.64 [-8.45, -2.83]
Bezerra 2023	70.05	11.73	20	69.51	7.69	21	1.2%	0.54 [-5.56, 6.64]
Bharath 2018	78	6.93	20	80	10.73	20	1.2%	-2.00 [-7.60, 3.60]
Boer 2014(1)	77	8	17	72	5	7	1.3%	5.00 [-0.31, 10.31]
Boer 2014(2)	73	9	15	72	5	7	1.2%	1.00 [-4.87, 6.87]
Boff 2020	72.61	14	34	73.22	7.72	31	1.3%	-0.61 [-6.04, 4.82]
Bruyndonckx 2015	60	4	27	63	7	21	1.7%	-3.00 [-6.35, 0.35]
Chuensiri 2018(1)	77	6.63	11	74	6.63	5	1.0%	3.00 [-4.01, 10.01]
Chuensiri 2018(2)	71	7.75	15	74	6.63	6	1.1%	-3.00 [-9.60, 3.60]
Cvetković 2018(1)	64	3.94	10	64.57	8.42	7	1.1%	-0.57 [-7.27, 6.13]
Cvetković 2018(2)	66	8	11	64.57	8.42	7	0.9%	1.43 [-6.40, 9.26]
Davis 2020	59.1	5.38	90	58	5.23	85	2.0%	1.10 [-0.47, 2.67]
Espinoza-Silva 2019(1)	61.42	14.53	69	61.45	9.09	30	1.4%	-0.03 [-4.76, 4.70]
Espinoza-Silva 2019(2)	62.32	8.66	141	57.41	9.24	34	1.7%	4.91 [1.49, 8.33]
Faria 2020(1)	70.5	6.87	24	70.8	7.06	13	1.4%	-0.30 [-5.02, 4.42]
Faria 2020(2)	68.2	7.06	26	70.8	7.06	13	1.4%	-2.60 [-7.30, 2.10]
Farpour-Lambert 2009	70.9	5	22	71.8	5.43	22	1.7%	-0.90 [-3.98, 2.18]
Gonzalez-Galvez 2024(1)	61.4	7.8	9	67.4	9.01	6	0.8%	-6.00 [-14.83, 2.83]
Gonzalez-Galvez 2024(2)	64.9	7.63	11	67.4	9.01	6	0.8%	-2.50 [-11.00, 6.00]
Guijun 2009	84	1.28	16	88.5	1.57	10	2.0%	-4.50 [-5.66, -3.34]
Hamila 2018(1)	64	3.9	7	68.7	6.9	2	0.7%	-4.70 [-14.69, 5.29]
Hamila 2018(2)	60.1	6.3	8	68.7	6.9	3	0.8%	-8.60 [-17.55, 0.35]
Hamila 2018(3)	68.3	5.3	8	68.7	6.9	3	0.8%	-0.40 [-9.03, 8.23]
Harder-Lauridsen 2014	60.9	11.24	18	62.6	11.1	18	1.0%	-1.70 [-9.00, 5.60]
Heidarianpour 2023	77.53	4.35	15	80	4.7	15	1.7%	-2.47 [-5.71, 0.77]
Hongling 2012	71	5	30	73	6	30	1.8%	-2.00 [-4.79, 0.79]
Horner 2015(1)	58.75	10.88	27	60.88	6.48	8	1.2%	-2.13 [-8.21, 3.95]
Horner 2015(2)	58.56	7.29	23	60.88	6.48	8	1.3%	-2.32 [-7.71, 3.07]
Jain 2022	71	7	40	75	9	27	1.5%	-4.00 [-8.03, 0.03]
Jian 2014	75.56	1.45	44	84.81	1.9	44	2.1%	-9.25 [-9.96, -8.54]
Kai 2024	81	1	20	82	1	24	2.1%	-1.00 [-1.59, -0.41]
Kelishadi 2008	74.6	11.81	45	74.4	11.74	47	1.4%	0.20 [-4.61, 5.01]
Kelly 2004	65	6.32	10	68	9.49	10	1.0%	-3.00 [-10.07, 4.07]
Kim 2007	81.4	10.85	14	80.8	10.74	12	0.8%	0.60 [-7.72, 8.92]
Kim 2011	69	6.93	12	65	6.32	10	1.3%	4.00 [-1.54, 9.54]
Kim 2020	80.2	1.4	24	81.2	2.8	24	2.0%	-1.00 [-2.25, 0.25]
Liqun 2014	70.6	8.9	76	72.4	9	97	1.8%	-1.80 [-4.49, 0.89]
Luo 2013(1)	57.3	7.35	43	64.09	6.15	34	1.7%	-6.79 [-9.81, -3.77]
Luo 2013(2)	57	5.32	52	60.48	7.32	38	1.8%	-3.48 [-6.22, -0.74]
Machado 2022	56.9	5.8	20	63.2	13.4	15	1.0%	-6.30 [-13.54, 0.94]
Meng 2022(1)	67	3	12	71	3.7	6	1.7%	-4.00 [-7.41, -0.59]
Meng 2022(2)	68	4	11	71	3.7	7	1.6%	-3.00 [-6.62, 0.62]
Murphy 2009	70.9	8.4	23	71.5	7.5	12	1.3%	-0.60 [-6.06, 4.86]
Park 2007	67.5	4.6	19	69.9	6.5	21	1.7%	-2.40 [-5.87, 1.07]
Plavsic 2020	69.2	6.2	22	72.4	6.6	22	1.6%	-3.20 [-6.98, 0.58]
Racil(1) 2016(1)	74	2	17	74	2	7	2.0%	0.00 [-1.76, 1.76]
Racil(1) 2016(2)	75	4	16	74	2	7	1.8%	1.00 [-1.46, 3.46]
Racil 2024(1)	78.61	3.6	12	80.2	3.5	5	1.6%	-1.59 [-5.27, 2.09]
Racil 2024(2)	79.23	3.6	12	80.2	3.5	6	1.7%	-0.97 [-4.43, 2.49]
Salus(1) 2022	71.3	13.1	14	78.4	8.61	14	0.9%	-7.10 [-15.31, 1.11]
Savoye 2007	66.4	9.41	75	67.8	9.17	44	1.7%	-1.40 [-4.85, 2.05]
Savoye 2011	65.3	9.3	45	64.3	9.7	31	1.5%	1.00 [-3.36, 5.36]
Seo 2019	61.44	9.47	26	65.59	6.43	44	1.5%	-4.15 [-8.26, -0.04]
Shalitin 2009	64	12.9	55	63	13.65	55	1.4%	1.00 [-3.96, 5.96]
Shu 2023(1)	66.1	2.4	16	70.3	4.8	8	1.6%	-4.20 [-7.73, -0.67]
Shu 2023(2)	69	3.5	15	70.3	4.8	8	1.6%	-1.30 [-5.07, 2.47]
Sigal 2014(1)	72	8.66	75	73	8.72	25	1.6%	-1.00 [-4.94, 2.94]
Sigal 2014(2)	74	8.83	78	73	8.72	25	1.6%	1.00 [-2.94, 4.94]
Sigal 2014(3)	73	8.66	75	73	8.72	26	1.6%	0.00 [-3.88, 3.88]
Son 2017	79.8	6.62	20	82.4	8.9	20	1.4%	-2.60 [-7.46, 2.26]
Tjonna 2009	64.9	7.16	20	67.3	7.5	22	1.5%	-2.40 [-6.83, 2.03]
Vasconcellos 2016	79	14	10	79	4	10	0.8%	0.00 [-9.02, 9.02]
Wong 2008	71.7	7.5	12	70.8	6.7	12	1.2%	0.90 [-4.79, 6.59]
Woo 2012	73.45	9.57	10	72.25	5.76	10	1.0%	1.20 [-5.72, 8.12]
Yu 2020	67.4	6.1	99	68.5	6.5	72	1.9%	-1.10 [-3.02, 0.82]
Yuanyuan 2019(1)	66.89	5.79	10	67.13	5.88	3	0.9%	-0.24 [-7.80, 7.32]
Yuanyuan 2019(2)	61.02	5.76	10	67.13	5.88	3	0.9%	-6.11 [-13.66, 1.44]
Yuanyuan 2019(3)	60.12	5.24	9	67.13	5.88	4	1.1%	-7.01 [-13.71, -0.31]
Zhaogang 2021	66.17	6.27	49	67.54	7.58	49	1.8%	-1.37 [-4.12, 1.38]
**Total (95% CI)**			**2066**			**1459**	**100.0%**	**-1.70 [-2.68, -0.71]**

Heterogeneity: Tau² = 12.08; Chi² = 506.73, df = 70 (P < 0.00001); I² = 86%
Test for overall effect: Z = 3.37 (P = 0.0007)

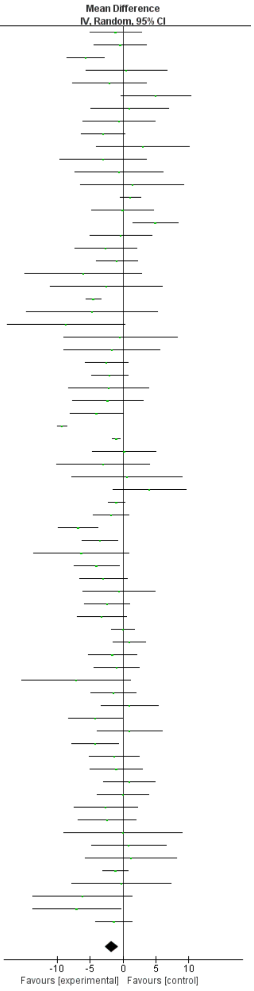

Figure S1.10. Forest plot of the effect of exercise on TC

Study or Subgroup	Experimental Mean	Experimental SD	Experimental Total	Control Mean	Control SD	Control Total	Weight	Mean Difference IV, Random, 95% CI
Ahmadi 2020(1)	4.08	0.62	28	4.18	1.12	9	0.7%	-0.10 [-0.87, 0.67]
Ahmadi 2020(2)	4.08	1.04	29	4.18	1.12	9	0.6%	-0.10 [-0.92, 0.72]
Ahmadi 2020(3)	4.25	0.93	29	4.18	1.12	9	0.6%	0.07 [-0.74, 0.88]
Benson 2008	4.3	1.1	32	4.2	0.7	35	1.2%	0.10 [-0.35, 0.55]
Bezerra 2023	4.2	0.74	20	4.54	0.81	21	1.1%	-0.34 [-0.81, 0.13]
Boer 2014(1)	4	0.59	17	4.45	0.67	7	0.9%	-0.45 [-1.02, 0.12]
Boer 2014(2)	4.24	0.81	15	4.45	0.67	7	0.8%	-0.21 [-0.85, 0.43]
Bowen 2010	4.1	0.89	22	4.05	0.59	18	1.1%	0.05 [-0.41, 0.51]
Bruyndonckx 2015	3.63	0.51	27	3.98	0.5	21	1.5%	-0.35 [-0.64, -0.06]
Chae 2010	4.2	0.76	19	4.51	0.59	19	1.2%	-0.31 [-0.74, 0.12]
Chuensiri 2018(1)	3.78	0.69	11	4.45	0.77	5	0.6%	-0.67 [-1.46, 0.12]
Chuensiri 2018(2)	3.54	0.7	15	4.45	0.77	6	0.7%	-0.91 [-1.62, -0.20]
Davis 2020	4.23	0.95	90	4.19	0.53	85	1.7%	0.04 [-0.19, 0.27]
DeBar 2012	4.12	0.8	74	4.15	0.63	42	1.6%	-0.03 [-0.29, 0.23]
Dias 2018(1)	3.79	0.64	19	4.25	0.81	9	0.9%	-0.46 [-1.06, 0.14]
Dias 2018(2)	4.26	0.87	22	4.25	0.81	10	0.9%	0.01 [-0.61, 0.63]
Duft 2020	4.32	0.89	18	4.4	0.91	19	0.9%	-0.08 [-0.66, 0.50]
Elmahgoub 2009	4.23	0.9	15	4.37	0.77	15	0.9%	-0.14 [-0.74, 0.46]
Faria 2020(1)	3.6	0.75	24	3.88	0.77	13	1.0%	-0.28 [-0.80, 0.24]
Faria 2020(2)	3.81	0.76	26	3.88	0.77	13	1.0%	-0.07 [-0.58, 0.44]
Farpour-Lambert 2009	4.04	0.79	22	4	0.5	22	1.3%	0.04 [-0.35, 0.43]
Ghorbanian 2013	4.75	0.66	15	5.87	0.9	15	0.9%	-1.12 [-1.68, -0.56]
Guijun 2009	4.59	0.15	16	5.02	0.12	10	1.9%	-0.43 [-0.53, -0.33]
Gulijianati 2022	4.07	0.62	47	5.93	0.77	46	1.5%	-1.86 [-2.14, -1.58]
Guo 2015	4.5	0.7	23	5	1.2	12	0.7%	-0.50 [-1.24, 0.24]
Harder-Lauridsen 2014	3.8	0.84	14	4.3	0.46	14	1.1%	-0.50 [-1.00, 0.00]
Hong 2016	3.57	0.74	55	4.06	0.73	55	1.6%	-0.49 [-0.76, -0.22]
Hongling 2012	4.1	0.7	30	4.2	1	30	1.2%	-0.10 [-0.54, 0.34]
Horner 2015(1)	3.87	0.87	27	3.72	0.48	8	1.1%	0.15 [-0.32, 0.62]
Horner 2015(2)	3.24	0.6	23	3.72	0.48	8	1.2%	-0.48 [-0.89, -0.07]
Jian 2014	4.2	0.13	44	4.78	0.12	44	1.9%	-0.58 [-0.63, -0.53]
Jianjun 2012	3.86	0.63	20	5.78	0.55	15	1.3%	-1.92 [-2.31, -1.53]
Jun 2006(1)	3.72	0.6	33	3.81	0.75	31	1.4%	-0.09 [-0.42, 0.24]
Jun 2006(2)	3.7	0.56	35	3.87	0.77	32	1.4%	-0.17 [-0.49, 0.15]
Ke 2024	4.1	0.4	10	4.9	0.9	10	0.9%	-0.80 [-1.41, -0.19]
Kelishadi 2008	4.18	0.56	45	4.14	0.56	47	1.6%	0.04 [-0.19, 0.27]
Kelly 2004	4.01	0.7	10	3.92	0.82	10	0.8%	0.09 [-0.58, 0.76]
Kim 2007	4.02	0.69	14	4.63	0.9	12	0.9%	-0.61 [-1.23, 0.01]
Kim 2022	4.17	0.82	13	3.74	0.64	13	0.9%	0.43 [-0.14, 1.00]
Lee 2010(1)	4.55	0.48	20	4.46	0.88	9	0.9%	0.09 [-0.52, 0.70]
Lee 2010(2)	4.52	0.89	16	4.46	0.88	9	0.7%	0.06 [-0.66, 0.78]
Liangmei 2015	3.63	0.83	12	4.1	0.89	12	0.8%	-0.47 [-1.16, 0.22]
Lidan 2012	3.93	0.27	30	4.35	0.58	30	1.6%	-0.42 [-0.65, -0.19]
Lingling 2021	5	0.87	20	5.47	1.29	20	0.8%	-0.47 [-1.15, 0.21]
Liqun 2014	4.32	0.78	76	4.44	0.81	97	1.6%	-0.12 [-0.36, 0.12]
Lira 2017(1)	4.41	1.04	26	4.24	0.93	16	0.9%	0.17 [-0.44, 0.78]
Lira 2017(2)	4.3	0.86	25	4.24	0.93	17	1.0%	0.06 [-0.50, 0.62]
Liu 2018	3.52	0.66	30	4	0.88	20	1.2%	-0.48 [-0.93, -0.03]
Lopes 2016	4.31	1.09	17	4.42	0.92	16	0.8%	-0.11 [-0.80, 0.58]
Luo 2013(1)	3.58	0.74	43	4.33	0.56	34	1.5%	-0.75 [-1.04, -0.46]
Luo 2013(2)	3.64	0.78	52	4.47	0.54	38	1.6%	-0.83 [-1.10, -0.56]
Meng 2022(1)	4	0.5	12	4.2	0.6	6	1.0%	-0.20 [-0.76, 0.36]
Meng 2022(2)	3.9	0.5	11	4.2	0.6	7	1.0%	-0.30 [-0.83, 0.23]
Mohammadi 2019	4.24	0.49	10	4.41	0.36	10	1.3%	-0.17 [-0.55, 0.21]
Monteiro 2015(1)	2.91	0.65	18	3.37	0.86	8	0.8%	-0.46 [-1.13, 0.21]
Monteiro 2015(2)	2.85	0.49	14	3.37	0.86	8	0.8%	-0.52 [-1.17, 0.13]
Morgan 2021	3.87	0.39	15	4.96	0.51	15	1.4%	-1.09 [-1.41, -0.77]
Murphy 2009	4.16	0.82	23	4.11	0.5	12	1.2%	0.05 [-0.39, 0.49]
Park 2007	4.16	0.62	19	4.54	0.74	21	1.2%	-0.38 [-0.80, 0.04]
Plavsic 2020	4.02	0.74	22	3.93	0.6	22	1.3%	0.09 [-0.31, 0.49]
Racil 2013(1)	3.53	0.31	11	3.86	0.16	6	1.7%	-0.33 [-0.55, -0.11]
Racil 2013(2)	3.9	0.32	11	3.86	0.16	6	1.7%	0.04 [-0.19, 0.27]
Ramezani 2017(1)	4.02	1.31	15	6.29	1.21	5	0.3%	-2.27 [-3.52, -1.02]
Ramezani 2017(2)	4.49	1.1	15	6.29	1.21	5	0.3%	-1.80 [-3.00, -0.60]
Ramezani 2017(3)	4.24	1.26	15	6.29	1.21	5	0.3%	-2.05 [-3.29, -0.81]
Salus(2) 2022	3.99	0.37	14	4.64	0.75	14	1.2%	-0.65 [-1.09, -0.21]
Savoye 2007	4.16	0.84	75	4.34	0.84	44	1.5%	-0.18 [-0.49, 0.13]
Savoye 2011	4.2	0.76	75	4.25	0.81	44	1.5%	-0.05 [-0.34, 0.24]
Saygın 2011	2.48	0.85	20	3.03	0.63	19	1.1%	-0.55 [-1.02, -0.08]
Seo 2012	4.39	2.54	10	4.05	1.79	10	0.1%	0.34 [-1.59, 2.27]
Shalitin 2009	4.28	0.81	55	4.17	0.88	55	1.5%	0.11 [-0.21, 0.43]
Staiano 2017	4.08	0.38	20	4.21	0.63	18	1.4%	-0.13 [-0.47, 0.21]
Staiano 2018	3.8	0.95	22	4.49	0.98	23	1.0%	-0.69 [-1.25, -0.13]
Sun 2011	3.9	0.6	25	4	0.6	17	1.3%	-0.10 [-0.47, 0.27]
Tas 2023	3.78	0.7	31	3.82	0.7	6	0.9%	-0.04 [-0.65, 0.57]
Tsang 2009	4.1	0.83	11	3.81	0.85	8	0.7%	0.29 [-0.48, 1.06]
Vasconcellos 2016	3.88	0.65	10	4.69	0.78	10	0.8%	-0.81 [-1.44, -0.18]
Wen 2008	3.89	0.53	24	4.83	0.8	13	1.1%	-0.94 [-1.42, -0.46]
Wong 2008	4.7	1	12	4.5	0.7	12	0.8%	0.20 [-0.49, 0.89]
Woo 2004	4.5	0.8	41	4.5	0.8	41	1.4%	0.00 [-0.35, 0.35]
Woo 2012	4.07	0.83	10	4.76	0.6	10	0.8%	-0.69 [-1.32, -0.06]
Xianbo 2011	0.9	0.4	25	1.2	0.5	17	1.5%	-0.30 [-0.58, -0.02]
Xinhui 2016	3.64	0.84	14	4.11	0.89	14	0.8%	-0.47 [-1.11, 0.17]
Xishao 2009	3.93	0.27	34	4.35	0.58	33	1.7%	-0.42 [-0.64, -0.20]
Xishao 2010	3.93	0.27	100	4.35	0.58	100	1.8%	-0.42 [-0.55, -0.29]
Yanqin 2017	4.32	0.14	35	4.76	0.11	33	1.9%	-0.44 [-0.50, -0.38]
Zehsaz 2016	3.91	0.21	16	4.19	0.24	16	1.8%	-0.28 [-0.44, -0.12]
Zehsaz 2017	4.19	1.14	16	4.2	1.08	16	0.7%	-0.01 [-0.78, 0.76]
Zhaogang 2021	3	0.78	49	4.01	0.84	49	1.4%	-1.01 [-1.33, -0.69]
**Total (95% CI)**			**2370**			**1882**	**100.0%**	**-0.35 [-0.43, -0.27]**

Heterogeneity: $Tau^2 = 0.08$; $Chi^2 = 452.93$, $df = 88$ ($P < 0.00001$); $I^2 = 81\%$
Test for overall effect: $Z = 8.92$ ($P < 0.00001$)

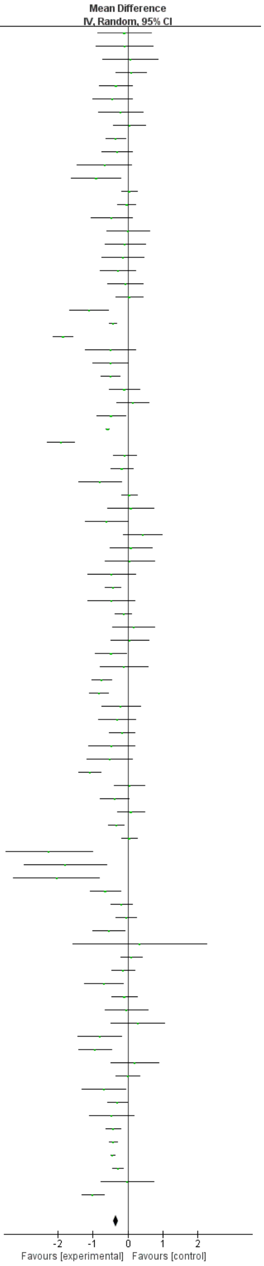

Figure S1.11. Forest plot of the effect of exercise on TG

Study or Subgroup	Experimental Mean	Experimental SD	Experimental Total	Control Mean	Control SD	Control Total	Weight	Mean Difference IV, Random, 95% CI
Ahmadi 2020(1)	1.33	0.5	28	1.56	0.62	9	0.8%	-0.23 [-0.68, 0.22]
Ahmadi 2020(2)	1.44	0.85	29	1.56	0.62	9	0.6%	-0.12 [-0.63, 0.39]
Ahmadi 2020(3)	1.42	0.7	29	1.56	0.62	9	0.7%	-0.14 [-0.62, 0.34]
Bezerra 2023	1.19	0.51	20	1.44	0.63	21	1.0%	-0.25 [-0.60, 0.10]
Boer 2014(1)	0.8	0.19	17	1.07	0.97	7	0.4%	-0.27 [-0.99, 0.45]
Boer 2014(2)	0.99	0.57	15	1.07	0.97	7	0.4%	-0.08 [-0.85, 0.69]
Boff 2020	1	0.45	34	1.23	0.63	31	1.2%	-0.23 [-0.50, 0.04]
Bowen 2010	0.89	0.44	22	1.19	0.46	18	1.1%	-0.30 [-0.58, -0.02]
Chae 2010	0.97	0.39	19	1.48	0.39	19	1.2%	-0.51 [-0.76, -0.26]
Chuensiri 2018(1)	0.88	0.37	11	1.23	0.37	5	0.9%	-0.35 [-0.74, 0.04]
Chuensiri 2018(2)	0.97	0.39	15	1.23	0.37	6	0.9%	-0.26 [-0.62, 0.10]
Davis 2020	0.78	0.61	90	0.9	0.55	85	1.4%	-0.12 [-0.29, 0.05]
DeBar 2012	1.15	0.59	74	1.21	0.66	42	1.3%	-0.06 [-0.30, 0.18]
Dias 2018(1)	0.96	0.37	19	1.26	0.73	9	0.7%	-0.30 [-0.81, 0.21]
Dias 2018(2)	1	0.47	22	1.26	0.73	10	0.7%	-0.26 [-0.75, 0.23]
Elmahgoub 2009	1.25	0.65	15	1.26	1.08	15	0.5%	-0.01 [-0.65, 0.63]
Faria 2020(1)	0.89	0.54	24	1.12	0.56	13	0.9%	-0.23 [-0.60, 0.14]
Faria 2020(2)	0.99	0.56	26	1.12	0.56	13	0.9%	-0.13 [-0.50, 0.24]
Farpour-Lambert 2009	0.64	0.32	22	0.82	0.49	22	1.2%	-0.18 [-0.42, 0.06]
Ghorbanian 2013	1.64	0.55	15	2.29	0.79	15	0.7%	-0.65 [-1.14, -0.16]
Guijun 2009	1.25	0.29	16	1.92	0.37	10	1.2%	-0.67 [-0.94, -0.40]
Gulijianati 2022	1.57	0.22	47	1.88	0.19	46	1.6%	-0.31 [-0.39, -0.23]
Guo 2015	1.51	0.86	23	1.31	0.56	12	0.7%	0.20 [-0.27, 0.67]
Harder-Lauridsen 2014	0.4	0.29	14	0.8	0.84	14	0.7%	-0.40 [-0.87, 0.07]
Hong 2016	1.07	0.22	55	1.28	0.27	55	1.6%	-0.21 [-0.30, -0.12]
Hongling 2012	0.9	0.5	30	1	0.4	30	1.3%	-0.10 [-0.33, 0.13]
Horner 2015(1)	0.88	0.68	27	0.88	0.49	8	0.8%	0.00 [-0.43, 0.43]
Horner 2015(2)	0.8	0.57	23	0.88	0.49	8	0.8%	-0.08 [-0.49, 0.33]
Jian 2014	1.05	0.21	44	1.48	0.36	44	1.6%	-0.43 [-0.55, -0.31]
Jianjun 2012	0.81	0.34	20	1.35	0.47	15	1.1%	-0.54 [-0.82, -0.26]
Jun 2006(1)	1.14	0.55	33	1.3	0.78	31	1.0%	-0.16 [-0.49, 0.17]
Jun 2006(2)	1.08	0.68	35	1.08	0.65	32	1.0%	0.00 [-0.32, 0.32]
Ke 2024	0.9	0.3	10	1.5	0.3	10	1.2%	-0.60 [-0.86, -0.34]
Kelishadi 2008	1.46	0.14	45	1.43	0.14	47	1.7%	0.03 [-0.03, 0.09]
Kelly 2004	1.22	0.54	10	1.32	0.7	10	0.6%	-0.10 [-0.65, 0.45]
Kim 2007	0.78	0.38	14	1.12	0.57	12	0.9%	-0.34 [-0.72, 0.04]
Kim 2022	0.62	0.15	13	0.65	0.09	13	1.6%	-0.03 [-0.13, 0.07]
Larsen 2016	0.89	0.4	47	1.01	0.7	36	1.2%	-0.12 [-0.38, 0.14]
Lee 2010(1)	1.28	0.64	20	1.74	1.03	9	0.4%	-0.46 [-1.19, 0.27]
Lee 2010(2)	1.44	0.83	16	1.74	1.03	9	0.3%	-0.30 [-1.09, 0.49]
Liangmei 2015	0.76	0.38	13	1.27	0.31	12	1.2%	-0.51 [-0.78, -0.24]
Lidan 2012	1.08	0.65	30	1.32	0.33	30	1.2%	-0.24 [-0.50, 0.02]
Lima 2020	0.6	0.25	216	0.67	0.31	109	1.7%	-0.07 [-0.14, -0.00]
Lingling 2021	1.71	0.31	20	2.25	0.32	20	1.4%	-0.54 [-0.74, -0.34]
Liqun 2014	0.99	0.19	76	1.02	0.2	97	1.7%	-0.03 [-0.09, 0.03]
Lira 2017(1)	1.41	0.75	26	1.24	0.54	16	0.9%	0.17 [-0.22, 0.56]
Lira 2017(2)	1.43	0.58	25	1.24	0.54	17	1.0%	0.19 [-0.15, 0.53]
Liu 2018	0.9	0.28	30	1.34	0.28	20	1.5%	-0.44 [-0.60, -0.28]
Lopes 2016	1.22	0.58	17	1.06	0.34	16	1.0%	0.16 [-0.16, 0.48]
Luo 2013(1)	0.53	0.41	43	1.52	0.41	34	1.4%	-0.99 [-1.17, -0.81]
Luo 2013(2)	0.49	0.73	52	1.6	0.42	38	1.3%	-1.11 [-1.35, -0.87]
Machado 2022	1.05	0.44	20	1.97	0.12	15	1.4%	-0.92 [-1.12, -0.72]
Meng 2022(1)	1.4	0.3	12	1.5	0.6	6	0.6%	-0.10 [-0.61, 0.41]
Meng 2022(2)	1.3	0.5	11	1.5	0.6	7	0.6%	-0.20 [-0.73, 0.33]
Meyer 2006	1.04	0.48	33	1.06	0.51	34	1.3%	-0.02 [-0.26, 0.22]
Migueles 2023	1.06	0.38	44	1.1	0.38	42	1.5%	-0.04 [-0.20, 0.12]
Mohammadi 2019	1.01	0.13	10	1.17	0.37	10	1.2%	-0.16 [-0.40, 0.08]
Monteiro 2015(1)	1.37	0.39	18	1.74	0.52	8	0.8%	-0.37 [-0.77, 0.03]
Monteiro 2015(2)	1.33	0.29	14	1.74	0.52	8	0.9%	-0.41 [-0.80, -0.02]
Morgan 2021	1.46	0.12	15	2.05	0.2	15	1.6%	-0.59 [-0.71, -0.47]
Murphy 2009	1.11	0.58	23	1.09	0.42	12	1.0%	0.02 [-0.32, 0.36]
Plavsic 2020	0.9	0.32	22	1	0.43	22	1.3%	-0.10 [-0.32, 0.12]
Racil 2013(1)	1.3	0.05	11	1.23	0.1	6	1.6%	0.07 [-0.02, 0.16]
Racil 2013(2)	1.34	0.09	11	1.23	0.1	6	1.6%	0.11 [0.01, 0.21]
Ramezani 2017(1)	1.9	0.57	15	3.1	0.59	5	0.5%	-1.20 [-1.79, -0.61]
Ramezani 2017(2)	2.18	0.47	15	3.1	0.59	5	0.6%	-0.92 [-1.49, -0.35]
Ramezani 2017(3)	2	0.56	15	3.1	0.59	5	0.5%	-1.10 [-1.69, -0.51]
Salus(2) 2022	1.2	0.37	14	1.69	0.75	14	0.8%	-0.49 [-0.93, -0.05]
Savoye 2007	1	0.37	75	1.13	0.53	44	1.4%	-0.13 [-0.31, 0.05]
Savoye 2011	1.01	0.26	75	1.09	0.41	44	1.5%	-0.08 [-0.21, 0.05]
Saygın 2011	1.59	0.24	20	1.73	0.2	19	1.5%	-0.14 [-0.28, -0.00]
Seo 2012	1.11	1.1	10	1.26	1.76	10	0.1%	-0.15 [-1.44, 1.14]
Seo 2019	1.17	0.02	26	1.1	0.02	44	1.7%	0.07 [0.06, 0.08]
Shalitin 2009	0.96	0.55	55	0.87	0.63	55	1.3%	0.09 [-0.13, 0.31]
Staiano 2017	1.04	0.33	20	0.9	0.39	18	1.3%	0.14 [-0.09, 0.37]
Staiano 2018	0.86	0.48	22	0.94	0.47	23	1.1%	-0.08 [-0.36, 0.20]
Sun 2011	0.9	0.4	25	1.2	0.5	17	1.1%	-0.30 [-0.58, -0.02]
Tas 2023	1.18	0.53	31	1.09	0.53	6	0.7%	0.09 [-0.37, 0.55]
Tjonna 2009	1.19	0.31	20	1.24	0.33	22	1.4%	-0.05 [-0.24, 0.14]
Tsang 2009	1.12	0.35	11	1.35	0.59	8	0.7%	-0.23 [-0.69, 0.23]
Vasconcellos 2016	1.11	0.22	10	1.64	1.03	10	0.5%	-0.53 [-1.18, 0.12]
Wen 2008	0.87	0.29	24	1.3	0.5	13	1.1%	-0.43 [-0.73, -0.13]
Wong 2008	1	0.5	12	1	0.4	12	0.9%	0.00 [-0.36, 0.36]
Woo 2004	1.5	0.7	41	1.2	0.6	41	1.1%	0.30 [0.02, 0.58]
Woo 2012	1.5	0.18	10	1.85	0.27	10	1.4%	-0.35 [-0.55, -0.15]
Xinhui 2016	0.76	0.39	14	1.28	0.32	14	1.2%	-0.52 [-0.78, -0.26]
Xishao 2009	1.08	0.65	34	1.32	0.33	33	1.2%	-0.24 [-0.49, 0.01]
Xishao 2010	1.08	0.45	100	1.32	0.33	100	1.6%	-0.24 [-0.35, -0.13]
Yanqin 2017	1.24	0.09	35	1.31	0.1	33	1.7%	-0.07 [-0.12, -0.02]
Yu 2020	1.2	0.6	99	1.3	0.6	72	1.4%	-0.10 [-0.28, 0.08]
Zehsaz 2016	1.11	0.08	16	1.22	0.08	16	1.7%	-0.11 [-0.17, -0.05]
Zehsaz 2017	0.95	0.22	16	1.08	0.18	16	1.5%	-0.13 [-0.27, 0.01]
**Total (95% CI)**			**2740**			**2125**	**100.0%**	**-0.23 [-0.28, -0.17]**

Heterogeneity: $Tau^2 = 0.04$; $Chi^2 = 1043.99$, $df = 91$ ($P < 0.00001$); $I^2 = 91\%$
Test for overall effect: $Z = 8.54$ ($P < 0.00001$)

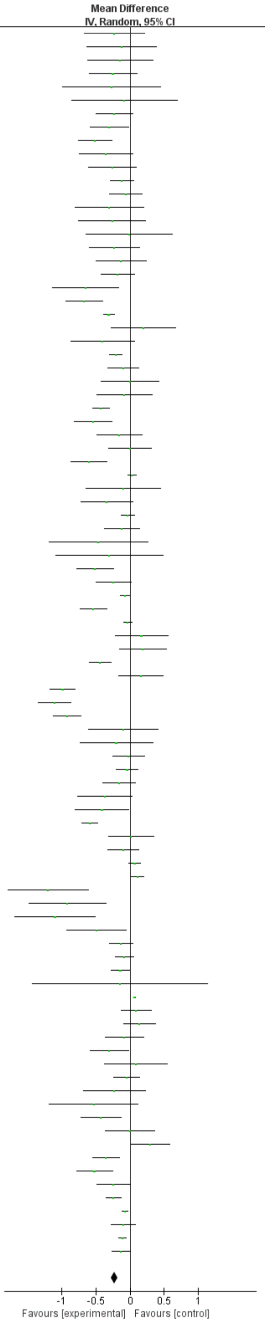

Figure S1.12. Forest plot of the effect of exercise on HDL-C

	Experimental			Control				Mean Difference
Study or Subgroup	Mean	SD	Total	Mean	SD	Total	Weight	IV, Random, 95% CI
Ahmadi 2020(1)	1.13	0.24	28	0.97	0.24	9	1.0%	0.16 [-0.02, 0.34]
Ahmadi 2020(2)	1.12	0.23	29	0.97	0.24	9	1.0%	0.15 [-0.03, 0.33]
Ahmadi 2020(3)	1.1	0.22	29	0.97	0.24	9	1.0%	0.13 [-0.05, 0.31]
Benson 2008	1.5	0.4	32	1.4	0.2	35	1.1%	0.10 [-0.05, 0.25]
Bezerra 2023	1.19	0.31	20	1.12	0.12	21	1.2%	0.07 [-0.08, 0.22]
Boer 2014(1)	1.54	0.29	17	1.45	0.4	7	0.5%	0.09 [-0.24, 0.42]
Boer 2014(2)	1.29	0.27	15	1.45	0.4	7	0.5%	-0.16 [-0.49, 0.17]
Boff 2020	1.3	0.36	34	1.37	0.53	31	0.8%	-0.07 [-0.29, 0.15]
Bowen 2010	1.13	0.19	22	1.11	0.2	18	1.4%	0.02 [-0.10, 0.14]
Bruyndonckx 2015	1.25	0.26	27	1.08	0.27	21	1.2%	0.17 [0.02, 0.32]
Chae 2010	1.37	0.25	19	1.31	0.28	19	1.1%	0.06 [-0.11, 0.23]
Chuensiri 2018(1)	1.19	0.26	11	1.34	0.26	5	0.6%	-0.15 [-0.42, 0.12]
Chuensiri 2018(2)	1.11	0.3	15	1.34	0.26	6	0.7%	-0.23 [-0.49, 0.03]
Davis 2020	1.33	0.42	90	0.9	0.44	85	1.3%	0.43 [0.30, 0.56]
DeBar 2012	1.12	0.22	74	1.15	0.22	42	1.6%	-0.03 [-0.11, 0.05]
Dias 2018(1)	1.24	0.27	19	1.3	0.31	9	0.7%	-0.06 [-0.30, 0.18]
Dias 2018(2)	1.25	0.26	22	1.3	0.31	10	0.8%	-0.05 [-0.27, 0.17]
Elmahgoub 2009	1.46	0.29	15	1.42	0.46	15	0.6%	0.04 [-0.24, 0.32]
Faria 2020(1)	1.34	0.26	24	1.3	0.27	13	1.0%	0.04 [-0.14, 0.22]
Faria 2020(2)	1.32	0.27	26	1.3	0.27	13	1.0%	0.02 [-0.16, 0.20]
Farpour-Lambert 2009	1.14	0.19	22	0.97	0.31	22	1.2%	0.17 [0.02, 0.32]
Ghorbanian 2013	1.17	0.19	15	1.15	0.16	15	1.3%	0.02 [-0.11, 0.15]
Guijun 2009	1.22	0.16	16	0.91	0.1	10	1.5%	0.31 [0.21, 0.41]
Gulijianati 2022	1.66	0.28	47	1.38	0.27	46	1.4%	0.28 [0.17, 0.39]
Guo 2015	1.34	0.27	23	1.11	0.27	12	0.9%	0.23 [0.04, 0.42]
Harder-Lauridsen 2014	1.4	0.4	14	1.6	0.43	14	0.5%	-0.20 [-0.51, 0.11]
Hongling 2012	1.2	0.2	30	1.2	0.3	30	1.3%	0.00 [-0.13, 0.13]
Horner 2015(1)	1.19	0.31	27	1.03	0.19	8	1.0%	0.16 [-0.02, 0.34]
Horner 2015(2)	1.02	0.23	23	1.03	0.19	8	1.1%	-0.01 [-0.17, 0.15]
Jian 2014	1.59	0.45	44	1.44	0.55	44	0.8%	0.15 [-0.06, 0.36]
Jianjun 2012	1.3	0.3	20	1.18	0.18	15	1.1%	0.12 [-0.04, 0.28]
Jun 2006(1)	1.61	0.27	33	1.45	0.32	31	1.2%	0.16 [0.01, 0.31]
Jun 2006(2)	1.61	0.26	35	1.53	0.44	32	1.0%	0.08 [-0.10, 0.26]
Karacabey 2009	1.53	0.19	20	1.17	0.09	20	1.6%	0.36 [0.27, 0.45]
Ke 2024	1.4	0.2	10	1.3	0.3	10	0.8%	0.10 [-0.12, 0.32]
Kelishadi 2008	1.01	0.06	45	1	0.06	47	1.9%	0.01 [-0.01, 0.03]
Kelly 2004	1.1	0.13	10	0.99	0.28	10	0.9%	0.11 [-0.08, 0.30]
Kim 2007	1.12	0.14	14	1.15	0.13	12	1.5%	-0.03 [-0.13, 0.07]
Kim 2022	2.26	1.08	13	1.82	1	13	0.1%	0.44 [-0.36, 1.24]
Lee 2010(1)	1.28	0.24	20	1.15	0.19	9	1.1%	0.13 [-0.03, 0.29]
Lee 2010(2)	1.27	0.26	16	1.15	0.19	9	1.0%	0.12 [-0.06, 0.30]
Liangmei 2015	1.2	0.18	12	1.22	0.18	12	1.2%	-0.02 [-0.16, 0.12]
Lingling 2021	1.07	0.16	20	0.97	0.22	20	1.4%	0.10 [-0.02, 0.22]
Liqun 2014	1.6	0.29	76	1.57	0.21	97	1.7%	0.03 [-0.05, 0.11]
Lira 2017(1)	1.18	0.22	26	1.12	0.26	16	1.2%	0.06 [-0.09, 0.21]
Lira 2017(2)	1.17	0.27	25	1.12	0.26	17	1.1%	0.05 [-0.11, 0.21]
Liu 2018	1.21	0.18	30	1.1	0.4	20	1.0%	0.11 [-0.08, 0.30]
Lopes 2016	1.37	0.32	17	1.55	0.47	16	0.6%	-0.18 [-0.46, 0.10]
Luo 2013(1)	1.15	0.21	43	1.17	0.17	34	1.6%	-0.02 [-0.10, 0.06]
Luo 2013(2)	1.09	0.18	52	1.2	0.16	38	1.7%	-0.11 [-0.18, -0.04]
Machado 2022	1.22	0.22	20	0.91	0.18	15	1.3%	0.31 [0.18, 0.44]
Meng 2022(1)	1.5	0.2	12	1.3	0.2	6	0.9%	0.20 [0.00, 0.40]
Meng 2022(2)	1.4	0.2	11	1.3	0.2	7	0.9%	0.10 [-0.09, 0.29]
Meyer 2006	1.09	0.24	33	1.11	0.31	34	1.3%	-0.02 [-0.15, 0.11]
Migueles 2023	1.33	0.21	42	1.27	0.21	37	1.6%	0.06 [-0.03, 0.15]
Mohammadi 2019	1.1	0.09	10	1.11	0.04	10	1.8%	-0.01 [-0.07, 0.05]
Monteiro 2015(1)	0.98	0.26	18	0.94	0.31	8	0.7%	0.04 [-0.21, 0.29]
Monteiro 2015(2)	1.1	0.32	14	0.94	0.31	8	0.6%	0.16 [-0.11, 0.43]
Morgan 2021	1.3	0.08	15	1.12	0.11	15	1.7%	0.18 [0.11, 0.25]
Murphy 2009	0.93	0.21	23	0.92	0.17	12	1.3%	0.01 [-0.12, 0.14]
Park 2007	1.26	0.16	19	1.26	0.2	21	1.4%	0.00 [-0.11, 0.11]
Plavsic 2020	1.33	0.27	22	1.3	0.26	22	1.1%	0.03 [-0.13, 0.19]
Racil 2013(1)	1.08	0.08	11	1	0.03	6	1.8%	0.08 [0.03, 0.13]
Racil 2013(2)	1.09	0.07	11	1	0.03	6	1.8%	0.09 [0.04, 0.14]
Ramezani 2017(1)	1.26	0.11	15	1.07	0.14	5	1.3%	0.19 [0.06, 0.32]
Ramezani 2017(2)	1.21	0.14	15	1.07	0.14	5	1.2%	0.14 [-0.00, 0.28]
Ramezani 2017(3)	1.19	0.14	15	1.07	0.14	5	1.2%	0.12 [-0.02, 0.26]
Salus(2) 2022	1.07	0.19	14	0.96	0.11	14	1.4%	0.11 [-0.01, 0.23]
Savoye 2007	1.17	0.15	75	1.15	0.45	44	1.3%	0.02 [-0.12, 0.16]
Savoye 2011	1.19	0.18	75	1.12	0.41	44	1.3%	0.07 [-0.06, 0.20]
Saygın 2011	1.36	0.21	20	1.08	0.12	19	1.5%	0.28 [0.17, 0.39]
Seo 2012	0.72	1.15	10	0.95	1.15	10	0.1%	-0.23 [-1.24, 0.78]
Seo 2019	1.25	0.24	26	1.22	0.26	44	1.4%	0.03 [-0.09, 0.15]
Shalitin 2009	1.29	0.27	55	1.24	0.3	55	1.5%	0.05 [-0.06, 0.16]
Staiano 2017	1.26	0.18	20	1.33	0.22	18	1.3%	-0.07 [-0.20, 0.06]
Staiano 2018	1.22	0.19	22	1.31	0.19	23	1.4%	-0.09 [-0.20, 0.02]
Sun 2011	1.1	0.2	25	1.1	0.2	17	1.3%	0.00 [-0.12, 0.12]
Tas 2023	1.04	0.23	31	1.14	0.23	6	0.9%	-0.10 [-0.30, 0.10]
Tjonna 2009	1.24	0.18	20	1.14	0.19	22	1.4%	0.10 [-0.01, 0.21]
Tsang 2009	0.96	0.21	11	0.91	0.23	8	0.9%	0.05 [-0.15, 0.25]
Vasconcellos 2016	1.15	0.13	10	1	0.44	10	0.6%	0.15 [-0.13, 0.43]
Wen 2008	1.31	0.18	24	0.95	0.27	13	1.1%	0.36 [0.20, 0.52]
Wong 2008	1.3	0.3	12	1.2	0.2	12	0.9%	0.10 [-0.10, 0.30]
Woo 2004	1.2	0.3	41	1.2	0.2	41	1.4%	0.00 [-0.11, 0.11]
Xinhui 2016	1.21	0.19	14	1.23	0.19	14	1.2%	-0.02 [-0.16, 0.12]
Yu 2020	1.5	0.3	99	1.3	0.2	72	1.7%	0.20 [0.12, 0.28]
Zehsaz 2016	1.17	0.31	16	1.16	0.26	16	0.9%	0.01 [-0.19, 0.21]
Zehsaz 2017	1.17	0.31	16	1.16	0.26	16	0.9%	0.01 [-0.19, 0.21]
**Total (95% CI)**			**2308**			**1811**	**100.0%**	**0.08 [0.05, 0.10]**

Heterogeneity: Tau² = 0.01; Chi² = 306.87, df = 87 (P < 0.00001); I² = 72%
Test for overall effect: Z = 5.95 (P < 0.00001)

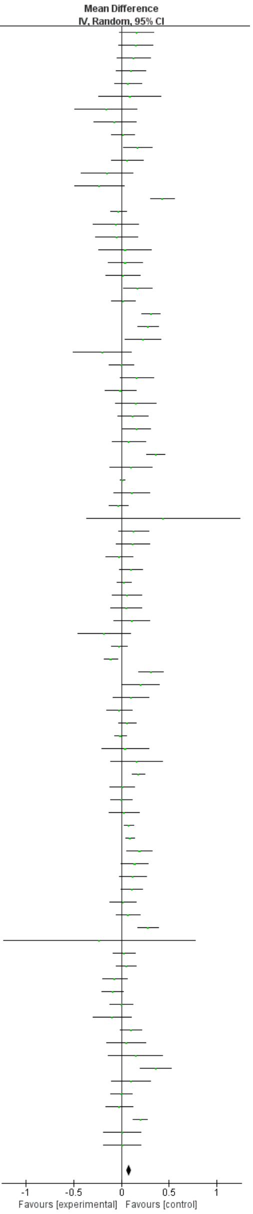

Figure S1.13. Forest plot of the effect of exercise on LDL-C

	Experimental			Control				Mean Difference
Study or Subgroup	Mean	SD	Total	Mean	SD	Total	Weight	IV, Random, 95% CI
Ahmadi 2020(1)	2.37	0.45	28	2.3	0.72	9	1.0%	0.07 [-0.43, 0.57]
Ahmadi 2020(2)	2.1	0.52	29	2.3	0.72	9	0.9%	-0.20 [-0.71, 0.31]
Ahmadi 2020(3)	2.49	0.68	29	2.3	0.72	9	0.9%	0.19 [-0.34, 0.72]
Benson 2008	2.3	0.9	32	2.4	0.6	35	1.2%	-0.10 [-0.47, 0.27]
Bezerra 2023	2.45	0.41	20	2.82	0.62	21	1.3%	-0.37 [-0.69, -0.05]
Boer 2014(1)	2.47	0.24	17	2.48	0.55	7	1.1%	-0.01 [-0.43, 0.41]
Boer 2014(2)	2.52	0.74	15	2.48	0.55	7	0.9%	0.04 [-0.51, 0.59]
Boff 2020	2.25	0.74	34	2.08	0.75	31	1.2%	0.17 [-0.19, 0.53]
Bowen 2010	3	0.68	22	3.29	0.59	18	1.2%	-0.29 [-0.68, 0.10]
Bruyndonckx 2015	1.84	0.33	27	2.24	0.36	21	1.6%	-0.40 [-0.60, -0.20]
Chae 2010	2.33	0.73	19	2.54	0.57	19	1.1%	-0.21 [-0.63, 0.21]
Chuensiri 2018(1)	2.28	0.51	11	2.69	0.51	5	0.9%	-0.41 [-0.95, 0.13]
Chuensiri 2018(2)	2.09	0.5	15	2.69	0.51	6	1.0%	-0.60 [-1.08, -0.12]
Davis 2020	2.44	0.86	90	2.57	0.8	85	1.5%	-0.13 [-0.38, 0.12]
DeBar 2012	2.48	0.69	74	2.45	0.53	42	1.5%	0.03 [-0.19, 0.25]
Dias 2018(1)	2.1	0.5	19	2.36	0.7	9	0.9%	-0.26 [-0.77, 0.25]
Dias 2018(2)	2.55	0.84	22	2.36	0.7	10	0.9%	0.19 [-0.37, 0.75]
Elmahgoub 2009	2.26	0.72	15	2.41	0.54	15	1.0%	-0.15 [-0.61, 0.31]
Faria 2020(1)	1.85	0.49	24	2.07	0.5	13	1.3%	-0.22 [-0.56, 0.12]
Faria 2020(2)	2.03	0.5	26	2.07	0.5	13	1.3%	-0.04 [-0.37, 0.29]
Farpour-Lambert 2009	2.72	0.76	22	2.69	0.55	22	1.2%	0.03 [-0.36, 0.42]
Ghorbanian 2013	3.12	0.57	15	3.88	0.72	15	1.0%	-0.76 [-1.22, -0.30]
Guijun 2009	2.35	0.06	16	3.29	0.25	10	1.6%	-0.94 [-1.10, -0.78]
Gulijianati 2022	2.17	0.29	47	2.98	0.35	46	1.7%	-0.81 [-0.94, -0.68]
Guo 2015	2.74	0.48	23	3.02	0.87	12	0.9%	-0.28 [-0.81, 0.25]
Harder-Lauridsen 2014	2.3	0.75	14	2.6	0.25	14	1.1%	-0.30 [-0.71, 0.11]
Hongling 2012	2.6	0.7	30	2.6	1	30	1.1%	0.00 [-0.44, 0.44]
Horner 2015(1)	2.2	0.71	27	2.27	0.34	8	1.2%	-0.07 [-0.43, 0.29]
Horner 2015(2)	1.86	0.43	23	2.27	0.34	8	1.4%	-0.41 [-0.70, -0.12]
Jian 2014	2.33	0.21	44	2.63	0.24	44	1.7%	-0.30 [-0.39, -0.21]
Jianjun 2012	1.71	0.48	20	2.92	0.67	15	1.1%	-1.21 [-1.61, -0.81]
Jun 2006(1)	1.91	0.41	33	2.14	0.64	31	1.4%	-0.23 [-0.50, 0.04]
Jun 2006(2)	1.8	0.43	35	2.13	1.04	32	1.2%	-0.33 [-0.72, 0.06]
Karacabey 2009	1.75	0.24	20	2.32	0.28	20	1.6%	-0.57 [-0.73, -0.41]
Ke 2024	2.6	0.4	10	3.4	0.7	10	1.0%	-0.80 [-1.30, -0.30]
Kelishadi 2008	3.1	0.36	45	3.16	0.33	47	1.7%	-0.06 [-0.20, 0.08]
Kelly 2004	2.36	0.76	10	2.32	0.73	10	0.7%	0.04 [-0.61, 0.69]
Kim 2007	2.54	0.74	14	2.96	0.85	12	0.8%	-0.42 [-1.04, 0.20]
Kim 2022	2.23	0.71	13	1.85	0.56	13	1.0%	0.38 [-0.11, 0.87]
Lee 2010(1)	2.73	0.42	20	2.63	0.61	9	1.1%	0.10 [-0.34, 0.54]
Lee 2010(2)	2.68	0.71	16	2.63	0.61	9	0.9%	0.05 [-0.48, 0.58]
Liangmei 2015	2.21	0.59	12	2.68	0.74	12	0.9%	-0.47 [-1.01, 0.07]
Lingling 2021	4	1.05	20	4.38	1	20	0.7%	-0.38 [-1.02, 0.26]
Liqun 2014	2.39	0.38	76	2.49	0.4	97	1.7%	-0.10 [-0.22, 0.02]
Lira 2017(1)	2.47	0.93	26	2.89	0.69	16	1.0%	-0.42 [-0.91, 0.07]
Lira 2017(2)	2.48	0.72	25	2.89	0.69	17	1.1%	-0.41 [-0.84, 0.02]
Liu 2018	1.98	0.52	30	2.67	0.59	20	1.3%	-0.69 [-1.01, -0.37]
Lopes 2016	2.41	0.8	17	2.39	0.96	16	0.8%	0.02 [-0.58, 0.62]
Luo 2013(1)	1.93	0.46	43	2.54	0.33	34	1.6%	-0.61 [-0.79, -0.43]
Luo 2013(2)	1.97	0.38	52	2.63	0.33	38	1.7%	-0.66 [-0.81, -0.51]
Machado 2022	2.15	0.6	20	2.13	0.56	15	1.2%	0.02 [-0.37, 0.41]
Meng 2022(1)	1.9	0.3	12	2.2	0.5	6	1.1%	-0.30 [-0.73, 0.13]
Meng 2022(2)	2	0.2	11	2.2	0.5	7	1.2%	-0.20 [-0.59, 0.19]
Meyer 2006	2.57	0.66	33	3.01	0.79	34	1.3%	-0.44 [-0.79, -0.09]
Migueles 2023	2.34	0.39	38	2.52	0.39	33	1.6%	-0.18 [-0.36, 0.00]
Mohammadi 2019	2.52	0.32	10	2.57	0.34	10	1.4%	-0.05 [-0.34, 0.24]
Monteiro 2015(1)	1.3	0.72	18	1.79	0.71	8	0.8%	-0.49 [-1.08, 0.10]
Monteiro 2015(2)	1.05	0.54	14	1.79	0.71	8	0.8%	-0.74 [-1.31, -0.17]
Morgan 2021	2.37	0.24	15	3.12	0.33	15	1.6%	-0.75 [-0.96, -0.54]
Murphy 2009	2.74	0.72	23	2.74	0.43	12	1.2%	0.00 [-0.38, 0.38]
Park 2007	2.35	0.49	19	2.54	0.66	21	1.2%	-0.19 [-0.55, 0.17]
Plavsic 2020	2.28	0.69	22	2.18	0.55	22	1.2%	0.10 [-0.27, 0.47]
Racil 2013(1)	2.18	0.4	11	2.62	0.18	6	1.4%	-0.44 [-0.72, -0.16]
Racil 2013(2)	2.55	0.32	11	2.62	0.18	6	1.5%	-0.07 [-0.31, 0.17]
Ramezani 2017(1)	1.89	1.02	15	3.8	1.17	5	0.3%	-1.91 [-3.06, -0.76]
Ramezani 2017(2)	2.28	0.93	15	3.8	1.17	5	0.3%	-1.52 [-2.65, -0.39]
Ramezani 2017(3)	2.13	1.09	15	3.8	1.17	5	0.3%	-1.67 [-2.83, -0.51]
Salus(2) 2022	2.05	0.37	14	2.6	0.37	14	1.4%	-0.55 [-0.82, -0.28]
Savoye 2007	2.52	0.92	75	2.52	0.68	44	1.4%	0.00 [-0.29, 0.29]
Savoye 2011	2.54	0.75	75	2.53	0.65	44	1.5%	0.01 [-0.25, 0.27]
Saygın 2011	2.4	0.37	20	2.51	0.28	19	1.6%	-0.11 [-0.32, 0.10]
Seo 2012	2.7	2	10	2.34	1.23	10	0.2%	0.36 [-1.10, 1.82]
Seo 2019	2.69	0.64	26	2.9	0.58	44	1.4%	-0.21 [-0.51, 0.09]
Shalitin 2009	2.55	0.67	55	2.53	0.73	55	1.4%	0.02 [-0.24, 0.28]
Staiano 2017	2.34	0.4	20	2.46	0.57	18	1.3%	-0.12 [-0.44, 0.20]
Staiano 2018	2.19	0.8	22	2.76	0.74	23	1.0%	-0.57 [-1.02, -0.12]
Sun 2011	2.3	0.5	25	2.4	0.5	17	1.3%	-0.10 [-0.41, 0.21]
Tas 2023	2.37	0.55	31	2.41	0.55	6	1.0%	-0.04 [-0.52, 0.44]
Tsang 2009	2.62	0.75	11	2.33	0.57	8	0.8%	0.29 [-0.30, 0.88]
Vasconcellos 2016	2.61	0.33	10	3.03	0.82	10	0.9%	-0.42 [-0.97, 0.13]
Wen 2008	2.21	0.47	24	3.29	0.87	13	0.9%	-1.08 [-1.59, -0.57]
Wong 2008	2.7	0.9	12	2.8	0.7	12	0.7%	-0.10 [-0.75, 0.55]
Woo 2004	2.6	0.8	41	2.7	0.7	41	1.3%	-0.10 [-0.43, 0.23]
Xianbo 2011	2.3	0.5	25	2.4	0.5	17	1.3%	-0.10 [-0.41, 0.21]
Xinhui 2016	2.21	0.61	14	2.68	0.75	14	0.9%	-0.47 [-0.98, 0.04]
Zehsaz 2016	1.87	0.25	16	2.24	0.19	16	1.7%	-0.37 [-0.52, -0.22]
Zehsaz 2017	2.54	0.92	16	2.47	0.87	16	0.8%	0.07 [-0.55, 0.69]
**Total (95% CI)**			**2210**			**1730**	**100.0%**	**-0.28 [-0.35, -0.20]**

Heterogeneity: Tau² = 0.07; Chi² = 383.87, df = 86 (P < 0.00001); I² = 78%
Test for overall effect: Z = 7.58 (P < 0.00001)

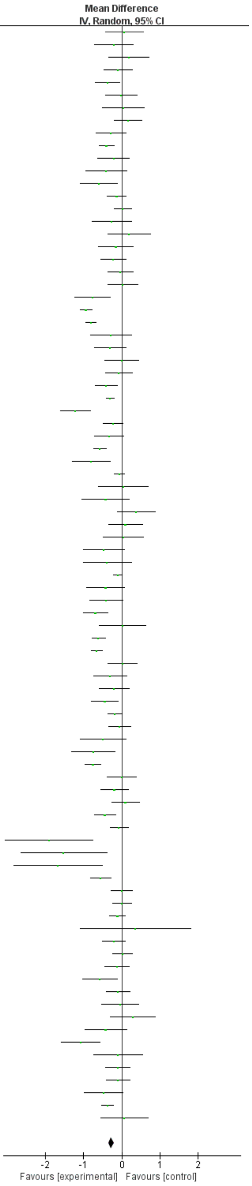

Figure S1.14. Forest plot of the effect of exercise on FPG

Study or Subgroup	Experimental Mean	Experimental SD	Experimental Total	Control Mean	Control SD	Control Total	Weight	Mean Difference IV, Random, 95% CI
Abassi 2020(1)	4.88	0.4	8	5.2	0.21	4	1.0%	-0.32 [-0.67, 0.03]
Abassi 2020(2)	5	0.39	8	5.2	0.21	4	1.0%	-0.20 [-0.54, 0.14]
Benson 2008	5.2	0.4	32	5	0.5	35	1.2%	0.20 [-0.02, 0.42]
Bezerra 2023	4.7	0.34	20	4.64	0.38	21	1.2%	0.06 [-0.16, 0.28]
Bharath 2018	4.3	0.89	20	5.4	0.44	20	0.9%	-1.10 [-1.54, -0.66]
Boer 2014(1)	4.72	0.39	17	4.72	0.23	7	1.2%	0.00 [-0.25, 0.25]
Boer 2014(2)	4.72	0.28	15	4.72	0.23	7	1.2%	0.00 [-0.22, 0.22]
Bowen 2010	4.79	0.38	22	4.64	0.41	18	1.2%	0.15 [-0.10, 0.40]
Carrel 2005	4.83	0.28	27	5.16	0.39	23	1.3%	-0.33 [-0.52, -0.14]
Davis(1) 2009	4.91	0.48	17	5.07	0.36	21	1.1%	-0.16 [-0.44, 0.12]
DAVIS(2) 2009(1)	4.68	0.37	9	5.11	0.22	5	1.1%	-0.43 [-0.74, -0.12]
DAVIS(2) 2009(2)	4.82	0.34	15	5.11	0.22	5	1.2%	-0.29 [-0.55, -0.03]
Davis 2020	5.59	0.48	90	5.61	0.6	85	1.3%	-0.02 [-0.18, 0.14]
DeBar 2012	4.63	0.46	74	4.59	0.49	42	1.3%	0.04 [-0.14, 0.22]
Dias 2018(1)	5.13	0.37	19	5.08	0.39	8	1.1%	0.05 [-0.27, 0.37]
Dias 2018(2)	5.19	0.37	22	5.08	0.39	9	1.1%	0.11 [-0.19, 0.41]
Duft 2020	4.55	0.41	18	4.75	0.47	19	1.1%	-0.20 [-0.48, 0.08]
Faria 2020(1)	4.65	0.38	24	4.71	0.39	13	1.1%	-0.06 [-0.32, 0.20]
Faria 2020(2)	4.7	0.39	26	4.71	0.39	13	1.2%	-0.01 [-0.27, 0.25]
Farpour-Lambert 2009	4.95	0.26	22	4.79	0.31	22	1.3%	0.16 [-0.01, 0.33]
Ghorbanian 2013	5.42	0.52	15	5.34	0.53	15	1.0%	0.08 [-0.30, 0.46]
Gulijianati 2022	4.83	0.36	47	5.46	0.41	46	1.3%	-0.63 [-0.79, -0.47]
Guo 2015	4.92	0.4	23	5.06	0.59	12	1.0%	-0.14 [-0.51, 0.23]
Harder-Lauridsen 2014	4.5	0.16	14	4.4	0.4	14	1.2%	0.10 [-0.13, 0.33]
Hongling 2012	4.1	0.8	30	4.2	0.4	30	1.1%	-0.10 [-0.42, 0.22]
Jiafen 2017	6.11	0.62	78	7.82	0.84	78	1.2%	-1.71 [-1.94, -1.48]
Jianjun 2012	3.78	0.79	20	5.62	0.74	15	0.8%	-1.84 [-2.35, -1.33]
Jun 2006(1)	3.92	0.45	33	4.18	0.46	31	1.2%	-0.26 [-0.48, -0.04]
Jun 2006(2)	3.97	0.27	35	4.03	0.37	32	1.3%	-0.06 [-0.22, 0.10]
Ke 2024	5.2	0.4	10	5.6	0.6	10	0.9%	-0.40 [-0.85, 0.05]
Kelishadi 2008	4.51	0.46	45	4.5	0.44	47	1.3%	0.01 [-0.17, 0.19]
Kelly 2004	4.66	0.25	10	4.8	0.47	10	1.0%	-0.14 [-0.47, 0.19]
Kelly 2019	5.36	0.29	13	5.23	0.29	13	1.2%	0.13 [-0.09, 0.35]
Kim 2007	4.72	0.33	14	4.72	0.37	12	1.1%	0.00 [-0.27, 0.27]
Kim 2020	4.82	0.12	24	5.22	0.15	24	1.4%	-0.40 [-0.48, -0.32]
Kim 2022	5	0.82	13	4.45	0.24	13	0.8%	0.55 [0.09, 1.01]
Lee 2010(1)	4.55	0.49	20	5.11	0.51	9	0.9%	-0.56 [-0.96, -0.16]
Lee 2010(2)	4.95	0.33	16	5.11	0.51	9	1.0%	-0.16 [-0.53, 0.21]
Lee 2012(1)	5.31	0.22	15	5.37	0.27	5	1.1%	-0.06 [-0.32, 0.20]
Lee 2012(2)	5.27	0.23	16	5.37	0.27	6	1.2%	-0.10 [-0.34, 0.14]
Lee 2013(1)	5.04	0.61	14	5.47	0.21	4	1.0%	-0.43 [-0.81, -0.05]
Lee 2013(2)	5.26	0.6	14	5.47	0.21	4	1.0%	-0.21 [-0.59, 0.17]
Lidan 2012	4.18	0.72	30	4.78	1.01	30	0.9%	-0.60 [-1.04, -0.16]
Lima 2020	4.58	0.34	198	4.71	0.38	100	1.4%	-0.13 [-0.22, -0.04]
Lira 2017(1)	4.54	0.53	26	4.4	0.3	16	1.2%	0.14 [-0.11, 0.39]
Lira 2017(2)	4.63	0.53	25	4.4	0.3	17	1.2%	0.23 [-0.02, 0.48]
Liu 2018	4.32	0.3	30	4.71	0.39	20	1.2%	-0.39 [-0.59, -0.19]
Lopes 2016	4.52	0.34	17	4.88	0.31	16	1.2%	-0.36 [-0.58, -0.14]
Luo 2013(1)	3.76	0.71	43	4.08	0.6	34	1.1%	-0.32 [-0.61, -0.03]
Luo 2013(2)	4.19	0.72	52	4.25	0.63	38	1.1%	-0.06 [-0.34, 0.22]
Machado 2022	5.06	0.29	20	5.39	0.51	15	1.1%	-0.33 [-0.62, -0.04]
McCormack 2014	4.38	0.18	10	4.24	0.27	8	1.2%	0.14 [-0.08, 0.36]
Meng 2022(1)	4.7	0.3	12	5.4	0.4	6	1.0%	-0.70 [-1.06, -0.34]
Meng 2022(2)	4.9	0.4	11	5.4	0.4	7	1.0%	-0.50 [-0.88, -0.12]
Migueles 2023	4.66	0.45	46	4.65	0.45	42	1.3%	0.01 [-0.18, 0.20]
Mohammadi 2019	4.3	0.29	10	4.57	0.37	10	1.1%	-0.27 [-0.56, 0.02]
Murphy 2009	5.07	0.24	23	5.08	0.16	12	1.3%	-0.01 [-0.14, 0.12]
Park 2007	4.77	0.52	19	5.28	0.63	21	1.0%	-0.51 [-0.87, -0.15]
Pena 2022	5.49	0.49	79	5.88	1.37	38	0.9%	-0.39 [-0.84, 0.06]
Racil(1) 2016(1)	4.6	0.4	17	4.6	0.4	7	1.0%	0.00 [-0.35, 0.35]
Racil(1) 2016(2)	4.7	0.2	16	4.6	0.4	7	1.1%	0.10 [-0.21, 0.41]
Racil(2) 2016(1)	4.6	0.4	23	4.8	0.4	9	1.1%	-0.20 [-0.51, 0.11]
Racil(2) 2016(2)	4.1	0.4	26	4.8	0.4	10	1.1%	-0.70 [-0.99, -0.41]
Racil 2013(1)	4.5	0.47	11	4.6	0.32	6	1.0%	-0.10 [-0.48, 0.28]
Racil 2013(2)	4.4	0.34	11	4.6	0.32	6	1.0%	-0.20 [-0.53, 0.13]
Racil 2024(1)	4.35	0.4	12	4.3	0.4	5	0.9%	0.05 [-0.37, 0.47]
Racil 2024(2)	4.05	0.4	12	4.3	0.4	6	0.9%	-0.25 [-0.64, 0.14]
Ramezani 2017(1)	4.89	0.61	15	5.86	0.92	5	0.4%	-0.97 [-1.83, -0.11]
Ramezani 2017(2)	5.26	0.77	15	5.86	0.92	5	0.4%	-0.60 [-1.50, 0.30]
Ramezani 2017(3)	5.06	0.63	15	5.86	0.92	5	0.4%	-0.80 [-1.67, 0.07]
Salus(2) 2022	5.11	0.37	14	5.19	0.37	14	1.1%	-0.08 [-0.35, 0.19]
Savoye 2007	4.92	0.36	75	4.9	0.48	44	1.3%	0.02 [-0.14, 0.18]
Savoye 2011	4.97	0.27	75	4.88	0.3	44	1.3%	0.09 [-0.02, 0.20]
Seo 2012	4.66	0.77	10	4.75	0.68	10	0.6%	-0.09 [-0.73, 0.55]
Shaibi 2006	5.21	0.35	11	5.09	0.35	10	1.1%	0.12 [-0.18, 0.42]
Shalitin 2009	4.49	0.4	55	4.55	0.45	55	1.3%	-0.06 [-0.22, 0.10]
Son 2017	4.82	0.52	20	5.22	0.67	20	1.0%	-0.40 [-0.77, -0.03]
Staiano 2017	4.95	1.76	20	5.74	1.78	18	0.3%	-0.79 [-1.92, 0.34]
Staiano 2018	5.01	0.3	22	4.95	0.25	23	1.3%	0.06 [-0.10, 0.22]
Tas 2023	5.09	0.49	31	5.05	0.49	6	0.9%	0.04 [-0.39, 0.47]
Tjonna 2009	4.9	0.31	20	5	0.33	22	1.2%	-0.10 [-0.29, 0.09]
Tsang 2009	5.15	0.48	11	4.83	0.39	8	0.9%	0.32 [-0.07, 0.71]
Vasconcellos 2016	5.1	0.36	10	5.23	0.46	10	1.0%	-0.13 [-0.49, 0.23]
Wen 2008	3.97	0.31	24	5.23	0.69	13	0.9%	-1.26 [-1.66, -0.86]
Wong 2008	4.7	0.4	12	4.8	0.8	12	0.8%	-0.10 [-0.61, 0.41]
Wong 2018	4.3	0.77	15	5.4	0.39	15	0.9%	-1.10 [-1.54, -0.66]
Woo 2004	4.4	0.6	41	4.3	0.6	41	1.2%	0.10 [-0.16, 0.36]
Xianbo 2011	4.8	0.4	25	4.6	0.4	17	1.2%	0.20 [-0.05, 0.45]
Xishao 2009	4.18	0.72	34	4.78	1.01	33	0.9%	-0.60 [-1.02, -0.18]
Xishao 2010	4.18	0.72	100	4.78	1.01	100	1.2%	-0.60 [-0.84, -0.36]
Yu 2020	4.6	0.5	99	5.1	0.4	72	1.3%	-0.50 [-0.64, -0.36]
Zehsaz 2016	4.8	0.5	16	4.8	0.5	16	1.0%	0.00 [-0.35, 0.35]
Zehsaz 2017	4.8	0.5	16	4.8	0.5	16	1.0%	0.00 [-0.35, 0.35]
Zhaogang 2021	4.03	0.28	49	4.25	0.54	49	1.3%	-0.22 [-0.39, -0.05]
**Total (95% CI)**			**2653**			**1989**	**100.0%**	**-0.20 [-0.26, -0.13]**

Heterogeneity: $Tau^2 = 0.08$; $Chi^2 = 637.17$, df = 93 (P < 0.00001); $I^2 = 85\%$
Test for overall effect: Z = 5.81 (P < 0.00001)

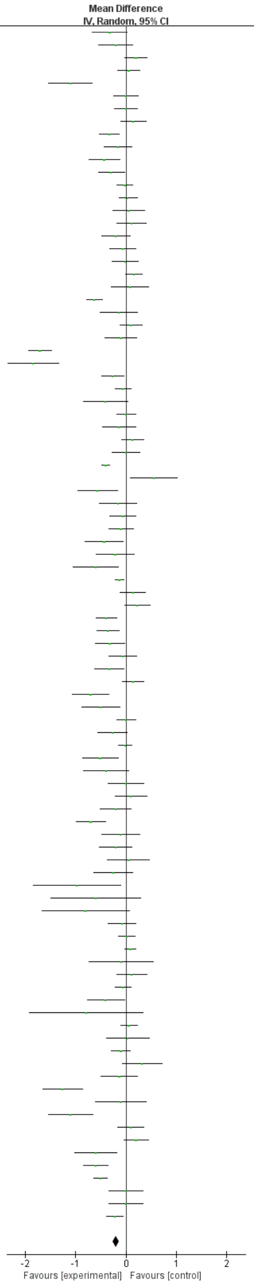

Figure S1.15. Forest plot of the effect of exercise on HbA1c

Study or Subgroup	Experimental Mean	Experimental SD	Experimental Total	Control Mean	Control SD	Control Total	Weight	Mean Difference IV, Fixed, 95% CI
Boff 2020	4.98	0.33	34	5	0.33	31	7.6%	-0.02 [-0.18, 0.14]
Dias 2018(1)	5.35	0.3	18	5.37	0.23	9	4.7%	-0.02 [-0.22, 0.18]
Dias 2018(2)	5.17	0.22	22	5.37	0.23	9	6.3%	-0.20 [-0.38, -0.02]
Faria 2020(1)	5.3	0.24	24	5.4	0.25	13	7.1%	-0.10 [-0.27, 0.07]
Faria 2020(2)	5.3	0.25	26	5.4	0.25	13	7.1%	-0.10 [-0.27, 0.07]
Harder-Lauridsen 2014	5.3	0.25	14	5.32	0.27	12	4.8%	-0.02 [-0.22, 0.18]
Meyer 2006	5.3	0.38	33	5.2	0.46	34	4.8%	0.10 [-0.10, 0.30]
Naylor 2016	9.2	2.83	8	6.5	0.45	5	0.0%	2.70 [0.70, 4.70]
Park 2007	5.3	0.3	19	5.4	0.3	21	5.7%	-0.10 [-0.29, 0.09]
Pena 2022	5.6	0.27	79	5.7	0.62	38	4.6%	-0.10 [-0.31, 0.11]
Plavsic 2020	5.03	0.24	22	5.06	0.22	22	10.6%	-0.03 [-0.17, 0.11]
Tjonna 2009	5.63	0.13	20	5.63	0.14	22	29.4%	0.00 [-0.08, 0.08]
Tsang 2009	5.31	0.35	11	5.38	0.13	8	3.8%	-0.07 [-0.30, 0.16]
Williams 2024	5.4	0.3	10	5.5	0.25	11	3.5%	-0.10 [-0.34, 0.14]
**Total (95% CI)**			**340**			**248**	**100.0%**	**-0.04 [-0.09, 0.00]**

Heterogeneity: $Chi^2 = 15.31$, df = 13 (P = 0.29); $I^2 = 15\%$
Test for overall effect: Z = 1.93 (P = 0.05)

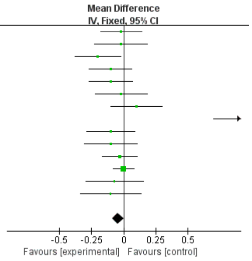

Figure S1.16. Forest plot of the effect of exercise on FINS

Study or Subgroup	Experimental Mean	Experimental SD	Experimental Total	Control Mean	Control SD	Control Total	Weight	Mean Difference IV, Random, 95% CI
Abassi 2020(1)	9.46	2.08	8	12.3	5.31	4	1.5%	-2.84 [-8.24, 2.56]
Abassi 2020(2)	10.6	4.1	8	12.3	5.31	4	1.4%	-1.70 [-7.63, 4.23]
Benson 2008	15.33	9.6	32	16.03	20.85	35	1.0%	-0.70 [-8.37, 6.97]
Bharath 2018	10.3	9.39	20	27	10.73	20	1.3%	-16.70 [-22.95, -10.45]
Bowen 2010	15.05	4.7	22	16.29	12.2	18	1.4%	-1.24 [-7.21, 4.73]
Carrel 2005	21.9	14	27	25.8	10	23	1.2%	-3.90 [-10.58, 2.78]
Davis(1) 2009	27.8	12.5	17	24.3	14.5	21	0.8%	3.50 [-5.09, 12.09]
DAVIS(2) 2009(1)	28.3	8.01	9	28	17.5	5	0.3%	0.30 [-15.91, 16.51]
DAVIS(2) 2009(2)	28.6	7.49	15	28	17.5	5	0.3%	0.60 [-15.20, 16.40]
Davis 2020	23.4	15.67	90	19.4	16.08	85	1.8%	4.00 [-0.71, 8.71]
Faria 2020(1)	10.8	4.14	24	11	4.21	13	2.7%	-0.20 [-3.03, 2.63]
Faria 2020(2)	11.4	4.33	26	11	4.21	13	2.7%	0.40 [-2.43, 3.23]
Farpour-Lambert 2009	16.13	6.77	22	13.5	5.27	22	2.3%	2.63 [-0.96, 6.22]
Gulijianati 2022	5.18	1.23	47	7.89	1.29	46	3.7%	-2.71 [-3.22, -2.20]
Jianjun 2012	13.34	4.53	20	28.92	6.44	15	2.2%	-15.58 [-19.40, -11.76]
Kelly 2004	11.12	5	10	14.12	7.43	10	1.5%	-3.00 [-8.55, 2.55]
Kim 2007	8.9	4.98	14	10.8	4.5	12	2.3%	-1.90 [-5.55, 1.75]
Kim 2020	9.1	1.2	24	13.7	2.2	24	3.6%	-4.60 [-5.60, -3.60]
Lee 2012(1)	20.1	69.38	15	40	69.05	5	0.0%	-19.90 [-89.87, 50.07]
Lee 2012(2)	17	66.94	16	40	69.05	6	0.0%	-23.00 [-87.25, 41.25]
Lee 2013(1)	20.57	14.71	14	31.05	13.23	4	0.3%	-10.48 [-25.56, 4.60]
Lee 2013(2)	44.15	17.49	14	31.05	13.23	4	0.3%	13.10 [-2.78, 28.98]
Lira 2017(1)	27.75	15.35	26	27.4	12.57	16	0.8%	0.35 [-8.18, 8.88]
Lira 2017(2)	25.48	10.3	25	27.4	12.57	17	1.1%	-1.92 [-9.13, 5.29]
Liu 2018	8.95	4.39	30	16.78	4.85	20	2.8%	-7.83 [-10.47, -5.19]
Lopes 2016	14.7	7.4	17	16.8	6.5	16	1.8%	-2.10 [-6.85, 2.65]
Luo 2013(1)	6.22	7.37	43	12.83	5.11	34	2.7%	-6.61 [-9.40, -3.82]
Luo 2013(2)	8.73	7.97	52	16.27	5.27	38	2.8%	-7.54 [-10.28, -4.80]
Machado 2022	14.7	7.6	20	29.9	6	15	1.9%	-15.20 [-19.71, -10.69]
McCormack 2014	27.67	21.32	10	21.17	17.33	8	0.2%	6.50 [-11.36, 24.36]
Meng 2022(1)	7.5	2.8	12	12.3	2.4	6	2.9%	-4.80 [-7.29, -2.31]
Meng 2022(2)	6.8	3	11	12.3	2.4	7	2.9%	-5.50 [-8.01, -2.99]
Meyer 2006	11.16	4.61	33	14.6	7.65	34	2.6%	-3.44 [-6.45, -0.43]
Migueles 2023	13.36	6.32	39	13.91	6.33	37	2.7%	-0.55 [-3.40, 2.30]
Park 2007	8.3	3	19	14.4	8.2	21	2.2%	-6.10 [-9.86, -2.34]
Pena 2022	19	8.83	78	21	12.33	38	1.9%	-2.00 [-6.38, 2.38]
Racil(1) 2016(1)	16.3	0.84	17	19.2	2	7	3.4%	-2.90 [-4.43, -1.37]
Racil(1) 2016(2)	17.3	1.5	16	19.2	2	7	3.3%	-1.90 [-3.55, -0.25]
Racil(2) 2016(1)	15.6	1.2	23	18.6	1.6	9	3.6%	-3.00 [-4.15, -1.85]
Racil(2) 2016(2)	14.6	1	26	18.6	1.6	10	3.6%	-4.00 [-5.06, -2.94]
Racil 2013(1)	15.5	1.19	11	18.8	1.95	6	3.3%	-3.30 [-5.01, -1.59]
Racil 2013(2)	17.9	1.45	11	18.8	1.95	6	3.3%	-0.90 [-2.68, 0.88]
Salus(2) 2022	20.64	6.73	14	31.8	24.69	14	0.4%	-11.16 [-24.57, 2.25]
Savoye 2007	17.9	27.11	75	29.5	16.2	44	0.9%	-11.60 [-19.38, -3.82]
Savoye 2011	18.4	4.72	75	27.4	17.73	44	1.6%	-9.00 [-14.35, -3.65]
Seo 2012	6.63	8.66	10	9.93	25.11	10	0.3%	-3.30 [-19.76, 13.16]
Shaibi 2006	11.5	8.29	11	19.1	8.54	10	1.1%	-7.60 [-14.81, -0.39]
Shalitin 2009	8.88	9.12	55	11.62	10.01	55	2.3%	-2.74 [-6.32, 0.84]
Son 2017	9.1	1.34	20	12.5	5.37	20	2.9%	-3.40 [-5.83, -0.97]
Staiano 2017	26.1	13.37	20	23.47	7.63	18	1.1%	2.63 [-4.21, 9.47]
Tas 2023	35.85	22.41	31	28.86	22.41	6	0.2%	6.99 [-12.60, 26.58]
Tjonna 2009	22.02	12.82	20	23.33	13.6	22	0.9%	-1.31 [-9.30, 6.68]
Tsang 2009	19.95	12.57	11	20.89	5	8	0.9%	-0.94 [-9.14, 7.26]
Vasconcellos 2016	31.5	9.2	10	37.1	18.6	10	0.4%	-5.60 [-18.46, 7.26]
Wen 2008	22.08	12.64	24	51.23	26.09	13	0.3%	-29.15 [-44.21, -14.09]
Wong 2018	10.3	8.13	15	27	9.3	15	1.3%	-16.70 [-22.95, -10.45]
Zehsaz 2016	12.3	2.6	16	16.6	3.1	16	3.2%	-4.30 [-6.28, -2.32]
**Total (95% CI)**			**1420**			**1051**	**100.0%**	**-3.93 [-4.80, -3.06]**

Heterogeneity: $Tau^2 = 5.23$; $Chi^2 = 233.37$, df = 56 (P < 0.00001); $I^2 = 76\%$
Test for overall effect: Z = 8.83 (P < 0.00001)

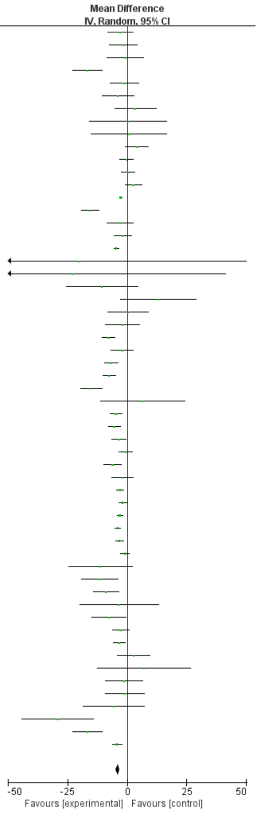

Figure S1.17. Forest plot of the effect of exercise on Depression

	Experimental			Control				Std. Mean Difference
Study or Subgroup	Mean	SD	Total	Mean	SD	Total	Weight	IV, Fixed, 95% CI
Brennan 2012	7.9	9.6	40	4.2	5.9	21	3.8%	0.43 [-0.11, 0.96]
CROKER 2012	49.24	6.91	33	48.13	6.97	30	4.5%	0.16 [-0.34, 0.65]
Daley 2006(1)	7.68	5.56	27	8.23	6.12	13	2.5%	-0.09 [-0.76, 0.57]
Daley 2006(2)	8.32	6	22	8.23	6.12	13	2.3%	0.01 [-0.67, 0.70]
Danielsen 2013	42.73	4.64	22	46.5	9.63	20	2.9%	-0.50 [-1.11, 0.12]
Goldfield 2015(1)	2.1	3.42	73	2.7	3.46	25	5.3%	-0.17 [-0.63, 0.28]
Goldfield 2015(2)	1.7	3.53	78	2.7	3.46	25	5.4%	-0.28 [-0.73, 0.17]
Goldfield 2015(3)	2.6	3.39	72	2.7	3.46	25	5.3%	-0.03 [-0.48, 0.43]
Heidarianpour 2023	12.45	2.19	15	14.14	3.65	15	2.1%	-0.55 [-1.28, 0.18]
Jelalian 2019	9.3	12.7	21	7	5.9	9	1.8%	0.20 [-0.58, 0.98]
Lofrano-Prado 2022	13.9	8.3	37	11.4	8.3	37	5.2%	0.30 [-0.16, 0.76]
Migueles 2023	7.31	4.59	44	7.31	4.59	44	6.3%	0.00 [-0.42, 0.42]
Moon 2004	1.7	0.5	34	1.7	0.5	28	4.4%	0.00 [-0.50, 0.50]
Petty 2009(1)	51.5	10.7	42	48.9	9.8	19	3.7%	0.25 [-0.30, 0.79]
Petty 2009(2)	48	9.5	42	48.9	9.8	19	3.7%	-0.09 [-0.63, 0.45]
Petty 2009(3)	47.5	9.9	27	51.7	11.2	15	2.7%	-0.40 [-1.03, 0.24]
Petty 2009(4)	45.4	6.5	28	51.7	11.2	15	2.6%	-0.74 [-1.38, -0.09]
Romero-Perez 2020	39.59	11.04	54	40.06	11.44	51	7.5%	-0.04 [-0.42, 0.34]
Wagener 2012	45.8	8.6	17	51	11.4	16	2.3%	-0.50 [-1.20, 0.19]
Weintraub 2008	2.78	1.72	9	3.92	3.32	12	1.4%	-0.40 [-1.27, 0.48]
Williams 2019	6.3	5.2	90	6.8	5.9	85	12.5%	-0.09 [-0.39, 0.21]
Yu 2020	16	16.2	99	13	18	72	11.9%	0.18 [-0.13, 0.48]
**Total (95% CI)**			**926**			**609**	**100.0%**	**-0.05 [-0.15, 0.06]**

Heterogeneity: Chi^2 = 22.65, df = 21 (P = 0.36); I^2 = 7%
Test for overall effect: Z = 0.85 (P = 0.39)

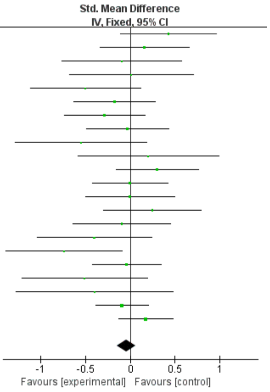

Figure S1.18. Forest plot of the effect of exercise on Anxiety

	Experimental			Control				Std. Mean Difference
Study or Subgroup	Mean	SD	Total	Mean	SD	Total	Weight	IV, Random, 95% CI
Brennan 2012	7	7.4	40	6.8	6	21	17.4%	0.03 [-0.50, 0.56]
Heidarianpour 2023	47.77	2.6	15	60.26	3.8	15	10.0%	-3.73 [-4.97, -2.49]
Migueles 2023	32.04	6.26	45	30.21	6.23	39	18.4%	0.29 [-0.14, 0.72]
Romero-Perez 2020	18.65	5.71	54	19.35	5.34	51	18.9%	-0.13 [-0.51, 0.26]
Wagener 2012	47.9	9.2	16	47.35	10.4	18	15.8%	0.05 [-0.62, 0.73]
Yu 2020	20	20.2	99	20	27.8	72	19.6%	0.00 [-0.30, 0.30]
**Total (95% CI)**			**269**			**216**	**100.0%**	**-0.33 [-0.87, 0.21]**

Heterogeneity: Tau^2 = 0.37; Chi^2 = 36.67, df = 5 (P < 0.00001); I^2 = 86%
Test for overall effect: Z = 1.19 (P = 0.23)

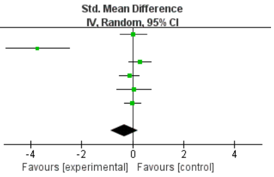

Figure S1.19. Forest plot of the effect of exercise on Self-esteem

	Experimental			Control				Std. Mean Difference
Study or Subgroup	Mean	SD	Total	Mean	SD	Total	Weight	IV, Fixed, 95% CI
Brennan 2012	20.6	6.3	42	19.2	4.7	21	7.3%	0.24 [-0.29, 0.76]
CROKER 2012	2.8	0.51	33	2.85	0.51	30	8.2%	-0.10 [-0.59, 0.40]
DeBar 2012	2.4	0.25	104	2.39	0.25	102	26.8%	0.04 [-0.23, 0.31]
Goldfield 2015(1)	17.2	4.84	65	16.3	5.02	23	8.8%	0.18 [-0.29, 0.66]
Goldfield 2015(2)	17.5	5.16	74	16.3	5.02	23	9.1%	0.23 [-0.24, 0.70]
Goldfield 2015(3)	16.6	4.84	65	16.3	5.02	24	9.1%	0.06 [-0.41, 0.53]
Lee 2016	3.26	0.47	15	2.58	0.77	15	3.4%	1.04 [0.27, 1.81]
Migueles 2023	35.17	4.02	47	34.8	4	40	11.3%	0.09 [-0.33, 0.51]
Schranz 2014	2.9	0.54	26	2.43	0.55	23	5.8%	0.85 [0.26, 1.44]
Staiano 2013(1)	24.08	3.88	13	20.45	5.82	5	1.7%	0.78 [-0.29, 1.85]
Staiano 2013(2)	22.33	5.74	9	20.45	5.82	6	1.8%	0.31 [-0.73, 1.35]
Wagener 2012	50.6	10.3	16	46.3	12.8	15	4.0%	0.36 [-0.35, 1.07]
Weintraub 2008	19.33	3.81	9	20.5	5.42	12	2.7%	-0.23 [-1.10, 0.63]
**Total (95% CI)**			**518**			**339**	**100.0%**	**0.18 [0.04, 0.33]**

Heterogeneity: Chi^2 = 14.85, df = 12 (P = 0.25); I^2 = 19%
Test for overall effect: Z = 2.56 (P = 0.01)

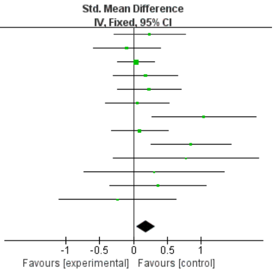

Figure S1.20. Forest plot of the effect of exercise on Self-worth

	Experimental			Control				Std. Mean Difference
Study or Subgroup	Mean	SD	Total	Mean	SD	Total	Weight	IV, Fixed, 95% CI
Daley 2006(1)	2.14	0.36	27	1.94	0.25	13	4.7%	0.60 [-0.08, 1.27]
Daley 2006(2)	2.14	0.33	22	1.94	0.25	13	4.3%	0.64 [-0.06, 1.35]
Danielsen 2013	3.26	0.52	22	2.98	0.63	20	5.7%	0.48 [-0.14, 1.09]
Goldfield 2015(1)	13.3	4.03	65	13	4.18	23	9.5%	0.07 [-0.40, 0.55]
Goldfield 2015(2)	14.1	5.16	74	13	4.18	23	9.8%	0.22 [-0.25, 0.69]
Goldfield 2015(3)	13.5	4.03	65	13	4.18	24	9.8%	0.12 [-0.35, 0.59]
Petty 2009(1)	3.2	0.62	42	3.1	0.7	19	7.3%	0.15 [-0.39, 0.70]
Petty 2009(2)	3.1	0.7	42	3.1	0.7	19	7.3%	0.00 [-0.54, 0.54]
Petty 2009(3)	2.9	0.8	27	2.9	0.6	15	5.4%	0.00 [-0.63, 0.63]
Petty 2009(4)	3.4	0.5	28	2.9	0.6	15	5.0%	0.92 [0.26, 1.57]
Schranz 2014	2.51	0.66	26	2.29	0.57	23	6.7%	0.35 [-0.22, 0.92]
Williams 2019	3.4	0.63	90	3.3	0.53	85	24.4%	0.17 [-0.13, 0.47]
**Total (95% CI)**			**530**			**292**	**100.0%**	**0.25 [0.10, 0.39]**

Heterogeneity: Chi^2 = 9.41, df = 11 (P = 0.58); I^2 = 0%
Test for overall effect: Z = 3.28 (P = 0.001)

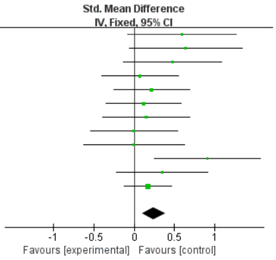

Figure S2. Forest plots of subgroup analyses for BMI in children and adolescents with overweight or obesity

Figure S2.1. Forest plot of subgroup analyses by type of exercise

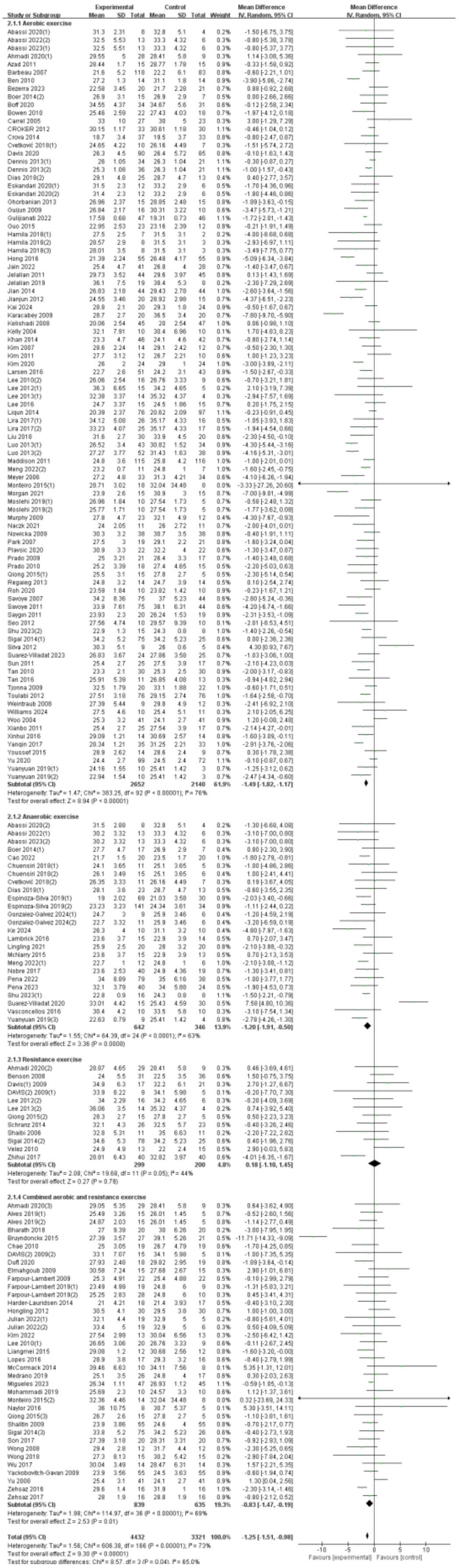

Figure S2.2. Forest plot of subgroup analyses by exercise intensity

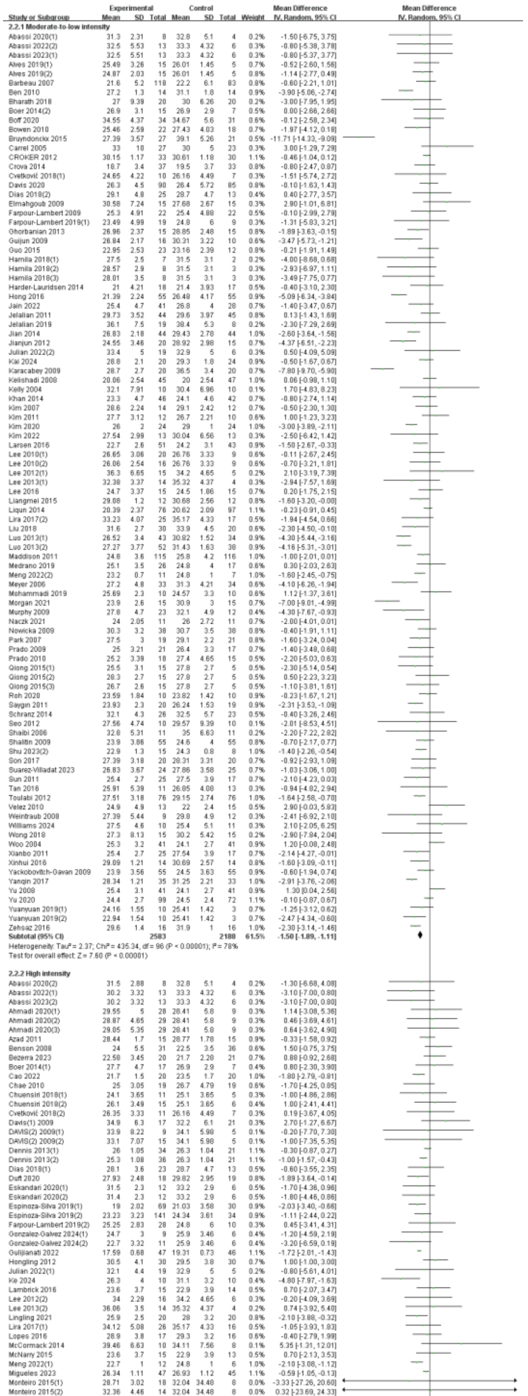

Figure S2.3. Forest plot of subgroup analyses by duration per session

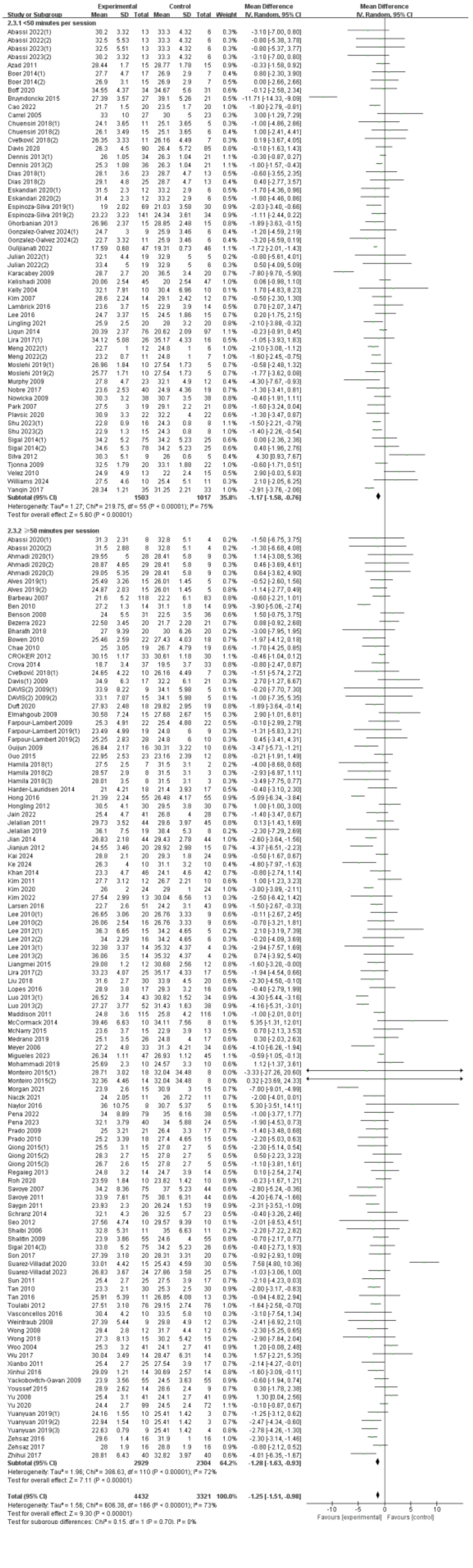

Figure S2.4. Forest plot of subgroup analyses by exercise frequency

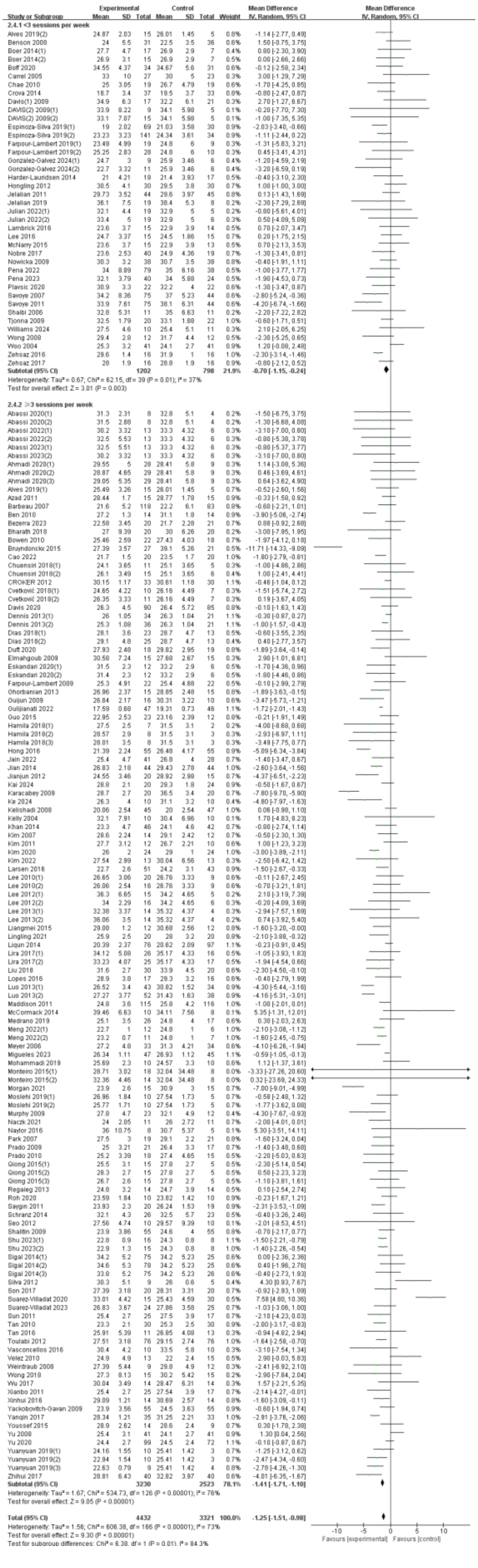

Figure S2.5. Forest plot of subgroup analyses by weekly exercise duration

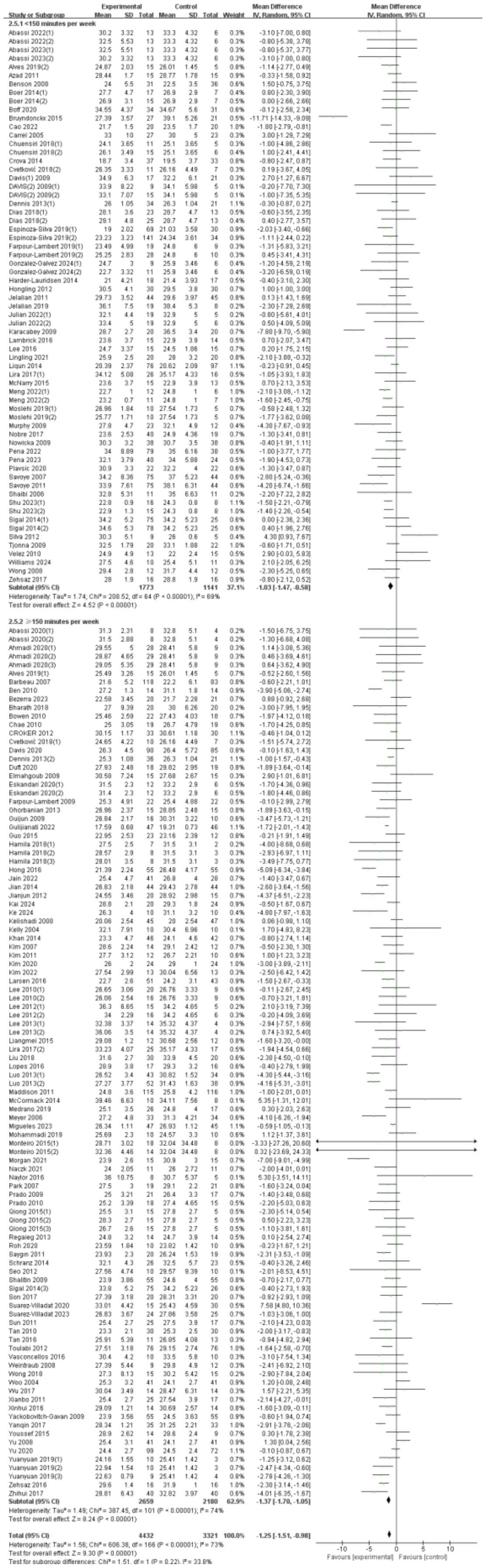

Figure S2.6. Forest plot of subgroup analyses by intervention duration

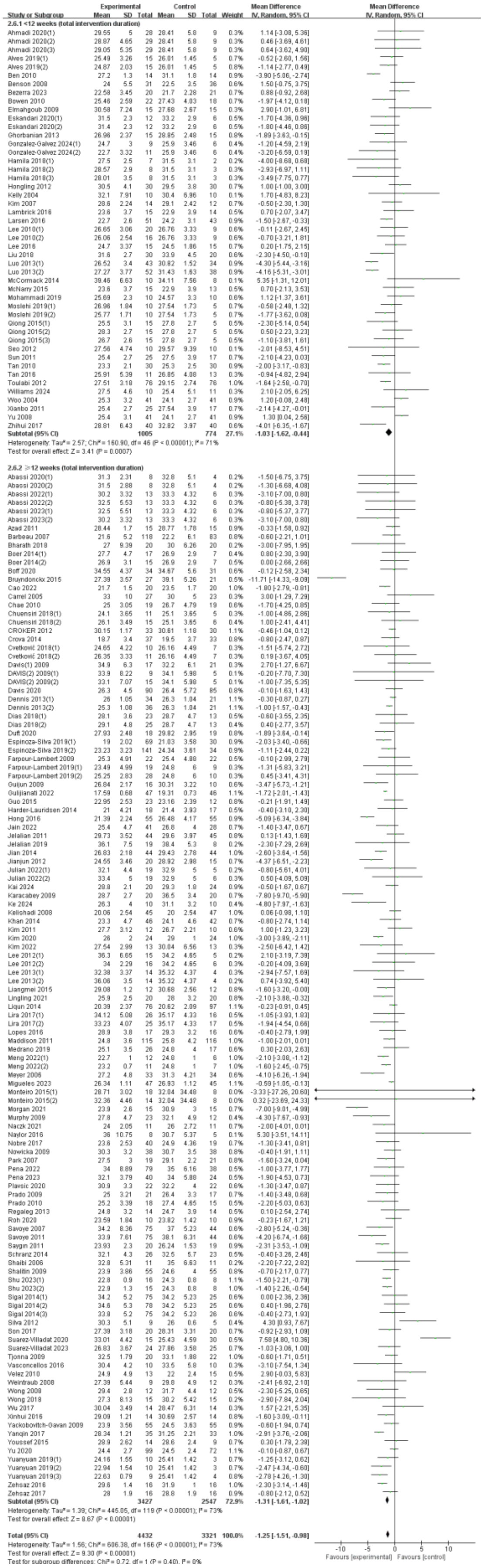

Figure S2.7. Forest plot of subgroup analyses by region

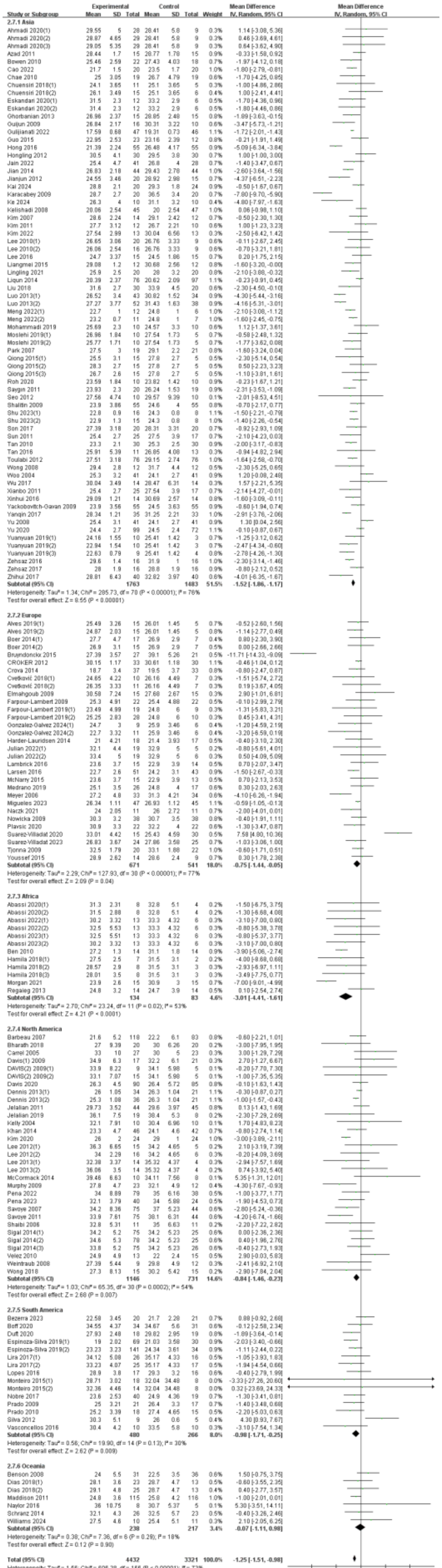

Figure S2.8. Forest plot of subgroup analyses by economic status

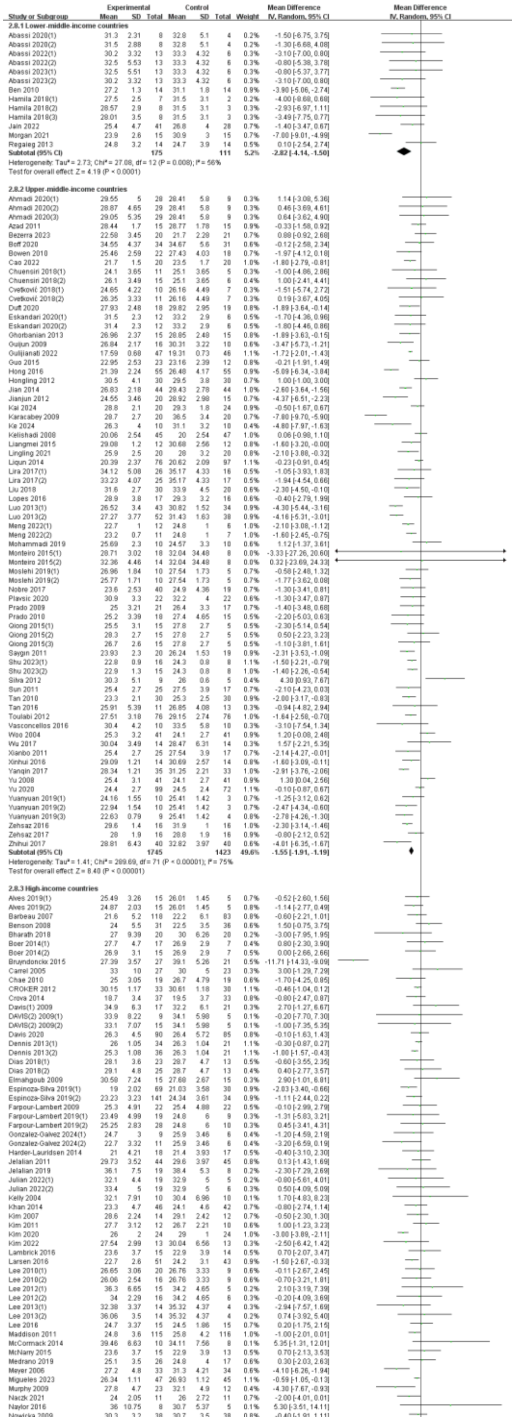

Figure S2.9. Forest plot of subgroup analyses by weight status

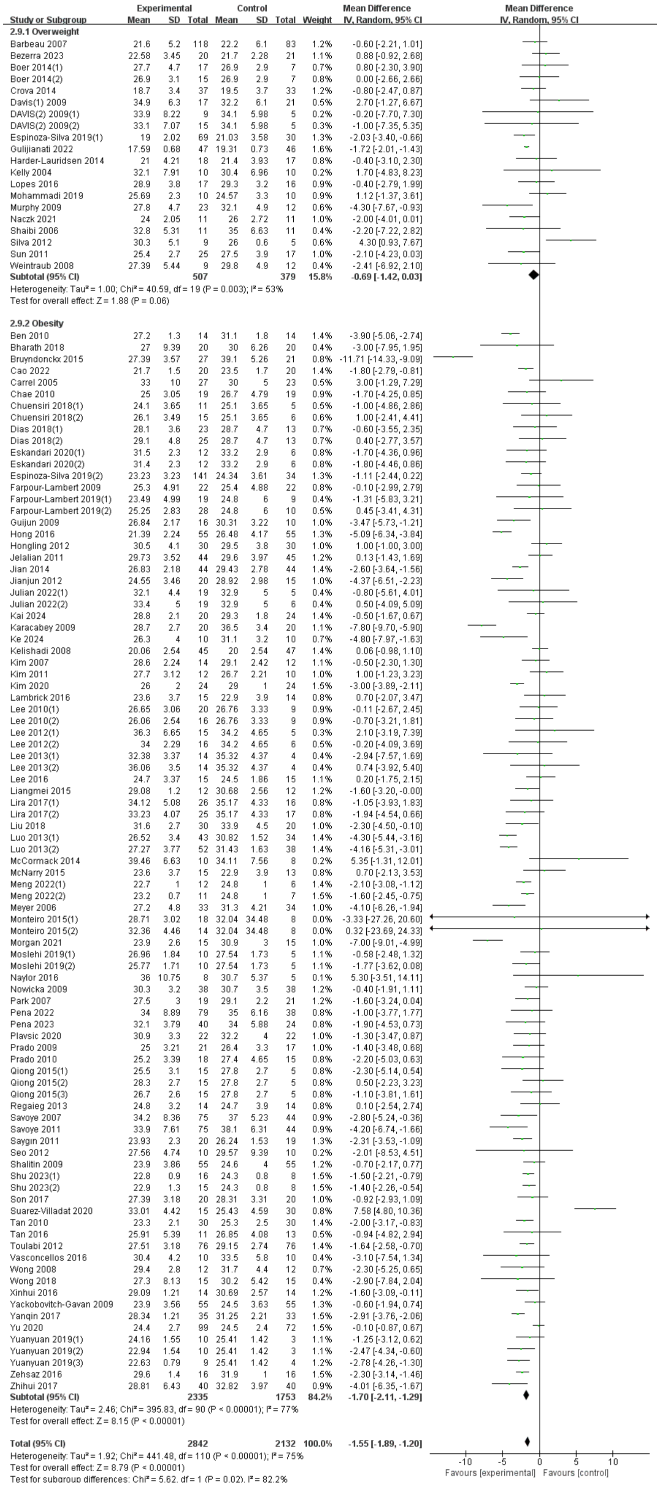

Figure S2.10. Forest plot of subgroup analyses by sex

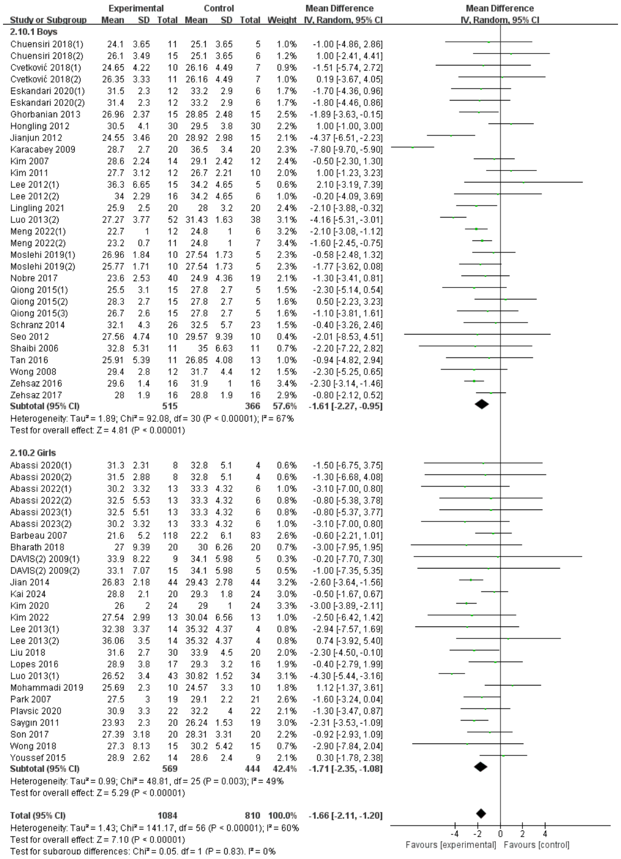

Figure S2.11. Forest plot of subgroup analyses by metabolic status

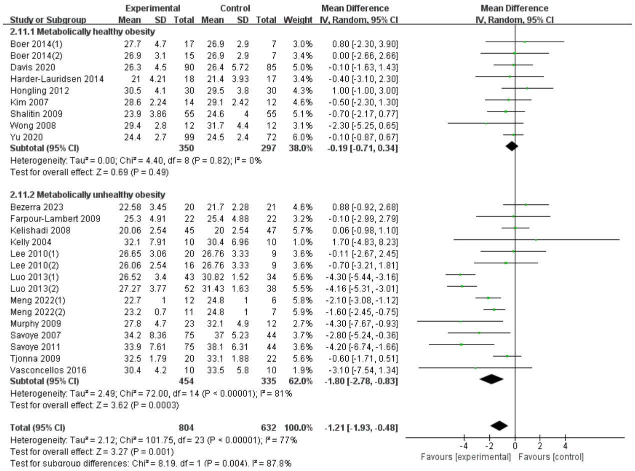

Figure S3. Forest plots of subgroup analyses for WC in children and adolescents with overweight or obesity

Figure S3.1. Forest plot of subgroup analyses by type of exercise

Study or Subgroup	Experimental Mean	Experimental SD	Experimental Total	Control Mean	Control SD	Control Total	Weight	Mean Difference IV, Random, 95% CI
**2.1.1 Aerobic exercise**								
Abassi 2020(1)	103	8.84	8	102	8.93	4	0.3%	1.00 [-9.68, 11.68]
Abassi 2022(2)	102	9.43	13	101	6.99	6	0.5%	1.00 [-6.59, 8.59]
Abassi 2023(1)	102	9.43	13	101	6.99	6	0.5%	1.00 [-6.59, 8.59]
Ahmadi 2020(1)	93.53	12.91	28	93.2	14.55	9	0.3%	0.33 [-10.31, 10.97]
Barbeau 2007	67.9	11.3	118	69.9	12.5	83	1.3%	-2.00 [-5.37, 1.37]
Bezerra 2023	68.7	7.35	20	67.02	4.14	21	1.2%	1.68 [-2.00, 5.36]
Boer 2014(2)	93.4	9.6	15	95.9	8.2	7	0.5%	-2.50 [-10.28, 5.28]
Boff 2020	100.51	11.17	34	101.96	15.38	31	0.7%	-1.45 [-8.04, 5.14]
CROKER 2012	89.58	3.28	33	90.48	3.31	30	1.7%	-0.90 [-2.53, 0.73]
Davis 2020	79.1	9.38	90	78	11.34	85	1.4%	1.10 [-1.99, 4.19]
Dennis 2013(1)	76.9	3.73	34	77.7	3.82	21	1.6%	-0.80 [-2.86, 1.26]
Dennis 2013(2)	74.8	3.9	36	77.7	3.82	21	1.6%	-2.90 [-4.97, -0.83]
Guo 2015	77.77	7.24	23	77.6	6.82	12	0.9%	0.17 [-4.69, 5.03]
Hamila 2018(1)	75.9	11.7	7	91.6	8.6	2	0.2%	-15.70 [-30.44, -0.96]
Hamila 2018(2)	86.1	6.5	8	91.6	8.6	3	0.3%	-5.50 [-16.22, 5.22]
Hamila 2018(3)	88.5	7.4	8	91.6	8.6	3	0.3%	-3.10 [-14.10, 7.90]
Hay 2016(1)	106	8.9	32	109	15	18	0.5%	-3.00 [-10.58, 4.58]
Jain 2022	85.7	11.5	41	88.4	9.3	28	0.9%	-2.70 [-7.63, 2.23]
Jian 2014	76.31	3.48	44	83.87	3.62	44	1.8%	-7.56 [-9.04, -6.08]
Jianjun 2012	92.46	8.36	20	107.64	7.76	15	0.9%	-15.18 [-20.55, -9.81]
Kai 2024	85.1	0.8	20	87	1.8	24	1.9%	-1.90 [-2.70, -1.10]
Kelishadi 2008	60.2	5.38	45	61	5.23	47	1.6%	-0.80 [-2.97, 1.37]
Kim 2007	89.6	7.48	14	91.2	4.16	12	1.0%	-1.60 [-6.17, 2.97]
Kim 2011	87.1	6.58	12	84.8	5.06	10	0.9%	2.30 [-2.57, 7.17]
Kim 2020	85.5	2.7	24	87.7	1.8	24	1.8%	-2.20 [-3.50, -0.90]
Larsen 2016	79.4	8.6	51	82	8.8	43	1.3%	-2.60 [-6.13, 0.93]
Lee 2010(2)	79.63	7.73	16	82.79	10.7	9	0.5%	-3.16 [-11.11, 4.79]
Lee 2012(1)	103.3	12.28	15	100.5	10.61	5	0.3%	2.80 [-8.39, 13.99]
Lee 2013(1)	104.11	10.97	14	110.53	8.76	4	0.3%	-6.42 [-16.75, 3.91]
Liqun 2014	66.33	9.21	76	69.27	9.29	97	1.4%	-2.94 [-5.72, -0.16]
Liu 2018	95.5	8.2	30	100.8	11.1	20	0.8%	-5.30 [-10.98, 0.38]
Lofrano-Prado 2022	102.6	12.7	37	105.9	13.5	37	0.8%	-3.30 [-9.27, 2.67]
Luo 2013(1)	82.29	7.7	43	95.37	5.81	34	1.4%	-13.08 [-16.10, -10.06]
Luo 2013(2)	84.34	7.47	52	98.07	8.4	38	1.3%	-13.73 [-17.08, -10.38]
Maddison 2011	84.4	10.8	115	88	10.7	116	1.4%	-3.60 [-6.37, -0.83]
Meng 2022(2)	78.5	7.5	11	84.9	7.2	7	0.6%	-6.40 [-13.33, 0.53]
Monteiro 2015(1)	85.52	6.08	18	95.52	11.8	8	0.5%	-10.00 [-18.65, -1.35]
Park 2007	81.9	6.6	19	85	5.6	21	1.2%	-3.10 [-6.91, 0.71]
Plavsic 2020	91.5	7.8	22	96.9	11.4	22	0.8%	-5.40 [-11.17, 0.37]
Racil(1) 2016(2)	91	4	16	94	4	7	1.2%	-3.00 [-6.55, 0.55]
Racil 2013(2)	91	4.6	11	92.8	3.7	6	1.1%	-1.80 [-5.82, 2.22]
Racil 2024(2)	92	4.9	12	94.83	4.3	6	1.0%	-2.83 [-7.25, 1.59]
Regaieg 2013	79.8	6.2	14	78.6	8.5	14	0.8%	1.20 [-4.31, 6.71]
Seo 2019	96.32	9.68	26	93.67	13.97	44	0.8%	2.65 [-2.91, 8.21]
Shu 2023(2)	80	4.7	15	82.9	5.2	8	1.1%	-2.90 [-7.22, 1.42]
Sigal 2014(1)	93.6	11.26	75	94.9	11.33	25	0.9%	-1.30 [-6.42, 3.82]
Staiano 2017	107.8	21.82	20	108.2	22.51	18	0.2%	-0.40 [-14.53, 13.73]
Suarez-Villadat 2023	73.29	7.93	24	74.77	8.61	25	1.0%	-1.48 [-6.11, 3.15]
Sun 2011	81.9	8.5	25	82.1	8.9	17	0.9%	-0.20 [-5.59, 5.19]
Tan 2010	79	7.2	30	81.7	7.2	30	1.2%	-2.70 [-6.34, 0.94]
Tjonna 2009	104.9	7.6	20	104.2	7.5	22	1.0%	0.70 [-3.87, 5.27]
Toulabi 2012	96.23	9.5	76	100.4	6.99	76	1.5%	-4.17 [-6.82, -1.52]
Xianbo 2011	81.9	8.5	25	82.1	8.9	17	0.9%	-0.20 [-5.59, 5.19]
Xinhui 2016	93.01	5.62	14	101.39	5.59	14	1.1%	-8.38 [-12.53, -4.23]
Yanqin 2017	83.32	7.14	35	88.12	6.85	33	1.3%	-4.80 [-8.13, -1.47]
Yu 2020	84	10.1	99	85.1	5.8	72	1.5%	-1.10 [-3.50, 1.30]
Yuanyuan 2019(1)	79.56	3.35	10	81.22	3.21	3	1.1%	-1.66 [-5.84, 2.52]
Yuanyuan 2019(2)	78.92	3.36	10	81.22	3.21	3	1.1%	-2.30 [-6.49, 1.89]
**Subtotal (95% CI)**			**1816**			**1467**	**57.3%**	**-2.93 [-3.87, -1.99]**
Heterogeneity: Tau² = 7.36; Chi² = 216.57, df = 57 (P < 0.00001); I² = 74%								
Test for overall effect: Z = 6.12 (P < 0.00001)								
**2.1.2 Anaerobic exercise**								
Abassi 2020(2)	98	8.59	8	102	8.93	4	0.3%	-4.00 [-14.58, 6.58]
Abassi 2022(1)	101	8.51	13	101	6.99	6	0.6%	0.00 [-7.26, 7.26]
Abassi 2023(2)	101	8.51	13	101	6.99	6	0.6%	0.00 [-7.26, 7.26]
Boer 2014(1)	91.5	13.1	17	95.9	8.2	7	0.4%	-4.40 [-13.10, 4.30]
Chuensiri 2018(1)	84.2	8.95	11	84.7	8.95	5	0.4%	-0.50 [-9.96, 8.96]
Chuensiri 2018(2)	88.6	8.91	15	84.7	8.95	6	0.5%	3.90 [-4.56, 12.36]
Espinoza-Silva 2019(1)	61.77	8.12	69	66.93	10.85	30	1.1%	-5.16 [-9.49, -0.83]
Espinoza-Silva 2019(2)	71.5	9.71	141	72.74	9.24	34	1.3%	-1.24 [-4.73, 2.25]
Hay 2016(2)	102	15	38	109	15	18	0.5%	-7.00 [-15.41, 1.41]
Ke 2024	84	10.4	10	100.1	8.3	10	0.5%	-16.10 [-24.35, -7.85]
Lambrick 2016	70.9	8.7	15	71.6	11.9	14	0.5%	-0.70 [-8.33, 6.93]
Lingling 2021	89.7	7	20	94.2	7.5	20	1.0%	-4.50 [-9.00, -0.00]
Meng 2022(1)	78.8	6.1	12	84.9	7.2	6	0.6%	-6.10 [-12.82, 0.62]
Pena 2022	107	17.78	79	111	18.49	38	0.6%	-4.00 [-11.07, 3.07]
Racil(1) 2016(1)	91	6	17	94	4	7	1.1%	-3.00 [-7.11, 1.11]
Racil(2) 2016(1)	90	6	23	92	3	9	1.4%	-2.00 [-5.14, 1.14]
Racil(2) 2016(2)	90	4	26	92	3	10	1.5%	-2.00 [-4.41, 0.41]
Racil 2013(1)	90.3	6.7	11	92.8	3.7	6	0.9%	-2.50 [-7.44, 2.44]
Racil 2024(1)	90.12	3.9	12	94.83	4.3	5	1.1%	-4.71 [-9.08, -0.34]
Salus(1) 2022	96.3	8.98	14	103.9	17.96	14	0.3%	-7.60 [-18.12, 2.92]
Shu 2023(1)	86	6.9	16	82.9	5.2	8	0.9%	3.10 [-1.84, 8.04]
Suarez-Villadat 2020	15.34	9.18	15	21.52	9.85	30	0.8%	-6.18 [-12.01, -0.35]
Vasconcellos 2016	90.5	8.9	10	101.9	17	10	0.3%	-11.40 [-23.29, 0.49]
Yuanyuan 2019(3)	73.14	3.24	9	81.22	3.21	4	1.2%	-8.08 [-11.87, -4.29]
**Subtotal (95% CI)**			**614**			**307**	**18.4%**	**-3.47 [-4.89, -2.05]**
Heterogeneity: Tau² = 4.04; Chi² = 36.44, df = 23 (P = 0.04); I² = 37%								
Test for overall effect: Z = 4.79 (P < 0.00001)								
**2.1.3 Resistance exercise**								
Ahmadi 2020(2)	92.32	13.3	29	93.2	14.55	9	0.3%	-0.88 [-11.55, 9.79]
Benson 2008	75.2	11.9	31	70.1	6.7	36	1.0%	5.10 [0.37, 9.83]
Lee 2012(2)	97.9	8	16	100.5	10.61	6	0.4%	-2.60 [-11.95, 6.75]
Lee 2013(2)	113.58	11.26	14	110.53	8.76	4	0.3%	3.05 [-7.37, 13.47]
Sigal 2014(2)	96.7	11.48	78	94.9	11.33	25	0.9%	1.80 [-3.32, 6.92]
Zhihui 2017	89.38	12.45	40	101.21	14.43	40	0.8%	-11.83 [-17.74, -5.92]
**Subtotal (95% CI)**			**208**			**120**	**3.7%**	**-0.93 [-6.78, 4.92]**
Heterogeneity: Tau² = 38.21; Chi² = 20.94, df = 5 (P = 0.0008); I² = 76%								
Test for overall effect: Z = 0.31 (P = 0.76)								
**2.1.4 Combined aerobic and resistance exercise**								
Ahmadi 2020(3)	94.58	13.9	29	93.2	14.55	9	0.3%	1.38 [-9.39, 12.15]
Bharath 2018	83.5	2.24	20	88.2	4.47	20	1.6%	-4.70 [-6.89, -2.51]
Duft 2020	89.29	7.46	18	92.99	7.14	19	1.0%	-3.70 [-8.41, 1.01]
Elmahgoub 2009	96.18	17.82	15	96.39	8.62	15	0.4%	-0.21 [-10.23, 9.81]
Faria 2020(1)	76	4.97	24	76.5	5.08	13	1.3%	-0.50 [-3.90, 2.90]
Faria 2020(2)	75.2	5.08	26	76.5	5.08	13	1.3%	-1.30 [-4.68, 2.08]
Farpour-Lambert 2019(1)	79.63	12.55	19	80	12	9	0.4%	-0.37 [-10.03, 9.29]
Farpour-Lambert 2019(2)	79.16	10.61	28	80	12	10	0.5%	-0.84 [-9.25, 7.57]
Harder-Lauridsen 2014	75.9	8.93	18	78.7	7.63	17	0.8%	-2.80 [-8.29, 2.69]
Heidarianpour 2023	68.29	12.96	15	69.47	12.18	15	0.4%	-1.18 [-10.18, 7.82]
Kim 2022	84.2	7.5	13	86.5	10.4	13	0.6%	-2.30 [-9.27, 4.67]
Lee 2010(1)	78.29	9.72	20	82.79	10.7	9	0.5%	-4.50 [-12.69, 3.69]
Liangmei 2015	93	5.61	12	101.38	5.58	12	1.0%	-8.38 [-12.86, -3.90]
Migueles 2023	91.85	3.75	47	93.02	3.74	45	1.8%	-1.17 [-2.70, 0.36]
Mohammadi 2019	80.1	9.6	10	75	6.3	10	0.6%	5.10 [-2.02, 12.22]
Monteiro 2015(2)	93.52	7.55	14	95.52	11.8	8	0.4%	-2.00 [-11.08, 7.08]
Shalitin 2009	78.7	14.1	55	79.3	14.1	55	0.9%	-0.60 [-5.87, 4.67]
Sigal 2014(3)	92.8	11.26	75	94.9	11.33	26	0.9%	-2.10 [-7.15, 2.95]
Son 2017	84.5	3.13	20	86.3	4.47	20	1.5%	-1.80 [-4.19, 0.59]
Wong 2018	83.5	1.94	15	88.2	3.87	15	1.6%	-4.70 [-6.89, -2.51]
Zehsaz 2016	85.3	3.1	16	89.34	2.7	16	1.6%	-4.04 [-6.05, -2.03]
Zehsaz 2017	83.9	4.9	16	85.8	6.1	16	1.2%	-1.90 [-5.73, 1.93]
**Subtotal (95% CI)**			**525**			**385**	**20.6%**	**-2.64 [-3.62, -1.67]**
Heterogeneity: Tau² = 1.14; Chi² = 28.08, df = 21 (P = 0.14); I² = 25%								
Test for overall effect: Z = 5.31 (P < 0.00001)								
**Total (95% CI)**			**3163**			**2279**	**100.0%**	**-2.86 [-3.52, -2.20]**
Heterogeneity: Tau² = 5.91; Chi² = 306.04, df = 109 (P < 0.00001); I² = 64%								
Test for overall effect: Z = 8.45 (P < 0.00001)								
Test for subgroup differences: Chi² = 1.33, df = 3 (P = 0.72), I² = 0%								

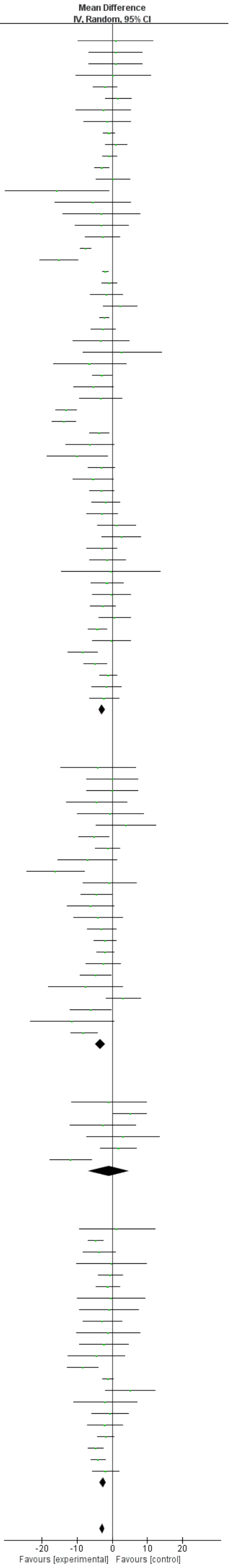

Figure S3.2. Forest plot of subgroup analyses by exercise intensity

Study or Subgroup	Experimental Mean	Experimental SD	Experimental Total	Control Mean	Control SD	Control Total	Weight	Mean Difference IV, Random, 95% CI
**2.2.1 Moderate-to-low intensity**								
Abassi 2020(1)	103	8.84	8	102	8.93	4	0.3%	1.00 [-9.68, 11.68]
Abassi 2022(2)	102	9.43	13	101	6.99	6	0.5%	1.00 [-6.59, 8.59]
Abassi 2023(1)	102	9.43	13	101	6.99	6	0.5%	1.00 [-6.59, 8.59]
Barbeau 2007	67.9	11.3	118	69.9	12.5	83	1.3%	-2.00 [-5.37, 1.37]
Bharath 2018	83.5	2.24	20	88.2	4.47	20	1.6%	-4.70 [-6.89, -2.51]
Boer 2014(2)	93.4	9.6	15	95.9	8.2	7	0.5%	-2.50 [-10.28, 5.28]
Boff 2020	100.51	11.17	34	101.96	15.38	31	0.7%	-1.45 [-8.04, 5.14]
CROKER 2012	89.58	3.28	33	90.48	3.31	30	1.7%	-0.90 [-2.53, 0.73]
Davis 2020	79.1	9.38	90	78	11.34	85	1.4%	1.10 [-1.99, 4.19]
Elmahgoub 2009	96.18	17.82	15	96.39	8.62	15	0.4%	-0.21 [-10.23, 9.81]
Faria 2020(1)	76	4.97	24	76.5	5.08	13	1.3%	-0.50 [-3.90, 2.90]
Farpour-Lambert 2019(1)	79.63	12.55	19	80	12	9	0.4%	-0.37 [-10.03, 9.29]
Guo 2015	77.77	7.24	23	77.6	6.82	12	0.9%	0.17 [-4.69, 5.03]
Hamila 2018(1)	75.9	11.7	7	91.6	8.6	2	0.2%	-15.70 [-30.44, -0.96]
Hamila 2018(2)	86.1	6.5	8	91.6	8.6	3	0.3%	-5.50 [-16.22, 5.22]
Hamila 2018(3)	88.5	7.4	8	91.6	8.6	3	0.3%	-3.10 [-14.10, 7.90]
Harder-Lauridsen 2014	75.9	8.93	18	78.7	7.63	17	0.8%	-2.80 [-8.29, 2.69]
Hay 2016(1)	106	8.9	32	109	15	18	0.5%	-3.00 [-10.58, 4.58]
Heidarianpour 2023	68.29	12.96	15	69.47	12.18	15	0.4%	-1.18 [-10.18, 7.82]
Jain 2022	85.7	11.5	41	88.4	9.3	28	0.9%	-2.70 [-7.63, 2.23]
Jian 2014	76.31	3.48	44	83.87	3.62	44	1.8%	-7.56 [-9.04, -6.08]
Jianjun 2012	92.46	8.36	20	107.64	7.76	15	0.9%	-15.18 [-20.55, -9.81]
Kai 2024	85.1	0.8	20	87	1.8	24	1.9%	-1.90 [-2.70, -1.10]
Kelishadi 2008	60.2	5.38	45	61	5.23	47	1.6%	-0.80 [-2.97, 1.37]
Kim 2007	89.6	7.48	14	91.2	4.16	12	1.0%	-1.60 [-6.17, 2.97]
Kim 2011	87.1	6.58	12	84.8	5.06	10	0.9%	2.30 [-2.57, 7.17]
Kim 2020	85.5	2.7	24	87.7	1.8	24	1.8%	-2.20 [-3.50, -0.90]
Kim 2022	84.2	7.5	13	86.5	10.4	13	0.6%	-2.30 [-9.27, 4.67]
Larsen 2016	79.4	8.6	51	82	8.8	43	1.3%	-2.60 [-6.13, 0.93]
Lee 2010(1)	78.29	9.72	20	82.79	10.7	9	0.5%	-4.50 [-12.69, 3.69]
Lee 2010(2)	79.63	7.73	16	82.79	10.7	9	0.5%	-3.16 [-11.11, 4.79]
Lee 2012(1)	103.3	12.28	15	100.5	10.61	5	0.3%	2.80 [-8.39, 13.99]
Lee 2013(1)	104.11	10.97	14	110.53	8.76	4	0.3%	-6.42 [-16.75, 3.91]
Liangmei 2015	93	5.61	12	101.38	5.58	12	1.0%	-8.38 [-12.86, -3.90]
Liqun 2014	66.33	9.21	76	69.27	9.29	97	1.4%	-2.94 [-5.72, -0.16]
Liu 2018	95.5	8.2	30	100.8	11.1	20	0.8%	-5.30 [-10.98, 0.38]
Lofrano-Prado 2022	102.6	12.7	37	105.9	13.5	37	0.8%	-3.30 [-9.27, 2.67]
Luo 2013(1)	82.29	7.7	43	95.37	5.81	34	1.4%	-13.08 [-16.10, -10.06]
Luo 2013(2)	84.34	7.47	52	98.07	8.4	38	1.3%	-13.73 [-17.08, -10.38]
Maddison 2011	84.4	10.8	115	88	10.7	116	1.4%	-3.60 [-6.37, -0.83]
Meng 2022(2)	78.5	7.5	11	84.9	7.2	7	0.6%	-6.40 [-13.33, 0.53]
Mohammadi 2019	80.1	9.6	10	75	6.3	10	0.6%	5.10 [-2.02, 12.22]
Park 2007	81.9	6.6	19	85	5.6	21	1.2%	-3.10 [-6.91, 0.71]
Racil(1) 2016(2)	91	4	16	94	4	7	1.2%	-3.00 [-6.55, 0.55]
Racil 2013(2)	91	4.6	11	92.8	3.7	6	1.1%	-1.80 [-5.82, 2.22]
Racil 2024(2)	92	4.9	12	94.83	4.3	6	1.0%	-2.83 [-7.25, 1.59]
Seo 2019	96.32	9.68	26	93.67	13.97	44	0.8%	2.65 [-2.91, 8.21]
Shalitin 2009	78.7	14.1	55	79.3	14.1	55	0.9%	-0.60 [-5.87, 4.67]
Shu 2023(2)	80	4.7	15	82.9	5.2	8	1.1%	-2.90 [-7.22, 1.42]
Son 2017	84.5	3.13	20	86.3	4.47	20	1.5%	-1.80 [-4.19, 0.59]
Staiano 2017	107.8	21.82	20	108.2	22.51	18	0.2%	-0.40 [-14.53, 13.73]
Suarez-Villadat 2023	73.29	7.93	24	74.77	8.61	25	1.0%	-1.48 [-6.11, 3.15]
Sun 2011	81.9	8.5	25	82.1	8.9	17	0.9%	-0.20 [-5.59, 5.19]
Toulabi 2012	96.23	9.5	76	100.4	6.99	76	1.5%	-4.17 [-6.82, -1.52]
Wong 2018	83.5	1.94	15	88.2	3.87	15	1.6%	-4.70 [-6.89, -2.51]
Xianbo 2011	81.9	8.5	25	82.1	8.9	17	0.9%	-0.20 [-5.59, 5.19]
Xinhui 2016	93.01	5.62	14	101.39	5.59	14	1.1%	-8.38 [-12.53, -4.23]
Yanqin 2017	83.32	7.14	35	88.12	6.85	33	1.3%	-4.80 [-8.13, -1.47]
Yu 2020	84	10.1	99	85.1	5.8	72	1.5%	-1.10 [-3.50, 1.30]
Yuanyuan 2019(1)	79.56	3.35	10	81.22	3.21	3	1.1%	-1.66 [-5.84, 2.52]
Yuanyuan 2019(2)	78.92	3.36	10	81.22	3.21	3	1.1%	-2.30 [-6.49, 1.89]
Zehsaz 2016	85.3	3.1	16	89.34	2.7	16	1.6%	-4.04 [-6.05, -2.03]
**Subtotal (95% CI)**			**1791**			**1513**	**60.6%**	**-3.14 [-4.03, -2.24]**
Heterogeneity: Tau² = 6.85; Chi² = 219.11, df = 61 (P < 0.00001); I² = 72%								
Test for overall effect: Z = 6.88 (P < 0.00001)								
**2.2.2 High intensity**								
Abassi 2020(2)	98	8.59	8	102	8.93	4	0.3%	-4.00 [-14.58, 6.58]
Abassi 2022(1)	101	8.51	13	101	6.99	6	0.6%	0.00 [-7.26, 7.26]
Abassi 2023(2)	101	8.51	13	101	6.99	6	0.6%	0.00 [-7.26, 7.26]
Ahmadi 2020(1)	93.53	12.91	28	93.2	14.55	9	0.3%	0.33 [-10.31, 10.97]
Ahmadi 2020(2)	92.32	13.3	29	93.2	14.55	9	0.3%	-0.88 [-11.55, 9.79]
Ahmadi 2020(3)	94.58	13.9	29	93.2	14.55	9	0.3%	1.38 [-9.39, 12.15]
Benson 2008	75.2	11.9	31	70.1	6.7	36	1.0%	5.10 [0.37, 9.83]
Bezerra 2023	68.7	7.35	20	67.02	4.14	21	1.2%	1.68 [-2.00, 5.36]
Boer 2014(1)	91.5	13.1	17	95.9	8.2	7	0.4%	-4.40 [-13.10, 4.30]
Chuensiri 2018(1)	84.2	8.95	11	84.7	8.95	5	0.4%	-0.50 [-9.96, 8.96]
Chuensiri 2018(2)	88.6	8.91	15	84.7	8.95	6	0.5%	3.90 [-4.56, 12.36]
Dennis 2013(1)	76.9	3.73	34	77.7	3.82	21	1.6%	-0.80 [-2.86, 1.26]
Dennis 2013(2)	74.8	3.9	36	77.7	3.82	21	1.6%	-2.90 [-4.97, -0.83]
Duft 2020	89.29	7.46	18	92.99	7.14	19	1.0%	-3.70 [-8.41, 1.01]
Espinoza-Silva 2019(1)	61.77	8.12	69	66.93	10.85	30	1.1%	-5.16 [-9.49, -0.83]
Espinoza-Silva 2019(2)	71.5	9.71	141	72.74	9.24	34	1.3%	-1.24 [-4.73, 2.25]
Faria 2020(2)	75.2	5.08	26	76.5	5.08	13	1.3%	-1.30 [-4.68, 2.08]
Farpour-Lambert 2019(2)	79.16	10.61	28	80	12	10	0.5%	-0.84 [-9.25, 7.57]
Hay 2016(2)	102	15	38	109	15	18	0.5%	-7.00 [-15.41, 1.41]
Ke 2024	84	10.4	10	100.1	8.3	10	0.5%	-16.10 [-24.35, -7.85]
Lambrick 2016	70.9	8.7	15	71.6	11.9	14	0.5%	-0.70 [-8.33, 6.93]
Lee 2012(2)	97.9	8	16	100.5	10.61	6	0.4%	-2.60 [-11.95, 6.75]
Lee 2013(2)	113.58	11.26	14	110.53	8.76	4	0.3%	3.05 [-7.37, 13.47]
Lingling 2021	89.7	7	20	94.2	7.5	20	1.0%	-4.50 [-9.00, -0.00]
Meng 2022(1)	78.8	6.1	12	84.9	7.2	6	0.6%	-6.10 [-12.82, 0.62]
Migueles 2023	91.85	3.75	47	93.02	3.74	45	1.8%	-1.17 [-2.70, 0.36]
Monteiro 2015(1)	85.52	6.08	18	95.52	11.8	8	0.5%	-10.00 [-18.65, -1.35]
Monteiro 2015(2)	93.52	7.55	14	95.52	11.8	8	0.4%	-2.00 [-11.08, 7.08]
Pena 2022	107	17.78	79	111	18.49	38	0.6%	-4.00 [-11.07, 3.07]
Plavsic 2020	91.5	7.8	22	96.9	11.4	22	0.8%	-5.40 [-11.17, 0.37]
Racil(1) 2016(1)	91	6	17	94	4	7	1.1%	-3.00 [-7.11, 1.11]
Racil(2) 2016(1)	90	6	23	92	3	9	1.4%	-2.00 [-5.14, 1.14]
Racil(2) 2016(2)	90	4	26	92	3	10	1.5%	-2.00 [-4.41, 0.41]
Racil 2013(1)	90.3	6.7	11	92.8	3.7	6	0.9%	-2.50 [-7.44, 2.44]
Racil 2024(1)	90.12	3.9	12	94.83	4.3	5	1.1%	-4.71 [-9.08, -0.34]
Regaieg 2013	79.8	6.2	14	78.6	8.5	14	0.8%	1.20 [-4.31, 6.71]
Salus(1) 2022	96.3	8.98	14	103.9	17.96	14	0.3%	-7.60 [-18.12, 2.92]
Shu 2023(1)	86	6.9	16	82.9	5.2	8	0.9%	3.10 [-1.84, 8.04]
Sigal 2014(1)	93.6	11.26	75	94.9	11.33	25	0.9%	-1.30 [-6.42, 3.82]
Sigal 2014(2)	96.7	11.48	78	94.9	11.33	25	0.9%	1.80 [-3.32, 6.92]
Sigal 2014(3)	92.8	11.26	75	94.9	11.33	26	0.9%	-2.10 [-7.15, 2.95]
Suarez-Villadat 2020	15.34	9.18	15	21.52	9.85	30	0.8%	-6.18 [-12.01, -0.35]
Tan 2010	79	7.2	30	81.7	7.2	30	1.2%	-2.70 [-6.34, 0.94]
Tjonna 2009	104.9	7.6	20	104.2	7.5	22	1.0%	0.70 [-3.87, 5.27]
Vasconcellos 2016	90.5	8.9	10	101.9	17	10	0.3%	-11.40 [-23.29, 0.49]
Yuanyuan 2019(3)	73.14	3.24	9	81.22	3.21	4	1.2%	-8.08 [-11.87, -4.29]
Zehsaz 2017	83.9	4.9	16	85.8	6.1	16	1.2%	-1.90 [-5.73, 1.93]
Zhihui 2017	89.38	12.45	40	101.21	14.43	40	0.8%	-11.83 [-17.74, -5.92]
**Subtotal (95% CI)**			**1372**			**766**	**39.4%**	**-2.35 [-3.29, -1.42]**
Heterogeneity: Tau² = 3.57; Chi² = 80.13, df = 47 (P = 0.002); I² = 41%								
Test for overall effect: Z = 4.93 (P < 0.00001)								
**Total (95% CI)**			**3163**			**2279**	**100.0%**	**-2.86 [-3.52, -2.20]**
Heterogeneity: Tau² = 5.91; Chi² = 306.04, df = 109 (P < 0.00001); I² = 64%								
Test for overall effect: Z = 8.45 (P < 0.00001)								
Test for subgroup differences: Chi² = 1.41, df = 1 (P = 0.24), I² = 28.9%								

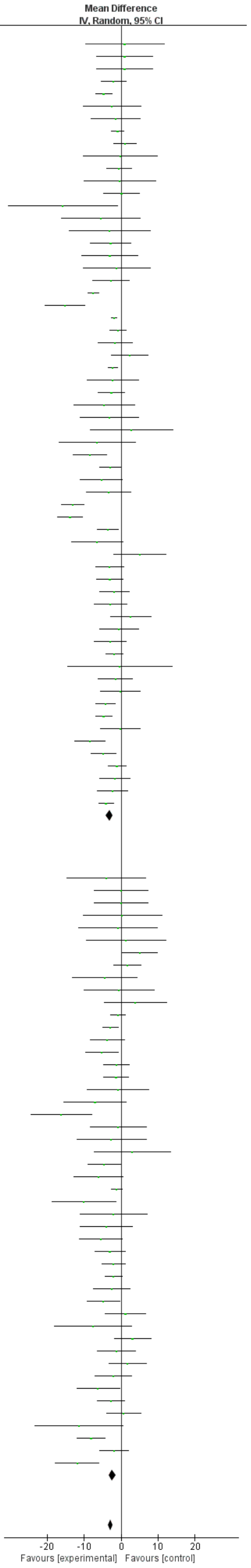

Figure S3.3. Forest plot of subgroup analyses by duration per session

Study or Subgroup	Experimental Mean	Experimental SD	Experimental Total	Control Mean	Control SD	Control Total	Weight	Mean Difference IV, Random, 95% CI
**2.3.1 <50 minutes per session**								
Abassi 2022(1)	101	8.51	13	101	6.99	6	0.6%	0.00 [-7.26, 7.26]
Abassi 2022(2)	102	9.43	13	101	6.99	6	0.5%	1.00 [-6.59, 8.59]
Abassi 2023(1)	102	9.43	13	101	6.99	6	0.5%	1.00 [-6.59, 8.59]
Abassi 2023(2)	101	8.51	13	101	6.99	6	0.6%	0.00 [-7.26, 7.26]
Boer 2014(1)	91.5	13.1	17	95.9	8.2	7	0.4%	-4.40 [-13.10, 4.30]
Boer 2014(2)	93.4	9.6	15	95.9	8.2	7	0.5%	-2.50 [-10.28, 5.28]
Boff 2020	100.51	11.17	34	101.96	15.38	31	0.7%	-1.45 [-8.04, 5.14]
Chuensiri 2018(1)	84.2	8.95	11	84.7	8.95	5	0.4%	-0.50 [-9.96, 8.96]
Chuensiri 2018(2)	88.6	8.91	15	84.7	8.95	6	0.5%	3.90 [-4.56, 12.36]
Davis 2020	79.1	9.38	90	78	11.34	85	1.4%	1.10 [-1.99, 4.19]
Dennis 2013(1)	76.9	3.73	34	77.7	3.82	21	1.6%	-0.80 [-2.86, 1.26]
Dennis 2013(2)	74.8	3.9	36	77.7	3.82	21	1.6%	-2.90 [-4.97, -0.83]
Espinoza-Silva 2019(1)	61.77	8.12	69	66.93	10.85	30	1.1%	-5.16 [-9.49, -0.83]
Espinoza-Silva 2019(2)	71.5	9.71	141	72.74	9.24	34	1.3%	-1.24 [-4.73, 2.25]
Faria 2020(2)	75.2	5.08	26	76.5	5.08	13	1.3%	-1.30 [-4.68, 2.08]
Hay 2016(1)	106	8.9	32	109	15	18	0.5%	-3.00 [-10.58, 4.58]
Hay 2016(2)	102	15	38	109	15	18	0.5%	-7.00 [-15.41, 1.41]
Kelishadi 2008	60.2	5.38	45	61	5.23	47	1.6%	-0.80 [-2.97, 1.37]
Kim 2007	89.6	7.48	14	91.2	4.16	12	1.0%	-1.60 [-6.17, 2.97]
Lambrick 2016	70.9	8.7	15	71.6	11.9	14	0.5%	-0.70 [-8.33, 6.93]
Lingling 2021	89.7	7	20	94.2	7.5	20	1.0%	-4.50 [-9.00, -0.00]
Liqun 2014	66.33	9.21	76	69.27	9.29	97	1.4%	-2.94 [-5.72, -0.16]
Meng 2022(1)	78.8	6.1	12	84.9	7.2	6	0.6%	-6.10 [-12.82, 0.62]
Meng 2022(2)	78.5	7.5	11	84.9	7.2	7	0.6%	-6.40 [-13.33, 0.53]
Park 2007	81.9	6.6	19	85	5.6	21	1.2%	-3.10 [-6.91, 0.71]
Plavsic 2020	91.5	7.8	22	96.9	11.4	22	0.8%	-5.40 [-11.17, 0.37]
Racil(1) 2016(1)	91	6	17	94	4	7	1.1%	-3.00 [-7.11, 1.11]
Racil(1) 2016(2)	91	4	16	94	4	7	1.2%	-3.00 [-6.55, 0.55]
Racil(2) 2016(1)	90	6	23	92	3	9	1.4%	-2.00 [-5.14, 1.14]
Racil(2) 2016(2)	90	4	26	92	3	10	1.5%	-2.00 [-4.41, 0.41]
Racil 2013(1)	90.3	6.7	11	92.8	3.7	6	0.9%	-2.50 [-7.44, 2.44]
Racil 2013(2)	91	4.6	11	92.8	3.7	6	1.1%	-1.80 [-5.82, 2.22]
Racil 2024(1)	90.12	3.9	12	94.83	4.3	5	1.1%	-4.71 [-9.08, -0.34]
Racil 2024(2)	92	4.9	12	94.83	4.3	6	1.0%	-2.83 [-7.25, 1.59]
Salus(1) 2022	96.3	8.98	14	103.9	17.96	14	0.3%	-7.60 [-18.12, 2.92]
Shu 2023(1)	86	6.9	16	82.9	5.2	8	0.9%	3.10 [-1.84, 8.04]
Shu 2023(2)	80	4.7	15	82.9	5.2	8	1.1%	-2.90 [-7.22, 1.42]
Sigal 2014(1)	93.6	11.26	75	94.9	11.33	25	0.9%	-1.30 [-6.42, 3.82]
Sigal 2014(2)	96.7	11.48	78	94.9	11.33	25	0.9%	1.80 [-3.32, 6.92]
Tjonna 2009	104.9	7.6	20	104.2	7.5	22	1.0%	0.70 [-3.87, 5.27]
Yanqin 2017	83.32	7.14	35	88.12	6.85	33	1.3%	-4.80 [-8.13, -1.47]
**Subtotal (95% CI)**			**1225**			**757**	**38.8%**	**-1.99 [-2.62, -1.36]**
Heterogeneity: Tau² = 0.00; Chi² = 35.15, df = 40 (P = 0.69); I² = 0%								
Test for overall effect: Z = 6.17 (P < 0.00001)								
**2.3.2 ⩾50 minutes per session**								
Abassi 2020(1)	103	8.84	8	102	8.93	4	0.3%	1.00 [-9.68, 11.68]
Abassi 2020(2)	98	8.59	8	102	8.93	4	0.3%	-4.00 [-14.58, 6.58]
Ahmadi 2020(1)	93.53	12.91	28	93.2	14.55	9	0.3%	0.33 [-10.31, 10.97]
Ahmadi 2020(2)	92.32	13.3	29	93.2	14.55	9	0.3%	-0.88 [-11.55, 9.79]
Ahmadi 2020(3)	94.58	13.9	29	93.2	14.55	9	0.3%	1.38 [-9.39, 12.15]
Barbeau 2007	67.9	11.3	118	69.9	12.5	83	1.3%	-2.00 [-5.37, 1.37]
Benson 2008	75.2	11.9	31	70.1	6.7	36	1.0%	5.10 [0.37, 9.83]
Bezerra 2023	68.7	7.35	20	67.02	4.14	21	1.2%	1.68 [-2.00, 5.36]
Bharath 2018	83.5	2.24	20	88.2	4.47	20	1.6%	-4.70 [-6.89, -2.51]
CROKER 2012	89.58	3.28	33	90.48	3.31	30	1.7%	-0.90 [-2.53, 0.73]
Duft 2020	89.29	7.46	18	92.99	7.14	19	1.0%	-3.70 [-8.41, 1.01]
Elmahgoub 2009	96.18	17.82	15	96.39	8.62	15	0.4%	-0.21 [-10.23, 9.81]
Faria 2020(1)	76	4.97	24	76.5	5.08	13	1.3%	-0.50 [-3.90, 2.90]
Farpour-Lambert 2019(1)	79.63	12.55	19	80	12	9	0.4%	-0.37 [-10.03, 9.29]
Farpour-Lambert 2019(2)	79.16	10.61	28	80	12	10	0.5%	-0.84 [-9.25, 7.57]
Guo 2015	77.77	7.24	23	77.6	6.82	12	0.9%	0.17 [-4.69, 5.03]
Hamila 2018(1)	75.9	11.7	7	91.6	8.6	2	0.2%	-15.70 [-30.44, -0.96]
Hamila 2018(2)	86.1	6.5	8	91.6	8.6	3	0.3%	-5.50 [-16.22, 5.22]
Hamila 2018(3)	88.5	7.4	8	91.6	8.6	3	0.3%	-3.10 [-14.10, 7.90]
Harder-Lauridsen 2014	75.9	8.93	18	78.7	7.63	17	0.8%	-2.80 [-8.29, 2.69]
Heidarianpour 2023	68.29	12.96	15	69.47	12.18	15	0.4%	-1.18 [-10.18, 7.82]
Jain 2022	85.7	11.5	41	88.4	9.3	28	0.9%	-2.70 [-7.63, 2.23]
Jian 2014	76.31	3.48	44	83.87	3.62	44	1.8%	-7.56 [-9.04, -6.08]
Jianjun 2012	92.46	8.36	20	107.64	7.76	15	0.9%	-15.18 [-20.55, -9.81]
Kai 2024	85.1	0.8	20	87	1.8	24	1.9%	-1.90 [-2.70, -1.10]
Ke 2024	84	10.4	10	100.1	8.3	10	0.5%	-16.10 [-24.35, -7.85]
Kim 2011	87.1	6.58	12	84.8	5.06	10	0.9%	2.30 [-2.57, 7.17]
Kim 2020	85.5	2.7	24	87.7	1.8	24	1.8%	-2.20 [-3.50, -0.90]
Kim 2022	84.2	7.5	13	86.5	10.4	13	0.6%	-2.30 [-9.27, 4.67]
Larsen 2016	79.4	8.6	51	82	8.8	43	1.3%	-2.60 [-6.13, 0.93]
Lee 2010(1)	78.29	9.72	20	82.79	10.7	9	0.5%	-4.50 [-12.69, 3.69]
Lee 2010(2)	79.63	7.73	16	82.79	10.7	9	0.5%	-3.16 [-11.11, 4.79]
Lee 2012(1)	103.3	12.28	15	100.5	10.61	5	0.3%	2.80 [-8.39, 13.99]
Lee 2012(2)	97.9	8	16	100.5	10.61	6	0.4%	-2.60 [-11.95, 6.75]
Lee 2013(1)	104.11	10.97	14	110.53	8.76	4	0.3%	-6.42 [-16.75, 3.91]
Lee 2013(2)	113.58	11.26	14	110.53	8.76	4	0.3%	3.05 [-7.37, 13.47]
Liangmei 2015	93	5.61	12	101.38	5.58	12	1.0%	-8.38 [-12.86, -3.90]
Liu 2018	95.5	8.2	30	100.8	11.1	20	0.8%	-5.30 [-10.98, 0.38]
Lofrano-Prado 2022	102.6	12.7	37	105.9	13.5	37	0.8%	-3.30 [-9.27, 2.67]
Luo 2013(1)	82.29	7.7	43	95.37	5.81	34	1.4%	-13.08 [-16.10, -10.06]
Luo 2013(2)	84.34	7.47	52	98.07	8.4	38	1.3%	-13.73 [-17.08, -10.38]
Maddison 2011	84.4	10.8	115	88	10.7	116	1.4%	-3.60 [-6.37, -0.83]
Migueles 2023	91.85	3.75	47	93.02	3.74	45	1.8%	-1.17 [-2.70, 0.36]
Mohammadi 2019	80.1	9.6	10	75	6.3	10	0.6%	5.10 [-2.02, 12.22]
Monteiro 2015(1)	85.52	6.08	18	95.52	11.8	8	0.5%	-10.00 [-18.65, -1.35]
Monteiro 2015(2)	93.52	7.55	14	95.52	11.8	8	0.4%	-2.00 [-11.08, 7.08]
Pena 2022	107	17.78	79	111	18.49	38	0.6%	-4.00 [-11.07, 3.07]
Regaieg 2013	79.8	6.2	14	78.6	8.5	14	0.8%	1.20 [-4.31, 6.71]
Seo 2019	96.32	9.68	26	93.67	13.97	44	0.8%	2.65 [-2.91, 8.21]
Shalitin 2009	78.7	14.1	55	79.3	14.1	55	0.9%	-0.60 [-5.87, 4.67]
Sigal 2014(3)	92.8	11.26	75	94.9	11.33	26	0.9%	-2.10 [-7.15, 2.95]
Son 2017	84.5	3.13	20	86.3	4.47	20	1.5%	-1.80 [-4.19, 0.59]
Staiano 2017	107.8	21.82	20	108.2	22.51	18	0.2%	-0.40 [-14.53, 13.73]
Suarez-Villadat 2020	15.34	9.18	15	21.52	9.85	30	0.8%	-6.18 [-12.01, -0.35]
Suarez-Villadat 2023	73.29	7.93	24	74.77	8.61	25	1.0%	-1.48 [-6.11, 3.15]
Sun 2011	81.9	8.5	25	82.1	8.9	17	0.9%	-0.20 [-5.59, 5.19]
Tan 2010	79	7.2	30	81.7	7.2	30	1.2%	-2.70 [-6.34, 0.94]
Toulabi 2012	96.23	9.5	76	100.4	6.99	76	1.5%	-4.17 [-6.82, -1.52]
Vasconcellos 2016	90.5	8.9	10	101.9	17	10	0.3%	-11.40 [-23.29, 0.49]
Wong 2018	83.5	1.94	15	88.2	3.87	15	1.6%	-4.70 [-6.89, -2.51]
Xianbo 2011	81.9	8.5	25	82.1	8.9	17	0.9%	-0.20 [-5.59, 5.19]
Xinhui 2016	93.01	5.62	14	101.39	5.59	14	1.1%	-8.38 [-12.53, -4.23]
Yu 2020	84	10.1	99	85.1	5.8	72	1.5%	-1.10 [-3.50, 1.30]
Yuanyuan 2019(1)	79.56	3.35	10	81.22	3.21	3	1.1%	-1.66 [-5.84, 2.52]
Yuanyuan 2019(2)	78.92	3.36	10	81.22	3.21	3	1.1%	-2.30 [-6.49, 1.89]
Yuanyuan 2019(3)	73.14	3.24	9	81.22	3.21	4	1.2%	-8.08 [-11.87, -4.29]
Zehsaz 2016	85.3	3.1	16	89.34	2.7	16	1.6%	-4.04 [-6.05, -2.03]
Zehsaz 2017	83.9	4.9	16	85.8	6.1	16	1.2%	-1.90 [-5.73, 1.93]
Zhihui 2017	89.38	12.45	40	101.21	14.43	40	0.8%	-11.83 [-17.74, -5.92]
**Subtotal (95% CI)**			**1938**			**1522**	**61.2%**	**-3.34 [-4.28, -2.39]**
Heterogeneity: Tau² = 8.37; Chi² = 262.62, df = 68 (P < 0.00001); I² = 74%								
Test for overall effect: Z = 6.93 (P < 0.00001)								
**Total (95% CI)**			**3163**			**2279**	**100.0%**	**-2.86 [-3.52, -2.20]**
Heterogeneity: Tau² = 5.91; Chi² = 306.04, df = 109 (P < 0.00001); I² = 64%								
Test for overall effect: Z = 8.45 (P < 0.00001)								
Test for subgroup differences: Chi² = 5.43, df = 1 (P = 0.02), I² = 81.6%								

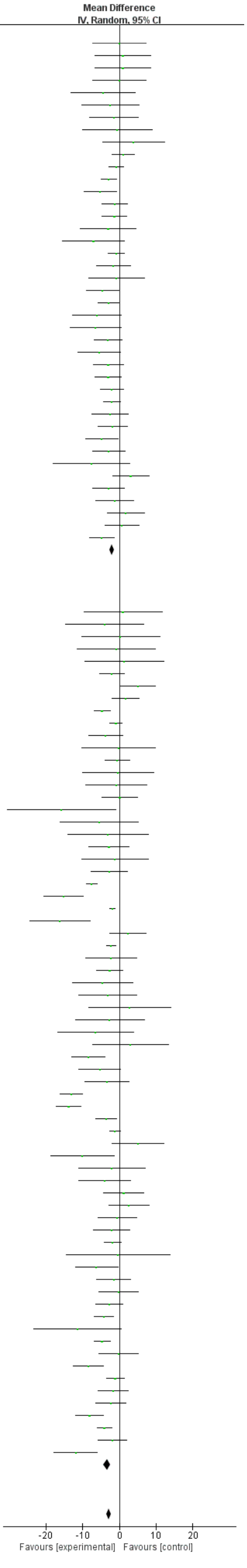

Figure S3.4. Forest plot of subgroup analyses by exercise frequency

Study or Subgroup	Experimental Mean	Experimental SD	Experimental Total	Control Mean	Control SD	Control Total	Weight	Mean Difference IV, Random, 95% CI
**2.4.1 <3 sessions per week**								
Benson 2008	75.2	11.9	31	70.1	6.7	36	1.0%	5.10 [0.37, 9.83]
Boer 2014(1)	91.5	13.1	17	95.9	8.2	7	0.4%	-4.40 [-13.10, 4.30]
Boer 2014(2)	93.4	9.6	15	95.9	8.2	7	0.5%	-2.50 [-10.28, 5.28]
Boff 2020	100.51	11.17	34	101.96	15.38	31	0.7%	-1.45 [-8.04, 5.14]
Espinoza-Silva 2019(1)	61.77	8.12	69	66.93	10.85	30	1.1%	-5.16 [-9.49, -0.83]
Espinoza-Silva 2019(2)	71.5	9.71	141	72.74	9.24	34	1.3%	-1.24 [-4.73, 2.25]
Faria 2020(1)	76	4.97	24	76.5	5.08	13	1.3%	-0.50 [-3.90, 2.90]
Faria 2020(2)	75.2	5.08	26	76.5	5.08	13	1.3%	-1.30 [-4.68, 2.08]
Farpour-Lambert 2019(1)	79.63	12.55	19	80	12	9	0.4%	-0.37 [-10.03, 9.29]
Farpour-Lambert 2019(2)	79.16	10.61	28	80	12	10	0.5%	-0.84 [-9.25, 7.57]
Harder-Lauridsen 2014	75.9	8.93	18	78.7	7.63	17	0.8%	-2.80 [-8.29, 2.69]
Lambrick 2016	70.9	8.7	15	71.6	11.9	14	0.5%	-0.70 [-8.33, 6.93]
Lofrano-Prado 2022	102.6	12.7	37	105.9	13.5	37	0.8%	-3.30 [-9.27, 2.67]
Pena 2022	107	17.78	79	111	18.49	38	0.6%	-4.00 [-11.07, 3.07]
Plavsic 2020	91.5	7.8	22	96.9	11.4	22	0.8%	-5.40 [-11.17, 0.37]
Tjonna 2009	104.9	7.6	20	104.2	7.5	22	1.0%	0.70 [-3.87, 5.27]
Zehsaz 2016	85.3	3.1	16	89.34	2.7	16	1.6%	-4.04 [-6.05, -2.03]
Zehsaz 2017	83.9	4.9	16	85.8	6.1	16	1.2%	-1.90 [-5.73, 1.93]
**Subtotal (95% CI)**			**627**			**372**	**15.7%**	**-1.99 [-3.17, -0.81]**
Heterogeneity: Tau² = 0.81; Chi² = 19.54, df = 17 (P = 0.30); I² = 13%								
Test for overall effect: Z = 3.30 (P = 0.0010)								
**2.4.2 ⩾3 sessions per week**								
Abassi 2020(1)	103	8.84	8	102	8.93	4	0.3%	1.00 [-9.68, 11.68]
Abassi 2020(2)	98	8.59	8	102	8.93	4	0.3%	-4.00 [-14.58, 6.58]
Abassi 2022(1)	101	8.51	13	101	6.99	6	0.6%	0.00 [-7.26, 7.26]
Abassi 2022(2)	102	9.43	13	101	6.99	6	0.5%	1.00 [-6.59, 8.59]
Abassi 2023(1)	102	9.43	13	101	6.99	6	0.5%	1.00 [-6.59, 8.59]
Abassi 2023(2)	101	8.51	13	101	6.99	6	0.6%	0.00 [-7.26, 7.26]
Ahmadi 2020(1)	93.53	12.91	28	93.2	14.55	9	0.3%	0.33 [-10.31, 10.97]
Ahmadi 2020(2)	92.32	13.3	29	93.2	14.55	9	0.3%	-0.88 [-11.55, 9.79]
Ahmadi 2020(3)	94.58	13.9	29	93.2	14.55	9	0.3%	1.38 [-9.39, 12.15]
Barbeau 2007	67.9	11.3	118	69.9	12.5	83	1.3%	-2.00 [-5.37, 1.37]
Bezerra 2023	68.7	7.35	20	67.02	4.14	21	1.2%	1.68 [-2.00, 5.36]
Bharath 2018	83.5	2.24	20	88.2	4.47	20	1.6%	-4.70 [-6.89, -2.51]
Chuensiri 2018(1)	84.2	8.95	11	84.7	8.95	5	0.4%	-0.50 [-9.96, 8.96]
Chuensiri 2018(2)	88.6	8.91	15	84.7	8.95	6	0.5%	3.90 [-4.56, 12.36]
CROKER 2012	89.58	3.28	33	90.48	3.31	30	1.7%	-0.90 [-2.53, 0.73]
Davis 2020	79.1	9.38	90	78	11.34	85	1.4%	1.10 [-1.99, 4.19]
Dennis 2013(1)	76.9	3.73	34	77.7	3.82	21	1.6%	-0.80 [-2.86, 1.26]
Dennis 2013(2)	74.8	3.9	36	77.7	3.82	21	1.6%	-2.90 [-4.97, -0.83]
Duft 2020	89.29	7.46	18	92.99	7.14	19	1.0%	-3.70 [-8.41, 1.01]
Elmahgoub 2009	96.18	17.82	15	96.39	8.62	15	0.4%	-0.21 [-10.23, 9.81]
Guo 2015	77.77	7.24	23	77.6	6.82	12	0.9%	0.17 [-4.69, 5.03]
Hamila 2018(1)	75.9	11.7	7	91.6	8.6	2	0.2%	-15.70 [-30.44, -0.96]
Hamila 2018(2)	86.1	6.5	8	91.6	8.6	3	0.3%	-5.50 [-16.22, 5.22]
Hamila 2018(3)	88.5	7.4	8	91.6	8.6	3	0.3%	-3.10 [-14.10, 7.90]
Hay 2016(1)	106	8.9	32	109	15	18	0.5%	-3.00 [-10.58, 4.58]
Hay 2016(2)	102	15	38	109	15	18	0.5%	-7.00 [-15.41, 1.41]
Heidarianpour 2023	68.29	12.96	15	69.47	12.18	15	0.4%	-1.18 [-10.18, 7.82]
Jain 2022	85.7	11.5	41	88.4	9.3	28	0.9%	-2.70 [-7.63, 2.23]
Jian 2014	76.31	3.48	44	83.87	3.62	44	1.8%	-7.56 [-9.04, -6.08]
Jianjun 2012	92.46	8.36	20	107.64	7.76	15	0.9%	-15.18 [-20.55, -9.81]
Kai 2024	85.1	0.8	20	87	1.8	24	1.9%	-1.90 [-2.70, -1.10]
Ke 2024	84	10.4	10	100.1	8.3	10	0.5%	-16.10 [-24.35, -7.85]
Kelishadi 2008	60.2	5.38	45	61	5.23	47	1.6%	-0.80 [-2.97, 1.37]
Kim 2007	89.6	7.48	14	91.2	4.16	12	1.0%	-1.60 [-6.17, 2.97]
Kim 2011	87.1	6.58	12	84.8	5.06	10	0.9%	2.30 [-2.57, 7.17]
Kim 2020	85.5	2.7	24	87.7	1.8	24	1.8%	-2.20 [-3.50, -0.90]
Kim 2022	84.2	7.5	13	86.5	10.4	13	0.6%	-2.30 [-9.27, 4.67]
Larsen 2016	79.4	8.6	51	82	8.8	43	1.3%	-2.60 [-6.13, 0.93]
Lee 2010(1)	78.29	9.72	20	82.79	10.7	9	0.5%	-4.50 [-12.69, 3.69]
Lee 2010(2)	79.63	7.73	16	82.79	10.7	9	0.5%	-3.16 [-11.11, 4.79]
Lee 2012(1)	103.3	12.28	15	100.5	10.61	5	0.3%	2.80 [-8.39, 13.99]
Lee 2012(2)	97.9	8	16	100.5	10.61	6	0.4%	-2.60 [-11.95, 6.75]
Lee 2013(1)	104.11	10.97	14	110.53	8.76	4	0.3%	-6.42 [-16.75, 3.91]
Lee 2013(2)	113.58	11.26	14	110.53	8.76	4	0.3%	3.05 [-7.37, 13.47]
Liangmei 2015	93	5.61	12	101.38	5.58	12	1.0%	-8.38 [-12.86, -3.90]
Lingling 2021	89.7	7	20	94.2	7.5	20	1.0%	-4.50 [-9.00, -0.00]
Liqun 2014	66.33	9.21	76	69.27	9.29	97	1.4%	-2.94 [-5.72, -0.16]
Liu 2018	95.5	8.2	30	100.8	11.1	20	0.8%	-5.30 [-10.98, 0.38]
Luo 2013(1)	82.29	7.7	43	95.37	5.81	34	1.4%	-13.08 [-16.10, -10.06]
Luo 2013(2)	84.34	7.47	52	98.07	8.4	38	1.3%	-13.73 [-17.08, -10.38]
Maddison 2011	84.4	10.8	115	88	10.7	116	1.4%	-3.60 [-6.37, -0.83]
Meng 2022(1)	78.8	6.1	12	84.9	7.2	6	0.6%	-6.10 [-12.82, 0.62]
Meng 2022(2)	78.5	7.5	11	84.9	7.2	7	0.6%	-6.40 [-13.33, 0.53]
Migueles 2023	91.85	3.75	47	93.02	3.74	45	1.8%	-1.17 [-2.70, 0.36]
Mohammadi 2019	80.1	9.6	10	75	6.3	10	0.6%	5.10 [-2.02, 12.22]
Monteiro 2015(1)	85.52	6.08	18	95.52	11.8	8	0.5%	-10.00 [-18.65, -1.35]
Monteiro 2015(2)	93.52	7.55	14	95.52	11.8	8	0.4%	-2.00 [-11.08, 7.08]
Park 2007	81.9	6.6	19	85	5.6	21	1.2%	-3.10 [-6.91, 0.71]
Racil(1) 2016(1)	91	6	17	94	4	7	1.1%	-3.00 [-7.11, 1.11]
Racil(1) 2016(2)	91	4	16	94	4	7	1.2%	-3.00 [-6.55, 0.55]
Racil(2) 2016(1)	90	6	23	92	3	9	1.4%	-2.00 [-5.14, 1.14]
Racil(2) 2016(2)	90	4	26	92	3	10	1.5%	-2.00 [-4.41, 0.41]
Racil 2013(1)	90.3	6.7	11	92.8	3.7	6	0.9%	-2.50 [-7.44, 2.44]
Racil 2013(2)	91	4.6	11	92.8	3.7	6	1.1%	-1.80 [-5.82, 2.22]
Racil 2024(1)	90.12	3.9	12	94.83	4.3	5	1.1%	-4.71 [-9.08, -0.34]
Racil 2024(2)	92	4.9	12	94.83	4.3	6	1.0%	-2.83 [-7.25, 1.59]
Regaieg 2013	79.8	6.2	14	78.6	8.5	14	0.8%	1.20 [-4.31, 6.71]
Salus(1) 2022	96.3	8.98	14	103.9	17.96	14	0.3%	-7.60 [-18.12, 2.92]
Seo 2019	96.32	9.68	26	93.67	13.97	44	0.8%	2.65 [-2.91, 8.21]
Shalitin 2009	78.7	14.1	55	79.3	14.1	55	0.9%	-0.60 [-5.87, 4.67]
Shu 2023(1)	86	6.9	16	82.9	5.2	8	0.9%	3.10 [-1.84, 8.04]
Shu 2023(2)	80	4.7	15	82.9	5.2	8	1.1%	-2.90 [-7.22, 1.42]
Sigal 2014(1)	93.6	11.26	75	94.9	11.33	25	0.9%	-1.30 [-6.42, 3.82]
Sigal 2014(2)	96.7	11.48	78	94.9	11.33	25	0.9%	1.80 [-3.32, 6.92]
Sigal 2014(3)	92.8	11.26	75	94.9	11.33	26	0.9%	-2.10 [-7.15, 2.95]
Son 2017	84.5	3.13	20	86.3	4.47	20	1.5%	-1.80 [-4.19, 0.59]
Staiano 2017	107.8	21.82	20	108.2	22.51	18	0.2%	-0.40 [-14.53, 13.73]
Suarez-Villadat 2020	15.34	9.18	15	21.52	9.85	30	0.8%	-6.18 [-12.01, -0.35]
Suarez-Villadat 2023	73.29	7.93	24	74.77	8.61	25	1.0%	-1.48 [-6.11, 3.15]
Sun 2011	81.9	8.5	25	82.1	8.9	17	0.9%	-0.20 [-5.59, 5.19]
Tan 2010	79	7.2	30	81.7	7.2	30	1.2%	-2.70 [-6.34, 0.94]
Toulabi 2012	96.23	9.5	76	100.4	6.99	76	1.5%	-4.17 [-6.82, -1.52]
Vasconcellos 2016	90.5	8.9	10	101.9	17	10	0.3%	-11.40 [-23.29, 0.49]
Wong 2018	83.5	1.94	15	88.2	3.87	15	1.6%	-4.70 [-6.89, -2.51]
Xianbo 2011	81.9	8.5	25	82.1	8.9	17	0.9%	-0.20 [-5.59, 5.19]
Xinhui 2016	93.01	5.62	14	101.39	5.59	14	1.1%	-8.38 [-12.53, -4.23]
Yanqin 2017	83.32	7.14	35	88.12	6.85	33	1.3%	-4.80 [-8.13, -1.47]
Yu 2020	84	10.1	99	85.1	5.8	72	1.5%	-1.10 [-3.50, 1.30]
Yuanyuan 2019(1)	79.56	3.35	10	81.22	3.21	3	1.1%	-1.66 [-5.84, 2.52]
Yuanyuan 2019(2)	78.92	3.36	10	81.22	3.21	3	1.1%	-2.30 [-6.49, 1.89]
Yuanyuan 2019(3)	73.14	3.24	9	81.22	3.21	4	1.2%	-8.08 [-11.87, -4.29]
Zhihui 2017	89.38	12.45	40	101.21	14.43	40	0.8%	-11.83 [-17.74, -5.92]
**Subtotal (95% CI)**			**2536**			**1907**	**84.3%**	**-3.04 [-3.79, -2.30]**
Heterogeneity: Tau² = 6.54; Chi² = 284.98, df = 91 (P < 0.00001); I² = 68%								
Test for overall effect: Z = 8.01 (P < 0.00001)								
**Total (95% CI)**			**3163**			**2279**	**100.0%**	**-2.86 [-3.52, -2.20]**
Heterogeneity: Tau² = 5.91; Chi² = 306.04, df = 109 (P < 0.00001); I² = 64%								
Test for overall effect: Z = 8.45 (P < 0.00001)								
Test for subgroup differences: Chi² = 2.19, df = 1 (P = 0.14), I² = 54.3%								

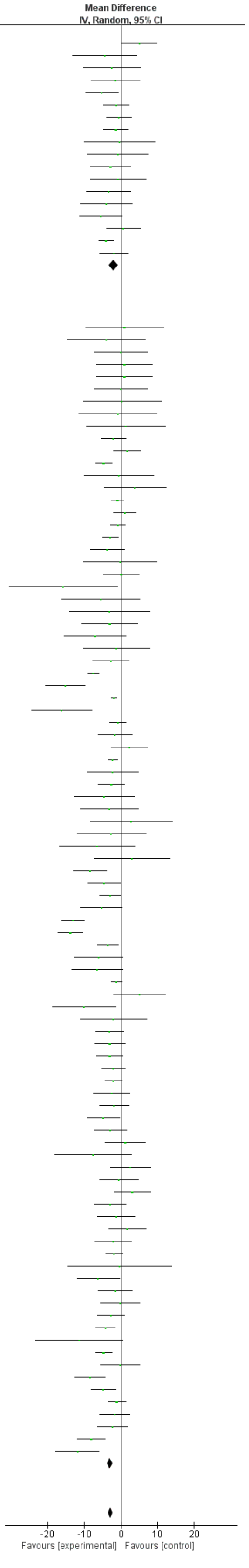

Figure S3.5. Forest plot of subgroup analyses by weekly exercise duration

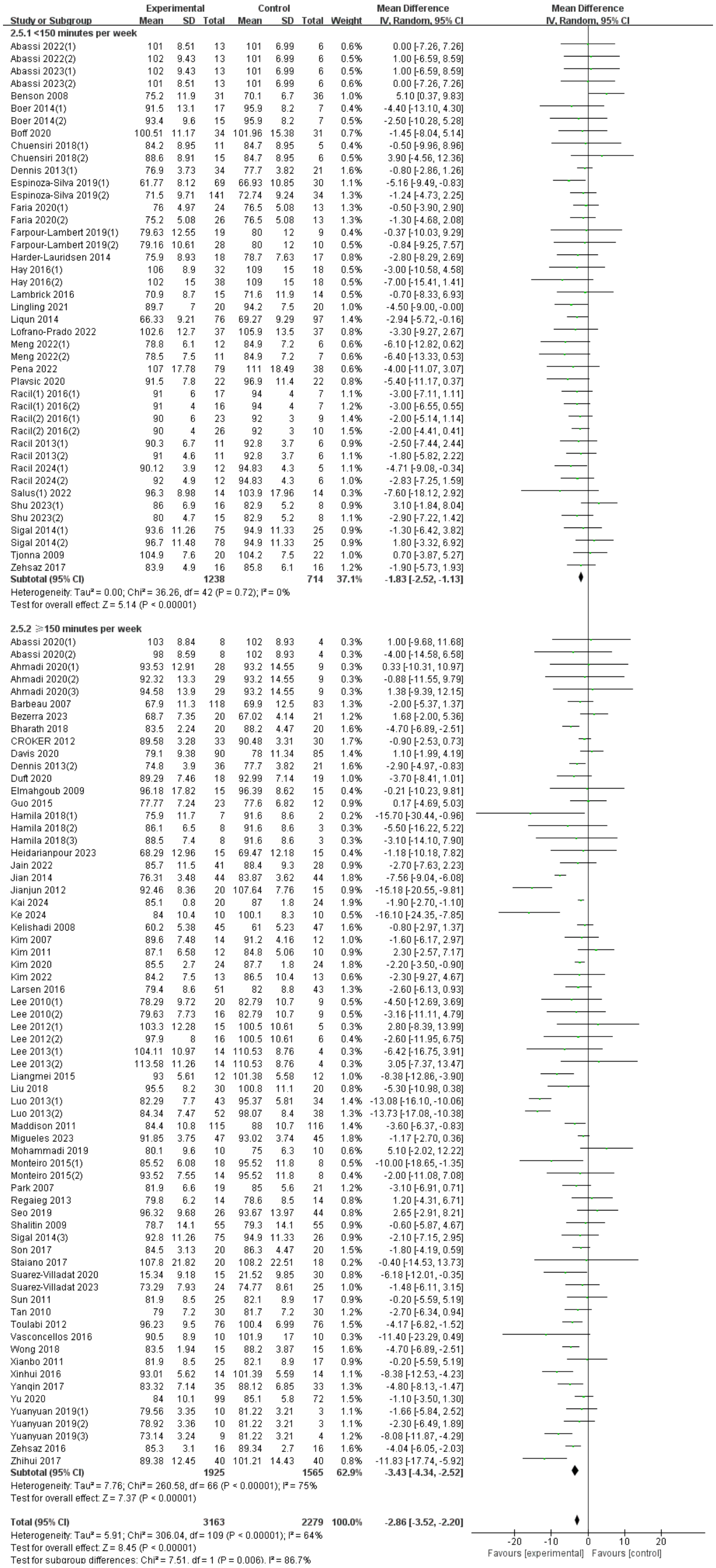

Figure S3.6. Forest plot of subgroup analyses by intervention duration

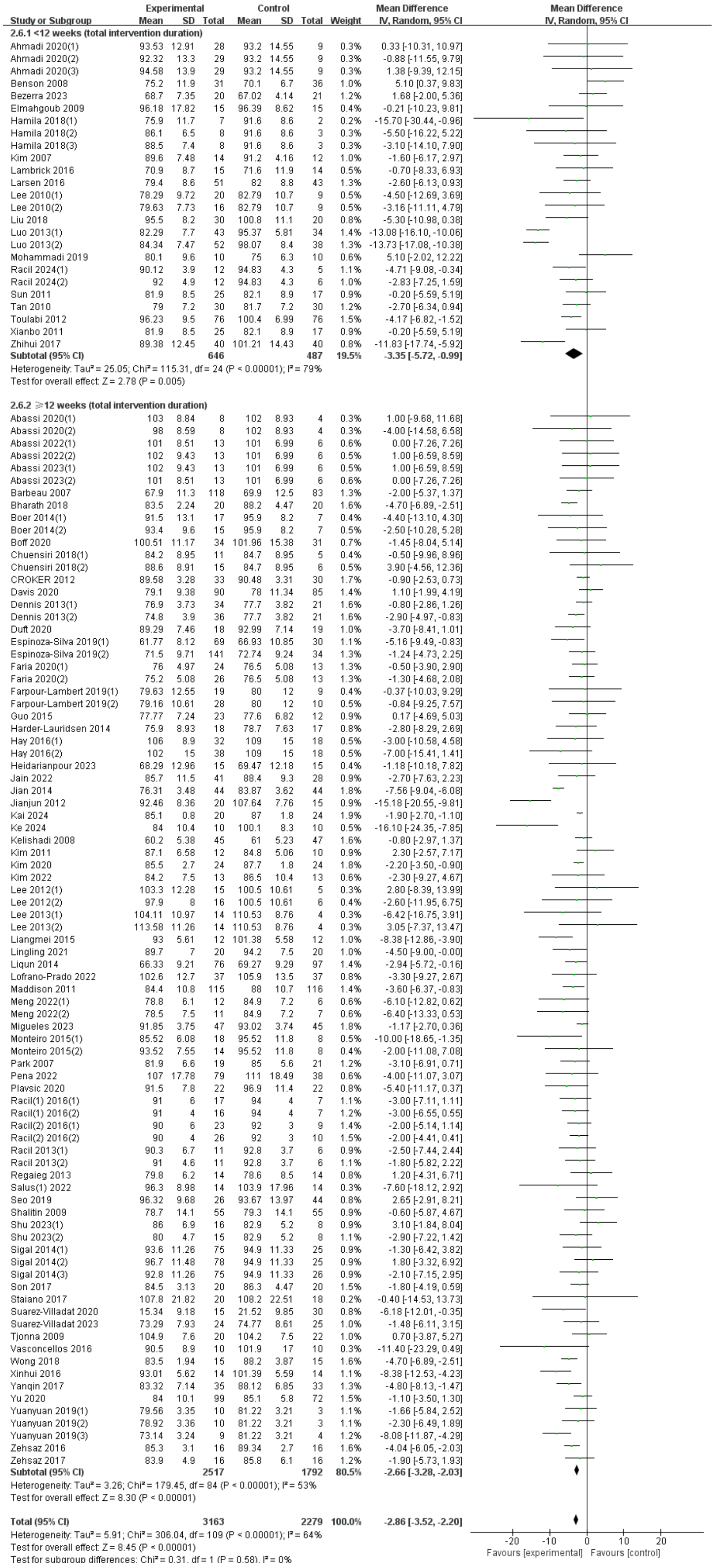

Figure S3.7. Forest plot of subgroup analyses by region

Study or Subgroup	Experimental Mean	Experimental SD	Experimental Total	Control Mean	Control SD	Control Total	Weight	Mean Difference IV, Random, 95% CI
**2.7.1 Asia**								
Ahmadi 2020(1)	93.53	12.91	28	93.2	14.55	9	0.3%	0.33 [-10.31, 10.97]
Ahmadi 2020(2)	92.32	13.3	29	93.2	14.55	9	0.3%	-0.88 [-11.55, 9.79]
Ahmadi 2020(3)	94.58	13.9	29	93.2	14.55	9	0.3%	1.38 [-9.39, 12.15]
Chuensiri 2018(1)	84.2	8.95	11	84.7	8.95	5	0.4%	-0.50 [-9.96, 8.96]
Chuensiri 2018(2)	88.6	8.91	15	84.7	8.95	6	0.5%	3.90 [-4.56, 12.36]
Guo 2015	77.77	7.24	23	77.6	6.82	12	0.9%	0.17 [-4.69, 5.03]
Heidarianpour 2023	68.29	12.96	15	69.47	12.18	15	0.4%	-1.18 [-10.18, 7.82]
Jain 2022	85.7	11.5	41	88.4	9.3	28	0.9%	-2.70 [-7.63, 2.23]
Jian 2014	76.31	3.48	44	83.87	3.62	44	1.8%	-7.56 [-9.04, -6.08]
Jianjun 2012	92.46	8.36	20	107.64	7.76	15	0.9%	-15.18 [-20.55, -9.81]
Kai 2024	85.1	0.8	20	87	1.8	24	1.9%	-1.90 [-2.70, -1.10]
Ke 2024	84	10.4	10	100.1	8.3	10	0.5%	-16.10 [-24.35, -7.85]
Kelishadi 2008	60.2	5.38	45	61	5.23	47	1.6%	-0.80 [-2.97, 1.37]
Kim 2007	89.6	7.48	14	91.2	4.16	12	1.0%	-1.60 [-6.17, 2.97]
Kim 2011	87.1	6.58	12	84.8	5.06	10	0.9%	2.30 [-2.57, 7.17]
Kim 2022	84.2	7.5	13	86.5	10.4	13	0.6%	-2.30 [-9.27, 4.67]
Lee 2010(1)	78.29	9.72	20	82.79	10.7	9	0.5%	-4.50 [-12.69, 3.69]
Lee 2010(2)	79.63	7.73	16	82.79	10.7	9	0.5%	-3.16 [-11.11, 4.79]
Liangmei 2015	93	5.61	12	101.38	5.58	12	1.0%	-8.38 [-12.86, -3.90]
Lingling 2021	89.7	7	20	94.2	7.5	20	1.0%	-4.50 [-9.00, -0.00]
Liqun 2014	66.33	9.21	76	69.27	9.29	97	1.4%	-2.94 [-5.72, -0.16]
Liu 2018	95.5	8.2	30	100.8	11.1	20	0.8%	-5.30 [-10.98, 0.38]
Luo 2013(1)	82.29	7.7	43	95.37	5.81	34	1.4%	-13.08 [-16.10, -10.06]
Luo 2013(2)	84.34	7.47	52	98.07	8.4	38	1.3%	-13.73 [-17.08, -10.38]
Meng 2022(1)	78.8	6.1	12	84.9	7.2	6	0.6%	-6.10 [-12.82, 0.62]
Meng 2022(2)	78.5	7.5	11	84.9	7.2	7	0.6%	-6.40 [-13.33, 0.53]
Mohammadi 2019	80.1	9.6	10	75	6.3	10	0.6%	5.10 [-2.02, 12.22]
Park 2007	81.9	6.6	19	85	5.6	21	1.2%	-3.10 [-6.91, 0.71]
Seo 2019	96.32	9.68	26	93.67	13.97	44	0.8%	2.65 [-2.91, 8.21]
Shalitin 2009	78.7	14.1	55	79.3	14.1	55	0.9%	-0.60 [-5.87, 4.67]
Shu 2023(1)	86	6.9	16	82.9	5.2	8	0.9%	3.10 [-1.84, 8.04]
Shu 2023(2)	80	4.7	15	82.9	5.2	8	1.1%	-2.90 [-7.22, 1.42]
Son 2017	84.5	3.13	20	86.3	4.47	20	1.5%	-1.80 [-4.19, 0.59]
Sun 2011	81.9	8.5	25	82.1	8.9	17	0.9%	-0.20 [-5.59, 5.19]
Tan 2010	79	7.2	30	81.7	7.2	30	1.2%	-2.70 [-6.34, 0.94]
Toulabi 2012	96.23	9.5	76	100.4	6.99	76	1.5%	-4.17 [-6.82, -1.52]
Xianbo 2011	81.9	8.5	25	82.1	8.9	17	0.9%	-0.20 [-5.59, 5.19]
Xinhui 2016	93.01	5.62	14	101.39	5.59	14	1.1%	-8.38 [-12.53, -4.23]
Yanqin 2017	83.32	7.14	35	88.12	6.85	33	1.3%	-4.80 [-8.13, -1.47]
Yu 2020	84	10.1	99	85.1	5.8	72	1.5%	-1.10 [-3.50, 1.30]
Yuanyuan 2019(1)	79.56	3.35	10	81.22	3.21	3	1.1%	-1.66 [-5.84, 2.52]
Yuanyuan 2019(2)	78.92	3.36	10	81.22	3.21	3	1.1%	-2.30 [-6.49, 1.89]
Yuanyuan 2019(3)	73.14	3.24	9	81.22	3.21	4	1.2%	-8.08 [-11.87, -4.29]
Zehsaz 2016	85.3	3.1	16	89.34	2.7	16	1.6%	-4.04 [-6.05, -2.03]
Zehsaz 2017	83.9	4.9	16	85.8	6.1	16	1.2%	-1.90 [-5.73, 1.93]
Zhihui 2017	89.38	12.45	40	101.21	14.43	40	0.8%	-11.83 [-17.74, -5.92]
**Subtotal (95% CI)**			**1227**			**1027**	**44.9%**	**-3.74 [-4.96, -2.51]**
Heterogeneity: Tau² = 11.44; Chi² = 220.51, df = 45 (P < 0.00001); I² = 80%								
Test for overall effect: Z = 5.98 (P < 0.00001)								
**2.7.2 Europe**								
Boer 2014(1)	91.5	13.1	17	95.9	8.2	7	0.4%	-4.40 [-13.10, 4.30]
Boer 2014(2)	93.4	9.6	15	95.9	8.2	7	0.5%	-2.50 [-10.28, 5.28]
CROKER 2012	89.58	3.28	33	90.48	3.31	30	1.7%	-0.90 [-2.53, 0.73]
Elmahgoub 2009	96.18	17.82	15	96.39	8.62	15	0.4%	-0.21 [-10.23, 9.81]
Farpour-Lambert 2019(1)	79.63	12.55	19	80	12	9	0.4%	-0.37 [-10.03, 9.29]
Farpour-Lambert 2019(2)	79.16	10.61	28	80	12	10	0.5%	-0.84 [-9.25, 7.57]
Harder-Lauridsen 2014	75.9	8.93	18	78.7	7.63	17	0.8%	-2.80 [-8.29, 2.69]
Lambrick 2016	70.9	8.7	15	71.6	11.9	14	0.5%	-0.70 [-8.33, 6.93]
Larsen 2016	79.4	8.6	51	82	8.8	43	1.3%	-2.60 [-6.13, 0.93]
Migueles 2023	91.85	3.75	47	93.02	3.74	45	1.8%	-1.17 [-2.70, 0.36]
Plavsic 2020	91.5	7.8	22	96.9	11.4	22	0.8%	-5.40 [-11.17, 0.37]
Salus(1) 2022	96.3	8.98	14	103.9	17.96	14	0.3%	-7.60 [-18.12, 2.92]
Suarez-Villadat 2020	15.34	9.18	15	21.52	9.85	30	0.8%	-6.18 [-12.01, -0.35]
Suarez-Villadat 2023	73.29	7.93	24	74.77	8.61	25	1.0%	-1.48 [-6.11, 3.15]
Tjonna 2009	104.9	7.6	20	104.2	7.5	22	1.0%	0.70 [-3.87, 5.27]
**Subtotal (95% CI)**			**353**			**310**	**12.2%**	**-1.48 [-2.41, -0.55]**
Heterogeneity: Tau² = 0.00; Chi² = 8.37, df = 14 (P = 0.87); I² = 0%								
Test for overall effect: Z = 3.12 (P = 0.002)								
**2.7.3 Africa**								
Abassi 2020(1)	103	8.84	8	102	8.93	4	0.3%	1.00 [-9.68, 11.68]
Abassi 2020(2)	98	8.59	8	102	8.93	4	0.3%	-4.00 [-14.58, 6.58]
Abassi 2022(1)	101	8.51	13	101	6.99	6	0.6%	0.00 [-7.26, 7.26]
Abassi 2022(2)	102	9.43	13	101	6.99	6	0.5%	1.00 [-6.59, 8.59]
Abassi 2023(1)	102	9.43	13	101	6.99	6	0.5%	1.00 [-6.59, 8.59]
Abassi 2023(2)	101	8.51	13	101	6.99	6	0.6%	0.00 [-7.26, 7.26]
Hamila 2018(1)	75.9	11.7	7	91.6	8.6	2	0.2%	-15.70 [-30.44, -0.96]
Hamila 2018(2)	86.1	6.5	8	91.6	8.6	3	0.3%	-5.50 [-16.22, 5.22]
Hamila 2018(3)	88.5	7.4	8	91.6	8.6	3	0.3%	-3.10 [-14.10, 7.90]
Racil(1) 2016(1)	91	6	17	94	4	7	1.1%	-3.00 [-7.11, 1.11]
Racil(1) 2016(2)	91	4	16	94	4	7	1.2%	-3.00 [-6.55, 0.55]
Racil(2) 2016(1)	90	6	23	92	3	9	1.4%	-2.00 [-5.14, 1.14]
Racil(2) 2016(2)	90	4	26	92	3	10	1.5%	-2.00 [-4.41, 0.41]
Racil 2013(1)	90.3	6.7	11	92.8	3.7	6	0.9%	-2.50 [-7.44, 2.44]
Racil 2013(2)	91	4.6	11	92.8	3.7	6	1.1%	-1.80 [-5.82, 2.22]
Racil 2024(1)	90.12	3.9	12	94.83	4.3	5	1.1%	-4.71 [-9.08, -0.34]
Racil 2024(2)	92	4.9	12	94.83	4.3	6	1.0%	-2.83 [-7.25, 1.59]
Regaieg 2013	79.8	6.2	14	78.6	8.5	14	0.8%	1.20 [-4.31, 6.71]
**Subtotal (95% CI)**			**233**			**110**	**14.0%**	**-2.18 [-3.33, -1.04]**
Heterogeneity: Tau² = 0.00; Chi² = 9.38, df = 17 (P = 0.93); I² = 0%								
Test for overall effect: Z = 3.74 (P = 0.0002)								
**2.7.4 North America**								
Barbeau 2007	67.9	11.3	118	69.9	12.5	83	1.3%	-2.00 [-5.37, 1.37]
Bharath 2018	83.5	2.24	20	88.2	4.47	20	1.6%	-4.70 [-6.89, -2.51]
Davis 2020	79.1	9.38	90	78	11.34	85	1.4%	1.10 [-1.99, 4.19]
Dennis 2013(1)	76.9	3.73	34	77.7	3.82	21	1.6%	-0.80 [-2.86, 1.26]
Dennis 2013(2)	74.8	3.9	36	77.7	3.82	21	1.6%	-2.90 [-4.97, -0.83]
Hay 2016(1)	106	8.9	32	109	15	18	0.5%	-3.00 [-10.58, 4.58]
Hay 2016(2)	102	15	38	109	15	18	0.5%	-7.00 [-15.41, 1.41]
Kim 2020	85.5	2.7	24	87.7	1.8	24	1.8%	-2.20 [-3.50, -0.90]
Lee 2012(1)	103.3	12.28	15	100.5	10.61	5	0.3%	2.80 [-8.39, 13.99]
Lee 2012(2)	97.9	8	16	100.5	10.61	6	0.4%	-2.60 [-11.95, 6.75]
Lee 2013(1)	104.11	10.97	14	110.53	8.76	4	0.3%	-6.42 [-16.75, 3.91]
Lee 2013(2)	113.58	11.26	14	110.53	8.76	4	0.3%	3.05 [-7.37, 13.47]
Pena 2022	107	17.78	79	111	18.49	38	0.6%	-4.00 [-11.07, 3.07]
Sigal 2014(1)	93.6	11.26	75	94.9	11.33	25	0.9%	-1.30 [-6.42, 3.82]
Sigal 2014(2)	96.7	11.48	78	94.9	11.33	25	0.9%	1.80 [-3.32, 6.92]
Sigal 2014(3)	92.8	11.26	75	94.9	11.33	26	0.9%	-2.10 [-7.15, 2.95]
Staiano 2017	107.8	21.82	20	108.2	22.51	18	0.2%	-0.40 [-14.53, 13.73]
Wong 2018	83.5	1.94	15	88.2	3.87	15	1.6%	-4.70 [-6.89, -2.51]
**Subtotal (95% CI)**			**793**			**456**	**16.8%**	**-2.33 [-3.32, -1.33]**
Heterogeneity: Tau² = 0.95; Chi² = 22.76, df = 17 (P = 0.16); I² = 25%								
Test for overall effect: Z = 4.59 (P < 0.00001)								
**2.7.5 South America**								
Bezerra 2023	68.7	7.35	20	67.02	4.14	21	1.2%	1.68 [-2.00, 5.36]
Boff 2020	100.51	11.17	34	101.96	15.38	31	0.7%	-1.45 [-8.04, 5.14]
Duft 2020	89.29	7.46	18	92.99	7.14	19	1.0%	-3.70 [-8.41, 1.01]
Espinoza-Silva 2019(1)	61.77	8.12	69	66.93	10.85	30	1.1%	-5.16 [-9.49, -0.83]
Espinoza-Silva 2019(2)	71.5	9.71	141	72.74	9.24	34	1.3%	-1.24 [-4.73, 2.25]
Faria 2020(1)	76	4.97	24	76.5	5.08	13	1.3%	-0.50 [-3.90, 2.90]
Faria 2020(2)	75.2	5.08	26	76.5	5.08	13	1.3%	-1.30 [-4.68, 2.08]
Lofrano-Prado 2022	102.6	12.7	37	105.9	13.5	37	0.8%	-3.30 [-9.27, 2.67]
Monteiro 2015(1)	85.52	6.08	18	95.52	11.8	8	0.5%	-10.00 [-18.65, -1.35]
Monteiro 2015(2)	93.52	7.55	14	95.52	11.8	8	0.4%	-2.00 [-11.08, 7.08]
Vasconcellos 2016	90.5	8.9	10	101.9	17	10	0.3%	-11.40 [-23.29, 0.49]
**Subtotal (95% CI)**			**411**			**224**	**9.7%**	**-2.08 [-3.77, -0.39]**
Heterogeneity: Tau² = 1.95; Chi² = 13.34, df = 10 (P = 0.21); I² = 25%								
Test for overall effect: Z = 2.41 (P = 0.02)								
**2.7.6 Oceania**								
Benson 2008	75.2	11.9	31	70.1	6.7	36	1.0%	5.10 [0.37, 9.83]
Maddison 2011	84.4	10.8	115	88	10.7	116	1.4%	-3.60 [-6.37, -0.83]
**Subtotal (95% CI)**			**146**			**152**	**2.4%**	**0.53 [-7.98, 9.05]**
Heterogeneity: Tau² = 33.94; Chi² = 9.68, df = 1 (P = 0.002); I² = 90%								
Test for overall effect: Z = 0.12 (P = 0.90)								
**Total (95% CI)**			**3163**			**2279**	**100.0%**	**-2.86 [-3.52, -2.20]**
Heterogeneity: Tau² = 5.91; Chi² = 306.04, df = 109 (P < 0.00001); I² = 64%								
Test for overall effect: Z = 8.45 (P < 0.00001)								
Test for subgroup differences: Chi² = 8.81, df = 5 (P = 0.12), I² = 43.2%								

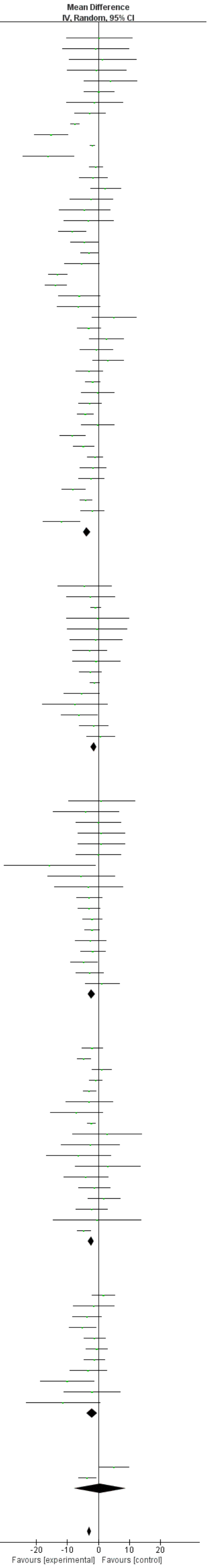

Figure S3.8. Forest plot of subgroup analyses by economic status

Study or Subgroup	Experimental Mean	Experimental SD	Experimental Total	Control Mean	Control SD	Control Total	Weight	Mean Difference IV, Random, 95% CI
**2.8.1 Lower-middle-income countries**								
Abassi 2020(1)	103	8.84	8	102	8.93	4	0.3%	1.00 [-9.68, 11.68]
Abassi 2020(2)	98	8.59	8	102	8.93	4	0.3%	-4.00 [-14.58, 6.58]
Abassi 2022(1)	101	8.51	13	101	6.99	6	0.6%	0.00 [-7.26, 7.26]
Abassi 2022(2)	102	9.43	13	101	6.99	6	0.5%	1.00 [-6.59, 8.59]
Abassi 2023(1)	102	9.43	13	101	6.99	6	0.5%	1.00 [-6.59, 8.59]
Abassi 2023(2)	101	8.51	13	101	6.99	6	0.6%	0.00 [-7.26, 7.26]
Hamila 2018(1)	75.9	11.7	7	91.6	8.6	2	0.2%	-15.70 [-30.44, -0.96]
Hamila 2018(2)	86.1	6.5	8	91.6	8.6	3	0.3%	-5.50 [-16.22, 5.22]
Hamila 2018(3)	88.5	7.4	8	91.6	8.6	3	0.3%	-3.10 [-14.10, 7.90]
Jain 2022	85.7	11.5	41	88.4	9.3	28	0.9%	-2.70 [-7.63, 2.23]
Racil(1) 2016(1)	91	6	17	94	4	7	1.1%	-3.00 [-7.11, 1.11]
Racil(1) 2016(2)	91	4	16	94	4	7	1.2%	-3.00 [-6.55, 0.55]
Racil(2) 2016(1)	90	6	23	92	3	9	1.4%	-2.00 [-5.14, 1.14]
Racil(2) 2016(2)	90	4	26	92	3	10	1.5%	-2.00 [-4.41, 0.41]
Racil 2013(1)	90.3	6.7	11	92.8	3.7	6	0.9%	-2.50 [-7.44, 2.44]
Racil 2013(2)	91	4.6	11	92.8	3.7	6	1.1%	-1.80 [-5.82, 2.22]
Racil 2024(1)	90.12	3.9	12	94.83	4.3	5	1.1%	-4.71 [-9.08, -0.34]
Racil 2024(2)	92	4.9	12	94.83	4.3	6	1.0%	-2.83 [-7.25, 1.59]
Regaieg 2013	79.8	6.2	14	78.6	8.5	14	0.8%	1.20 [-4.31, 6.71]
**Subtotal (95% CI)**			**274**			**138**	**14.9%**	**-2.21 [-3.32, -1.09]**
Heterogeneity: Tau² = 0.00; Chi² = 9.42, df = 18 (P = 0.95); I² = 0%								
Test for overall effect: Z = 3.88 (P = 0.0001)								
**2.8.2 Upper-middle-income countries**								
Ahmadi 2020(1)	93.53	12.91	28	93.2	14.55	9	0.3%	0.33 [-10.31, 10.97]
Ahmadi 2020(2)	92.32	13.3	29	93.2	14.55	9	0.3%	-0.88 [-11.55, 9.79]
Ahmadi 2020(3)	94.58	13.9	29	93.2	14.55	9	0.3%	1.38 [-9.39, 12.15]
Bezerra 2023	68.7	7.35	20	67.02	4.14	21	1.2%	1.68 [-2.00, 5.36]
Boff 2020	100.51	11.17	34	101.96	15.38	31	0.7%	-1.45 [-8.04, 5.14]
Chuensiri 2018(1)	84.2	8.95	11	84.7	8.95	5	0.4%	-0.50 [-9.96, 8.96]
Chuensiri 2018(2)	88.6	8.91	15	84.7	8.95	6	0.5%	3.90 [-4.56, 12.36]
Duft 2020	89.29	7.46	18	92.99	7.14	19	1.0%	-3.70 [-8.41, 1.01]
Faria 2020(1)	76	4.97	24	76.5	5.08	13	1.3%	-0.50 [-3.90, 2.90]
Faria 2020(2)	75.2	5.08	26	76.5	5.08	13	1.3%	-1.30 [-4.68, 2.08]
Guo 2015	77.77	7.24	23	77.6	6.82	12	0.9%	0.17 [-4.69, 5.03]
Heidarianpour 2023	68.29	12.96	15	69.47	12.18	15	0.4%	-1.18 [-10.18, 7.82]
Jian 2014	76.31	3.48	44	83.87	3.62	44	1.8%	-7.56 [-9.04, -6.08]
Jianjun 2012	92.46	8.36	20	107.64	7.76	15	0.9%	-15.18 [-20.55, -9.81]
Kai 2024	85.1	0.8	20	87	1.8	24	1.9%	-1.90 [-2.70, -1.10]
Ke 2024	84	10.4	10	100.1	8.3	10	0.5%	-16.10 [-24.35, -7.85]
Kelishadi 2008	60.2	5.38	45	61	5.23	47	1.6%	-0.80 [-2.97, 1.37]
Liangmei 2015	93	5.61	12	101.38	5.58	12	1.0%	-8.38 [-12.86, -3.90]
Lingling 2021	89.7	7	20	94.2	7.5	20	1.0%	-4.50 [-9.00, -0.00]
Liqun 2014	66.33	9.21	76	69.27	9.29	97	1.4%	-2.94 [-5.72, -0.16]
Liu 2018	95.5	8.2	30	100.8	11.1	20	0.8%	-5.30 [-10.98, 0.38]
Lofrano-Prado 2022	102.6	12.7	37	105.9	13.5	37	0.8%	-3.30 [-9.27, 2.67]
Luo 2013(1)	82.29	7.7	43	95.37	5.81	34	1.4%	-13.08 [-16.10, -10.06]
Luo 2013(2)	84.34	7.47	52	98.07	8.4	38	1.3%	-13.73 [-17.08, -10.38]
Meng 2022(1)	78.8	6.1	12	84.9	7.2	6	0.6%	-6.10 [-12.82, 0.62]
Meng 2022(2)	78.5	7.5	11	84.9	7.2	7	0.6%	-6.40 [-13.33, 0.53]
Mohammadi 2019	80.1	9.6	10	75	6.3	10	0.6%	5.10 [-2.02, 12.22]
Monteiro 2015(1)	85.52	6.08	18	95.52	11.8	8	0.5%	-10.00 [-18.65, -1.35]
Monteiro 2015(2)	93.52	7.55	14	95.52	11.8	8	0.4%	-2.00 [-11.08, 7.08]
Plavsic 2020	91.5	7.8	22	96.9	11.4	22	0.8%	-5.40 [-11.17, 0.37]
Shu 2023(1)	86	6.9	16	82.9	5.2	8	0.9%	3.10 [-1.84, 8.04]
Shu 2023(2)	80	4.7	15	82.9	5.2	8	1.1%	-2.90 [-7.22, 1.42]
Sun 2011	81.9	8.5	25	82.1	8.9	17	0.9%	-0.20 [-5.59, 5.19]
Tan 2010	79	7.2	30	81.7	7.2	30	1.2%	-2.70 [-6.34, 0.94]
Toulabi 2012	96.23	9.5	76	100.4	6.99	76	1.5%	-4.17 [-6.82, -1.52]
Vasconcellos 2016	90.5	8.9	10	101.9	17	10	0.3%	-11.40 [-23.29, 0.49]
Xianbo 2011	81.9	8.5	25	82.1	8.9	17	0.9%	-0.20 [-5.59, 5.19]
Xinhui 2016	93.01	5.62	14	101.39	5.59	14	1.1%	-8.38 [-12.53, -4.23]
Yanqin 2017	83.32	7.14	35	88.12	6.85	33	1.3%	-4.80 [-8.13, -1.47]
Yu 2020	84	10.1	99	85.1	5.8	72	1.5%	-1.10 [-3.50, 1.30]
Yuanyuan 2019(1)	79.56	3.35	10	81.22	3.21	3	1.1%	-1.66 [-5.84, 2.52]
Yuanyuan 2019(2)	78.92	3.36	10	81.22	3.21	3	1.1%	-2.30 [-6.49, 1.89]
Yuanyuan 2019(3)	73.14	3.24	9	81.22	3.21	4	1.2%	-8.08 [-11.87, -4.29]
Zehsaz 2016	85.3	3.1	16	89.34	2.7	16	1.6%	-4.04 [-6.05, -2.03]
Zehsaz 2017	83.9	4.9	16	85.8	6.1	16	1.2%	-1.90 [-5.73, 1.93]
Zhihui 2017	89.38	12.45	40	101.21	14.43	40	0.8%	-11.83 [-17.74, -5.92]
**Subtotal (95% CI)**			**1214**			**988**	**44.1%**	**-4.05 [-5.30, -2.79]**
Heterogeneity: Tau² = 11.90; Chi² = 223.18, df = 45 (P < 0.00001); I² = 80%								
Test for overall effect: Z = 6.33 (P < 0.00001)								
**2.8.3 High-income countries**								
Barbeau 2007	67.9	11.3	118	69.9	12.5	83	1.3%	-2.00 [-5.37, 1.37]
Benson 2008	75.2	11.9	31	70.1	6.7	36	1.0%	5.10 [0.37, 9.83]
Bharath 2018	83.5	2.24	20	88.2	4.47	20	1.6%	-4.70 [-6.89, -2.51]
Boer 2014(1)	91.5	13.1	17	95.9	8.2	7	0.4%	-4.40 [-13.10, 4.30]
Boer 2014(2)	93.4	9.6	15	95.9	8.2	7	0.5%	-2.50 [-10.28, 5.28]
CROKER 2012	89.58	3.28	33	90.48	3.31	30	1.7%	-0.90 [-2.53, 0.73]
Davis 2020	79.1	9.38	90	78	11.34	85	1.4%	1.10 [-1.99, 4.19]
Dennis 2013(1)	76.9	3.73	34	77.7	3.82	21	1.6%	-0.80 [-2.86, 1.26]
Dennis 2013(2)	74.8	3.9	36	77.7	3.82	21	1.6%	-2.90 [-4.97, -0.83]
Elmahgoub 2009	96.18	17.82	15	96.39	8.62	15	0.4%	-0.21 [-10.23, 9.81]
Espinoza-Silva 2019(1)	61.77	8.12	69	66.93	10.85	30	1.1%	-5.16 [-9.49, -0.83]
Espinoza-Silva 2019(2)	71.5	9.71	141	72.74	9.24	34	1.3%	-1.24 [-4.73, 2.25]
Farpour-Lambert 2019(1)	79.63	12.55	19	80	12	9	0.4%	-0.37 [-10.03, 9.29]
Farpour-Lambert 2019(2)	79.16	10.61	28	80	12	10	0.5%	-0.84 [-9.25, 7.57]
Harder-Lauridsen 2014	75.9	8.93	18	78.7	7.63	17	0.8%	-2.80 [-8.29, 2.69]
Hay 2016(1)	106	8.9	32	109	15	18	0.5%	-3.00 [-10.58, 4.58]
Hay 2016(2)	102	15	38	109	15	18	0.5%	-7.00 [-15.41, 1.41]
Kim 2007	89.6	7.48	14	91.2	4.16	12	1.0%	-1.60 [-6.17, 2.97]
Kim 2011	87.1	6.58	12	84.8	5.06	10	0.9%	2.30 [-2.57, 7.17]
Kim 2020	85.5	2.7	24	87.7	1.8	24	1.8%	-2.20 [-3.50, -0.90]
Kim 2022	84.2	7.5	13	86.5	10.4	13	0.6%	-2.30 [-9.27, 4.67]
Lambrick 2016	70.9	8.7	15	71.6	11.9	14	0.5%	-0.70 [-8.33, 6.93]
Larsen 2016	79.4	8.6	51	82	8.8	43	1.3%	-2.60 [-6.13, 0.93]
Lee 2010(1)	78.29	9.72	20	82.79	10.7	9	0.5%	-4.50 [-12.69, 3.69]
Lee 2010(2)	79.63	7.73	16	82.79	10.7	9	0.5%	-3.16 [-11.11, 4.79]
Lee 2012(1)	103.3	12.28	15	100.5	10.61	5	0.3%	2.80 [-8.39, 13.99]
Lee 2012(2)	97.9	8	16	100.5	10.61	6	0.4%	-2.60 [-11.95, 6.75]
Lee 2013(1)	104.11	10.97	14	110.53	8.76	4	0.3%	-6.42 [-16.75, 3.91]
Lee 2013(2)	113.58	11.26	14	110.53	8.76	4	0.3%	3.05 [-7.37, 13.47]
Maddison 2011	84.4	10.8	115	88	10.7	116	1.4%	-3.60 [-6.37, -0.83]
Migueles 2023	91.85	3.75	47	93.02	3.74	45	1.8%	-1.17 [-2.70, 0.36]
Park 2007	81.9	6.6	19	85	5.6	21	1.2%	-3.10 [-6.91, 0.71]
Pena 2022	107	17.78	79	111	18.49	38	0.6%	-4.00 [-11.07, 3.07]
Salus(1) 2022	96.3	8.98	14	103.9	17.96	14	0.3%	-7.60 [-18.12, 2.92]
Seo 2019	96.32	9.68	26	93.67	13.97	44	0.8%	2.65 [-2.91, 8.21]
Shalitin 2009	78.7	14.1	55	79.3	14.1	55	0.9%	-0.60 [-5.87, 4.67]
Sigal 2014(1)	93.6	11.26	75	94.9	11.33	25	0.9%	-1.30 [-6.42, 3.82]
Sigal 2014(2)	96.7	11.48	78	94.9	11.33	25	0.9%	1.80 [-3.32, 6.92]
Sigal 2014(3)	92.8	11.26	75	94.9	11.33	26	0.9%	-2.10 [-7.15, 2.95]
Son 2017	84.5	3.13	20	86.3	4.47	20	1.5%	-1.80 [-4.19, 0.59]
Staiano 2017	107.8	21.82	20	108.2	22.51	18	0.2%	-0.40 [-14.53, 13.73]
Suarez-Villadat 2020	15.34	9.18	15	21.52	9.85	30	0.8%	-6.18 [-12.01, -0.35]
Suarez-Villadat 2023	73.29	7.93	24	74.77	8.61	25	1.0%	-1.48 [-6.11, 3.15]
Tjonna 2009	104.9	7.6	20	104.2	7.5	22	1.0%	0.70 [-3.87, 5.27]
Wong 2018	83.5	1.94	15	88.2	3.87	15	1.6%	-4.70 [-6.89, -2.51]
**Subtotal (95% CI)**			**1675**			**1153**	**41.0%**	**-1.92 [-2.53, -1.30]**
Heterogeneity: Tau² = 0.52; Chi² = 51.09, df = 44 (P = 0.22); I² = 14%								
Test for overall effect: Z = 6.11 (P < 0.00001)								
**Total (95% CI)**			**3163**			**2279**	**100.0%**	**-2.86 [-3.52, -2.20]**
Heterogeneity: Tau² = 5.91; Chi² = 306.04, df = 109 (P < 0.00001); I² = 64%								
Test for overall effect: Z = 8.45 (P < 0.00001)								
Test for subgroup differences: Chi² = 8.97, df = 2 (P = 0.01), I² = 77.7%								

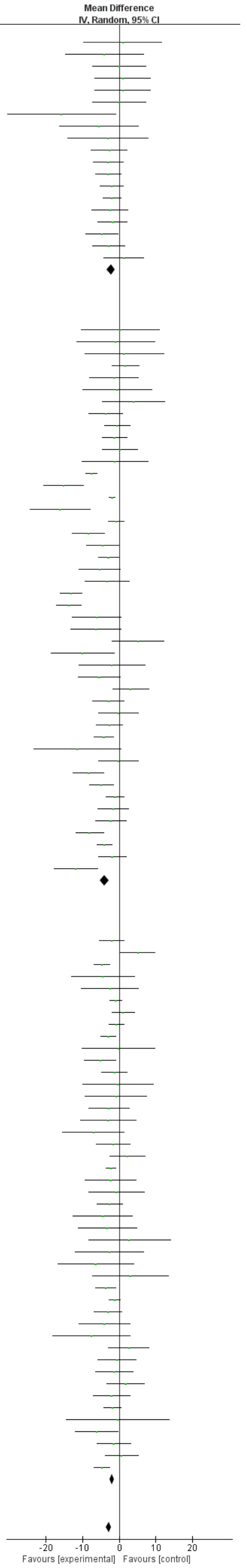

Figure S3.9. Forest plot of subgroup analyses by weight status

Study or Subgroup	Experimental Mean	Experimental SD	Experimental Total	Control Mean	Control SD	Control Total	Weight	Mean Difference IV, Random, 95% CI
**2.9.1 Overweight**								
Barbeau 2007	67.9	11.3	118	69.9	12.5	83	1.9%	-2.00 [-5.37, 1.37]
Bezerra 2023	68.7	7.35	20	67.02	4.14	21	1.8%	1.68 [-2.00, 5.36]
Boer 2014(1)	91.5	13.1	17	95.9	8.2	7	0.7%	-4.40 [-13.10, 4.30]
Boer 2014(2)	93.4	9.6	15	95.9	8.2	7	0.8%	-2.50 [-10.28, 5.28]
Espinoza-Silva 2019(1)	61.77	8.12	69	66.93	10.85	30	1.6%	-5.16 [-9.49, -0.83]
Faria 2020(1)	76	4.97	24	76.5	5.08	13	1.8%	-0.50 [-3.90, 2.90]
Faria 2020(2)	75.2	5.08	26	76.5	5.08	13	1.9%	-1.30 [-4.68, 2.08]
Harder-Lauridsen 2014	75.9	8.93	18	78.7	7.63	17	1.3%	-2.80 [-8.29, 2.69]
Mohammadi 2019	80.1	9.6	10	75	6.3	10	0.9%	5.10 [-2.02, 12.22]
Sun 2011	81.9	8.5	25	82.1	8.9	17	1.3%	-0.20 [-5.59, 5.19]
**Subtotal (95% CI)**			**342**			**218**	**14.0%**	**-1.16 [-2.67, 0.35]**
Heterogeneity: Tau² = 0.62; Chi² = 10.04, df = 9 (P = 0.35); I² = 10%								
Test for overall effect: Z = 1.50 (P = 0.13)								
**2.9.2 Obesity**								
Bharath 2018	83.5	2.24	20	88.2	4.47	20	2.2%	-4.70 [-6.89, -2.51]
Chuensiri 2018(1)	84.2	8.95	11	84.7	8.95	5	0.6%	-0.50 [-9.96, 8.96]
Chuensiri 2018(2)	88.6	8.91	15	84.7	8.95	6	0.8%	3.90 [-4.56, 12.36]
Espinoza-Silva 2019(2)	71.5	9.71	141	72.74	9.24	34	1.8%	-1.24 [-4.73, 2.25]
Farpour-Lambert 2019(1)	79.63	12.55	19	80	12	9	0.6%	-0.37 [-10.03, 9.29]
Farpour-Lambert 2019(2)	79.16	10.61	28	80	12	10	0.8%	-0.84 [-9.25, 7.57]
Jian 2014	76.31	3.48	44	83.87	3.62	44	2.4%	-7.56 [-9.04, -6.08]
Jianjun 2012	92.46	8.36	20	107.64	7.76	15	1.3%	-15.18 [-20.55, -9.81]
Kai 2024	85.1	0.8	20	87	1.8	24	2.5%	-1.90 [-2.70, -1.10]
Ke 2024	84	10.4	10	100.1	8.3	10	0.8%	-16.10 [-24.35, -7.85]
Kelishadi 2008	60.2	5.38	45	61	5.23	47	2.2%	-0.80 [-2.97, 1.37]
Kim 2007	89.6	7.48	14	91.2	4.16	12	1.5%	-1.60 [-6.17, 2.97]
Kim 2011	87.1	6.58	12	84.8	5.06	10	1.4%	2.30 [-2.57, 7.17]
Kim 2020	85.5	2.7	24	87.7	1.8	24	2.4%	-2.20 [-3.50, -0.90]
Lambrick 2016	70.9	8.7	15	71.6	11.9	14	0.9%	-0.70 [-8.33, 6.93]
Lee 2010(1)	78.29	9.72	20	82.79	10.7	9	0.8%	-4.50 [-12.69, 3.69]
Lee 2010(2)	79.63	7.73	16	82.79	10.7	9	0.8%	-3.16 [-11.11, 4.79]
Lee 2012(1)	103.3	12.28	15	100.5	10.61	5	0.5%	2.80 [-8.39, 13.99]
Lee 2012(2)	97.9	8	16	100.5	10.61	6	0.6%	-2.60 [-11.95, 6.75]
Lee 2013(1)	104.11	10.97	14	110.53	8.76	4	0.6%	-6.42 [-16.75, 3.91]
Lee 2013(2)	113.58	11.26	14	110.53	8.76	4	0.6%	3.05 [-7.37, 13.47]
Liangmei 2015	93	5.61	12	101.38	5.58	12	1.5%	-8.38 [-12.86, -3.90]
Liu 2018	95.5	8.2	30	100.8	11.1	20	1.2%	-5.30 [-10.98, 0.38]
Lofrano-Prado 2022	102.6	12.7	37	105.9	13.5	37	1.2%	-3.30 [-9.27, 2.67]
Luo 2013(1)	82.29	7.7	43	95.37	5.81	34	2.0%	-13.08 [-16.10, -10.06]
Luo 2013(2)	84.34	7.47	52	98.07	8.4	38	1.9%	-13.73 [-17.08, -10.38]
Meng 2022(1)	78.8	6.1	12	84.9	7.2	6	1.0%	-6.10 [-12.82, 0.62]
Meng 2022(2)	78.5	7.5	11	84.9	7.2	7	1.0%	-6.40 [-13.33, 0.53]
Monteiro 2015(1)	85.52	6.08	18	95.52	11.8	8	0.7%	-10.00 [-18.65, -1.35]
Monteiro 2015(2)	93.52	7.55	14	95.52	11.8	8	0.7%	-2.00 [-11.08, 7.08]
Park 2007	81.9	6.6	19	85	5.6	21	1.7%	-3.10 [-6.91, 0.71]
Pena 2022	107	17.78	79	111	18.49	38	1.0%	-4.00 [-11.07, 3.07]
Plavsic 2020	91.5	7.8	22	96.9	11.4	22	1.2%	-5.40 [-11.17, 0.37]
Racil(1) 2016(1)	91	6	17	94	4	7	1.6%	-3.00 [-7.11, 1.11]
Racil(1) 2016(2)	91	4	16	94	4	7	1.8%	-3.00 [-6.55, 0.55]
Racil(2) 2016(1)	90	6	23	92	3	9	1.9%	-2.00 [-5.14, 1.14]
Racil(2) 2016(2)	90	4	26	92	3	10	2.1%	-2.00 [-4.41, 0.41]
Racil 2013(1)	90.3	6.7	11	92.8	3.7	6	1.4%	-2.50 [-7.44, 2.44]
Racil 2013(2)	91	4.6	11	92.8	3.7	6	1.7%	-1.80 [-5.82, 2.22]
Racil 2024(1)	90.12	3.9	12	94.83	4.3	5	1.6%	-4.71 [-9.08, -0.34]
Racil 2024(2)	92	4.9	12	94.83	4.3	6	1.5%	-2.83 [-7.25, 1.59]
Regaieg 2013	79.8	6.2	14	78.6	8.5	14	1.3%	1.20 [-4.31, 6.71]
Salus(1) 2022	96.3	8.98	14	103.9	17.96	14	0.5%	-7.60 [-18.12, 2.92]
Seo 2019	96.32	9.68	26	93.67	13.97	44	1.3%	2.65 [-2.91, 8.21]
Shalitin 2009	78.7	14.1	55	79.3	14.1	55	1.3%	-0.60 [-5.87, 4.67]
Shu 2023(1)	86	6.9	16	82.9	5.2	8	1.4%	3.10 [-1.84, 8.04]
Shu 2023(2)	80	4.7	15	82.9	5.2	8	1.6%	-2.90 [-7.22, 1.42]
Son 2017	84.5	3.13	20	86.3	4.47	20	2.2%	-1.80 [-4.19, 0.59]
Suarez-Villadat 2020	15.34	9.18	15	21.52	9.85	30	1.2%	-6.18 [-12.01, -0.35]
Tan 2010	79	7.2	30	81.7	7.2	30	1.8%	-2.70 [-6.34, 0.94]
Toulabi 2012	96.23	9.5	76	100.4	6.99	76	2.1%	-4.17 [-6.82, -1.52]
Vasconcellos 2016	90.5	8.9	10	101.9	17	10	0.4%	-11.40 [-23.29, 0.49]
Wong 2018	83.5	1.94	15	88.2	3.87	15	2.2%	-4.70 [-6.89, -2.51]
Xinhui 2016	93.01	5.62	14	101.39	5.59	14	1.6%	-8.38 [-12.53, -4.23]
Yanqin 2017	83.32	7.14	35	88.12	6.85	33	1.9%	-4.80 [-8.13, -1.47]
Yu 2020	84	10.1	99	85.1	5.8	72	2.1%	-1.10 [-3.50, 1.30]
Yuanyuan 2019(1)	79.56	3.35	10	81.22	3.21	3	1.6%	-1.66 [-5.84, 2.52]
Yuanyuan 2019(2)	78.92	3.36	10	81.22	3.21	3	1.6%	-2.30 [-6.49, 1.89]
Yuanyuan 2019(3)	73.14	3.24	9	81.22	3.21	4	1.7%	-8.08 [-11.87, -4.29]
Zehsaz 2016	85.3	3.1	16	89.34	2.7	16	2.3%	-4.04 [-6.05, -2.03]
Zhihui 2017	89.38	12.45	40	101.21	14.43	40	1.2%	-11.83 [-17.74, -5.92]
**Subtotal (95% CI)**			**1549**			**1137**	**86.0%**	**-3.93 [-4.88, -2.98]**
Heterogeneity: Tau² = 8.01; Chi² = 231.26, df = 60 (P < 0.00001); I² = 74%								
Test for overall effect: Z = 8.09 (P < 0.00001)								
**Total (95% CI)**			**1891**			**1355**	**100.0%**	**-3.54 [-4.41, -2.67]**
Heterogeneity: Tau² = 7.71; Chi² = 250.91, df = 70 (P < 0.00001); I² = 72%								
Test for overall effect: Z = 7.96 (P < 0.00001)								
Test for subgroup differences: Chi² = 9.22, df = 1 (P = 0.002), I² = 89.2%								

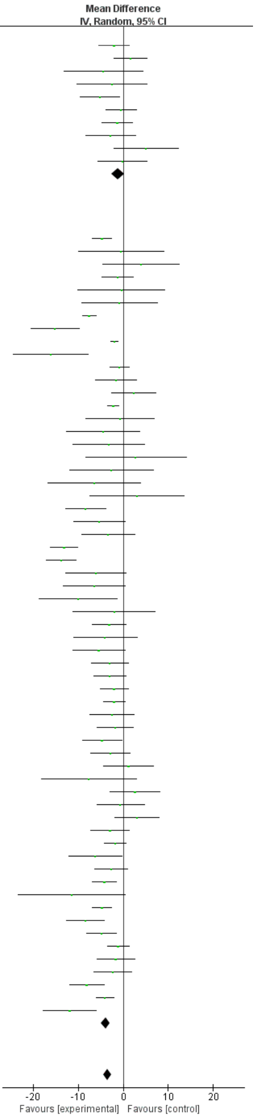

Figure S3.10. Forest plot of subgroup analyses by sex

Study or Subgroup	Experimental Mean	Experimental SD	Experimental Total	Control Mean	Control SD	Control Total	Weight	Mean Difference IV, Random, 95% CI
**2.10.1 Boys**								
Chuensiri 2018(1)	84.2	8.95	11	84.7	8.95	5	1.1%	-0.50 [-9.96, 8.96]
Chuensiri 2018(2)	88.6	8.91	15	84.7	8.95	6	1.2%	3.90 [-4.56, 12.36]
Jianjun 2012	92.46	8.36	20	107.64	7.76	15	2.1%	-15.18 [-20.55, -9.81]
Kim 2007	89.6	7.48	14	91.2	4.16	12	2.5%	-1.60 [-6.17, 2.97]
Kim 2011	87.1	6.58	12	84.8	5.06	10	2.3%	2.30 [-2.57, 7.17]
Lee 2012(1)	103.3	12.28	15	100.5	10.61	5	0.8%	2.80 [-8.39, 13.99]
Lee 2012(2)	97.9	8	16	100.5	10.61	6	1.1%	-2.60 [-11.95, 6.75]
Lingling 2021	89.7	7	20	94.2	7.5	20	2.5%	-4.50 [-9.00, -0.00]
Luo 2013(2)	84.34	7.47	52	98.07	8.4	38	3.0%	-13.73 [-17.08, -10.38]
Meng 2022(1)	78.8	6.1	12	84.9	7.2	6	1.7%	-6.10 [-12.82, 0.62]
Meng 2022(2)	78.5	7.5	11	84.9	7.2	7	1.6%	-6.40 [-13.33, 0.53]
Salus(1) 2022	96.3	8.98	14	103.9	17.96	14	0.9%	-7.60 [-18.12, 2.92]
Zehsaz 2016	85.3	3.1	16	89.34	2.7	16	3.7%	-4.04 [-6.05, -2.03]
Zehsaz 2017	83.9	4.9	16	85.8	6.1	16	2.8%	-1.90 [-5.73, 1.93]
**Subtotal (95% CI)**			**244**			**176**	**27.4%**	**-4.39 [-7.42, -1.35]**
Heterogeneity: Tau² = 23.00; Chi² = 61.74, df = 13 (P < 0.00001); I² = 79%								
Test for overall effect: Z = 2.83 (P = 0.005)								
**2.10.2 Girls**								
Abassi 2020(1)	103	8.84	8	102	8.93	4	0.9%	1.00 [-9.68, 11.68]
Abassi 2020(2)	98	8.59	8	102	8.93	4	0.9%	-4.00 [-14.58, 6.58]
Abassi 2022(1)	101	8.51	13	101	6.99	6	1.5%	0.00 [-7.26, 7.26]
Abassi 2022(2)	102	9.43	13	101	6.99	6	1.4%	1.00 [-6.59, 8.59]
Abassi 2023(1)	102	9.43	13	101	6.99	6	1.4%	1.00 [-6.59, 8.59]
Abassi 2023(2)	101	8.51	13	101	6.99	6	1.5%	0.00 [-7.26, 7.26]
Barbeau 2007	67.9	11.3	118	69.9	12.5	83	3.0%	-2.00 [-5.37, 1.37]
Bharath 2018	83.5	2.24	20	88.2	4.47	20	3.6%	-4.70 [-6.89, -2.51]
Heidarianpour 2023	68.29	12.96	15	69.47	12.18	15	1.1%	-1.18 [-10.18, 7.82]
Jian 2014	76.31	3.48	44	83.87	3.62	44	3.9%	-7.56 [-9.04, -6.08]
Kai 2024	85.1	0.8	20	87	1.8	24	4.1%	-1.90 [-2.70, -1.10]
Kim 2020	85.5	2.7	24	87.7	1.8	24	3.9%	-2.20 [-3.50, -0.90]
Kim 2022	84.2	7.5	13	86.5	10.4	13	1.6%	-2.30 [-9.27, 4.67]
Lee 2013(1)	104.11	10.97	14	110.53	8.76	4	0.9%	-6.42 [-16.75, 3.91]
Lee 2013(2)	113.58	11.26	14	110.53	8.76	4	0.9%	3.05 [-7.37, 13.47]
Liu 2018	95.5	8.2	30	100.8	11.1	20	2.0%	-5.30 [-10.98, 0.38]
Luo 2013(1)	82.29	7.7	43	95.37	5.81	34	3.2%	-13.08 [-16.10, -10.06]
Mohammadi 2019	80.1	9.6	10	75	6.3	10	1.6%	5.10 [-2.02, 12.22]
Park 2007	81.9	6.6	19	85	5.6	21	2.8%	-3.10 [-6.91, 0.71]
Plavsic 2020	91.5	7.8	22	96.9	11.4	22	2.0%	-5.40 [-11.17, 0.37]
Racil(1) 2016(1)	91	6	17	94	4	7	2.7%	-3.00 [-7.11, 1.11]
Racil(1) 2016(2)	91	4	16	94	4	7	2.9%	-3.00 [-6.55, 0.55]
Racil(2) 2016(1)	90	6	23	92	3	9	3.1%	-2.00 [-5.14, 1.14]
Racil(2) 2016(2)	90	4	26	92	3	10	3.5%	-2.00 [-4.41, 0.41]
Racil 2013(1)	90.3	6.7	11	92.8	3.7	6	2.3%	-2.50 [-7.44, 2.44]
Racil 2013(2)	91	4.6	11	92.8	3.7	6	2.7%	-1.80 [-5.82, 2.22]
Racil 2024(1)	90.12	3.9	12	94.83	4.3	5	2.6%	-4.71 [-9.08, -0.34]
Racil 2024(2)	92	4.9	12	94.83	4.3	6	2.5%	-2.83 [-7.25, 1.59]
Son 2017	84.5	3.13	20	86.3	4.47	20	3.5%	-1.80 [-4.19, 0.59]
Staiano 2017	107.8	21.82	20	108.2	22.51	18	0.6%	-0.40 [-14.53, 13.73]
Wong 2018	83.5	1.94	15	88.2	3.87	15	3.6%	-4.70 [-6.89, -2.51]
**Subtotal (95% CI)**			**657**			**479**	**72.6%**	**-3.18 [-4.37, -2.00]**
Heterogeneity: Tau² = 5.66; Chi² = 107.62, df = 30 (P < 0.00001); I² = 72%								
Test for overall effect: Z = 5.26 (P < 0.00001)								
**Total (95% CI)**			**901**			**655**	**100.0%**	**-3.53 [-4.66, -2.40]**
Heterogeneity: Tau² = 7.99; Chi² = 176.81, df = 44 (P < 0.00001); I² = 75%								
Test for overall effect: Z = 6.12 (P < 0.00001)								
Test for subgroup differences: Chi² = 0.53, df = 1 (P = 0.47), I² = 0%								

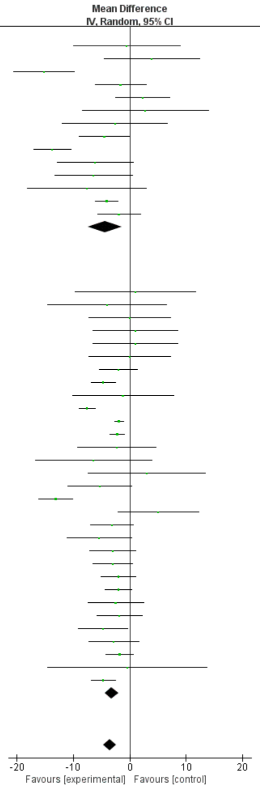

Figure S3.11. Forest plot of subgroup analyses by metabolic status

Study or Subgroup	Experimental Mean	Experimental SD	Experimental Total	Control Mean	Control SD	Control Total	Weight	Mean Difference IV, Random, 95% CI
**2.11.1 Metabolically healthy obesity**								
Boer 2014(1)	91.5	13.1	17	95.9	8.2	7	3.7%	-4.40 [-13.10, 4.30]
Boer 2014(2)	93.4	9.6	15	95.9	8.2	7	4.1%	-2.50 [-10.28, 5.28]
Davis 2020	79.1	9.38	90	78	11.34	85	6.3%	1.10 [-1.99, 4.19]
Faria 2020(1)	76	4.97	24	76.5	5.08	13	6.2%	-0.50 [-3.90, 2.90]
Faria 2020(2)	75.2	5.08	26	76.5	5.08	13	6.2%	-1.30 [-4.68, 2.08]
Harder-Lauridsen 2014	75.9	8.93	18	78.7	7.63	17	5.2%	-2.80 [-8.29, 2.69]
Kim 2007	89.6	7.48	14	91.2	4.16	12	5.7%	-1.60 [-6.17, 2.97]
Shalitin 2009	78.7	14.1	55	79.3	14.1	55	5.3%	-0.60 [-5.87, 4.67]
Yu 2020	84	10.1	99	85.1	5.8	72	6.6%	-1.10 [-3.50, 1.30]
**Subtotal (95% CI)**			**358**			**281**	**49.3%**	**-0.87 [-2.17, 0.42]**
Heterogeneity: Tau² = 0.00; Chi² = 3.08, df = 8 (P = 0.93); I² = 0%								
Test for overall effect: Z = 1.33 (P = 0.18)								
**2.11.2 Metabolically unhealthy obesity**								
Bezerra 2023	68.7	7.35	20	67.02	4.14	21	6.1%	1.68 [-2.00, 5.36]
Kelishadi 2008	60.2	5.38	45	61	5.23	47	6.7%	-0.80 [-2.97, 1.37]
Lee 2010(1)	78.29	9.72	20	82.79	10.7	9	3.9%	-4.50 [-12.69, 3.69]
Lee 2010(2)	79.63	7.73	16	82.79	10.7	9	4.0%	-3.16 [-11.11, 4.79]
Luo 2013(1)	82.29	7.7	43	95.37	5.81	34	6.4%	-13.08 [-16.10, -10.06]
Luo 2013(2)	84.34	7.47	52	98.07	8.4	38	6.2%	-13.73 [-17.08, -10.38]
Meng 2022(1)	78.8	6.1	12	84.9	7.2	6	4.6%	-6.10 [-12.82, 0.62]
Meng 2022(2)	78.5	7.5	11	84.9	7.2	7	4.5%	-6.40 [-13.33, 0.53]
Tjonna 2009	104.9	7.6	20	104.2	7.5	22	5.6%	0.70 [-3.87, 5.27]
Vasconcellos 2016	90.5	8.9	10	101.9	17	10	2.6%	-11.40 [-23.29, 0.49]
**Subtotal (95% CI)**			**249**			**203**	**50.7%**	**-5.51 [-9.95, -1.08]**
Heterogeneity: Tau² = 41.83; Chi² = 88.16, df = 9 (P < 0.00001); I² = 90%								
Test for overall effect: Z = 2.44 (P = 0.01)								
**Total (95% CI)**			**607**			**484**	**100.0%**	**-3.42 [-5.87, -0.97]**
Heterogeneity: Tau² = 22.25; Chi² = 112.10, df = 18 (P < 0.00001); I² = 84%								
Test for overall effect: Z = 2.73 (P = 0.006)								
Test for subgroup differences: Chi² = 3.87, df = 1 (P = 0.05), I² = 74.2%								

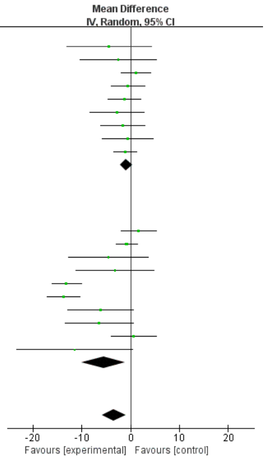

Figure S4. Forest plots of subgroup analyses for BF% in children and adolescents with overweight or obesity

Figure S4.1. Forest plot of subgroup analyses by type of exercise

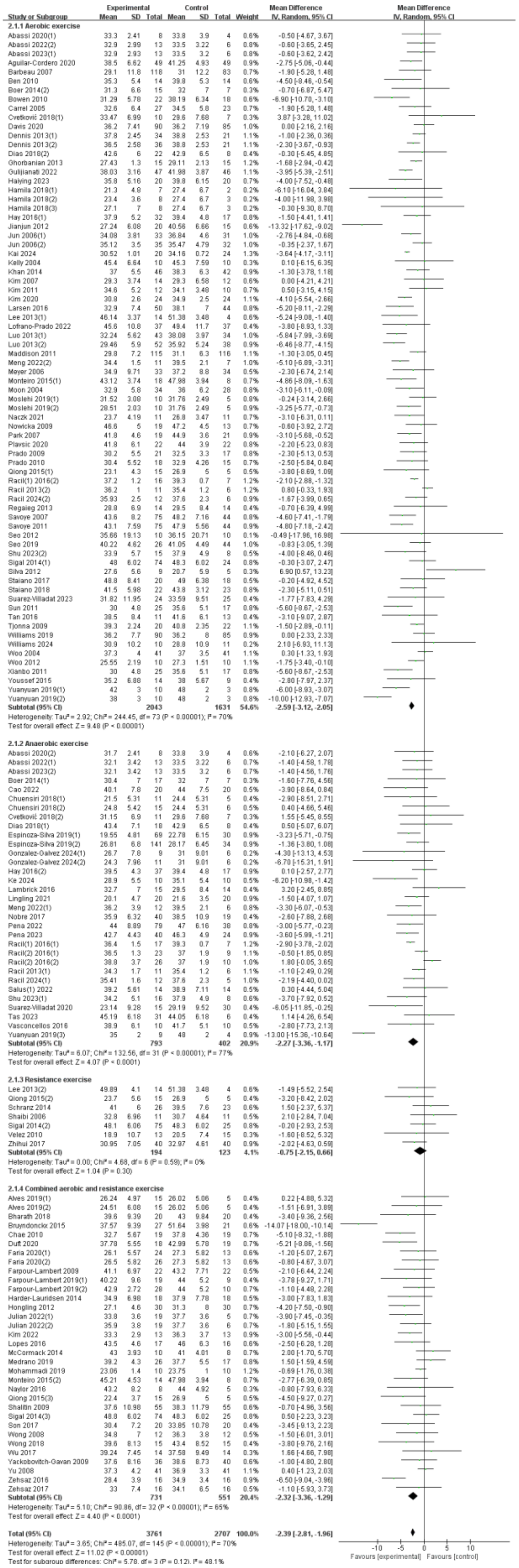

Figure S4.2. Forest plot of subgroup analyses by exercise intensity

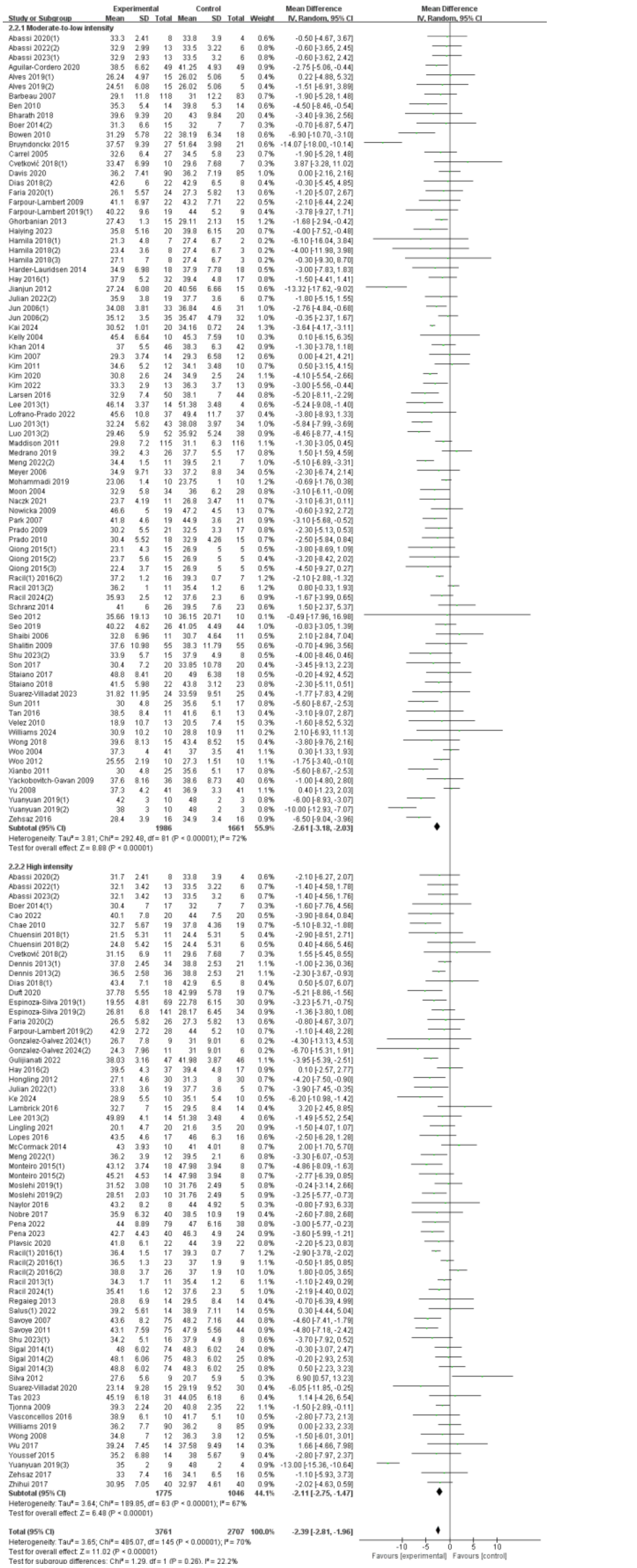

Figure S4.3. Forest plot of subgroup analyses by duration per session

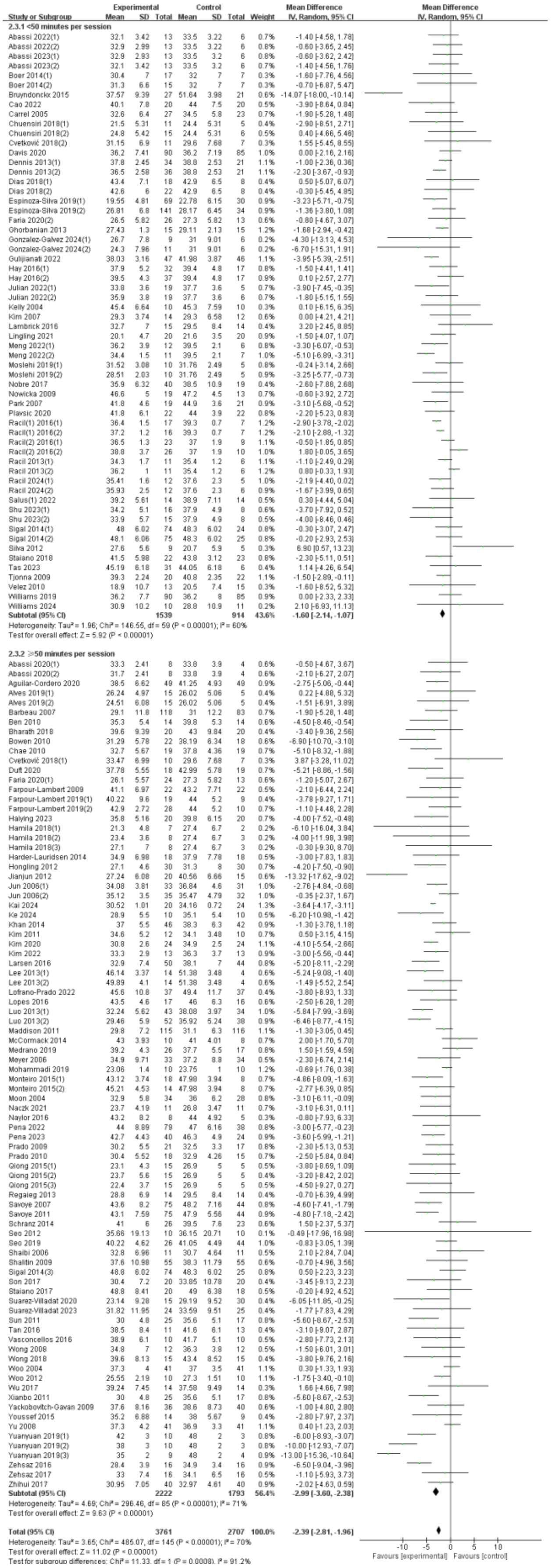

Figure S4.4. Forest plot of subgroup analyses by exercise frequency

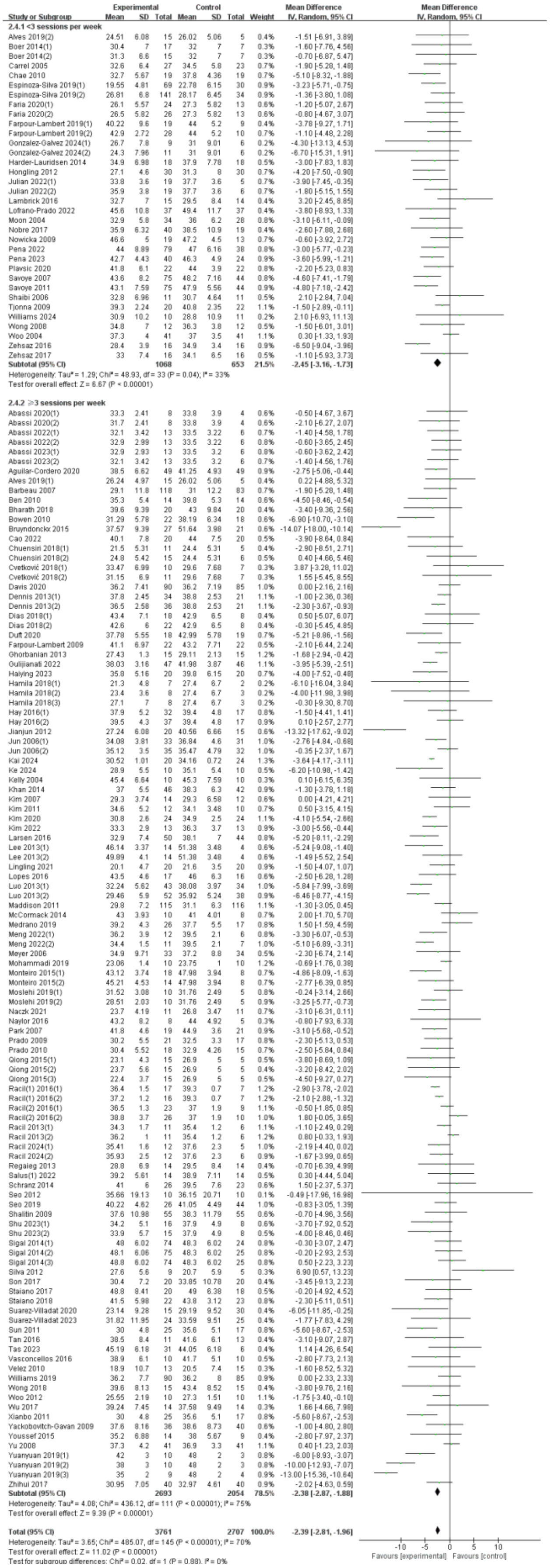

Figure S4.5. Forest plot of subgroup analyses by weekly exercise duration

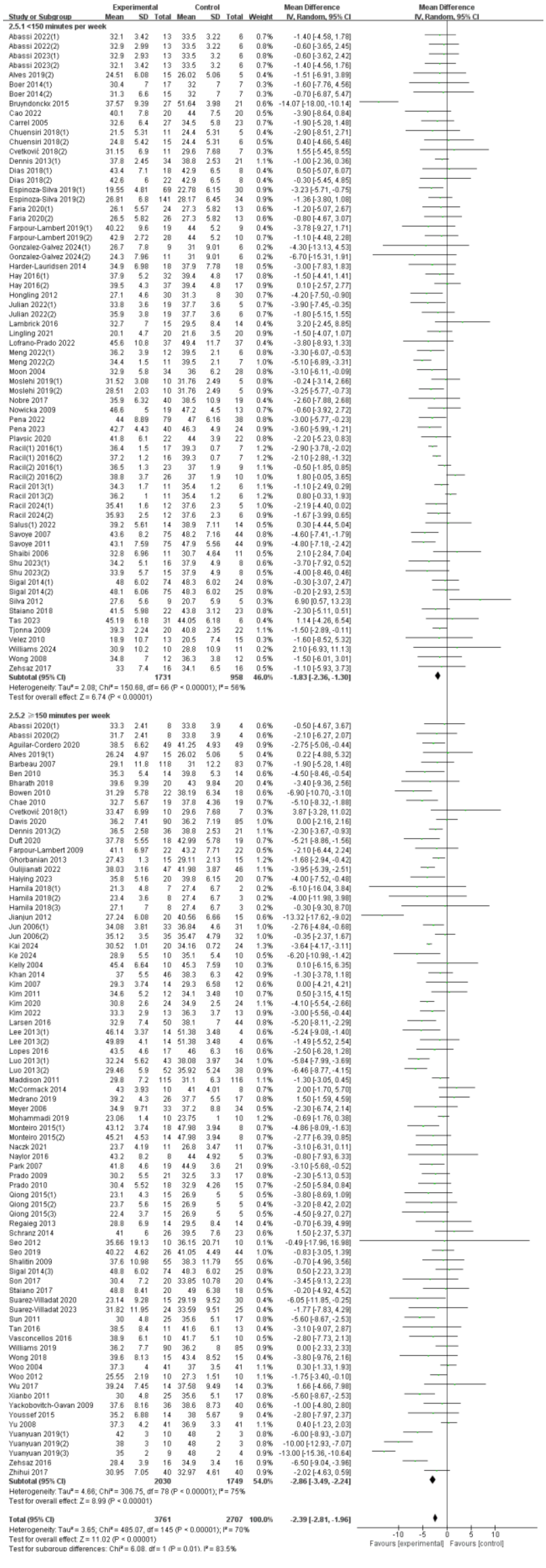

Figure S4.6. Forest plot of subgroup analyses by intervention duration

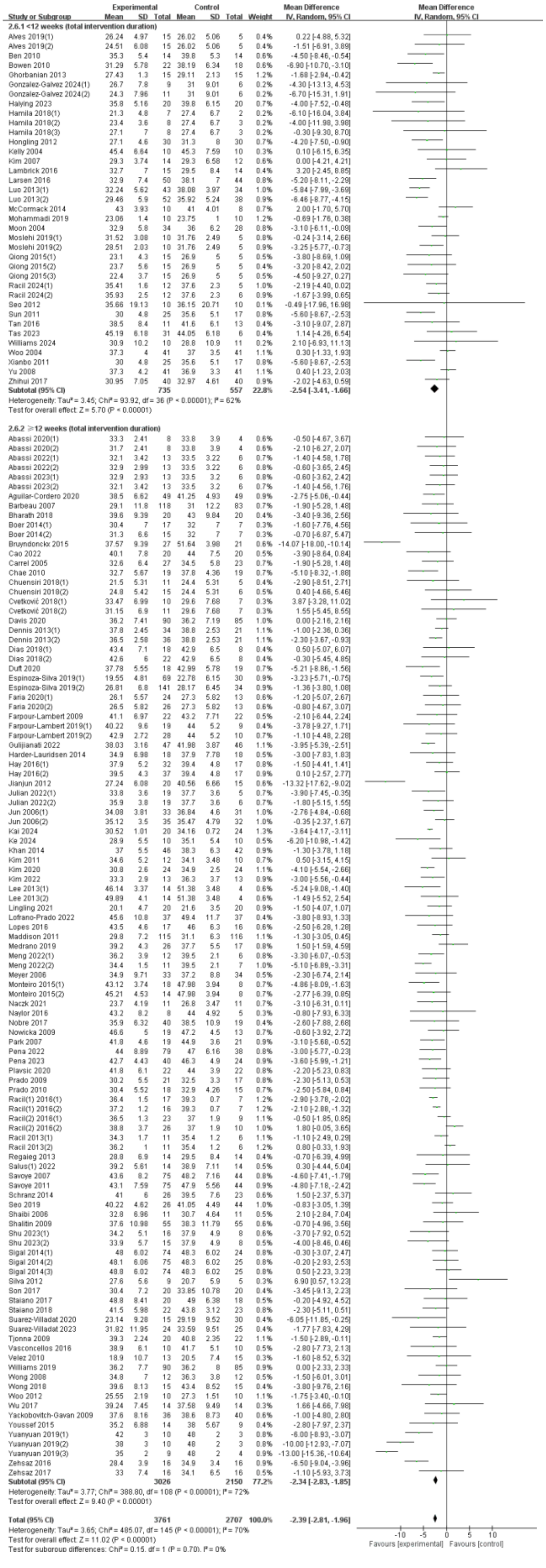

Figure S4.7. Forest plot of subgroup analyses by region

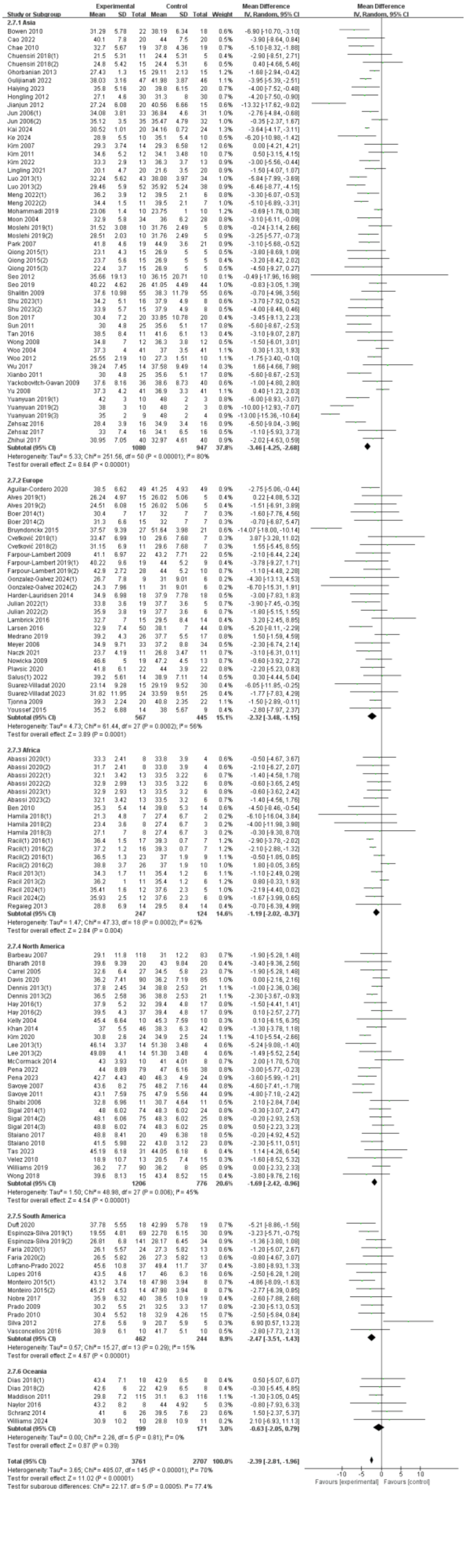

Figure S4.8. Forest plot of subgroup analyses by economic status

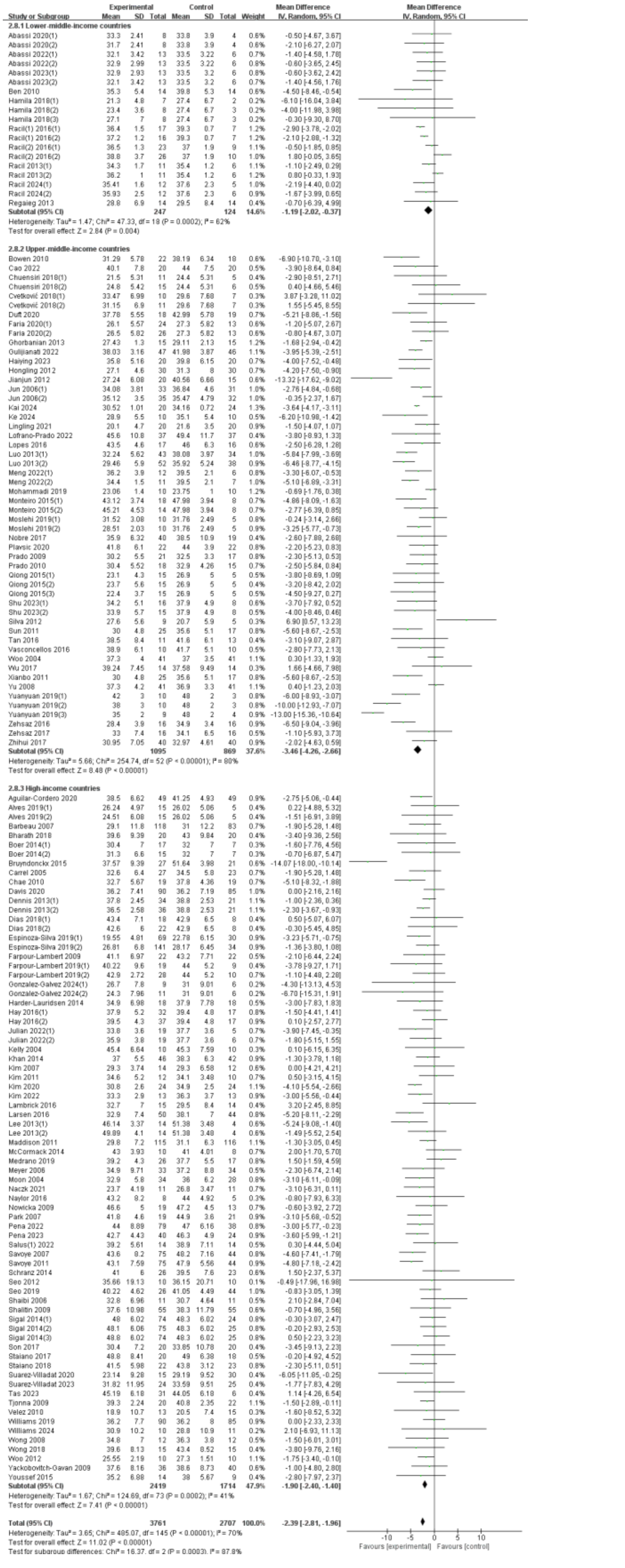

Figure S4.9. Forest plot of subgroup analyses by weight status

Study or Subgroup	Experimental Mean	Experimental SD	Experimental Total	Control Mean	Control SD	Control Total	Weight	Mean Difference IV, Random, 95% CI
**2.9.1 Overweight**								
Barbeau 2007	29.1	11.8	118	31	12.2	83	1.1%	-1.90 [-5.28, 1.48]
Boer 2014(1)	30.4	7	17	32	7	7	0.5%	-1.60 [-7.76, 4.56]
Boer 2014(2)	31.3	6.6	15	32	7	7	0.5%	-0.70 [-6.87, 5.47]
Espinoza-Silva 2019(1)	19.55	4.81	69	22.78	6.15	30	1.3%	-3.23 [-5.71, -0.75]
Faria 2020(1)	26.1	5.57	24	27.3	5.82	13	0.9%	-1.20 [-5.07, 2.67]
Faria 2020(2)	26.5	5.82	26	27.3	5.82	13	0.9%	-0.80 [-4.67, 3.07]
Gulijianati 2022	38.03	3.16	47	41.98	3.87	46	1.6%	-3.95 [-5.39, -2.51]
Harder-Lauridsen 2014	34.9	6.98	18	37.9	7.78	18	0.7%	-3.00 [-7.83, 1.83]
Kelly 2004	45.4	6.64	10	45.3	7.59	10	0.5%	0.10 [-6.15, 6.35]
Lopes 2016	43.5	4.6	17	46	6.3	16	1.0%	-2.50 [-6.28, 1.28]
Mohammadi 2019	23.06	1.4	10	23.75	1	10	1.7%	-0.69 [-1.76, 0.38]
Naczk 2021	23.7	4.19	11	26.8	3.47	11	1.1%	-3.10 [-6.31, 0.11]
Shaibi 2006	32.8	6.96	11	30.7	4.64	11	0.7%	2.10 [-2.84, 7.04]
Silva 2012	27.6	5.6	9	20.7	5.9	5	0.5%	6.90 [0.57, 13.23]
Sun 2011	30	4.8	25	35.6	5.1	17	1.1%	-5.60 [-8.67, -2.53]
Woo 2012	25.55	2.19	10	27.3	1.51	10	1.5%	-1.75 [-3.40, -0.10]
**Subtotal (95% CI)**			**437**			**307**	**15.7%**	**-1.94 [-3.04, -0.85]**
Heterogeneity: Tau² = 2.01; Chi² = 31.21, df = 15 (P = 0.008); I² = 52%								
Test for overall effect: Z = 3.48 (P = 0.0005)								
**2.9.2 Obesity**								
Ben 2010	35.3	5.4	14	39.8	5.3	14	0.9%	-4.50 [-8.46, -0.54]
Bharath 2018	39.6	9.39	20	43	9.84	20	0.6%	-3.40 [-9.36, 2.56]
Bruyndonckx 2015	37.57	9.39	27	51.64	3.98	21	0.9%	-14.07 [-18.00, -10.14]
Cao 2022	40.1	7.8	20	44	7.5	20	0.8%	-3.90 [-8.64, 0.84]
Carrel 2005	32.6	6.4	27	34.5	5.8	23	1.0%	-1.90 [-5.28, 1.48]
Chae 2010	32.7	5.67	19	37.8	4.36	19	1.1%	-5.10 [-8.32, -1.88]
Chuensiri 2018(1)	21.5	5.31	11	24.4	5.31	5	0.6%	-2.90 [-8.51, 2.71]
Chuensiri 2018(2)	24.8	5.42	15	24.4	5.31	6	0.7%	0.40 [-4.66, 5.46]
Dias 2018(1)	43.4	7.1	18	42.9	6.5	8	0.6%	0.50 [-5.07, 6.07]
Dias 2018(2)	42.6	6	22	42.9	6.5	8	0.7%	-0.30 [-5.45, 4.85]
Espinoza-Silva 2019(2)	26.81	6.8	141	28.17	6.45	34	1.3%	-1.36 [-3.80, 1.08]
Farpour-Lambert 2009	41.1	6.97	22	43.2	7.71	22	0.8%	-2.10 [-6.44, 2.24]
Farpour-Lambert 2019(1)	40.22	9.6	19	44	5.2	9	0.6%	-3.78 [-9.27, 1.71]
Farpour-Lambert 2019(2)	42.9	2.72	28	44	5.2	10	1.1%	-1.10 [-4.48, 2.28]
Haiying 2023	35.8	5.16	20	39.8	6.15	20	1.0%	-4.00 [-7.52, -0.48]
Hongling 2012	27.1	4.6	30	31.3	8	30	1.1%	-4.20 [-7.50, -0.90]
Jianjun 2012	27.24	6.08	20	40.56	6.66	15	0.8%	-13.32 [-17.62, -9.02]
Julian 2022(1)	33.8	3.6	19	37.7	3.6	5	1.0%	-3.90 [-7.45, -0.35]
Julian 2022(2)	35.9	3.8	19	37.7	3.6	6	1.1%	-1.80 [-5.15, 1.55]
Jun 2006(1)	34.08	3.81	33	36.84	4.6	31	1.4%	-2.76 [-4.84, -0.68]
Jun 2006(2)	35.12	3.5	35	35.47	4.79	32	1.4%	-0.35 [-2.37, 1.67]
Kai 2024	30.52	1.01	20	34.16	0.72	24	1.8%	-3.64 [-4.17, -3.11]
Ke 2024	28.9	5.5	10	35.1	5.4	10	0.7%	-6.20 [-10.98, -1.42]
Kim 2007	29.3	3.74	14	29.3	6.58	12	0.9%	0.00 [-4.21, 4.21]
Kim 2011	34.6	5.2	12	34.1	3.48	10	1.0%	0.50 [-3.15, 4.15]
Kim 2020	30.8	2.6	24	34.9	2.5	24	1.6%	-4.10 [-5.54, -2.66]
Lambrick 2016	32.7	7	15	29.5	8.4	14	0.6%	3.20 [-2.45, 8.85]
Lee 2013(1)	46.14	3.37	14	51.38	3.48	4	0.9%	-5.24 [-9.08, -1.40]
Lee 2013(2)	49.89	4.1	14	51.38	3.48	4	0.9%	-1.49 [-5.52, 2.54]
Lofrano-Prado 2022	45.6	10.8	37	49.4	11.7	37	0.7%	-3.80 [-8.93, 1.33]
Luo 2013(1)	32.24	5.62	43	38.08	3.97	34	1.4%	-5.84 [-7.99, -3.69]
Luo 2013(2)	29.46	5.9	52	35.92	5.24	38	1.3%	-6.46 [-8.77, -4.15]
McCormack 2014	43	3.93	10	41	4.01	8	1.0%	2.00 [-1.70, 5.70]
Meng 2022(1)	36.2	3.9	12	39.5	2.1	6	1.2%	-3.30 [-6.07, -0.53]
Meng 2022(2)	34.4	1.5	11	39.5	2.1	7	1.5%	-5.10 [-6.89, -3.31]
Meyer 2006	34.9	9.71	33	37.2	8.8	34	0.8%	-2.30 [-6.74, 2.14]
Monteiro 2015(1)	43.12	3.74	18	47.98	3.94	8	1.1%	-4.86 [-8.09, -1.63]
Monteiro 2015(2)	45.21	4.53	14	47.98	3.94	8	1.0%	-2.77 [-6.39, 0.85]
Moon 2004	32.9	5.8	34	36	6.2	28	1.1%	-3.10 [-6.11, -0.09]
Moslehi 2019(1)	31.52	3.08	10	31.76	2.49	5	1.2%	-0.24 [-3.14, 2.66]
Moslehi 2019(2)	28.51	2.03	10	31.76	2.49	5	1.3%	-3.25 [-5.77, -0.73]
Naylor 2016	43.2	8.2	8	44	4.92	5	0.4%	-0.80 [-7.93, 6.33]
Nowicka 2009	46.6	5	19	47.2	4.5	13	1.1%	-0.60 [-3.92, 2.72]
Park 2007	41.8	4.6	19	44.9	3.6	21	1.3%	-3.10 [-5.68, -0.52]
Pena 2022	44	8.89	79	47	6.16	38	1.2%	-3.00 [-5.77, -0.23]
Pena 2023	42.7	4.43	40	46.3	4.9	24	1.3%	-3.60 [-5.99, -1.21]
Plavsic 2020	41.8	6.1	22	44	3.9	22	1.1%	-2.20 [-5.23, 0.83]
Prado 2009	30.2	5.5	21	32.5	3.3	17	1.2%	-2.30 [-5.13, 0.53]
Prado 2010	30.4	5.52	18	32.9	4.26	15	1.1%	-2.50 [-5.84, 0.84]
Qiong 2015(1)	23.1	4.3	15	26.9	5	5	0.7%	-3.80 [-8.69, 1.09]
Qiong 2015(2)	23.7	5.6	15	26.9	5	5	0.7%	-3.20 [-8.42, 2.02]
Qiong 2015(3)	22.4	3.7	15	26.9	5	5	0.7%	-4.50 [-9.27, 0.27]
Racil(1) 2016(1)	36.4	1.5	17	39.3	0.7	7	1.7%	-2.90 [-3.78, -2.02]
Racil(1) 2016(2)	37.2	1.2	16	39.3	0.7	7	1.7%	-2.10 [-2.88, -1.32]
Racil(2) 2016(1)	36.5	1.3	23	37	1.9	9	1.6%	-0.50 [-1.85, 0.85]
Racil(2) 2016(2)	38.8	3.7	26	37	1.9	10	1.5%	1.80 [-0.05, 3.65]
Racil 2013(1)	34.3	1.7	11	35.4	1.2	6	1.6%	-1.10 [-2.49, 0.29]
Racil 2013(2)	36.2	1	11	35.4	1.2	6	1.7%	0.80 [-0.33, 1.93]
Racil 2024(1)	35.41	1.6	12	37.6	2.3	5	1.4%	-2.19 [-4.40, 0.02]
Racil 2024(2)	35.93	2.5	12	37.6	2.3	6	1.3%	-1.67 [-3.99, 0.65]
Regaieg 2013	28.8	6.9	14	29.5	8.4	14	0.6%	-0.70 [-6.39, 4.99]
Salus(1) 2022	39.2	5.61	14	38.9	7.11	14	0.8%	0.30 [-4.44, 5.04]
Savoye 2007	43.6	8.2	75	48.2	7.16	44	1.2%	-4.60 [-7.41, -1.79]
Savoye 2011	43.1	7.59	75	47.9	5.56	44	1.3%	-4.80 [-7.18, -2.42]
Seo 2012	35.66	19.13	10	36.15	20.71	10	0.1%	-0.49 [-17.96, 16.98]
Seo 2019	40.22	4.62	26	41.05	4.49	44	1.4%	-0.83 [-3.05, 1.39]
Shalitin 2009	37.6	10.98	55	38.3	11.79	55	0.8%	-0.70 [-4.96, 3.56]
Shu 2023(1)	34.2	5.1	16	37.9	4.9	8	0.9%	-3.70 [-7.92, 0.52]
Shu 2023(2)	33.9	5.7	15	37.9	4.9	8	0.8%	-4.00 [-8.46, 0.46]
Son 2017	30.4	7.2	20	33.85	10.78	20	0.6%	-3.45 [-9.13, 2.23]
Suarez-Villadat 2020	23.14	9.28	15	29.19	9.52	30	0.6%	-6.05 [-11.85, -0.25]
Tan 2016	38.5	8.4	11	41.6	6.1	13	0.6%	-3.10 [-9.07, 2.87]
Tas 2023	45.19	6.18	31	44.05	6.18	6	0.6%	1.14 [-4.26, 6.54]
Vasconcellos 2016	38.9	6.1	10	41.7	5.1	10	0.7%	-2.80 [-7.73, 2.13]
Wong 2008	34.8	7	12	36.3	3.8	12	0.8%	-1.50 [-6.01, 3.01]
Wong 2018	39.6	8.13	15	43.4	8.52	15	0.6%	-3.80 [-9.76, 2.16]
Yackobovitch-Gavan 2009	37.6	8.16	36	38.6	8.73	40	0.9%	-1.00 [-4.80, 2.80]
Yuanyuan 2019(1)	42	3	10	48	2	3	1.2%	-6.00 [-8.93, -3.07]
Yuanyuan 2019(2)	38	3	10	48	2	3	1.2%	-10.00 [-12.93, -7.07]
Yuanyuan 2019(3)	35	2	9	48	2	4	1.3%	-13.00 [-15.36, -10.64]
Zehsaz 2016	28.4	3.9	16	34.9	3.4	16	1.3%	-6.50 [-9.04, -3.96]
Zhihui 2017	30.95	7.05	40	32.97	4.61	40	1.3%	-2.02 [-4.63, 0.59]
**Subtotal (95% CI)**			**1920**			**1357**	**84.3%**	**-3.06 [-3.67, -2.46]**
Heterogeneity: Tau² = 4.65; Chi² = 343.97, df = 81 (P < 0.00001); I² = 76%								
Test for overall effect: Z = 9.91 (P < 0.00001)								
**Total (95% CI)**			**2357**			**1664**	**100.0%**	**-2.87 [-3.41, -2.32]**
Heterogeneity: Tau² = 4.27; Chi² = 382.65, df = 97 (P < 0.00001); I² = 75%								
Test for overall effect: Z = 10.38 (P < 0.00001)								
Test for subgroup differences: Chi² = 3.06, df = 1 (P = 0.08), I² = 67.4%								

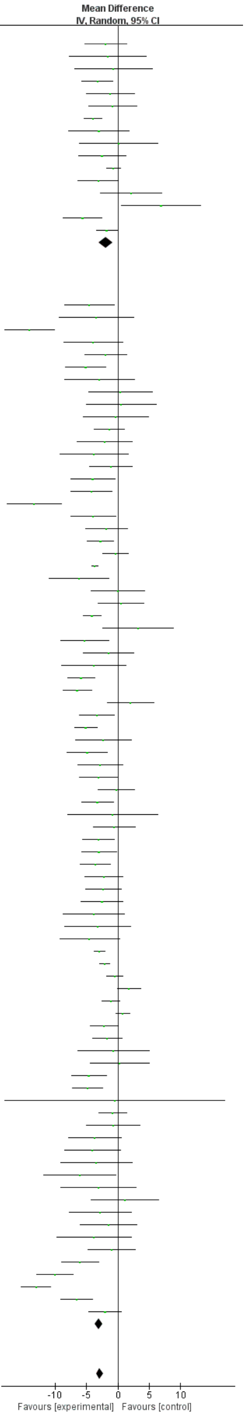

Figure S4.10. Forest plot of subgroup analyses by sex

Study or Subgroup	Experimental Mean	Experimental SD	Experimental Total	Control Mean	Control SD	Control Total	Weight	Mean Difference IV, Random, 95% CI
**2.10.1 Boys**								
Chuensiri 2018(1)	21.5	5.31	11	24.4	5.31	5	0.9%	-2.90 [-8.51, 2.71]
Chuensiri 2018(2)	24.8	5.42	15	24.4	5.31	6	1.0%	0.40 [-4.66, 5.46]
Cvetković 2018(1)	33.47	6.99	10	29.6	7.68	7	0.6%	3.87 [-3.28, 11.02]
Cvetković 2018(2)	31.15	6.9	11	29.6	7.68	7	0.6%	1.55 [-5.45, 8.55]
Ghorbanian 2013	27.43	1.3	15	29.11	2.13	15	3.0%	-1.68 [-2.94, -0.42]
Hongling 2012	27.1	4.6	30	31.3	8	30	1.7%	-4.20 [-7.50, -0.90]
Jianjun 2012	27.24	6.08	20	40.56	6.66	15	1.3%	-13.32 [-17.62, -9.02]
Kim 2007	29.3	3.74	14	29.3	6.58	12	1.3%	0.00 [-4.21, 4.21]
Kim 2011	34.6	5.2	12	34.1	3.48	10	1.6%	0.50 [-3.15, 4.15]
Lingling 2021	20.1	4.7	20	21.6	3.5	20	2.2%	-1.50 [-4.07, 1.07]
Luo 2013(2)	29.46	5.9	52	35.92	5.24	38	2.3%	-6.46 [-8.77, -4.15]
Meng 2022(1)	36.2	3.9	12	39.5	2.1	6	2.0%	-3.30 [-6.07, -0.53]
Meng 2022(2)	34.4	1.5	11	39.5	2.1	7	2.7%	-5.10 [-6.89, -3.31]
Moslehi 2019(1)	31.52	3.08	10	31.76	2.49	5	1.9%	-0.24 [-3.14, 2.66]
Moslehi 2019(2)	28.51	2.03	10	31.76	2.49	5	2.2%	-3.25 [-5.77, -0.73]
Nobre 2017	35.9	6.32	40	38.5	10.9	19	1.0%	-2.60 [-7.88, 2.68]
Qiong 2015(1)	23.1	4.3	15	26.9	5	5	1.1%	-3.80 [-8.69, 1.09]
Qiong 2015(2)	23.7	5.6	15	26.9	5	5	1.0%	-3.20 [-8.42, 2.02]
Qiong 2015(3)	22.4	3.7	15	26.9	5	5	1.1%	-4.50 [-9.27, 0.27]
Salus(1) 2022	39.2	5.61	14	38.9	7.11	14	1.1%	0.30 [-4.44, 5.04]
Schranz 2014	41	6	26	39.5	7.6	23	1.5%	1.50 [-2.37, 5.37]
Seo 2012	35.66	19.13	10	36.15	20.71	10	0.1%	-0.49 [-17.96, 16.98]
Shaibi 2006	32.8	6.96	11	30.7	4.64	11	1.1%	2.10 [-2.84, 7.04]
Tan 2016	38.5	8.4	11	41.6	6.1	13	0.8%	-3.10 [-9.07, 2.87]
Wong 2008	34.8	7	12	36.3	3.8	12	1.2%	-1.50 [-6.01, 3.01]
Zehsaz 2016	28.4	3.9	16	34.9	3.4	16	2.2%	-6.50 [-9.04, -3.96]
Zehsaz 2017	33	7.4	16	34.1	6.5	16	1.1%	-1.10 [-5.93, 3.73]
**Subtotal (95% CI)**			**454**			**337**	**38.5%**	**-2.56 [-3.76, -1.36]**
Heterogeneity: Tau² = 5.61; Chi² = 77.96, df = 26 (P < 0.00001); I² = 67%								
Test for overall effect: Z = 4.19 (P < 0.0001)								
**2.10.2 Girls**								
Abassi 2020(1)	33.3	2.41	8	33.8	3.9	4	1.3%	-0.50 [-4.67, 3.67]
Abassi 2020(2)	31.7	2.41	8	33.8	3.9	4	1.3%	-2.10 [-6.27, 2.07]
Abassi 2022(1)	32.1	3.42	13	33.5	3.22	6	1.8%	-1.40 [-4.58, 1.78]
Abassi 2022(2)	32.9	2.99	13	33.5	3.22	6	1.9%	-0.60 [-3.65, 2.45]
Abassi 2023(1)	32.9	2.93	13	33.5	3.2	6	1.9%	-0.60 [-3.62, 2.42]
Abassi 2023(2)	32.1	3.42	13	33.5	3.2	6	1.8%	-1.40 [-4.56, 1.76]
Barbeau 2007	29.1	11.8	118	31	12.2	83	1.7%	-1.90 [-5.28, 1.48]
Bharath 2018	39.6	9.39	20	43	9.84	20	0.8%	-3.40 [-9.36, 2.56]
Kai 2024	30.52	1.01	20	34.16	0.72	24	3.4%	-3.64 [-4.17, -3.11]
Kim 2020	30.8	2.6	24	34.9	2.5	24	2.9%	-4.10 [-5.54, -2.66]
Kim 2022	33.3	2.9	13	36.3	3.7	13	2.2%	-3.00 [-5.56, -0.44]
Lee 2013(1)	46.14	3.37	14	51.38	3.48	4	1.5%	-5.24 [-9.08, -1.40]
Lee 2013(2)	49.89	4.1	14	51.38	3.48	4	1.4%	-1.49 [-5.52, 2.54]
Lopes 2016	43.5	4.6	17	46	6.3	16	1.5%	-2.50 [-6.28, 1.28]
Luo 2013(1)	32.24	5.62	43	38.08	3.97	34	2.4%	-5.84 [-7.99, -3.69]
Mohammadi 2019	23.06	1.4	10	23.75	1	10	3.1%	-0.69 [-1.76, 0.38]
Park 2007	41.8	4.6	19	44.9	3.6	21	2.1%	-3.10 [-5.68, -0.52]
Plavsic 2020	41.8	6.1	22	44	3.9	22	1.9%	-2.20 [-5.23, 0.83]
Racil(1) 2016(1)	36.4	1.5	17	39.3	0.7	7	3.2%	-2.90 [-3.78, -2.02]
Racil(1) 2016(2)	37.2	1.2	16	39.3	0.7	7	3.3%	-2.10 [-2.88, -1.32]
Racil(2) 2016(1)	36.5	1.3	23	37	1.9	9	3.0%	-0.50 [-1.85, 0.85]
Racil(2) 2016(2)	38.8	3.7	26	37	1.9	10	2.6%	1.80 [-0.05, 3.65]
Racil 2013(1)	34.3	1.7	11	35.4	1.2	6	2.9%	-1.10 [-2.49, 0.29]
Racil 2013(2)	36.2	1	11	35.4	1.2	6	3.1%	0.80 [-0.33, 1.93]
Racil 2024(1)	35.41	1.6	12	37.6	2.3	5	2.4%	-2.19 [-4.40, 0.02]
Racil 2024(2)	35.93	2.5	12	37.6	2.3	6	2.3%	-1.67 [-3.99, 0.65]
Son 2017	30.4	7.2	20	33.85	10.78	20	0.9%	-3.45 [-9.13, 2.23]
Staiano 2017	48.8	8.41	20	49	6.38	18	1.1%	-0.20 [-4.92, 4.52]
Wong 2018	39.6	8.13	15	43.4	8.52	15	0.8%	-3.80 [-9.76, 2.16]
Youssef 2015	35.2	6.88	14	38	5.67	9	1.0%	-2.80 [-7.97, 2.37]
**Subtotal (95% CI)**			**599**			**425**	**61.5%**	**-1.94 [-2.66, -1.22]**
Heterogeneity: Tau² = 2.24; Chi² = 118.13, df = 29 (P < 0.00001); I² = 75%								
Test for overall effect: Z = 5.26 (P < 0.00001)								
**Total (95% CI)**			**1053**			**762**	**100.0%**	**-2.23 [-2.84, -1.62]**
Heterogeneity: Tau² = 2.80; Chi² = 199.11, df = 56 (P < 0.00001); I² = 72%								
Test for overall effect: Z = 7.15 (P < 0.00001)								
Test for subgroup differences: Chi² = 0.76, df = 1 (P = 0.38), I² = 0%								

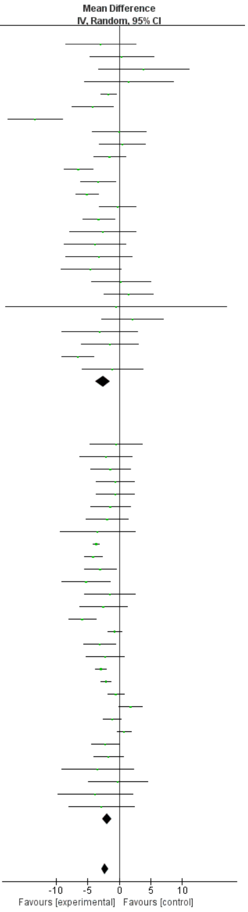

Figure S4.11. Forest plot of subgroup analyses by metabolic status

Study or Subgroup	Experimental Mean	Experimental SD	Experimental Total	Control Mean	Control SD	Control Total	Weight	Mean Difference IV, Random, 95% CI
**2.11.1 Metabolically healthy obesity**								
Boer 2014(1)	30.4	7	17	32	7	7	2.3%	-1.60 [-7.76, 4.56]
Boer 2014(2)	31.3	6.6	15	32	7	7	2.3%	-0.70 [-6.87, 5.47]
Davis 2020	36.2	7.41	90	36.2	7.19	85	7.3%	0.00 [-2.16, 2.16]
Faria 2020(1)	26.1	5.57	24	27.3	5.82	13	4.4%	-1.20 [-5.07, 2.67]
Faria 2020(2)	26.5	5.82	26	27.3	5.82	13	4.4%	-0.80 [-4.67, 3.07]
Harder-Lauridsen 2014	34.9	6.98	18	37.9	7.78	18	3.3%	-3.00 [-7.83, 1.83]
Hongling 2012	27.1	4.6	30	31.3	8	30	5.2%	-4.20 [-7.50, -0.90]
Kim 2007	29.3	3.74	14	29.3	6.58	12	4.0%	0.00 [-4.21, 4.21]
Shalitin 2009	37.6	10.98	55	38.3	11.79	55	3.9%	-0.70 [-4.96, 3.56]
Wong 2008	34.8	7	12	36.3	3.8	12	3.6%	-1.50 [-6.01, 3.01]
**Subtotal (95% CI)**			**301**			**252**	**40.7%**	**-1.18 [-2.38, 0.02]**
Heterogeneity: Tau² = 0.00; Chi² = 5.35, df = 9 (P = 0.80); I² = 0%								
Test for overall effect: Z = 1.93 (P = 0.05)								
**2.11.2 Metabolically unhealthy obesity**								
Farpour-Lambert 2009	41.1	6.97	22	43.2	7.71	22	3.8%	-2.10 [-6.44, 2.24]
Kelly 2004	45.4	6.64	10	45.3	7.59	10	2.3%	0.10 [-6.15, 6.35]
Luo 2013(1)	32.24	5.62	43	38.08	3.97	34	7.3%	-5.84 [-7.99, -3.69]
Luo 2013(2)	29.46	5.9	52	35.92	5.24	38	7.0%	-6.46 [-8.77, -4.15]
Meng 2022(1)	36.2	3.9	12	39.5	2.1	6	6.1%	-3.30 [-6.07, -0.53]
Meng 2022(2)	34.4	1.5	11	39.5	2.1	7	8.0%	-5.10 [-6.89, -3.31]
Savoye 2007	43.6	8.2	75	48.2	7.16	44	6.0%	-4.60 [-7.41, -1.79]
Savoye 2011	43.1	7.59	75	47.9	5.56	44	6.8%	-4.80 [-7.18, -2.42]
Tjonna 2009	39.3	2.24	20	40.8	2.35	22	8.8%	-1.50 [-2.89, -0.11]
Vasconcellos 2016	38.9	6.1	10	41.7	5.1	10	3.2%	-2.80 [-7.73, 2.13]
**Subtotal (95% CI)**			**330**			**237**	**59.3%**	**-4.03 [-5.39, -2.68]**
Heterogeneity: Tau² = 2.68; Chi² = 24.44, df = 9 (P = 0.004); I² = 63%								
Test for overall effect: Z = 5.83 (P < 0.00001)								
**Total (95% CI)**			**631**			**489**	**100.0%**	**-2.93 [-4.00, -1.86]**
Heterogeneity: Tau² = 2.87; Chi² = 43.76, df = 19 (P = 0.001); I² = 57%								
Test for overall effect: Z = 5.38 (P < 0.00001)								
Test for subgroup differences: Chi² = 9.55, df = 1 (P = 0.002), I² = 89.5%								

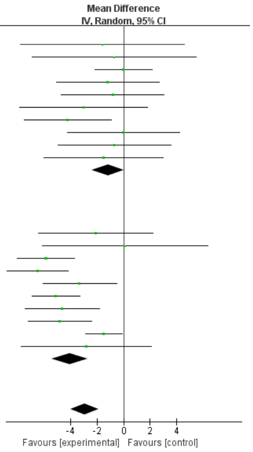

Figure S5. Forest plots of subgroup analyses for VO_2max in children and adolescents with overweight or obesity

Figure S5.1. Forest plot of subgroup analyses by type of exercise

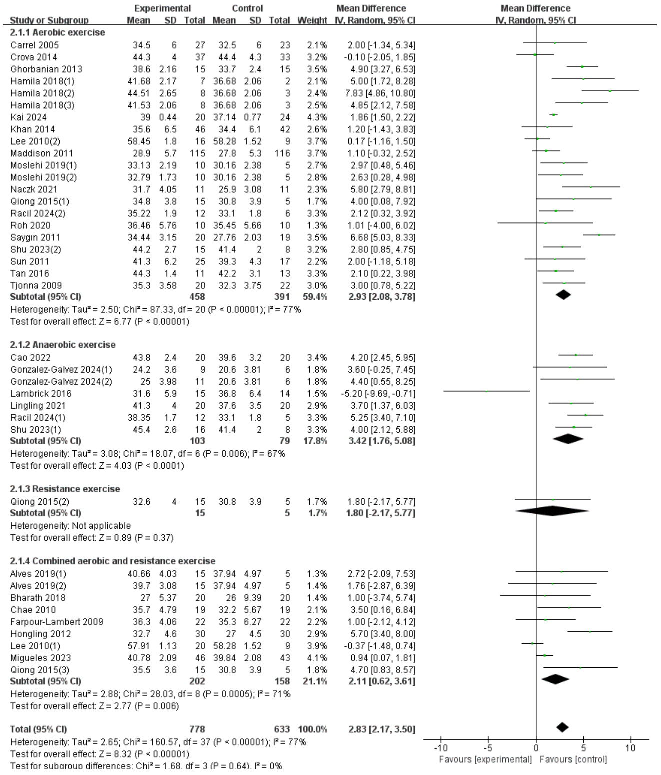

Figure S5.2. Forest plot of subgroup analyses by exercise intensity

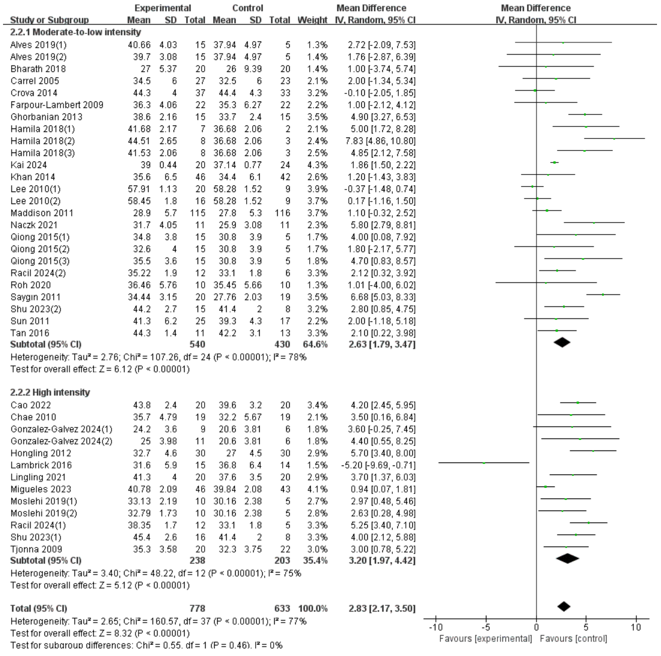

Figure S5.3. Forest plot of subgroup analyses by duration per session

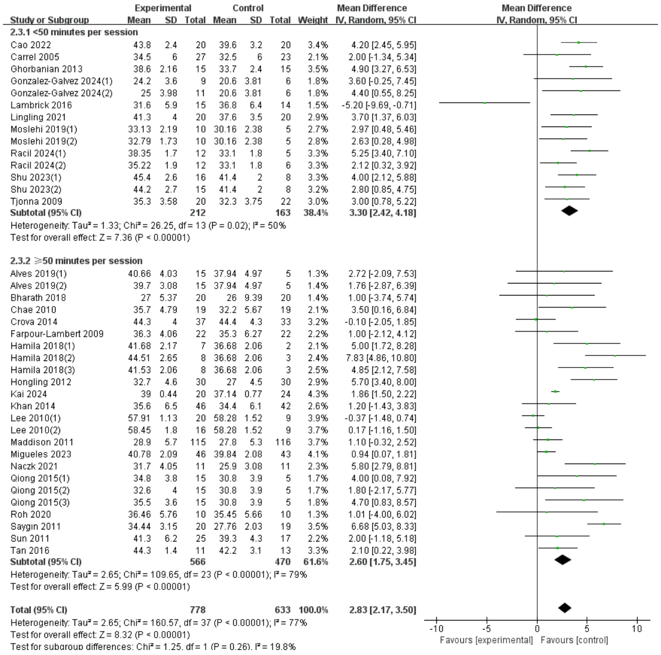

Figure S5.4. Forest plot of subgroup analyses by exercise frequency

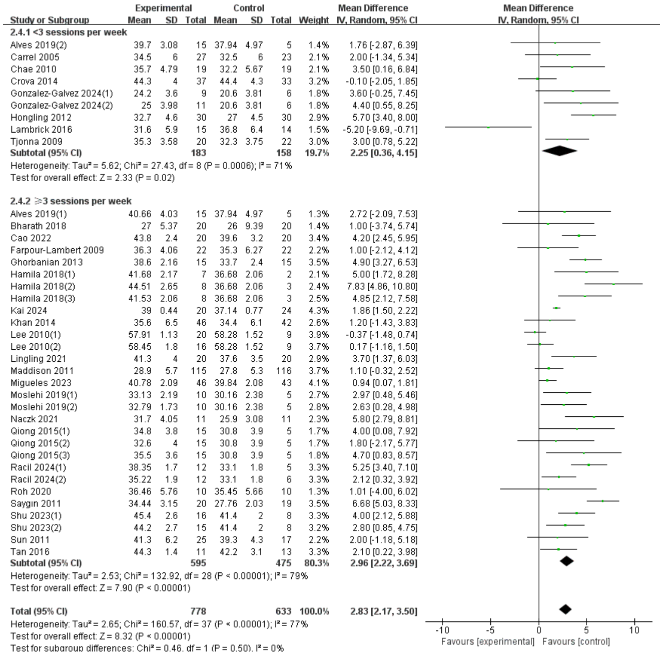

Figure S5.5. Forest plot of subgroup analyses by weekly exercise duration

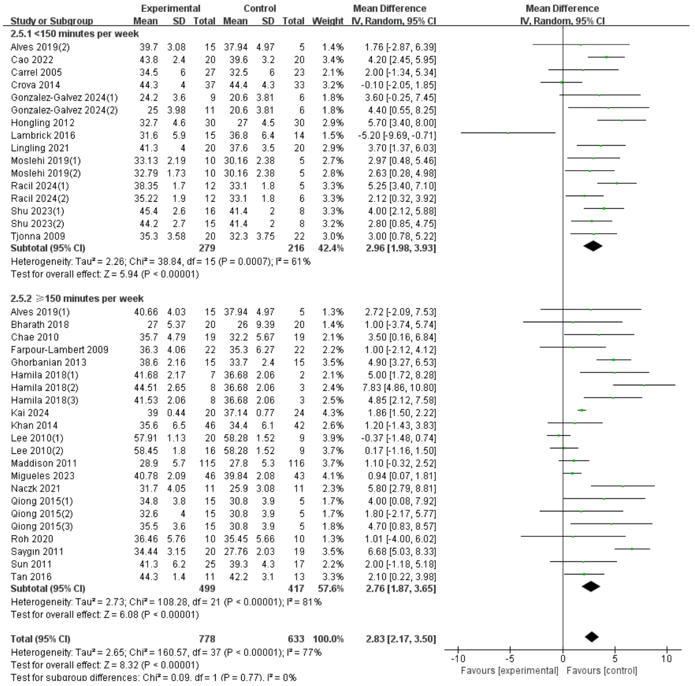

Figure S5.6. Forest plot of subgroup analyses by intervention duration

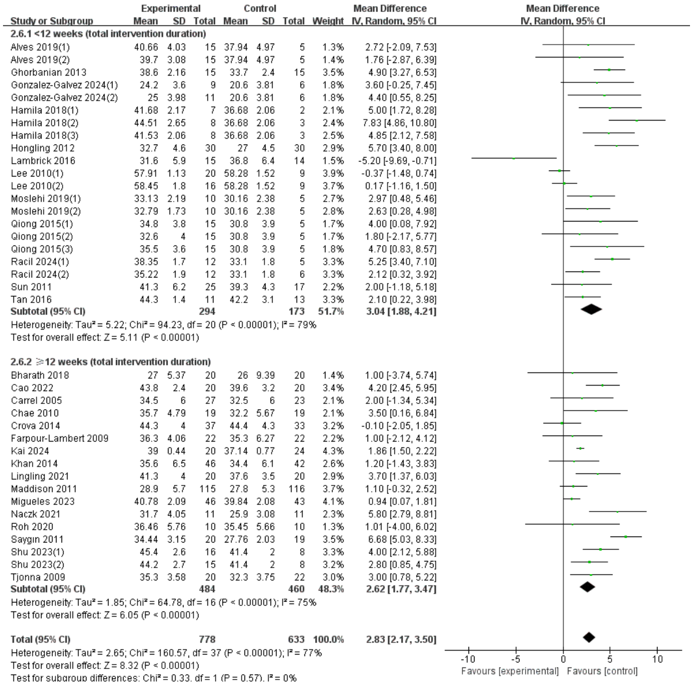

Figure S5.7. Forest plot of subgroup analyses by region

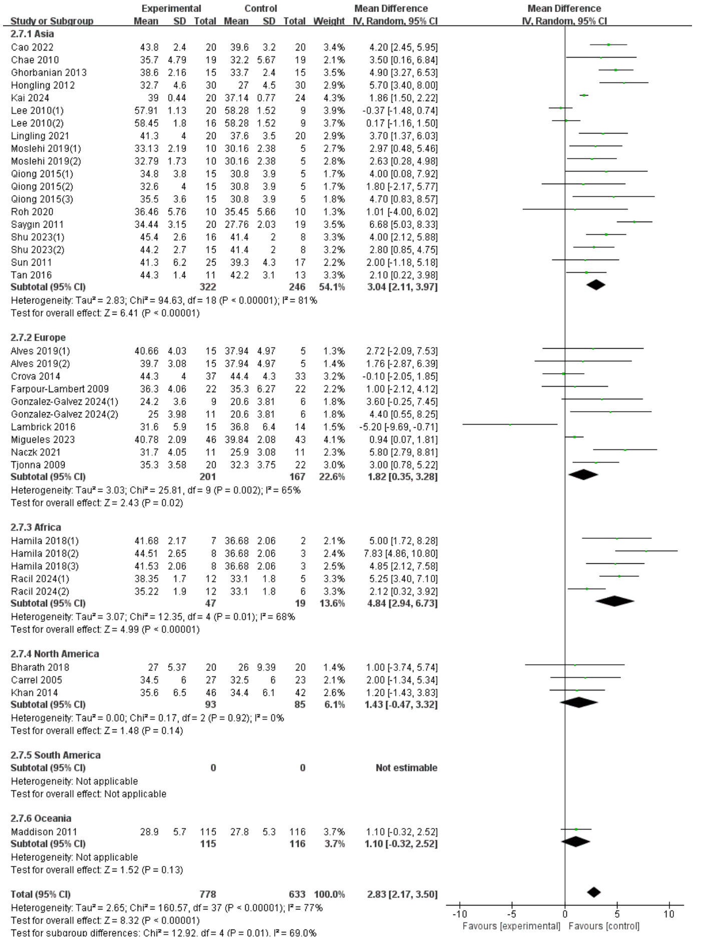

Figure S5.8. Forest plot of subgroup analyses by economic status

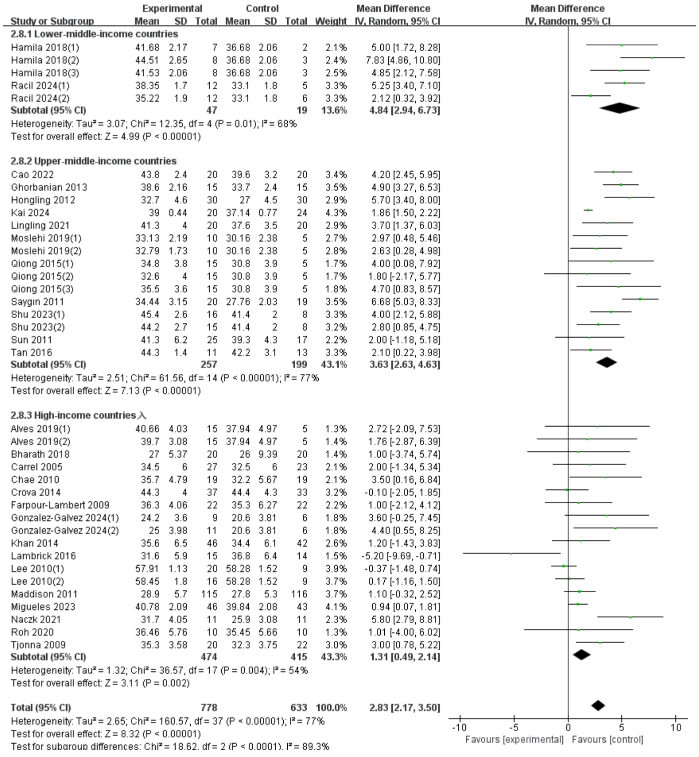

Figure S5.9. Forest plot of subgroup analyses by weight status

Study or Subgroup	Experimental Mean	Experimental SD	Experimental Total	Control Mean	Control SD	Control Total	Weight	Mean Difference IV, Random, 95% CI
**2.9.1 Overweight**								
Crova 2014	44.3	4	37	44.4	4.3	33	4.8%	-0.10 [-2.05, 1.85]
Naczk 2021	31.7	4.05	11	25.9	3.08	11	3.6%	5.80 [2.79, 8.81]
Sun 2011	41.3	6.2	25	39.3	4.3	17	3.4%	2.00 [-1.18, 5.18]
**Subtotal (95% CI)**			**73**			**61**	**11.8%**	**2.44 [-1.10, 5.98]**
Heterogeneity: Tau² = 7.86; Chi² = 10.44, df = 2 (P = 0.005); I² = 81%								
Test for overall effect: Z = 1.35 (P = 0.18)								
**2.9.2 Obesity**								
Bharath 2018	27	5.37	20	26	9.39	20	2.2%	1.00 [-3.74, 5.74]
Cao 2022	43.8	2.4	20	39.6	3.2	20	5.1%	4.20 [2.45, 5.95]
Carrel 2005	34.5	6	27	32.5	6	23	3.3%	2.00 [-1.34, 5.34]
Chae 2010	35.7	4.79	19	32.2	5.67	19	3.3%	3.50 [0.16, 6.84]
Farpour-Lambert 2009	36.3	4.06	22	35.3	6.27	22	3.5%	1.00 [-2.12, 4.12]
Hongling 2012	32.7	4.6	30	27	4.5	30	4.4%	5.70 [3.40, 8.00]
Kai 2024	39	0.44	20	37.14	0.77	24	6.4%	1.86 [1.50, 2.22]
Lambrick 2016	31.6	5.9	15	36.8	6.4	14	2.3%	-5.20 [-9.69, -0.71]
Lee 2010(1)	57.91	1.13	20	58.28	1.52	9	5.8%	-0.37 [-1.48, 0.74]
Lee 2010(2)	58.45	1.8	16	58.28	1.52	9	5.6%	0.17 [-1.16, 1.50]
Moslehi 2019(1)	33.13	2.19	10	30.16	2.38	5	4.2%	2.97 [0.48, 5.46]
Moslehi 2019(2)	32.79	1.73	10	30.16	2.38	5	4.3%	2.63 [0.28, 4.98]
Qiong 2015(1)	34.8	3.8	15	30.8	3.9	5	2.7%	4.00 [0.08, 7.92]
Qiong 2015(2)	32.6	4	15	30.8	3.9	5	2.7%	1.80 [-2.17, 5.77]
Qiong 2015(3)	35.5	3.6	15	30.8	3.9	5	2.8%	4.70 [0.83, 8.57]
Racil 2024(1)	38.35	1.7	12	33.1	1.8	5	4.9%	5.25 [3.40, 7.10]
Racil 2024(2)	35.22	1.9	12	33.1	1.8	6	5.0%	2.12 [0.32, 3.92]
Saygın 2011	34.44	3.15	20	27.76	2.03	19	5.2%	6.68 [5.03, 8.33]
Shu 2023(1)	45.4	2.6	16	41.4	2	8	4.9%	4.00 [2.12, 5.88]
Shu 2023(2)	44.2	2.7	15	41.4	2	8	4.8%	2.80 [0.85, 4.75]
Tan 2016	44.3	1.4	11	42.2	3.1	13	4.9%	2.10 [0.22, 3.98]
**Subtotal (95% CI)**			**360**			**274**	**88.2%**	**2.67 [1.76, 3.59]**
Heterogeneity: Tau² = 2.97; Chi² = 103.55, df = 20 (P < 0.00001); I² = 81%								
Test for overall effect: Z = 5.74 (P < 0.00001)								
**Total (95% CI)**			**433**			**335**	**100.0%**	**2.63 [1.77, 3.49]**
Heterogeneity: Tau² = 2.98; Chi² = 114.22, df = 23 (P < 0.00001); I² = 80%								
Test for overall effect: Z = 6.01 (P < 0.00001)								
Test for subgroup differences: Chi² = 0.02, df = 1 (P = 0.90), I² = 0%								

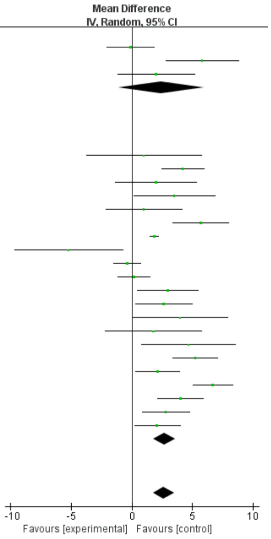

Figure S5.10. Forest plot of subgroup analyses by sex

Study or Subgroup	Experimental Mean	Experimental SD	Experimental Total	Control Mean	Control SD	Control Total	Weight	Mean Difference IV, Random, 95% CI
**2.10.1 Boys**								
Ghorbanian 2013	38.6	2.16	15	33.7	2.4	15	8.8%	4.90 [3.27, 6.53]
Hongling 2012	32.7	4.6	30	27	4.5	30	7.4%	5.70 [3.40, 8.00]
Lingling 2021	41.3	4	20	37.6	3.5	20	7.4%	3.70 [1.37, 6.03]
Moslehi 2019(1)	33.13	2.19	10	30.16	2.38	5	7.0%	2.97 [0.48, 5.46]
Moslehi 2019(2)	32.79	1.73	10	30.16	2.38	5	7.3%	2.63 [0.28, 4.98]
Qiong 2015(1)	34.8	3.8	15	30.8	3.9	5	4.6%	4.00 [0.08, 7.92]
Qiong 2015(2)	32.6	4	15	30.8	3.9	5	4.6%	1.80 [-2.17, 5.77]
Qiong 2015(3)	35.5	3.6	15	30.8	3.9	5	4.7%	4.70 [0.83, 8.57]
Tan 2016	44.3	1.4	11	42.2	3.1	13	8.3%	2.10 [0.22, 3.98]
**Subtotal (95% CI)**			**141**			**103**	**60.1%**	**3.69 [2.76, 4.61]**
Heterogeneity: Tau² = 0.40; Chi² = 10.05, df = 8 (P = 0.26); I² = 20%								
Test for overall effect: Z = 7.83 (P < 0.00001)								
**2.10.2 Girls**								
Bharath 2018	27	5.37	20	26	9.39	20	3.7%	1.00 [-3.74, 5.74]
Kai 2024	39	0.44	20	37.14	0.77	24	10.7%	1.86 [1.50, 2.22]
Racil 2024(1)	38.35	1.7	12	33.1	1.8	5	8.3%	5.25 [3.40, 7.10]
Racil 2024(2)	35.22	1.9	12	33.1	1.8	6	8.5%	2.12 [0.32, 3.92]
Saygın 2011	34.44	3.15	20	27.76	2.03	19	8.7%	6.68 [5.03, 8.33]
**Subtotal (95% CI)**			**84**			**74**	**39.9%**	**3.57 [1.33, 5.81]**
Heterogeneity: Tau² = 5.33; Chi² = 42.22, df = 4 (P < 0.00001); I² = 91%								
Test for overall effect: Z = 3.12 (P = 0.002)								
**Total (95% CI)**			**225**			**177**	**100.0%**	**3.63 [2.51, 4.74]**
Heterogeneity: Tau² = 3.00; Chi² = 64.22, df = 13 (P < 0.00001); I² = 80%								
Test for overall effect: Z = 6.36 (P < 0.00001)								
Test for subgroup differences: Chi² = 0.01, df = 1 (P = 0.93), I² = 0%								

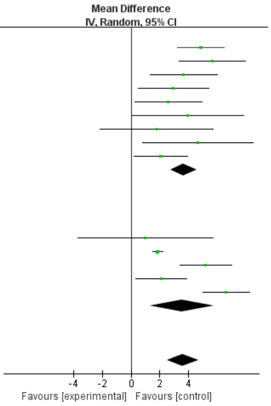

Figure S6. Forest plots of subgroup analyses for VO_2peak in children and adolescents with overweight or obesity

Figure S6.1. Forest plot of subgroup analyses by type of exercise

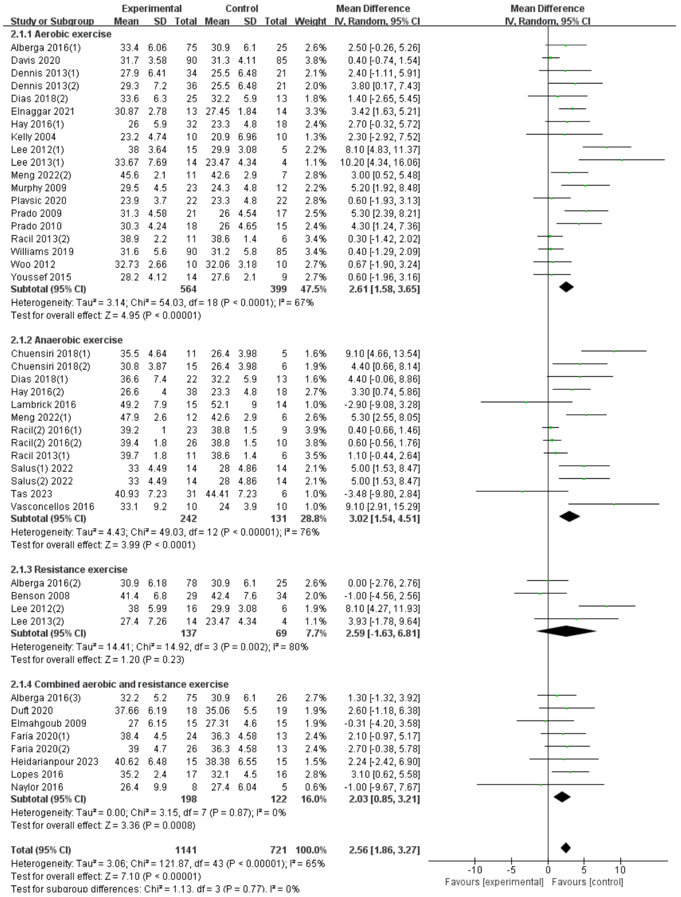

Figure S6.2. Forest plot of subgroup analyses by exercise intensity

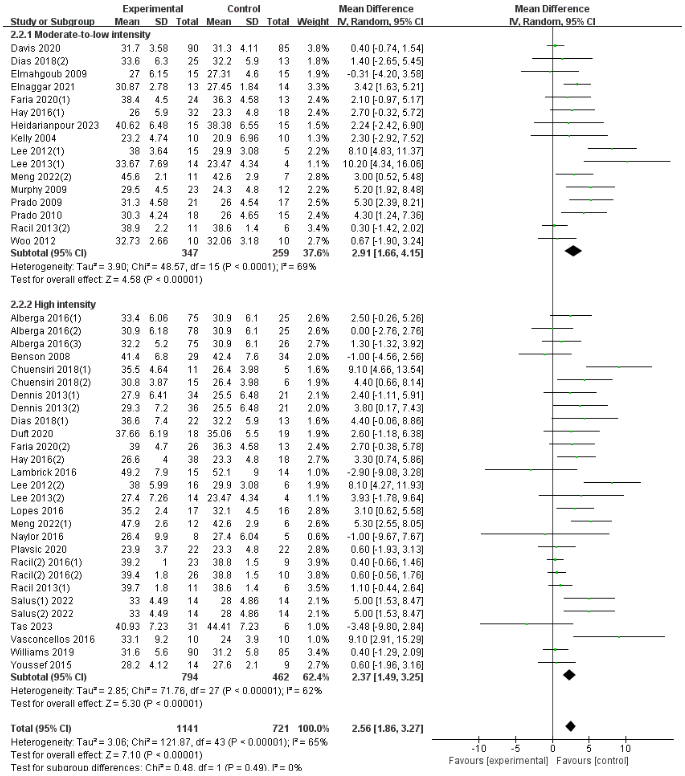

Figure S6.3. Forest plot of subgroup analyses by duration per session

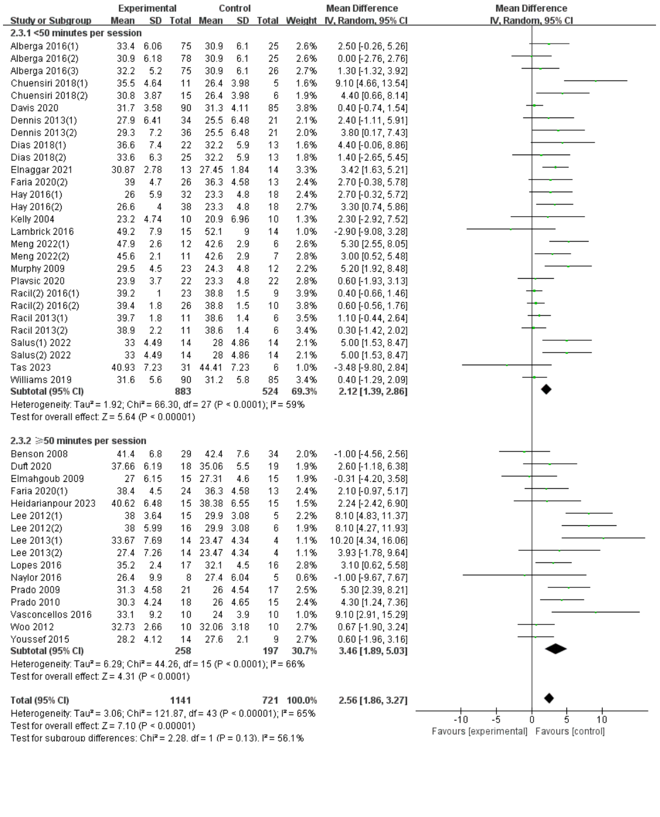

Figure S6.4. Forest plot of subgroup analyses by exercise frequency

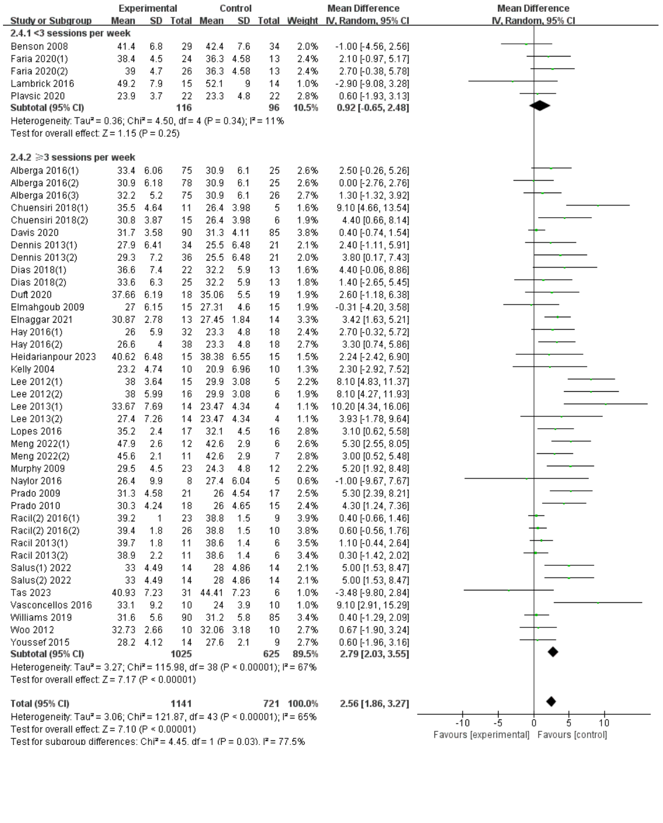

Figure S6.5. Forest plot of subgroup analyses by weekly exercise duration

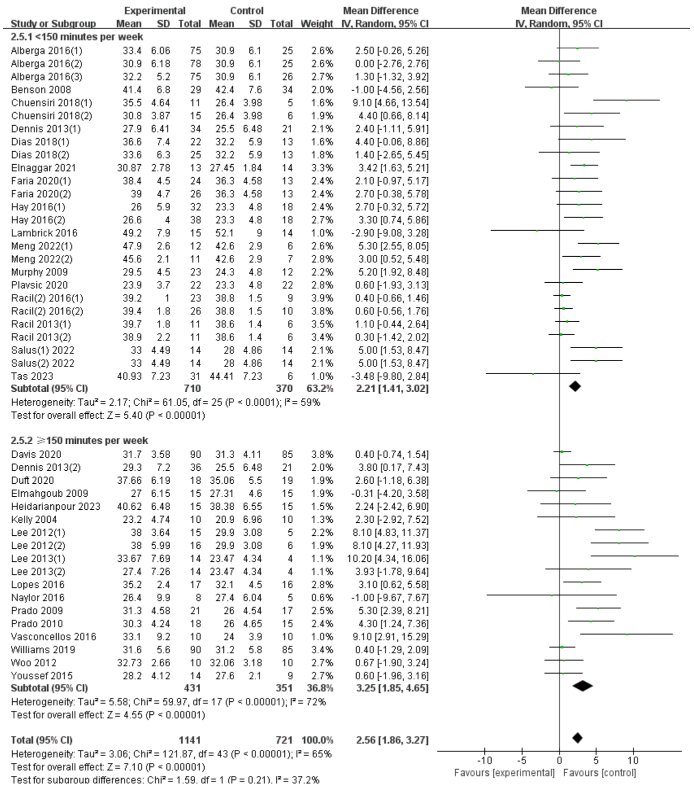

Figure S6.6. Forest plot of subgroup analyses by intervention duration

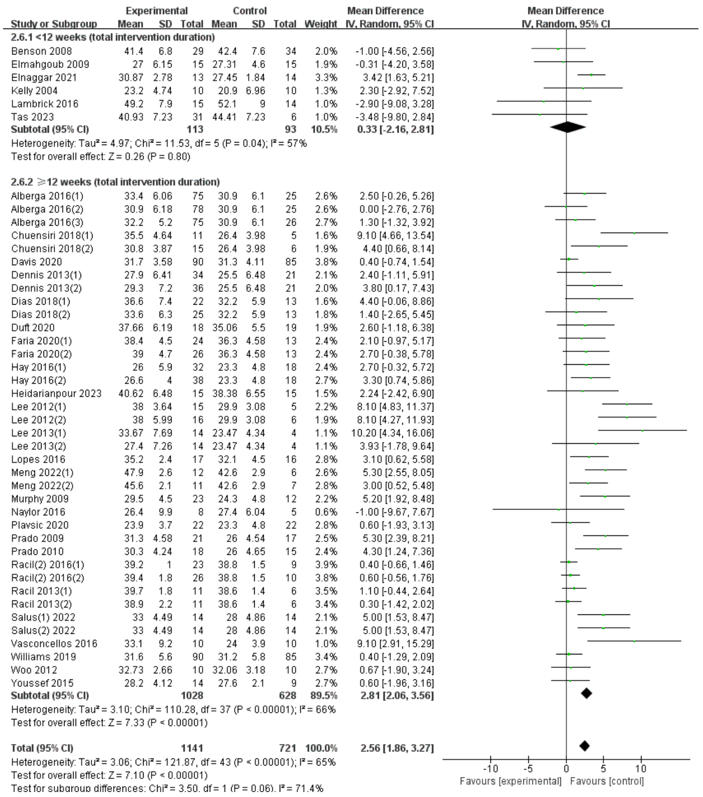

Figure S6.7. Forest plot of subgroup analyses by region

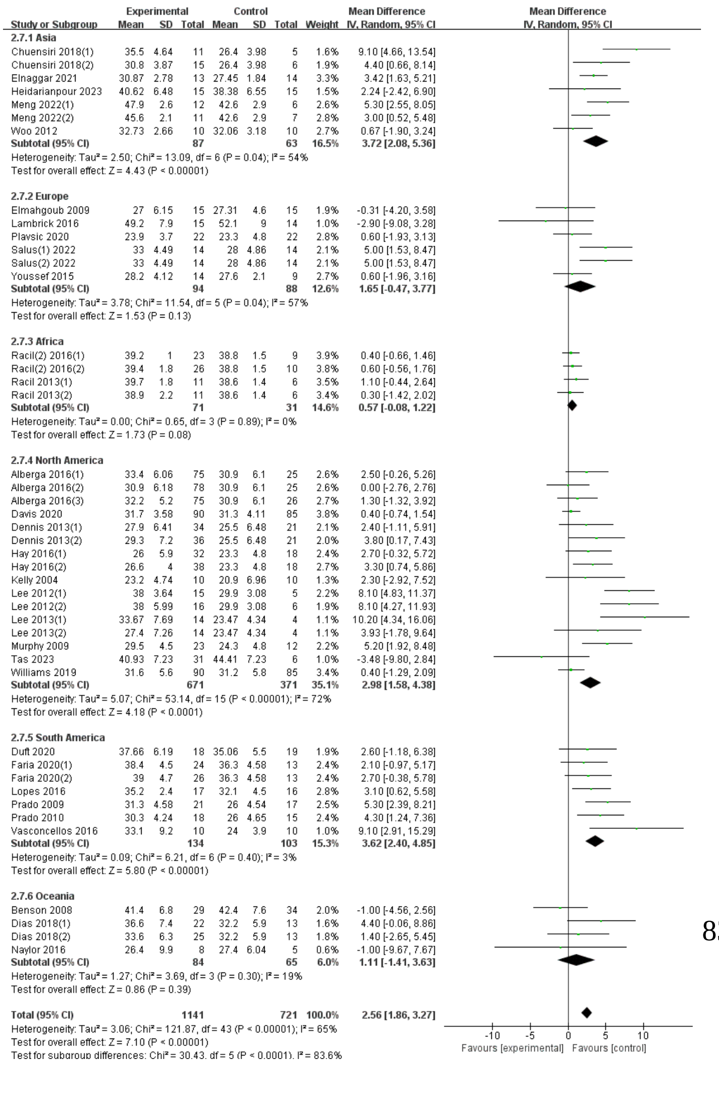

Figure S6.8. Forest plot of subgroup analyses by economic status

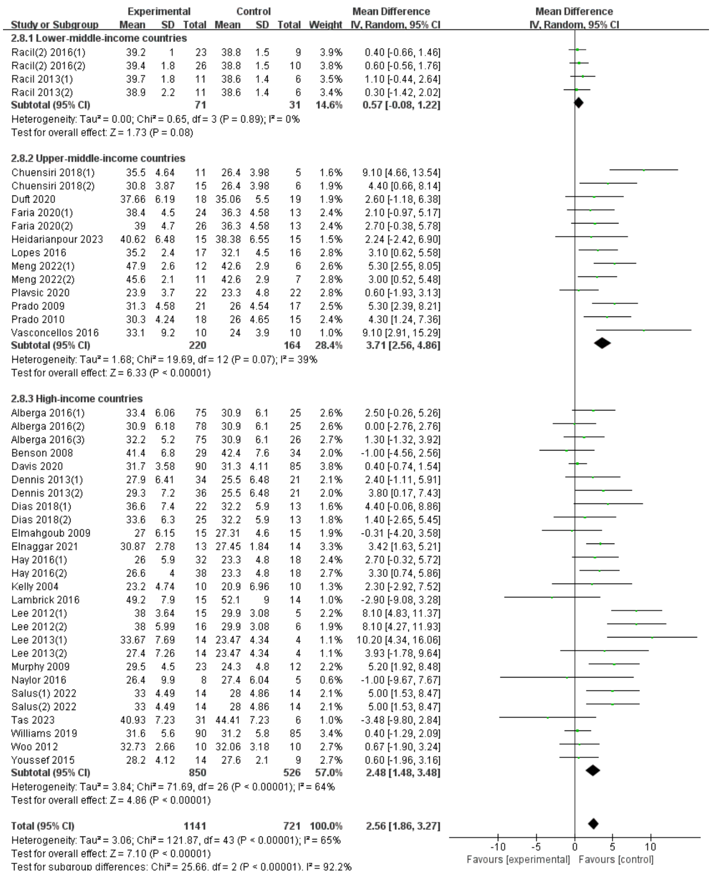

Figure S6.9. Forest plot of subgroup analyses by weight status

Study or Subgroup	Experimental Mean	Experimental SD	Experimental Total	Control Mean	Control SD	Control Total	Weight	Mean Difference IV, Random, 95% CI
**2.9.1 Overweight**								
Faria 2020(1)	38.4	4.5	24	36.3	4.58	13	3.2%	2.10 [-0.97, 5.17]
Faria 2020(2)	39	4.7	26	36.3	4.58	13	3.2%	2.70 [-0.38, 5.78]
Kelly 2004	23.2	4.74	10	20.9	6.96	10	1.9%	2.30 [-2.92, 7.52]
Lopes 2016	35.2	2.4	17	32.1	4.5	16	3.7%	3.10 [0.62, 5.58]
Murphy 2009	29.5	4.5	23	24.3	4.8	12	3.1%	5.20 [1.92, 8.48]
Woo 2012	32.73	2.66	10	32.06	3.18	10	3.7%	0.67 [-1.90, 3.24]
**Subtotal (95% CI)**			**110**			**74**	**18.8%**	**2.57 [1.33, 3.80]**
Heterogeneity: Tau² = 0.00; Chi² = 4.85, df = 5 (P = 0.43); I² = 0%								
Test for overall effect: Z = 4.07 (P < 0.0001)								
**2.9.2 Obesity**								
Alberga 2016(1)	33.4	6.06	75	30.9	6.1	25	3.5%	2.50 [-0.26, 5.26]
Alberga 2016(2)	30.9	6.18	78	30.9	6.1	25	3.5%	0.00 [-2.76, 2.76]
Alberga 2016(3)	32.2	5.2	75	30.9	6.1	26	3.6%	1.30 [-1.32, 3.92]
Chuensiri 2018(1)	35.5	4.64	11	26.4	3.98	5	2.3%	9.10 [4.66, 13.54]
Chuensiri 2018(2)	30.8	3.87	15	26.4	3.98	6	2.7%	4.40 [0.66, 8.14]
Dias 2018(1)	36.6	7.4	22	32.2	5.9	13	2.3%	4.40 [-0.06, 8.86]
Dias 2018(2)	33.6	6.3	25	32.2	5.9	13	2.5%	1.40 [-2.65, 5.45]
Elnaggar 2021	30.87	2.78	13	27.45	1.84	14	4.3%	3.42 [1.63, 5.21]
Lambrick 2016	49.2	7.9	15	52.1	9	14	1.5%	-2.90 [-9.08, 3.28]
Lee 2012(1)	38	3.64	15	29.9	3.08	5	3.1%	8.10 [4.83, 11.37]
Lee 2012(2)	38	5.99	16	29.9	3.08	6	2.7%	8.10 [4.27, 11.93]
Lee 2013(1)	33.67	7.69	14	23.47	4.34	4	1.6%	10.20 [4.34, 16.06]
Lee 2013(2)	27.4	7.26	14	23.47	4.34	4	1.7%	3.93 [-1.78, 9.64]
Meng 2022(1)	47.9	2.6	12	42.6	2.9	6	3.5%	5.30 [2.55, 8.05]
Meng 2022(2)	45.6	2.1	11	42.6	2.9	7	3.7%	3.00 [0.52, 5.48]
Naylor 2016	26.4	9.9	8	27.4	6.04	5	0.9%	-1.00 [-9.67, 7.67]
Plavsic 2020	23.9	3.7	22	23.3	4.8	22	3.7%	0.60 [-1.93, 3.13]
Prado 2009	31.3	4.58	21	26	4.54	17	3.4%	5.30 [2.39, 8.21]
Prado 2010	30.3	4.24	18	26	4.65	15	3.2%	4.30 [1.24, 7.36]
Racil(2) 2016(1)	39.2	1	23	38.8	1.5	9	4.9%	0.40 [-0.66, 1.46]
Racil(2) 2016(2)	39.4	1.8	26	38.8	1.5	10	4.8%	0.60 [-0.56, 1.76]
Racil 2013(1)	39.7	1.8	11	38.6	1.4	6	4.5%	1.10 [-0.44, 2.64]
Racil 2013(2)	38.9	2.2	11	38.6	1.4	6	4.4%	0.30 [-1.42, 2.02]
Salus(1) 2022	33	4.49	14	28	4.86	14	2.9%	5.00 [1.53, 8.47]
Salus(2) 2022	33	4.49	14	28	4.86	14	2.9%	5.00 [1.53, 8.47]
Tas 2023	40.93	7.23	31	44.41	7.23	6	1.4%	-3.48 [-9.80, 2.84]
Vasconcellos 2016	33.1	9.2	10	24	3.9	10	1.5%	9.10 [2.91, 15.29]
**Subtotal (95% CI)**			**620**			**307**	**81.2%**	**3.14 [2.09, 4.18]**
Heterogeneity: Tau² = 4.66; Chi² = 98.93, df = 26 (P < 0.00001); I² = 74%								
Test for overall effect: Z = 5.88 (P < 0.00001)								
**Total (95% CI)**			**730**			**381**	**100.0%**	**3.02 [2.12, 3.91]**
Heterogeneity: Tau² = 3.96; Chi² = 104.47, df = 32 (P < 0.00001); I² = 69%								
Test for overall effect: Z = 6.62 (P < 0.00001)								
Test for subgroup differences: Chi² = 0.48, df = 1 (P = 0.49), I² = 0%								

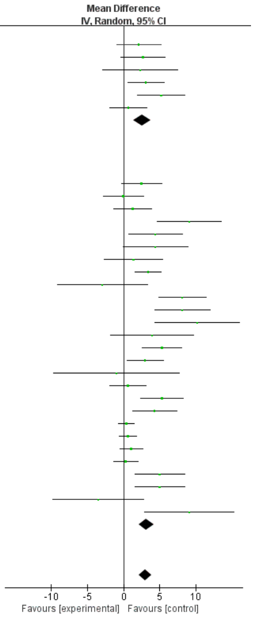

Figure S6.10. Forest plot of subgroup analyses by sex

Study or Subgroup	Experimental Mean	Experimental SD	Experimental Total	Control Mean	Control SD	Control Total	Weight	Mean Difference IV, Random, 95% CI
**2.10.1 Boys**								
Chuensiri 2018(1)	35.5	4.64	11	26.4	3.98	5	4.0%	9.10 [4.66, 13.54]
Chuensiri 2018(2)	30.8	3.87	15	26.4	3.98	6	4.8%	4.40 [0.66, 8.14]
Lee 2012(1)	38	3.64	15	29.9	3.08	5	5.3%	8.10 [4.83, 11.37]
Lee 2012(2)	38	5.99	16	29.9	3.08	6	4.7%	8.10 [4.27, 11.93]
Meng 2022(1)	47.9	2.6	12	42.6	2.9	6	5.9%	5.30 [2.55, 8.05]
Meng 2022(2)	45.6	2.1	11	42.6	2.9	7	6.3%	3.00 [0.52, 5.48]
Salus(1) 2022	33	4.49	14	28	4.86	14	5.1%	5.00 [1.53, 8.47]
Salus(2) 2022	33	4.49	14	28	4.86	14	5.1%	5.00 [1.53, 8.47]
**Subtotal (95% CI)**			**108**			**63**	**41.1%**	**5.70 [4.22, 7.19]**
Heterogeneity: Tau² = 1.69; Chi² = 11.14, df = 7 (P = 0.13); I² = 37%								
Test for overall effect: Z = 7.51 (P < 0.00001)								
**2.10.2 Girls**								
Heidarianpour 2023	40.62	6.48	15	38.38	6.55	15	3.8%	2.24 [-2.42, 6.90]
Lee 2013(1)	33.67	7.69	14	23.47	4.34	4	2.9%	10.20 [4.34, 16.06]
Lee 2013(2)	27.4	7.26	14	23.47	4.34	4	3.0%	3.93 [-1.78, 9.64]
Lopes 2016	35.2	2.4	17	32.1	4.5	16	6.3%	3.10 [0.62, 5.58]
Plavsic 2020	23.9	3.7	22	23.3	4.8	22	6.2%	0.60 [-1.93, 3.13]
Racil(2) 2016(1)	39.2	1	23	38.8	1.5	9	7.9%	0.40 [-0.66, 1.46]
Racil(2) 2016(2)	39.4	1.8	26	38.8	1.5	10	7.8%	0.60 [-0.56, 1.76]
Racil 2013(1)	39.7	1.8	11	38.6	1.4	6	7.4%	1.10 [-0.44, 2.64]
Racil 2013(2)	38.9	2.2	11	38.6	1.4	6	7.2%	0.30 [-1.42, 2.02]
Youssef 2015	28.2	4.12	14	27.6	2.1	9	6.2%	0.60 [-1.96, 3.16]
**Subtotal (95% CI)**			**167**			**101**	**58.9%**	**1.14 [0.26, 2.03]**
Heterogeneity: Tau² = 0.74; Chi² = 15.84, df = 9 (P = 0.07); I² = 43%								
Test for overall effect: Z = 2.52 (P = 0.01)								
**Total (95% CI)**			**275**			**164**	**100.0%**	**3.35 [2.11, 4.59]**
Heterogeneity: Tau² = 4.78; Chi² = 76.34, df = 17 (P < 0.00001); I² = 78%								
Test for overall effect: Z = 5.29 (P < 0.00001)								
Test for subgroup differences: Chi² = 26.63, df = 1 (P < 0.00001), I² = 96.2%								

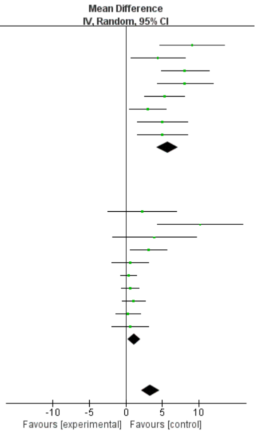

Figure S6.11. Forest plot of subgroup analyses by metabolic status

Study or Subgroup	Experimental Mean	Experimental SD	Experimental Total	Control Mean	Control SD	Control Total	Weight	Mean Difference IV, Random, 95% CI
**2.11.1 Metabolically healthy obesity**								
Davis 2020	31.7	3.58	90	31.3	4.11	85	19.0%	0.40 [-0.74, 1.54]
Faria 2020(1)	38.4	4.5	24	36.3	4.58	13	13.0%	2.10 [-0.97, 5.17]
Faria 2020(2)	39	4.7	26	36.3	4.58	13	13.0%	2.70 [-0.38, 5.78]
**Subtotal (95% CI)**			**140**			**111**	**45.0%**	**1.11 [-0.30, 2.51]**
Heterogeneity: Tau² = 0.45; Chi² = 2.62, df = 2 (P = 0.27); I² = 24%								
Test for overall effect: Z = 1.54 (P = 0.12)								
**2.11.2 Metabolically unhealthy obesity**								
Kelly 2004	23.2	4.74	10	20.9	6.96	10	7.7%	2.30 [-2.92, 7.52]
Meng 2022(1)	47.9	2.6	12	42.6	2.9	6	14.0%	5.30 [2.55, 8.05]
Meng 2022(2)	45.6	2.1	11	42.6	2.9	7	14.9%	3.00 [0.52, 5.48]
Murphy 2009	29.5	4.5	23	24.3	4.8	12	12.3%	5.20 [1.92, 8.48]
Vasconcellos 2016	33.1	9.2	10	24	3.9	10	6.1%	9.10 [2.91, 15.29]
**Subtotal (95% CI)**			**66**			**45**	**55.0%**	**4.48 [2.82, 6.15]**
Heterogeneity: Tau² = 0.55; Chi² = 4.70, df = 4 (P = 0.32); I² = 15%								
Test for overall effect: Z = 5.28 (P < 0.00001)								
**Total (95% CI)**			**206**			**156**	**100.0%**	**3.26 [1.44, 5.09]**
Heterogeneity: Tau² = 4.21; Chi² = 22.59, df = 7 (P = 0.002); I² = 69%								
Test for overall effect: Z = 3.51 (P = 0.0005)								
Test for subgroup differences: Chi² = 9.24, df = 1 (P = 0.002), I² = 89.2%								

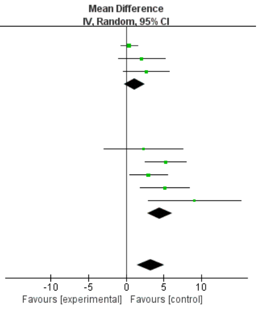

Figure S7. Forest plots of subgroup analyses for HRmax in children and adolescents with overweight or obesity

Figure S7.1. Forest plot of subgroup analyses by type of exercise

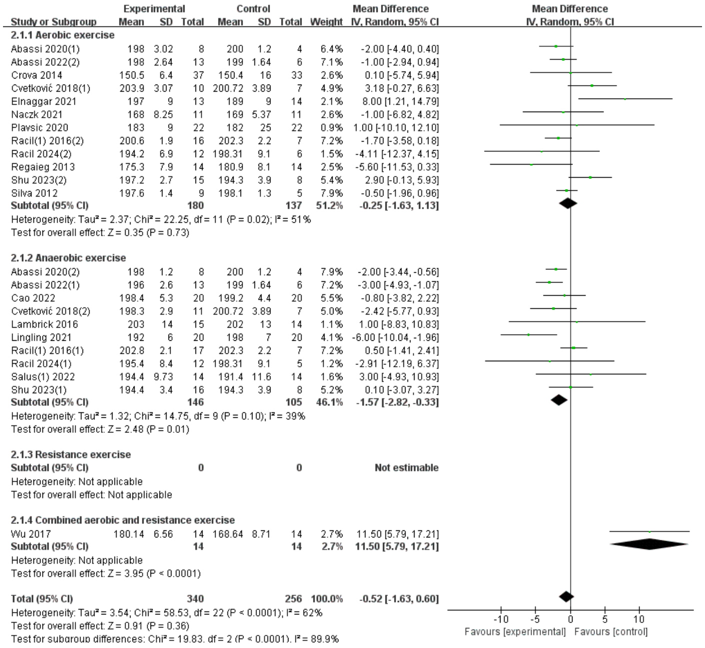

Figure S7.2. Forest plot of subgroup analyses by exercise intensity

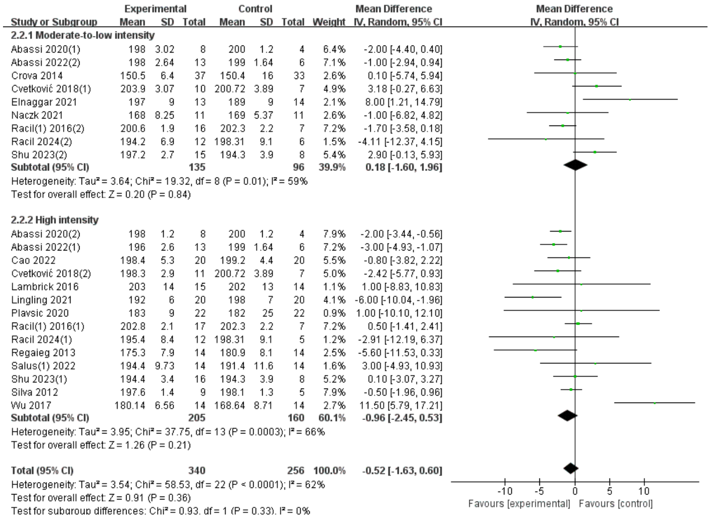

Figure S7.3. Forest plot of subgroup analyses by duration per session

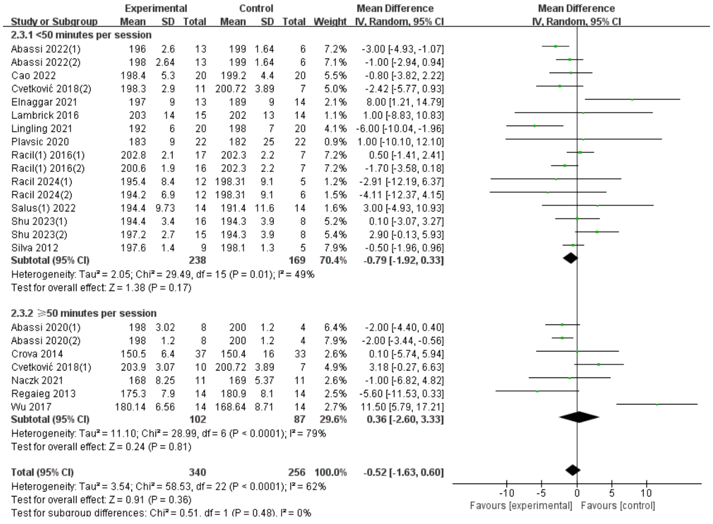

Figure S7.4. Forest plot of subgroup analyses by exercise frequency

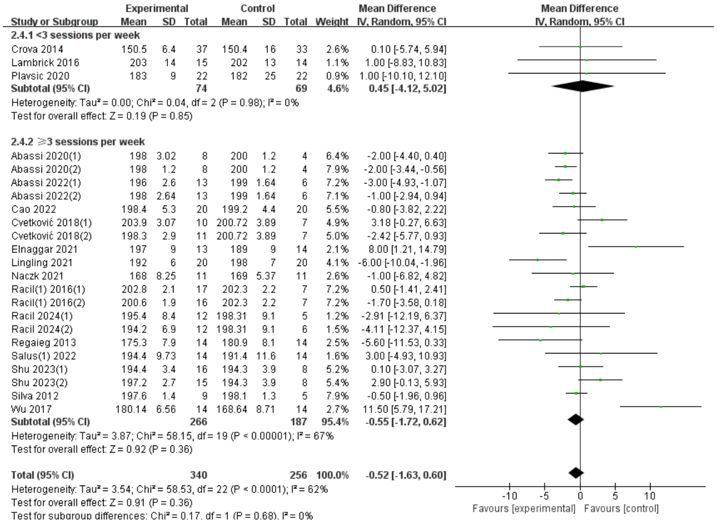

Figure S7.5. Forest plot of subgroup analyses by weekly exercise duration

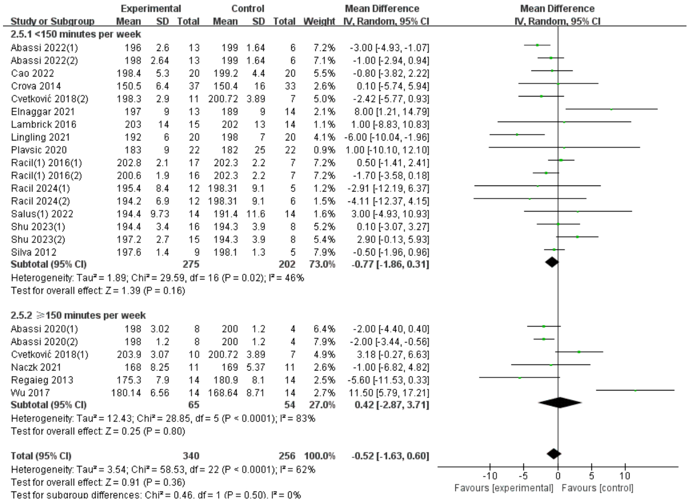

Figure S7.6. Forest plot of subgroup analyses by intervention duration

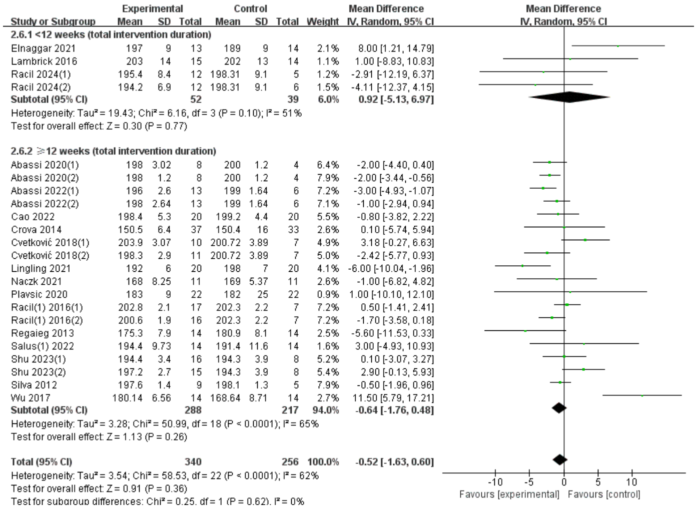

Figure S7.7. Forest plot of subgroup analyses by region

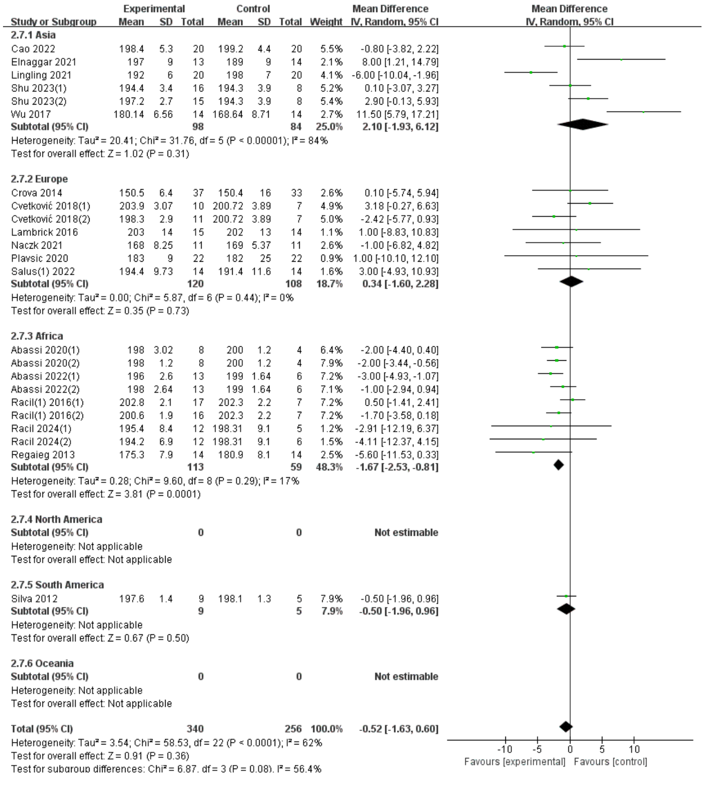

Figure S7.8. Forest plot of subgroup analyses by economic status

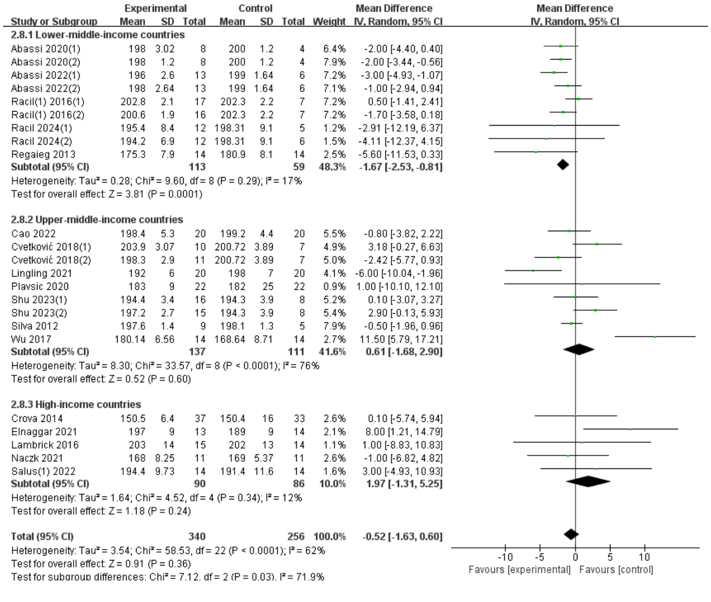

Figure S7.9. Forest plot of subgroup analyses by weight status

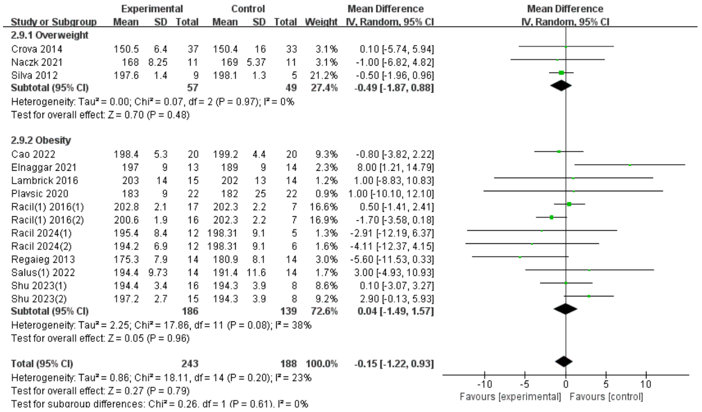

Figure S7.10. Forest plot of subgroup analyses by sex

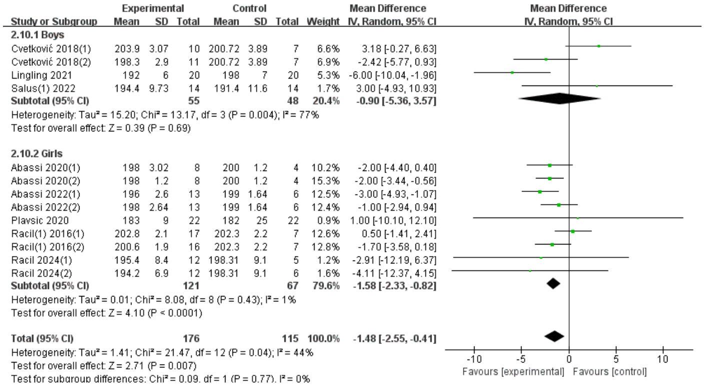

Figure S8. Forest plots of subgroup analyses for HRrest in children and adolescents with overweight or obesity

Figure S8.1. Forest plot of subgroup analyses by type of exercise

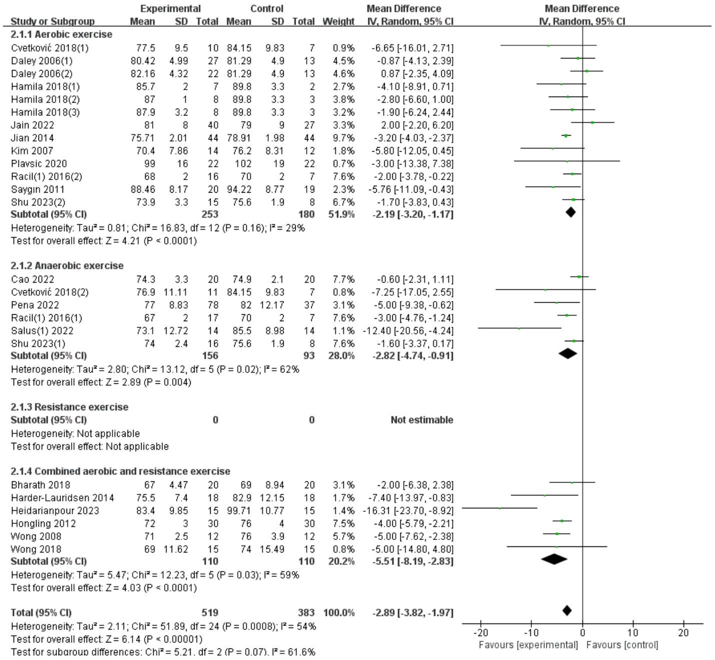

Figure S8.2. Forest plot of subgroup analyses by exercise intensity

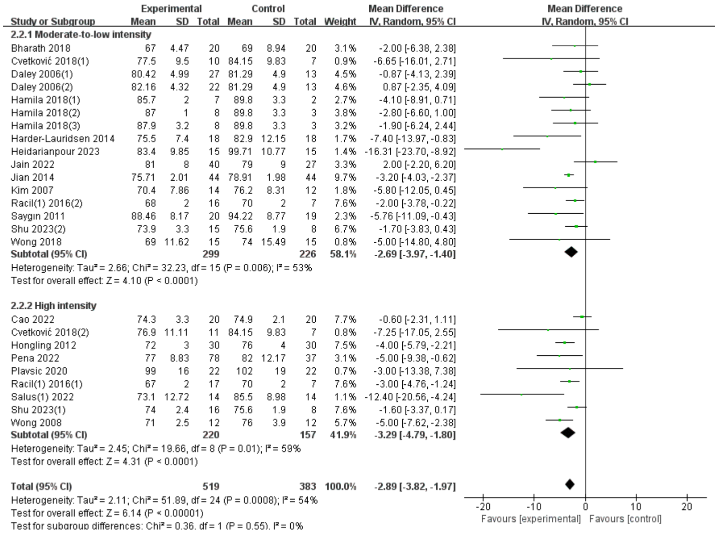

Figure S8.3. Forest plot of subgroup analyses by duration per session

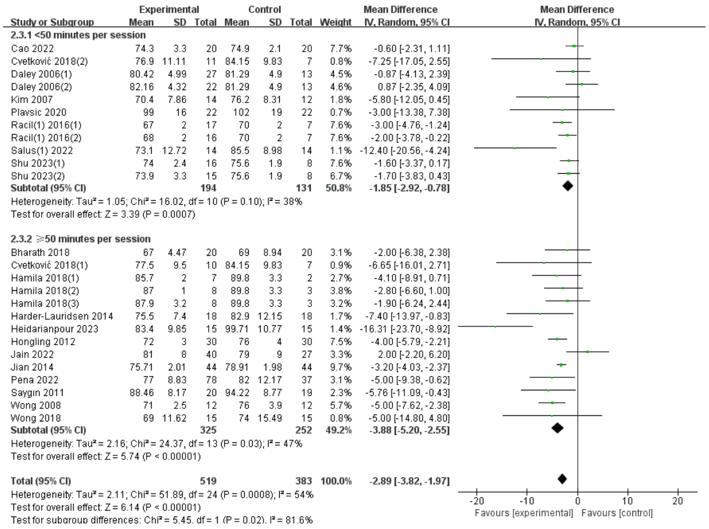

Figure S8.4. Forest plot of subgroup analyses by exercise frequency

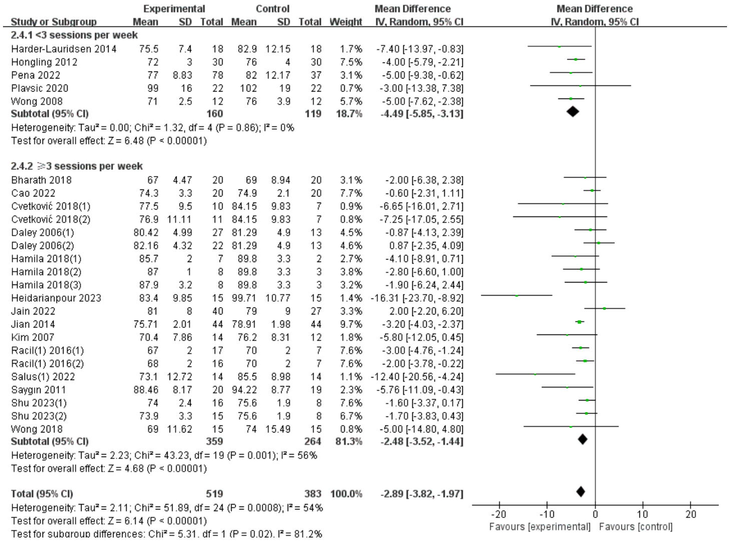

Figure S8.5. Forest plot of subgroup analyses by weekly exercise duration

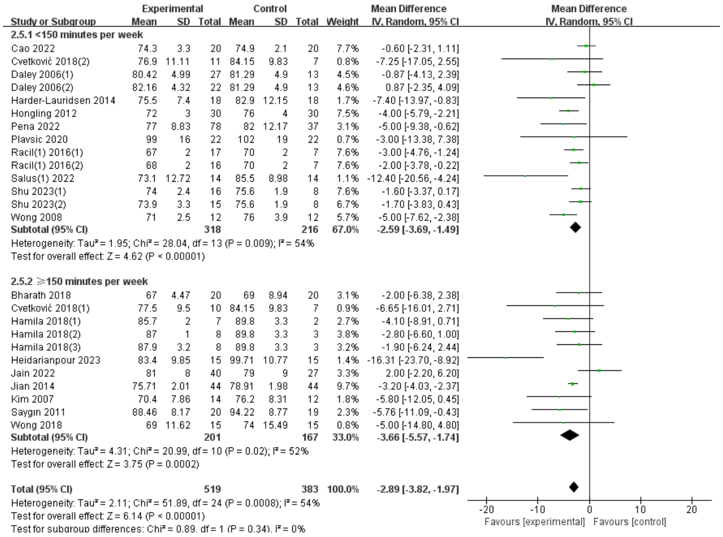

Figure S8.6. Forest plot of subgroup analyses by intervention duration

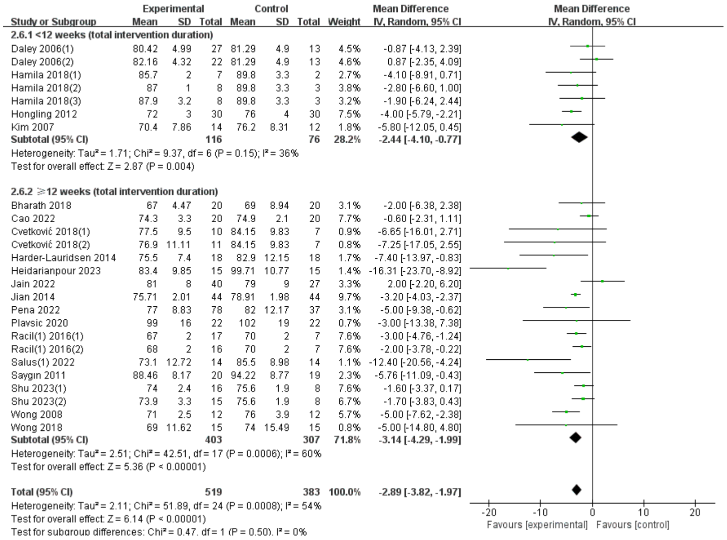

Figure S8.7. Forest plot of subgroup analyses by region

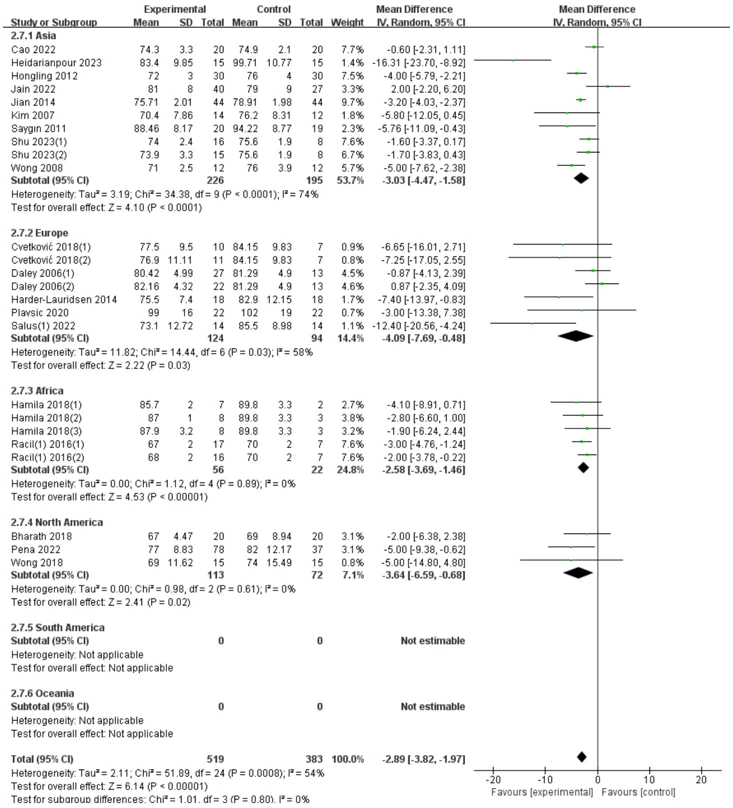

Figure S8.8. Forest plot of subgroup analyses by economic status

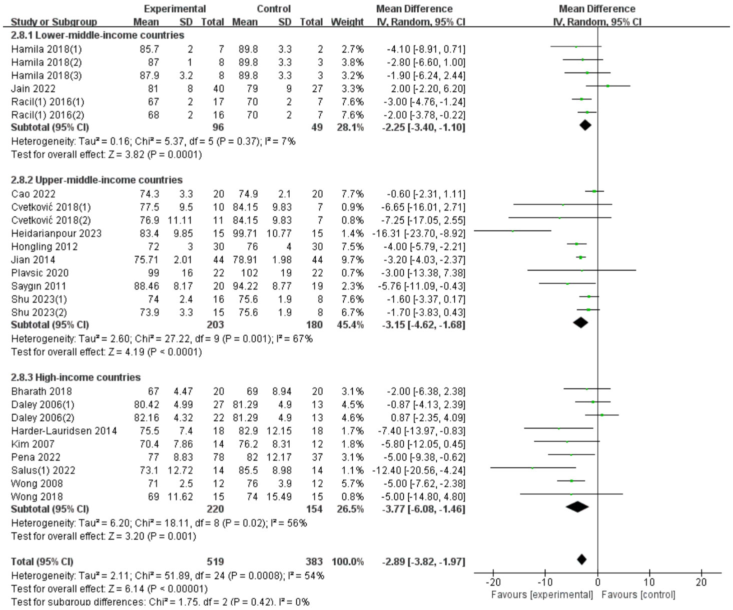

Figure S8.9. Forest plot of subgroup analyses by weight status

Study or Subgroup	Experimental Mean	Experimental SD	Experimental Total	Control Mean	Control SD	Control Total	Weight	Mean Difference IV, Random, 95% CI
**2.9.1 Overweight**								
Harder-Lauridsen 2014	75.5	7.4	18	82.9	12.15	18	1.6%	-7.40 [-13.97, -0.83]
**Subtotal (95% CI)**			**18**			**18**	**1.6%**	**-7.40 [-13.97, -0.83]**
Heterogeneity: Not applicable								
Test for overall effect: Z = 2.21 (P = 0.03)								
**2.9.2 Obesity**								
Bharath 2018	67	4.47	20	69	8.94	20	3.1%	-2.00 [-6.38, 2.38]
Cao 2022	74.3	3.3	20	74.9	2.1	20	9.7%	-0.60 [-2.31, 1.11]
Daley 2006(1)	80.42	4.99	27	81.29	4.9	13	4.9%	-0.87 [-4.13, 2.39]
Daley 2006(2)	82.16	4.32	22	81.29	4.9	13	5.0%	0.87 [-2.35, 4.09]
Hongling 2012	72	3	30	76	4	30	9.4%	-4.00 [-5.79, -2.21]
Jian 2014	75.71	2.01	44	78.91	1.98	44	13.7%	-3.20 [-4.03, -2.37]
Kim 2007	70.4	7.86	14	76.2	8.31	12	1.7%	-5.80 [-12.05, 0.45]
Pena 2022	77	8.83	78	82	12.17	37	3.1%	-5.00 [-9.38, -0.62]
Plavsic 2020	99	16	22	102	19	22	0.7%	-3.00 [-13.38, 7.38]
Racil(1) 2016(1)	67	2	17	70	2	7	9.5%	-3.00 [-4.76, -1.24]
Racil(1) 2016(2)	68	2	16	70	2	7	9.5%	-2.00 [-3.78, -0.22]
Salus(1) 2022	73.1	12.72	14	85.5	8.98	14	1.1%	-12.40 [-20.56, -4.24]
Saygın 2011	88.46	8.17	20	94.22	8.77	19	2.3%	-5.76 [-11.09, -0.43]
Shu 2023(1)	74	2.4	16	75.6	1.9	8	9.5%	-1.60 [-3.37, 0.17]
Shu 2023(2)	73.9	3.3	15	75.6	1.9	8	8.1%	-1.70 [-3.83, 0.43]
Wong 2008	71	2.5	12	76	3.9	12	6.5%	-5.00 [-7.62, -2.38]
Wong 2018	69	11.62	15	74	15.49	15	0.7%	-5.00 [-14.80, 4.80]
**Subtotal (95% CI)**			**402**			**301**	**98.4%**	**-2.63 [-3.50, -1.77]**
Heterogeneity: Tau² = 1.19; Chi² = 30.14, df = 16 (P = 0.02); I² = 47%								
Test for overall effect: Z = 5.99 (P < 0.00001)								
**Total (95% CI)**			**420**			**319**	**100.0%**	**-2.71 [-3.58, -1.85]**
Heterogeneity: Tau² = 1.26; Chi² = 32.09, df = 17 (P = 0.01); I² = 47%								
Test for overall effect: Z = 6.12 (P < 0.00001)								
Test for subgroup differences: Chi² = 1.99, df = 1 (P = 0.16), I² = 49.6%								

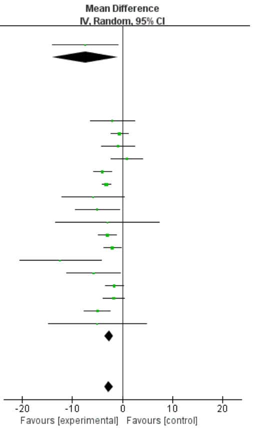

Figure S8.10. Forest plot of subgroup analyses by sex

Study or Subgroup	Experimental Mean	Experimental SD	Experimental Total	Control Mean	Control SD	Control Total	Weight	Mean Difference IV, Random, 95% CI
**2.10.1 Boys**								
Cvetković 2018(1)	77.5	9.5	10	84.15	9.83	7	1.5%	-6.65 [-16.01, 2.71]
Cvetković 2018(2)	76.9	11.11	11	84.15	9.83	7	1.4%	-7.25 [-17.05, 2.55]
Hongling 2012	72	3	30	76	4	30	15.2%	-4.00 [-5.79, -2.21]
Kim 2007	70.4	7.86	14	76.2	8.31	12	3.1%	-5.80 [-12.05, 0.45]
Salus(1) 2022	73.1	12.72	14	85.5	8.98	14	1.9%	-12.40 [-20.56, -4.24]
Wong 2008	71	2.5	12	76	3.9	12	10.9%	-5.00 [-7.62, -2.38]
**Subtotal (95% CI)**			**91**			**82**	**33.9%**	**-4.73 [-6.12, -3.35]**
Heterogeneity: Tau² = 0.00; Chi² = 4.61, df = 5 (P = 0.47); I² = 0%								
Test for overall effect: Z = 6.69 (P < 0.00001)								
**2.10.2 Girls**								
Bharath 2018	67	4.47	20	69	8.94	20	5.5%	-2.00 [-6.38, 2.38]
Heidarianpour 2023	83.4	9.85	15	99.71	10.77	15	2.3%	-16.31 [-23.70, -8.92]
Jian 2014	75.71	2.01	44	78.91	1.98	44	21.0%	-3.20 [-4.03, -2.37]
Plavsic 2020	99	16	22	102	19	22	1.2%	-3.00 [-13.38, 7.38]
Racil(1) 2016(1)	67	2	17	70	2	7	15.4%	-3.00 [-4.76, -1.24]
Racil(1) 2016(2)	68	2	16	70	2	7	15.3%	-2.00 [-3.78, -0.22]
Saygın 2011	88.46	8.17	20	94.22	8.77	19	4.1%	-5.76 [-11.09, -0.43]
Wong 2018	69	11.62	15	74	15.49	15	1.4%	-5.00 [-14.80, 4.80]
**Subtotal (95% CI)**			**169**			**149**	**66.1%**	**-3.47 [-5.00, -1.95]**
Heterogeneity: Tau² = 1.82; Chi² = 15.17, df = 7 (P = 0.03); I² = 54%								
Test for overall effect: Z = 4.46 (P < 0.00001)								
**Total (95% CI)**			**260**			**231**	**100.0%**	**-4.03 [-5.21, -2.85]**
Heterogeneity: Tau² = 1.55; Chi² = 23.95, df = 13 (P = 0.03); I² = 46%								
Test for overall effect: Z = 6.69 (P < 0.00001)								
Test for subgroup differences: Chi² = 1.44, df = 1 (P = 0.23), I² = 30.4%								

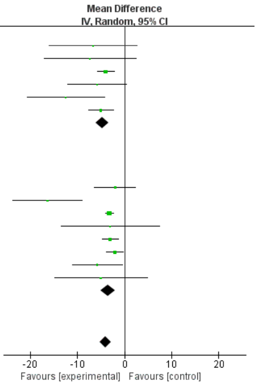

Figure S9. Forest plots of subgroup analyses for SBP in children and adolescents with overweight or obesity

Figure S9.1. Forest plot of subgroup analyses by type of exercise

Study or Subgroup	Experimental Mean	Experimental SD	Experimental Total	Control Mean	Control SD	Control Total	Weight	Mean Difference IV, Random, 95% CI
**2.1.1 Aerobic exercise**								
Abassi 2022(2)	123	3.8	13	125	3.8	6	1.6%	-2.00 [-5.68, 1.68]
Aguilar-Cordero 2020	124.69	5.72	49	131.16	12.24	49	1.6%	-6.47 [-10.25, -2.69]
Bezerra 2023	111.15	11.3	20	111.26	6.41	21	1.4%	-0.11 [-5.77, 5.55]
Boer 2014(2)	119	9	15	119	10	7	1.1%	0.00 [-8.70, 8.70]
Boff 2020	122.84	10.37	34	121	11.08	31	1.4%	1.84 [-3.39, 7.07]
Cvetković 2018(1)	117.5	11.12	10	124.85	11.58	7	0.9%	-7.35 [-18.35, 3.65]
Davis 2020	105.1	6.88	90	102.3	6	85	1.7%	2.80 [0.89, 4.71]
Guijun 2009	114.97	1.5	16	123.15	2.7	10	1.7%	-8.18 [-10.01, -6.35]
Hamila 2018(1)	116.4	7.1	7	125.5	11.2	2	0.5%	-9.10 [-25.49, 7.29]
Hamila 2018(2)	118.6	5.7	8	125.5	11.2	3	0.7%	-6.90 [-20.17, 6.37]
Hamila 2018(3)	121.8	6.2	8	125.5	11.2	3	0.7%	-3.70 [-17.08, 9.68]
Horner 2015(1)	109.58	19.56	27	116.63	10.23	8	0.9%	-7.05 [-17.28, 3.18]
Jain 2022	114	8	40	116	8	27	1.6%	-2.00 [-5.91, 1.91]
Jian 2014	120.55	2.56	44	129.72	2.24	44	1.8%	-9.17 [-10.18, -8.16]
Kai 2024	127	1	20	132	3	24	1.8%	-5.00 [-6.28, -3.72]
Kelishadi 2008	116.9	45.88	45	116.7	45.9	47	0.4%	0.20 [-18.56, 18.96]
Kelly 2004	120	6.32	10	122	15.81	10	0.9%	-2.00 [-12.55, 8.55]
Kim 2007	122.9	7.11	14	124.2	10.74	12	1.2%	-1.30 [-8.43, 5.83]
Kim 2011	114	10.39	12	118	15.81	10	0.8%	-4.00 [-15.43, 7.43]
Kim 2020	127.6	2.3	24	133.2	4.1	24	1.7%	-5.60 [-7.48, -3.72]
Larsen 2016	100.1	6.9	51	103.1	7.7	44	1.7%	-3.00 [-5.96, -0.04]
Lee 2010(2)	116.53	10.99	16	121.12	16.03	9	0.8%	-4.59 [-16.37, 7.19]
Liqun 2014	106.5	12.5	76	109.8	9.3	97	1.6%	-3.30 [-6.66, 0.06]
Luo 2013(1)	100.72	6.21	43	112.21	8.26	34	1.6%	-11.49 [-14.83, -8.15]
Luo 2013(2)	97.5	6.22	52	107.89	9.11	38	1.6%	-10.39 [-13.74, -7.04]
Machado 2022	100.3	4.8	20	110.2	13.3	15	1.3%	-9.90 [-16.95, -2.85]
Meng 2022(2)	114	7	11	116	4	7	1.5%	-2.00 [-7.09, 3.09]
Meyer 2006	120	13.3	33	133	19.6	34	1.2%	-13.00 [-21.00, -5.00]
Murphy 2009	111.4	8	23	114.2	5.7	12	1.5%	-2.80 [-7.39, 1.79]
Park 2007	112	7.6	19	119.3	7.5	21	1.5%	-7.30 [-11.99, -2.61]
Plavsic 2020	110.3	8.5	22	114.4	6.8	22	1.5%	-4.10 [-8.65, 0.45]
Racil(1) 2016(2)	115	5	16	118	5	7	1.5%	-3.00 [-7.44, 1.44]
Racil 2024(2)	119.33	4.7	12	119.1	4.7	6	1.5%	0.23 [-4.38, 4.84]
Savoye 2007	121	11	75	120.4	12.54	44	1.5%	0.60 [-3.86, 5.06]
Savoye 2011	121.7	10.24	75	121.7	10.82	44	1.6%	0.00 [-3.95, 3.95]
Saygın 2011	121.6	1.03	20	135.1	0.84	19	1.8%	-13.50 [-14.09, -12.91]
Seo 2019	123.06	11.07	26	123.91	15.95	44	1.3%	-0.85 [-7.20, 5.50]
Shu 2023(2)	113.2	6.3	15	117.1	5	8	1.5%	-3.90 [-8.61, 0.81]
Sigal 2014(1)	111	8.66	75	110	8.72	25	1.6%	1.00 [-2.94, 4.94]
Tjonna 2009	119.4	7.16	20	122.5	7.04	22	1.5%	-3.10 [-7.40, 1.20]
Woo 2012	103.05	7.75	10	116.3	7.18	10	1.3%	-13.25 [-19.80, -6.70]
Yu 2020	102.7	8.1	99	102.1	8.9	72	1.7%	0.60 [-2.00, 3.20]
Yuanyuan 2019(1)	105.47	7.21	10	106.57	7.62	3	1.0%	-1.10 [-10.81, 8.61]
Yuanyuan 2019(2)	99.01	7.24	10	106.57	7.62	3	1.0%	-7.56 [-17.28, 2.16]
Zhaogang 2021	98.24	9.64	49	101.24	10.04	49	1.6%	-3.00 [-6.90, 0.90]
**Subtotal (95% CI)**			**1384**			**1119**	**60.8%**	**-4.26 [-6.13, -2.39]**
Heterogeneity: Tau² = 31.08; Chi² = 704.06, df = 44 (P < 0.00001); I² = 94%								
Test for overall effect: Z = 4.47 (P < 0.00001)								
**2.1.2 Anaerobic exercise**								
Abassi 2022(1)	122	2.3	13	125	3.8	6	1.6%	-3.00 [-6.29, 0.29]
Boer 2014(1)	113	8	17	119	10	7	1.1%	-6.00 [-14.33, 2.33]
Chuensiri 2018(1)	125	13.27	11	121	13.27	5	0.7%	4.00 [-10.03, 18.03]
Chuensiri 2018(2)	116	11.62	15	121	13.27	6	0.8%	-5.00 [-17.14, 7.14]
Cvetković 2018(2)	116.5	14.15	11	124.85	11.58	7	0.8%	-8.35 [-20.33, 3.63]
Espinoza-Silva 2019(1)	103.06	13.53	69	102.6	10.14	30	1.5%	0.46 [-4.37, 5.29]
Espinoza-Silva 2019(2)	103.03	8.49	141	102.91	8.9	34	1.6%	0.12 [-3.18, 3.42]
Gonzalez-Galvez 2024(1)	123	11.4	9	126.5	13.16	6	0.7%	-3.50 [-16.40, 9.40]
Gonzalez-Galvez 2024(2)	116.3	11.28	11	126.5	13.16	6	0.8%	-10.20 [-22.66, 2.26]
Meng 2022(1)	110	5	12	116	4	6	1.5%	-6.00 [-10.27, -1.73]
Racil(1) 2016(1)	115	5	17	118	5	7	1.5%	-3.00 [-7.40, 1.40]
Racil 2024(1)	119.15	5	12	119.1	4.7	5	1.5%	0.05 [-4.95, 5.05]
Salus(1) 2022	125.9	8.98	14	120.7	16.46	14	1.0%	5.20 [-4.62, 15.02]
Shu 2023(1)	111.9	4.7	16	117.1	5	8	1.6%	-5.20 [-9.36, -1.04]
Vasconcellos 2016	123	13	10	129	6	10	1.1%	-6.00 [-14.87, 2.87]
Yuanyuan 2019(3)	97.65	6.62	9	106.57	7.62	4	1.1%	-8.92 [-17.55, -0.29]
**Subtotal (95% CI)**			**387**			**161**	**18.8%**	**-2.88 [-4.52, -1.24]**
Heterogeneity: Tau² = 1.84; Chi² = 18.22, df = 15 (P = 0.25); I² = 18%								
Test for overall effect: Z = 3.44 (P = 0.0006)								
**2.1.3 Resistance exercise**								
Horner 2015(2)	110.89	8.04	23	116.63	10.23	8	1.2%	-5.74 [-13.55, 2.07]
Sigal 2014(2)	112	8.83	78	110	8.72	25	1.6%	2.00 [-1.94, 5.94]
**Subtotal (95% CI)**			**101**			**33**	**2.7%**	**-1.10 [-8.54, 6.33]**
Heterogeneity: Tau² = 19.99; Chi² = 3.01, df = 1 (P = 0.08); I² = 67%								
Test for overall effect: Z = 0.29 (P = 0.77)								
**2.1.4 Combined aerobic and resistance exercise**								
Bharath 2018	120	1.79	20	124	6.26	20	1.7%	-4.00 [-6.85, -1.15]
Bruyndonckx 2015	108	6	27	120	11	21	1.4%	-12.00 [-17.22, -6.78]
Faria 2020(1)	115.7	8.05	24	117.3	8.29	13	1.4%	-1.60 [-7.14, 3.94]
Faria 2020(2)	114.5	8.29	26	117.3	8.29	13	1.4%	-2.80 [-8.32, 2.72]
Farpour-Lambert 2009	120.7	13.69	22	125.7	14.85	22	1.1%	-5.00 [-13.44, 3.44]
Harder-Lauridsen 2014	104.5	9.38	18	102.3	9.98	18	1.3%	2.20 [-4.13, 8.53]
Heidarianpour 2023	108.22	12.39	15	118.2	13.47	15	1.0%	-9.98 [-19.24, -0.72]
Hongling 2012	113	5	30	116	6	30	1.7%	-3.00 [-5.79, -0.21]
Lee 2010(1)	119.34	15.19	20	121.12	16.03	9	0.8%	-1.78 [-14.19, 10.63]
Shalitin 2009	111	17.8	55	112	18.54	55	1.3%	-1.00 [-7.79, 5.79]
Sigal 2014(3)	110	8.66	75	110	8.72	26	1.6%	0.00 [-3.88, 3.88]
Son 2017	123.7	9.53	20	130.6	6.22	20	1.5%	-6.90 [-11.89, -1.91]
Wong 2008	113.8	7.1	12	117	6.2	12	1.4%	-3.20 [-8.53, 2.13]
**Subtotal (95% CI)**			**364**			**274**	**17.6%**	**-3.62 [-5.56, -1.69]**
Heterogeneity: Tau² = 5.01; Chi² = 21.57, df = 12 (P = 0.04); I² = 44%								
Test for overall effect: Z = 3.67 (P = 0.0002)								
**Total (95% CI)**			**2236**			**1587**	**100.0%**	**-3.90 [-5.34, -2.46]**
Heterogeneity: Tau² = 30.25; Chi² = 876.13, df = 75 (P < 0.00001); I² = 91%								
Test for overall effect: Z = 5.31 (P < 0.00001)								
Test for subgroup differences: Chi² = 1.60, df = 3 (P = 0.66), I² = 0%								

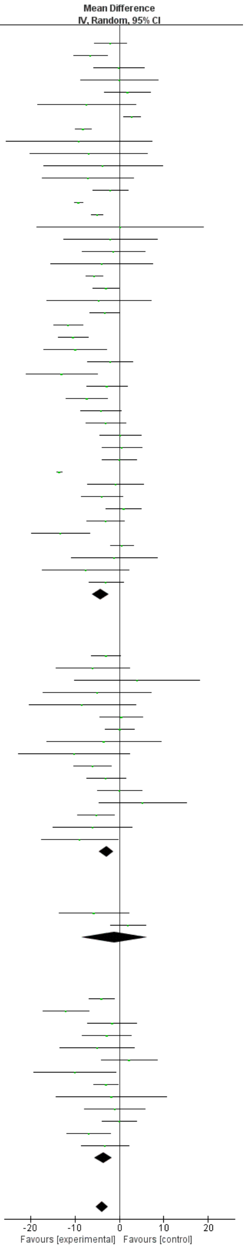

Figure S9.2. Forest plot of subgroup analyses by exercise intensity

Study or Subgroup	Experimental Mean	Experimental SD	Experimental Total	Control Mean	Control SD	Control Total	Weight	Mean Difference IV, Random, 95% CI
**2.2.1 Moderate-to-low intensity**								
Abassi 2022(2)	123	3.8	13	125	3.8	6	1.6%	-2.00 [-5.68, 1.68]
Aguilar-Cordero 2020	124.69	5.72	49	131.16	12.24	49	1.6%	-6.47 [-10.25, -2.69]
Bharath 2018	120	1.79	20	124	6.26	20	1.7%	-4.00 [-6.85, -1.15]
Boer 2014(2)	119	9	15	119	10	7	1.1%	0.00 [-8.70, 8.70]
Boff 2020	122.84	10.37	34	121	11.08	31	1.4%	1.84 [-3.39, 7.07]
Bruyndonckx 2015	108	6	27	120	11	21	1.4%	-12.00 [-17.22, -6.78]
Cvetković 2018(1)	117.5	11.12	10	124.85	11.58	7	0.9%	-7.35 [-18.35, 3.65]
Davis 2020	105.1	6.88	90	102.3	6	85	1.7%	2.80 [0.89, 4.71]
Faria 2020(1)	115.7	8.05	24	117.3	8.29	13	1.4%	-1.60 [-7.14, 3.94]
Farpour-Lambert 2009	120.7	13.69	22	125.7	14.85	22	1.1%	-5.00 [-13.44, 3.44]
Guijun 2009	114.97	1.5	16	123.15	2.7	10	1.7%	-8.18 [-10.01, -6.35]
Hamila 2018(1)	116.4	7.1	7	125.5	11.2	2	0.5%	-9.10 [-25.49, 7.29]
Hamila 2018(2)	118.6	5.7	8	125.5	11.2	3	0.7%	-6.90 [-20.17, 6.37]
Hamila 2018(3)	121.8	6.2	8	125.5	11.2	3	0.7%	-3.70 [-17.08, 9.68]
Harder-Lauridsen 2014	104.5	9.38	18	102.3	9.98	18	1.3%	2.20 [-4.13, 8.53]
Heidarianpour 2023	108.22	12.39	15	118.2	13.47	15	1.0%	-9.98 [-19.24, -0.72]
Horner 2015(1)	109.58	19.56	27	116.63	10.23	8	0.9%	-7.05 [-17.28, 3.18]
Jain 2022	114	8	40	116	8	27	1.6%	-2.00 [-5.91, 1.91]
Jian 2014	120.55	2.56	44	129.72	2.24	44	1.8%	-9.17 [-10.18, -8.16]
Kai 2024	127	1	20	132	3	24	1.8%	-5.00 [-6.28, -3.72]
Kelishadi 2008	116.9	45.88	45	116.7	45.9	47	0.4%	0.20 [-18.56, 18.96]
Kelly 2004	120	6.32	10	122	15.81	10	0.9%	-2.00 [-12.55, 8.55]
Kim 2007	122.9	7.11	14	124.2	10.74	12	1.2%	-1.30 [-8.43, 5.83]
Kim 2011	114	10.39	12	118	15.81	10	0.8%	-4.00 [-15.43, 7.43]
Kim 2020	127.6	2.3	24	133.2	4.1	24	1.7%	-5.60 [-7.48, -3.72]
Larsen 2016	100.1	6.9	51	103.1	7.7	44	1.7%	-3.00 [-5.96, -0.04]
Lee 2010(1)	119.34	15.19	20	121.12	16.03	9	0.8%	-1.78 [-14.19, 10.63]
Lee 2010(2)	116.53	10.99	16	121.12	16.03	9	0.8%	-4.59 [-16.37, 7.19]
Liqun 2014	106.5	12.5	76	109.8	9.3	97	1.6%	-3.30 [-6.66, 0.06]
Luo 2013(1)	100.72	6.21	43	112.21	8.26	34	1.6%	-11.49 [-14.83, -8.15]
Luo 2013(2)	97.5	6.22	52	107.89	9.11	38	1.6%	-10.39 [-13.74, -7.04]
Machado 2022	100.3	4.8	20	110.2	13.3	15	1.3%	-9.90 [-16.95, -2.85]
Meng 2022(2)	114	7	11	116	4	7	1.5%	-2.00 [-7.09, 3.09]
Meyer 2006	120	13.3	33	133	19.6	34	1.2%	-13.00 [-21.00, -5.00]
Murphy 2009	111.4	8	23	114.2	5.7	12	1.5%	-2.80 [-7.39, 1.79]
Park 2007	112	7.6	19	119.3	7.5	21	1.5%	-7.30 [-11.99, -2.61]
Racil(1) 2016(2)	115	5	16	118	5	7	1.5%	-3.00 [-7.44, 1.44]
Racil 2024(2)	119.33	4.7	12	119.1	4.7	6	1.5%	0.23 [-4.38, 4.84]
Saygın 2011	121.6	1.03	20	135.1	0.84	19	1.8%	-13.50 [-14.09, -12.91]
Seo 2019	123.06	11.07	26	123.91	15.95	44	1.3%	-0.85 [-7.20, 5.50]
Shalitin 2009	111	17.8	55	112	18.54	55	1.3%	-1.00 [-7.79, 5.79]
Shu 2023(2)	113.2	6.3	15	117.1	5	8	1.5%	-3.90 [-8.61, 0.81]
Son 2017	123.7	9.53	20	130.6	6.22	20	1.5%	-6.90 [-11.89, -1.91]
Woo 2012	103.05	7.75	10	116.3	7.18	10	1.3%	-13.25 [-19.80, -6.70]
Yu 2020	102.7	8.1	99	102.1	8.9	72	1.7%	0.60 [-2.00, 3.20]
Yuanyuan 2019(1)	105.47	7.21	10	106.57	7.62	3	1.0%	-1.10 [-10.81, 8.61]
Yuanyuan 2019(2)	99.01	7.24	10	106.57	7.62	3	1.0%	-7.56 [-17.28, 2.16]
Zhaogang 2021	98.24	9.64	49	101.24	10.04	49	1.6%	-3.00 [-6.90, 0.90]
**Subtotal (95% CI)**			**1318**			**1134**	**63.2%**	**-4.79 [-6.57, -3.00]**
Heterogeneity: Tau² = 29.03; Chi² = 663.33, df = 47 (P < 0.00001); I² = 93%								
Test for overall effect: Z = 5.26 (P < 0.00001)								
**2.2.2 High intensity**								
Abassi 2022(1)	122	2.3	13	125	3.8	6	1.6%	-3.00 [-6.29, 0.29]
Bezerra 2023	111.15	11.3	20	111.26	6.41	21	1.4%	-0.11 [-5.77, 5.55]
Boer 2014(1)	113	8	17	119	10	7	1.1%	-6.00 [-14.33, 2.33]
Chuensiri 2018(1)	125	13.27	11	121	13.27	5	0.7%	4.00 [-10.03, 18.03]
Chuensiri 2018(2)	116	11.62	15	121	13.27	6	0.8%	-5.00 [-17.14, 7.14]
Cvetković 2018(2)	116.5	14.15	11	124.85	11.58	7	0.8%	-8.35 [-20.33, 3.63]
Espinoza-Silva 2019(1)	103.06	13.53	69	102.6	10.14	30	1.5%	0.46 [-4.37, 5.29]
Espinoza-Silva 2019(2)	103.03	8.49	141	102.91	8.9	34	1.6%	0.12 [-3.18, 3.42]
Faria 2020(2)	114.5	8.29	26	117.3	8.29	13	1.4%	-2.80 [-8.32, 2.72]
Gonzalez-Galvez 2024(1)	123	11.4	9	126.5	13.16	6	0.7%	-3.50 [-16.40, 9.40]
Gonzalez-Galvez 2024(2)	116.3	11.28	11	126.5	13.16	6	0.8%	-10.20 [-22.66, 2.26]
Hongling 2012	113	5	30	116	6	30	1.7%	-3.00 [-5.79, -0.21]
Horner 2015(2)	110.89	8.04	23	116.63	10.23	8	1.2%	-5.74 [-13.55, 2.07]
Meng 2022(1)	110	5	12	116	4	6	1.5%	-6.00 [-10.27, -1.73]
Plavsic 2020	110.3	8.5	22	114.4	6.8	22	1.5%	-4.10 [-8.65, 0.45]
Racil(1) 2016(1)	115	5	17	118	5	7	1.5%	-3.00 [-7.40, 1.40]
Racil 2024(1)	119.15	5	12	119.1	4.7	5	1.5%	0.05 [-4.95, 5.05]
Salus(1) 2022	125.9	8.98	14	120.7	16.46	14	1.0%	5.20 [-4.62, 15.02]
Savoye 2007	121	11	75	120.4	12.54	44	1.5%	0.60 [-3.86, 5.06]
Savoye 2011	121.7	10.24	75	121.7	10.82	44	1.6%	0.00 [-3.95, 3.95]
Shu 2023(1)	111.9	4.7	16	117.1	5	8	1.6%	-5.20 [-9.36, -1.04]
Sigal 2014(1)	111	8.66	75	110	8.72	25	1.6%	1.00 [-2.94, 4.94]
Sigal 2014(2)	112	8.83	78	110	8.72	25	1.6%	2.00 [-1.94, 5.94]
Sigal 2014(3)	110	8.66	75	110	8.72	26	1.6%	0.00 [-3.88, 3.88]
Tjonna 2009	119.4	7.16	20	122.5	7.04	22	1.5%	-3.10 [-7.40, 1.20]
Vasconcellos 2016	123	13	10	129	6	10	1.1%	-6.00 [-14.87, 2.87]
Wong 2008	113.8	7.1	12	117	6.2	12	1.4%	-3.20 [-8.53, 2.13]
Yuanyuan 2019(3)	97.65	6.62	9	106.57	7.62	4	1.1%	-8.92 [-17.55, -0.29]
**Subtotal (95% CI)**			**918**			**453**	**36.8%**	**-1.96 [-3.01, -0.92]**
Heterogeneity: Tau² = 1.17; Chi² = 31.96, df = 27 (P = 0.23); I² = 16%								
Test for overall effect: Z = 3.69 (P = 0.0002)								
**Total (95% CI)**			**2236**			**1587**	**100.0%**	**-3.90 [-5.34, -2.46]**
Heterogeneity: Tau² = 30.25; Chi² = 876.13, df = 75 (P < 0.00001); I² = 91%								
Test for overall effect: Z = 5.31 (P < 0.00001)								
Test for subgroup differences: Chi² = 7.17, df = 1 (P = 0.007), I² = 86.1%								

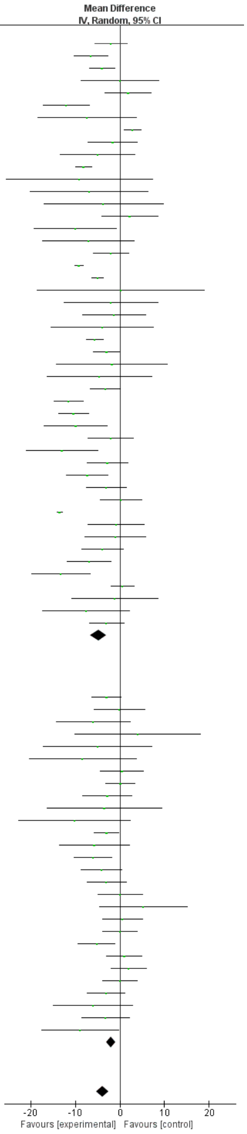

Figure S9.3. Forest plot of subgroup analyses by duration per session

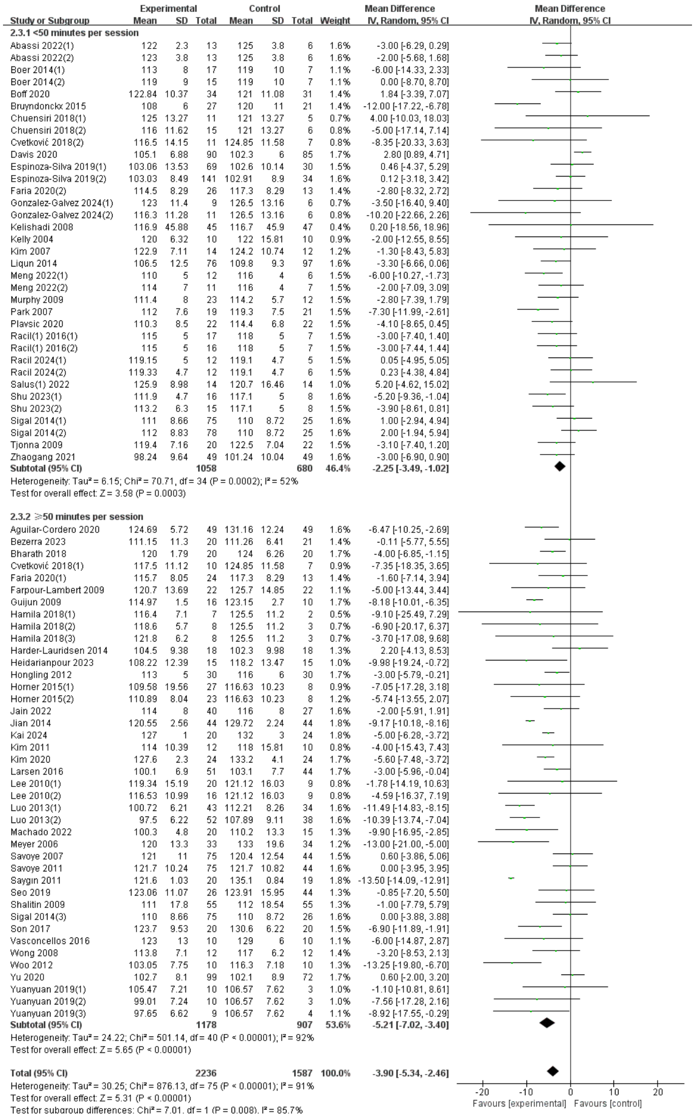

Figure S9.4. Forest plot of subgroup analyses by exercise frequency

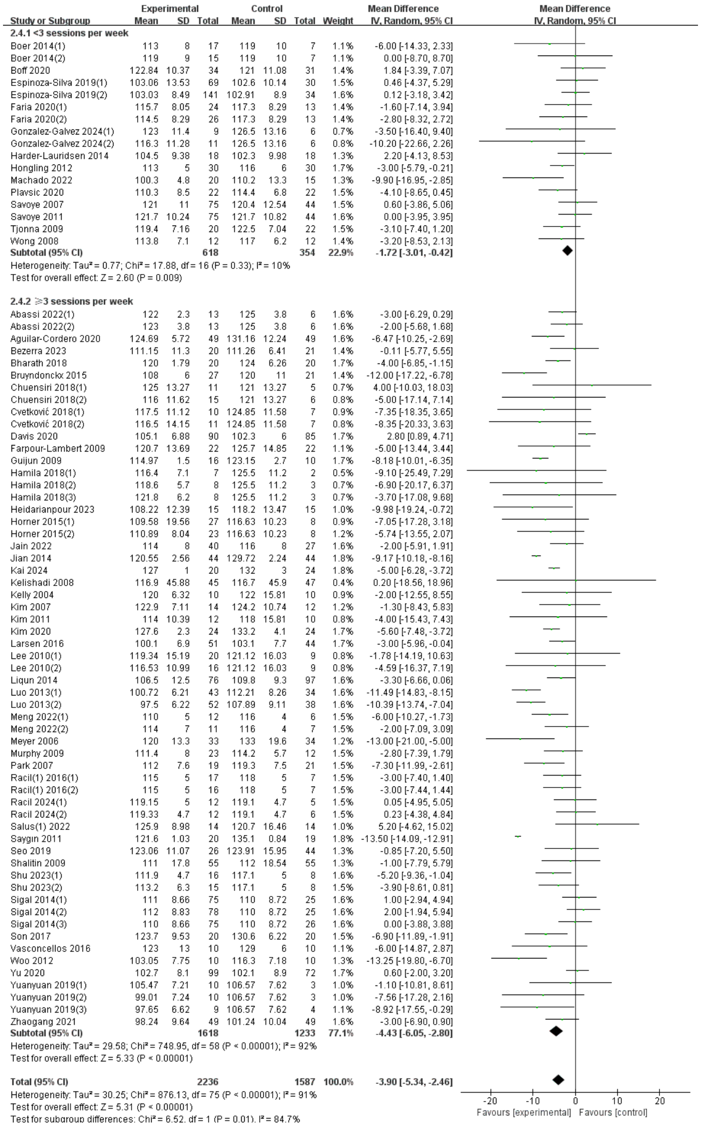

Figure S9.5. Forest plot of subgroup analyses by weekly exercise duration

Study or Subgroup	Experimental Mean	Experimental SD	Experimental Total	Control Mean	Control SD	Control Total	Weight	Mean Difference IV, Random, 95% CI
**2.5.1 <150 minutes per week**								
Abassi 2022(1)	122	2.3	13	125	3.8	6	1.6%	-3.00 [-6.29, 0.29]
Abassi 2022(2)	123	3.8	13	125	3.8	6	1.6%	-2.00 [-5.68, 1.68]
Boer 2014(1)	113	8	17	119	10	7	1.1%	-6.00 [-14.33, 2.33]
Boer 2014(2)	119	9	15	119	10	7	1.1%	0.00 [-8.70, 8.70]
Boff 2020	122.84	10.37	34	121	11.08	31	1.4%	1.84 [-3.39, 7.07]
Bruyndonckx 2015	108	6	27	120	11	21	1.4%	-12.00 [-17.22, -6.78]
Chuensiri 2018(1)	125	13.27	11	121	13.27	5	0.7%	4.00 [-10.03, 18.03]
Chuensiri 2018(2)	116	11.62	15	121	13.27	6	0.8%	-5.00 [-17.14, 7.14]
Cvetković 2018(2)	116.5	14.15	11	124.85	11.58	7	0.8%	-8.35 [-20.33, 3.63]
Espinoza-Silva 2019(1)	103.06	13.53	69	102.6	10.14	30	1.5%	0.46 [-4.37, 5.29]
Espinoza-Silva 2019(2)	103.03	8.49	141	102.91	8.9	34	1.6%	0.12 [-3.18, 3.42]
Faria 2020(1)	115.7	8.05	24	117.3	8.29	13	1.4%	-1.60 [-7.14, 3.94]
Faria 2020(2)	114.5	8.29	26	117.3	8.29	13	1.4%	-2.80 [-8.32, 2.72]
Gonzalez-Galvez 2024(1)	123	11.4	9	126.5	13.16	6	0.7%	-3.50 [-16.40, 9.40]
Gonzalez-Galvez 2024(2)	116.3	11.28	11	126.5	13.16	6	0.8%	-10.20 [-22.66, 2.26]
Harder-Lauridsen 2014	104.5	9.38	18	102.3	9.98	18	1.3%	2.20 [-4.13, 8.53]
Hongling 2012	113	5	30	116	6	30	1.7%	-3.00 [-5.79, -0.21]
Liqun 2014	106.5	12.5	76	109.8	9.3	97	1.6%	-3.30 [-6.66, 0.06]
Machado 2022	100.3	4.8	20	110.2	13.3	15	1.3%	-9.90 [-16.95, -2.85]
Meng 2022(1)	110	5	12	116	4	6	1.5%	-6.00 [-10.27, -1.73]
Meng 2022(2)	114	7	11	116	4	7	1.5%	-2.00 [-7.09, 3.09]
Murphy 2009	111.4	8	23	114.2	5.7	12	1.5%	-2.80 [-7.39, 1.79]
Plavsic 2020	110.3	8.5	22	114.4	6.8	22	1.5%	-4.10 [-8.65, 0.45]
Racil(1) 2016(1)	115	5	17	118	5	7	1.5%	-3.00 [-7.40, 1.40]
Racil(1) 2016(2)	115	5	16	118	5	7	1.5%	-3.00 [-7.44, 1.44]
Racil 2024(1)	119.15	5	12	119.1	4.7	5	1.5%	0.05 [-4.95, 5.05]
Racil 2024(2)	119.33	4.7	12	119.1	4.7	6	1.5%	0.23 [-4.38, 4.84]
Salus(1) 2022	125.9	8.98	14	120.7	16.46	14	1.0%	5.20 [-4.62, 15.02]
Savoye 2007	121	11	75	120.4	12.54	44	1.5%	0.60 [-3.86, 5.06]
Savoye 2011	121.7	10.24	75	121.7	10.82	44	1.6%	0.00 [-3.95, 3.95]
Shu 2023(1)	111.9	4.7	16	117.1	5	8	1.6%	-5.20 [-9.36, -1.04]
Shu 2023(2)	113.2	6.3	15	117.1	5	8	1.5%	-3.90 [-8.61, 0.81]
Sigal 2014(1)	111	8.66	75	110	8.72	25	1.6%	1.00 [-2.94, 4.94]
Sigal 2014(2)	112	8.83	78	110	8.72	25	1.6%	2.00 [-1.94, 5.94]
Tjonna 2009	119.4	7.16	20	122.5	7.04	22	1.5%	-3.10 [-7.40, 1.20]
Wong 2008	113.8	7.1	12	117	6.2	12	1.4%	-3.20 [-8.53, 2.13]
**Subtotal (95% CI)**			**1085**			**632**	**49.2%**	**-2.24 [-3.25, -1.23]**
Heterogeneity: Tau² = 2.82; Chi² = 51.48, df = 35 (P = 0.04); I² = 32%								
Test for overall effect: Z = 4.36 (P < 0.0001)								
**2.5.2 ≥150 minutes per week**								
Aguilar-Cordero 2020	124.69	5.72	49	131.16	12.24	49	1.6%	-6.47 [-10.25, -2.69]
Bezerra 2023	111.15	11.3	20	111.26	6.41	21	1.4%	-0.11 [-5.77, 5.55]
Bharath 2018	120	1.79	20	124	6.26	20	1.7%	-4.00 [-6.85, -1.15]
Cvetković 2018(1)	117.5	11.12	10	124.85	11.58	7	0.9%	-7.35 [-18.35, 3.65]
Davis 2020	105.1	6.88	90	102.3	6	85	1.7%	2.80 [0.89, 4.71]
Farpour-Lambert 2009	120.7	13.69	22	125.7	14.85	22	1.1%	-5.00 [-13.44, 3.44]
Guijun 2009	114.97	1.5	16	123.15	2.7	10	1.7%	-8.18 [-10.01, -6.35]
Hamila 2018(1)	116.4	7.1	7	125.5	11.2	2	0.5%	-9.10 [-25.49, 7.29]
Hamila 2018(2)	118.6	5.7	8	125.5	11.2	3	0.7%	-6.90 [-20.17, 6.37]
Hamila 2018(3)	121.8	6.2	8	125.5	11.2	3	0.7%	-3.70 [-17.08, 9.68]
Heidarianpour 2023	108.22	12.39	15	118.2	13.47	15	1.0%	-9.98 [-19.24, -0.72]
Horner 2015(1)	109.58	19.56	27	116.63	10.23	8	0.9%	-7.05 [-17.28, 3.18]
Horner 2015(2)	110.89	8.04	23	116.63	10.23	8	1.2%	-5.74 [-13.55, 2.07]
Jain 2022	114	8	40	116	8	27	1.6%	-2.00 [-5.91, 1.91]
Jian 2014	120.55	2.56	44	129.72	2.24	44	1.8%	-9.17 [-10.18, -8.16]
Kai 2024	127	1	20	132	3	24	1.8%	-5.00 [-6.28, -3.72]
Kelishadi 2008	116.9	45.88	45	116.7	45.9	47	0.4%	0.20 [-18.56, 18.96]
Kelly 2004	120	6.32	10	122	15.81	10	0.9%	-2.00 [-12.55, 8.55]
Kim 2007	122.9	7.11	14	124.2	10.74	12	1.2%	-1.30 [-8.43, 5.83]
Kim 2011	114	10.39	12	118	15.81	10	0.8%	-4.00 [-15.43, 7.43]
Kim 2020	127.6	2.3	24	133.2	4.1	24	1.7%	-5.60 [-7.48, -3.72]
Larsen 2016	100.1	6.9	51	103.1	7.7	44	1.7%	-3.00 [-5.96, -0.04]
Lee 2010(1)	119.34	15.19	20	121.12	16.03	9	0.8%	-1.78 [-14.19, 10.63]
Lee 2010(2)	116.53	10.99	16	121.12	16.03	9	0.8%	-4.59 [-16.37, 7.19]
Luo 2013(1)	100.72	6.21	43	112.21	8.26	34	1.6%	-11.49 [-14.83, -8.15]
Luo 2013(2)	97.5	6.22	52	107.89	9.11	38	1.6%	-10.39 [-13.74, -7.04]
Meyer 2006	120	13.3	33	133	19.6	34	1.2%	-13.00 [-21.00, -5.00]
Park 2007	112	7.6	19	119.3	7.5	21	1.5%	-7.30 [-11.99, -2.61]
Saygın 2011	121.6	1.03	20	135.1	0.84	19	1.8%	-13.50 [-14.09, -12.91]
Seo 2019	123.06	11.07	26	123.91	15.95	44	1.3%	-0.85 [-7.20, 5.50]
Shalitin 2009	111	17.8	55	112	18.54	55	1.3%	-1.00 [-7.79, 5.79]
Sigal 2014(3)	110	8.66	75	110	8.72	26	1.6%	0.00 [-3.88, 3.88]
Son 2017	123.7	9.53	20	130.6	6.22	20	1.5%	-6.90 [-11.89, -1.91]
Vasconcellos 2016	123	13	10	129	6	10	1.1%	-6.00 [-14.87, 2.87]
Woo 2012	103.05	7.75	10	116.3	7.18	10	1.3%	-13.25 [-19.80, -6.70]
Yu 2020	102.7	8.1	99	102.1	8.9	72	1.7%	0.60 [-2.00, 3.20]
Yuanyuan 2019(1)	105.47	7.21	10	106.57	7.62	3	1.0%	-1.10 [-10.81, 8.61]
Yuanyuan 2019(2)	99.01	7.24	10	106.57	7.62	3	1.0%	-7.56 [-17.28, 2.16]
Yuanyuan 2019(3)	97.65	6.62	9	106.57	7.62	4	1.1%	-8.92 [-17.55, -0.29]
Zhaogang 2021	98.24	9.64	49	101.24	10.04	49	1.6%	-3.00 [-6.90, 0.90]
**Subtotal (95% CI)**			**1151**			**955**	**50.8%**	**-5.38 [-7.35, -3.40]**
Heterogeneity: Tau² = 28.47; Chi² = 588.34, df = 39 (P < 0.00001); I² = 93%								
Test for overall effect: Z = 5.34 (P < 0.00001)								
**Total (95% CI)**			**2236**			**1587**	**100.0%**	**-3.90 [-5.34, -2.46]**
Heterogeneity: Tau² = 30.25; Chi² = 876.13, df = 75 (P < 0.00001); I² = 91%								
Test for overall effect: Z = 5.31 (P < 0.00001)								
Test for subgroup differences: Chi² = 7.67, df = 1 (P = 0.006), I² = 87.0%								

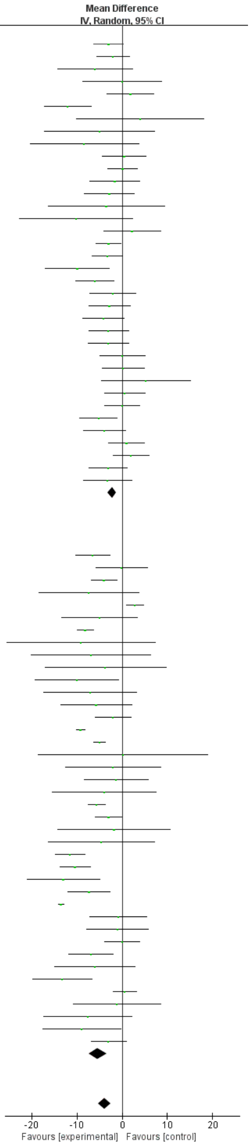

Figure S9.6. Forest plot of subgroup analyses by intervention duration

Study or Subgroup	Experimental Mean	Experimental SD	Experimental Total	Control Mean	Control SD	Control Total	Weight	Mean Difference IV, Random, 95% CI
**2.6.1 <12 weeks (total intervention duration)**								
Bezerra 2023	111.15	11.3	20	111.26	6.41	21	1.4%	-0.11 [-5.77, 5.55]
Gonzalez-Galvez 2024(1)	123	11.4	9	126.5	13.16	6	0.7%	-3.50 [-16.40, 9.40]
Gonzalez-Galvez 2024(2)	116.3	11.28	11	126.5	13.16	6	0.8%	-10.20 [-22.66, 2.26]
Hamila 2018(1)	116.4	7.1	7	125.5	11.2	2	0.5%	-9.10 [-25.49, 7.29]
Hamila 2018(2)	118.6	5.7	8	125.5	11.2	3	0.7%	-6.90 [-20.17, 6.37]
Hamila 2018(3)	121.8	6.2	8	125.5	11.2	3	0.7%	-3.70 [-17.08, 9.68]
Hongling 2012	113	5	30	116	6	30	1.7%	-3.00 [-5.79, -0.21]
Kelly 2004	120	6.32	10	122	15.81	10	0.9%	-2.00 [-12.55, 8.55]
Kim 2007	122.9	7.11	14	124.2	10.74	12	1.2%	-1.30 [-8.43, 5.83]
Larsen 2016	100.1	6.9	51	103.1	7.7	44	1.7%	-3.00 [-5.96, -0.04]
Lee 2010(1)	119.34	15.19	20	121.12	16.03	9	0.8%	-1.78 [-14.19, 10.63]
Lee 2010(2)	116.53	10.99	16	121.12	16.03	9	0.8%	-4.59 [-16.37, 7.19]
Luo 2013(1)	100.72	6.21	43	112.21	8.26	34	1.6%	-11.49 [-14.83, -8.15]
Luo 2013(2)	97.5	6.22	52	107.89	9.11	38	1.6%	-10.39 [-13.74, -7.04]
Racil 2024(1)	119.15	5	12	119.1	4.7	5	1.5%	0.05 [-4.95, 5.05]
Racil 2024(2)	119.33	4.7	12	119.1	4.7	6	1.5%	0.23 [-4.38, 4.84]
**Subtotal (95% CI)**			**323**			**238**	**18.2%**	**-4.26 [-6.84, -1.67]**
Heterogeneity: Tau² = 13.63; Chi² = 42.51, df = 15 (P = 0.0002); I² = 65%								
Test for overall effect: Z = 3.23 (P = 0.001)								
**2.6.2 ≥12 weeks (total intervention duration)**								
Abassi 2022(1)	122	2.3	13	125	3.8	6	1.6%	-3.00 [-6.29, 0.29]
Abassi 2022(2)	123	3.8	13	125	3.8	6	1.6%	-2.00 [-5.68, 1.68]
Aguilar-Cordero 2020	124.69	5.72	49	131.16	12.24	49	1.6%	-6.47 [-10.25, -2.69]
Bharath 2018	120	1.79	20	124	6.26	20	1.7%	-4.00 [-6.85, -1.15]
Boer 2014(1)	113	8	17	119	10	7	1.1%	-6.00 [-14.33, 2.33]
Boer 2014(2)	119	9	15	119	10	7	1.1%	0.00 [-8.70, 8.70]
Boff 2020	122.84	10.37	34	121	11.08	31	1.4%	1.84 [-3.39, 7.07]
Bruyndonckx 2015	108	6	27	120	11	21	1.4%	-12.00 [-17.22, -6.78]
Chuensiri 2018(1)	125	13.27	11	121	13.27	5	0.7%	4.00 [-10.03, 18.03]
Chuensiri 2018(2)	116	11.62	15	121	13.27	6	0.8%	-5.00 [-17.14, 7.14]
Cvetković 2018(1)	117.5	11.12	10	124.85	11.58	7	0.9%	-7.35 [-18.35, 3.65]
Cvetković 2018(2)	116.5	14.15	11	124.85	11.58	7	0.8%	-8.35 [-20.33, 3.63]
Davis 2020	105.1	6.88	90	102.3	6	85	1.7%	2.80 [0.89, 4.71]
Espinoza-Silva 2019(1)	103.06	13.53	69	102.6	10.14	30	1.5%	0.46 [-4.37, 5.29]
Espinoza-Silva 2019(2)	103.03	8.49	141	102.91	8.9	34	1.6%	0.12 [-3.18, 3.42]
Faria 2020(1)	115.7	8.05	24	117.3	8.29	13	1.4%	-1.60 [-7.14, 3.94]
Faria 2020(2)	114.5	8.29	26	117.3	8.29	13	1.4%	-2.80 [-8.32, 2.72]
Farpour-Lambert 2009	120.7	13.69	22	125.7	14.85	22	1.1%	-5.00 [-13.44, 3.44]
Guijun 2009	114.97	1.5	16	123.15	2.7	10	1.7%	-8.18 [-10.01, -6.35]
Harder-Lauridsen 2014	104.5	9.38	18	102.3	9.98	18	1.3%	2.20 [-4.13, 8.53]
Heidarianpour 2023	108.22	12.39	15	118.2	13.47	15	1.0%	-9.98 [-19.24, -0.72]
Horner 2015(1)	109.58	19.56	27	116.63	10.23	8	0.9%	-7.05 [-17.28, 3.18]
Horner 2015(2)	110.89	8.04	23	116.63	10.23	8	1.2%	-5.74 [-13.55, 2.07]
Jain 2022	114	8	40	116	8	27	1.6%	-2.00 [-5.91, 1.91]
Jian 2014	120.55	2.56	44	129.72	2.24	44	1.8%	-9.17 [-10.18, -8.16]
Kai 2024	127	1	20	132	3	24	1.8%	-5.00 [-6.28, -3.72]
Kelishadi 2008	116.9	45.88	45	116.7	45.9	47	0.4%	0.20 [-18.56, 18.96]
Kim 2011	114	10.39	12	118	15.81	10	0.8%	-4.00 [-15.43, 7.43]
Kim 2020	127.6	2.3	24	133.2	4.1	24	1.7%	-5.60 [-7.48, -3.72]
Liqun 2014	106.5	12.5	76	109.8	9.3	97	1.6%	-3.30 [-6.66, 0.06]
Machado 2022	100.3	4.8	20	110.2	13.3	15	1.3%	-9.90 [-16.95, -2.85]
Meng 2022(1)	110	5	12	116	4	6	1.5%	-6.00 [-10.27, -1.73]
Meng 2022(2)	114	7	11	116	4	7	1.5%	-2.00 [-7.09, 3.09]
Meyer 2006	120	13.3	33	133	19.6	34	1.2%	-13.00 [-21.00, -5.00]
Murphy 2009	111.4	8	23	114.2	5.7	12	1.5%	-2.80 [-7.39, 1.79]
Park 2007	112	7.6	19	119.3	7.5	21	1.5%	-7.30 [-11.99, -2.61]
Plavsic 2020	110.3	8.5	22	114.4	6.8	22	1.5%	-4.10 [-8.65, 0.45]
Racil(1) 2016(1)	115	5	17	118	5	7	1.5%	-3.00 [-7.40, 1.40]
Racil(1) 2016(2)	115	5	16	118	5	7	1.5%	-3.00 [-7.44, 1.44]
Salus(1) 2022	125.9	8.98	14	120.7	16.46	14	1.0%	5.20 [-4.62, 15.02]
Savoye 2007	121	11	75	120.4	12.54	44	1.5%	0.60 [-3.86, 5.06]
Savoye 2011	121.7	10.24	75	121.7	10.82	44	1.6%	0.00 [-3.95, 3.95]
Saygın 2011	121.6	1.03	20	135.1	0.84	19	1.8%	-13.50 [-14.09, -12.91]
Seo 2019	123.06	11.07	26	123.91	15.95	44	1.3%	-0.85 [-7.20, 5.50]
Shalitin 2009	111	17.8	55	112	18.54	55	1.3%	-1.00 [-7.79, 5.79]
Shu 2023(1)	111.9	4.7	16	117.1	5	8	1.6%	-5.20 [-9.36, -1.04]
Shu 2023(2)	113.2	6.3	15	117.1	5	8	1.5%	-3.90 [-8.61, 0.81]
Sigal 2014(1)	111	8.66	75	110	8.72	25	1.6%	1.00 [-2.94, 4.94]
Sigal 2014(2)	112	8.83	78	110	8.72	25	1.6%	2.00 [-1.94, 5.94]
Sigal 2014(3)	110	8.66	75	110	8.72	26	1.6%	0.00 [-3.88, 3.88]
Son 2017	123.7	9.53	20	130.6	6.22	20	1.5%	-6.90 [-11.89, -1.91]
Tjonna 2009	119.4	7.16	20	122.5	7.04	22	1.5%	-3.10 [-7.40, 1.20]
Vasconcellos 2016	123	13	10	129	6	10	1.1%	-6.00 [-14.87, 2.87]
Wong 2008	113.8	7.1	12	117	6.2	12	1.4%	-3.20 [-8.53, 2.13]
Woo 2012	103.05	7.75	10	116.3	7.18	10	1.3%	-13.25 [-19.80, -6.70]
Yu 2020	102.7	8.1	99	102.1	8.9	72	1.7%	0.60 [-2.00, 3.20]
Yuanyuan 2019(1)	105.47	7.21	10	106.57	7.62	3	1.0%	-1.10 [-10.81, 8.61]
Yuanyuan 2019(2)	99.01	7.24	10	106.57	7.62	3	1.0%	-7.56 [-17.28, 2.16]
Yuanyuan 2019(3)	97.65	6.62	9	106.57	7.62	4	1.1%	-8.92 [-17.55, -0.29]
Zhaogang 2021	98.24	9.64	49	101.24	10.04	49	1.6%	-3.00 [-6.90, 0.90]
**Subtotal (95% CI)**			**1913**			**1349**	**81.8%**	**-3.83 [-5.45, -2.21]**
Heterogeneity: Tau² = 31.64; Chi² = 811.39, df = 59 (P < 0.00001); I² = 93%								
Test for overall effect: Z = 4.63 (P < 0.00001)								
**Total (95% CI)**			**2236**			**1587**	**100.0%**	**-3.90 [-5.34, -2.46]**
Heterogeneity: Tau² = 30.25; Chi² = 876.13, df = 75 (P < 0.00001); I² = 91%								
Test for overall effect: Z = 5.31 (P < 0.00001)								
Test for subgroup differences: Chi² = 0.07, df = 1 (P = 0.78), I² = 0%								

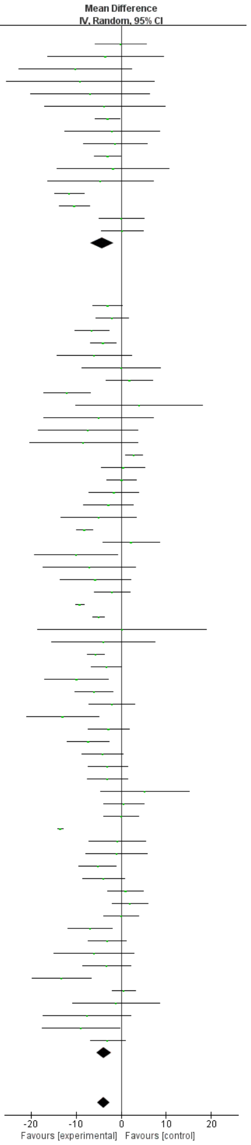

Figure S9.7. Forest plot of subgroup analyses by region

Study or Subgroup	Experimental Mean	Experimental SD	Experimental Total	Control Mean	Control SD	Control Total	Weight	Mean Difference IV, Random, 95% CI
**2.7.1 Asia**								
Chuensiri 2018(1)	125	13.27	11	121	13.27	5	0.7%	4.00 [-10.03, 18.03]
Chuensiri 2018(2)	116	11.62	15	121	13.27	6	0.8%	-5.00 [-17.14, 7.14]
Guijun 2009	114.97	1.5	16	123.15	2.7	10	1.7%	-8.18 [-10.01, -6.35]
Heidarianpour 2023	108.22	12.39	15	118.2	13.47	15	1.0%	-9.98 [-19.24, -0.72]
Hongling 2012	113	5	30	116	6	30	1.7%	-3.00 [-5.79, -0.21]
Jain 2022	114	8	40	116	8	27	1.6%	-2.00 [-5.91, 1.91]
Jian 2014	120.55	2.56	44	129.72	2.24	44	1.8%	-9.17 [-10.18, -8.16]
Kai 2024	127	1	20	132	3	24	1.8%	-5.00 [-6.28, -3.72]
Kelishadi 2008	116.9	45.88	45	116.7	45.9	47	0.4%	0.20 [-18.56, 18.96]
Kim 2007	122.9	7.11	14	124.2	10.74	12	1.2%	-1.30 [-8.43, 5.83]
Kim 2011	114	10.39	12	118	15.81	10	0.8%	-4.00 [-15.43, 7.43]
Lee 2010(1)	119.34	15.19	20	121.12	16.03	9	0.8%	-1.78 [-14.19, 10.63]
Lee 2010(2)	116.53	10.99	16	121.12	16.03	9	0.8%	-4.59 [-16.37, 7.19]
Liqun 2014	106.5	12.5	76	109.8	9.3	97	1.6%	-3.30 [-6.66, 0.06]
Luo 2013(1)	100.72	6.21	43	112.21	8.26	34	1.6%	-11.49 [-14.83, -8.15]
Luo 2013(2)	97.5	6.22	52	107.89	9.11	38	1.6%	-10.39 [-13.74, -7.04]
Meng 2022(1)	110	5	12	116	4	6	1.5%	-6.00 [-10.27, -1.73]
Meng 2022(2)	114	7	11	116	4	7	1.5%	-2.00 [-7.09, 3.09]
Park 2007	112	7.6	19	119.3	7.5	21	1.5%	-7.30 [-11.99, -2.61]
Saygın 2011	121.6	1.03	20	135.1	0.84	19	1.8%	-13.50 [-14.09, -12.91]
Seo 2019	123.06	11.07	26	123.91	15.95	44	1.3%	-0.85 [-7.20, 5.50]
Shalitin 2009	111	17.8	55	112	18.54	55	1.3%	-1.00 [-7.79, 5.79]
Shu 2023(1)	111.9	4.7	16	117.1	5	8	1.6%	-5.20 [-9.36, -1.04]
Shu 2023(2)	113.2	6.3	15	117.1	5	8	1.5%	-3.90 [-8.61, 0.81]
Son 2017	123.7	9.53	20	130.6	6.22	20	1.5%	-6.90 [-11.89, -1.91]
Wong 2008	113.8	7.1	12	117	6.2	12	1.4%	-3.20 [-8.53, 2.13]
Woo 2012	103.05	7.75	10	116.3	7.18	10	1.3%	-13.25 [-19.80, -6.70]
Yu 2020	102.7	8.1	99	102.1	8.9	72	1.7%	0.60 [-2.00, 3.20]
Yuanyuan 2019(1)	105.47	7.21	10	106.57	7.62	3	1.0%	-1.10 [-10.81, 8.61]
Yuanyuan 2019(2)	99.01	7.24	10	106.57	7.62	3	1.0%	-7.56 [-17.28, 2.16]
Yuanyuan 2019(3)	97.65	6.62	9	106.57	7.62	4	1.1%	-8.92 [-17.55, -0.29]
Zhaogang 2021	98.24	9.64	49	101.24	10.04	49	1.6%	-3.00 [-6.90, 0.90]
**Subtotal (95% CI)**			**862**			**758**	**42.5%**	**-5.42 [-7.39, -3.46]**
Heterogeneity: Tau^2 = 22.41; Chi^2 = 400.70, df = 31 (P < 0.00001); I^2 = 92%								
Test for overall effect: Z = 5.41 (P < 0.00001)								
**2.7.2 Europe**								
Aguilar-Cordero 2020	124.69	5.72	49	131.16	12.24	49	1.6%	-6.47 [-10.25, -2.69]
Boer 2014(1)	113	8	17	119	10	7	1.1%	-6.00 [-14.33, 2.33]
Boer 2014(2)	119	9	15	119	10	7	1.1%	0.00 [-8.70, 8.70]
Bruyndonckx 2015	108	6	27	120	11	21	1.4%	-12.00 [-17.22, -6.78]
Cvetković 2018(1)	117.5	11.12	10	124.85	11.58	7	0.9%	-7.35 [-18.35, 3.65]
Cvetković 2018(2)	116.5	14.15	11	124.85	11.58	7	0.8%	-8.35 [-20.33, 3.63]
Farpour-Lambert 2009	120.7	13.69	22	125.7	14.85	22	1.1%	-5.00 [-13.44, 3.44]
Gonzalez-Galvez 2024(1)	123	11.4	9	126.5	13.16	6	0.7%	-3.50 [-16.40, 9.40]
Gonzalez-Galvez 2024(2)	116.3	11.28	11	126.5	13.16	6	0.8%	-10.20 [-22.66, 2.26]
Harder-Lauridsen 2014	104.5	9.38	18	102.3	9.98	18	1.3%	2.20 [-4.13, 8.53]
Larsen 2016	100.1	6.9	51	103.1	7.7	44	1.7%	-3.00 [-5.96, -0.04]
Meyer 2006	120	13.3	33	133	19.6	34	1.2%	-13.00 [-21.00, -5.00]
Plavsic 2020	110.3	8.5	22	114.4	6.8	22	1.5%	-4.10 [-8.65, 0.45]
Salus(1) 2022	125.9	8.98	14	120.7	16.46	14	1.0%	5.20 [-4.62, 15.02]
Tjonna 2009	119.4	7.16	20	122.5	7.04	22	1.5%	-3.10 [-7.40, 1.20]
**Subtotal (95% CI)**			**329**			**286**	**17.7%**	**-4.82 [-7.11, -2.54]**
Heterogeneity: Tau^2 = 7.72; Chi^2 = 25.40, df = 14 (P = 0.03); I^2 = 45%								
Test for overall effect: Z = 4.15 (P < 0.0001)								
**2.7.3 Africa**								
Abassi 2022(1)	122	2.3	13	125	3.8	6	1.6%	-3.00 [-6.29, 0.29]
Abassi 2022(2)	123	3.8	13	125	3.8	6	1.6%	-2.00 [-5.68, 1.68]
Hamila 2018(1)	116.4	7.1	7	125.5	11.2	2	0.5%	-9.10 [-25.49, 7.29]
Hamila 2018(2)	118.6	5.7	8	125.5	11.2	3	0.7%	-6.90 [-20.17, 6.37]
Hamila 2018(3)	121.8	6.2	8	125.5	11.2	3	0.7%	-3.70 [-17.08, 9.68]
Racil(1) 2016(1)	115	5	17	118	5	7	1.5%	-3.00 [-7.40, 1.40]
Racil(1) 2016(2)	115	5	16	118	5	7	1.5%	-3.00 [-7.44, 1.44]
Racil 2024(1)	119.15	5	12	119.1	4.7	5	1.5%	0.05 [-4.95, 5.05]
Racil 2024(2)	119.33	4.7	12	119.1	4.7	6	1.5%	0.23 [-4.38, 4.84]
**Subtotal (95% CI)**			**106**			**45**	**11.2%**	**-2.19 [-3.83, -0.55]**
Heterogeneity: Tau^2 = 0.00; Chi^2 = 3.55, df = 8 (P = 0.90); I^2 = 0%								
Test for overall effect: Z = 2.62 (P = 0.009)								
**2.7.4 North America**								
Bharath 2018	120	1.79	20	124	6.26	20	1.7%	-4.00 [-6.85, -1.15]
Davis 2020	105.1	6.88	90	102.3	6	85	1.7%	2.80 [0.89, 4.71]
Horner 2015(1)	109.58	19.56	27	116.63	10.23	8	0.9%	-7.05 [-17.28, 3.18]
Horner 2015(2)	110.89	8.04	23	116.63	10.23	8	1.2%	-5.74 [-13.55, 2.07]
Kelly 2004	120	6.32	10	122	15.81	10	0.9%	-2.00 [-12.55, 8.55]
Kim 2020	127.6	2.3	24	133.2	4.1	24	1.7%	-5.60 [-7.48, -3.72]
Murphy 2009	111.4	8	23	114.2	5.7	12	1.5%	-2.80 [-7.39, 1.79]
Savoye 2007	121	11	75	120.4	12.54	44	1.5%	0.60 [-3.86, 5.06]
Savoye 2011	121.7	10.24	75	121.7	10.82	44	1.6%	0.00 [-3.95, 3.95]
Sigal 2014(1)	111	8.66	75	110	8.72	25	1.6%	1.00 [-2.94, 4.94]
Sigal 2014(2)	112	8.83	78	110	8.72	25	1.6%	2.00 [-1.94, 5.94]
Sigal 2014(3)	110	8.66	75	110	8.72	26	1.6%	0.00 [-3.88, 3.88]
**Subtotal (95% CI)**			**595**			**331**	**17.5%**	**-1.28 [-3.61, 1.05]**
Heterogeneity: Tau^2 = 11.28; Chi^2 = 49.65, df = 11 (P < 0.00001); I^2 = 78%								
Test for overall effect: Z = 1.08 (P = 0.28)								
**2.7.5 South America**								
Bezerra 2023	111.15	11.3	20	111.26	6.41	21	1.4%	-0.11 [-5.77, 5.55]
Boff 2020	122.84	10.37	34	121	11.08	31	1.4%	1.84 [-3.39, 7.07]
Espinoza-Silva 2019(1)	103.06	13.53	69	102.6	10.14	30	1.5%	0.46 [-4.37, 5.29]
Espinoza-Silva 2019(2)	103.03	8.49	141	102.91	8.9	34	1.6%	0.12 [-3.18, 3.42]
Faria 2020(1)	115.7	8.05	24	117.3	8.29	13	1.4%	-1.60 [-7.14, 3.94]
Faria 2020(2)	114.5	8.29	26	117.3	8.29	13	1.4%	-2.80 [-8.32, 2.72]
Machado 2022	100.3	4.8	20	110.2	13.3	15	1.3%	-9.90 [-16.95, -2.85]
Vasconcellos 2016	123	13	10	129	6	10	1.1%	-6.00 [-14.87, 2.87]
**Subtotal (95% CI)**			**344**			**167**	**11.1%**	**-1.36 [-3.61, 0.88]**
Heterogeneity: Tau^2 = 2.92; Chi^2 = 9.80, df = 7 (P = 0.20); I^2 = 29%								
Test for overall effect: Z = 1.19 (P = 0.23)								
**2.7.6 Oceania**								
**Subtotal (95% CI)**			**0**			**0**		**Not estimable**
Heterogeneity: Not applicable								
Test for overall effect: Not applicable								
**Total (95% CI)**			**2236**			**1587**	**100.0%**	**-3.90 [-5.34, -2.46]**
Heterogeneity: Tau^2 = 30.25; Chi^2 = 876.13, df = 75 (P < 0.00001); I^2 = 91%								
Test for overall effect: Z = 5.31 (P < 0.00001)								
Test for subgroup differences: Chi^2 = 13.35, df = 4 (P = 0.010), I^2 = 70.0%								

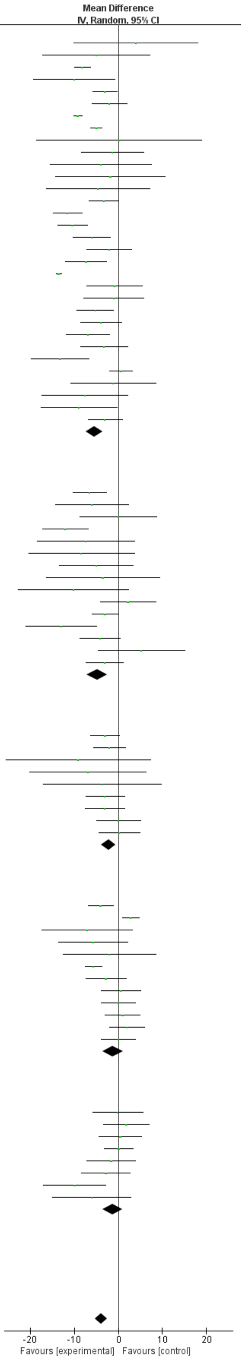

Figure S9.8. Forest plot of subgroup analyses by economic status

Study or Subgroup	Experimental Mean	Experimental SD	Experimental Total	Control Mean	Control SD	Control Total	Weight	Mean Difference IV, Random, 95% CI
**2.8.1 Lower-middle-income countries**								
Abassi 2022(1)	122	2.3	13	125	3.8	6	1.6%	-3.00 [-6.29, 0.29]
Abassi 2022(2)	123	3.8	13	125	3.8	6	1.6%	-2.00 [-5.68, 1.68]
Hamila 2018(1)	116.4	7.1	7	125.5	11.2	2	0.5%	-9.10 [-25.49, 7.29]
Hamila 2018(2)	118.6	5.7	8	125.5	11.2	3	0.7%	-6.90 [-20.17, 6.37]
Hamila 2018(3)	121.8	6.2	8	125.5	11.2	3	0.7%	-3.70 [-17.08, 9.68]
Jain 2022	114	8	40	116	8	27	1.6%	-2.00 [-5.91, 1.91]
Racil(1) 2016(1)	115	5	17	118	5	7	1.5%	-3.00 [-7.40, 1.40]
Racil(1) 2016(2)	115	5	16	118	5	7	1.5%	-3.00 [-7.44, 1.44]
Racil 2024(1)	119.15	5	12	119.1	4.7	5	1.5%	0.05 [-4.95, 5.05]
Racil 2024(2)	119.33	4.7	12	119.1	4.7	6	1.5%	0.23 [-4.38, 4.84]
**Subtotal (95% CI)**			**146**			**72**	**12.8%**	**-2.16 [-3.68, -0.65]**
Heterogeneity: Tau^2 = 0.00; Chi^2 = 3.56, df = 9 (P = 0.94); I^2 = 0%								
Test for overall effect: Z = 2.80 (P = 0.005)								
**2.8.2 Upper-middle-income countries**								
Bezerra 2023	111.15	11.3	20	111.26	6.41	21	1.4%	-0.11 [-5.77, 5.55]
Boff 2020	122.84	10.37	34	121	11.08	31	1.4%	1.84 [-3.39, 7.07]
Chuensiri 2018(1)	125	13.27	11	121	13.27	5	0.7%	4.00 [-10.03, 18.03]
Chuensiri 2018(2)	116	11.62	15	121	13.27	6	0.8%	-5.00 [-17.14, 7.14]
Cvetković 2018(1)	117.5	11.12	10	124.85	11.58	7	0.9%	-7.35 [-18.35, 3.65]
Cvetković 2018(2)	116.5	14.15	11	124.85	11.58	7	0.8%	-8.35 [-20.33, 3.63]
Faria 2020(1)	115.7	8.05	24	117.3	8.29	13	1.4%	-1.60 [-7.14, 3.94]
Faria 2020(2)	114.5	8.29	26	117.3	8.29	13	1.4%	-2.80 [-8.32, 2.72]
Guijun 2009	114.97	1.5	16	123.15	2.7	10	1.7%	-8.18 [-10.01, -6.35]
Heidarianpour 2023	108.22	12.39	15	118.2	13.47	15	1.0%	-9.98 [-19.24, -0.72]
Hongling 2012	113	5	30	116	6	30	1.7%	-3.00 [-5.79, -0.21]
Jian 2014	120.55	2.56	44	129.72	2.24	44	1.8%	-9.17 [-10.18, -8.16]
Kai 2024	127	1	20	132	3	24	1.8%	-5.00 [-6.28, -3.72]
Kelishadi 2008	116.9	45.88	45	116.7	45.9	47	0.4%	0.20 [-18.56, 18.96]
Liqun 2014	106.5	12.5	76	109.8	9.3	97	1.6%	-3.30 [-6.66, 0.06]
Luo 2013(1)	100.72	6.21	43	112.21	8.26	34	1.6%	-11.49 [-14.83, -8.15]
Luo 2013(2)	97.5	6.22	52	107.89	9.11	38	1.6%	-10.39 [-13.74, -7.04]
Machado 2022	100.3	4.8	20	110.2	13.3	15	1.3%	-9.90 [-16.95, -2.85]
Meng 2022(1)	110	5	12	116	4	6	1.5%	-6.00 [-10.27, -1.73]
Meng 2022(2)	114	7	11	116	4	7	1.5%	-2.00 [-7.09, 3.09]
Plavsic 2020	110.3	8.5	22	114.4	6.8	22	1.5%	-4.10 [-8.65, 0.45]
Saygın 2011	121.6	1.03	20	135.1	0.84	19	1.8%	-13.50 [-14.09, -12.91]
Shu 2023(1)	111.9	4.7	16	117.1	5	8	1.6%	-5.20 [-9.36, -1.04]
Shu 2023(2)	113.2	6.3	15	117.1	5	8	1.5%	-3.90 [-8.61, 0.81]
Vasconcellos 2016	123	13	10	129	6	10	1.1%	-6.00 [-14.87, 2.87]
Yu 2020	102.7	8.1	99	102.1	8.9	72	1.7%	0.60 [-2.00, 3.20]
Yuanyuan 2019(1)	105.47	7.21	10	106.57	7.62	3	1.0%	-1.10 [-10.81, 8.61]
Yuanyuan 2019(2)	99.01	7.24	10	106.57	7.62	3	1.0%	-7.56 [-17.28, 2.16]
Yuanyuan 2019(3)	97.65	6.62	9	106.57	7.62	4	1.1%	-8.92 [-17.55, -0.29]
Zhaogang 2021	98.24	9.64	49	101.24	10.04	49	1.6%	-3.00 [-6.90, 0.90]
**Subtotal (95% CI)**			**795**			**668**	**40.1%**	**-5.29 [-7.34, -3.24]**
Heterogeneity: Tau^2 = 23.26; Chi^2 = 404.98, df = 29 (P < 0.00001); I^2 = 93%								
Test for overall effect: Z = 5.05 (P < 0.00001)								
**2.8.3 High-income countries**								
Aguilar-Cordero 2020	124.69	5.72	49	131.16	12.24	49	1.6%	-6.47 [-10.25, -2.69]
Bharath 2018	120	1.79	20	124	6.26	20	1.7%	-4.00 [-6.85, -1.15]
Boer 2014(1)	113	8	17	119	10	7	1.1%	-6.00 [-14.33, 2.33]
Boer 2014(2)	119	9	15	119	10	7	1.1%	0.00 [-8.70, 8.70]
Bruyndonckx 2015	108	6	27	120	11	21	1.4%	-12.00 [-17.22, -6.78]
Davis 2020	105.1	6.88	90	102.3	6	85	1.7%	2.80 [0.89, 4.71]
Espinoza-Silva 2019(1)	103.06	13.53	69	102.6	10.14	30	1.5%	0.46 [-4.37, 5.29]
Espinoza-Silva 2019(2)	103.03	8.49	141	102.91	8.9	34	1.6%	0.12 [-3.18, 3.42]
Farpour-Lambert 2009	120.7	13.69	22	125.7	14.85	22	1.1%	-5.00 [-13.44, 3.44]
Gonzalez-Galvez 2024(1)	123	11.4	9	126.5	13.16	6	0.7%	-3.50 [-16.40, 9.40]
Gonzalez-Galvez 2024(2)	116.3	11.28	11	126.5	13.16	6	0.8%	-10.20 [-22.66, 2.26]
Harder-Lauridsen 2014	104.5	9.38	18	102.3	9.98	18	1.3%	2.20 [-4.13, 8.53]
Horner 2015(1)	109.58	19.56	27	116.63	10.23	8	0.9%	-7.05 [-17.28, 3.18]
Horner 2015(2)	110.89	8.04	23	116.63	10.23	8	1.2%	-5.74 [-13.55, 2.07]
Kelly 2004	120	6.32	10	122	15.81	10	0.9%	-2.00 [-12.55, 8.55]
Kim 2007	122.9	7.11	14	124.2	10.74	12	1.2%	-1.30 [-8.43, 5.83]
Kim 2011	114	10.39	12	118	15.81	10	0.8%	-4.00 [-15.43, 7.43]
Kim 2020	127.6	2.3	24	133.2	4.1	24	1.7%	-5.60 [-7.48, -3.72]
Larsen 2016	100.1	6.9	51	103.1	7.7	44	1.7%	-3.00 [-5.96, -0.04]
Lee 2010(1)	119.34	15.19	20	121.12	16.03	9	0.8%	-1.78 [-14.19, 10.63]
Lee 2010(2)	116.53	10.99	16	121.12	16.03	9	0.8%	-4.59 [-16.37, 7.19]
Meyer 2006	120	13.3	33	133	19.6	34	1.2%	-13.00 [-21.00, -5.00]
Murphy 2009	111.4	8	23	114.2	5.7	12	1.5%	-2.80 [-7.39, 1.79]
Park 2007	112	7.6	19	119.3	7.5	21	1.5%	-7.30 [-11.99, -2.61]
Salus(1) 2022	125.9	8.98	14	120.7	16.46	14	1.0%	5.20 [-4.62, 15.02]
Savoye 2007	121	11	75	120.4	12.54	44	1.5%	0.60 [-3.86, 5.06]
Savoye 2011	121.7	10.24	75	121.7	10.82	44	1.6%	0.00 [-3.95, 3.95]
Seo 2019	123.06	11.07	26	123.91	15.95	44	1.3%	-0.85 [-7.20, 5.50]
Shalitin 2009	111	17.8	55	112	18.54	55	1.3%	-1.00 [-7.79, 5.79]
Sigal 2014(1)	111	8.66	75	110	8.72	25	1.6%	1.00 [-2.94, 4.94]
Sigal 2014(2)	112	8.83	78	110	8.72	25	1.6%	2.00 [-1.94, 5.94]
Sigal 2014(3)	110	8.66	75	110	8.72	26	1.6%	0.00 [-3.88, 3.88]
Son 2017	123.7	9.53	20	130.6	6.22	20	1.5%	-6.90 [-11.89, -1.91]
Tjonna 2009	119.4	7.16	20	122.5	7.04	22	1.5%	-3.10 [-7.40, 1.20]
Wong 2008	113.8	7.1	12	117	6.2	12	1.4%	-3.20 [-8.53, 2.13]
Woo 2012	103.05	7.75	10	116.3	7.18	10	1.3%	-13.25 [-19.80, -6.70]
**Subtotal (95% CI)**			**1295**			**847**	**47.1%**	**-2.96 [-4.44, -1.47]**
Heterogeneity: Tau^2 = 11.33; Chi^2 = 108.52, df = 35 (P < 0.00001); I^2 = 68%								
Test for overall effect: Z = 3.90 (P < 0.0001)								
**Total (95% CI)**			**2236**			**1587**	**100.0%**	**-3.90 [-5.34, -2.46]**
Heterogeneity: Tau^2 = 30.25; Chi^2 = 876.13, df = 75 (P < 0.00001); I^2 = 91%								
Test for overall effect: Z = 5.31 (P < 0.00001)								
Test for subgroup differences: Chi^2 = 5.87, df = 2 (P = 0.05), I^2 = 66.0%								

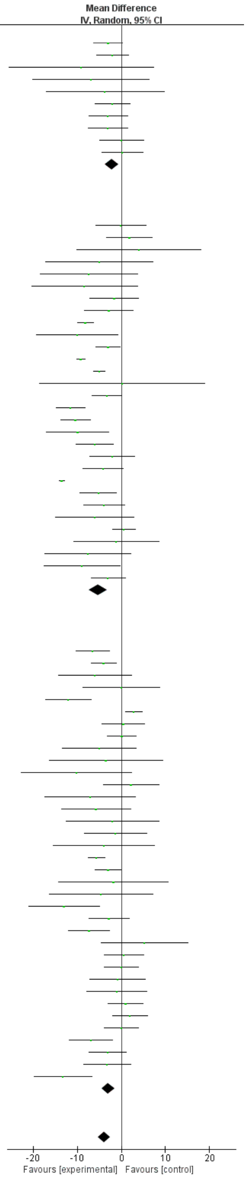

Figure S9.9. Forest plot of subgroup analyses by weight status

Study or Subgroup	Experimental Mean	Experimental SD	Experimental Total	Control Mean	Control SD	Control Total	Weight	Mean Difference IV, Random, 95% CI
**2.9.1 Overweight**								
Bezerra 2023	111.15	11.3	20	111.26	6.41	21	1.9%	-0.11 [-5.77, 5.55]
Boer 2014(1)	113	8	17	119	10	7	1.5%	-6.00 [-14.33, 2.33]
Boer 2014(2)	119	9	15	119	10	7	1.4%	0.00 [-8.70, 8.70]
Espinoza-Silva 2019(1)	103.06	13.53	69	102.6	10.14	30	2.0%	0.46 [-4.37, 5.29]
Faria 2020(1)	115.7	8.05	24	117.3	8.29	13	1.9%	-1.60 [-7.14, 3.94]
Faria 2020(2)	114.5	8.29	26	117.3	8.29	13	1.9%	-2.80 [-8.32, 2.72]
Harder-Lauridsen 2014	104.5	9.38	18	102.3	9.98	18	1.8%	2.20 [-4.13, 8.53]
Kelly 2004	120	6.32	10	122	15.81	10	1.2%	-2.00 [-12.55, 8.55]
Murphy 2009	111.4	8	23	114.2	5.7	12	2.1%	-2.80 [-7.39, 1.79]
Woo 2012	103.05	7.75	10	116.3	7.18	10	1.8%	-13.25 [-19.80, -6.70]
**Subtotal (95% CI)**			**232**			**141**	**17.5%**	**-2.43 [-5.04, 0.18]**
Heterogeneity: Tau² = 7.27; Chi² = 15.68, df = 9 (P = 0.07); I² = 43%								
Test for overall effect: Z = 1.82 (P = 0.07)								
**2.9.2 Obesity**								
Bharath 2018	120	1.79	20	124	6.26	20	2.3%	-4.00 [-6.85, -1.15]
Bruyndonckx 2015	108	6	27	120	11	21	2.0%	-12.00 [-17.22, -6.78]
Chuensiri 2018(1)	125	13.27	11	121	13.27	5	0.8%	4.00 [-10.03, 18.03]
Chuensiri 2018(2)	116	11.62	15	121	13.27	6	1.0%	-5.00 [-17.14, 7.14]
Espinoza-Silva 2019(2)	103.03	8.49	141	102.91	8.9	34	2.3%	0.12 [-3.18, 3.42]
Farpour-Lambert 2009	120.7	13.69	22	125.7	14.85	22	1.5%	-5.00 [-13.44, 3.44]
Guijun 2009	114.97	1.5	16	123.15	2.7	10	2.4%	-8.18 [-10.01, -6.35]
Hongling 2012	113	5	30	116	6	30	2.3%	-3.00 [-5.79, -0.21]
Horner 2015(1)	109.58	19.56	27	116.63	10.23	8	1.2%	-7.05 [-17.28, 3.18]
Horner 2015(2)	110.89	8.04	23	116.63	10.23	8	1.6%	-5.74 [-13.55, 2.07]
Jian 2014	120.55	2.56	44	129.72	2.24	44	2.5%	-9.17 [-10.18, -8.16]
Kai 2024	127	1	20	132	3	24	2.5%	-5.00 [-6.28, -3.72]
Kelishadi 2008	116.9	45.88	45	116.7	45.9	47	0.6%	0.20 [-18.56, 18.96]
Kim 2007	122.9	7.11	14	124.2	10.74	12	1.7%	-1.30 [-8.43, 5.83]
Kim 2011	114	10.39	12	118	15.81	10	1.1%	-4.00 [-15.43, 7.43]
Kim 2020	127.6	2.3	24	133.2	4.1	24	2.4%	-5.60 [-7.48, -3.72]
Lee 2010(1)	119.34	15.19	20	121.12	16.03	9	1.0%	-1.78 [-14.19, 10.63]
Lee 2010(2)	116.53	10.99	16	121.12	16.03	9	1.0%	-4.59 [-16.37, 7.19]
Luo 2013(1)	100.72	6.21	43	112.21	8.26	34	2.3%	-11.49 [-14.83, -8.15]
Luo 2013(2)	97.5	6.22	52	107.89	9.11	38	2.3%	-10.39 [-13.74, -7.04]
Meng 2022(1)	110	5	12	116	4	6	2.1%	-6.00 [-10.27, -1.73]
Meng 2022(2)	114	7	11	116	4	7	2.0%	-2.00 [-7.09, 3.09]
Meyer 2006	120	13.3	33	133	19.6	34	1.5%	-13.00 [-21.00, -5.00]
Park 2007	112	7.6	19	119.3	7.5	21	2.1%	-7.30 [-11.99, -2.61]
Plavsic 2020	110.3	8.5	22	114.4	6.8	22	2.1%	-4.10 [-8.65, 0.45]
Racil(1) 2016(1)	115	5	17	118	5	7	2.1%	-3.00 [-7.40, 1.40]
Racil(1) 2016(2)	115	5	16	118	5	7	2.1%	-3.00 [-7.44, 1.44]
Racil 2024(1)	119.15	5	12	119.1	4.7	5	2.0%	0.05 [-4.95, 5.05]
Racil 2024(2)	119.33	4.7	12	119.1	4.7	6	2.1%	0.23 [-4.38, 4.84]
Salus(1) 2022	125.9	8.98	14	120.7	16.46	14	1.3%	5.20 [-4.62, 15.02]
Savoye 2007	121	11	75	120.4	12.54	44	2.1%	0.60 [-3.86, 5.06]
Savoye 2011	121.7	10.24	75	121.7	10.82	44	2.2%	0.00 [-3.95, 3.95]
Saygın 2011	121.6	1.03	20	135.1	0.84	19	2.5%	-13.50 [-14.09, -12.91]
Seo 2019	123.06	11.07	26	123.91	15.95	44	1.8%	-0.85 [-7.20, 5.50]
Shalitin 2009	111	17.8	55	112	18.54	55	1.7%	-1.00 [-7.79, 5.79]
Shu 2023(1)	111.9	4.7	16	117.1	5	8	2.1%	-5.20 [-9.36, -1.04]
Shu 2023(2)	113.2	6.3	15	117.1	5	8	2.1%	-3.90 [-8.61, 0.81]
Son 2017	123.7	9.53	20	130.6	6.22	20	2.0%	-6.90 [-11.89, -1.91]
Vasconcellos 2016	123	13	10	129	6	10	1.4%	-6.00 [-14.87, 2.87]
Wong 2008	113.8	7.1	12	117	6.2	12	2.0%	-3.20 [-8.53, 2.13]
Yu 2020	102.7	8.1	99	102.1	8.9	72	2.4%	0.60 [-2.00, 3.20]
Yuanyuan 2019(1)	105.47	7.21	10	106.57	7.62	3	1.3%	-1.10 [-10.81, 8.61]
Yuanyuan 2019(2)	99.01	7.24	10	106.57	7.62	3	1.3%	-7.56 [-17.28, 2.16]
Yuanyuan 2019(3)	97.65	6.62	9	106.57	7.62	4	1.4%	-8.92 [-17.55, -0.29]
Zhaogang 2021	98.24	9.64	49	101.24	10.04	49	2.2%	-3.00 [-6.90, 0.90]
**Subtotal (95% CI)**			**1291**			**939**	**82.5%**	**-4.56 [-6.28, -2.85]**
Heterogeneity: Tau² = 24.79; Chi² = 543.48, df = 44 (P < 0.00001); I² = 92%								
Test for overall effect: Z = 5.22 (P < 0.00001)								
**Total (95% CI)**			**1523**			**1080**	**100.0%**	**-4.20 [-5.78, -2.62]**
Heterogeneity: Tau² = 25.80; Chi² = 606.24, df = 54 (P < 0.00001); I² = 91%								
Test for overall effect: Z = 5.21 (P < 0.00001)								
Test for subgroup differences: Chi² = 1.80, df = 1 (P = 0.18), I² = 44.4%								

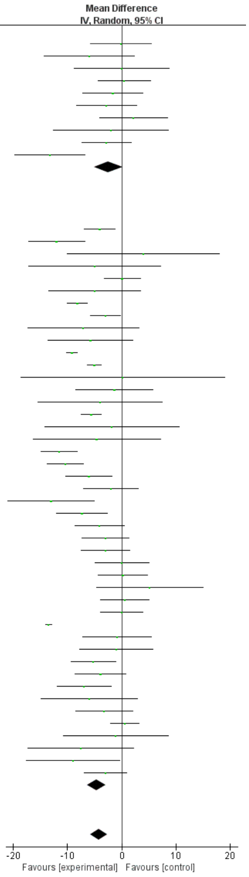

Figure S9.10. Forest plot of subgroup analyses by sex

Study or Subgroup	Experimental Mean	Experimental SD	Experimental Total	Control Mean	Control SD	Control Total	Weight	Mean Difference IV, Random, 95% CI
**2.10.1 Boys**								
Chuensiri 2018(1)	125	13.27	11	121	13.27	5	1.5%	4.00 [-10.03, 18.03]
Chuensiri 2018(2)	116	11.62	15	121	13.27	6	1.8%	-5.00 [-17.14, 7.14]
Cvetković 2018(1)	117.5	11.12	10	124.85	11.58	7	2.0%	-7.35 [-18.35, 3.65]
Cvetković 2018(2)	116.5	14.15	11	124.85	11.58	7	1.8%	-8.35 [-20.33, 3.63]
Hongling 2012	113	5	30	116	6	30	4.4%	-3.00 [-5.79, -0.21]
Kim 2007	122.9	7.11	14	124.2	10.74	12	3.1%	-1.30 [-8.43, 5.83]
Kim 2011	114	10.39	12	118	15.81	10	1.9%	-4.00 [-15.43, 7.43]
Luo 2013(2)	97.5	6.22	52	107.89	9.11	38	4.3%	-10.39 [-13.74, -7.04]
Meng 2022(1)	110	5	12	116	4	6	4.0%	-6.00 [-10.27, -1.73]
Meng 2022(2)	114	7	11	116	4	7	3.7%	-2.00 [-7.09, 3.09]
Salus(1) 2022	125.9	8.98	14	120.7	16.46	14	2.3%	5.20 [-4.62, 15.02]
Wong 2008	113.8	7.1	12	117	6.2	12	3.6%	-3.20 [-8.53, 2.13]
**Subtotal (95% CI)**			**204**			**154**	**34.5%**	**-4.21 [-6.74, -1.67]**
Heterogeneity: Tau² = 7.81; Chi² = 21.06, df = 11 (P = 0.03); I² = 48%								
Test for overall effect: Z = 3.25 (P = 0.001)								
**2.10.2 Girls**								
Abassi 2022(1)	122	2.3	13	125	3.8	6	4.3%	-3.00 [-6.29, 0.29]
Abassi 2022(2)	123	3.8	13	125	3.8	6	4.2%	-2.00 [-5.68, 1.68]
Bharath 2018	120	1.79	20	124	6.26	20	4.4%	-4.00 [-6.85, -1.15]
Heidarianpour 2023	108.22	12.39	15	118.2	13.47	15	2.4%	-9.98 [-19.24, -0.72]
Jian 2014	120.55	2.56	44	129.72	2.24	44	4.8%	-9.17 [-10.18, -8.16]
Kai 2024	127	1	20	132	3	24	4.7%	-5.00 [-6.28, -3.72]
Kim 2020	127.6	2.3	24	133.2	4.1	24	4.6%	-5.60 [-7.48, -3.72]
Luo 2013(1)	100.72	6.21	43	112.21	8.26	34	4.3%	-11.49 [-14.83, -8.15]
Park 2007	112	7.6	19	119.3	7.5	21	3.9%	-7.30 [-11.99, -2.61]
Plavsic 2020	110.3	8.5	22	114.4	6.8	22	3.9%	-4.10 [-8.65, 0.45]
Racil(1) 2016(1)	115	5	17	118	5	7	3.9%	-3.00 [-7.40, 1.40]
Racil(1) 2016(2)	115	5	16	118	5	7	3.9%	-3.00 [-7.44, 1.44]
Racil 2024(1)	119.15	5	12	119.1	4.7	5	3.8%	0.05 [-4.95, 5.05]
Racil 2024(2)	119.33	4.7	12	119.1	4.7	6	3.9%	0.23 [-4.38, 4.84]
Saygın 2011	121.6	1.03	20	135.1	0.84	19	4.8%	-13.50 [-14.09, -12.91]
Son 2017	123.7	9.53	20	130.6	6.22	20	3.8%	-6.90 [-11.89, -1.91]
**Subtotal (95% CI)**			**330**			**280**	**65.5%**	**-5.54 [-8.06, -3.02]**
Heterogeneity: Tau² = 22.46; Chi² = 333.94, df = 15 (P < 0.00001); I² = 96%								
Test for overall effect: Z = 4.31 (P < 0.0001)								
**Total (95% CI)**			**534**			**434**	**100.0%**	**-4.97 [-7.02, -2.91]**
Heterogeneity: Tau² = 22.84; Chi² = 397.80, df = 27 (P < 0.00001); I² = 93%								
Test for overall effect: Z = 4.73 (P < 0.00001)								
Test for subgroup differences: Chi² = 0.53, df = 1 (P = 0.46), I² = 0%								

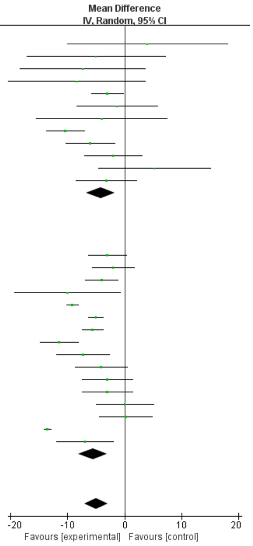

Figure S10. Forest plots of subgroup analyses for DBP in children and adolescents with overweight or obesity

Figure S10.1. Forest plot of subgroup analyses by type of exercise

Study or Subgroup	Experimental Mean	Experimental SD	Experimental Total	Control Mean	Control SD	Control Total	Weight	Mean Difference IV, Random, 95% CI
**2.1.1 Aerobic exercise**								
Abassi 2022(2)	77.4	4.42	13	77.8	4	6	1.6%	-0.40 [-4.40, 3.60]
Aguilar-Cordero 2020	72.77	4.97	49	78.41	8.7	49	1.8%	-5.64 [-8.45, -2.83]
Bezerra 2023	70.05	11.73	20	69.51	7.69	21	1.2%	0.54 [-5.56, 6.64]
Boer 2014(2)	73	9	15	72	5	7	1.2%	1.00 [-4.87, 6.87]
Boff 2020	72.61	14	34	73.22	7.72	31	1.3%	-0.61 [-6.04, 4.82]
Cvetković 2018(1)	64	3.94	10	64.57	8.42	7	1.1%	-0.57 [-7.27, 6.13]
Davis 2020	59.1	5.38	90	58	5.23	85	2.0%	1.10 [-0.47, 2.67]
Guijun 2009	84	1.28	16	88.5	1.57	10	2.0%	-4.50 [-5.66, -3.34]
Hamila 2018(1)	64	3.9	7	68.7	6.9	2	0.7%	-4.70 [-14.69, 5.29]
Hamila 2018(2)	60.1	6.3	8	68.7	6.9	3	0.8%	-8.60 [-17.55, 0.35]
Hamila 2018(3)	68.3	5.3	8	68.7	6.9	3	0.8%	-0.40 [-9.03, 8.23]
Horner 2015(1)	58.75	10.88	27	60.88	6.48	8	1.2%	-2.13 [-8.21, 3.95]
Jain 2022	71	7	40	75	9	27	1.5%	-4.00 [-8.03, 0.03]
Jian 2014	75.56	1.45	44	84.81	1.9	44	2.1%	-9.25 [-9.96, -8.54]
Kai 2024	81	1	20	82	1	24	2.1%	-1.00 [-1.59, -0.41]
Kelishadi 2008	74.6	11.81	45	74.4	11.74	47	1.4%	0.20 [-4.61, 5.01]
Kelly 2004	65	6.32	10	68	9.49	10	1.0%	-3.00 [-10.07, 4.07]
Kim 2007	81.4	10.85	14	80.8	10.74	12	0.8%	0.60 [-7.72, 8.92]
Kim 2011	69	6.93	12	65	6.32	10	1.3%	4.00 [-1.54, 9.54]
Kim 2020	80.2	1.4	24	81.2	2.8	24	2.0%	-1.00 [-2.25, 0.25]
Liqun 2014	70.6	8.9	76	72.4	9	97	1.8%	-1.80 [-4.49, 0.89]
Luo 2013(1)	57.3	7.35	43	64.09	6.15	34	1.7%	-6.79 [-9.81, -3.77]
Luo 2013(2)	57	5.32	52	60.48	7.32	38	1.8%	-3.48 [-6.22, -0.74]
Machado 2022	56.9	5.8	20	63.2	13.4	15	1.0%	-6.30 [-13.54, 0.94]
Meng 2022(2)	68	4	11	71	3.7	7	1.6%	-3.00 [-6.62, 0.62]
Murphy 2009	70.9	8.4	23	71.5	7.5	12	1.3%	-0.60 [-6.06, 4.86]
Park 2007	67.5	4.6	19	69.9	6.5	21	1.7%	-2.40 [-5.87, 1.07]
Plavsic 2020	69.2	6.2	22	72.4	6.6	22	1.6%	-3.20 [-6.98, 0.58]
Racil(1) 2016(2)	75	4	16	74	2	7	1.8%	1.00 [-1.46, 3.46]
Racil 2024(2)	79.23	3.6	12	80.2	3.5	6	1.7%	-0.97 [-4.43, 2.49]
Savoye 2007	66.4	9.41	75	67.8	9.17	44	1.7%	-1.40 [-4.85, 2.05]
Savoye 2011	65.3	9.3	45	64.3	9.7	31	1.5%	1.00 [-3.36, 5.36]
Seo 2019	61.44	9.47	26	65.59	6.43	44	1.5%	-4.15 [-8.26, -0.04]
Shu 2023(2)	69	3.5	15	70.3	4.8	8	1.6%	-1.30 [-5.07, 2.47]
Sigal 2014(1)	72	8.66	75	73	8.72	25	1.6%	-1.00 [-4.94, 2.94]
Tjonna 2009	64.9	7.16	20	67.3	7.5	22	1.5%	-2.40 [-6.83, 2.03]
Woo 2012	73.45	9.57	10	72.25	5.76	10	1.0%	1.20 [-5.72, 8.12]
Yu 2020	67.4	6.1	99	68.5	6.5	72	1.9%	-1.10 [-3.02, 0.82]
Yuanyuan 2019(1)	66.89	5.79	10	67.13	5.88	3	0.9%	-0.24 [-7.80, 7.32]
Yuanyuan 2019(2)	61.02	5.76	10	67.13	5.88	3	0.9%	-6.11 [-13.66, 1.44]
Zhaogang 2021	66.17	6.27	49	67.54	7.58	49	1.8%	-1.37 [-4.12, 1.38]
**Subtotal (95% CI)**			**1234**			**1000**	**59.6%**	**-2.04 [-3.39, -0.69]**
Heterogeneity: Tau² = 13.98; Chi² = 438.14, df = 40 (P < 0.00001); I² = 91%								
Test for overall effect: Z = 2.97 (P = 0.003)								
**2.1.2 Anaerobic exercise**								
Abassi 2022(1)	76.7	4.22	13	77.8	4	6	1.6%	-1.10 [-5.04, 2.84]
Boer 2014(1)	77	8	17	72	5	7	1.3%	5.00 [-0.31, 10.31]
Chuensiri 2018(1)	77	6.63	11	74	6.63	5	1.0%	3.00 [-4.01, 10.01]
Chuensiri 2018(2)	71	7.75	15	74	6.63	6	1.1%	-3.00 [-9.60, 3.60]
Cvetković 2018(2)	66	8	11	64.57	8.42	7	0.9%	1.43 [-6.40, 9.26]
Espinoza-Silva 2019(1)	61.42	14.53	69	61.45	9.09	30	1.4%	-0.03 [-4.76, 4.70]
Espinoza-Silva 2019(2)	62.32	8.66	141	57.41	9.24	34	1.7%	4.91 [1.49, 8.33]
Gonzalez-Galvez 2024(1)	61.4	7.8	9	67.4	9.01	6	0.8%	-6.00 [-14.83, 2.83]
Gonzalez-Galvez 2024(2)	64.9	7.63	11	67.4	9.01	6	0.8%	-2.50 [-11.00, 6.00]
Meng 2022(1)	67	3	12	71	3.7	6	1.7%	-4.00 [-7.41, -0.59]
Racil(1) 2016(1)	74	2	17	74	2	7	2.0%	0.00 [-1.76, 1.76]
Racil 2024(1)	78.61	3.6	12	80.2	3.5	5	1.6%	-1.59 [-5.27, 2.09]
Salus(1) 2022	71.3	13.1	14	78.4	8.61	14	0.9%	-7.10 [-15.31, 1.11]
Shu 2023(1)	66.1	2.4	16	70.3	4.8	8	1.6%	-4.20 [-7.73, -0.67]
Vasconcellos 2016	79	14	10	79	4	10	0.8%	0.00 [-9.02, 9.02]
Yuanyuan 2019(3)	60.12	5.24	9	67.13	5.88	4	1.1%	-7.01 [-13.71, -0.31]
**Subtotal (95% CI)**			**387**			**161**	**20.1%**	**-1.01 [-2.78, 0.75]**
Heterogeneity: Tau² = 5.77; Chi² = 32.23, df = 15 (P = 0.006); I² = 53%								
Test for overall effect: Z = 1.13 (P = 0.26)								
**2.1.3 Resistance exercise**								
Horner 2015(2)	58.56	7.29	23	60.88	6.48	8	1.3%	-2.32 [-7.71, 3.07]
Sigal 2014(2)	74	8.83	78	73	8.72	25	1.6%	1.00 [-2.94, 4.94]
**Subtotal (95% CI)**			**101**			**33**	**2.8%**	**-0.16 [-3.34, 3.02]**
Heterogeneity: Tau² = 0.00; Chi² = 0.95, df = 1 (P = 0.33); I² = 0%								
Test for overall effect: Z = 0.10 (P = 0.92)								
**2.1.4 Combined aerobic and resistance exercise**								
Bharath 2018	78	6.93	20	80	10.73	20	1.2%	-2.00 [-7.60, 3.60]
Bruyndonckx 2015	60	4	27	63	7	21	1.7%	-3.00 [-6.35, 0.35]
Faria 2020(1)	70.5	6.87	24	70.8	7.06	13	1.4%	-0.30 [-5.02, 4.42]
Faria 2020(2)	68.2	7.06	26	70.8	7.06	13	1.4%	-2.60 [-7.30, 2.10]
Farpour-Lambert 2009	70.9	5	22	71.8	5.43	22	1.7%	-0.90 [-3.98, 2.18]
Harder-Lauridsen 2014	60.9	11.24	18	62.6	11.1	18	1.0%	-1.70 [-9.00, 5.60]
Heidarianpour 2023	77.53	4.35	15	80	4.7	15	1.7%	-2.47 [-5.71, 0.77]
Hongling 2012	71	5	30	73	6	30	1.8%	-2.00 [-4.79, 0.79]
Shalitin 2009	64	12.9	55	63	13.65	55	1.4%	1.00 [-3.96, 5.96]
Sigal 2014(3)	73	8.66	75	73	8.72	26	1.6%	0.00 [-3.88, 3.88]
Son 2017	79.8	6.62	20	82.4	8.9	20	1.4%	-2.60 [-7.46, 2.26]
Wong 2008	71.7	7.5	12	70.8	6.7	12	1.2%	0.90 [-4.79, 6.59]
**Subtotal (95% CI)**			**344**			**265**	**17.5%**	**-1.52 [-2.68, -0.35]**
Heterogeneity: Tau² = 0.00; Chi² = 4.30, df = 11 (P = 0.96); I² = 0%								
Test for overall effect: Z = 2.56 (P = 0.01)								
**Total (95% CI)**			**2066**			**1459**	**100.0%**	**-1.70 [-2.68, -0.71]**
Heterogeneity: Tau² = 12.08; Chi² = 506.73, df = 70 (P < 0.00001); I² = 86%								
Test for overall effect: Z = 3.37 (P = 0.0007)								
Test for subgroup differences: Chi² = 1.59, df = 3 (P = 0.66), I² = 0%								

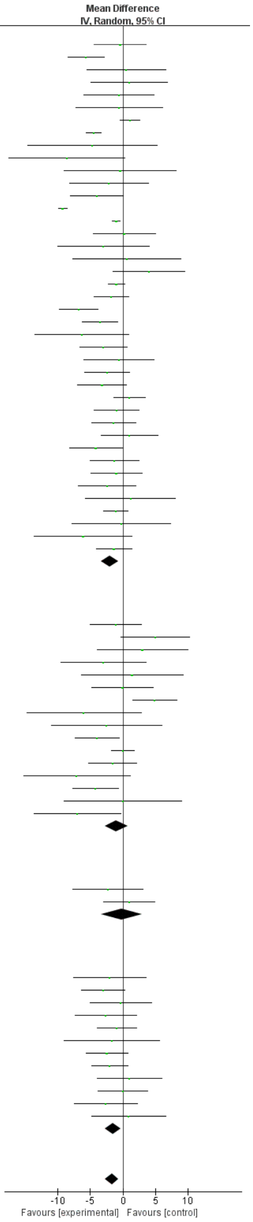

Figure S10.2. Forest plot of subgroup analyses by exercise intensity

Study or Subgroup	Experimental Mean	Experimental SD	Experimental Total	Control Mean	Control SD	Control Total	Weight	Mean Difference IV, Random, 95% CI
**2.2.1 Moderate-to-low intensity**								
Abassi 2022(2)	77.4	4.42	13	77.8	4	6	1.6%	-0.40 [-4.40, 3.60]
Aguilar-Cordero 2020	72.77	4.97	49	78.41	8.7	49	1.8%	-5.64 [-8.45, -2.83]
Bharath 2018	78	6.93	20	80	10.73	20	1.2%	-2.00 [-7.60, 3.60]
Boer 2014(2)	73	9	15	72	5	7	1.2%	1.00 [-4.87, 6.87]
Boff 2020	72.61	14	34	73.22	7.72	31	1.3%	-0.61 [-6.04, 4.82]
Bruyndonckx 2015	60	4	27	63	7	21	1.7%	-3.00 [-6.35, 0.35]
Cvetković 2018(1)	64	3.94	10	64.57	8.42	7	1.1%	-0.57 [-7.27, 6.13]
Davis 2020	59.1	5.38	90	58	5.23	85	2.0%	1.10 [-0.47, 2.67]
Faria 2020(1)	70.5	6.87	24	70.8	7.06	13	1.4%	-0.30 [-5.02, 4.42]
Farpour-Lambert 2009	70.9	5	22	71.8	5.43	22	1.7%	-0.90 [-3.98, 2.18]
Guijun 2009	84	1.28	16	88.5	1.57	10	2.0%	-4.50 [-5.66, -3.34]
Hamila 2018(1)	64	3.9	7	68.7	6.9	2	0.7%	-4.70 [-14.69, 5.29]
Hamila 2018(2)	60.1	6.3	8	68.7	6.9	3	0.8%	-8.60 [-17.55, 0.35]
Hamila 2018(3)	68.3	5.3	8	68.7	6.9	3	0.8%	-0.40 [-9.03, 8.23]
Harder-Lauridsen 2014	60.9	11.24	18	62.6	11.1	18	1.0%	-1.70 [-9.00, 5.60]
Heidarianpour 2023	77.53	4.35	15	80	4.7	15	1.7%	-2.47 [-5.71, 0.77]
Horner 2015(1)	58.75	10.88	27	60.88	6.48	8	1.2%	-2.13 [-8.21, 3.95]
Jain 2022	71	7	40	75	9	27	1.5%	-4.00 [-8.03, 0.03]
Jian 2014	75.56	1.45	44	84.81	1.9	44	2.1%	-9.25 [-9.96, -8.54]
Kai 2024	81	1	20	82	1	24	2.1%	-1.00 [-1.59, -0.41]
Kelishadi 2008	74.6	11.81	45	74.4	11.74	47	1.4%	0.20 [-4.61, 5.01]
Kelly 2004	65	6.32	10	68	9.49	10	1.0%	-3.00 [-10.07, 4.07]
Kim 2007	81.4	10.85	14	80.8	10.74	12	0.8%	0.60 [-7.72, 8.92]
Kim 2011	69	6.93	12	65	6.32	10	1.3%	4.00 [-1.54, 9.54]
Kim 2020	80.2	1.4	24	81.2	2.8	24	2.0%	-1.00 [-2.25, 0.25]
Liqun 2014	70.6	8.9	76	72.4	9	97	1.8%	-1.80 [-4.49, 0.89]
Luo 2013(1)	57.3	7.35	43	64.09	6.15	34	1.7%	-6.79 [-9.81, -3.77]
Luo 2013(2)	57	5.32	52	60.48	7.32	38	1.8%	-3.48 [-6.22, -0.74]
Machado 2022	56.9	5.8	20	63.2	13.4	15	1.0%	-6.30 [-13.54, 0.94]
Meng 2022(2)	68	4	11	71	3.7	7	1.6%	-3.00 [-6.62, 0.62]
Murphy 2009	70.9	8.4	23	71.5	7.5	12	1.3%	-0.60 [-6.06, 4.86]
Park 2007	67.5	4.6	19	69.9	6.5	21	1.7%	-2.40 [-5.87, 1.07]
Racil(1) 2016(2)	75	4	16	74	2	7	1.8%	1.00 [-1.46, 3.46]
Racil 2024(2)	79.23	3.6	12	80.2	3.5	6	1.7%	-0.97 [-4.43, 2.49]
Seo 2019	61.44	9.47	26	65.59	6.43	44	1.5%	-4.15 [-8.26, -0.04]
Shalitin 2009	64	12.9	55	63	13.65	55	1.4%	1.00 [-3.96, 5.96]
Shu 2023(2)	69	3.5	15	70.3	4.8	8	1.6%	-1.30 [-5.07, 2.47]
Son 2017	79.8	6.62	20	82.4	8.9	20	1.4%	-2.60 [-7.46, 2.26]
Woo 2012	73.45	9.57	10	72.25	5.76	10	1.0%	1.20 [-5.72, 8.12]
Yu 2020	67.4	6.1	99	68.5	6.5	72	1.9%	-1.10 [-3.02, 0.82]
Yuanyuan 2019(1)	66.89	5.79	10	67.13	5.88	3	0.9%	-0.24 [-7.80, 7.32]
Yuanyuan 2019(2)	61.02	5.76	10	67.13	5.88	3	0.9%	-6.11 [-13.66, 1.44]
Zhaogang 2021	66.17	6.27	49	67.54	7.58	49	1.8%	-1.37 [-4.12, 1.38]
**Subtotal (95% CI)**			**1178**			**1019**	**62.1%**	**-2.07 [-3.38, -0.77]**
Heterogeneity: Tau² = 13.64; Chi² = 437.75, df = 42 (P < 0.00001); I² = 90%								
Test for overall effect: Z = 3.11 (P = 0.002)								
**2.2.2 High intensity**								
Abassi 2022(1)	76.7	4.22	13	77.8	4	6	1.6%	-1.10 [-5.04, 2.84]
Bezerra 2023	70.05	11.73	20	69.51	7.69	21	1.2%	0.54 [-5.56, 6.64]
Boer 2014(1)	77	8	17	72	5	7	1.3%	5.00 [-0.31, 10.31]
Chuensiri 2018(1)	77	6.63	11	74	6.63	5	1.0%	3.00 [-4.01, 10.01]
Chuensiri 2018(2)	71	7.75	15	74	6.63	6	1.1%	-3.00 [-9.60, 3.60]
Cvetković 2018(2)	66	8	11	64.57	8.42	7	0.9%	1.43 [-6.40, 9.26]
Espinoza-Silva 2019(1)	61.42	14.53	69	61.45	9.09	30	1.4%	-0.03 [-4.76, 4.70]
Espinoza-Silva 2019(2)	62.32	8.66	141	57.41	9.24	34	1.7%	4.91 [1.49, 8.33]
Faria 2020(2)	68.2	7.06	26	70.8	7.06	13	1.4%	-2.60 [-7.30, 2.10]
Gonzalez-Galvez 2024(1)	61.4	7.8	9	67.4	9.01	6	0.8%	-6.00 [-14.83, 2.83]
Gonzalez-Galvez 2024(2)	64.9	7.63	11	67.4	9.01	6	0.8%	-2.50 [-11.00, 6.00]
Hongling 2012	71	5	30	73	6	30	1.8%	-2.00 [-4.79, 0.79]
Horner 2015(2)	58.56	7.29	23	60.88	6.48	8	1.3%	-2.32 [-7.71, 3.07]
Meng 2022(1)	67	3	12	71	3.7	6	1.7%	-4.00 [-7.41, -0.59]
Plavsic 2020	69.2	6.2	22	72.4	6.6	22	1.6%	-3.20 [-6.98, 0.58]
Racil(1) 2016(1)	74	2	17	74	2	7	2.0%	0.00 [-1.76, 1.76]
Racil 2024(1)	78.61	3.6	12	80.2	3.5	5	1.6%	-1.59 [-5.27, 2.09]
Salus(1) 2022	71.3	13.1	14	78.4	8.61	14	0.9%	-7.10 [-15.31, 1.11]
Savoye 2007	66.4	9.41	75	67.8	9.17	44	1.7%	-1.40 [-4.85, 2.05]
Savoye 2011	65.3	9.3	45	64.3	9.7	31	1.5%	1.00 [-3.36, 5.36]
Shu 2023(1)	66.1	2.4	16	70.3	4.8	8	1.6%	-4.20 [-7.73, -0.67]
Sigal 2014(1)	72	8.66	75	73	8.72	25	1.6%	-1.00 [-4.94, 2.94]
Sigal 2014(2)	74	8.83	78	73	8.72	25	1.6%	1.00 [-2.94, 4.94]
Sigal 2014(3)	73	8.66	75	73	8.72	26	1.6%	0.00 [-3.88, 3.88]
Tjonna 2009	64.9	7.16	20	67.3	7.5	22	1.5%	-2.40 [-6.83, 2.03]
Vasconcellos 2016	79	14	10	79	4	10	0.8%	0.00 [-9.02, 9.02]
Wong 2008	71.7	7.5	12	70.8	6.7	12	1.2%	0.90 [-4.79, 6.59]
Yuanyuan 2019(3)	60.12	5.24	9	67.13	5.88	4	1.1%	-7.01 [-13.71, -0.31]
**Subtotal (95% CI)**			**888**			**440**	**37.9%**	**-0.98 [-1.98, 0.02]**
Heterogeneity: Tau² = 1.89; Chi² = 38.14, df = 27 (P = 0.08); I² = 29%								
Test for overall effect: Z = 1.93 (P = 0.05)								
**Total (95% CI)**			**2066**			**1459**	**100.0%**	**-1.70 [-2.68, -0.71]**
Heterogeneity: Tau² = 12.08; Chi² = 506.73, df = 70 (P < 0.00001); I² = 86%								
Test for overall effect: Z = 3.37 (P = 0.0007)								
Test for subgroup differences: Chi² = 1.70, df = 1 (P = 0.19), I² = 41.1%								

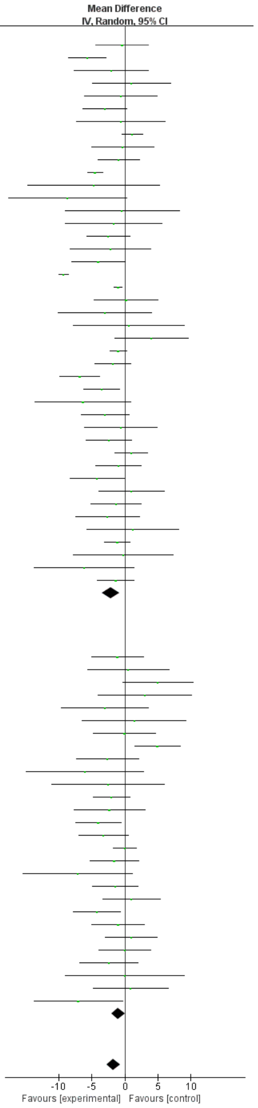

Figure S10.3. Forest plot of subgroup analyses by duration per session

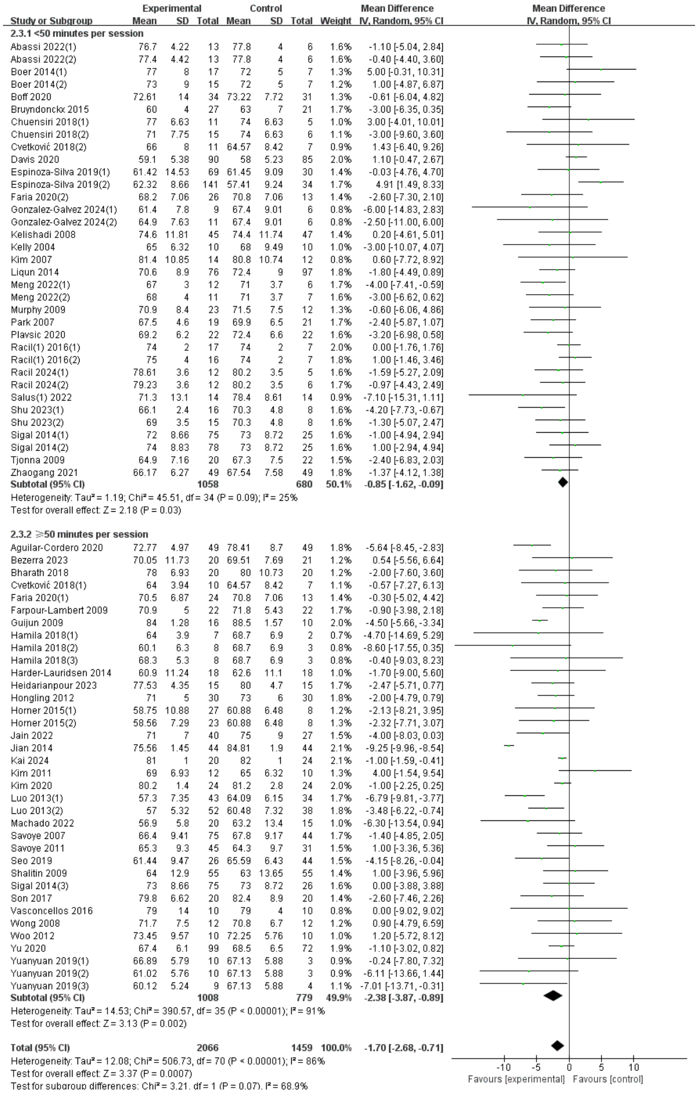

Figure S10.4. Forest plot of subgroup analyses by exercise frequency

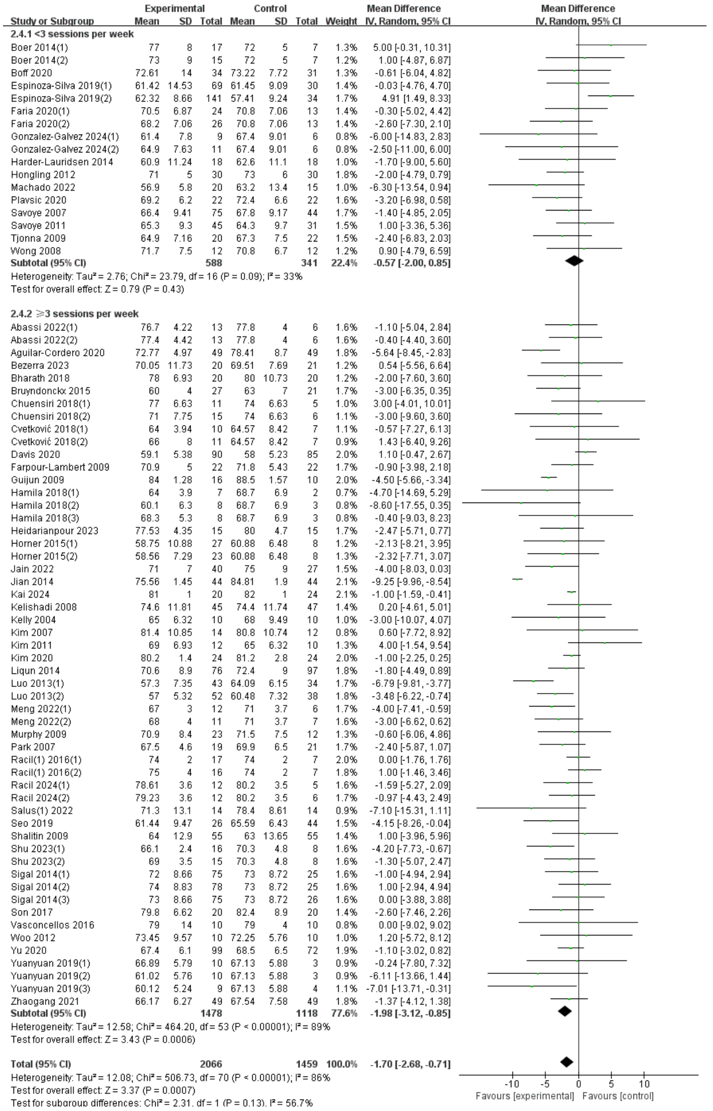

Figure S10.5. Forest plot of subgroup analyses by weekly exercise duration

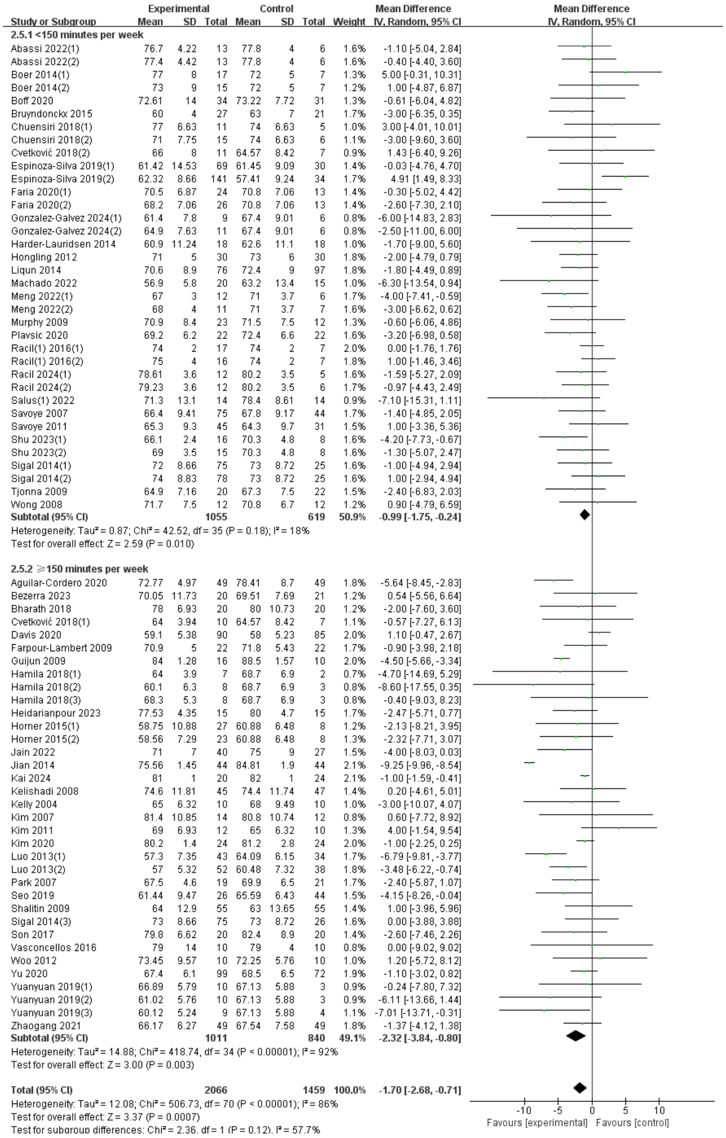

Figure S10.6. Forest plot of subgroup analyses by intervention duration

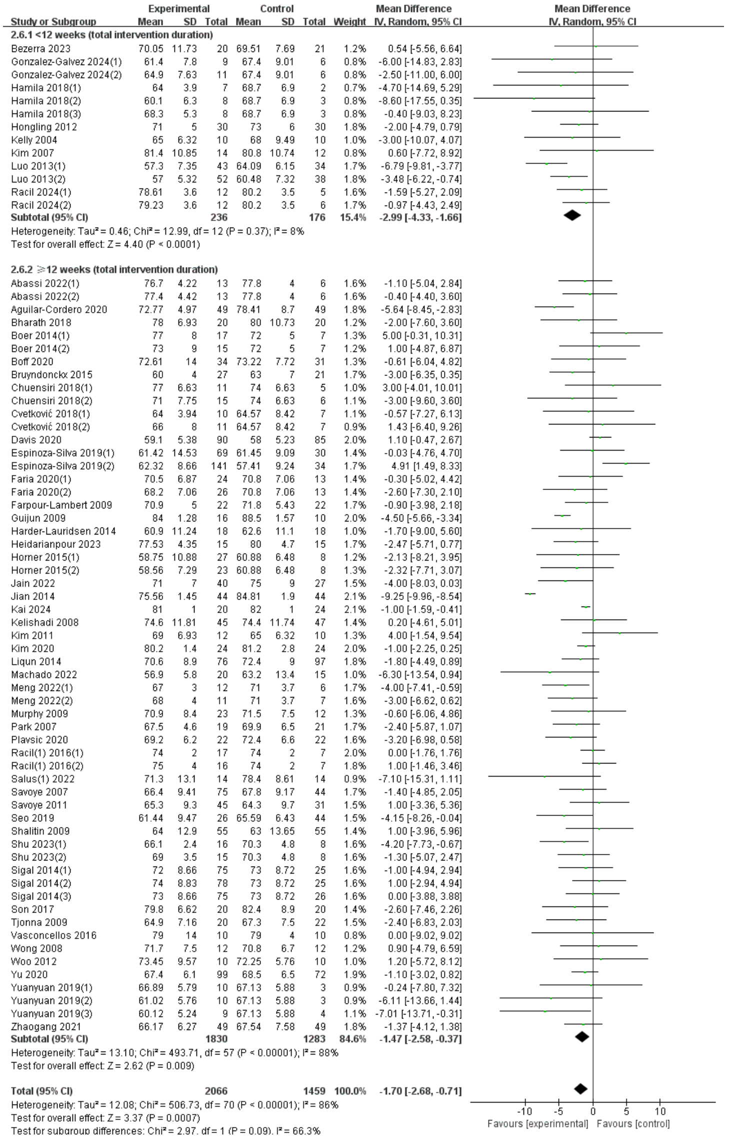

Figure S10.7. Forest plot of subgroup analyses by region

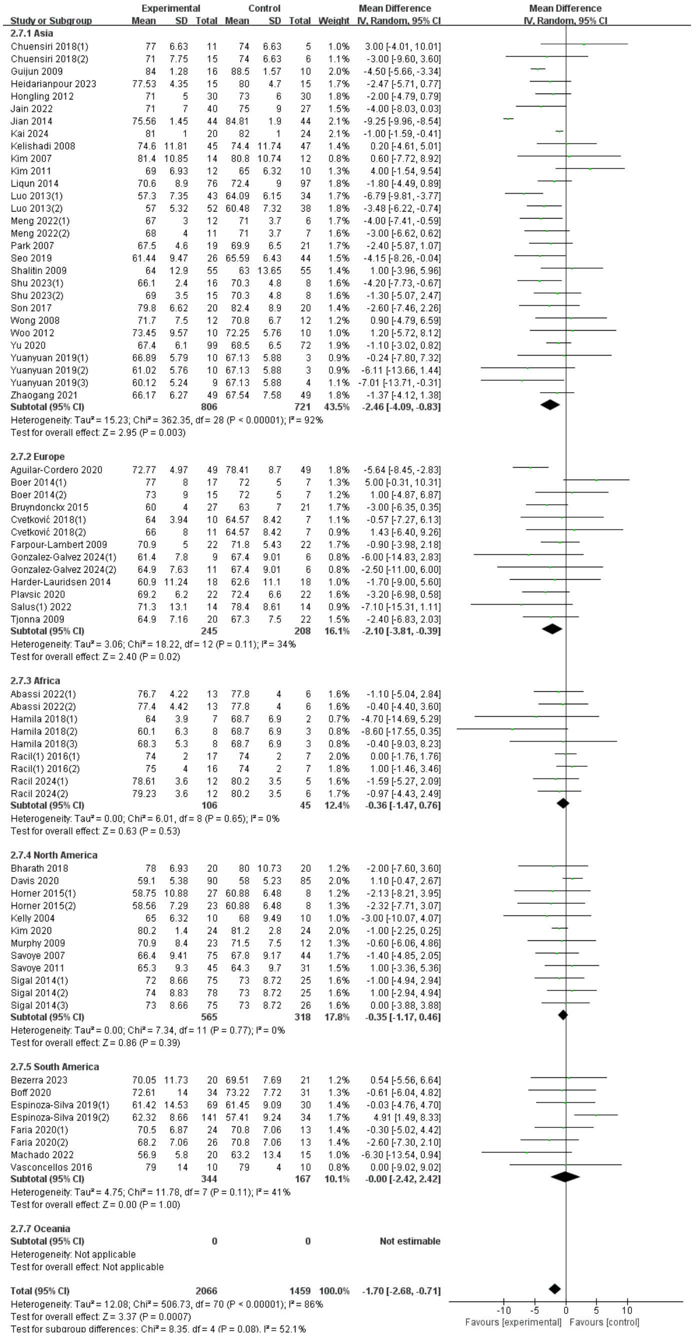

Figure S10.8. Forest plot of subgroup analyses by economic status

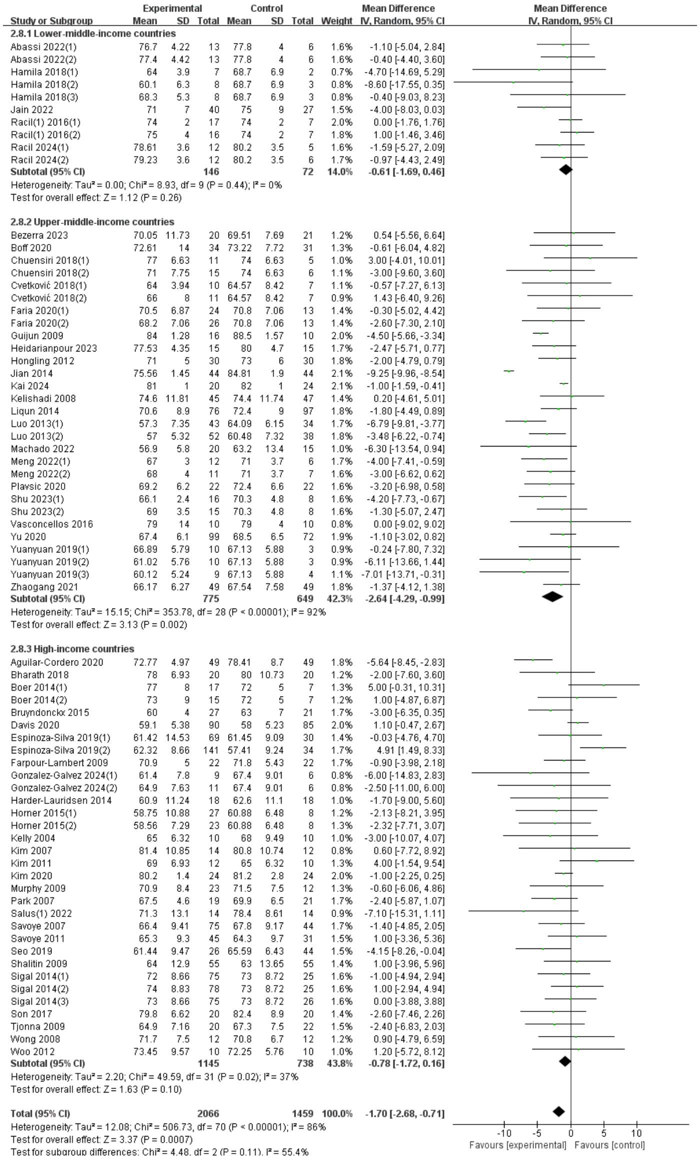

Figure S10.9. Forest plot of subgroup analyses by weight status

Study or Subgroup	Experimental Mean	Experimental SD	Experimental Total	Control Mean	Control SD	Control Total	Weight	Mean Difference IV, Random, 95% CI
**2.9.1 Overweight**								
Bezerra 2023	70.05	11.73	20	69.51	7.69	21	1.6%	0.54 [-5.56, 6.64]
Boer 2014(1)	77	8	17	72	5	7	1.8%	5.00 [-0.31, 10.31]
Boer 2014(2)	73	9	15	72	5	7	1.7%	1.00 [-4.87, 6.87]
Espinoza-Silva 2019(1)	61.42	14.53	69	61.45	9.09	30	1.9%	-0.03 [-4.76, 4.70]
Faria 2020(1)	70.5	6.87	24	70.8	7.06	13	1.9%	-0.30 [-5.02, 4.42]
Faria 2020(2)	68.2	7.06	26	70.8	7.06	13	1.9%	-2.60 [-7.30, 2.10]
Harder-Lauridsen 2014	60.9	11.24	18	62.6	11.1	18	1.4%	-1.70 [-9.00, 5.60]
Kelly 2004	65	6.32	10	68	9.49	10	1.4%	-3.00 [-10.07, 4.07]
Murphy 2009	70.9	8.4	23	71.5	7.5	12	1.7%	-0.60 [-6.06, 4.86]
Woo 2012	73.45	9.57	10	72.25	5.76	10	1.4%	1.20 [-5.72, 8.12]
**Subtotal (95% CI)**			**232**			**141**	**16.7%**	**0.00 [-1.76, 1.77]**
Heterogeneity: Tau² = 0.00; Chi² = 5.80, df = 9 (P = 0.76); I² = 0%								
Test for overall effect: Z = 0.00 (P = 1.00)								
**2.9.2 Obesity**								
Bharath 2018	78	6.93	20	80	10.73	20	1.7%	-2.00 [-7.60, 3.60]
Bruyndonckx 2015	60	4	27	63	7	21	2.3%	-3.00 [-6.35, 0.35]
Chuensiri 2018(1)	77	6.63	11	74	6.63	5	1.4%	3.00 [-4.01, 10.01]
Chuensiri 2018(2)	71	7.75	15	74	6.63	6	1.5%	-3.00 [-9.60, 3.60]
Espinoza-Silva 2019(2)	62.32	8.66	141	57.41	9.24	34	2.2%	4.91 [1.49, 8.33]
Farpour-Lambert 2009	70.9	5	22	71.8	5.43	22	2.3%	-0.90 [-3.98, 2.18]
Guijun 2009	84	1.28	16	88.5	1.57	10	2.7%	-4.50 [-5.66, -3.34]
Hongling 2012	71	5	30	73	6	30	2.4%	-2.00 [-4.79, 0.79]
Horner 2015(1)	58.75	10.88	27	60.88	6.48	8	1.6%	-2.13 [-8.21, 3.95]
Horner 2015(2)	58.56	7.29	23	60.88	6.48	8	1.8%	-2.32 [-7.71, 3.07]
Jian 2014	75.56	1.45	44	84.81	1.9	44	2.7%	-9.25 [-9.96, -8.54]
Kai 2024	81	1	20	82	1	24	2.7%	-1.00 [-1.59, -0.41]
Kelishadi 2008	74.6	11.81	45	74.4	11.74	47	1.9%	0.20 [-4.61, 5.01]
Kim 2007	81.4	10.85	14	80.8	10.74	12	1.2%	0.60 [-7.72, 8.92]
Kim 2011	69	6.93	12	65	6.32	10	1.7%	4.00 [-1.54, 9.54]
Kim 2020	80.2	1.4	24	81.2	2.8	24	2.7%	-1.00 [-2.25, 0.25]
Luo 2013(1)	57.3	7.35	43	64.09	6.15	34	2.3%	-6.79 [-9.81, -3.77]
Luo 2013(2)	57	5.32	52	60.48	7.32	38	2.4%	-3.48 [-6.22, -0.74]
Meng 2022(1)	67	3	12	71	3.7	6	2.2%	-4.00 [-7.41, -0.59]
Meng 2022(2)	68	4	11	71	3.7	7	2.2%	-3.00 [-6.62, 0.62]
Park 2007	67.5	4.6	19	69.9	6.5	21	2.2%	-2.40 [-5.87, 1.07]
Plavsic 2020	69.2	6.2	22	72.4	6.6	22	2.2%	-3.20 [-6.98, 0.58]
Racil(1) 2016(1)	74	2	17	74	2	7	2.6%	0.00 [-1.76, 1.76]
Racil(1) 2016(2)	75	4	16	74	2	7	2.5%	1.00 [-1.46, 3.46]
Racil 2024(1)	78.61	3.6	12	80.2	3.5	5	2.2%	-1.59 [-5.27, 2.09]
Racil 2024(2)	79.23	3.6	12	80.2	3.5	6	2.2%	-0.97 [-4.43, 2.49]
Salus(1) 2022	71.3	13.1	14	78.4	8.61	14	1.2%	-7.10 [-15.31, 1.11]
Savoye 2007	66.4	9.41	75	67.8	9.17	44	2.2%	-1.40 [-4.85, 2.05]
Savoye 2011	65.3	9.3	45	64.3	9.7	31	2.0%	1.00 [-3.36, 5.36]
Seo 2019	61.44	9.47	26	65.59	6.43	44	2.1%	-4.15 [-8.26, -0.04]
Shalitin 2009	64	12.9	55	63	13.65	55	1.9%	1.00 [-3.96, 5.96]
Shu 2023(1)	66.1	2.4	16	70.3	4.8	8	2.2%	-4.20 [-7.73, -0.67]
Shu 2023(2)	69	3.5	15	70.3	4.8	8	2.2%	-1.30 [-5.07, 2.47]
Son 2017	79.8	6.62	20	82.4	8.9	20	1.9%	-2.60 [-7.46, 2.26]
Vasconcellos 2016	79	14	10	79	4	10	1.1%	0.00 [-9.02, 9.02]
Wong 2008	71.7	7.5	12	70.8	6.7	12	1.7%	0.90 [-4.79, 6.59]
Yu 2020	67.4	6.1	99	68.5	6.5	72	2.6%	-1.10 [-3.02, 0.82]
Yuanyuan 2019(1)	66.89	5.79	10	67.13	5.88	3	1.3%	-0.24 [-7.80, 7.32]
Yuanyuan 2019(2)	61.02	5.76	10	67.13	5.88	3	1.3%	-6.11 [-13.66, 1.44]
Yuanyuan 2019(3)	60.12	5.24	9	67.13	5.88	4	1.5%	-7.01 [-13.71, -0.31]
Zhaogang 2021	66.17	6.27	49	67.54	7.58	49	2.4%	-1.37 [-4.12, 1.38]
**Subtotal (95% CI)**			**1172**			**855**	**83.3%**	**-1.92 [-3.25, -0.60]**
Heterogeneity: Tau² = 13.88; Chi² = 441.40, df = 40 (P < 0.00001); I² = 91%								
Test for overall effect: Z = 2.85 (P = 0.004)								
**Total (95% CI)**			**1404**			**996**	**100.0%**	**-1.60 [-2.80, -0.41]**
Heterogeneity: Tau² = 13.52; Chi² = 459.94, df = 50 (P < 0.00001); I² = 89%								
Test for overall effect: Z = 2.63 (P = 0.009)								
Test for subgroup differences: Chi² = 2.92, df = 1 (P = 0.09), I² = 65.8%								

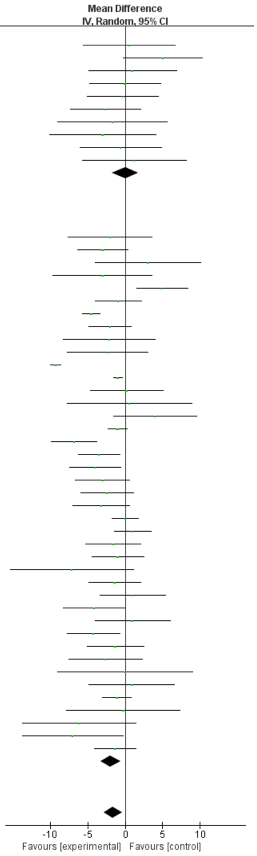

Figure S10.10. Forest plot of subgroup analyses by sex

Study or Subgroup	Experimental Mean	Experimental SD	Experimental Total	Control Mean	Control SD	Control Total	Weight	Mean Difference IV, Random, 95% CI
**2.10.1 Boys**								
Chuensiri 2018(1)	77	6.63	11	74	6.63	5	2.7%	3.00 [-4.01, 10.01]
Chuensiri 2018(2)	71	7.75	15	74	6.63	6	2.9%	-3.00 [-9.60, 3.60]
Cvetković 2018(1)	64	3.94	10	64.57	8.42	7	2.8%	-0.57 [-7.27, 6.13]
Cvetković 2018(2)	66	8	11	64.57	8.42	7	2.5%	1.43 [-6.40, 9.26]
Hongling 2012	71	5	30	73	6	30	4.2%	-2.00 [-4.79, 0.79]
Kim 2007	81.4	10.85	14	80.8	10.74	12	2.3%	0.60 [-7.72, 8.92]
Kim 2011	69	6.93	12	65	6.32	10	3.2%	4.00 [-1.54, 9.54]
Luo 2013(2)	57	5.32	52	60.48	7.32	38	4.3%	-3.48 [-6.22, -0.74]
Meng 2022(1)	67	3	12	71	3.7	6	4.0%	-4.00 [-7.41, -0.59]
Meng 2022(2)	68	4	11	71	3.7	7	4.0%	-3.00 [-6.62, 0.62]
Salus(1) 2022	71.3	13.1	14	78.4	8.61	14	2.4%	-7.10 [-15.31, 1.11]
Wong 2008	71.7	7.5	12	70.8	6.7	12	3.2%	0.90 [-4.79, 6.59]
**Subtotal (95% CI)**			**204**			**154**	**38.5%**	**-1.88 [-3.36, -0.39]**
Heterogeneity: Tau² = 1.04; Chi² = 13.04, df = 11 (P = 0.29); I² = 16%								
Test for overall effect: Z = 2.48 (P = 0.01)								
**2.10.2 Girls**								
Abassi 2022(1)	76.7	4.22	13	77.8	4	6	3.8%	-1.10 [-5.04, 2.84]
Abassi 2022(2)	77.4	4.42	13	77.8	4	6	3.8%	-0.40 [-4.40, 3.60]
Bharath 2018	78	6.93	20	80	10.73	20	3.2%	-2.00 [-7.60, 3.60]
Heidarianpour 2023	77.53	4.35	15	80	4.7	15	4.1%	-2.47 [-5.71, 0.77]
Jian 2014	75.56	1.45	44	84.81	1.9	44	4.7%	-9.25 [-9.96, -8.54]
Kai 2024	81	1	20	82	1	24	4.7%	-1.00 [-1.59, -0.41]
Kim 2020	80.2	1.4	24	81.2	2.8	24	4.6%	-1.00 [-2.25, 0.25]
Luo 2013(1)	57.3	7.35	43	64.09	6.15	34	4.2%	-6.79 [-9.81, -3.77]
Park 2007	67.5	4.6	19	69.9	6.5	21	4.0%	-2.40 [-5.87, 1.07]
Plavsic 2020	69.2	6.2	22	72.4	6.6	22	3.9%	-3.20 [-6.98, 0.58]
Racil(1) 2016(1)	74	2	17	74	2	7	4.5%	0.00 [-1.76, 1.76]
Racil(1) 2016(2)	75	4	16	74	2	7	4.3%	1.00 [-1.46, 3.46]
Racil 2024(1)	78.61	3.6	12	80.2	3.5	5	3.9%	-1.59 [-5.27, 2.09]
Racil 2024(2)	79.23	3.6	12	80.2	3.5	6	4.0%	-0.97 [-4.43, 2.49]
Son 2017	79.8	6.62	20	82.4	8.9	20	3.5%	-2.60 [-7.46, 2.26]
**Subtotal (95% CI)**			**310**			**261**	**61.5%**	**-2.28 [-4.69, 0.12]**
Heterogeneity: Tau² = 19.82; Chi² = 378.75, df = 14 (P < 0.00001); I² = 96%								
Test for overall effect: Z = 1.86 (P = 0.06)								
**Total (95% CI)**			**514**			**415**	**100.0%**	**-1.90 [-3.68, -0.12]**
Heterogeneity: Tau² = 17.42; Chi² = 396.51, df = 26 (P < 0.00001); I² = 93%								
Test for overall effect: Z = 2.09 (P = 0.04)								
Test for subgroup differences: Chi² = 0.08, df = 1 (P = 0.78), I² = 0%								

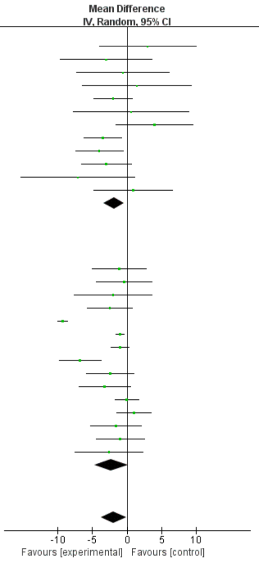

Figure S11. Forest plots of subgroup analyses for TC in children and adolescents with overweight or obesity

Figure S11.1. Forest plot of subgroup analyses by type of exercise

	Experimental			Control				Mean Difference
Study or Subgroup	Mean	SD	Total	Mean	SD	Total	Weight	IV, Random, 95% CI
**2.1.1 Aerobic exercise**								
Ahmadi 2020(1)	4.08	0.62	28	4.18	1.12	9	0.7%	-0.10 [-0.87, 0.67]
Bezerra 2023	4.2	0.74	20	4.54	0.81	21	1.1%	-0.34 [-0.81, 0.13]
Boer 2014(2)	4.24	0.81	15	4.45	0.67	7	0.8%	-0.21 [-0.85, 0.43]
Bowen 2010	4.1	0.89	22	4.05	0.59	18	1.1%	0.05 [-0.41, 0.51]
Davis 2020	4.23	0.95	90	4.19	0.53	85	1.7%	0.04 [-0.19, 0.27]
DeBar 2012	4.12	0.8	74	4.15	0.63	42	1.6%	-0.03 [-0.29, 0.23]
Dias 2018(2)	4.26	0.87	22	4.25	0.81	10	0.9%	0.01 [-0.61, 0.63]
Ghorbanian 2013	4.75	0.66	15	5.87	0.9	15	0.9%	-1.12 [-1.68, -0.56]
Guijun 2009	4.59	0.15	16	5.02	0.12	10	1.9%	-0.43 [-0.53, -0.33]
Gulijianati 2022	4.07	0.62	47	5.93	0.77	46	1.5%	-1.86 [-2.14, -1.58]
Guo 2015	4.5	0.7	23	5	1.2	12	0.7%	-0.50 [-1.24, 0.24]
Hong 2016	3.57	0.74	55	4.06	0.73	55	1.6%	-0.49 [-0.76, -0.22]
Horner 2015(1)	3.87	0.87	27	3.72	0.48	8	1.1%	0.15 [-0.32, 0.62]
Jian 2014	4.2	0.13	44	4.78	0.12	44	1.9%	-0.58 [-0.63, -0.53]
Jianjun 2012	3.86	0.63	20	5.78	0.55	15	1.3%	-1.92 [-2.31, -1.53]
Jun 2006(1)	3.72	0.6	33	3.81	0.75	31	1.4%	-0.09 [-0.42, 0.24]
Jun 2006(2)	3.7	0.56	35	3.87	0.77	32	1.4%	-0.17 [-0.49, 0.15]
Kelishadi 2008	4.18	0.56	45	4.14	0.56	47	1.6%	0.04 [-0.19, 0.27]
Kelly 2004	4.01	0.7	10	3.92	0.82	10	0.8%	0.09 [-0.58, 0.76]
Kim 2007	4.02	0.69	14	4.63	0.9	12	0.9%	-0.61 [-1.23, 0.01]
Lee 2010(2)	4.52	0.89	16	4.46	0.88	9	0.7%	0.06 [-0.66, 0.78]
Lidan 2012	3.93	0.27	30	4.35	0.58	30	1.6%	-0.42 [-0.65, -0.19]
Liqun 2014	4.32	0.78	76	4.44	0.81	97	1.6%	-0.12 [-0.36, 0.12]
Lira 2017(1)	4.41	1.04	26	4.24	0.93	16	0.9%	0.17 [-0.44, 0.78]
Lira 2017(2)	4.3	0.86	25	4.24	0.93	17	1.0%	0.06 [-0.50, 0.62]
Liu 2018	3.52	0.66	30	4	0.88	20	1.2%	-0.48 [-0.93, -0.03]
Luo 2013(1)	3.58	0.74	43	4.33	0.56	34	1.5%	-0.75 [-1.04, -0.46]
Luo 2013(2)	3.64	0.78	52	4.47	0.54	38	1.6%	-0.83 [-1.10, -0.56]
Meng 2022(2)	3.9	0.5	11	4.2	0.6	7	1.0%	-0.30 [-0.83, 0.23]
Monteiro 2015(1)	2.91	0.65	18	3.37	0.86	8	0.8%	-0.46 [-1.13, 0.21]
Morgan 2021	3.87	0.39	15	4.96	0.51	15	1.4%	-1.09 [-1.41, -0.77]
Murphy 2009	4.16	0.82	23	4.11	0.5	12	1.2%	0.05 [-0.39, 0.49]
Park 2007	4.16	0.62	19	4.54	0.74	21	1.2%	-0.38 [-0.80, 0.04]
Plavsic 2020	4.02	0.74	22	3.93	0.6	22	1.3%	0.09 [-0.31, 0.49]
Racil 2013(2)	3.9	0.32	11	3.86	0.16	6	1.7%	0.04 [-0.19, 0.27]
Ramezani 2017(1)	4.02	1.31	15	6.29	1.21	5	0.3%	-2.27 [-3.52, -1.02]
Savoye 2007	4.16	0.84	75	4.34	0.84	44	1.5%	-0.18 [-0.49, 0.13]
Savoye 2011	4.2	0.76	75	4.25	0.81	44	1.5%	-0.05 [-0.34, 0.24]
Saygın 2011	2.48	0.85	20	3.03	0.63	19	1.1%	-0.55 [-1.02, -0.08]
Seo 2012	4.39	2.54	10	4.05	1.79	10	0.1%	0.34 [-1.59, 2.27]
Staiano 2017	4.08	0.38	20	4.21	0.63	18	1.4%	-0.13 [-0.47, 0.21]
Staiano 2018	3.8	0.95	22	4.49	0.98	23	1.0%	-0.69 [-1.25, -0.13]
Sun 2011	3.9	0.6	25	4	0.6	17	1.3%	-0.10 [-0.47, 0.27]
Tsang 2009	4.1	0.83	11	3.81	0.85	8	0.7%	0.29 [-0.48, 1.06]
Woo 2004	4.5	0.8	41	4.5	0.8	41	1.4%	0.00 [-0.35, 0.35]
Woo 2012	4.07	0.83	10	4.76	0.6	10	0.8%	-0.69 [-1.32, -0.06]
Xianbo 2011	0.9	0.4	25	1.2	0.5	17	1.5%	-0.30 [-0.58, -0.02]
Xinhui 2016	3.64	0.84	14	4.11	0.89	14	0.8%	-0.47 [-1.11, 0.17]
Xishao 2009	3.93	0.27	34	4.35	0.58	33	1.7%	-0.42 [-0.64, -0.20]
Xishao 2010	3.93	0.27	100	4.35	0.58	100	1.8%	-0.42 [-0.55, -0.29]
Yanqin 2017	4.32	0.14	35	4.76	0.11	33	1.9%	-0.44 [-0.50, -0.38]
Zhaogang 2021	3	0.78	49	4.01	0.84	49	1.4%	-1.01 [-1.33, -0.69]
**Subtotal (95% CI)**			**1653**			**1366**	**63.9%**	**-0.38 [-0.48, -0.28]**
Heterogeneity: Tau² = 0.09; Chi² = 371.11, df = 51 (P < 0.00001); I² = 86%								
Test for overall effect: Z = 7.50 (P < 0.00001)								
**2.1.2 Anaerobic exercise**								
Boer 2014(1)	4	0.59	17	4.45	0.67	7	0.9%	-0.45 [-1.02, 0.12]
Chuensiri 2018(1)	3.78	0.69	11	4.45	0.77	5	0.6%	-0.67 [-1.46, 0.12]
Chuensiri 2018(2)	3.54	0.7	15	4.45	0.77	6	0.7%	-0.91 [-1.62, -0.20]
Dias 2018(1)	3.79	0.64	19	4.25	0.81	9	0.9%	-0.46 [-1.06, 0.14]
Ke 2024	4.1	0.4	10	4.9	0.9	10	0.9%	-0.80 [-1.41, -0.19]
Lingling 2021	5	0.87	20	5.47	1.29	20	0.8%	-0.47 [-1.15, 0.21]
Meng 2022(1)	4	0.5	12	4.2	0.6	6	1.0%	-0.20 [-0.76, 0.36]
Racil 2013(1)	3.53	0.31	11	3.86	0.16	6	1.7%	-0.33 [-0.55, -0.11]
Salus(2) 2022	3.99	0.37	14	4.64	0.75	14	1.2%	-0.65 [-1.09, -0.21]
Tas 2023	3.78	0.7	31	3.82	0.7	6	0.9%	-0.04 [-0.65, 0.57]
Vasconcellos 2016	3.88	0.65	10	4.69	0.78	10	0.8%	-0.81 [-1.44, -0.18]
**Subtotal (95% CI)**			**170**			**99**	**10.4%**	**-0.45 [-0.59, -0.30]**
Heterogeneity: Tau² = 0.00; Chi² = 8.84, df = 10 (P = 0.55); I² = 0%								
Test for overall effect: Z = 6.08 (P < 0.00001)								
**2.1.3 Resistance exercise**								
Ahmadi 2020(2)	4.08	1.04	29	4.18	1.12	9	0.6%	-0.10 [-0.92, 0.72]
Benson 2008	4.3	1.1	32	4.2	0.7	35	1.2%	0.10 [-0.35, 0.55]
Horner 2015(2)	3.24	0.6	23	3.72	0.48	8	1.2%	-0.48 [-0.89, -0.07]
Ramezani 2017(2)	4.49	1.1	15	6.29	1.21	5	0.3%	-1.80 [-3.00, -0.60]
**Subtotal (95% CI)**			**99**			**57**	**3.4%**	**-0.41 [-0.98, 0.17]**
Heterogeneity: Tau² = 0.22; Chi² = 10.06, df = 3 (P = 0.02); I² = 70%								
Test for overall effect: Z = 1.38 (P = 0.17)								
**2.1.4 Combined aerobic and resistance exercise**								
Ahmadi 2020(3)	4.25	0.93	29	4.18	1.12	9	0.6%	0.07 [-0.74, 0.88]
Bruyndonckx 2015	3.63	0.51	27	3.98	0.5	21	1.5%	-0.35 [-0.64, -0.06]
Chae 2010	4.2	0.76	19	4.51	0.59	19	1.2%	-0.31 [-0.74, 0.12]
Duft 2020	4.32	0.89	18	4.4	0.91	19	0.9%	-0.08 [-0.66, 0.50]
Elmahgoub 2009	4.23	0.9	15	4.37	0.77	15	0.9%	-0.14 [-0.74, 0.46]
Faria 2020(1)	3.6	0.75	24	3.88	0.77	13	1.0%	-0.28 [-0.80, 0.24]
Faria 2020(2)	3.81	0.76	26	3.88	0.77	13	1.0%	-0.07 [-0.58, 0.44]
Farpour-Lambert 2009	4.04	0.79	22	4	0.5	22	1.3%	0.04 [-0.35, 0.43]
Harder-Lauridsen 2014	3.8	0.84	14	4.3	0.46	14	1.1%	-0.50 [-1.00, 0.00]
Hongling 2012	4.1	0.7	30	4.2	1	30	1.2%	-0.10 [-0.54, 0.34]
Kim 2022	4.17	0.82	13	3.74	0.64	13	0.9%	0.43 [-0.14, 1.00]
Lee 2010(1)	4.55	0.48	20	4.46	0.88	9	0.9%	0.09 [-0.52, 0.70]
Liangmei 2015	3.63	0.83	12	4.1	0.89	12	0.8%	-0.47 [-1.16, 0.22]
Lopes 2016	4.31	1.09	17	4.42	0.92	16	0.8%	-0.11 [-0.80, 0.58]
Mohammadi 2019	4.24	0.49	10	4.41	0.36	10	1.3%	-0.17 [-0.55, 0.21]
Monteiro 2015(2)	2.85	0.49	14	3.37	0.86	8	0.8%	-0.52 [-1.17, 0.13]
Ramezani 2017(3)	4.24	1.26	15	6.29	1.21	5	0.3%	-2.05 [-3.29, -0.81]
Shalitin 2009	4.28	0.81	55	4.17	0.88	55	1.5%	0.11 [-0.21, 0.43]
Wen 2008	3.89	0.53	24	4.83	0.8	13	1.1%	-0.94 [-1.42, -0.46]
Wong 2008	4.7	1	12	4.5	0.7	12	0.8%	0.20 [-0.49, 0.89]
Zehsaz 2016	3.91	0.21	16	4.19	0.24	16	1.8%	-0.28 [-0.44, -0.12]
Zehsaz 2017	4.19	1.14	16	4.2	1.08	16	0.7%	-0.01 [-0.78, 0.76]
**Subtotal (95% CI)**			**448**			**360**	**22.4%**	**-0.20 [-0.33, -0.07]**
Heterogeneity: Tau² = 0.04; Chi² = 36.22, df = 21 (P = 0.02); I² = 42%								
Test for overall effect: Z = 2.94 (P = 0.003)								
**Total (95% CI)**			**2370**			**1882**	**100.0%**	**-0.35 [-0.43, -0.27]**
Heterogeneity: Tau² = 0.08; Chi² = 452.93, df = 88 (P < 0.00001); I² = 81%								
Test for overall effect: Z = 8.92 (P < 0.00001)								
Test for subgroup differences: Chi² = 6.99, df = 3 (P = 0.07), I² = 57.1%								

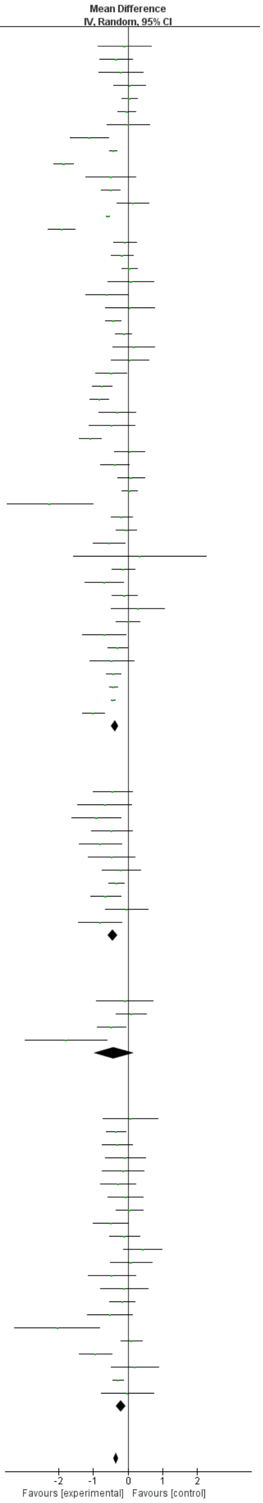

Figure S11.2. Forest plot of subgroup analyses by exercise intensity

	Experimental			Control				Mean Difference
Study or Subgroup	Mean	SD	Total	Mean	SD	Total	Weight	IV, Random, 95% CI
**2.2.1 Moderate-to-low intensity**								
Boer 2014(2)	4.24	0.81	15	4.45	0.67	7	0.8%	-0.21 [-0.85, 0.43]
Bowen 2010	4.1	0.89	22	4.05	0.59	18	1.1%	0.05 [-0.41, 0.51]
Bruyndonckx 2015	3.63	0.51	27	3.98	0.5	21	1.5%	-0.35 [-0.64, -0.06]
Davis 2020	4.23	0.95	90	4.19	0.53	85	1.7%	0.04 [-0.19, 0.27]
DeBar 2012	4.12	0.8	74	4.15	0.63	42	1.6%	-0.03 [-0.29, 0.23]
Dias 2018(2)	4.26	0.87	22	4.25	0.81	10	0.9%	0.01 [-0.61, 0.63]
Elmahgoub 2009	4.23	0.9	15	4.37	0.77	15	0.9%	-0.14 [-0.74, 0.46]
Faria 2020(1)	3.6	0.75	24	3.88	0.77	13	1.0%	-0.28 [-0.80, 0.24]
Farpour-Lambert 2009	4.04	0.79	22	4	0.5	22	1.3%	0.04 [-0.35, 0.43]
Ghorbanian 2013	4.75	0.66	15	5.87	0.9	15	0.9%	-1.12 [-1.68, -0.56]
Guijun 2009	4.59	0.15	16	5.02	0.12	10	1.9%	-0.43 [-0.53, -0.33]
Guo 2015	4.5	0.7	23	5	1.2	12	0.7%	-0.50 [-1.24, 0.24]
Harder-Lauridsen 2014	3.8	0.84	14	4.3	0.46	14	1.1%	-0.50 [-1.00, 0.00]
Hong 2016	3.57	0.74	55	4.06	0.73	55	1.6%	-0.49 [-0.76, -0.22]
Horner 2015(1)	3.87	0.87	27	3.72	0.48	8	1.1%	0.15 [-0.32, 0.62]
Jian 2014	4.2	0.13	44	4.78	0.12	44	1.9%	-0.58 [-0.63, -0.53]
Jianjun 2012	3.86	0.63	20	5.78	0.55	15	1.3%	-1.92 [-2.31, -1.53]
Jun 2006(1)	3.72	0.6	33	3.81	0.75	31	1.4%	-0.09 [-0.42, 0.24]
Jun 2006(2)	3.7	0.56	35	3.87	0.77	32	1.4%	-0.17 [-0.49, 0.15]
Kelishadi 2008	4.18	0.56	45	4.14	0.56	47	1.6%	0.04 [-0.19, 0.27]
Kelly 2004	4.01	0.7	10	3.92	0.82	10	0.8%	0.09 [-0.58, 0.76]
Kim 2007	4.02	0.69	14	4.63	0.9	12	0.9%	-0.61 [-1.23, 0.01]
Kim 2022	4.17	0.82	13	3.74	0.64	13	0.9%	0.43 [-0.14, 1.00]
Lee 2010(1)	4.55	0.48	20	4.46	0.88	9	0.9%	0.09 [-0.52, 0.70]
Lee 2010(2)	4.52	0.89	16	4.46	0.88	9	0.7%	0.06 [-0.66, 0.78]
Liangmei 2015	3.63	0.83	12	4.1	0.89	12	0.8%	-0.47 [-1.16, 0.22]
Lidan 2012	3.93	0.27	30	4.35	0.58	30	1.6%	-0.42 [-0.65, -0.19]
Liqun 2014	4.32	0.78	76	4.44	0.81	97	1.6%	-0.12 [-0.36, 0.12]
Lira 2017(2)	4.3	0.86	25	4.24	0.93	17	1.0%	0.06 [-0.50, 0.62]
Liu 2018	3.52	0.66	30	4	0.88	20	1.2%	-0.48 [-0.93, -0.03]
Luo 2013(1)	3.58	0.74	43	4.33	0.56	34	1.5%	-0.75 [-1.04, -0.46]
Luo 2013(2)	3.64	0.78	52	4.47	0.54	38	1.6%	-0.83 [-1.10, -0.56]
Meng 2022(2)	3.9	0.5	11	4.2	0.6	7	1.0%	-0.30 [-0.83, 0.23]
Mohammadi 2019	4.24	0.49	10	4.41	0.36	10	1.3%	-0.17 [-0.55, 0.21]
Morgan 2021	3.87	0.39	15	4.96	0.51	15	1.4%	-1.09 [-1.41, -0.77]
Murphy 2009	4.16	0.82	23	4.11	0.5	12	1.2%	0.05 [-0.39, 0.49]
Park 2007	4.16	0.62	19	4.54	0.74	21	1.2%	-0.38 [-0.80, 0.04]
Racil 2013(2)	3.9	0.32	11	3.86	0.16	6	1.7%	0.04 [-0.19, 0.27]
Ramezani 2017(1)	4.02	1.31	15	6.29	1.21	5	0.3%	-2.27 [-3.52, -1.02]
Ramezani 2017(2)	4.49	1.1	15	6.29	1.21	5	0.3%	-1.80 [-3.00, -0.60]
Ramezani 2017(3)	4.24	1.26	15	6.29	1.21	5	0.3%	-2.05 [-3.29, -0.81]
Saygın 2011	2.48	0.85	20	3.03	0.63	19	1.1%	-0.55 [-1.02, -0.08]
Seo 2012	4.39	2.54	10	4.05	1.79	10	0.1%	0.34 [-1.59, 2.27]
Shalitin 2009	4.28	0.81	55	4.17	0.88	55	1.5%	0.11 [-0.21, 0.43]
Staiano 2017	4.08	0.38	20	4.21	0.63	18	1.4%	-0.13 [-0.47, 0.21]
Staiano 2018	3.8	0.95	22	4.49	0.98	23	1.0%	-0.69 [-1.25, -0.13]
Sun 2011	3.9	0.6	25	4	0.6	17	1.3%	-0.10 [-0.47, 0.27]
Tsang 2009	4.1	0.83	11	3.81	0.85	8	0.7%	0.29 [-0.48, 1.06]
Wen 2008	3.89	0.53	24	4.83	0.8	13	1.1%	-0.94 [-1.42, -0.46]
Woo 2004	4.5	0.8	41	4.5	0.8	41	1.4%	0.00 [-0.35, 0.35]
Woo 2012	4.07	0.83	10	4.76	0.6	10	0.8%	-0.69 [-1.32, -0.06]
Xianbo 2011	0.9	0.4	25	1.2	0.5	17	1.5%	-0.30 [-0.58, -0.02]
Xinhui 2016	3.64	0.84	14	4.11	0.89	14	0.8%	-0.47 [-1.11, 0.17]
Xishao 2009	3.93	0.27	34	4.35	0.58	33	1.7%	-0.42 [-0.64, -0.20]
Xishao 2010	3.93	0.27	100	4.35	0.58	100	1.8%	-0.42 [-0.55, -0.29]
Yanqin 2017	4.32	0.14	35	4.76	0.11	33	1.9%	-0.44 [-0.50, -0.38]
Zehsaz 2016	3.91	0.21	16	4.19	0.24	16	1.8%	-0.28 [-0.44, -0.12]
Zhaogang 2021	3	0.78	49	4.01	0.84	49	1.4%	-1.01 [-1.33, -0.69]
**Subtotal (95% CI)**			**1624**			**1379**	**69.4%**	**-0.35 [-0.44, -0.27]**
Heterogeneity: Tau² = 0.06; Chi² = 308.18, df = 57 (P < 0.00001); I² = 82%								
Test for overall effect: Z = 8.10 (P < 0.00001)								
**2.2.2 High intensity**								
Ahmadi 2020(1)	4.08	0.62	28	4.18	1.12	9	0.7%	-0.10 [-0.87, 0.67]
Ahmadi 2020(2)	4.08	1.04	29	4.18	1.12	9	0.6%	-0.10 [-0.92, 0.72]
Ahmadi 2020(3)	4.25	0.93	29	4.18	1.12	9	0.6%	0.07 [-0.74, 0.88]
Benson 2008	4.3	1.1	32	4.2	0.7	35	1.2%	0.10 [-0.35, 0.55]
Bezerra 2023	4.2	0.74	20	4.54	0.81	21	1.1%	-0.34 [-0.81, 0.13]
Boer 2014(1)	4	0.59	17	4.45	0.67	7	0.9%	-0.45 [-1.02, 0.12]
Chae 2010	4.2	0.76	19	4.51	0.59	19	1.2%	-0.31 [-0.74, 0.12]
Chuensiri 2018(1)	3.78	0.69	11	4.45	0.77	5	0.6%	-0.67 [-1.46, 0.12]
Chuensiri 2018(2)	3.54	0.7	15	4.45	0.77	6	0.7%	-0.91 [-1.62, -0.20]
Dias 2018(1)	3.79	0.64	19	4.25	0.81	9	0.9%	-0.46 [-1.06, 0.14]
Duft 2020	4.32	0.89	18	4.4	0.91	19	0.9%	-0.08 [-0.66, 0.50]
Faria 2020(2)	3.81	0.76	26	3.88	0.77	13	1.0%	-0.07 [-0.58, 0.44]
Gulijianati 2022	4.07	0.62	47	5.93	0.77	46	1.5%	-1.86 [-2.14, -1.58]
Hongling 2012	4.1	0.7	30	4.2	1	30	1.2%	-0.10 [-0.54, 0.34]
Horner 2015(2)	3.24	0.6	23	3.72	0.48	8	1.2%	-0.48 [-0.89, -0.07]
Ke 2024	4.1	0.4	10	4.9	0.9	10	0.9%	-0.80 [-1.41, -0.19]
Lingling 2021	5	0.87	20	5.47	1.29	20	0.8%	-0.47 [-1.15, 0.21]
Lira 2017(1)	4.41	1.04	26	4.24	0.93	16	0.9%	0.17 [-0.44, 0.78]
Lopes 2016	4.31	1.09	17	4.42	0.92	16	0.8%	-0.11 [-0.80, 0.58]
Meng 2022(1)	4	0.5	12	4.2	0.6	6	1.0%	-0.20 [-0.76, 0.36]
Monteiro 2015(1)	2.91	0.65	18	3.37	0.86	8	0.8%	-0.46 [-1.13, 0.21]
Monteiro 2015(2)	2.85	0.49	14	3.37	0.86	8	0.8%	-0.52 [-1.17, 0.13]
Plavsic 2020	4.02	0.74	22	3.93	0.6	22	1.3%	0.09 [-0.31, 0.49]
Racil 2013(1)	3.53	0.31	11	3.86	0.16	6	1.7%	-0.33 [-0.55, -0.11]
Salus(2) 2022	3.99	0.37	14	4.64	0.75	14	1.2%	-0.65 [-1.09, -0.21]
Savoye 2007	4.16	0.84	75	4.34	0.84	44	1.5%	-0.18 [-0.49, 0.13]
Savoye 2011	4.2	0.76	75	4.25	0.81	44	1.5%	-0.05 [-0.34, 0.24]
Tas 2023	3.78	0.7	31	3.82	0.7	6	0.9%	-0.04 [-0.65, 0.57]
Vasconcellos 2016	3.88	0.65	10	4.69	0.78	10	0.8%	-0.81 [-1.44, -0.18]
Wong 2008	4.7	1	12	4.5	0.7	12	0.8%	0.20 [-0.49, 0.89]
Zehsaz 2017	4.19	1.14	16	4.2	1.08	16	0.7%	-0.01 [-0.78, 0.76]
**Subtotal (95% CI)**			**746**			**503**	**30.6%**	**-0.33 [-0.53, -0.13]**
Heterogeneity: Tau² = 0.23; Chi² = 143.88, df = 30 (P < 0.00001); I² = 79%								
Test for overall effect: Z = 3.31 (P = 0.0009)								
**Total (95% CI)**			**2370**			**1882**	**100.0%**	**-0.35 [-0.43, -0.27]**
Heterogeneity: Tau² = 0.08; Chi² = 452.93, df = 88 (P < 0.00001); I² = 81%								
Test for overall effect: Z = 8.92 (P < 0.00001)								
Test for subgroup differences: Chi² = 0.05, df = 1 (P = 0.83), I² = 0%								

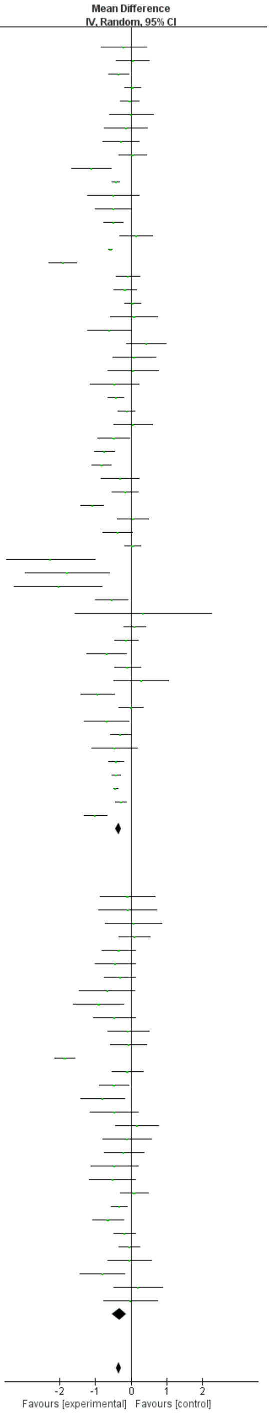

Figure S11.3. Forest plot of subgroup analyses by duration per session

Study or Subgroup	Experimental Mean	Experimental SD	Experimental Total	Control Mean	Control SD	Control Total	Weight	Mean Difference IV, Random, 95% CI
**2.3.1 <50 minutes per session**								
Boer 2014(1)	4	0.59	17	4.45	0.67	7	0.9%	-0.45 [-1.02, 0.12]
Boer 2014(2)	4.24	0.81	15	4.45	0.67	7	0.8%	-0.21 [-0.85, 0.43]
Bruyndonckx 2015	3.63	0.51	27	3.98	0.5	21	1.5%	-0.35 [-0.64, -0.06]
Chuensiri 2018(1)	3.78	0.69	11	4.45	0.77	5	0.6%	-0.67 [-1.46, 0.12]
Chuensiri 2018(2)	3.54	0.7	15	4.45	0.77	6	0.7%	-0.91 [-1.62, -0.20]
Davis 2020	4.23	0.95	90	4.19	0.53	85	1.7%	0.04 [-0.19, 0.27]
DeBar 2012	4.12	0.8	74	4.15	0.63	42	1.6%	-0.03 [-0.29, 0.23]
Dias 2018(1)	3.79	0.64	19	4.25	0.81	9	0.9%	-0.46 [-1.06, 0.14]
Dias 2018(2)	4.26	0.87	22	4.25	0.81	10	0.9%	0.01 [-0.61, 0.63]
Faria 2020(2)	3.81	0.76	26	3.88	0.77	13	1.0%	-0.07 [-0.58, 0.44]
Ghorbanian 2013	4.75	0.66	15	5.87	0.9	15	0.9%	-1.12 [-1.68, -0.56]
Gulijianati 2022	4.07	0.62	47	5.93	0.77	46	1.5%	-1.86 [-2.14, -1.58]
Kelishadi 2008	4.18	0.56	45	4.14	0.56	47	1.6%	0.04 [-0.19, 0.27]
Kelly 2004	4.01	0.7	10	3.92	0.82	10	0.8%	0.09 [-0.58, 0.76]
Kim 2007	4.02	0.69	14	4.63	0.9	12	0.9%	-0.61 [-1.23, 0.01]
Lingling 2021	5	0.87	20	5.47	1.29	20	0.8%	-0.47 [-1.15, 0.21]
Liqun 2014	4.32	0.78	76	4.44	0.81	97	1.6%	-0.12 [-0.36, 0.12]
Lira 2017(1)	4.41	1.04	26	4.24	0.93	16	0.9%	0.17 [-0.44, 0.78]
Meng 2022(1)	4	0.5	12	4.2	0.6	6	1.0%	-0.20 [-0.76, 0.36]
Meng 2022(2)	3.9	0.5	11	4.2	0.6	7	1.0%	-0.30 [-0.83, 0.23]
Murphy 2009	4.16	0.82	23	4.11	0.5	12	1.2%	0.05 [-0.39, 0.49]
Park 2007	4.16	0.62	19	4.54	0.74	21	1.2%	-0.38 [-0.80, 0.04]
Plavsic 2020	4.02	0.74	22	3.93	0.6	22	1.3%	0.09 [-0.31, 0.49]
Racil 2013(1)	3.53	0.31	11	3.86	0.16	6	1.7%	-0.33 [-0.55, -0.11]
Racil 2013(2)	3.9	0.32	11	3.86	0.16	6	1.7%	0.04 [-0.19, 0.27]
Salus(2) 2022	3.99	0.37	14	4.64	0.75	14	1.2%	-0.65 [-1.09, -0.21]
Staiano 2018	3.8	0.95	22	4.49	0.98	23	1.0%	-0.69 [-1.25, -0.13]
Tas 2023	3.78	0.7	31	3.82	0.7	6	0.9%	-0.04 [-0.65, 0.57]
Yanqin 2017	4.32	0.14	35	4.76	0.11	33	1.9%	-0.44 [-0.50, -0.38]
Zhaogang 2021	3	0.78	49	4.01	0.84	49	1.4%	-1.01 [-1.33, -0.69]
**Subtotal (95% CI)**			**829**			**673**	**35.1%**	**-0.36 [-0.51, -0.20]**
Heterogeneity: Tau² = 0.14; Chi² = 204.95, df = 29 (P < 0.00001); I² = 86%								
Test for overall effect: Z = 4.39 (P < 0.0001)								
**2.3.2 ≥50 minutes per session**								
Ahmadi 2020(1)	4.08	0.62	28	4.18	1.12	9	0.7%	-0.10 [-0.87, 0.67]
Ahmadi 2020(2)	4.08	1.04	29	4.18	1.12	9	0.6%	-0.10 [-0.92, 0.72]
Ahmadi 2020(3)	4.25	0.93	29	4.18	1.12	9	0.6%	0.07 [-0.74, 0.88]
Benson 2008	4.3	1.1	32	4.2	0.7	35	1.2%	0.10 [-0.35, 0.55]
Bezerra 2023	4.2	0.74	20	4.54	0.81	21	1.1%	-0.34 [-0.81, 0.13]
Bowen 2010	4.1	0.89	22	4.05	0.59	18	1.1%	0.05 [-0.41, 0.51]
Chae 2010	4.2	0.76	19	4.51	0.59	19	1.2%	-0.31 [-0.74, 0.12]
Duft 2020	4.32	0.89	18	4.4	0.91	19	0.9%	-0.08 [-0.66, 0.50]
Elmahgoub 2009	4.23	0.9	15	4.37	0.77	15	0.9%	-0.14 [-0.74, 0.46]
Faria 2020(1)	3.6	0.75	24	3.88	0.77	13	1.0%	-0.28 [-0.80, 0.24]
Farpour-Lambert 2009	4.04	0.79	22	4	0.5	22	1.3%	0.04 [-0.35, 0.43]
Guijun 2009	4.59	0.15	16	5.02	0.12	10	1.9%	-0.43 [-0.53, -0.33]
Guo 2015	4.5	0.7	23	5	1.2	12	0.7%	-0.50 [-1.24, 0.24]
Harder-Lauridsen 2014	3.8	0.84	14	4.3	0.46	14	1.1%	-0.50 [-1.00, 0.00]
Hong 2016	3.57	0.74	55	4.06	0.73	55	1.6%	-0.49 [-0.76, -0.22]
Hongling 2012	4.1	0.7	30	4.2	1	30	1.2%	-0.10 [-0.54, 0.34]
Horner 2015(1)	3.87	0.87	27	3.72	0.48	8	1.1%	0.15 [-0.32, 0.62]
Horner 2015(2)	3.24	0.6	23	3.72	0.48	8	1.2%	-0.48 [-0.89, -0.07]
Jian 2014	4.2	0.13	44	4.78	0.12	44	1.9%	-0.58 [-0.63, -0.53]
Jianjun 2012	3.86	0.63	20	5.78	0.55	15	1.3%	-1.92 [-2.31, -1.53]
Jun 2006(1)	3.72	0.6	33	3.81	0.75	31	1.4%	-0.09 [-0.42, 0.24]
Jun 2006(2)	3.7	0.56	35	3.87	0.77	32	1.4%	-0.17 [-0.49, 0.15]
Ke 2024	4.1	0.4	10	4.9	0.9	10	0.9%	-0.80 [-1.41, -0.19]
Kim 2022	4.17	0.82	13	3.74	0.64	13	0.9%	0.43 [-0.14, 1.00]
Lee 2010(1)	4.55	0.48	20	4.46	0.88	9	0.9%	0.09 [-0.52, 0.70]
Lee 2010(2)	4.52	0.89	16	4.46	0.88	9	0.7%	0.06 [-0.66, 0.78]
Liangmei 2015	3.63	0.83	12	4.1	0.89	12	0.8%	-0.47 [-1.16, 0.22]
Lidan 2012	3.93	0.27	30	4.35	0.58	30	1.6%	-0.42 [-0.65, -0.19]
Lira 2017(2)	4.3	0.86	25	4.24	0.93	17	1.0%	0.06 [-0.50, 0.62]
Liu 2018	3.52	0.66	30	4	0.88	20	1.2%	-0.48 [-0.93, -0.03]
Lopes 2016	4.31	1.09	17	4.42	0.92	16	0.8%	-0.11 [-0.80, 0.58]
Luo 2013(1)	3.58	0.74	43	4.33	0.56	34	1.5%	-0.75 [-1.04, -0.46]
Luo 2013(2)	3.64	0.78	52	4.47	0.54	38	1.6%	-0.83 [-1.10, -0.56]
Mohammadi 2019	4.24	0.49	10	4.41	0.36	10	1.3%	-0.17 [-0.55, 0.21]
Monteiro 2015(1)	2.91	0.65	18	3.37	0.86	8	0.8%	-0.46 [-1.13, 0.21]
Monteiro 2015(2)	2.85	0.49	14	3.37	0.86	8	0.8%	-0.52 [-1.17, 0.13]
Morgan 2021	3.87	0.39	15	4.96	0.51	15	1.4%	-1.09 [-1.41, -0.77]
Ramezani 2017(1)	4.02	1.31	15	6.29	1.21	5	0.3%	-2.27 [-3.52, -1.02]
Ramezani 2017(2)	4.49	1.1	15	6.29	1.21	5	0.3%	-1.80 [-3.00, -0.60]
Ramezani 2017(3)	4.24	1.26	15	6.29	1.21	5	0.3%	-2.05 [-3.29, -0.81]
Savoye 2007	4.16	0.84	75	4.34	0.84	44	1.5%	-0.18 [-0.49, 0.13]
Savoye 2011	4.2	0.76	75	4.25	0.81	44	1.5%	-0.05 [-0.34, 0.24]
Saygın 2011	2.48	0.85	20	3.03	0.63	19	1.1%	-0.55 [-1.02, -0.08]
Seo 2012	4.39	2.54	10	4.05	1.79	10	0.1%	0.34 [-1.59, 2.27]
Shalitin 2009	4.28	0.81	55	4.17	0.88	55	1.5%	0.11 [-0.21, 0.43]
Staiano 2017	4.08	0.38	20	4.21	0.63	18	1.4%	-0.13 [-0.47, 0.21]
Sun 2011	3.9	0.6	25	4	0.6	17	1.3%	-0.10 [-0.47, 0.27]
Tsang 2009	4.1	0.83	11	3.81	0.85	8	0.7%	0.29 [-0.48, 1.06]
Vasconcellos 2016	3.88	0.65	10	4.69	0.78	10	0.8%	-0.81 [-1.44, -0.18]
Wen 2008	3.89	0.53	24	4.83	0.8	13	1.1%	-0.94 [-1.42, -0.46]
Wong 2008	4.7	1	12	4.5	0.7	12	0.8%	0.20 [-0.49, 0.89]
Woo 2004	4.5	0.8	41	4.5	0.8	41	1.4%	0.00 [-0.35, 0.35]
Woo 2012	4.07	0.83	10	4.76	0.6	10	0.8%	-0.69 [-1.32, -0.06]
Xianbo 2011	0.9	0.4	25	1.2	0.5	17	1.5%	-0.30 [-0.58, -0.02]
Xinhui 2016	3.64	0.84	14	4.11	0.89	14	0.8%	-0.47 [-1.11, 0.17]
Xishao 2009	3.93	0.27	34	4.35	0.58	33	1.7%	-0.42 [-0.64, -0.20]
Xishao 2010	3.93	0.27	100	4.35	0.58	100	1.8%	-0.42 [-0.55, -0.29]
Zehsaz 2016	3.91	0.21	16	4.19	0.24	16	1.8%	-0.28 [-0.44, -0.12]
Zehsaz 2017	4.19	1.14	16	4.2	1.08	16	0.7%	-0.01 [-0.78, 0.76]
**Subtotal (95% CI)**			**1541**			**1209**	**64.9%**	**-0.35 [-0.44, -0.26]**
Heterogeneity: Tau² = 0.07; Chi² = 240.94, df = 58 (P < 0.00001); I² = 76%								
Test for overall effect: Z = 7.52 (P < 0.00001)								
**Total (95% CI)**			**2370**			**1882**	**100.0%**	**-0.35 [-0.43, -0.27]**
Heterogeneity: Tau² = 0.08; Chi² = 452.93, df = 88 (P < 0.00001); I² = 81%								
Test for overall effect: Z = 8.92 (P < 0.00001)								
Test for subgroup differences: Chi² = 0.00, df = 1 (P = 0.95), I² = 0%								

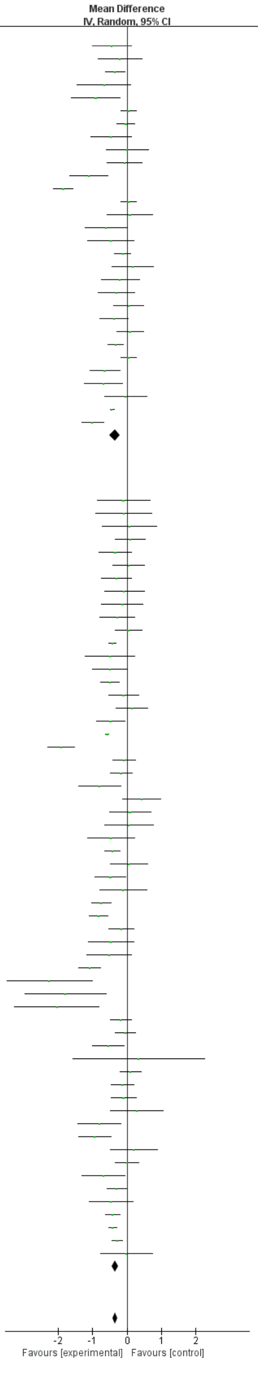

Figure S11.4. Forest plot of subgroup analyses by exercise frequency

Study or Subgroup	Experimental Mean	Experimental SD	Experimental Total	Control Mean	Control SD	Control Total	Weight	Mean Difference IV, Random, 95% CI
**2.4.1 <3 sessions per week**								
Benson 2008	4.3	1.1	32	4.2	0.7	35	1.2%	0.10 [-0.35, 0.55]
Boer 2014(1)	4	0.59	17	4.45	0.67	7	0.9%	-0.45 [-1.02, 0.12]
Boer 2014(2)	4.24	0.81	15	4.45	0.67	7	0.8%	-0.21 [-0.85, 0.43]
Chae 2010	4.2	0.76	19	4.51	0.59	19	1.2%	-0.31 [-0.74, 0.12]
Faria 2020(1)	3.6	0.75	24	3.88	0.77	13	1.0%	-0.28 [-0.80, 0.24]
Faria 2020(2)	3.81	0.76	26	3.88	0.77	13	1.0%	-0.07 [-0.58, 0.44]
Harder-Lauridsen 2014	3.8	0.84	14	4.3	0.46	14	1.1%	-0.50 [-1.00, 0.00]
Hongling 2012	4.1	0.7	30	4.2	1	30	1.2%	-0.10 [-0.54, 0.34]
Plavsic 2020	4.02	0.74	22	3.93	0.6	22	1.3%	0.09 [-0.31, 0.49]
Savoye 2007	4.16	0.84	75	4.34	0.84	44	1.5%	-0.18 [-0.49, 0.13]
Savoye 2011	4.2	0.76	75	4.25	0.81	44	1.5%	-0.05 [-0.34, 0.24]
Wong 2008	4.7	1	12	4.5	0.7	12	0.8%	0.20 [-0.49, 0.89]
Woo 2004	4.5	0.8	41	4.5	0.8	41	1.4%	0.00 [-0.35, 0.35]
Zehsaz 2016	3.91	0.21	16	4.19	0.24	16	1.8%	-0.28 [-0.44, -0.12]
Zehsaz 2017	4.19	1.14	16	4.2	1.08	16	0.7%	-0.01 [-0.78, 0.76]
**Subtotal (95% CI)**			**434**			**333**	**17.3%**	**-0.17 [-0.27, -0.08]**
Heterogeneity: Tau² = 0.00; Chi² = 11.20, df = 14 (P = 0.67); I² = 0%								
Test for overall effect: Z = 3.64 (P = 0.0003)								
**2.4.2 ≥3 sessions per week**								
Ahmadi 2020(1)	4.08	0.62	28	4.18	1.12	9	0.7%	-0.10 [-0.87, 0.67]
Ahmadi 2020(2)	4.08	1.04	29	4.18	1.12	9	0.6%	-0.10 [-0.92, 0.72]
Ahmadi 2020(3)	4.25	0.93	29	4.18	1.12	9	0.6%	0.07 [-0.74, 0.88]
Bezerra 2023	4.2	0.74	20	4.54	0.81	21	1.1%	-0.34 [-0.81, 0.13]
Bowen 2010	4.1	0.89	22	4.05	0.59	18	1.1%	0.05 [-0.41, 0.51]
Bruyndonckx 2015	3.63	0.51	27	3.98	0.5	21	1.5%	-0.35 [-0.64, -0.06]
Chuensiri 2018(1)	3.78	0.69	11	4.45	0.77	5	0.6%	-0.67 [-1.46, 0.12]
Chuensiri 2018(2)	3.54	0.7	15	4.45	0.77	6	0.7%	-0.91 [-1.62, -0.20]
Davis 2020	4.23	0.95	90	4.19	0.53	85	1.7%	0.04 [-0.19, 0.27]
DeBar 2012	4.12	0.8	74	4.15	0.63	42	1.6%	-0.03 [-0.29, 0.23]
Dias 2018(1)	3.79	0.64	19	4.25	0.81	9	0.9%	-0.46 [-1.06, 0.14]
Dias 2018(2)	4.26	0.87	22	4.25	0.81	10	0.9%	0.01 [-0.61, 0.63]
Duft 2020	4.32	0.89	18	4.4	0.91	19	0.9%	-0.08 [-0.66, 0.50]
Elmahgoub 2009	4.23	0.9	15	4.37	0.77	15	0.9%	-0.14 [-0.74, 0.46]
Farpour-Lambert 2009	4.04	0.79	22	4	0.5	22	1.3%	0.04 [-0.35, 0.43]
Ghorbanian 2013	4.75	0.66	15	5.87	0.9	15	0.9%	-1.12 [-1.68, -0.56]
Guijun 2009	4.59	0.15	16	5.02	0.12	10	1.9%	-0.43 [-0.53, -0.33]
Gulijianati 2022	4.07	0.62	47	5.93	0.77	46	1.5%	-1.86 [-2.14, -1.58]
Guo 2015	4.5	0.7	23	5	1.2	12	0.7%	-0.50 [-1.24, 0.24]
Hong 2016	3.57	0.74	55	4.06	0.73	55	1.6%	-0.49 [-0.76, -0.22]
Horner 2015(1)	3.87	0.87	27	3.72	0.48	8	1.1%	0.15 [-0.32, 0.62]
Horner 2015(2)	3.24	0.6	23	3.72	0.48	8	1.2%	-0.48 [-0.89, -0.07]
Jian 2014	4.2	0.13	44	4.78	0.12	44	1.9%	-0.58 [-0.63, -0.53]
Jianjun 2012	3.86	0.63	20	5.78	0.55	15	1.3%	-1.92 [-2.31, -1.53]
Jun 2006(1)	3.72	0.6	33	3.81	0.75	31	1.4%	-0.09 [-0.42, 0.24]
Jun 2006(2)	3.7	0.56	35	3.87	0.77	32	1.4%	-0.17 [-0.49, 0.15]
Ke 2024	4.1	0.4	10	4.9	0.9	10	0.9%	-0.80 [-1.41, -0.19]
Kelishadi 2008	4.18	0.56	45	4.14	0.56	47	1.6%	0.04 [-0.19, 0.27]
Kelly 2004	4.01	0.7	10	3.92	0.82	10	0.8%	0.09 [-0.58, 0.76]
Kim 2007	4.02	0.69	14	4.63	0.9	12	0.9%	-0.61 [-1.23, 0.01]
Kim 2022	4.17	0.82	13	3.74	0.64	13	0.9%	0.43 [-0.14, 1.00]
Lee 2010(1)	4.55	0.48	20	4.46	0.88	9	0.9%	0.09 [-0.52, 0.70]
Lee 2010(2)	4.52	0.89	16	4.46	0.88	9	0.7%	0.06 [-0.66, 0.78]
Liangmei 2015	3.63	0.83	12	4.1	0.89	12	0.8%	-0.47 [-1.16, 0.22]
Lidan 2012	3.93	0.27	30	4.35	0.58	30	1.6%	-0.42 [-0.65, -0.19]
Lingling 2021	5	0.87	20	5.47	1.29	20	0.8%	-0.47 [-1.15, 0.21]
Liqun 2014	4.32	0.78	76	4.44	0.81	97	1.6%	-0.12 [-0.36, 0.12]
Lira 2017(1)	4.41	1.04	26	4.24	0.93	16	0.9%	0.17 [-0.44, 0.78]
Lira 2017(2)	4.3	0.86	25	4.24	0.93	17	1.0%	0.06 [-0.50, 0.62]
Liu 2018	3.52	0.66	30	4	0.88	20	1.2%	-0.48 [-0.93, -0.03]
Lopes 2016	4.31	1.09	17	4.42	0.92	16	0.8%	-0.11 [-0.80, 0.58]
Luo 2013(1)	3.58	0.74	43	4.33	0.56	34	1.5%	-0.75 [-1.04, -0.46]
Luo 2013(2)	3.64	0.78	52	4.47	0.54	38	1.6%	-0.83 [-1.10, -0.56]
Meng 2022(1)	4	0.5	12	4.2	0.6	6	1.0%	-0.20 [-0.76, 0.36]
Meng 2022(2)	3.9	0.5	11	4.2	0.6	7	1.0%	-0.30 [-0.83, 0.23]
Mohammadi 2019	4.24	0.49	10	4.41	0.36	10	1.3%	-0.17 [-0.55, 0.21]
Monteiro 2015(1)	2.91	0.65	18	3.37	0.86	8	0.8%	-0.46 [-1.13, 0.21]
Monteiro 2015(2)	2.85	0.49	14	3.37	0.86	8	0.8%	-0.52 [-1.17, 0.13]
Morgan 2021	3.87	0.39	15	4.96	0.51	15	1.4%	-1.09 [-1.41, -0.77]
Murphy 2009	4.16	0.82	23	4.11	0.5	12	1.2%	0.05 [-0.39, 0.49]
Park 2007	4.16	0.62	19	4.54	0.74	21	1.2%	-0.38 [-0.80, 0.04]
Racil 2013(1)	3.53	0.31	11	3.86	0.16	6	1.7%	-0.33 [-0.55, -0.11]
Racil 2013(2)	3.9	0.32	11	3.86	0.16	6	1.7%	0.04 [-0.19, 0.27]
Ramezani 2017(1)	4.02	1.31	15	6.29	1.21	5	0.3%	-2.27 [-3.52, -1.02]
Ramezani 2017(2)	4.49	1.1	15	6.29	1.21	5	0.3%	-1.80 [-3.00, -0.60]
Ramezani 2017(3)	4.24	1.26	15	6.29	1.21	5	0.3%	-2.05 [-3.29, -0.81]
Salus(2) 2022	3.99	0.37	14	4.64	0.75	14	1.2%	-0.65 [-1.09, -0.21]
Saygın 2011	2.48	0.85	20	3.03	0.63	19	1.1%	-0.55 [-1.02, -0.08]
Seo 2012	4.39	2.54	10	4.05	1.79	10	0.1%	0.34 [-1.59, 2.27]
Shalitin 2009	4.28	0.81	55	4.17	0.88	55	1.5%	0.11 [-0.21, 0.43]
Staiano 2017	4.08	0.38	20	4.21	0.63	18	1.4%	-0.13 [-0.47, 0.21]
Staiano 2018	3.8	0.95	22	4.49	0.98	23	1.0%	-0.69 [-1.25, -0.13]
Sun 2011	3.9	0.6	25	4	0.6	17	1.3%	-0.10 [-0.47, 0.27]
Tas 2023	3.78	0.7	31	3.82	0.7	6	0.9%	-0.04 [-0.65, 0.57]
Tsang 2009	4.1	0.83	11	3.81	0.85	8	0.7%	0.29 [-0.48, 1.06]
Vasconcellos 2016	3.88	0.65	10	4.69	0.78	10	0.8%	-0.81 [-1.44, -0.18]
Wen 2008	3.89	0.53	24	4.83	0.8	13	1.1%	-0.94 [-1.42, -0.46]
Woo 2012	4.07	0.83	10	4.76	0.6	10	0.8%	-0.69 [-1.32, -0.06]
Xianbo 2011	0.9	0.4	25	1.2	0.5	17	1.5%	-0.30 [-0.58, -0.02]
Xinhui 2016	3.64	0.84	14	4.11	0.89	14	0.8%	-0.47 [-1.11, 0.17]
Xishao 2009	3.93	0.27	34	4.35	0.58	33	1.7%	-0.42 [-0.64, -0.20]
Xishao 2010	3.93	0.27	100	4.35	0.58	100	1.8%	-0.42 [-0.55, -0.29]
Yanqin 2017	4.32	0.14	35	4.76	0.11	33	1.9%	-0.44 [-0.50, -0.38]
Zhaogang 2021	3	0.78	49	4.01	0.84	49	1.4%	-1.01 [-1.33, -0.69]
**Subtotal (95% CI)**			**1936**			**1549**	**82.7%**	**-0.39 [-0.48, -0.31]**
Heterogeneity: Tau² = 0.08; Chi² = 410.07, df = 73 (P < 0.00001); I² = 82%								
Test for overall effect: Z = 9.02 (P < 0.00001)								
**Total (95% CI)**			**2370**			**1882**	**100.0%**	**-0.35 [-0.43, -0.27]**
Heterogeneity: Tau² = 0.08; Chi² = 452.93, df = 88 (P < 0.00001); I² = 81%								
Test for overall effect: Z = 8.92 (P < 0.00001)								
Test for subgroup differences: Chi² = 11.62, df = 1 (P = 0.0007), I² = 91.4%								

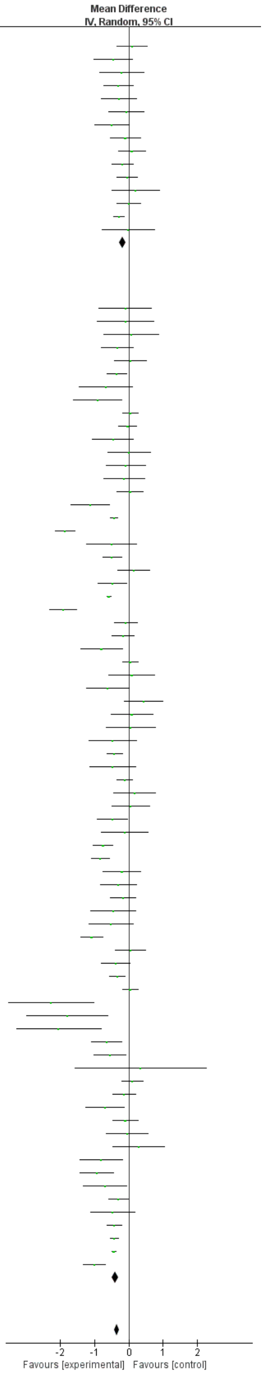

Figure S11.5. Forest plot of subgroup analyses by weekly exercise duration

Study or Subgroup	Experimental Mean	Experimental SD	Experimental Total	Control Mean	Control SD	Control Total	Weight	Mean Difference IV, Random, 95% CI
**2.5.1 <150 minutes per week**								
Benson 2008	4.3	1.1	32	4.2	0.7	35	1.2%	0.10 [-0.35, 0.55]
Boer 2014(1)	4	0.59	17	4.45	0.67	7	0.9%	-0.45 [-1.02, 0.12]
Boer 2014(2)	4.24	0.81	15	4.45	0.67	7	0.8%	-0.21 [-0.85, 0.43]
Bruyndonckx 2015	3.63	0.51	27	3.98	0.5	21	1.5%	-0.35 [-0.64, -0.06]
Chuensiri 2018(1)	3.78	0.69	11	4.45	0.77	5	0.6%	-0.67 [-1.46, 0.12]
Chuensiri 2018(2)	3.54	0.7	15	4.45	0.77	6	0.7%	-0.91 [-1.62, -0.20]
Dias 2018(1)	3.79	0.64	19	4.25	0.81	9	0.9%	-0.46 [-1.06, 0.14]
Dias 2018(2)	4.26	0.87	22	4.25	0.81	10	0.9%	0.01 [-0.61, 0.63]
Faria 2020(1)	3.6	0.75	24	3.88	0.77	13	1.0%	-0.28 [-0.80, 0.24]
Faria 2020(2)	3.81	0.76	26	3.88	0.77	13	1.0%	-0.07 [-0.58, 0.44]
Harder-Lauridsen 2014	3.8	0.84	14	4.3	0.46	14	1.1%	-0.50 [-1.00, 0.00]
Hongling 2012	4.1	0.7	30	4.2	1	30	1.2%	-0.10 [-0.54, 0.34]
Lingling 2021	5	0.87	20	5.47	1.29	20	0.8%	-0.47 [-1.15, 0.21]
Liqun 2014	4.32	0.78	76	4.44	0.81	97	1.6%	-0.12 [-0.36, 0.12]
Lira 2017(1)	4.41	1.04	26	4.24	0.93	16	0.9%	0.17 [-0.44, 0.78]
Meng 2022(1)	4	0.5	12	4.2	0.6	6	1.0%	-0.20 [-0.76, 0.36]
Meng 2022(2)	3.9	0.5	11	4.2	0.6	7	1.0%	-0.30 [-0.83, 0.23]
Murphy 2009	4.16	0.82	23	4.11	0.5	12	1.2%	0.05 [-0.39, 0.49]
Plavsic 2020	4.02	0.74	22	3.93	0.6	22	1.3%	0.09 [-0.31, 0.49]
Racil 2013(1)	3.53	0.31	11	3.86	0.16	6	1.7%	-0.33 [-0.55, -0.11]
Racil 2013(2)	3.9	0.32	11	3.86	0.16	6	1.7%	0.04 [-0.19, 0.27]
Salus(2) 2022	3.99	0.37	14	4.64	0.75	14	1.2%	-0.65 [-1.09, -0.21]
Savoye 2007	4.16	0.84	75	4.34	0.84	44	1.5%	-0.18 [-0.49, 0.13]
Savoye 2011	4.2	0.76	75	4.25	0.81	44	1.5%	-0.05 [-0.34, 0.24]
Staiano 2018	3.8	0.95	22	4.49	0.98	23	1.0%	-0.69 [-1.25, -0.13]
Tas 2023	3.78	0.7	31	3.82	0.7	6	0.9%	-0.04 [-0.65, 0.57]
Wong 2008	4.7	1	12	4.5	0.7	12	0.8%	0.20 [-0.49, 0.89]
Zehsaz 2017	4.19	1.14	16	4.2	1.08	16	0.7%	-0.01 [-0.78, 0.76]
**Subtotal (95% CI)**			**709**			**521**	**30.3%**	**-0.19 [-0.28, -0.10]**
Heterogeneity: Tau² = 0.01; Chi² = 32.85, df = 27 (P = 0.20); I² = 18%								
Test for overall effect: Z = 4.17 (P < 0.0001)								
**2.5.2 ≥150 minutes per week**								
Ahmadi 2020(1)	4.08	0.62	28	4.18	1.12	9	0.7%	-0.10 [-0.87, 0.67]
Ahmadi 2020(2)	4.08	1.04	29	4.18	1.12	9	0.6%	-0.10 [-0.92, 0.72]
Ahmadi 2020(3)	4.25	0.93	29	4.18	1.12	9	0.6%	0.07 [-0.74, 0.88]
Bezerra 2023	4.2	0.74	20	4.54	0.81	21	1.1%	-0.34 [-0.81, 0.13]
Bowen 2010	4.1	0.89	22	4.05	0.59	18	1.1%	0.05 [-0.41, 0.51]
Chae 2010	4.2	0.76	19	4.51	0.59	19	1.2%	-0.31 [-0.74, 0.12]
Davis 2020	4.23	0.95	90	4.19	0.53	85	1.7%	0.04 [-0.19, 0.27]
DeBar 2012	4.12	0.8	74	4.15	0.63	42	1.6%	-0.03 [-0.29, 0.23]
Duft 2020	4.32	0.89	18	4.4	0.91	19	0.9%	-0.08 [-0.66, 0.50]
Elmahgoub 2009	4.23	0.9	15	4.37	0.77	15	0.9%	-0.14 [-0.74, 0.46]
Farpour-Lambert 2009	4.04	0.79	22	4	0.5	22	1.3%	0.04 [-0.35, 0.43]
Ghorbanian 2013	4.75	0.66	15	5.87	0.9	15	0.9%	-1.12 [-1.68, -0.56]
Guijun 2009	4.59	0.15	16	5.02	0.12	10	1.9%	-0.43 [-0.53, -0.33]
Gulijianati 2022	4.07	0.62	47	5.93	0.77	46	1.5%	-1.86 [-2.14, -1.58]
Guo 2015	4.5	0.7	23	5	1.2	12	0.7%	-0.50 [-1.24, 0.24]
Hong 2016	3.57	0.74	55	4.06	0.73	55	1.6%	-0.49 [-0.76, -0.22]
Horner 2015(1)	3.87	0.87	27	3.72	0.48	8	1.1%	0.15 [-0.32, 0.62]
Horner 2015(2)	3.24	0.6	23	3.72	0.48	8	1.2%	-0.48 [-0.89, -0.07]
Jian 2014	4.2	0.13	44	4.78	0.12	44	1.9%	-0.58 [-0.63, -0.53]
Jianjun 2012	3.86	0.63	20	5.78	0.55	15	1.3%	-1.92 [-2.31, -1.53]
Jun 2006(1)	3.72	0.6	33	3.81	0.75	31	1.4%	-0.09 [-0.42, 0.24]
Jun 2006(2)	3.7	0.56	35	3.87	0.77	32	1.4%	-0.17 [-0.49, 0.15]
Ke 2024	4.1	0.4	10	4.9	0.9	10	0.9%	-0.80 [-1.41, -0.19]
Kelishadi 2008	4.18	0.56	45	4.14	0.56	47	1.6%	0.04 [-0.19, 0.27]
Kelly 2004	4.01	0.7	10	3.92	0.82	10	0.8%	0.09 [-0.58, 0.76]
Kim 2007	4.02	0.69	14	4.63	0.9	12	0.9%	-0.61 [-1.23, 0.01]
Kim 2022	4.17	0.82	13	3.74	0.64	13	0.9%	0.43 [-0.14, 1.00]
Lee 2010(1)	4.55	0.48	20	4.46	0.88	9	0.9%	0.09 [-0.52, 0.70]
Lee 2010(2)	4.52	0.89	16	4.46	0.88	9	0.7%	0.06 [-0.66, 0.78]
Liangmei 2015	3.63	0.83	12	4.1	0.89	12	0.8%	-0.47 [-1.16, 0.22]
Lidan 2012	3.93	0.27	30	4.35	0.58	30	1.6%	-0.42 [-0.65, -0.19]
Lira 2017(2)	4.3	0.86	25	4.24	0.93	17	1.0%	0.06 [-0.50, 0.62]
Liu 2018	3.52	0.66	30	4	0.88	20	1.2%	-0.48 [-0.93, -0.03]
Lopes 2016	4.31	1.09	17	4.42	0.92	16	0.8%	-0.11 [-0.80, 0.58]
Luo 2013(1)	3.58	0.74	43	4.33	0.56	34	1.5%	-0.75 [-1.04, -0.46]
Luo 2013(2)	3.64	0.78	52	4.47	0.54	38	1.6%	-0.83 [-1.10, -0.56]
Mohammadi 2019	4.24	0.49	10	4.41	0.36	10	1.3%	-0.17 [-0.55, 0.21]
Monteiro 2015(1)	2.91	0.65	18	3.37	0.86	8	0.8%	-0.46 [-1.13, 0.21]
Monteiro 2015(2)	2.85	0.49	14	3.37	0.86	8	0.8%	-0.52 [-1.17, 0.13]
Morgan 2021	3.87	0.39	15	4.96	0.51	15	1.4%	-1.09 [-1.41, -0.77]
Park 2007	4.16	0.62	19	4.54	0.74	21	1.2%	-0.38 [-0.80, 0.04]
Ramezani 2017(1)	4.02	1.31	15	6.29	1.21	5	0.3%	-2.27 [-3.52, -1.02]
Ramezani 2017(2)	4.49	1.1	15	6.29	1.21	5	0.3%	-1.80 [-3.00, -0.60]
Ramezani 2017(3)	4.24	1.26	15	6.29	1.21	5	0.3%	-2.05 [-3.29, -0.81]
Saygın 2011	2.48	0.85	20	3.03	0.63	19	1.1%	-0.55 [-1.02, -0.08]
Seo 2012	4.39	2.54	10	4.05	1.79	10	0.1%	0.34 [-1.59, 2.27]
Shalitin 2009	4.28	0.81	55	4.17	0.88	55	1.5%	0.11 [-0.21, 0.43]
Staiano 2017	4.08	0.38	20	4.21	0.63	18	1.4%	-0.13 [-0.47, 0.21]
Sun 2011	3.9	0.6	25	4	0.6	17	1.3%	-0.10 [-0.47, 0.27]
Tsang 2009	4.1	0.83	11	3.81	0.85	8	0.7%	0.29 [-0.48, 1.06]
Vasconcellos 2016	3.88	0.65	10	4.69	0.78	10	0.8%	-0.81 [-1.44, -0.18]
Wen 2008	3.89	0.53	24	4.83	0.8	13	1.1%	-0.94 [-1.42, -0.46]
Woo 2004	4.5	0.8	41	4.5	0.8	41	1.4%	0.00 [-0.35, 0.35]
Woo 2012	4.07	0.83	10	4.76	0.6	10	0.8%	-0.69 [-1.32, -0.06]
Xianbo 2011	0.9	0.4	25	1.2	0.5	17	1.5%	-0.30 [-0.58, -0.02]
Xinhui 2016	3.64	0.84	14	4.11	0.89	14	0.8%	-0.47 [-1.11, 0.17]
Xishao 2009	3.93	0.27	34	4.35	0.58	33	1.7%	-0.42 [-0.64, -0.20]
Xishao 2010	3.93	0.27	100	4.35	0.58	100	1.8%	-0.42 [-0.55, -0.29]
Yanqin 2017	4.32	0.14	35	4.76	0.11	33	1.9%	-0.44 [-0.50, -0.38]
Zehsaz 2016	3.91	0.21	16	4.19	0.24	16	1.8%	-0.28 [-0.44, -0.12]
Zhaogang 2021	3	0.78	49	4.01	0.84	49	1.4%	-1.01 [-1.33, -0.69]
**Subtotal (95% CI)**			**1661**			**1361**	**69.7%**	**-0.41 [-0.51, -0.32]**
Heterogeneity: Tau² = 0.08; Chi² = 376.56, df = 60 (P < 0.00001); I² = 84%								
Test for overall effect: Z = 8.65 (P < 0.00001)								
**Total (95% CI)**			**2370**			**1882**	**100.0%**	**-0.35 [-0.43, -0.27]**
Heterogeneity: Tau² = 0.08; Chi² = 452.93, df = 88 (P < 0.00001); I² = 81%								
Test for overall effect: Z = 8.92 (P < 0.00001)								
Test for subgroup differences: Chi² = 10.89, df = 1 (P = 0.0010), I² = 90.8%								

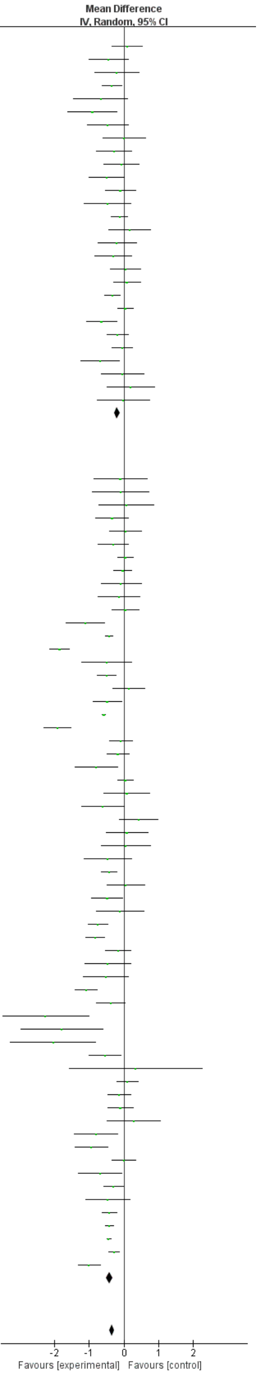

Figure S11.6. Forest plot of subgroup analyses by intervention duration

Study or Subgroup	Experimental Mean	Experimental SD	Experimental Total	Control Mean	Control SD	Control Total	Weight	Mean Difference IV, Random, 95% CI
**2.6.1 <12 weeks (total intervention duration)**								
Ahmadi 2020(1)	4.08	0.62	28	4.18	1.12	9	0.7%	-0.10 [-0.87, 0.67]
Ahmadi 2020(2)	4.08	1.04	29	4.18	1.12	9	0.6%	-0.10 [-0.92, 0.72]
Ahmadi 2020(3)	4.25	0.93	29	4.18	1.12	9	0.6%	0.07 [-0.74, 0.88]
Benson 2008	4.3	1.1	32	4.2	0.7	35	1.2%	0.10 [-0.35, 0.55]
Bezerra 2023	4.2	0.74	20	4.54	0.81	21	1.1%	-0.34 [-0.81, 0.13]
Bowen 2010	4.1	0.89	22	4.05	0.59	18	1.1%	0.05 [-0.41, 0.51]
Elmahgoub 2009	4.23	0.9	15	4.37	0.77	15	0.9%	-0.14 [-0.74, 0.46]
Ghorbanian 2013	4.75	0.66	15	5.87	0.9	15	0.9%	-1.12 [-1.68, -0.56]
Hongling 2012	4.1	0.7	30	4.2	1	30	1.2%	-0.10 [-0.54, 0.34]
Kelly 2004	4.01	0.7	10	3.92	0.82	10	0.8%	0.09 [-0.58, 0.76]
Kim 2007	4.02	0.69	14	4.63	0.9	12	0.9%	-0.61 [-1.23, 0.01]
Lee 2010(1)	4.55	0.48	20	4.46	0.88	9	0.9%	0.09 [-0.52, 0.70]
Lee 2010(2)	4.52	0.89	16	4.46	0.88	9	0.7%	0.06 [-0.66, 0.78]
Liu 2018	3.52	0.66	30	4	0.88	20	1.2%	-0.48 [-0.93, -0.03]
Luo 2013(1)	3.58	0.74	43	4.33	0.56	34	1.5%	-0.75 [-1.04, -0.46]
Luo 2013(2)	3.64	0.78	52	4.47	0.54	38	1.6%	-0.83 [-1.10, -0.56]
Mohammadi 2019	4.24	0.49	10	4.41	0.36	10	1.3%	-0.17 [-0.55, 0.21]
Ramezani 2017(1)	4.02	1.31	15	6.29	1.21	5	0.3%	-2.27 [-3.52, -1.02]
Ramezani 2017(2)	4.49	1.1	15	6.29	1.21	5	0.3%	-1.80 [-3.00, -0.60]
Ramezani 2017(3)	4.24	1.26	15	6.29	1.21	5	0.3%	-2.05 [-3.29, -0.81]
Seo 2012	4.39	2.54	10	4.05	1.79	10	0.1%	0.34 [-1.59, 2.27]
Sun 2011	3.9	0.6	25	4	0.6	17	1.3%	-0.10 [-0.47, 0.27]
Tas 2023	3.78	0.7	31	3.82	0.7	6	0.9%	-0.04 [-0.65, 0.57]
Woo 2004	4.5	0.8	41	4.5	0.8	41	1.4%	0.00 [-0.35, 0.35]
Xianbo 2011	0.9	0.4	25	1.2	0.5	17	1.5%	-0.30 [-0.58, -0.02]
**Subtotal (95% CI)**			**592**			**409**	**23.4%**	**-0.34 [-0.52, -0.15]**
Heterogeneity: Tau² = 0.12; Chi² = 72.61, df = 24 (P < 0.00001); I² = 67%								
Test for overall effect: Z = 3.61 (P = 0.0003)								
**2.6.2 ≥12 weeks (total intervention duration)**								
Boer 2014(1)	4	0.59	17	4.45	0.67	7	0.9%	-0.45 [-1.02, 0.12]
Boer 2014(2)	4.24	0.81	15	4.45	0.67	7	0.8%	-0.21 [-0.85, 0.43]
Bruyndonckx 2015	3.63	0.51	27	3.98	0.5	21	1.5%	-0.35 [-0.64, -0.06]
Chae 2010	4.2	0.76	19	4.51	0.59	19	1.2%	-0.31 [-0.74, 0.12]
Chuensiri 2018(1)	3.78	0.69	11	4.45	0.77	5	0.6%	-0.67 [-1.46, 0.12]
Chuensiri 2018(2)	3.54	0.7	15	4.45	0.77	6	0.7%	-0.91 [-1.62, -0.20]
Davis 2020	4.23	0.95	90	4.19	0.53	85	1.7%	0.04 [-0.19, 0.27]
DeBar 2012	4.12	0.8	74	4.15	0.63	42	1.6%	-0.03 [-0.29, 0.23]
Dias 2018(1)	3.79	0.64	19	4.25	0.81	9	0.9%	-0.46 [-1.06, 0.14]
Dias 2018(2)	4.26	0.87	22	4.25	0.81	10	0.9%	0.01 [-0.61, 0.63]
Duft 2020	4.32	0.89	18	4.4	0.91	19	0.9%	-0.08 [-0.66, 0.50]
Faria 2020(1)	3.6	0.75	24	3.88	0.77	13	1.0%	-0.28 [-0.80, 0.24]
Faria 2020(2)	3.81	0.76	26	3.88	0.77	13	1.0%	-0.07 [-0.58, 0.44]
Farpour-Lambert 2009	4.04	0.79	22	4	0.5	22	1.3%	0.04 [-0.35, 0.43]
Guijun 2009	4.59	0.15	16	5.02	0.12	10	1.9%	-0.43 [-0.53, -0.33]
Gulijianati 2022	4.07	0.62	47	5.93	0.77	46	1.5%	-1.86 [-2.14, -1.58]
Guo 2015	4.5	0.7	23	5	1.2	12	0.7%	-0.50 [-1.24, 0.24]
Harder-Lauridsen 2014	3.8	0.84	14	4.3	0.46	14	1.1%	-0.50 [-1.00, 0.00]
Hong 2016	3.57	0.74	55	4.06	0.73	55	1.6%	-0.49 [-0.76, -0.22]
Horner 2015(1)	3.87	0.87	27	3.72	0.48	8	1.1%	0.15 [-0.32, 0.62]
Horner 2015(2)	3.24	0.6	23	3.72	0.48	8	1.2%	-0.48 [-0.89, -0.07]
Jian 2014	4.2	0.13	44	4.78	0.12	44	1.9%	-0.58 [-0.63, -0.53]
Jianjun 2012	3.86	0.63	20	5.78	0.55	15	1.3%	-1.92 [-2.31, -1.53]
Jun 2006(1)	3.72	0.6	33	3.81	0.75	31	1.4%	-0.09 [-0.42, 0.24]
Jun 2006(2)	3.7	0.56	35	3.87	0.77	32	1.4%	-0.17 [-0.49, 0.15]
Ke 2024	4.1	0.4	10	4.9	0.9	10	0.9%	-0.80 [-1.41, -0.19]
Kelishadi 2008	4.18	0.56	45	4.14	0.56	47	1.6%	0.04 [-0.19, 0.27]
Kim 2022	4.17	0.82	13	3.74	0.64	13	0.9%	0.43 [-0.14, 1.00]
Liangmei 2015	3.63	0.83	12	4.1	0.89	12	0.8%	-0.47 [-1.16, 0.22]
Lidan 2012	3.93	0.27	30	4.35	0.58	30	1.6%	-0.42 [-0.65, -0.19]
Lingling 2021	5	0.87	20	5.47	1.29	20	0.8%	-0.47 [-1.15, 0.21]
Liqun 2014	4.32	0.78	76	4.44	0.81	97	1.6%	-0.12 [-0.36, 0.12]
Lira 2017(1)	4.41	1.04	26	4.24	0.93	16	0.9%	0.17 [-0.44, 0.78]
Lira 2017(2)	4.3	0.86	25	4.24	0.93	17	1.0%	0.06 [-0.50, 0.62]
Lopes 2016	4.31	1.09	17	4.42	0.92	16	0.8%	-0.11 [-0.80, 0.58]
Meng 2022(1)	4	0.5	12	4.2	0.6	6	1.0%	-0.20 [-0.76, 0.36]
Meng 2022(2)	3.9	0.5	11	4.2	0.6	7	1.0%	-0.30 [-0.83, 0.23]
Monteiro 2015(1)	2.91	0.65	18	3.37	0.86	8	0.8%	-0.46 [-1.13, 0.21]
Monteiro 2015(2)	2.85	0.49	14	3.37	0.86	8	0.8%	-0.52 [-1.17, 0.13]
Morgan 2021	3.87	0.39	15	4.96	0.51	15	1.4%	-1.09 [-1.41, -0.77]
Murphy 2009	4.16	0.82	23	4.11	0.5	12	1.2%	0.05 [-0.39, 0.49]
Park 2007	4.16	0.62	19	4.54	0.74	21	1.2%	-0.38 [-0.80, 0.04]
Plavsic 2020	4.02	0.74	22	3.93	0.6	22	1.3%	0.09 [-0.31, 0.49]
Racil 2013(1)	3.53	0.31	11	3.86	0.16	6	1.7%	-0.33 [-0.55, -0.11]
Racil 2013(2)	3.9	0.32	11	3.86	0.16	6	1.7%	0.04 [-0.19, 0.27]
Salus(2) 2022	3.99	0.37	14	4.64	0.75	14	1.2%	-0.65 [-1.09, -0.21]
Savoye 2007	4.16	0.84	75	4.34	0.84	44	1.5%	-0.18 [-0.49, 0.13]
Savoye 2011	4.2	0.76	75	4.25	0.81	44	1.5%	-0.05 [-0.34, 0.24]
Saygın 2011	2.48	0.85	20	3.03	0.63	19	1.1%	-0.55 [-1.02, -0.08]
Shalitin 2009	4.28	0.81	55	4.17	0.88	55	1.5%	0.11 [-0.21, 0.43]
Staiano 2017	4.08	0.38	20	4.21	0.63	18	1.4%	-0.13 [-0.47, 0.21]
Staiano 2018	3.8	0.95	22	4.49	0.98	23	1.0%	-0.69 [-1.25, -0.13]
Tsang 2009	4.1	0.83	11	3.81	0.85	8	0.7%	0.29 [-0.48, 1.06]
Vasconcellos 2016	3.88	0.65	10	4.69	0.78	10	0.8%	-0.81 [-1.44, -0.18]
Wen 2008	3.89	0.53	24	4.83	0.8	13	1.1%	-0.94 [-1.42, -0.46]
Wong 2008	4.7	1	12	4.5	0.7	12	0.8%	0.20 [-0.49, 0.89]
Woo 2012	4.07	0.83	10	4.76	0.6	10	0.8%	-0.69 [-1.32, -0.06]
Xinhui 2016	3.64	0.84	14	4.11	0.89	14	0.8%	-0.47 [-1.11, 0.17]
Xishao 2009	3.93	0.27	34	4.35	0.58	33	1.7%	-0.42 [-0.64, -0.20]
Xishao 2010	3.93	0.27	100	4.35	0.58	100	1.8%	-0.42 [-0.55, -0.29]
Yanqin 2017	4.32	0.14	35	4.76	0.11	33	1.9%	-0.44 [-0.50, -0.38]
Zehsaz 2016	3.91	0.21	16	4.19	0.24	16	1.8%	-0.28 [-0.44, -0.12]
Zehsaz 2017	4.19	1.14	16	4.2	1.08	16	0.7%	-0.01 [-0.78, 0.76]
Zhaogang 2021	3	0.78	49	4.01	0.84	49	1.4%	-1.01 [-1.33, -0.69]
**Subtotal (95% CI)**			**1778**			**1473**	**76.6%**	**-0.36 [-0.44, -0.27]**
Heterogeneity: Tau² = 0.08; Chi² = 377.37, df = 63 (P < 0.00001); I² = 83%								
Test for overall effect: Z = 8.06 (P < 0.00001)								
**Total (95% CI)**			**1778**			**1882**	**100.0%**	**-0.35 [-0.43, -0.27]**
Heterogeneity: Tau² = 0.08; Chi² = 452.93, df = 88 (P < 0.00001); I² = 81%								
Test for overall effect: Z = 8.92 (P < 0.00001)								
Test for subgroup differences: Chi² = 0.05, df = 1 (P = 0.83), I² = 0%								

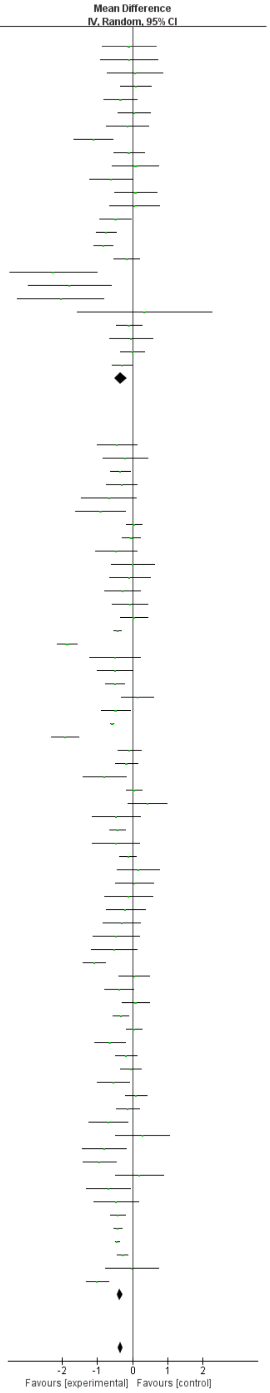

Figure S11.7. Forest plot of subgroup analyses by region

Study or Subgroup	Experimental Mean	Experimental SD	Experimental Total	Control Mean	Control SD	Control Total	Weight	Mean Difference IV, Random, 95% CI
**2.7.1 Asia**								
Ahmadi 2020(1)	4.08	0.62	28	4.18	1.12	9	0.7%	-0.10 [-0.87, 0.67]
Ahmadi 2020(2)	4.08	1.04	29	4.18	1.12	9	0.6%	-0.10 [-0.92, 0.72]
Ahmadi 2020(3)	4.25	0.93	29	4.18	1.12	9	0.6%	0.07 [-0.74, 0.88]
Bowen 2010	4.1	0.89	22	4.05	0.59	18	1.1%	0.05 [-0.41, 0.51]
Chae 2010	4.2	0.76	19	4.51	0.59	19	1.2%	-0.31 [-0.74, 0.12]
Chuensiri 2018(1)	3.78	0.69	11	4.45	0.77	5	0.6%	-0.67 [-1.46, 0.12]
Chuensiri 2018(2)	3.54	0.7	15	4.45	0.77	6	0.7%	-0.91 [-1.62, -0.20]
Ghorbanian 2013	4.75	0.66	15	5.87	0.9	15	0.9%	-1.12 [-1.68, -0.56]
Guijun 2009	4.59	0.15	16	5.02	0.12	10	1.9%	-0.43 [-0.53, -0.33]
Gulijianati 2022	4.07	0.62	47	5.93	0.77	46	1.5%	-1.86 [-2.14, -1.58]
Guo 2015	4.5	0.7	23	5	1.2	12	0.7%	-0.50 [-1.24, 0.24]
Hong 2016	3.57	0.74	55	4.06	0.73	55	1.6%	-0.49 [-0.76, -0.22]
Hongling 2012	4.1	0.7	30	4.2	1	30	1.2%	-0.10 [-0.54, 0.34]
Jian 2014	4.2	0.13	44	4.78	0.12	44	1.9%	-0.58 [-0.63, -0.53]
Jianjun 2012	3.86	0.63	20	5.78	0.55	15	1.3%	-1.92 [-2.31, -1.53]
Jun 2006(1)	3.72	0.6	33	3.81	0.75	31	1.4%	-0.09 [-0.42, 0.24]
Jun 2006(2)	3.7	0.56	35	3.87	0.77	32	1.4%	-0.17 [-0.49, 0.15]
Ke 2024	4.1	0.4	10	4.9	0.9	10	0.9%	-0.80 [-1.41, -0.19]
Kelishadi 2008	4.18	0.56	45	4.14	0.56	47	1.6%	0.04 [-0.19, 0.27]
Kim 2007	4.02	0.69	14	4.63	0.9	12	0.9%	-0.61 [-1.23, 0.01]
Kim 2022	4.17	0.82	13	3.74	0.64	13	0.9%	0.43 [-0.14, 1.00]
Lee 2010(1)	4.55	0.48	20	4.46	0.88	9	0.9%	0.09 [-0.52, 0.70]
Lee 2010(2)	4.52	0.89	16	4.46	0.88	9	0.7%	0.06 [-0.66, 0.78]
Liangmei 2015	3.63	0.83	12	4.1	0.89	12	0.8%	-0.47 [-1.16, 0.22]
Lidan 2012	3.93	0.27	30	4.35	0.58	30	1.6%	-0.42 [-0.65, -0.19]
Lingling 2021	5	0.87	20	5.47	1.29	20	0.8%	-0.47 [-1.15, 0.21]
Liqun 2014	4.32	0.78	76	4.44	0.81	97	1.6%	-0.12 [-0.36, 0.12]
Liu 2018	3.52	0.66	30	4	0.88	20	1.2%	-0.48 [-0.93, -0.03]
Luo 2013(1)	3.58	0.74	43	4.33	0.56	34	1.5%	-0.75 [-1.04, -0.46]
Luo 2013(2)	3.64	0.78	52	4.47	0.54	38	1.6%	-0.83 [-1.10, -0.56]
Meng 2022(1)	4	0.5	12	4.2	0.6	6	1.0%	-0.20 [-0.76, 0.36]
Meng 2022(2)	3.9	0.5	11	4.2	0.6	7	1.0%	-0.30 [-0.83, 0.23]
Mohammadi 2019	4.24	0.49	10	4.41	0.36	10	1.3%	-0.17 [-0.55, 0.21]
Park 2007	4.16	0.62	19	4.54	0.74	21	1.2%	-0.38 [-0.80, 0.04]
Ramezani 2017(1)	4.02	1.31	15	6.29	1.21	5	0.3%	-2.27 [-3.52, -1.02]
Ramezani 2017(2)	4.49	1.1	15	6.29	1.21	5	0.3%	-1.80 [-3.00, -0.60]
Ramezani 2017(3)	4.24	1.26	15	6.29	1.21	5	0.3%	-2.05 [-3.29, -0.81]
Saygın 2011	2.48	0.85	20	3.03	0.63	19	1.1%	-0.55 [-1.02, -0.08]
Seo 2012	4.39	2.54	10	4.05	1.79	10	0.1%	0.34 [-1.59, 2.27]
Shalitin 2009	4.28	0.81	55	4.17	0.88	55	1.5%	0.11 [-0.21, 0.43]
Sun 2011	3.9	0.6	25	4	0.6	17	1.3%	-0.10 [-0.47, 0.27]
Wen 2008	3.89	0.53	24	4.83	0.8	13	1.1%	-0.94 [-1.42, -0.46]
Wong 2008	4.7	1	12	4.5	0.7	12	0.8%	0.20 [-0.49, 0.89]
Woo 2004	4.5	0.8	41	4.5	0.8	41	1.4%	0.00 [-0.35, 0.35]
Woo 2012	4.07	0.83	10	4.76	0.6	10	0.8%	-0.69 [-1.32, -0.06]
Xianbo 2011	0.9	0.4	25	1.2	0.5	17	1.5%	-0.30 [-0.58, -0.02]
Xinhui 2016	3.64	0.84	14	4.11	0.89	14	0.8%	-0.47 [-1.11, 0.17]
Xishao 2009	3.93	0.27	34	4.35	0.58	33	1.7%	-0.42 [-0.64, -0.20]
Xishao 2010	3.93	0.27	100	4.35	0.58	100	1.8%	-0.42 [-0.55, -0.29]
Yanqin 2017	4.32	0.14	35	4.76	0.11	33	1.9%	-0.44 [-0.50, -0.38]
Zehsaz 2016	3.91	0.21	16	4.19	0.24	16	1.8%	-0.28 [-0.44, -0.12]
Zehsaz 2017	4.19	1.14	16	4.2	1.08	16	0.7%	-0.01 [-0.78, 0.76]
Zhaogang 2021	3	0.78	49	4.01	0.84	49	1.4%	-1.01 [-1.33, -0.69]
**Subtotal (95% CI)**			**1435**			**1230**	**59.7%**	**-0.45 [-0.55, -0.35]**
Heterogeneity: Tau² = 0.07; Chi² = 318.53, df = 52 (P < 0.00001); I² = 84%								
Test for overall effect: Z = 9.06 (P < 0.00001)								
**2.7.2 Europe**								
Boer 2014(1)	4	0.59	17	4.45	0.67	7	0.9%	-0.45 [-1.02, 0.12]
Boer 2014(2)	4.24	0.81	15	4.45	0.67	7	0.8%	-0.21 [-0.85, 0.43]
Bruyndonckx 2015	3.63	0.51	27	3.98	0.5	21	1.5%	-0.35 [-0.64, -0.06]
Elmahgoub 2009	4.23	0.9	15	4.37	0.77	15	0.9%	-0.14 [-0.74, 0.46]
Farpour-Lambert 2009	4.04	0.79	22	4	0.5	22	1.3%	0.04 [-0.35, 0.43]
Harder-Lauridsen 2014	3.8	0.84	14	4.3	0.46	14	1.1%	-0.50 [-1.00, 0.00]
Plavsic 2020	4.02	0.74	22	3.93	0.6	22	1.3%	0.09 [-0.31, 0.49]
Salus(2) 2022	3.99	0.37	14	4.64	0.75	14	1.2%	-0.65 [-1.09, -0.21]
**Subtotal (95% CI)**			**146**			**122**	**9.0%**	**-0.26 [-0.45, -0.07]**
Heterogeneity: Tau² = 0.02; Chi² = 10.14, df = 7 (P = 0.18); I² = 31%								
Test for overall effect: Z = 2.68 (P = 0.007)								
**2.7.3 Africa**								
Morgan 2021	3.87	0.39	15	4.96	0.51	15	1.4%	-1.09 [-1.41, -0.77]
Racil 2013(1)	3.53	0.31	11	3.86	0.16	6	1.7%	-0.33 [-0.55, -0.11]
Racil 2013(2)	3.9	0.32	11	3.86	0.16	6	1.7%	0.04 [-0.19, 0.27]
**Subtotal (95% CI)**			**37**			**27**	**4.7%**	**-0.45 [-1.03, 0.13]**
Heterogeneity: Tau² = 0.24; Chi² = 31.10, df = 2 (P < 0.00001); I² = 94%								
Test for overall effect: Z = 1.52 (P = 0.13)								
**2.7.4 North America**								
Davis 2020	4.23	0.95	90	4.19	0.53	85	1.7%	0.04 [-0.19, 0.27]
DeBar 2012	4.12	0.8	74	4.15	0.63	42	1.6%	-0.03 [-0.29, 0.23]
Horner 2015(1)	3.87	0.87	27	3.72	0.48	8	1.1%	0.15 [-0.32, 0.62]
Horner 2015(2)	3.24	0.6	23	3.72	0.48	8	1.2%	-0.48 [-0.89, -0.07]
Kelly 2004	4.01	0.7	10	3.92	0.82	10	0.8%	0.09 [-0.58, 0.76]
Murphy 2009	4.16	0.82	23	4.11	0.5	12	1.2%	0.05 [-0.39, 0.49]
Savoye 2007	4.16	0.84	75	4.34	0.84	44	1.5%	-0.18 [-0.49, 0.13]
Savoye 2011	4.2	0.76	75	4.25	0.81	44	1.5%	-0.05 [-0.34, 0.24]
Staiano 2017	4.08	0.38	20	4.21	0.63	18	1.4%	-0.13 [-0.47, 0.21]
Staiano 2018	3.8	0.95	22	4.49	0.98	23	1.0%	-0.69 [-1.25, -0.13]
Tas 2023	3.78	0.7	31	3.82	0.7	6	0.9%	-0.04 [-0.65, 0.57]
**Subtotal (95% CI)**			**470**			**300**	**13.8%**	**-0.09 [-0.20, 0.03]**
Heterogeneity: Tau² = 0.00; Chi² = 11.36, df = 10 (P = 0.33); I² = 12%								
Test for overall effect: Z = 1.47 (P = 0.14)								
**2.7.5 South America**								
Bezerra 2023	4.2	0.74	20	4.54	0.81	21	1.1%	-0.34 [-0.81, 0.13]
Duft 2020	4.32	0.89	18	4.4	0.91	19	0.9%	-0.08 [-0.66, 0.50]
Faria 2020(1)	3.6	0.75	24	3.88	0.77	13	1.0%	-0.28 [-0.80, 0.24]
Faria 2020(2)	3.81	0.76	26	3.88	0.77	13	1.0%	-0.07 [-0.58, 0.44]
Lira 2017(1)	4.41	1.04	26	4.24	0.93	16	0.9%	0.17 [-0.44, 0.78]
Lira 2017(2)	4.3	0.86	25	4.24	0.93	17	1.0%	0.06 [-0.50, 0.62]
Lopes 2016	4.31	1.09	17	4.42	0.92	16	0.8%	-0.11 [-0.80, 0.58]
Monteiro 2015(1)	2.91	0.65	18	3.37	0.86	8	0.8%	-0.46 [-1.13, 0.21]
Monteiro 2015(2)	2.85	0.49	14	3.37	0.86	8	0.8%	-0.52 [-1.17, 0.13]
Vasconcellos 2016	3.88	0.65	10	4.69	0.78	10	0.8%	-0.81 [-1.44, -0.18]
**Subtotal (95% CI)**			**198**			**141**	**9.2%**	**-0.23 [-0.41, -0.05]**
Heterogeneity: Tau² = 0.00; Chi² = 8.20, df = 9 (P = 0.51); I² = 0%								
Test for overall effect: Z = 2.48 (P = 0.01)								
**2.7.7 Oceania**								
Benson 2008	4.3	1.1	32	4.2	0.7	35	1.2%	0.10 [-0.35, 0.55]
Dias 2018(1)	3.79	0.64	19	4.25	0.81	9	0.9%	-0.46 [-1.06, 0.14]
Dias 2018(2)	4.26	0.87	22	4.25	0.81	10	0.9%	0.01 [-0.61, 0.63]
Tsang 2009	4.1	0.83	11	3.81	0.85	8	0.7%	0.29 [-0.48, 1.06]
**Subtotal (95% CI)**			**84**			**62**	**3.6%**	**-0.02 [-0.31, 0.27]**
Heterogeneity: Tau² = 0.00; Chi² = 2.96, df = 3 (P = 0.40); I² = 0%								
Test for overall effect: Z = 0.14 (P = 0.89)								
**Total (95% CI)**			**2370**			**1882**	**100.0%**	**-0.35 [-0.43, -0.27]**
Heterogeneity: Tau² = 0.08; Chi² = 452.93, df = 88 (P < 0.00001); I² = 81%								
Test for overall effect: Z = 8.92 (P < 0.00001)								
Test for subgroup differences: Chi² = 26.07, df = 5 (P < 0.0001), I² = 80.8%								

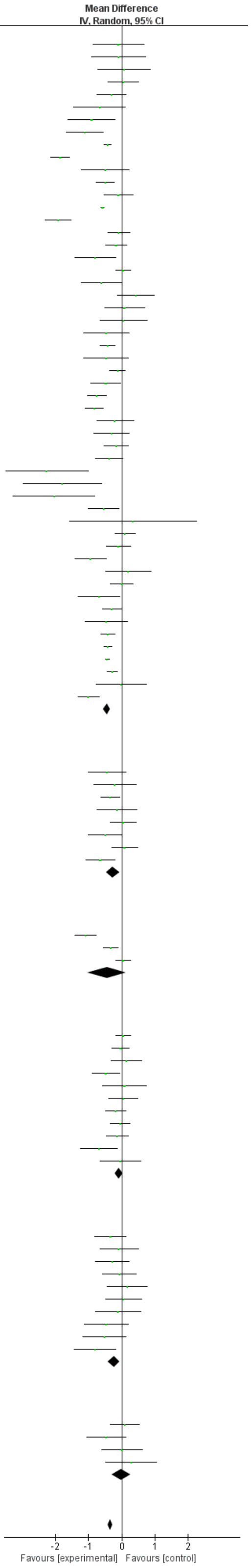

Figure S11.8. Forest plot of subgroup analyses by economic status

Study or Subgroup	Experimental Mean	Experimental SD	Experimental Total	Control Mean	Control SD	Control Total	Weight	Mean Difference IV, Random, 95% CI
**2.8.1 Lower-middle-income countries**								
Morgan 2021	3.87	0.39	15	4.96	0.51	15	1.4%	-1.09 [-1.41, -0.77]
Racil 2013(1)	3.53	0.31	11	3.86	0.16	6	1.7%	-0.33 [-0.55, -0.11]
Racil 2013(2)	3.9	0.32	11	3.86	0.16	6	1.7%	0.04 [-0.19, 0.27]
**Subtotal (95% CI)**			**37**			**27**	**4.7%**	**-0.45 [-1.03, 0.13]**
Heterogeneity: Tau² = 0.24; Chi² = 31.10, df = 2 (P < 0.00001); I² = 94%								
Test for overall effect: Z = 1.52 (P = 0.13)								
**2.8.2 Upper-middle-income countries**								
Ahmadi 2020(1)	4.08	0.62	28	4.18	1.12	9	0.7%	-0.10 [-0.87, 0.67]
Ahmadi 2020(2)	4.08	1.04	29	4.18	1.12	9	0.6%	-0.10 [-0.92, 0.72]
Ahmadi 2020(3)	4.25	0.93	29	4.18	1.12	9	0.6%	0.07 [-0.74, 0.88]
Bezerra 2023	4.2	0.74	20	4.54	0.81	21	1.1%	-0.34 [-0.81, 0.13]
Bowen 2010	4.1	0.89	22	4.05	0.59	18	1.1%	0.05 [-0.41, 0.51]
Chuensiri 2018(1)	3.78	0.69	11	4.45	0.77	5	0.6%	-0.67 [-1.46, 0.12]
Chuensiri 2018(2)	3.54	0.7	15	4.45	0.77	6	0.7%	-0.91 [-1.62, -0.20]
Duft 2020	4.32	0.89	18	4.4	0.91	19	0.9%	-0.08 [-0.66, 0.50]
Faria 2020(1)	3.6	0.75	24	3.88	0.77	13	1.0%	-0.28 [-0.80, 0.24]
Faria 2020(2)	3.81	0.76	26	3.88	0.77	13	1.0%	-0.07 [-0.58, 0.44]
Ghorbanian 2013	4.75	0.66	15	5.87	0.9	15	0.9%	-1.12 [-1.68, -0.56]
Guijun 2009	4.59	0.15	16	5.02	0.12	10	1.9%	-0.43 [-0.53, -0.33]
Gulijianati 2022	4.07	0.62	47	5.93	0.77	46	1.5%	-1.86 [-2.14, -1.58]
Guo 2015	4.5	0.7	23	5	1.2	12	0.7%	-0.50 [-1.24, 0.24]
Hong 2016	3.57	0.74	55	4.06	0.73	55	1.6%	-0.49 [-0.76, -0.22]
Hongling 2012	4.1	0.7	30	4.2	1	30	1.2%	-0.10 [-0.54, 0.34]
Jian 2014	4.2	0.13	44	4.78	0.12	44	1.9%	-0.58 [-0.63, -0.53]
Jianjun 2012	3.86	0.63	20	5.78	0.55	15	1.3%	-1.92 [-2.31, -1.53]
Jun 2006(1)	3.72	0.6	33	3.81	0.75	31	1.4%	-0.09 [-0.42, 0.24]
Jun 2006(2)	3.7	0.56	35	3.87	0.77	32	1.4%	-0.17 [-0.49, 0.15]
Ke 2024	4.1	0.4	10	4.9	0.9	10	0.9%	-0.80 [-1.41, -0.19]
Kelishadi 2008	4.18	0.56	45	4.14	0.56	47	1.6%	0.04 [-0.19, 0.27]
Liangmei 2015	3.63	0.83	12	4.1	0.89	12	0.8%	-0.47 [-1.16, 0.22]
Lidan 2012	3.93	0.27	30	4.35	0.58	30	1.6%	-0.42 [-0.65, -0.19]
Lingling 2021	5	0.87	20	5.47	1.29	20	0.8%	-0.47 [-1.15, 0.21]
Liqun 2014	4.32	0.78	76	4.44	0.81	97	1.6%	-0.12 [-0.36, 0.12]
Lira 2017(1)	4.41	1.04	26	4.24	0.93	16	0.9%	0.17 [-0.44, 0.78]
Lira 2017(2)	4.3	0.86	25	4.24	0.93	17	1.0%	0.06 [-0.50, 0.62]
Liu 2018	3.52	0.66	30	4	0.88	20	1.2%	-0.48 [-0.93, -0.03]
Lopes 2016	4.31	1.09	17	4.42	0.92	16	0.8%	-0.11 [-0.80, 0.58]
Luo 2013(1)	3.58	0.74	43	4.33	0.56	34	1.5%	-0.75 [-1.04, -0.46]
Luo 2013(2)	3.64	0.78	52	4.47	0.54	38	1.6%	-0.83 [-1.10, -0.56]
Meng 2022(1)	4	0.5	12	4.2	0.6	6	1.0%	-0.20 [-0.76, 0.36]
Meng 2022(2)	3.9	0.5	11	4.2	0.6	7	1.0%	-0.30 [-0.83, 0.23]
Mohammadi 2019	4.24	0.49	10	4.41	0.36	10	1.3%	-0.17 [-0.55, 0.21]
Monteiro 2015(1)	2.91	0.65	18	3.37	0.86	8	0.8%	-0.46 [-1.13, 0.21]
Monteiro 2015(2)	2.85	0.49	14	3.37	0.86	8	0.8%	-0.52 [-1.17, 0.13]
Plavsic 2020	4.02	0.74	22	3.93	0.6	22	1.3%	0.09 [-0.31, 0.49]
Ramezani 2017(1)	4.02	1.31	15	6.29	1.21	5	0.3%	-2.27 [-3.52, -1.02]
Ramezani 2017(2)	4.49	1.1	15	6.29	1.21	5	0.3%	-1.80 [-3.00, -0.60]
Ramezani 2017(3)	4.24	1.26	15	6.29	1.21	5	0.3%	-2.05 [-3.29, -0.81]
Saygın 2011	2.48	0.85	20	3.03	0.63	19	1.1%	-0.55 [-1.02, -0.08]
Sun 2011	3.9	0.6	25	4	0.6	17	1.3%	-0.10 [-0.47, 0.27]
Vasconcellos 2016	3.88	0.65	10	4.69	0.78	10	0.8%	-0.81 [-1.44, -0.18]
Wen 2008	3.89	0.53	24	4.83	0.8	13	1.1%	-0.94 [-1.42, -0.46]
Woo 2004	4.5	0.8	41	4.5	0.8	41	1.4%	0.00 [-0.35, 0.35]
Xianbo 2011	0.9	0.4	25	1.2	0.5	17	1.5%	-0.30 [-0.58, -0.02]
Xinhui 2016	3.64	0.84	14	4.11	0.89	14	0.8%	-0.47 [-1.11, 0.17]
Xishao 2009	3.93	0.27	34	4.35	0.58	33	1.7%	-0.42 [-0.64, -0.20]
Xishao 2010	3.93	0.27	100	4.35	0.58	100	1.8%	-0.42 [-0.55, -0.29]
Yanqin 2017	4.32	0.14	35	4.76	0.11	33	1.9%	-0.44 [-0.50, -0.38]
Zehsaz 2016	3.91	0.21	16	4.19	0.24	16	1.8%	-0.28 [-0.44, -0.12]
Zehsaz 2017	4.19	1.14	16	4.2	1.08	16	0.7%	-0.01 [-0.78, 0.76]
Zhaogang 2021	3	0.78	49	4.01	0.84	49	1.4%	-1.01 [-1.33, -0.69]
**Subtotal (95% CI)**			**1467**			**1223**	**61.1%**	**-0.46 [-0.55, -0.36]**
Heterogeneity: Tau² = 0.07; Chi² = 307.22, df = 53 (P < 0.00001); I² = 83%								
Test for overall effect: Z = 9.43 (P < 0.00001)								
**2.8.3 High-income countries**								
Benson 2008	4.3	1.1	32	4.2	0.7	35	1.2%	0.10 [-0.35, 0.55]
Boer 2014(1)	4	0.59	17	4.45	0.67	7	0.9%	-0.45 [-1.02, 0.12]
Boer 2014(2)	4.24	0.81	15	4.45	0.67	7	0.8%	-0.21 [-0.85, 0.43]
Bruyndonckx 2015	3.63	0.51	27	3.98	0.5	21	1.5%	-0.35 [-0.64, -0.06]
Chae 2010	4.2	0.76	19	4.51	0.59	19	1.2%	-0.31 [-0.74, 0.12]
Davis 2020	4.23	0.95	90	4.19	0.53	85	1.7%	0.04 [-0.19, 0.27]
DeBar 2012	4.12	0.8	74	4.15	0.63	42	1.6%	-0.03 [-0.29, 0.23]
Dias 2018(1)	3.79	0.64	19	4.25	0.81	9	0.9%	-0.46 [-1.06, 0.14]
Dias 2018(2)	4.26	0.87	22	4.25	0.81	10	0.9%	0.01 [-0.61, 0.63]
Elmahgoub 2009	4.23	0.9	15	4.37	0.77	15	0.9%	-0.14 [-0.74, 0.46]
Farpour-Lambert 2009	4.04	0.79	22	4	0.5	22	1.3%	0.04 [-0.35, 0.43]
Harder-Lauridsen 2014	3.8	0.84	14	4.3	0.46	14	1.1%	-0.50 [-1.00, 0.00]
Horner 2015(1)	3.87	0.87	27	3.72	0.48	8	1.1%	0.15 [-0.32, 0.62]
Horner 2015(2)	3.24	0.6	23	3.72	0.48	8	1.2%	-0.48 [-0.89, -0.07]
Kelly 2004	4.01	0.7	10	3.92	0.82	10	0.8%	0.09 [-0.58, 0.76]
Kim 2007	4.02	0.69	14	4.63	0.9	12	0.9%	-0.61 [-1.23, 0.01]
Kim 2022	4.17	0.82	13	3.74	0.64	13	0.9%	0.43 [-0.14, 1.00]
Lee 2010(1)	4.55	0.48	20	4.46	0.88	9	0.9%	0.09 [-0.52, 0.70]
Lee 2010(2)	4.52	0.89	16	4.46	0.88	9	0.7%	0.06 [-0.66, 0.78]
Murphy 2009	4.16	0.82	23	4.11	0.5	12	1.2%	0.05 [-0.39, 0.49]
Park 2007	4.16	0.62	19	4.54	0.74	21	1.2%	-0.38 [-0.80, 0.04]
Salus(2) 2022	3.99	0.37	14	4.64	0.75	14	1.2%	-0.65 [-1.09, -0.21]
Savoye 2007	4.16	0.84	75	4.34	0.84	44	1.5%	-0.18 [-0.49, 0.13]
Savoye 2011	4.2	0.76	75	4.25	0.81	44	1.5%	-0.05 [-0.34, 0.24]
Seo 2012	4.39	2.54	10	4.05	1.79	10	0.1%	0.34 [-1.59, 2.27]
Shalitin 2009	4.28	0.81	55	4.17	0.88	55	1.5%	0.11 [-0.21, 0.43]
Staiano 2017	4.08	0.38	20	4.21	0.63	18	1.4%	-0.13 [-0.47, 0.21]
Staiano 2018	3.8	0.95	22	4.49	0.98	23	1.0%	-0.69 [-1.25, -0.13]
Tas 2023	3.78	0.7	31	3.82	0.7	6	0.9%	-0.04 [-0.65, 0.57]
Tsang 2009	4.1	0.83	11	3.81	0.85	8	0.7%	0.29 [-0.48, 1.06]
Wong 2008	4.7	1	12	4.5	0.7	12	0.8%	0.20 [-0.49, 0.89]
Woo 2012	4.07	0.83	10	4.76	0.6	10	0.8%	-0.69 [-1.32, -0.06]
**Subtotal (95% CI)**			**866**			**632**	**34.1%**	**-0.14 [-0.24, -0.05]**
Heterogeneity: Tau² = 0.02; Chi² = 42.56, df = 31 (P = 0.08); I² = 27%								
Test for overall effect: Z = 3.00 (P = 0.003)								
**Total (95% CI)**			**2370**			**1882**	**100.0%**	**-0.35 [-0.43, -0.27]**
Heterogeneity: Tau² = 0.08; Chi² = 452.93, df = 88 (P < 0.00001); I² = 81%								
Test for overall effect: Z = 8.92 (P < 0.00001)								
Test for subgroup differences: Chi² = 21.14, df = 2 (P < 0.0001), I² = 90.5%								

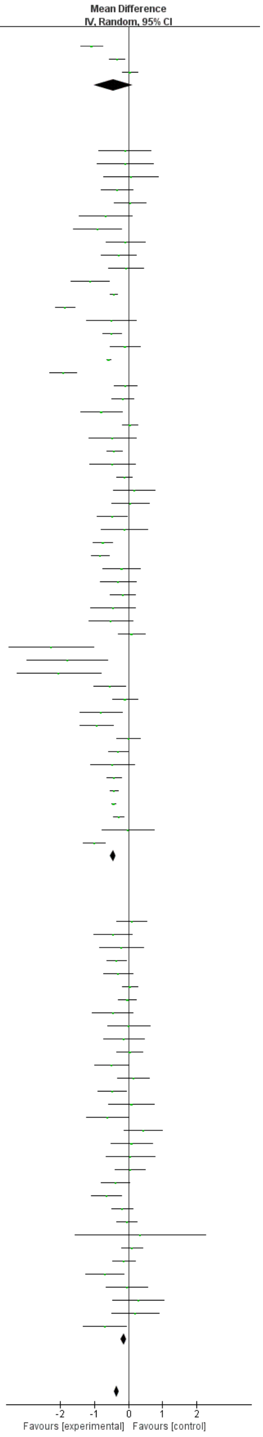

Figure S11.9. Forest plot of subgroup analyses by weight status

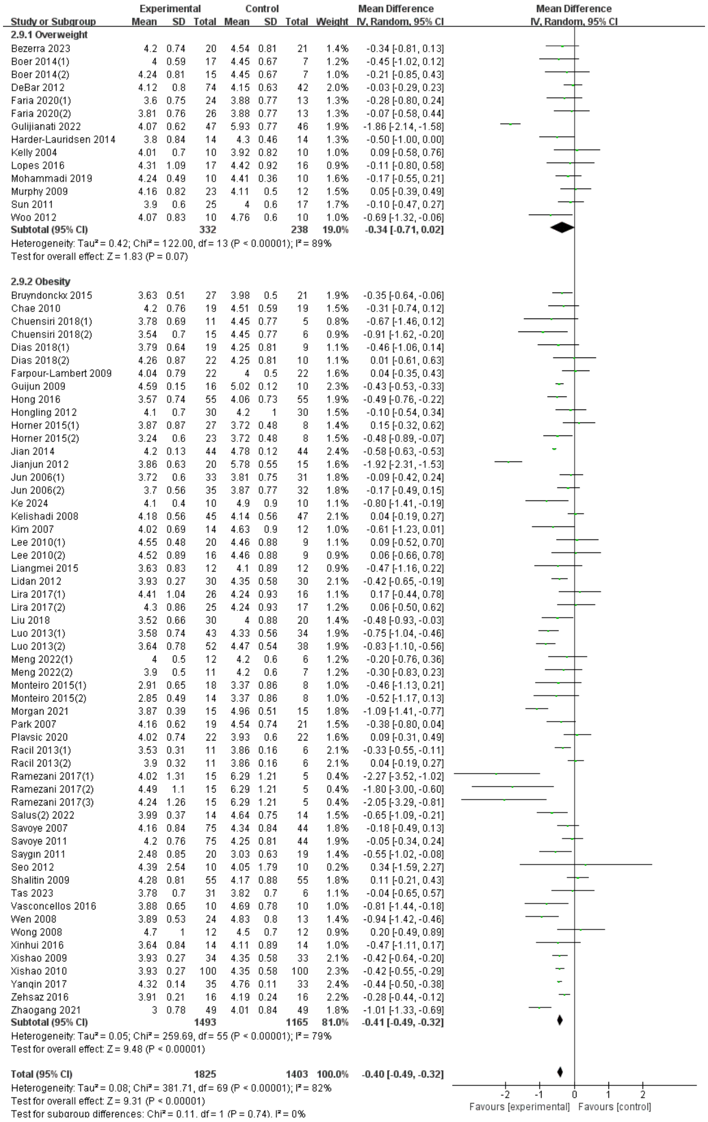

Figure S11.10. Forest plot of subgroup analyses by sex

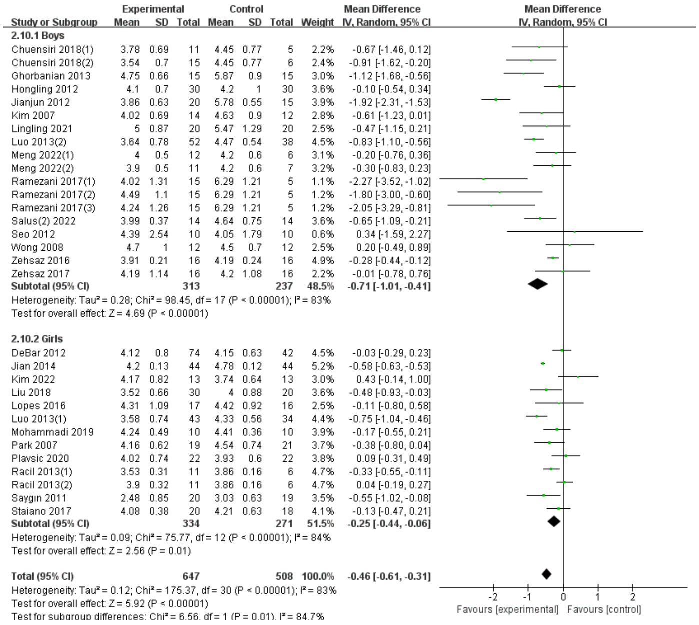

Figure S11.11. Forest plot of subgroup analyses by metabolic status

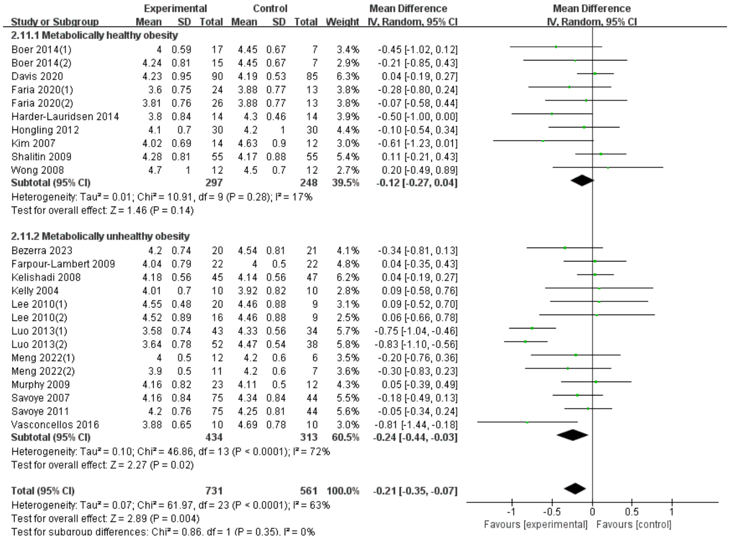

Figure S12. Forest plots of subgroup analyses for TG in children and adolescents with overweight or obesity

Figure S12.1. Forest plot of subgroup analyses by type of exercise

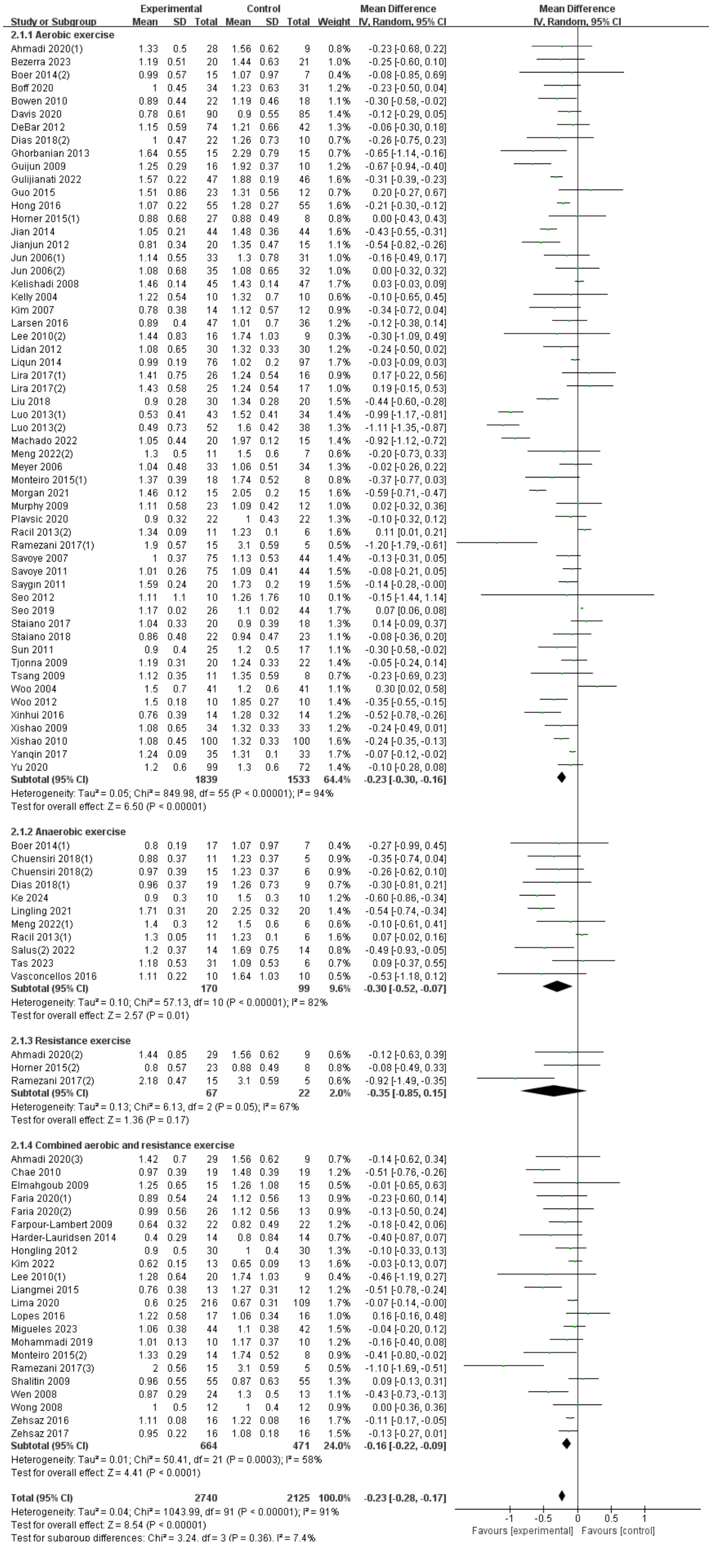

Figure S12.2. Forest plot of subgroup analyses by exercise intensity

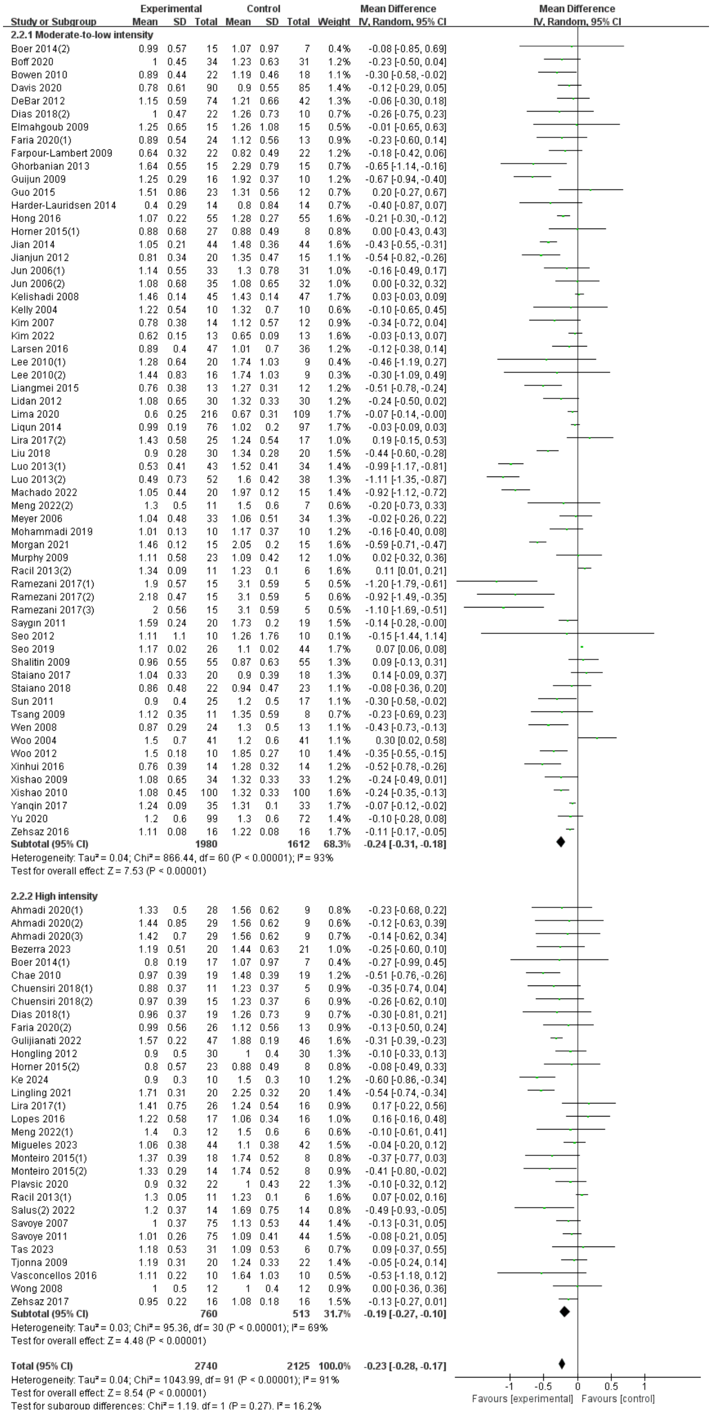

Figure S12.3. Forest plot of subgroup analyses by duration per session

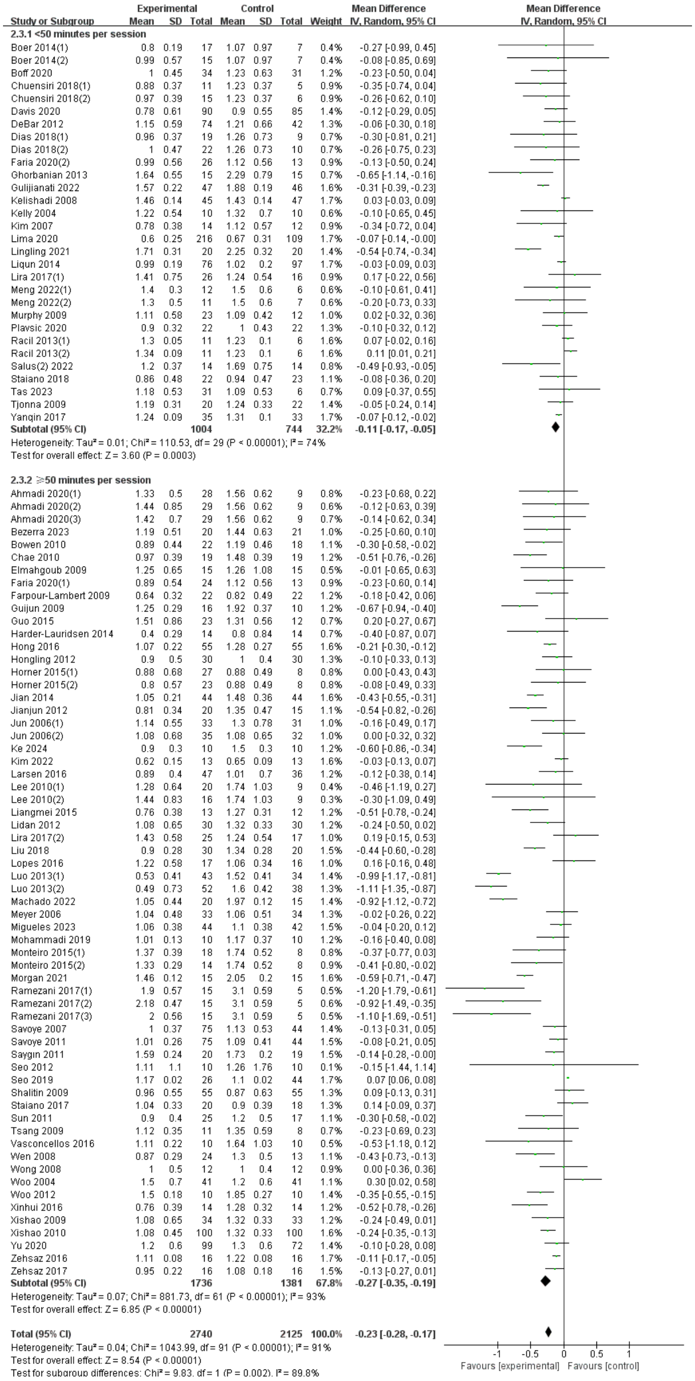

Figure S12.4. Forest plot of subgroup analyses by exercise frequency

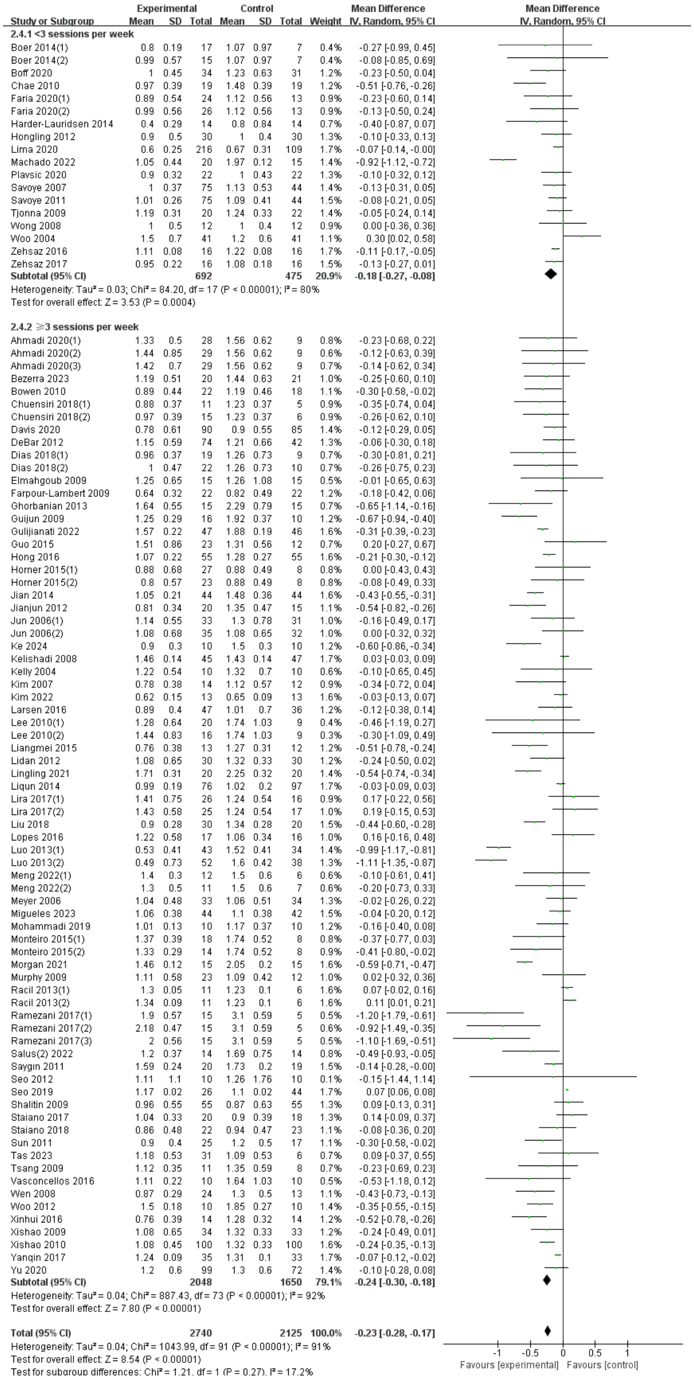

Figure S12.5. Forest plot of subgroup analyses by weekly exercise duration

Study or Subgroup	Experimental Mean	Experimental SD	Experimental Total	Control Mean	Control SD	Control Total	Weight	Mean Difference IV, Random, 95% CI
**2.5.1 <150 minutes per week**								
Boer 2014(1)	0.8	0.19	17	1.07	0.97	7	0.4%	-0.27 [-0.99, 0.45]
Boer 2014(2)	0.99	0.57	15	1.07	0.97	7	0.4%	-0.08 [-0.85, 0.69]
Boff 2020	1	0.45	34	1.23	0.63	31	1.2%	-0.23 [-0.50, 0.04]
Chuensiri 2018(1)	0.88	0.37	11	1.23	0.37	5	0.9%	-0.35 [-0.74, 0.04]
Chuensiri 2018(2)	0.97	0.39	15	1.23	0.37	6	0.9%	-0.26 [-0.62, 0.10]
Dias 2018(1)	0.96	0.37	19	1.26	0.73	9	0.7%	-0.30 [-0.81, 0.21]
Dias 2018(2)	1	0.47	22	1.26	0.73	10	0.7%	-0.26 [-0.75, 0.23]
Faria 2020(1)	0.89	0.54	24	1.12	0.56	13	0.9%	-0.23 [-0.60, 0.14]
Faria 2020(2)	0.99	0.56	26	1.12	0.56	13	0.9%	-0.13 [-0.50, 0.24]
Harder-Lauridsen 2014	0.4	0.29	14	0.8	0.84	14	0.7%	-0.40 [-0.87, 0.07]
Hongling 2012	0.9	0.5	30	1	0.4	30	1.3%	-0.10 [-0.33, 0.13]
Lima 2020	0.6	0.25	216	0.67	0.31	109	1.7%	-0.07 [-0.14, -0.00]
Lingling 2021	1.71	0.31	20	2.25	0.32	20	1.4%	-0.54 [-0.74, -0.34]
Liqun 2014	0.99	0.19	76	1.02	0.2	97	1.7%	-0.03 [-0.09, 0.03]
Lira 2017(1)	1.41	0.75	26	1.24	0.54	16	0.9%	0.17 [-0.22, 0.56]
Machado 2022	1.05	0.44	20	1.97	0.12	15	1.4%	-0.92 [-1.12, -0.72]
Meng 2022(1)	1.4	0.3	12	1.5	0.6	6	0.6%	-0.10 [-0.61, 0.41]
Meng 2022(2)	1.3	0.5	11	1.5	0.6	7	0.6%	-0.20 [-0.73, 0.33]
Murphy 2009	1.11	0.58	23	1.09	0.42	12	1.0%	0.02 [-0.32, 0.36]
Plavsic 2020	0.9	0.32	22	1	0.43	22	1.3%	-0.10 [-0.32, 0.12]
Racil 2013(1)	1.3	0.05	11	1.23	0.1	6	1.6%	0.07 [-0.02, 0.16]
Racil 2013(2)	1.34	0.09	11	1.23	0.1	6	1.6%	0.11 [0.01, 0.21]
Salus(2) 2022	1.2	0.37	14	1.69	0.75	14	0.8%	-0.49 [-0.93, -0.05]
Savoye 2007	1	0.37	75	1.13	0.53	44	1.4%	-0.13 [-0.31, 0.05]
Savoye 2011	1.01	0.26	75	1.09	0.41	44	1.5%	-0.08 [-0.21, 0.05]
Staiano 2018	0.86	0.48	22	0.94	0.47	23	1.1%	-0.08 [-0.36, 0.20]
Tas 2023	1.18	0.53	31	1.09	0.53	6	0.7%	0.09 [-0.37, 0.55]
Tjonna 2009	1.19	0.31	20	1.24	0.33	22	1.4%	-0.05 [-0.24, 0.14]
Wong 2008	1	0.5	12	1	0.4	12	0.9%	0.00 [-0.36, 0.36]
Zehsaz 2017	0.95	0.22	16	1.08	0.18	16	1.5%	-0.13 [-0.27, 0.01]
**Subtotal (95% CI)**			**940**			**642**	**32.0%**	**-0.15 [-0.23, -0.07]**
Heterogeneity: Tau² = 0.03; Chi² = 132.06, df = 29 (P < 0.00001); I² = 78%								
Test for overall effect: Z = 3.77 (P = 0.0002)								
**2.5.2 ≥150 minutes per week**								
Ahmadi 2020(1)	1.33	0.5	28	1.56	0.62	9	0.8%	-0.23 [-0.68, 0.22]
Ahmadi 2020(2)	1.44	0.85	29	1.56	0.62	9	0.6%	-0.12 [-0.63, 0.39]
Ahmadi 2020(3)	1.42	0.7	29	1.56	0.62	9	0.7%	-0.14 [-0.62, 0.34]
Bezerra 2023	1.19	0.51	20	1.44	0.63	21	1.0%	-0.25 [-0.60, 0.10]
Bowen 2010	0.89	0.44	22	1.19	0.46	18	1.1%	-0.30 [-0.58, -0.02]
Chae 2010	0.97	0.39	19	1.48	0.39	19	1.2%	-0.51 [-0.76, -0.26]
Davis 2020	0.78	0.61	90	0.9	0.55	85	1.4%	-0.12 [-0.29, 0.05]
DeBar 2012	1.15	0.59	74	1.21	0.66	42	1.3%	-0.06 [-0.30, 0.18]
Elmahgoub 2009	1.25	0.65	15	1.26	1.08	15	0.5%	-0.01 [-0.65, 0.63]
Farpour-Lambert 2009	0.64	0.32	22	0.82	0.49	22	1.2%	-0.18 [-0.42, 0.06]
Ghorbanian 2013	1.64	0.55	15	2.29	0.79	15	0.7%	-0.65 [-1.14, -0.16]
Guijun 2009	1.25	0.29	16	1.92	0.37	10	1.2%	-0.67 [-0.94, -0.40]
Gulijianati 2022	1.57	0.22	47	1.88	0.19	46	1.6%	-0.31 [-0.39, -0.23]
Guo 2015	1.51	0.86	23	1.31	0.56	12	0.7%	0.20 [-0.27, 0.67]
Hong 2016	1.07	0.22	55	1.28	0.27	55	1.6%	-0.21 [-0.30, -0.12]
Horner 2015(1)	0.88	0.68	27	0.88	0.49	8	0.8%	0.00 [-0.43, 0.43]
Horner 2015(2)	0.8	0.57	23	0.88	0.49	8	0.8%	-0.08 [-0.49, 0.33]
Jian 2014	1.05	0.21	44	1.48	0.36	44	1.6%	-0.43 [-0.55, -0.31]
Jianjun 2012	0.81	0.34	20	1.35	0.47	15	1.1%	-0.54 [-0.82, -0.26]
Jun 2006(1)	1.14	0.55	33	1.3	0.78	31	1.0%	-0.16 [-0.49, 0.17]
Jun 2006(2)	1.08	0.68	35	1.08	0.65	32	1.0%	0.00 [-0.32, 0.32]
Ke 2024	0.9	0.3	10	1.5	0.3	10	1.2%	-0.60 [-0.86, -0.34]
Kelishadi 2008	1.46	0.14	45	1.43	0.14	47	1.7%	0.03 [-0.03, 0.09]
Kelly 2004	1.22	0.54	10	1.32	0.7	10	0.6%	-0.10 [-0.65, 0.45]
Kim 2007	0.78	0.38	14	1.12	0.57	12	0.9%	-0.34 [-0.72, 0.04]
Kim 2022	0.62	0.15	13	0.65	0.09	13	1.6%	-0.03 [-0.13, 0.07]
Larsen 2016	0.89	0.4	47	1.01	0.7	36	1.2%	-0.12 [-0.38, 0.14]
Lee 2010(1)	1.28	0.64	20	1.74	1.03	9	0.4%	-0.46 [-1.19, 0.27]
Lee 2010(2)	1.44	0.83	16	1.74	1.03	9	0.3%	-0.30 [-1.09, 0.49]
Liangmei 2015	0.76	0.38	13	1.27	0.31	12	1.2%	-0.51 [-0.78, -0.24]
Lidan 2012	1.08	0.65	30	1.32	0.33	30	1.2%	-0.24 [-0.50, 0.02]
Lira 2017(2)	1.43	0.58	25	1.24	0.54	17	1.0%	0.19 [-0.15, 0.53]
Liu 2018	0.9	0.28	30	1.34	0.28	20	1.5%	-0.44 [-0.60, -0.28]
Lopes 2016	1.22	0.58	17	1.06	0.34	16	1.0%	0.16 [-0.16, 0.48]
Luo 2013(1)	0.53	0.41	43	1.52	0.41	34	1.4%	-0.99 [-1.17, -0.81]
Luo 2013(2)	0.49	0.73	52	1.6	0.42	38	1.3%	-1.11 [-1.35, -0.87]
Meyer 2006	1.04	0.48	33	1.06	0.51	34	1.3%	-0.02 [-0.26, 0.22]
Migueles 2023	1.06	0.38	44	1.1	0.38	42	1.5%	-0.04 [-0.20, 0.12]
Mohammadi 2019	1.01	0.13	10	1.17	0.37	10	1.2%	-0.16 [-0.40, 0.08]
Monteiro 2015(1)	1.37	0.39	18	1.74	0.52	8	0.8%	-0.37 [-0.77, 0.03]
Monteiro 2015(2)	1.33	0.29	14	1.74	0.52	8	0.9%	-0.41 [-0.80, -0.02]
Morgan 2021	1.46	0.12	15	2.05	0.2	15	1.6%	-0.59 [-0.71, -0.47]
Ramezani 2017(1)	1.9	0.57	15	3.1	0.59	5	0.5%	-1.20 [-1.79, -0.61]
Ramezani 2017(2)	2.18	0.47	15	3.1	0.59	5	0.6%	-0.92 [-1.49, -0.35]
Ramezani 2017(3)	2	0.56	15	3.1	0.59	5	0.5%	-1.10 [-1.69, -0.51]
Saygın 2011	1.59	0.24	20	1.73	0.2	19	1.5%	-0.14 [-0.28, -0.00]
Seo 2012	1.11	1.1	10	1.26	1.76	10	0.1%	-0.15 [-1.44, 1.14]
Seo 2019	1.17	0.02	26	1.1	0.02	44	1.7%	0.07 [0.06, 0.08]
Shalitin 2009	0.96	0.55	55	0.87	0.63	55	1.3%	0.09 [-0.13, 0.31]
Staiano 2017	1.04	0.33	20	0.9	0.39	18	1.3%	0.14 [-0.09, 0.37]
Sun 2011	0.9	0.4	25	1.2	0.5	17	1.1%	-0.30 [-0.58, -0.02]
Tsang 2009	1.12	0.35	11	1.35	0.59	8	0.7%	-0.23 [-0.69, 0.23]
Vasconcellos 2016	1.11	0.22	10	1.64	1.03	10	0.5%	-0.53 [-1.18, 0.12]
Wen 2008	0.87	0.29	24	1.3	0.5	13	1.1%	-0.43 [-0.73, -0.13]
Woo 2004	1.5	0.7	41	1.2	0.6	41	1.1%	0.30 [0.02, 0.58]
Woo 2012	1.5	0.18	10	1.85	0.27	10	1.4%	-0.35 [-0.55, -0.15]
Xinhui 2016	0.76	0.39	14	1.28	0.32	14	1.2%	-0.52 [-0.78, -0.26]
Xishao 2009	1.08	0.65	34	1.32	0.33	33	1.2%	-0.24 [-0.49, 0.01]
Xishao 2010	1.08	0.45	100	1.32	0.33	100	1.6%	-0.24 [-0.35, -0.13]
Yanqin 2017	1.24	0.09	35	1.31	0.1	33	1.7%	-0.07 [-0.12, -0.02]
Yu 2020	1.2	0.6	99	1.3	0.6	72	1.4%	-0.10 [-0.28, 0.08]
Zehsaz 2016	1.11	0.08	16	1.22	0.08	16	1.7%	-0.11 [-0.17, -0.05]
**Subtotal (95% CI)**			**1800**			**1483**	**68.0%**	**-0.26 [-0.33, -0.19]**
Heterogeneity: Tau² = 0.05; Chi² = 876.12, df = 61 (P < 0.00001); I² = 93%								
Test for overall effect: Z = 7.54 (P < 0.00001)								
**Total (95% CI)**			**2740**			**2125**	**100.0%**	**-0.23 [-0.28, -0.17]**
Heterogeneity: Tau² = 0.04; Chi² = 1043.99, df = 91 (P < 0.00001); I² = 91%								
Test for overall effect: Z = 8.54 (P < 0.00001)								
Test for subgroup differences: Chi² = 3.87, df = 1 (P = 0.05), I² = 74.1%								

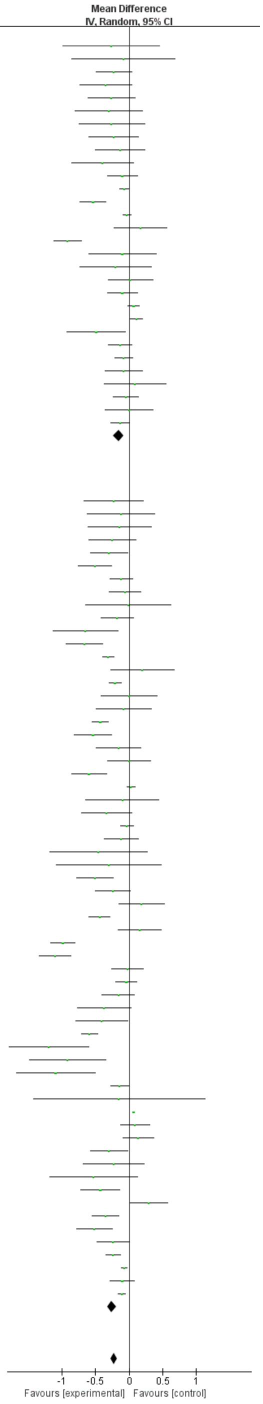

Figure S12.6. Forest plot of subgroup analyses by intervention duration

Study or Subgroup	Experimental Mean	Experimental SD	Experimental Total	Control Mean	Control SD	Control Total	Weight	Mean Difference IV, Random, 95% CI
**2.6.1 <12 weeks (total intervention duration)**								
Ahmadi 2020(1)	1.33	0.5	28	1.56	0.62	9	0.8%	-0.23 [-0.68, 0.22]
Ahmadi 2020(2)	1.44	0.85	29	1.56	0.62	9	0.6%	-0.12 [-0.63, 0.39]
Ahmadi 2020(3)	1.42	0.7	29	1.56	0.62	9	0.7%	-0.14 [-0.62, 0.34]
Bezerra 2023	1.19	0.51	20	1.44	0.63	21	1.0%	-0.25 [-0.60, 0.10]
Bowen 2010	0.89	0.44	22	1.19	0.46	18	1.1%	-0.30 [-0.58, -0.02]
Elmahgoub 2009	1.25	0.65	15	1.26	1.08	15	0.5%	-0.01 [-0.65, 0.63]
Ghorbanian 2013	1.64	0.55	15	2.29	0.79	15	0.7%	-0.65 [-1.14, -0.16]
Hongling 2012	0.9	0.5	30	1	0.4	30	1.3%	-0.10 [-0.33, 0.13]
Kelly 2004	1.22	0.54	10	1.32	0.7	10	0.6%	-0.10 [-0.65, 0.45]
Kim 2007	0.78	0.38	14	1.12	0.57	12	0.9%	-0.34 [-0.72, 0.04]
Larsen 2016	0.89	0.4	47	1.01	0.7	36	1.2%	-0.12 [-0.38, 0.14]
Lee 2010(1)	1.28	0.64	20	1.74	1.03	9	0.4%	-0.46 [-1.19, 0.27]
Lee 2010(2)	1.44	0.83	16	1.74	1.03	9	0.3%	-0.30 [-1.09, 0.49]
Liu 2018	0.9	0.28	30	1.34	0.28	20	1.5%	-0.44 [-0.60, -0.28]
Luo 2013(1)	0.53	0.41	43	1.52	0.41	34	1.4%	-0.99 [-1.17, -0.81]
Luo 2013(2)	0.49	0.73	52	1.6	0.42	38	1.3%	-1.11 [-1.35, -0.87]
Mohammadi 2019	1.01	0.13	10	1.17	0.37	10	1.2%	-0.16 [-0.40, 0.08]
Ramezani 2017(1)	1.9	0.57	15	3.1	0.59	5	0.5%	-1.20 [-1.79, -0.61]
Ramezani 2017(2)	2.18	0.47	15	3.1	0.59	5	0.6%	-0.92 [-1.49, -0.35]
Ramezani 2017(3)	2	0.56	15	3.1	0.59	5	0.5%	-1.10 [-1.69, -0.51]
Seo 2012	1.11	1.1	10	1.26	1.76	10	0.1%	-0.15 [-1.44, 1.14]
Sun 2011	0.9	0.4	25	1.2	0.5	17	1.1%	-0.30 [-0.58, -0.02]
Tas 2023	1.18	0.53	31	1.09	0.53	6	0.7%	0.09 [-0.37, 0.55]
Woo 2004	1.5	0.7	41	1.2	0.6	41	1.1%	0.30 [0.02, 0.58]
**Subtotal (95% CI)**			**582**			**393**	**20.2%**	**-0.38 [-0.55, -0.20]**
Heterogeneity: Tau² = 0.14; Chi² = 140.12, df = 23 (P < 0.00001); I² = 84%								
Test for overall effect: Z = 4.19 (P < 0.0001)								
**2.6.2 ≥12 weeks (total intervention duration)**								
Boer 2014(1)	0.8	0.19	17	1.07	0.97	7	0.4%	-0.27 [-0.99, 0.45]
Boer 2014(2)	0.99	0.57	15	1.07	0.97	7	0.4%	-0.08 [-0.85, 0.69]
Boff 2020	1	0.45	34	1.23	0.63	31	1.2%	-0.23 [-0.50, 0.04]
Chae 2010	0.97	0.39	19	1.48	0.39	19	1.2%	-0.51 [-0.76, -0.26]
Chuensiri 2018(1)	0.88	0.37	11	1.23	0.37	5	0.9%	-0.35 [-0.74, 0.04]
Chuensiri 2018(2)	0.97	0.39	15	1.23	0.37	6	0.9%	-0.26 [-0.62, 0.10]
Davis 2020	0.78	0.61	90	0.9	0.55	85	1.4%	-0.12 [-0.29, 0.05]
DeBar 2012	1.15	0.59	74	1.21	0.66	42	1.3%	-0.06 [-0.30, 0.18]
Dias 2018(1)	0.96	0.37	19	1.26	0.73	9	0.7%	-0.30 [-0.81, 0.21]
Dias 2018(2)	1	0.47	22	1.26	0.73	10	0.7%	-0.26 [-0.75, 0.23]
Faria 2020(1)	0.89	0.54	24	1.12	0.56	13	0.9%	-0.23 [-0.60, 0.14]
Faria 2020(2)	0.99	0.56	26	1.12	0.56	13	0.9%	-0.13 [-0.50, 0.24]
Farpour-Lambert 2009	0.64	0.32	22	0.82	0.49	22	1.2%	-0.18 [-0.42, 0.06]
Guijun 2009	1.25	0.29	16	1.92	0.37	10	1.2%	-0.67 [-0.94, -0.40]
Gulijianati 2022	1.57	0.22	47	1.88	0.19	46	1.6%	-0.31 [-0.39, -0.23]
Guo 2015	1.51	0.86	23	1.31	0.56	12	0.7%	0.20 [-0.27, 0.67]
Harder-Lauridsen 2014	0.4	0.29	14	0.8	0.84	14	0.7%	-0.40 [-0.87, 0.07]
Hong 2016	1.07	0.22	55	1.28	0.27	55	1.6%	-0.21 [-0.30, -0.12]
Horner 2015(1)	0.88	0.68	27	0.88	0.49	8	0.8%	0.00 [-0.43, 0.43]
Horner 2015(2)	0.8	0.57	23	0.88	0.49	8	0.8%	-0.08 [-0.49, 0.33]
Jian 2014	1.05	0.21	44	1.48	0.36	44	1.6%	-0.43 [-0.55, -0.31]
Jianjun 2012	0.81	0.34	20	1.35	0.47	15	1.1%	-0.54 [-0.82, -0.26]
Jun 2006(1)	1.14	0.55	33	1.3	0.78	31	1.0%	-0.16 [-0.49, 0.17]
Jun 2006(2)	1.08	0.68	35	1.08	0.65	32	1.0%	0.00 [-0.32, 0.32]
Ke 2024	0.9	0.3	10	1.5	0.3	10	1.2%	-0.60 [-0.86, -0.34]
Kelishadi 2008	1.46	0.14	45	1.43	0.14	47	1.7%	0.03 [-0.03, 0.09]
Kim 2022	0.62	0.15	13	0.65	0.09	13	1.6%	-0.03 [-0.13, 0.07]
Liangmei 2015	0.76	0.38	13	1.27	0.31	12	1.2%	-0.51 [-0.78, -0.24]
Lidan 2012	1.08	0.65	30	1.32	0.33	30	1.2%	-0.24 [-0.50, 0.02]
Lima 2020	0.6	0.25	216	0.67	0.31	109	1.7%	-0.07 [-0.14, -0.00]
Lingling 2021	1.71	0.31	20	2.25	0.32	20	1.4%	-0.54 [-0.74, -0.34]
Liqun 2014	0.99	0.19	76	1.02	0.2	97	1.7%	-0.03 [-0.09, 0.03]
Lira 2017(1)	1.41	0.75	26	1.24	0.54	16	0.9%	0.17 [-0.22, 0.56]
Lira 2017(2)	1.43	0.58	25	1.24	0.54	17	1.0%	0.19 [-0.15, 0.53]
Lopes 2016	1.22	0.58	17	1.06	0.34	16	1.0%	0.16 [-0.16, 0.48]
Machado 2022	1.05	0.44	20	1.97	0.12	15	1.4%	-0.92 [-1.12, -0.72]
Meng 2022(1)	1.4	0.3	12	1.5	0.6	6	0.6%	-0.10 [-0.61, 0.41]
Meng 2022(2)	1.3	0.5	11	1.5	0.6	7	0.6%	-0.20 [-0.73, 0.33]
Meyer 2006	1.04	0.48	33	1.06	0.51	34	1.3%	-0.02 [-0.26, 0.22]
Migueles 2023	1.06	0.38	44	1.1	0.38	42	1.5%	-0.04 [-0.20, 0.12]
Monteiro 2015(1)	1.37	0.39	18	1.74	0.52	8	0.8%	-0.37 [-0.77, 0.03]
Monteiro 2015(2)	1.33	0.29	14	1.74	0.52	8	0.9%	-0.41 [-0.80, -0.02]
Morgan 2021	1.46	0.12	15	2.05	0.2	15	1.6%	-0.59 [-0.71, -0.47]
Murphy 2009	1.11	0.58	23	1.09	0.42	12	1.0%	0.02 [-0.32, 0.36]
Plavsic 2020	0.9	0.32	22	1	0.43	22	1.3%	-0.10 [-0.32, 0.12]
Racil 2013(1)	1.3	0.05	11	1.23	0.1	6	1.6%	0.07 [-0.02, 0.16]
Racil 2013(2)	1.34	0.09	11	1.23	0.1	6	1.6%	0.11 [0.01, 0.21]
Salus(2) 2022	1.2	0.37	14	1.69	0.75	14	0.8%	-0.49 [-0.93, -0.05]
Savoye 2007	1	0.37	75	1.13	0.53	44	1.4%	-0.13 [-0.31, 0.05]
Savoye 2011	1.01	0.26	75	1.09	0.41	44	1.5%	-0.08 [-0.21, 0.05]
Saygın 2011	1.59	0.24	20	1.73	0.2	19	1.5%	-0.14 [-0.28, -0.00]
Seo 2019	1.17	0.02	26	1.1	0.02	44	1.7%	0.07 [0.06, 0.08]
Shalitin 2009	0.96	0.55	55	0.87	0.63	55	1.3%	0.09 [-0.13, 0.31]
Staiano 2017	1.04	0.33	20	0.9	0.39	18	1.3%	0.14 [-0.09, 0.37]
Staiano 2018	0.86	0.48	22	0.94	0.47	23	1.1%	-0.08 [-0.36, 0.20]
Tjonna 2009	1.19	0.31	20	1.24	0.33	22	1.4%	-0.05 [-0.24, 0.14]
Tsang 2009	1.12	0.35	11	1.35	0.59	8	0.7%	-0.23 [-0.69, 0.23]
Vasconcellos 2016	1.11	0.22	10	1.64	1.03	10	0.5%	-0.53 [-1.18, 0.12]
Wen 2008	0.87	0.29	24	1.3	0.5	13	1.1%	-0.43 [-0.73, -0.13]
Wong 2008	1	0.5	12	1	0.4	12	0.9%	0.00 [-0.36, 0.36]
Woo 2012	1.5	0.18	10	1.85	0.27	10	1.4%	-0.35 [-0.55, -0.15]
Xinhui 2016	0.76	0.39	14	1.28	0.32	14	1.2%	-0.52 [-0.78, -0.26]
Xishao 2009	1.08	0.65	34	1.32	0.33	33	1.2%	-0.24 [-0.49, 0.01]
Xishao 2010	1.08	0.45	100	1.32	0.33	100	1.6%	-0.24 [-0.35, -0.13]
Yanqin 2017	1.24	0.09	35	1.31	0.1	33	1.7%	-0.07 [-0.12, -0.02]
Yu 2020	1.2	0.6	99	1.3	0.6	72	1.4%	-0.10 [-0.28, 0.08]
Zehsaz 2016	1.11	0.08	16	1.22	0.08	16	1.7%	-0.11 [-0.17, -0.05]
Zehsaz 2017	0.95	0.22	16	1.08	0.18	16	1.5%	-0.13 [-0.27, 0.01]
**Subtotal (95% CI)**			**2158**			**1732**	**79.8%**	**-0.18 [-0.24, -0.13]**
Heterogeneity: Tau² = 0.03; Chi² = 733.03, df = 67 (P < 0.00001); I² = 91%								
Test for overall effect: Z = 6.98 (P < 0.00001)								
**Total (95% CI)**			**2740**			**2125**	**100.0%**	**-0.23 [-0.28, -0.17]**
Heterogeneity: Tau² = 0.04; Chi² = 1043.99, df = 91 (P < 0.00001); I² = 91%								
Test for overall effect: Z = 8.54 (P < 0.00001)								
Test for subgroup differences: Chi² = 4.21, df = 1 (P = 0.04), I² = 76.3%								

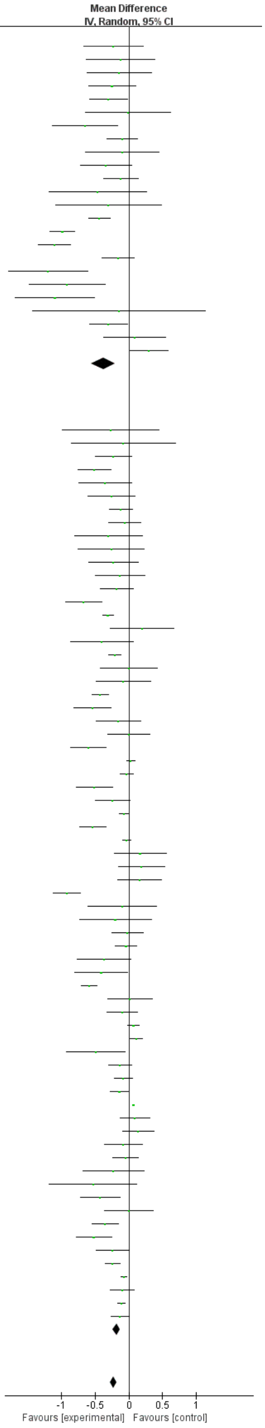

Figure S12.7. Forest plot of subgroup analyses by region

Study or Subgroup	Experimental Mean	Experimental SD	Experimental Total	Control Mean	Control SD	Control Total	Weight	Mean Difference IV, Random, 95% CI
**2.7.1 Asia**								
Ahmadi 2020(1)	1.33	0.5	28	1.56	0.62	9	0.8%	-0.23 [-0.68, 0.22]
Ahmadi 2020(2)	1.44	0.85	29	1.56	0.62	9	0.6%	-0.12 [-0.63, 0.39]
Ahmadi 2020(3)	1.42	0.7	29	1.56	0.62	9	0.7%	-0.14 [-0.62, 0.34]
Bowen 2010	0.89	0.44	22	1.19	0.46	18	1.1%	-0.30 [-0.58, -0.02]
Chae 2010	0.97	0.39	19	1.48	0.39	19	1.2%	-0.51 [-0.76, -0.26]
Chuensiri 2018(1)	0.88	0.37	11	1.23	0.37	5	0.9%	-0.35 [-0.74, 0.04]
Chuensiri 2018(2)	0.97	0.39	15	1.23	0.37	6	0.9%	-0.26 [-0.62, 0.10]
Ghorbanian 2013	1.64	0.55	15	2.29	0.79	15	0.7%	-0.65 [-1.14, -0.16]
Guijun 2009	1.25	0.29	16	1.92	0.37	10	1.2%	-0.67 [-0.94, -0.40]
Gulijianati 2022	1.57	0.22	47	1.88	0.19	46	1.6%	-0.31 [-0.39, -0.23]
Guo 2015	1.51	0.86	23	1.31	0.56	12	0.7%	0.20 [-0.27, 0.67]
Hong 2016	1.07	0.22	55	1.28	0.27	55	1.6%	-0.21 [-0.30, -0.12]
Hongling 2012	0.9	0.5	30	1	0.4	30	1.3%	-0.10 [-0.33, 0.13]
Jian 2014	1.05	0.21	44	1.48	0.36	44	1.6%	-0.43 [-0.55, -0.31]
Jianjun 2012	0.81	0.34	20	1.35	0.47	15	1.1%	-0.54 [-0.82, -0.26]
Jun 2006(1)	1.14	0.55	33	1.3	0.78	31	1.0%	-0.16 [-0.49, 0.17]
Jun 2006(2)	1.08	0.68	35	1.08	0.65	32	1.0%	0.00 [-0.32, 0.32]
Ke 2024	0.9	0.3	10	1.5	0.3	10	1.2%	-0.60 [-0.86, -0.34]
Kelishadi 2008	1.46	0.14	45	1.43	0.14	47	1.7%	0.03 [-0.03, 0.09]
Kim 2007	0.78	0.38	14	1.12	0.57	12	0.9%	-0.34 [-0.72, 0.04]
Kim 2022	0.62	0.15	13	0.65	0.09	13	1.6%	-0.03 [-0.13, 0.07]
Lee 2010(1)	1.28	0.64	20	1.74	1.03	9	0.4%	-0.46 [-1.19, 0.27]
Lee 2010(2)	1.44	0.83	16	1.74	1.03	9	0.3%	-0.30 [-1.09, 0.49]
Liangmei 2015	0.76	0.38	13	1.27	0.31	12	1.2%	-0.51 [-0.78, -0.24]
Lidan 2012	1.08	0.65	30	1.32	0.33	30	1.2%	-0.24 [-0.50, 0.02]
Lingling 2021	1.71	0.31	20	2.25	0.32	20	1.4%	-0.54 [-0.74, -0.34]
Liqun 2014	0.99	0.19	76	1.02	0.2	97	1.7%	-0.03 [-0.09, 0.03]
Liu 2018	0.9	0.28	30	1.34	0.28	20	1.5%	-0.44 [-0.60, -0.28]
Luo 2013(1)	0.53	0.41	43	1.52	0.41	34	1.4%	-0.99 [-1.17, -0.81]
Luo 2013(2)	0.49	0.73	52	1.6	0.42	38	1.3%	-1.11 [-1.35, -0.87]
Meng 2022(1)	1.4	0.3	12	1.5	0.6	6	0.6%	-0.10 [-0.61, 0.41]
Meng 2022(2)	1.3	0.5	11	1.5	0.6	7	0.6%	-0.20 [-0.73, 0.33]
Mohammadi 2019	1.01	0.13	10	1.17	0.37	10	1.2%	-0.16 [-0.40, 0.08]
Ramezani 2017(1)	1.9	0.57	15	3.1	0.59	5	0.5%	-1.20 [-1.79, -0.61]
Ramezani 2017(2)	2.18	0.47	15	3.1	0.59	5	0.6%	-0.92 [-1.49, -0.35]
Ramezani 2017(3)	2	0.56	15	3.1	0.59	5	0.5%	-1.10 [-1.69, -0.51]
Saygın 2011	1.59	0.24	20	1.73	0.2	19	1.5%	-0.14 [-0.28, -0.00]
Seo 2012	1.11	1.1	10	1.26	1.76	10	0.1%	-0.15 [-1.44, 1.14]
Seo 2019	1.17	0.02	26	1.1	0.02	44	1.7%	0.07 [0.06, 0.08]
Shalitin 2009	0.96	0.55	55	0.87	0.63	55	1.3%	0.09 [-0.13, 0.31]
Sun 2011	0.9	0.4	25	1.2	0.5	17	1.1%	-0.30 [-0.58, -0.02]
Wen 2008	0.87	0.29	24	1.3	0.5	13	1.1%	-0.43 [-0.73, -0.13]
Wong 2008	1	0.5	12	1	0.4	12	0.9%	0.00 [-0.36, 0.36]
Woo 2004	1.5	0.7	41	1.2	0.6	41	1.1%	0.30 [0.02, 0.58]
Woo 2012	1.5	0.18	10	1.85	0.27	10	1.4%	-0.35 [-0.55, -0.15]
Xinhui 2016	0.76	0.39	14	1.28	0.32	14	1.2%	-0.52 [-0.78, -0.26]
Xishao 2009	1.08	0.65	34	1.32	0.33	33	1.2%	-0.24 [-0.49, 0.01]
Xishao 2010	1.08	0.45	100	1.32	0.33	100	1.6%	-0.24 [-0.35, -0.13]
Yanqin 2017	1.24	0.09	35	1.31	0.1	33	1.7%	-0.07 [-0.12, -0.02]
Yu 2020	1.2	0.6	99	1.3	0.6	72	1.4%	-0.10 [-0.28, 0.08]
Zehsaz 2016	1.11	0.08	16	1.22	0.08	16	1.7%	-0.11 [-0.17, -0.05]
Zehsaz 2017	0.95	0.22	16	1.08	0.18	16	1.5%	-0.13 [-0.27, 0.01]
**Subtotal (95% CI)**			**1468**			**1259**	**58.6%**	**-0.29 [-0.36, -0.22]**
Heterogeneity: Tau^2 = 0.04; Chi^2 = 794.33, df = 51 (P < 0.00001); I^2 = 94%								
Test for overall effect: Z = 8.17 (P < 0.00001)								
**2.7.2 Europe**								
Boer 2014(1)	0.8	0.19	17	1.07	0.97	7	0.4%	-0.27 [-0.99, 0.45]
Boer 2014(2)	0.99	0.57	15	1.07	0.97	7	0.4%	-0.08 [-0.85, 0.69]
Elmahgoub 2009	1.25	0.65	15	1.26	1.08	15	0.5%	-0.01 [-0.65, 0.63]
Farpour-Lambert 2009	0.64	0.32	22	0.82	0.49	22	1.2%	-0.18 [-0.42, 0.06]
Harder-Lauridsen 2014	0.4	0.29	14	0.8	0.84	14	0.7%	-0.40 [-0.87, 0.07]
Larsen 2016	0.89	0.4	47	1.01	0.7	36	1.2%	-0.12 [-0.38, 0.14]
Lima 2020	0.6	0.25	216	0.67	0.31	109	1.7%	-0.07 [-0.14, -0.00]
Meyer 2006	1.04	0.48	33	1.06	0.51	34	1.3%	-0.02 [-0.26, 0.22]
Migueles 2023	1.06	0.38	44	1.1	0.38	42	1.5%	-0.04 [-0.20, 0.12]
Plavsic 2020	0.9	0.32	22	1	0.43	22	1.3%	-0.10 [-0.32, 0.12]
Salus(2) 2022	1.2	0.37	14	1.69	0.75	14	0.8%	-0.49 [-0.93, -0.05]
Tjonna 2009	1.19	0.31	20	1.24	0.33	22	1.4%	-0.05 [-0.24, 0.14]
**Subtotal (95% CI)**			**479**			**344**	**12.2%**	**-0.08 [-0.13, -0.03]**
Heterogeneity: Tau^2 = 0.00; Chi^2 = 6.91, df = 11 (P = 0.81); I^2 = 0%								
Test for overall effect: Z = 3.12 (P = 0.002)								
**2.7.3 Africa**								
Morgan 2021	1.46	0.12	15	2.05	0.2	15	1.6%	-0.59 [-0.71, -0.47]
Racil 2013(1)	1.3	0.05	11	1.23	0.1	6	1.6%	0.07 [-0.02, 0.16]
Racil 2013(2)	1.34	0.09	11	1.23	0.1	6	1.6%	0.11 [0.01, 0.21]
**Subtotal (95% CI)**			**37**			**27**	**4.8%**	**-0.13 [-0.54, 0.27]**
Heterogeneity: Tau^2 = 0.12; Chi^2 = 98.37, df = 2 (P < 0.00001); I^2 = 98%								
Test for overall effect: Z = 0.66 (P = 0.51)								
**2.7.4 North America**								
Davis 2020	0.78	0.61	90	0.9	0.55	85	1.4%	-0.12 [-0.29, 0.05]
DeBar 2012	1.15	0.59	74	1.21	0.66	42	1.3%	-0.06 [-0.30, 0.18]
Horner 2015(1)	0.88	0.68	27	0.88	0.49	8	0.8%	0.00 [-0.43, 0.43]
Horner 2015(2)	0.8	0.57	23	0.88	0.49	8	0.8%	-0.08 [-0.49, 0.33]
Kelly 2004	1.22	0.54	10	1.32	0.7	10	0.6%	-0.10 [-0.65, 0.45]
Murphy 2009	1.11	0.58	23	1.09	0.42	12	1.0%	0.02 [-0.32, 0.36]
Savoye 2007	1	0.37	75	1.13	0.53	44	1.4%	-0.13 [-0.31, 0.05]
Savoye 2011	1.01	0.26	75	1.09	0.41	44	1.5%	-0.08 [-0.21, 0.05]
Staiano 2017	1.04	0.33	20	0.9	0.39	18	1.3%	0.14 [-0.09, 0.37]
Staiano 2018	0.86	0.48	22	0.94	0.47	23	1.1%	-0.08 [-0.36, 0.20]
Tas 2023	1.18	0.53	31	1.09	0.53	6	0.7%	0.09 [-0.37, 0.55]
**Subtotal (95% CI)**			**470**			**300**	**12.0%**	**-0.06 [-0.13, 0.01]**
Heterogeneity: Tau^2 = 0.00; Chi^2 = 4.77, df = 10 (P = 0.91); I^2 = 0%								
Test for overall effect: Z = 1.71 (P = 0.09)								
**2.7.5 South America**								
Bezerra 2023	1.19	0.51	20	1.44	0.63	21	1.0%	-0.25 [-0.60, 0.10]
Boff 2020	1	0.45	34	1.23	0.63	31	1.2%	-0.23 [-0.50, 0.04]
Faria 2020(1)	0.89	0.54	24	1.12	0.56	13	0.9%	-0.23 [-0.60, 0.14]
Faria 2020(2)	0.99	0.56	26	1.12	0.56	13	0.9%	-0.13 [-0.50, 0.24]
Lira 2017(1)	1.41	0.75	26	1.24	0.54	16	0.9%	0.17 [-0.22, 0.56]
Lira 2017(2)	1.43	0.58	25	1.24	0.54	17	1.0%	0.19 [-0.15, 0.53]
Lopes 2016	1.22	0.58	17	1.06	0.34	16	1.0%	0.16 [-0.16, 0.48]
Machado 2022	1.05	0.44	20	1.97	0.12	15	1.4%	-0.92 [-1.12, -0.72]
Monteiro 2015(1)	1.37	0.39	18	1.74	0.52	8	0.8%	-0.37 [-0.77, 0.03]
Monteiro 2015(2)	1.33	0.29	14	1.74	0.52	8	0.9%	-0.41 [-0.80, -0.02]
Vasconcellos 2016	1.11	0.22	10	1.64	1.03	10	0.5%	-0.53 [-1.18, 0.12]
**Subtotal (95% CI)**			**234**			**168**	**10.3%**	**-0.23 [-0.48, 0.02]**
Heterogeneity: Tau^2 = 0.15; Chi^2 = 59.35, df = 10 (P < 0.00001); I^2 = 83%								
Test for overall effect: Z = 1.80 (P = 0.07)								
**2.7.6 Oceania**								
Dias 2018(1)	0.96	0.37	19	1.26	0.73	9	0.7%	-0.30 [-0.81, 0.21]
Dias 2018(2)	1	0.47	22	1.26	0.73	10	0.7%	-0.26 [-0.75, 0.23]
Tsang 2009	1.12	0.35	11	1.35	0.59	8	0.7%	-0.23 [-0.69, 0.23]
**Subtotal (95% CI)**			**52**			**27**	**2.1%**	**-0.26 [-0.54, 0.02]**
Heterogeneity: Tau^2 = 0.00; Chi^2 = 0.04, df = 2 (P = 0.98); I^2 = 0%								
Test for overall effect: Z = 1.83 (P = 0.07)								
**Total (95% CI)**			**2740**			**2125**	**100.0%**	**-0.23 [-0.28, -0.17]**
Heterogeneity: Tau^2 = 0.04; Chi^2 = 1043.99, df = 91 (P < 0.00001); I^2 = 91%								
Test for overall effect: Z = 8.54 (P < 0.00001)								
Test for subgroup differences: Chi^2 = 28.14, df = 5 (P < 0.0001), I^2 = 82.2%								

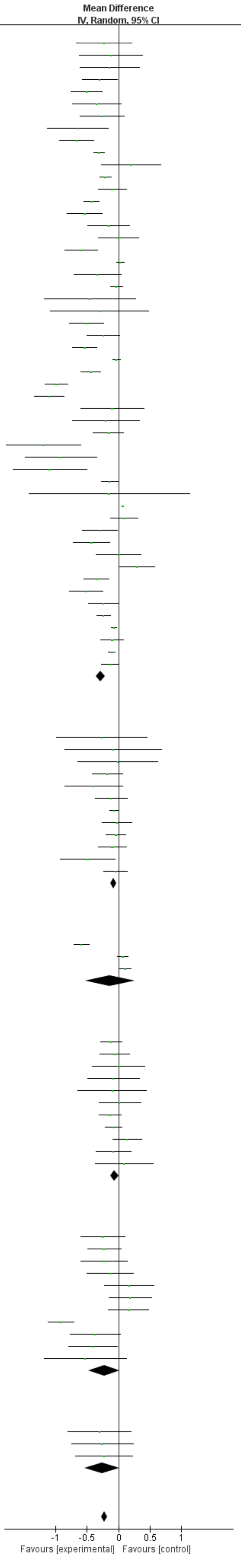

Figure S12.8. Forest plot of subgroup analyses by economic status

Study or Subgroup	Experimental Mean	Experimental SD	Experimental Total	Control Mean	Control SD	Control Total	Weight	Mean Difference IV, Random, 95% CI
**2.8.1 Lower-middle-income countries**								
Morgan 2021	1.46	0.12	15	2.05	0.2	15	1.6%	-0.59 [-0.71, -0.47]
Racil 2013(1)	1.3	0.05	11	1.23	0.1	6	1.6%	0.07 [-0.02, 0.16]
Racil 2013(2)	1.34	0.09	11	1.23	0.1	6	1.6%	0.11 [0.01, 0.21]
**Subtotal (95% CI)**			**37**			**27**	**4.8%**	**-0.13 [-0.54, 0.27]**
Heterogeneity: Tau^2 = 0.12; Chi^2 = 98.37, df = 2 (P < 0.00001); I^2 = 98%								
Test for overall effect: Z = 0.66 (P = 0.51)								
**2.8.2 Upper-middle-income countries**								
Ahmadi 2020(1)	1.33	0.5	28	1.56	0.62	9	0.8%	-0.23 [-0.68, 0.22]
Ahmadi 2020(2)	1.44	0.85	29	1.56	0.62	9	0.6%	-0.12 [-0.63, 0.39]
Ahmadi 2020(3)	1.42	0.7	29	1.56	0.62	9	0.7%	-0.14 [-0.62, 0.34]
Bezerra 2023	1.19	0.51	20	1.44	0.63	21	1.0%	-0.25 [-0.60, 0.10]
Boff 2020	1	0.45	34	1.23	0.63	31	1.2%	-0.23 [-0.50, 0.04]
Bowen 2010	0.89	0.44	22	1.19	0.46	18	1.1%	-0.30 [-0.58, -0.02]
Chuensiri 2018(1)	0.88	0.37	11	1.23	0.37	5	0.9%	-0.35 [-0.74, 0.04]
Chuensiri 2018(2)	0.97	0.39	15	1.23	0.37	6	0.9%	-0.26 [-0.62, 0.10]
Faria 2020(1)	0.89	0.54	24	1.12	0.56	13	0.9%	-0.23 [-0.60, 0.14]
Faria 2020(2)	0.99	0.56	26	1.12	0.56	13	0.9%	-0.13 [-0.50, 0.24]
Ghorbanian 2013	1.64	0.55	15	2.29	0.79	15	0.7%	-0.65 [-1.14, -0.16]
Guijun 2009	1.25	0.29	16	1.92	0.37	10	1.2%	-0.67 [-0.94, -0.40]
Gulijianati 2022	1.57	0.22	47	1.88	0.19	46	1.6%	-0.31 [-0.39, -0.23]
Guo 2015	1.51	0.86	23	1.31	0.56	12	0.7%	0.20 [-0.27, 0.67]
Hong 2016	1.07	0.22	55	1.28	0.27	55	1.6%	-0.21 [-0.30, -0.12]
Hongling 2012	0.9	0.5	30	1	0.4	30	1.3%	-0.10 [-0.33, 0.13]
Jian 2014	1.05	0.21	44	1.48	0.36	44	1.6%	-0.43 [-0.55, -0.31]
Jianjun 2012	0.81	0.34	20	1.35	0.47	15	1.1%	-0.54 [-0.82, -0.26]
Jun 2006(1)	1.14	0.55	33	1.3	0.78	31	1.0%	-0.16 [-0.49, 0.17]
Jun 2006(2)	1.08	0.68	35	1.08	0.65	32	1.0%	0.00 [-0.32, 0.32]
Ke 2024	0.9	0.3	10	1.5	0.3	10	1.2%	-0.60 [-0.86, -0.34]
Kelishadi 2008	1.46	0.14	45	1.43	0.14	47	1.7%	0.03 [-0.03, 0.09]
Liangmei 2015	0.76	0.38	13	1.27	0.31	12	1.2%	-0.51 [-0.78, -0.24]
Lidan 2012	1.08	0.65	30	1.32	0.33	30	1.2%	-0.24 [-0.50, 0.02]
Lingling 2021	1.71	0.31	20	2.25	0.32	20	1.4%	-0.54 [-0.74, -0.34]
Liqun 2014	0.99	0.19	76	1.02	0.2	97	1.7%	-0.03 [-0.09, 0.03]
Lira 2017(1)	1.41	0.75	26	1.24	0.54	16	0.9%	0.17 [-0.22, 0.56]
Lira 2017(2)	1.43	0.58	25	1.24	0.54	17	1.0%	0.19 [-0.15, 0.53]
Liu 2018	0.9	0.28	30	1.34	0.28	20	1.5%	-0.44 [-0.60, -0.28]
Lopes 2016	1.22	0.58	17	1.06	0.34	16	1.0%	0.16 [-0.16, 0.48]
Luo 2013(1)	0.53	0.41	43	1.52	0.41	34	1.4%	-0.99 [-1.17, -0.81]
Luo 2013(2)	0.49	0.73	52	1.6	0.42	38	1.3%	-1.11 [-1.35, -0.87]
Machado 2022	1.05	0.44	20	1.97	0.12	15	1.4%	-0.92 [-1.12, -0.72]
Meng 2022(1)	1.4	0.3	12	1.5	0.6	6	0.6%	-0.10 [-0.61, 0.41]
Meng 2022(2)	1.3	0.5	11	1.5	0.6	7	0.6%	-0.20 [-0.73, 0.33]
Mohammadi 2019	1.01	0.13	10	1.17	0.37	10	1.2%	-0.16 [-0.40, 0.08]
Monteiro 2015(1)	1.37	0.39	18	1.74	0.52	8	0.8%	-0.37 [-0.77, 0.03]
Monteiro 2015(2)	1.33	0.29	14	1.74	0.52	8	0.9%	-0.41 [-0.80, -0.02]
Plavsic 2020	0.9	0.32	22	1	0.43	22	1.3%	-0.10 [-0.32, 0.12]
Ramezani 2017(1)	1.9	0.57	15	3.1	0.59	5	0.5%	-1.20 [-1.79, -0.61]
Ramezani 2017(2)	2.18	0.47	15	3.1	0.59	5	0.6%	-0.92 [-1.49, -0.35]
Ramezani 2017(3)	2	0.56	15	3.1	0.59	5	0.5%	-1.10 [-1.69, -0.51]
Saygın 2011	1.59	0.24	20	1.73	0.2	19	1.5%	-0.14 [-0.28, -0.00]
Sun 2011	0.9	0.4	25	1.2	0.5	17	1.1%	-0.30 [-0.58, -0.02]
Vasconcellos 2016	1.11	0.22	10	1.64	1.03	10	0.5%	-0.53 [-1.18, 0.12]
Wen 2008	0.87	0.29	24	1.3	0.5	13	1.1%	-0.43 [-0.73, -0.13]
Woo 2004	1.5	0.7	41	1.2	0.6	41	1.1%	0.30 [0.02, 0.58]
Xinhui 2016	0.76	0.39	14	1.28	0.32	14	1.2%	-0.52 [-0.78, -0.26]
Xishao 2009	1.08	0.65	34	1.32	0.33	33	1.2%	-0.24 [-0.49, 0.01]
Xishao 2010	1.08	0.45	100	1.32	0.33	100	1.6%	-0.24 [-0.35, -0.13]
Yanqin 2017	1.24	0.09	35	1.31	0.1	33	1.7%	-0.07 [-0.12, -0.02]
Yu 2020	1.2	0.6	99	1.3	0.6	72	1.4%	-0.10 [-0.28, 0.08]
Zehsaz 2016	1.11	0.08	16	1.22	0.08	16	1.7%	-0.11 [-0.17, -0.05]
Zehsaz 2017	0.95	0.22	16	1.08	0.18	16	1.5%	-0.13 [-0.27, 0.01]
**Subtotal (95% CI)**			**1529**			**1256**	**60.3%**	**-0.30 [-0.37, -0.23]**
Heterogeneity: Tau^2 = 0.05; Chi^2 = 447.22, df = 53 (P < 0.00001); I^2 = 88%								
Test for overall effect: Z = 8.35 (P < 0.00001)								
**2.8.3 High-income countries**								
Boer 2014(1)	0.8	0.19	17	1.07	0.97	7	0.4%	-0.27 [-0.99, 0.45]
Boer 2014(2)	0.99	0.57	15	1.07	0.97	7	0.4%	-0.08 [-0.85, 0.69]
Chae 2010	0.97	0.39	19	1.48	0.39	19	1.2%	-0.51 [-0.76, -0.26]
Davis 2020	0.78	0.61	90	0.9	0.55	85	1.4%	-0.12 [-0.29, 0.05]
DeBar 2012	1.15	0.59	74	1.21	0.66	42	1.3%	-0.06 [-0.30, 0.18]
Dias 2018(1)	0.96	0.37	19	1.26	0.73	9	0.7%	-0.30 [-0.81, 0.21]
Dias 2018(2)	1	0.47	22	1.26	0.73	10	0.7%	-0.26 [-0.75, 0.23]
Elmahgoub 2009	1.25	0.65	15	1.26	1.08	15	0.5%	-0.01 [-0.65, 0.63]
Farpour-Lambert 2009	0.64	0.32	22	0.82	0.49	22	1.2%	-0.18 [-0.42, 0.06]
Harder-Lauridsen 2014	0.4	0.29	14	0.8	0.84	14	0.7%	-0.40 [-0.87, 0.07]
Horner 2015(1)	0.88	0.68	27	0.88	0.49	8	0.8%	0.00 [-0.43, 0.43]
Horner 2015(2)	0.8	0.57	23	0.88	0.49	8	0.8%	-0.08 [-0.49, 0.33]
Kelly 2004	1.22	0.54	10	1.32	0.7	10	0.6%	-0.10 [-0.65, 0.45]
Kim 2007	0.78	0.38	14	1.12	0.57	12	0.9%	-0.34 [-0.72, 0.04]
Kim 2022	0.62	0.15	13	0.65	0.09	13	1.6%	-0.03 [-0.13, 0.07]
Larsen 2016	0.89	0.4	47	1.01	0.7	36	1.2%	-0.12 [-0.38, 0.14]
Lee 2010(1)	1.28	0.64	20	1.74	1.03	9	0.4%	-0.46 [-1.19, 0.27]
Lee 2010(2)	1.44	0.83	16	1.74	1.03	9	0.3%	-0.30 [-1.09, 0.49]
Lima 2020	0.6	0.25	216	0.67	0.31	109	1.7%	-0.07 [-0.14, -0.00]
Meyer 2006	1.04	0.48	33	1.06	0.51	34	1.3%	-0.02 [-0.26, 0.22]
Migueles 2023	1.06	0.38	44	1.1	0.38	42	1.5%	-0.04 [-0.20, 0.12]
Murphy 2009	1.11	0.58	23	1.09	0.42	12	1.0%	0.02 [-0.32, 0.36]
Salus(2) 2022	1.2	0.37	14	1.69	0.75	14	0.8%	-0.49 [-0.93, -0.05]
Savoye 2007	1	0.37	75	1.13	0.53	44	1.4%	-0.13 [-0.31, 0.05]
Savoye 2011	1.01	0.26	75	1.09	0.41	44	1.5%	-0.08 [-0.21, 0.05]
Seo 2012	1.11	1.1	10	1.26	1.76	10	0.1%	-0.15 [-1.44, 1.14]
Seo 2019	1.17	0.02	26	1.1	0.02	44	1.7%	0.07 [0.06, 0.08]
Shalitin 2009	0.96	0.55	55	0.87	0.63	55	1.3%	0.09 [-0.13, 0.31]
Staiano 2017	1.04	0.33	20	0.9	0.39	18	1.3%	0.14 [-0.09, 0.37]
Staiano 2018	0.86	0.48	22	0.94	0.47	23	1.1%	-0.08 [-0.36, 0.20]
Tas 2023	1.18	0.53	31	1.09	0.53	6	0.7%	0.09 [-0.37, 0.55]
Tjonna 2009	1.19	0.31	20	1.24	0.33	22	1.4%	-0.05 [-0.24, 0.14]
Tsang 2009	1.12	0.35	11	1.35	0.59	8	0.7%	-0.23 [-0.69, 0.23]
Wong 2008	1	0.5	12	1	0.4	12	0.9%	0.00 [-0.36, 0.36]
Woo 2012	1.5	0.18	10	1.85	0.27	10	1.4%	-0.35 [-0.55, -0.15]
**Subtotal (95% CI)**			**1174**			**842**	**34.9%**	**-0.10 [-0.16, -0.04]**
Heterogeneity: Tau^2 = 0.01; Chi^2 = 106.25, df = 34 (P < 0.00001); I^2 = 68%								
Test for overall effect: Z = 3.16 (P = 0.002)								
**Total (95% CI)**			**2740**			**2125**	**100.0%**	**-0.23 [-0.28, -0.17]**
Heterogeneity: Tau^2 = 0.04; Chi^2 = 1043.99, df = 91 (P < 0.00001); I^2 = 91%								
Test for overall effect: Z = 8.54 (P < 0.00001)								
Test for subgroup differences: Chi^2 = 17.53, df = 2 (P = 0.0002), I^2 = 88.6%								

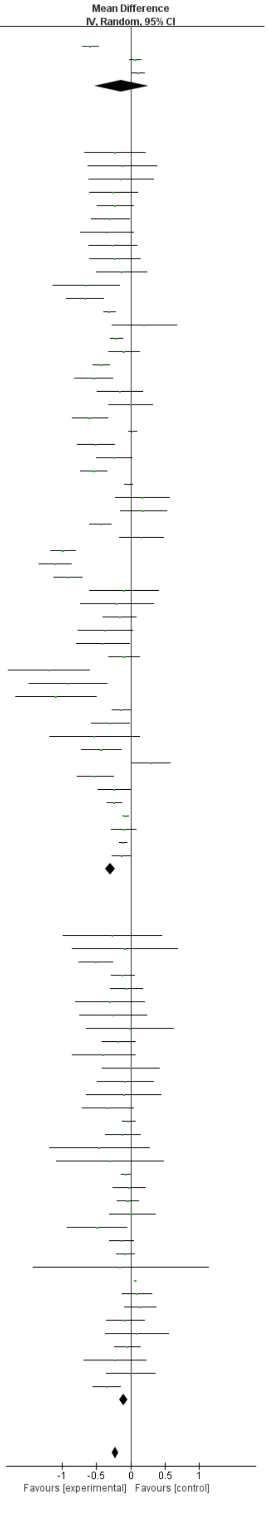

Figure S12.9. Forest plot of subgroup analyses by weight status

Study or Subgroup	Experimental Mean	Experimental SD	Experimental Total	Control Mean	Control SD	Control Total	Weight	Mean Difference IV, Random, 95% CI
**2.9.1 Overweight**								
Bezerra 2023	1.19	0.51	20	1.44	0.63	21	1.3%	-0.25 [-0.60, 0.10]
Boer 2014(1)	0.8	0.19	17	1.07	0.97	7	0.6%	-0.27 [-0.99, 0.45]
Boer 2014(2)	0.99	0.57	15	1.07	0.97	7	0.5%	-0.08 [-0.85, 0.69]
DeBar 2012	1.15	0.59	74	1.21	0.66	42	1.7%	-0.06 [-0.30, 0.18]
Faria 2020(1)	0.89	0.54	24	1.12	0.56	13	1.2%	-0.23 [-0.60, 0.14]
Faria 2020(2)	0.99	0.56	26	1.12	0.56	13	1.2%	-0.13 [-0.50, 0.24]
Gulijianati 2022	1.57	0.22	47	1.88	0.19	46	2.1%	-0.31 [-0.39, -0.23]
Harder-Lauridsen 2014	0.4	0.29	14	0.8	0.84	14	1.0%	-0.40 [-0.87, 0.07]
Kelly 2004	1.22	0.54	10	1.32	0.7	10	0.8%	-0.10 [-0.65, 0.45]
Lopes 2016	1.22	0.58	17	1.06	0.34	16	1.4%	0.16 [-0.16, 0.48]
Mohammadi 2019	1.01	0.13	10	1.17	0.37	10	1.7%	-0.16 [-0.40, 0.08]
Murphy 2009	1.11	0.58	23	1.09	0.42	12	1.4%	0.02 [-0.32, 0.36]
Sun 2011	0.9	0.4	25	1.2	0.5	17	1.5%	-0.30 [-0.58, -0.02]
Woo 2012	1.5	0.18	10	1.85	0.27	10	1.8%	-0.35 [-0.55, -0.15]
**Subtotal (95% CI)**			**332**			**238**	**18.2%**	**-0.21 [-0.30, -0.13]**
Heterogeneity: Tau² = 0.00; Chi² = 15.96, df = 13 (P = 0.25); I² = 19%								
Test for overall effect: Z = 5.03 (P < 0.00001)								
**2.9.2 Obesity**								
Chae 2010	0.97	0.39	19	1.48	0.39	19	1.6%	-0.51 [-0.76, -0.26]
Chuensiri 2018(1)	0.88	0.37	11	1.23	0.37	5	1.2%	-0.35 [-0.74, 0.04]
Chuensiri 2018(2)	0.97	0.39	15	1.23	0.37	6	1.3%	-0.26 [-0.62, 0.10]
Dias 2018(1)	0.96	0.37	19	1.26	0.73	9	0.9%	-0.30 [-0.81, 0.21]
Dias 2018(2)	1	0.47	22	1.26	0.73	10	0.9%	-0.26 [-0.75, 0.23]
Farpour-Lambert 2009	0.64	0.32	22	0.82	0.49	22	1.7%	-0.18 [-0.42, 0.06]
Guijun 2009	1.25	0.29	16	1.92	0.37	10	1.6%	-0.67 [-0.94, -0.40]
Hong 2016	1.07	0.22	55	1.28	0.27	55	2.1%	-0.21 [-0.30, -0.12]
Hongling 2012	0.9	0.5	30	1	0.4	30	1.7%	-0.10 [-0.33, 0.13]
Horner 2015(1)	0.88	0.68	27	0.88	0.49	8	1.1%	0.00 [-0.43, 0.43]
Horner 2015(2)	0.8	0.57	23	0.88	0.49	8	1.1%	-0.08 [-0.49, 0.33]
Jian 2014	1.05	0.21	44	1.48	0.36	44	2.0%	-0.43 [-0.55, -0.31]
Jianjun 2012	0.81	0.34	20	1.35	0.47	15	1.5%	-0.54 [-0.82, -0.26]
Jun 2006(1)	1.14	0.55	33	1.3	0.78	31	1.4%	-0.16 [-0.49, 0.17]
Jun 2006(2)	1.08	0.68	35	1.08	0.65	32	1.4%	0.00 [-0.32, 0.32]
Ke 2024	0.9	0.3	10	1.5	0.3	10	1.6%	-0.60 [-0.86, -0.34]
Kelishadi 2008	1.46	0.14	45	1.43	0.14	47	2.2%	0.03 [-0.03, 0.09]
Kim 2007	0.78	0.38	14	1.12	0.57	12	1.2%	-0.34 [-0.72, 0.04]
Lee 2010(1)	1.28	0.64	20	1.74	1.03	9	0.6%	-0.46 [-1.19, 0.27]
Lee 2010(2)	1.44	0.83	16	1.74	1.03	9	0.5%	-0.30 [-1.09, 0.49]
Liangmei 2015	0.76	0.38	13	1.27	0.31	12	1.6%	-0.51 [-0.78, -0.24]
Lidan 2012	1.08	0.65	30	1.32	0.33	30	1.6%	-0.24 [-0.50, 0.02]
Lira 2017(1)	1.41	0.75	26	1.24	0.54	16	1.2%	0.17 [-0.22, 0.56]
Lira 2017(2)	1.43	0.58	25	1.24	0.54	17	1.3%	0.19 [-0.15, 0.53]
Liu 2018	0.9	0.28	30	1.34	0.28	20	1.9%	-0.44 [-0.60, -0.28]
Luo 2013(1)	0.53	0.41	43	1.52	0.41	34	1.9%	-0.99 [-1.17, -0.81]
Luo 2013(2)	0.49	0.73	52	1.6	0.42	38	1.7%	-1.11 [-1.35, -0.87]
Meng 2022(1)	1.4	0.3	12	1.5	0.6	6	0.9%	-0.10 [-0.61, 0.41]
Meng 2022(2)	1.3	0.5	11	1.5	0.6	7	0.8%	-0.20 [-0.73, 0.33]
Meyer 2006	1.04	0.48	33	1.06	0.51	34	1.7%	-0.02 [-0.26, 0.22]
Monteiro 2015(1)	1.37	0.39	18	1.74	0.52	8	1.2%	-0.37 [-0.77, 0.03]
Monteiro 2015(2)	1.33	0.29	14	1.74	0.52	8	1.2%	-0.41 [-0.80, -0.02]
Morgan 2021	1.46	0.12	15	2.05	0.2	15	2.1%	-0.59 [-0.71, -0.47]
Plavsic 2020	0.9	0.32	22	1	0.43	22	1.7%	-0.10 [-0.32, 0.12]
Racil 2013(1)	1.3	0.05	11	1.23	0.1	6	2.1%	0.07 [-0.02, 0.16]
Racil 2013(2)	1.34	0.09	11	1.23	0.1	6	2.1%	0.11 [0.01, 0.21]
Ramezani 2017(1)	1.9	0.57	15	3.1	0.59	5	0.7%	-1.20 [-1.79, -0.61]
Ramezani 2017(2)	2.18	0.47	15	3.1	0.59	5	0.8%	-0.92 [-1.49, -0.35]
Ramezani 2017(3)	2	0.56	15	3.1	0.59	5	0.7%	-1.10 [-1.69, -0.51]
Salus(2) 2022	1.2	0.37	14	1.69	0.75	14	1.1%	-0.49 [-0.93, -0.05]
Savoye 2007	1	0.37	75	1.13	0.53	44	1.9%	-0.13 [-0.31, 0.05]
Savoye 2011	1.01	0.26	75	1.09	0.41	44	2.0%	-0.08 [-0.21, 0.05]
Saygın 2011	1.59	0.24	20	1.73	0.2	19	2.0%	-0.14 [-0.28, -0.00]
Seo 2012	1.11	1.1	10	1.26	1.76	10	0.2%	-0.15 [-1.44, 1.14]
Seo 2019	1.17	0.02	26	1.1	0.02	44	2.2%	0.07 [0.06, 0.08]
Shalitin 2009	0.96	0.55	55	0.87	0.63	55	1.7%	0.09 [-0.13, 0.31]
Tas 2023	1.18	0.53	31	1.09	0.53	6	1.0%	0.09 [-0.37, 0.55]
Vasconcellos 2016	1.11	0.22	10	1.64	1.03	10	0.6%	-0.53 [-1.18, 0.12]
Wen 2008	0.87	0.29	24	1.3	0.5	13	1.5%	-0.43 [-0.73, -0.13]
Wong 2008	1	0.5	12	1	0.4	12	1.3%	0.00 [-0.36, 0.36]
Xinhui 2016	0.76	0.39	14	1.28	0.32	14	1.6%	-0.52 [-0.78, -0.26]
Xishao 2009	1.08	0.65	34	1.32	0.33	33	1.6%	-0.24 [-0.49, 0.01]
Xishao 2010	1.08	0.45	100	1.32	0.33	100	2.1%	-0.24 [-0.35, -0.13]
Yanqin 2017	1.24	0.09	35	1.31	0.1	33	2.2%	-0.07 [-0.12, -0.02]
Yu 2020	1.2	0.6	99	1.3	0.6	72	1.9%	-0.10 [-0.28, 0.08]
Zehsaz 2016	1.11	0.08	16	1.22	0.08	16	2.2%	-0.11 [-0.17, -0.05]
**Subtotal (95% CI)**			**1557**			**1224**	**81.8%**	**-0.27 [-0.34, -0.20]**
Heterogeneity: Tau² = 0.05; Chi² = 788.36, df = 55 (P < 0.00001); I² = 93%								
Test for overall effect: Z = 7.56 (P < 0.00001)								
**Total (95% CI)**			**1889**			**1462**	**100.0%**	**-0.25 [-0.31, -0.19]**
Heterogeneity: Tau² = 0.05; Chi² = 883.10, df = 69 (P < 0.00001); I² = 92%								
Test for overall effect: Z = 7.87 (P < 0.00001)								
Test for subgroup differences: Chi² = 0.94, df = 1 (P = 0.33), I² = 0%								

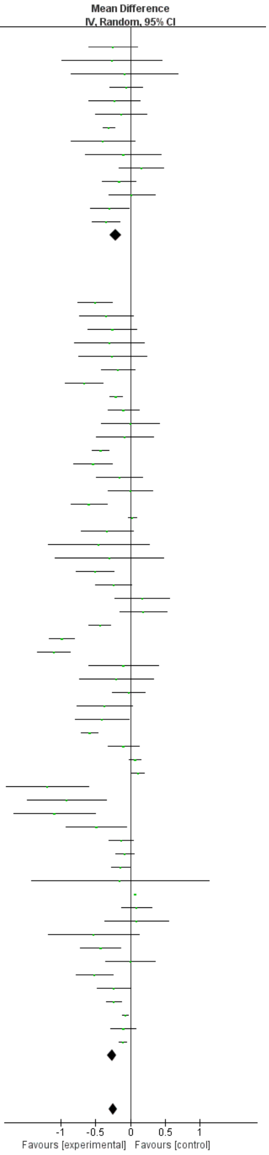

Figure S12.10. Forest plot of subgroup analyses by sex

Study or Subgroup	Experimental Mean	Experimental SD	Experimental Total	Control Mean	Control SD	Control Total	Weight	Mean Difference IV, Random, 95% CI
**2.10.1 Boys**								
Chuensiri 2018(1)	0.88	0.37	11	1.23	0.37	5	2.9%	-0.35 [-0.74, 0.04]
Chuensiri 2018(2)	0.97	0.39	15	1.23	0.37	6	3.1%	-0.26 [-0.62, 0.10]
Ghorbanian 2013	1.64	0.55	15	2.29	0.79	15	2.4%	-0.65 [-1.14, -0.16]
Hongling 2012	0.9	0.5	30	1	0.4	30	3.8%	-0.10 [-0.33, 0.13]
Jianjun 2012	0.81	0.34	20	1.35	0.47	15	3.5%	-0.54 [-0.82, -0.26]
Kim 2007	0.78	0.38	14	1.12	0.57	12	3.0%	-0.34 [-0.72, 0.04]
Lingling 2021	1.71	0.31	20	2.25	0.32	20	4.0%	-0.54 [-0.74, -0.34]
Luo 2013(2)	0.49	0.73	52	1.6	0.42	38	3.7%	-1.11 [-1.35, -0.87]
Meng 2022(1)	1.4	0.3	12	1.5	0.6	6	2.3%	-0.10 [-0.61, 0.41]
Meng 2022(2)	1.3	0.5	11	1.5	0.6	7	2.2%	-0.20 [-0.73, 0.33]
Ramezani 2017(1)	1.9	0.57	15	3.1	0.59	5	2.0%	-1.20 [-1.79, -0.61]
Ramezani 2017(2)	2.18	0.47	15	3.1	0.59	5	2.1%	-0.92 [-1.49, -0.35]
Ramezani 2017(3)	2	0.56	15	3.1	0.59	5	2.0%	-1.10 [-1.69, -0.51]
Salus(2) 2022	1.2	0.37	14	1.69	0.75	14	2.7%	-0.49 [-0.93, -0.05]
Seo 2012	1.11	1.1	10	1.26	1.76	10	0.7%	-0.15 [-1.44, 1.14]
Wong 2008	1	0.5	12	1	0.4	12	3.1%	0.00 [-0.36, 0.36]
Zehsaz 2016	1.11	0.08	16	1.22	0.08	16	4.4%	-0.11 [-0.17, -0.05]
Zehsaz 2017	0.95	0.22	16	1.08	0.18	16	4.2%	-0.13 [-0.27, 0.01]
**Subtotal (95% CI)**			**313**			**237**	**52.1%**	**-0.44 [-0.61, -0.27]**
Heterogeneity: Tau² = 0.09; Chi² = 118.00, df = 17 (P < 0.00001); I² = 86%								
Test for overall effect: Z = 5.11 (P < 0.00001)								
**2.10.2 Girls**								
DeBar 2012	1.15	0.59	74	1.21	0.66	42	3.7%	-0.06 [-0.30, 0.18]
Jian 2014	1.05	0.21	44	1.48	0.36	44	4.3%	-0.43 [-0.55, -0.31]
Kim 2022	0.62	0.15	13	0.65	0.09	13	4.3%	-0.03 [-0.13, 0.07]
Liu 2018	0.9	0.28	30	1.34	0.28	20	4.1%	-0.44 [-0.60, -0.28]
Lopes 2016	1.22	0.58	17	1.06	0.34	16	3.3%	0.16 [-0.16, 0.48]
Luo 2013(1)	0.53	0.41	43	1.52	0.41	34	4.0%	-0.99 [-1.17, -0.81]
Mohammadi 2019	1.01	0.13	10	1.17	0.37	10	3.7%	-0.16 [-0.40, 0.08]
Plavsic 2020	0.9	0.32	22	1	0.43	22	3.8%	-0.10 [-0.32, 0.12]
Racil 2013(1)	1.3	0.05	11	1.23	0.1	6	4.4%	0.07 [-0.02, 0.16]
Racil 2013(2)	1.34	0.09	11	1.23	0.1	6	4.3%	0.11 [0.01, 0.21]
Saygın 2011	1.59	0.24	20	1.73	0.2	19	4.2%	-0.14 [-0.28, -0.00]
Staiano 2017	1.04	0.33	20	0.9	0.39	18	3.8%	0.14 [-0.09, 0.37]
**Subtotal (95% CI)**			**315**			**250**	**47.9%**	**-0.16 [-0.33, 0.01]**
Heterogeneity: Tau² = 0.08; Chi² = 177.69, df = 11 (P < 0.00001); I² = 94%								
Test for overall effect: Z = 1.85 (P = 0.06)								
**Total (95% CI)**			**628**			**487**	**100.0%**	**-0.30 [-0.42, -0.19]**
Heterogeneity: Tau² = 0.07; Chi² = 307.97, df = 29 (P < 0.00001); I² = 91%								
Test for overall effect: Z = 5.28 (P < 0.00001)								
Test for subgroup differences: Chi² = 5.24, df = 1 (P = 0.02), I² = 80.9%								

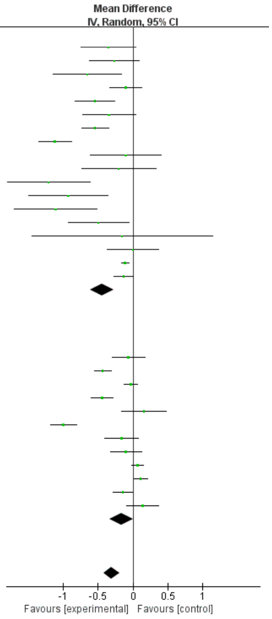

Figure S13. Forest plots of subgroup analyses for HDL-C in children and adolescents with overweight or obesity

Figure S13.1. Forest plot of subgroup analyses by type of exercise

Study or Subgroup	Experimental Mean	Experimental SD	Experimental Total	Control Mean	Control SD	Control Total	Weight	Mean Difference IV, Random, 95% CI
**2.1.1 Aerobic exercise**								
Ahmadi 2020(1)	1.13	0.24	28	0.97	0.24	9	1.0%	0.16 [-0.02, 0.34]
Bezerra 2023	1.19	0.31	20	1.12	0.12	21	1.2%	0.07 [-0.08, 0.22]
Boer 2014(2)	1.29	0.27	15	1.45	0.4	7	0.5%	-0.16 [-0.49, 0.17]
Boff 2020	1.3	0.36	34	1.37	0.53	31	0.8%	-0.07 [-0.29, 0.15]
Bowen 2010	1.13	0.19	22	1.11	0.2	18	1.4%	0.02 [-0.10, 0.14]
Davis 2020	1.33	0.42	90	0.9	0.44	85	1.3%	0.43 [0.30, 0.56]
DeBar 2012	1.12	0.22	74	1.15	0.22	42	1.6%	-0.03 [-0.11, 0.05]
Dias 2018(2)	1.25	0.26	22	1.3	0.31	10	0.8%	-0.05 [-0.27, 0.17]
Ghorbanian 2013	1.17	0.19	15	1.15	0.16	15	1.3%	0.02 [-0.11, 0.15]
Guijun 2009	1.22	0.16	16	0.91	0.1	10	1.5%	0.31 [0.21, 0.41]
Gulijianati 2022	1.66	0.28	47	1.38	0.27	46	1.4%	0.28 [0.17, 0.39]
Guo 2015	1.34	0.27	23	1.11	0.27	12	0.9%	0.23 [0.04, 0.42]
Horner 2015(1)	1.19	0.31	27	1.03	0.19	8	1.0%	0.16 [-0.02, 0.34]
Jian 2014	1.59	0.45	44	1.44	0.55	44	0.8%	0.15 [-0.06, 0.36]
Jianjun 2012	1.3	0.3	20	1.18	0.18	15	1.1%	0.12 [-0.04, 0.28]
Jun 2006(1)	1.61	0.27	33	1.45	0.32	31	1.2%	0.16 [0.01, 0.31]
Jun 2006(2)	1.61	0.26	35	1.53	0.44	32	1.0%	0.08 [-0.10, 0.26]
Karacabey 2009	1.53	0.19	20	1.17	0.09	20	1.6%	0.36 [0.27, 0.45]
Kelishadi 2008	1.01	0.06	45	1	0.06	47	1.9%	0.01 [-0.01, 0.03]
Kelly 2004	1.1	0.13	10	0.99	0.28	10	0.9%	0.11 [-0.08, 0.30]
Kim 2007	1.12	0.14	14	1.15	0.13	12	1.5%	-0.03 [-0.13, 0.07]
Lee 2010(2)	1.27	0.26	16	1.15	0.19	9	1.0%	0.12 [-0.06, 0.30]
Liqun 2014	1.6	0.29	76	1.57	0.21	97	1.7%	0.03 [-0.05, 0.11]
Lira 2017(1)	1.18	0.22	26	1.12	0.26	16	1.2%	0.06 [-0.09, 0.21]
Lira 2017(2)	1.17	0.27	25	1.12	0.26	17	1.1%	0.05 [-0.11, 0.21]
Liu 2018	1.21	0.18	30	1.1	0.4	20	1.0%	0.11 [-0.08, 0.30]
Luo 2013(1)	1.15	0.21	43	1.17	0.17	34	1.6%	-0.02 [-0.10, 0.06]
Luo 2013(2)	1.09	0.18	52	1.2	0.16	38	1.7%	-0.11 [-0.18, -0.04]
Machado 2022	1.22	0.22	20	0.91	0.18	15	1.3%	0.31 [0.18, 0.44]
Meng 2022(2)	1.4	0.2	11	1.3	0.2	7	0.9%	0.10 [-0.09, 0.29]
Meyer 2006	1.09	0.24	33	1.11	0.31	34	1.3%	-0.02 [-0.15, 0.11]
Monteiro 2015(1)	0.98	0.26	18	0.94	0.31	8	0.7%	0.04 [-0.21, 0.29]
Morgan 2021	1.3	0.08	15	1.12	0.11	15	1.7%	0.18 [0.11, 0.25]
Murphy 2009	0.93	0.21	23	0.92	0.17	12	1.3%	0.01 [-0.12, 0.14]
Park 2007	1.26	0.16	19	1.26	0.2	21	1.4%	0.00 [-0.11, 0.11]
Plavsic 2020	1.33	0.27	22	1.3	0.26	22	1.1%	0.03 [-0.13, 0.19]
Racil 2013(2)	1.09	0.07	11	1	0.03	6	1.8%	0.09 [0.04, 0.14]
Ramezani 2017(1)	1.26	0.11	15	1.07	0.14	5	1.3%	0.19 [0.06, 0.32]
Savoye 2007	1.17	0.15	75	1.15	0.45	44	1.3%	0.02 [-0.12, 0.16]
Savoye 2011	1.19	0.18	75	1.12	0.41	44	1.3%	0.07 [-0.06, 0.20]
Saygın 2011	1.36	0.21	20	1.08	0.12	19	1.5%	0.28 [0.17, 0.39]
Seo 2012	0.72	1.15	10	0.95	1.15	10	0.1%	-0.23 [-1.24, 0.78]
Seo 2019	1.25	0.24	26	1.22	0.26	44	1.4%	0.03 [-0.09, 0.15]
Staiano 2017	1.26	0.18	20	1.33	0.22	18	1.3%	-0.07 [-0.20, 0.06]
Staiano 2018	1.22	0.19	22	1.31	0.19	23	1.4%	-0.09 [-0.20, 0.02]
Sun 2011	1.1	0.2	25	1.1	0.2	17	1.3%	0.00 [-0.12, 0.12]
Tjonna 2009	1.24	0.18	20	1.14	0.19	22	1.4%	0.10 [-0.01, 0.21]
Tsang 2009	0.96	0.21	11	0.91	0.23	8	0.9%	0.05 [-0.15, 0.25]
Woo 2004	1.2	0.3	41	1.2	0.2	41	1.4%	0.00 [-0.11, 0.11]
Xinhui 2016	1.21	0.19	14	1.23	0.19	14	1.2%	-0.02 [-0.16, 0.12]
Yu 2020	1.5	0.3	99	1.3	0.2	72	1.7%	0.20 [0.12, 0.28]
**Subtotal (95% CI)**			**1567**			**1277**	**63.2%**	**0.08 [0.05, 0.12]**
Heterogeneity: Tau² = 0.01; Chi² = 255.01, df = 50 (P < 0.00001); I² = 80%								
Test for overall effect: Z = 4.68 (P < 0.00001)								
**2.1.2 Anaerobic exercise**								
Boer 2014(1)	1.54	0.29	17	1.45	0.4	7	0.5%	0.09 [-0.24, 0.42]
Chuensiri 2018(1)	1.19	0.26	11	1.34	0.26	5	0.6%	-0.15 [-0.42, 0.12]
Chuensiri 2018(2)	1.11	0.3	15	1.34	0.26	6	0.7%	-0.23 [-0.49, 0.03]
Dias 2018(1)	1.24	0.27	19	1.3	0.31	9	0.7%	-0.06 [-0.30, 0.18]
Ke 2024	1.4	0.2	10	1.3	0.3	10	0.8%	0.10 [-0.12, 0.32]
Lingling 2021	1.07	0.16	20	0.97	0.22	20	1.4%	0.10 [-0.02, 0.22]
Meng 2022(1)	1.5	0.2	12	1.3	0.2	6	0.9%	0.20 [0.00, 0.40]
Racil 2013(1)	1.08	0.08	11	1	0.03	6	1.8%	0.08 [0.03, 0.13]
Salus(2) 2022	1.07	0.19	14	0.96	0.11	14	1.4%	0.11 [-0.01, 0.23]
Tas 2023	1.04	0.23	31	1.14	0.23	6	0.9%	-0.10 [-0.30, 0.10]
Vasconcellos 2016	1.15	0.13	10	1	0.44	10	0.6%	0.15 [-0.13, 0.43]
**Subtotal (95% CI)**			**170**			**99**	**10.2%**	**0.06 [-0.01, 0.12]**
Heterogeneity: Tau² = 0.00; Chi² = 14.51, df = 10 (P = 0.15); I² = 31%								
Test for overall effect: Z = 1.79 (P = 0.07)								
**2.1.3 Resistance exercise**								
Ahmadi 2020(2)	1.12	0.23	29	0.97	0.24	9	1.0%	0.15 [-0.03, 0.33]
Benson 2008	1.5	0.4	32	1.4	0.2	35	1.1%	0.10 [-0.05, 0.25]
Horner 2015(2)	1.02	0.23	23	1.03	0.19	8	1.1%	-0.01 [-0.17, 0.15]
Ramezani 2017(2)	1.21	0.14	15	1.07	0.14	5	1.2%	0.14 [-0.00, 0.28]
**Subtotal (95% CI)**			**99**			**57**	**4.5%**	**0.10 [0.02, 0.17]**
Heterogeneity: Tau² = 0.00; Chi² = 2.38, df = 3 (P = 0.50); I² = 0%								
Test for overall effect: Z = 2.40 (P = 0.02)								
**2.1.4 Combined aerobic and resistance exercise**								
Ahmadi 2020(3)	1.1	0.22	29	0.97	0.24	9	1.0%	0.13 [-0.05, 0.31]
Bruyndonckx 2015	1.25	0.26	27	1.08	0.27	21	1.2%	0.17 [0.02, 0.32]
Chae 2010	1.37	0.25	19	1.31	0.28	19	1.1%	0.06 [-0.11, 0.23]
Elmahgoub 2009	1.46	0.29	15	1.42	0.46	15	0.6%	0.04 [-0.24, 0.32]
Faria 2020(1)	1.34	0.26	24	1.3	0.27	13	1.0%	0.04 [-0.14, 0.22]
Faria 2020(2)	1.32	0.27	26	1.3	0.27	13	1.0%	0.02 [-0.16, 0.20]
Farpour-Lambert 2009	1.14	0.19	22	0.97	0.31	22	1.2%	0.17 [0.02, 0.32]
Harder-Lauridsen 2014	1.4	0.4	14	1.6	0.43	14	0.5%	-0.20 [-0.51, 0.11]
Hongling 2012	1.2	0.2	30	1.2	0.3	30	1.3%	0.00 [-0.13, 0.13]
Kim 2022	2.26	1.08	13	1.82	1	13	0.1%	0.44 [-0.36, 1.24]
Lee 2010(1)	1.28	0.24	20	1.15	0.19	9	1.1%	0.13 [-0.03, 0.29]
Liangmei 2015	1.2	0.18	12	1.22	0.18	12	1.2%	-0.02 [-0.16, 0.12]
Lopes 2016	1.37	0.32	17	1.55	0.47	16	0.6%	-0.18 [-0.46, 0.10]
Migueles 2023	1.33	0.21	42	1.27	0.21	37	1.6%	0.06 [-0.03, 0.15]
Mohammadi 2019	1.1	0.09	10	1.11	0.04	10	1.8%	-0.01 [-0.07, 0.05]
Monteiro 2015(2)	1.1	0.32	14	0.94	0.31	8	0.6%	0.16 [-0.11, 0.43]
Ramezani 2017(3)	1.19	0.14	15	1.07	0.14	5	1.2%	0.12 [-0.02, 0.26]
Shalitin 2009	1.29	0.27	55	1.24	0.3	55	1.5%	0.05 [-0.06, 0.16]
Wen 2008	1.31	0.18	24	0.95	0.27	13	1.1%	0.36 [0.20, 0.52]
Wong 2008	1.3	0.3	12	1.2	0.2	12	0.9%	0.10 [-0.10, 0.30]
Zehsaz 2016	1.17	0.31	16	1.16	0.26	16	0.9%	0.01 [-0.19, 0.21]
Zehsaz 2017	1.17	0.31	16	1.16	0.26	16	0.9%	0.01 [-0.19, 0.21]
**Subtotal (95% CI)**			**472**			**378**	**22.2%**	**0.07 [0.02, 0.11]**
Heterogeneity: Tau² = 0.00; Chi² = 33.87, df = 21 (P = 0.04); I² = 38%								
Test for overall effect: Z = 3.02 (P = 0.003)								
**Total (95% CI)**			**2308**			**1811**	**100.0%**	**0.08 [0.05, 0.10]**
Heterogeneity: Tau² = 0.01; Chi² = 306.87, df = 87 (P < 0.00001); I² = 72%								
Test for overall effect: Z = 5.95 (P < 0.00001)								
Test for subgroup differences: Chi² = 1.06, df = 3 (P = 0.79), I² = 0%								

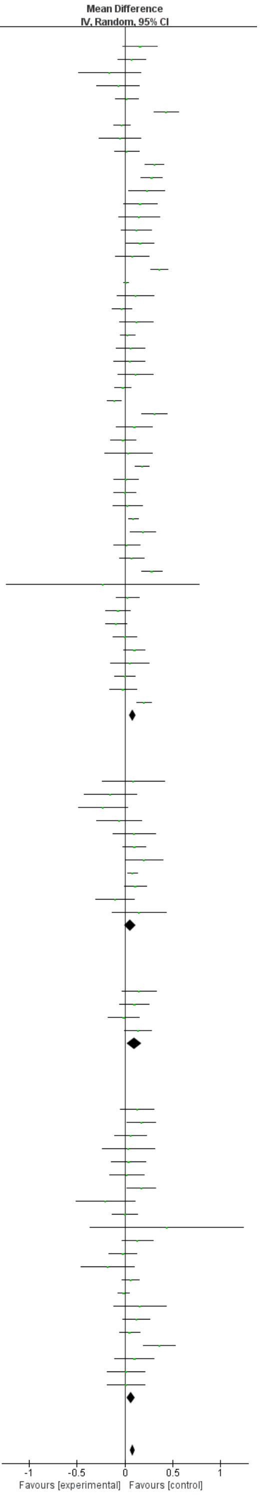

Figure S13.2. Forest plot of subgroup analyses by exercise intensity

Study or Subgroup	Experimental Mean	Experimental SD	Experimental Total	Control Mean	Control SD	Control Total	Weight	Mean Difference IV, Random, 95% CI
**2.2.1 Moderate-to-low intensity**								
Boer 2014(2)	1.29	0.27	15	1.45	0.4	7	0.5%	-0.16 [-0.49, 0.17]
Boff 2020	1.3	0.36	34	1.37	0.53	31	0.8%	-0.07 [-0.29, 0.15]
Bowen 2010	1.13	0.19	22	1.11	0.2	18	1.4%	0.02 [-0.10, 0.14]
Bruyndonckx 2015	1.25	0.26	27	1.08	0.27	21	1.2%	0.17 [0.02, 0.32]
Davis 2020	1.33	0.42	90	0.9	0.44	85	1.3%	0.43 [0.30, 0.56]
DeBar 2012	1.12	0.22	74	1.15	0.22	42	1.6%	-0.03 [-0.11, 0.05]
Dias 2018(2)	1.25	0.26	22	1.3	0.31	10	0.8%	-0.05 [-0.27, 0.17]
Elmahgoub 2009	1.46	0.29	15	1.42	0.46	15	0.6%	0.04 [-0.24, 0.32]
Faria 2020(1)	1.34	0.26	24	1.3	0.27	13	1.0%	0.04 [-0.14, 0.22]
Farpour-Lambert 2009	1.14	0.19	22	0.97	0.31	22	1.2%	0.17 [0.02, 0.32]
Ghorbanian 2013	1.17	0.19	15	1.15	0.16	15	1.3%	0.02 [-0.11, 0.15]
Guijun 2009	1.22	0.16	16	0.91	0.1	10	1.5%	0.31 [0.21, 0.41]
Guo 2015	1.34	0.27	23	1.11	0.27	12	0.9%	0.23 [0.04, 0.42]
Harder-Lauridsen 2014	1.4	0.4	14	1.6	0.43	14	0.5%	-0.20 [-0.51, 0.11]
Horner 2015(1)	1.19	0.31	27	1.03	0.19	8	1.0%	0.16 [-0.02, 0.34]
Jian 2014	1.59	0.45	44	1.44	0.55	44	0.8%	0.15 [-0.06, 0.36]
Jianjun 2012	1.3	0.3	20	1.18	0.18	15	1.1%	0.12 [-0.04, 0.28]
Jun 2006(1)	1.61	0.27	33	1.45	0.32	31	1.2%	0.16 [0.01, 0.31]
Jun 2006(2)	1.61	0.26	35	1.53	0.44	32	1.0%	0.08 [-0.10, 0.26]
Karacabey 2009	1.53	0.19	20	1.17	0.09	20	1.6%	0.36 [0.27, 0.45]
Kelishadi 2008	1.01	0.06	45	1	0.06	47	1.9%	0.01 [-0.01, 0.03]
Kelly 2004	1.1	0.13	10	0.99	0.28	10	0.9%	0.11 [-0.08, 0.30]
Kim 2007	1.12	0.14	14	1.15	0.13	12	1.5%	-0.03 [-0.13, 0.07]
Kim 2022	2.26	1.08	13	1.82	1	13	0.1%	0.44 [-0.36, 1.24]
Lee 2010(1)	1.28	0.24	20	1.15	0.19	9	1.1%	0.13 [-0.03, 0.29]
Lee 2010(2)	1.27	0.26	16	1.15	0.19	9	1.0%	0.12 [-0.06, 0.30]
Liangmei 2015	1.2	0.18	12	1.22	0.18	12	1.2%	-0.02 [-0.16, 0.12]
Liqun 2014	1.6	0.29	76	1.57	0.21	97	1.7%	0.03 [-0.05, 0.11]
Lira 2017(2)	1.17	0.27	25	1.12	0.26	17	1.1%	0.05 [-0.11, 0.21]
Liu 2018	1.21	0.18	30	1.1	0.4	20	1.0%	0.11 [-0.08, 0.30]
Luo 2013(1)	1.15	0.21	43	1.17	0.17	34	1.6%	-0.02 [-0.10, 0.06]
Luo 2013(2)	1.09	0.18	52	1.2	0.16	38	1.7%	-0.11 [-0.18, -0.04]
Machado 2022	1.22	0.22	20	0.91	0.18	15	1.3%	0.31 [0.18, 0.44]
Meng 2022(2)	1.4	0.2	11	1.3	0.2	7	0.9%	0.10 [-0.09, 0.29]
Meyer 2006	1.09	0.24	33	1.11	0.31	34	1.3%	-0.02 [-0.15, 0.11]
Mohammadi 2019	1.1	0.09	10	1.11	0.04	10	1.8%	-0.01 [-0.07, 0.05]
Morgan 2021	1.3	0.08	15	1.12	0.11	15	1.7%	0.18 [0.11, 0.25]
Murphy 2009	0.93	0.21	23	0.92	0.17	12	1.3%	0.01 [-0.12, 0.14]
Park 2007	1.26	0.16	19	1.26	0.2	21	1.4%	0.00 [-0.11, 0.11]
Racil 2013(2)	1.09	0.07	11	1	0.03	6	1.8%	0.09 [0.04, 0.14]
Ramezani 2017(1)	1.26	0.11	15	1.07	0.14	5	1.3%	0.19 [0.06, 0.32]
Ramezani 2017(2)	1.21	0.14	15	1.07	0.14	5	1.2%	0.14 [-0.00, 0.28]
Ramezani 2017(3)	1.19	0.14	15	1.07	0.14	5	1.2%	0.12 [-0.02, 0.26]
Saygın 2011	1.36	0.21	20	1.08	0.12	19	1.5%	0.28 [0.17, 0.39]
Seo 2012	0.72	1.15	10	0.95	1.15	10	0.1%	-0.23 [-1.24, 0.78]
Seo 2019	1.25	0.24	26	1.22	0.26	44	1.4%	0.03 [-0.09, 0.15]
Shalitin 2009	1.29	0.27	55	1.24	0.3	55	1.5%	0.05 [-0.06, 0.16]
Staiano 2017	1.26	0.18	20	1.33	0.22	18	1.3%	-0.07 [-0.20, 0.06]
Staiano 2018	1.22	0.19	22	1.31	0.19	23	1.4%	-0.09 [-0.20, 0.02]
Sun 2011	1.1	0.2	25	1.1	0.2	17	1.3%	0.00 [-0.12, 0.12]
Tsang 2009	0.96	0.21	11	0.91	0.23	8	0.9%	0.05 [-0.15, 0.25]
Wen 2008	1.31	0.18	24	0.95	0.27	13	1.1%	0.36 [0.20, 0.52]
Woo 2004	1.2	0.3	41	1.2	0.2	41	1.4%	0.00 [-0.11, 0.11]
Xinhui 2016	1.21	0.19	14	1.23	0.19	14	1.2%	-0.02 [-0.16, 0.12]
Yu 2020	1.5	0.3	99	1.3	0.2	72	1.7%	0.20 [0.12, 0.28]
Zehsaz 2016	1.17	0.31	16	1.16	0.26	16	0.9%	0.01 [-0.19, 0.21]
**Subtotal (95% CI)**			**1518**			**1268**	**67.1%**	**0.08 [0.05, 0.12]**
Heterogeneity: Tau² = 0.01; Chi² = 267.91, df = 55 (P < 0.00001); I² = 79%								
Test for overall effect: Z = 4.82 (P < 0.00001)								
**2.2.2 High intensity**								
Ahmadi 2020(1)	1.13	0.24	28	0.97	0.24	9	1.0%	0.16 [-0.02, 0.34]
Ahmadi 2020(2)	1.12	0.23	29	0.97	0.24	9	1.0%	0.15 [-0.03, 0.33]
Ahmadi 2020(3)	1.1	0.22	29	0.97	0.24	9	1.0%	0.13 [-0.05, 0.31]
Benson 2008	1.5	0.4	32	1.4	0.2	35	1.1%	0.10 [-0.05, 0.25]
Bezerra 2023	1.19	0.31	20	1.12	0.12	21	1.2%	0.07 [-0.08, 0.22]
Boer 2014(1)	1.54	0.29	17	1.45	0.4	7	0.5%	0.09 [-0.24, 0.42]
Chae 2010	1.37	0.25	19	1.31	0.28	19	1.1%	0.06 [-0.11, 0.23]
Chuensiri 2018(1)	1.19	0.26	11	1.34	0.26	5	0.6%	-0.15 [-0.42, 0.12]
Chuensiri 2018(2)	1.11	0.3	15	1.34	0.26	6	0.7%	-0.23 [-0.49, 0.03]
Dias 2018(1)	1.24	0.27	19	1.3	0.31	9	0.7%	-0.06 [-0.30, 0.18]
Faria 2020(2)	1.32	0.27	26	1.3	0.27	13	1.0%	0.02 [-0.16, 0.20]
Gulijianati 2022	1.66	0.28	47	1.38	0.27	46	1.4%	0.28 [0.17, 0.39]
Hongling 2012	1.2	0.2	30	1.2	0.3	30	1.3%	0.00 [-0.13, 0.13]
Horner 2015(2)	1.02	0.23	23	1.03	0.19	8	1.1%	-0.01 [-0.17, 0.15]
Ke 2024	1.4	0.2	10	1.3	0.3	10	0.8%	0.10 [-0.12, 0.32]
Lingling 2021	1.07	0.16	20	0.97	0.22	20	1.4%	0.10 [-0.02, 0.22]
Lira 2017(1)	1.18	0.22	26	1.12	0.26	16	1.2%	0.06 [-0.09, 0.21]
Lopes 2016	1.37	0.32	17	1.55	0.47	16	0.6%	-0.18 [-0.46, 0.10]
Meng 2022(1)	1.5	0.2	12	1.3	0.2	6	0.9%	0.20 [0.00, 0.40]
Migueles 2023	1.33	0.21	42	1.27	0.21	37	1.6%	0.06 [-0.03, 0.15]
Monteiro 2015(1)	0.98	0.26	18	0.94	0.31	8	0.7%	0.04 [-0.21, 0.29]
Monteiro 2015(2)	1.1	0.32	14	0.94	0.31	8	0.6%	0.16 [-0.11, 0.43]
Plavsic 2020	1.33	0.27	22	1.3	0.26	22	1.1%	0.03 [-0.13, 0.19]
Racil 2013(1)	1.08	0.08	11	1	0.03	6	1.8%	0.08 [0.03, 0.13]
Salus(2) 2022	1.07	0.19	14	0.96	0.11	14	1.4%	0.11 [-0.01, 0.23]
Savoye 2007	1.17	0.15	75	1.15	0.45	44	1.3%	0.02 [-0.12, 0.16]
Savoye 2011	1.19	0.18	75	1.12	0.41	44	1.3%	0.07 [-0.06, 0.20]
Tas 2023	1.04	0.23	31	1.14	0.23	6	0.9%	-0.10 [-0.30, 0.10]
Tjonna 2009	1.24	0.18	20	1.14	0.19	22	1.4%	0.10 [-0.01, 0.21]
Vasconcellos 2016	1.15	0.13	10	1	0.44	10	0.6%	0.15 [-0.13, 0.43]
Wong 2008	1.3	0.3	12	1.2	0.2	12	0.9%	0.10 [-0.10, 0.30]
Zehsaz 2017	1.17	0.31	16	1.16	0.26	16	0.9%	0.01 [-0.19, 0.21]
**Subtotal (95% CI)**			**790**			**543**	**32.9%**	**0.07 [0.04, 0.10]**
Heterogeneity: Tau² = 0.00; Chi² = 37.73, df = 31 (P = 0.19); I² = 18%								
Test for overall effect: Z = 4.73 (P < 0.00001)								
**Total (95% CI)**			**2308**			**1811**	**100.0%**	**0.08 [0.05, 0.10]**
Heterogeneity: Tau² = 0.01; Chi² = 306.87, df = 87 (P < 0.00001); I² = 72%								
Test for overall effect: Z = 5.95 (P < 0.00001)								
Test for subgroup differences: Chi² = 0.21, df = 1 (P = 0.65), I² = 0%								

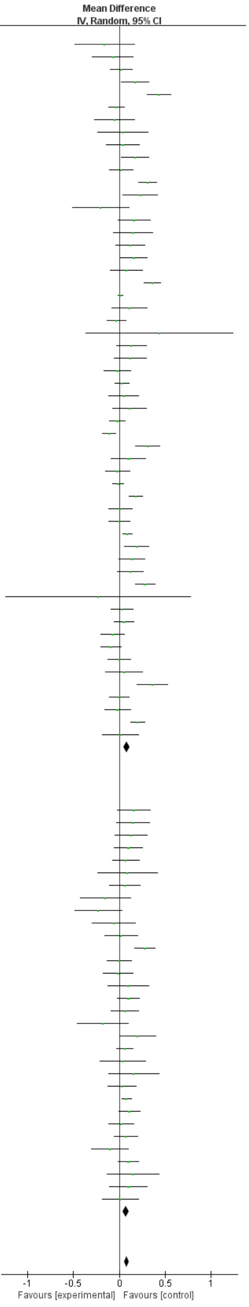

Figure S13.3. Forest plot of subgroup analyses by duration per session

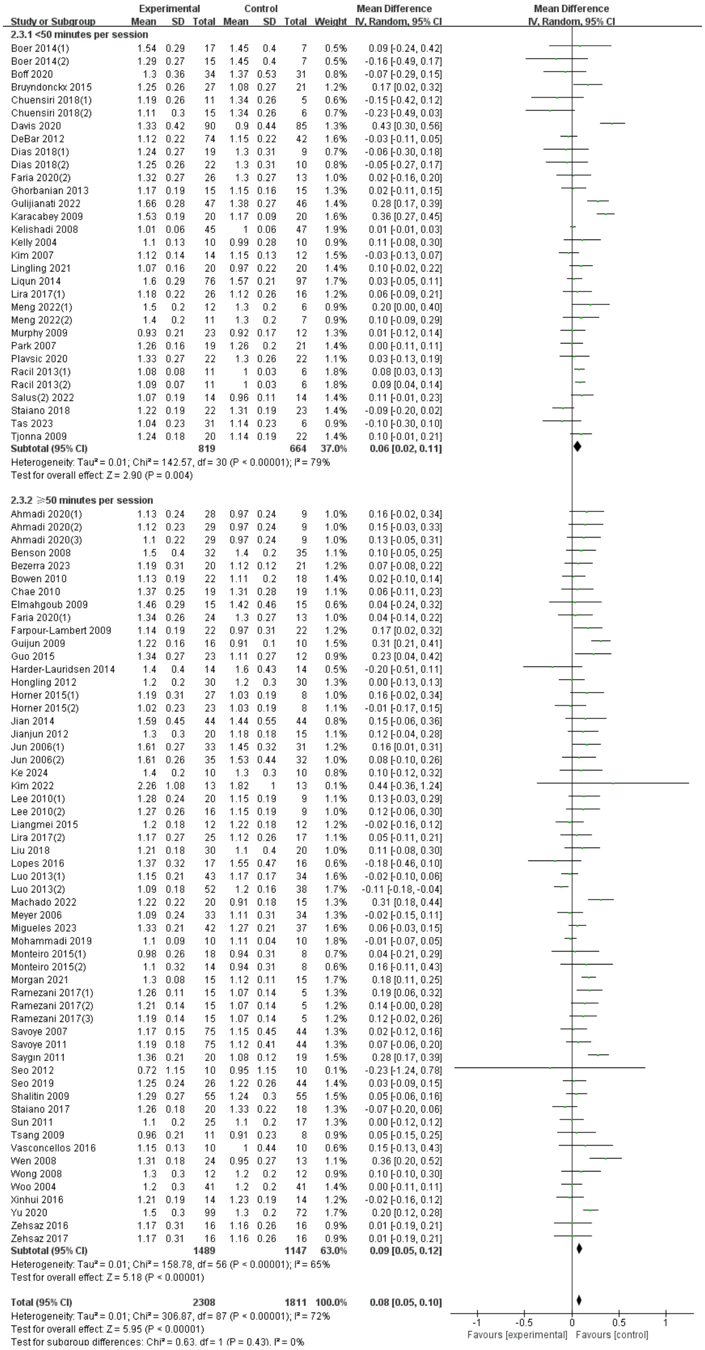

Figure S13.4. Forest plot of subgroup analyses by exercise frequency

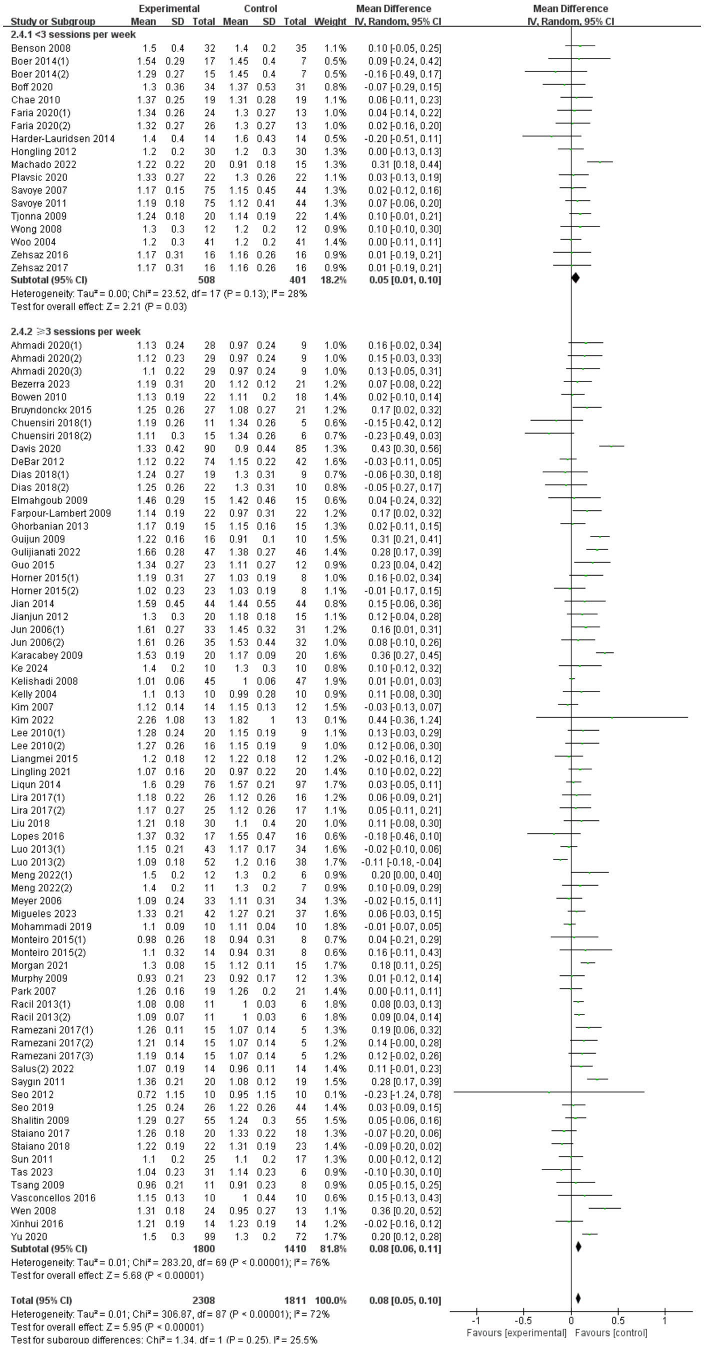

Figure S13.5. Forest plot of subgroup analyses by weekly exercise duration

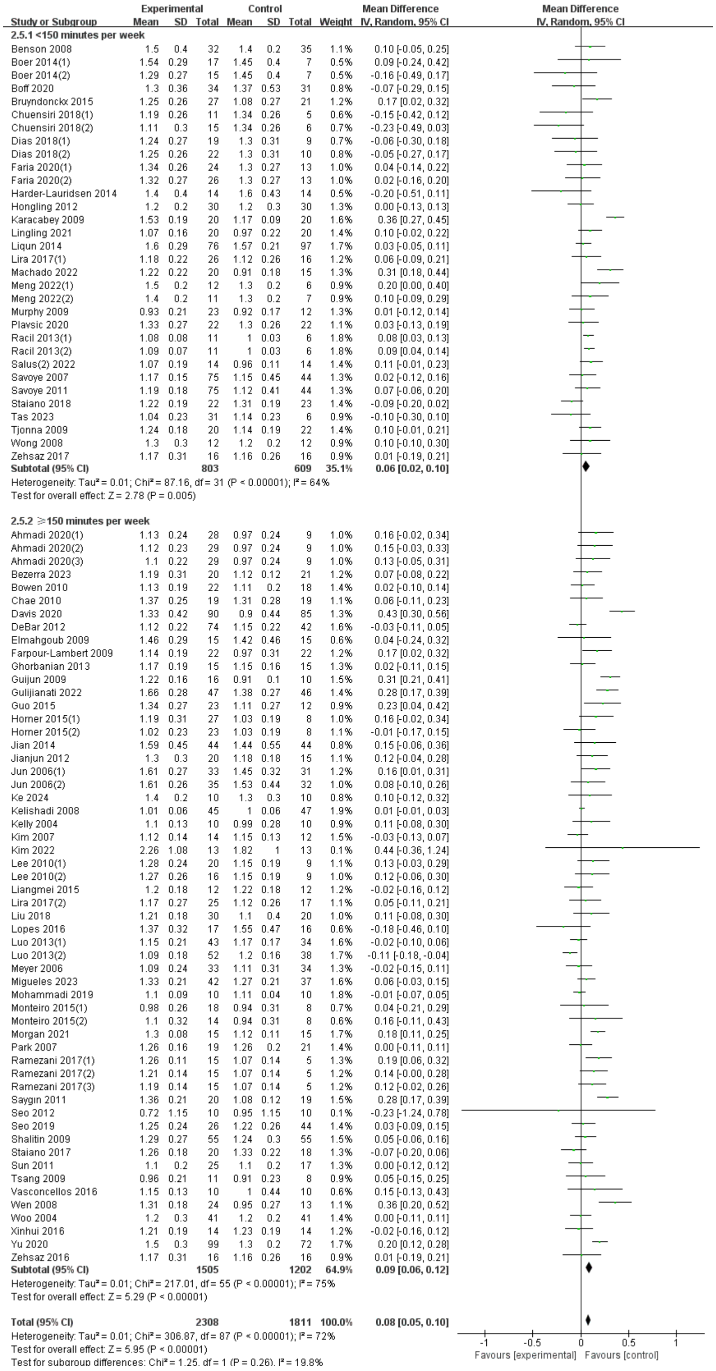

Figure S13.6. Forest plot of subgroup analyses by intervention duration

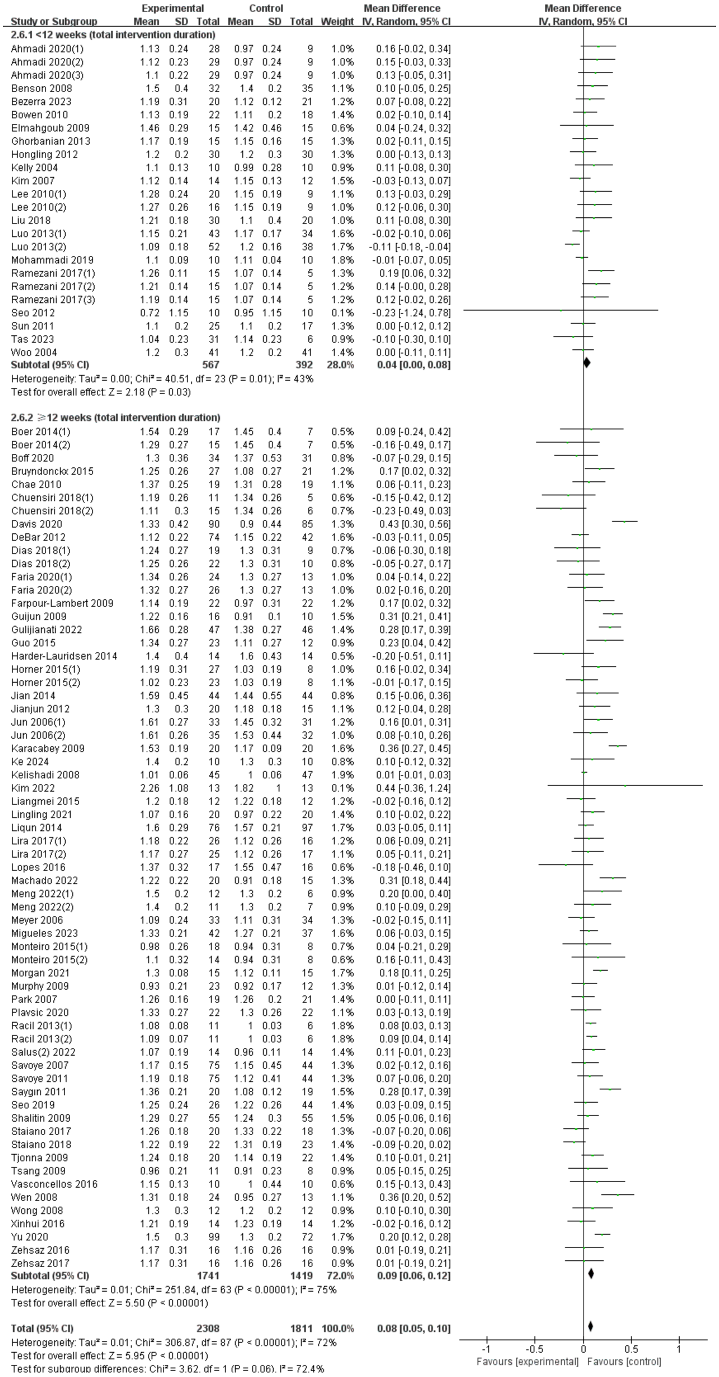

Figure S13.7. Forest plot of subgroup analyses by region

	Experimental			Control				Mean Difference
**Study or Subgroup**	**Mean**	**SD**	**Total**	**Mean**	**SD**	**Total**	**Weight**	**IV, Random, 95% CI**
**2.7.1 Asia**								
Ahmadi 2020(1)	1.13	0.24	28	0.97	0.24	9	1.0%	0.16 [-0.02, 0.34]
Ahmadi 2020(2)	1.12	0.23	29	0.97	0.24	9	1.0%	0.15 [-0.03, 0.33]
Ahmadi 2020(3)	1.1	0.22	29	0.97	0.24	9	1.0%	0.13 [-0.05, 0.31]
Bowen 2010	1.13	0.19	22	1.11	0.2	18	1.4%	0.02 [-0.10, 0.14]
Chae 2010	1.37	0.25	19	1.31	0.28	19	1.1%	0.06 [-0.11, 0.23]
Chuensiri 2018(1)	1.19	0.26	11	1.34	0.26	5	0.6%	-0.15 [-0.42, 0.12]
Chuensiri 2018(2)	1.11	0.3	15	1.34	0.26	6	0.7%	-0.23 [-0.49, 0.03]
Ghorbanian 2013	1.17	0.19	15	1.15	0.16	15	1.3%	0.02 [-0.11, 0.15]
Guijun 2009	1.22	0.16	16	0.91	0.1	10	1.5%	0.31 [0.21, 0.41]
Gulijianati 2022	1.66	0.28	47	1.38	0.27	46	1.4%	0.28 [0.17, 0.39]
Guo 2015	1.34	0.27	23	1.11	0.27	12	0.9%	0.23 [0.04, 0.42]
Hongling 2012	1.2	0.2	30	1.2	0.3	30	1.3%	0.00 [-0.13, 0.13]
Jian 2014	1.59	0.45	44	1.44	0.55	44	0.8%	0.15 [-0.06, 0.36]
Jianjun 2012	1.3	0.3	20	1.18	0.18	15	1.1%	0.12 [-0.04, 0.28]
Jun 2006(1)	1.61	0.27	33	1.45	0.32	31	1.2%	0.16 [0.01, 0.31]
Jun 2006(2)	1.61	0.26	35	1.53	0.44	32	1.0%	0.08 [-0.10, 0.26]
Karacabey 2009	1.53	0.19	20	1.17	0.09	20	1.6%	0.36 [0.27, 0.45]
Ke 2024	1.4	0.2	10	1.3	0.3	10	0.8%	0.10 [-0.12, 0.32]
Kelishadi 2008	1.01	0.06	45	1	0.06	47	1.9%	0.01 [-0.01, 0.03]
Kim 2007	1.12	0.14	14	1.15	0.13	12	1.5%	-0.03 [-0.13, 0.07]
Kim 2022	2.26	1.08	13	1.82	1	13	0.1%	0.44 [-0.36, 1.24]
Lee 2010(1)	1.28	0.24	20	1.15	0.19	9	1.1%	0.13 [-0.03, 0.29]
Lee 2010(2)	1.27	0.26	16	1.15	0.19	9	1.0%	0.12 [-0.06, 0.30]
Liangmei 2015	1.2	0.18	12	1.22	0.18	12	1.2%	-0.02 [-0.16, 0.12]
Lingling 2021	1.07	0.16	20	0.97	0.22	20	1.4%	0.10 [-0.02, 0.22]
Liqun 2014	1.6	0.29	76	1.57	0.21	97	1.7%	0.03 [-0.05, 0.11]
Liu 2018	1.21	0.18	30	1.1	0.4	20	1.0%	0.11 [-0.08, 0.30]
Luo 2013(1)	1.15	0.21	43	1.17	0.17	34	1.6%	-0.02 [-0.10, 0.06]
Luo 2013(2)	1.09	0.18	52	1.2	0.16	38	1.7%	-0.11 [-0.18, -0.04]
Meng 2022(1)	1.5	0.2	12	1.3	0.2	6	0.9%	0.20 [0.00, 0.40]
Meng 2022(2)	1.4	0.2	11	1.3	0.2	7	0.9%	0.10 [-0.09, 0.29]
Mohammadi 2019	1.1	0.09	10	1.11	0.04	10	1.8%	-0.01 [-0.07, 0.05]
Park 2007	1.26	0.16	19	1.26	0.2	21	1.4%	0.00 [-0.11, 0.11]
Ramezani 2017(1)	1.26	0.11	15	1.07	0.14	5	1.3%	0.19 [0.06, 0.32]
Ramezani 2017(2)	1.21	0.14	15	1.07	0.14	5	1.2%	0.14 [-0.00, 0.28]
Ramezani 2017(3)	1.19	0.14	15	1.07	0.14	5	1.2%	0.12 [-0.02, 0.26]
Saygın 2011	1.36	0.21	20	1.08	0.12	19	1.5%	0.28 [0.17, 0.39]
Seo 2012	0.72	1.15	10	0.95	1.15	10	0.1%	-0.23 [-1.24, 0.78]
Seo 2019	1.25	0.24	26	1.22	0.26	44	1.4%	0.03 [-0.09, 0.15]
Shalitin 2009	1.29	0.27	55	1.24	0.3	55	1.5%	0.05 [-0.06, 0.16]
Sun 2011	1.1	0.2	25	1.1	0.2	17	1.3%	0.00 [-0.12, 0.12]
Wen 2008	1.31	0.18	24	0.95	0.27	13	1.1%	0.36 [0.20, 0.52]
Wong 2008	1.3	0.3	12	1.2	0.2	12	0.9%	0.10 [-0.10, 0.30]
Woo 2004	1.2	0.3	41	1.2	0.2	41	1.4%	0.00 [-0.11, 0.11]
Xinhui 2016	1.21	0.19	14	1.23	0.19	14	1.2%	-0.02 [-0.16, 0.12]
Yu 2020	1.5	0.3	99	1.3	0.2	72	1.7%	0.20 [0.12, 0.28]
Zehsaz 2016	1.17	0.31	16	1.16	0.26	16	0.9%	0.01 [-0.19, 0.21]
Zehsaz 2017	1.17	0.31	16	1.16	0.26	16	0.9%	0.01 [-0.19, 0.21]
**Subtotal (95% CI)**			**1242**			**1039**	**56.4%**	**0.09 [0.05, 0.13]**
Heterogeneity: Tau^2 = 0.01; Chi^2 = 207.24, df = 47 (P < 0.00001); I^2 = 77%								
Test for overall effect: Z = 4.82 (P < 0.00001)								
**2.7.2 Europe**								
Boer 2014(1)	1.54	0.29	17	1.45	0.4	7	0.5%	0.09 [-0.24, 0.42]
Boer 2014(2)	1.29	0.27	15	1.45	0.4	7	0.5%	-0.16 [-0.49, 0.17]
Bruyndonckx 2015	1.25	0.26	27	1.08	0.27	21	1.2%	0.17 [0.02, 0.32]
Elmahgoub 2009	1.46	0.29	15	1.42	0.46	15	0.6%	0.04 [-0.24, 0.32]
Farpour-Lambert 2009	1.14	0.19	22	0.97	0.31	22	1.2%	0.17 [0.02, 0.32]
Harder-Lauridsen 2014	1.4	0.4	14	1.6	0.43	14	0.5%	-0.20 [-0.51, 0.11]
Meyer 2006	1.09	0.24	33	1.11	0.31	34	1.3%	-0.02 [-0.15, 0.11]
Migueles 2023	1.33	0.21	42	1.27	0.21	37	1.6%	0.06 [-0.03, 0.15]
Plavsic 2020	1.33	0.27	22	1.3	0.26	22	1.1%	0.03 [-0.13, 0.19]
Salus(2) 2022	1.07	0.19	14	0.96	0.11	14	1.4%	0.11 [-0.01, 0.23]
Tjonna 2009	1.24	0.18	20	1.14	0.19	22	1.4%	0.10 [-0.01, 0.21]
**Subtotal (95% CI)**			**241**			**215**	**11.2%**	**0.07 [0.02, 0.12]**
Heterogeneity: Tau^2 = 0.00; Chi^2 = 11.07, df = 10 (P = 0.35); I^2 = 10%								
Test for overall effect: Z = 2.88 (P = 0.004)								
**2.7.3 Africa**								
Morgan 2021	1.3	0.08	15	1.12	0.11	15	1.7%	0.18 [0.11, 0.25]
Racil 2013(1)	1.08	0.08	11	1	0.03	6	1.8%	0.08 [0.03, 0.13]
Racil 2013(2)	1.09	0.07	11	1	0.03	6	1.8%	0.09 [0.04, 0.14]
**Subtotal (95% CI)**			**37**			**27**	**5.4%**	**0.11 [0.06, 0.17]**
Heterogeneity: Tau^2 = 0.00; Chi^2 = 5.79, df = 2 (P = 0.06); I^2 = 65%								
Test for overall effect: Z = 4.00 (P < 0.0001)								
**2.7.4 North America**								
Davis 2020	1.33	0.42	90	0.9	0.44	85	1.3%	0.43 [0.30, 0.56]
DeBar 2012	1.12	0.22	74	1.15	0.22	42	1.6%	-0.03 [-0.11, 0.05]
Horner 2015(1)	1.19	0.31	27	1.03	0.19	8	1.0%	0.16 [-0.02, 0.34]
Horner 2015(2)	1.02	0.23	23	1.03	0.19	8	1.1%	-0.01 [-0.17, 0.15]
Kelly 2004	1.1	0.13	10	0.99	0.28	10	0.9%	0.11 [-0.08, 0.30]
Murphy 2009	0.93	0.21	23	0.92	0.17	12	1.3%	0.01 [-0.12, 0.14]
Savoye 2007	1.17	0.15	75	1.15	0.45	44	1.3%	0.02 [-0.12, 0.16]
Savoye 2011	1.19	0.18	75	1.12	0.41	44	1.3%	0.07 [-0.06, 0.20]
Staiano 2017	1.26	0.18	20	1.33	0.22	18	1.3%	-0.07 [-0.20, 0.06]
Staiano 2018	1.22	0.19	22	1.31	0.19	23	1.4%	-0.09 [-0.20, 0.02]
Tas 2023	1.04	0.23	31	1.14	0.23	6	0.9%	-0.10 [-0.30, 0.10]
**Subtotal (95% CI)**			**470**			**300**	**13.5%**	**0.04 [-0.05, 0.14]**
Heterogeneity: Tau^2 = 0.02; Chi^2 = 51.61, df = 10 (P < 0.00001); I^2 = 81%								
Test for overall effect: Z = 0.95 (P = 0.34)								
**2.7.5 South America**								
Bezerra 2023	1.19	0.31	20	1.12	0.12	21	1.2%	0.07 [-0.08, 0.22]
Boff 2020	1.3	0.36	34	1.37	0.53	31	0.8%	-0.07 [-0.29, 0.15]
Faria 2020(1)	1.34	0.26	24	1.3	0.27	13	1.0%	0.04 [-0.14, 0.22]
Faria 2020(2)	1.32	0.27	26	1.3	0.27	13	1.0%	0.02 [-0.16, 0.20]
Lira 2017(1)	1.18	0.22	26	1.12	0.26	16	1.2%	0.06 [-0.09, 0.21]
Lira 2017(2)	1.17	0.27	25	1.12	0.26	17	1.1%	0.05 [-0.11, 0.21]
Lopes 2016	1.37	0.32	17	1.55	0.47	16	0.6%	-0.18 [-0.46, 0.10]
Machado 2022	1.22	0.22	20	0.91	0.18	15	1.3%	0.31 [0.18, 0.44]
Monteiro 2015(1)	0.98	0.26	18	0.94	0.31	8	0.7%	0.04 [-0.21, 0.29]
Monteiro 2015(2)	1.1	0.32	14	0.94	0.31	8	0.6%	0.16 [-0.11, 0.43]
Vasconcellos 2016	1.15	0.13	10	1	0.44	10	0.6%	0.15 [-0.13, 0.43]
**Subtotal (95% CI)**			**234**			**168**	**10.0%**	**0.07 [-0.01, 0.15]**
Heterogeneity: Tau^2 = 0.01; Chi^2 = 18.14, df = 10 (P = 0.05); I^2 = 45%								
Test for overall effect: Z = 1.83 (P = 0.07)								
**2.7.6 Oceania**								
Benson 2008	1.5	0.4	32	1.4	0.2	35	1.1%	0.10 [-0.05, 0.25]
Dias 2018(1)	1.24	0.27	19	1.3	0.31	9	0.7%	-0.06 [-0.30, 0.18]
Dias 2018(2)	1.25	0.26	22	1.3	0.31	10	0.8%	-0.05 [-0.27, 0.17]
Tsang 2009	0.96	0.21	11	0.91	0.23	8	0.9%	0.05 [-0.15, 0.25]
**Subtotal (95% CI)**			**84**			**62**	**3.6%**	**0.03 [-0.07, 0.13]**
Heterogeneity: Tau^2 = 0.00; Chi^2 = 1.90, df = 3 (P = 0.59); I^2 = 0%								
Test for overall effect: Z = 0.64 (P = 0.52)								
**Total (95% CI)**			**2308**			**1811**	**100.0%**	**0.08 [0.05, 0.10]**
Heterogeneity: Tau^2 = 0.01; Chi^2 = 306.87, df = 87 (P < 0.00001); I^2 = 72%								
Test for overall effect: Z = 5.95 (P < 0.00001)								
Test for subgroup differences: Chi^2 = 3.21, df = 5 (P = 0.67), I^2 = 0%								

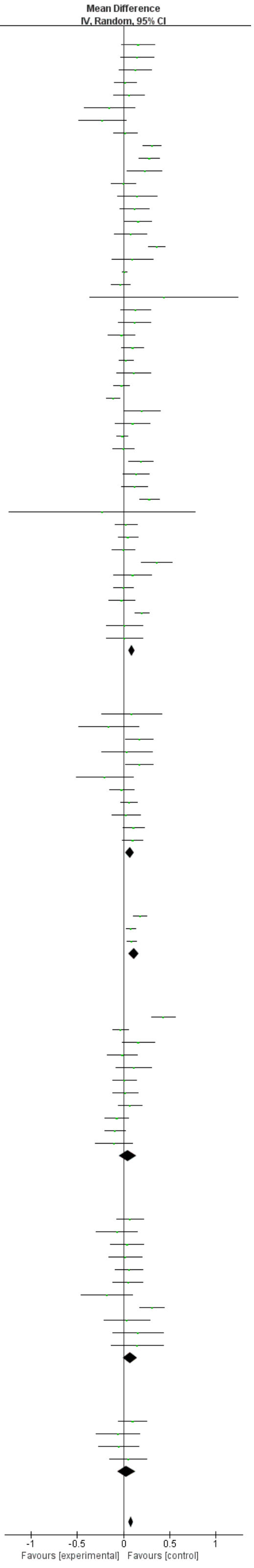

Figure S13.8. Forest plot of subgroup analyses by economic status

	Experimental			Control				Mean Difference
**Study or Subgroup**	**Mean**	**SD**	**Total**	**Mean**	**SD**	**Total**	**Weight**	**IV, Random, 95% CI**
**2.8.1 Lower-middle-income countries**								
Morgan 2021	1.3	0.08	15	1.12	0.11	15	1.7%	0.18 [0.11, 0.25]
Racil 2013(1)	1.08	0.08	11	1	0.03	6	1.8%	0.08 [0.03, 0.13]
Racil 2013(2)	1.09	0.07	11	1	0.03	6	1.8%	0.09 [0.04, 0.14]
**Subtotal (95% CI)**			**37**			**27**	**5.4%**	**0.11 [0.06, 0.17]**
Heterogeneity: Tau^2 = 0.00; Chi^2 = 5.79, df = 2 (P = 0.06); I^2 = 65%								
Test for overall effect: Z = 4.00 (P < 0.0001)								
**2.8.2 Upper-middle-income countries**								
Ahmadi 2020(1)	1.13	0.24	28	0.97	0.24	9	1.0%	0.16 [-0.02, 0.34]
Ahmadi 2020(2)	1.12	0.23	29	0.97	0.24	9	1.0%	0.15 [-0.03, 0.33]
Ahmadi 2020(3)	1.1	0.22	29	0.97	0.24	9	1.0%	0.13 [-0.05, 0.31]
Bezerra 2023	1.19	0.31	20	1.12	0.12	21	1.2%	0.07 [-0.08, 0.22]
Boff 2020	1.3	0.36	34	1.37	0.53	31	0.8%	-0.07 [-0.29, 0.15]
Bowen 2010	1.13	0.19	22	1.11	0.2	18	1.4%	0.02 [-0.10, 0.14]
Chuensiri 2018(1)	1.19	0.26	11	1.34	0.26	5	0.6%	-0.15 [-0.42, 0.12]
Chuensiri 2018(2)	1.11	0.3	15	1.34	0.26	6	0.7%	-0.23 [-0.49, 0.03]
Faria 2020(1)	1.34	0.26	24	1.3	0.27	13	1.0%	0.04 [-0.14, 0.22]
Faria 2020(2)	1.32	0.27	26	1.3	0.27	13	1.0%	0.02 [-0.16, 0.20]
Ghorbanian 2013	1.17	0.19	15	1.15	0.16	15	1.3%	0.02 [-0.11, 0.15]
Guijun 2009	1.22	0.16	16	0.91	0.1	10	1.5%	0.31 [0.21, 0.41]
Gulijianati 2022	1.66	0.28	47	1.38	0.27	46	1.4%	0.28 [0.17, 0.39]
Guo 2015	1.34	0.27	23	1.11	0.27	12	0.9%	0.23 [0.04, 0.42]
Hongling 2012	1.2	0.2	30	1.2	0.3	30	1.3%	0.00 [-0.13, 0.13]
Jian 2014	1.59	0.45	44	1.44	0.55	44	0.8%	0.15 [-0.06, 0.36]
Jianjun 2012	1.3	0.3	20	1.18	0.18	15	1.1%	0.12 [-0.04, 0.28]
Jun 2006(1)	1.61	0.27	33	1.45	0.32	31	1.2%	0.16 [0.01, 0.31]
Jun 2006(2)	1.61	0.26	35	1.53	0.44	32	1.0%	0.08 [-0.10, 0.26]
Karacabey 2009	1.53	0.19	20	1.17	0.09	20	1.6%	0.36 [0.27, 0.45]
Ke 2024	1.4	0.2	10	1.3	0.3	10	0.8%	0.10 [-0.12, 0.32]
Kelishadi 2008	1.01	0.06	45	1	0.06	47	1.9%	0.01 [-0.01, 0.03]
Liangmei 2015	1.2	0.18	12	1.22	0.18	12	1.2%	-0.02 [-0.16, 0.12]
Lingling 2021	1.07	0.16	20	0.97	0.22	20	1.4%	0.10 [-0.02, 0.22]
Liqun 2014	1.6	0.29	76	1.57	0.21	97	1.7%	0.03 [-0.05, 0.11]
Lira 2017(1)	1.18	0.22	26	1.12	0.26	16	1.2%	0.06 [-0.09, 0.21]
Lira 2017(2)	1.17	0.27	25	1.12	0.26	17	1.1%	0.05 [-0.11, 0.21]
Liu 2018	1.21	0.18	30	1.1	0.4	20	1.0%	0.11 [-0.08, 0.30]
Lopes 2016	1.37	0.32	17	1.55	0.47	16	0.6%	-0.18 [-0.46, 0.10]
Luo 2013(1)	1.15	0.21	43	1.17	0.17	34	1.6%	-0.02 [-0.10, 0.06]
Luo 2013(2)	1.09	0.18	52	1.2	0.16	38	1.7%	-0.11 [-0.18, -0.04]
Machado 2022	1.22	0.22	20	0.91	0.18	15	1.3%	0.31 [0.18, 0.44]
Meng 2022(1)	1.5	0.2	12	1.3	0.2	6	0.9%	0.20 [0.00, 0.40]
Meng 2022(2)	1.4	0.2	11	1.3	0.2	7	0.9%	0.10 [-0.09, 0.29]
Mohammadi 2019	1.1	0.09	10	1.11	0.04	10	1.8%	-0.01 [-0.07, 0.05]
Monteiro 2015(1)	0.98	0.26	18	0.94	0.31	8	0.7%	0.04 [-0.21, 0.29]
Monteiro 2015(2)	1.1	0.32	14	0.94	0.31	8	0.6%	0.16 [-0.11, 0.43]
Plavsic 2020	1.33	0.27	22	1.3	0.26	22	1.1%	0.03 [-0.13, 0.19]
Ramezani 2017(1)	1.26	0.11	15	1.07	0.14	5	1.3%	0.19 [0.06, 0.32]
Ramezani 2017(2)	1.21	0.14	15	1.07	0.14	5	1.2%	0.14 [-0.00, 0.28]
Ramezani 2017(3)	1.19	0.14	15	1.07	0.14	5	1.2%	0.12 [-0.02, 0.26]
Saygın 2011	1.36	0.21	20	1.08	0.12	19	1.5%	0.28 [0.17, 0.39]
Sun 2011	1.1	0.2	25	1.1	0.2	17	1.3%	0.00 [-0.12, 0.12]
Vasconcellos 2016	1.15	0.13	10	1	0.44	10	0.6%	0.15 [-0.13, 0.43]
Wen 2008	1.31	0.18	24	0.95	0.27	13	1.1%	0.36 [0.20, 0.52]
Woo 2004	1.2	0.3	41	1.2	0.2	41	1.4%	0.00 [-0.11, 0.11]
Xinhui 2016	1.21	0.19	14	1.23	0.19	14	1.2%	-0.02 [-0.16, 0.12]
Yu 2020	1.5	0.3	99	1.3	0.2	72	1.7%	0.20 [0.12, 0.28]
Zehsaz 2016	1.17	0.31	16	1.16	0.26	16	0.9%	0.01 [-0.19, 0.21]
Zehsaz 2017	1.17	0.31	16	1.16	0.26	16	0.9%	0.01 [-0.19, 0.21]
**Subtotal (95% CI)**			**1294**			**1025**	**57.6%**	**0.09 [0.05, 0.13]**
Heterogeneity: Tau^2 = 0.01; Chi^2 = 219.96, df = 49 (P < 0.00001); I^2 = 78%								
Test for overall effect: Z = 4.82 (P < 0.00001)								
**2.8.3 High-income countries**								
Benson 2008	1.5	0.4	32	1.4	0.2	35	1.1%	0.10 [-0.05, 0.25]
Boer 2014(1)	1.54	0.29	17	1.45	0.4	7	0.5%	0.09 [-0.24, 0.42]
Boer 2014(2)	1.29	0.27	15	1.45	0.4	7	0.5%	-0.16 [-0.49, 0.17]
Bruyndonckx 2015	1.25	0.26	27	1.08	0.27	21	1.2%	0.17 [0.02, 0.32]
Chae 2010	1.37	0.25	19	1.31	0.28	19	1.1%	0.06 [-0.11, 0.23]
Davis 2020	1.33	0.42	90	0.9	0.44	85	1.3%	0.43 [0.30, 0.56]
DeBar 2012	1.12	0.22	74	1.15	0.22	42	1.6%	-0.03 [-0.11, 0.05]
Dias 2018(1)	1.24	0.27	19	1.3	0.31	9	0.7%	-0.06 [-0.30, 0.18]
Dias 2018(2)	1.25	0.26	22	1.3	0.31	10	0.8%	-0.05 [-0.27, 0.17]
Elmahgoub 2009	1.46	0.29	15	1.42	0.46	15	0.6%	0.04 [-0.24, 0.32]
Farpour-Lambert 2009	1.14	0.19	22	0.97	0.31	22	1.2%	0.17 [0.02, 0.32]
Harder-Lauridsen 2014	1.4	0.4	14	1.6	0.43	14	0.5%	-0.20 [-0.51, 0.11]
Horner 2015(1)	1.19	0.31	27	1.03	0.19	8	1.0%	0.16 [-0.02, 0.34]
Horner 2015(2)	1.02	0.23	23	1.03	0.19	8	1.1%	-0.01 [-0.17, 0.15]
Kelly 2004	1.1	0.13	10	0.99	0.28	10	0.9%	0.11 [-0.08, 0.30]
Kim 2007	1.12	0.14	14	1.15	0.13	12	1.5%	-0.03 [-0.13, 0.07]
Kim 2022	2.26	1.08	13	1.82	1	13	0.1%	0.44 [-0.36, 1.24]
Lee 2010(1)	1.28	0.24	20	1.15	0.19	9	1.1%	0.13 [-0.03, 0.29]
Lee 2010(2)	1.27	0.26	16	1.15	0.19	9	1.0%	0.12 [-0.06, 0.30]
Meyer 2006	1.09	0.24	33	1.11	0.31	34	1.3%	-0.02 [-0.15, 0.11]
Migueles 2023	1.33	0.21	42	1.27	0.21	37	1.6%	0.06 [-0.03, 0.15]
Murphy 2009	0.93	0.21	23	0.92	0.17	12	1.3%	0.01 [-0.12, 0.14]
Park 2007	1.26	0.16	19	1.26	0.2	21	1.4%	0.00 [-0.11, 0.11]
Salus(2) 2022	1.07	0.19	14	0.96	0.11	14	1.4%	0.11 [-0.01, 0.23]
Savoye 2007	1.17	0.15	75	1.15	0.45	44	1.3%	0.02 [-0.12, 0.16]
Savoye 2011	1.19	0.18	75	1.12	0.41	44	1.3%	0.07 [-0.06, 0.20]
Seo 2012	0.72	1.15	10	0.95	1.15	10	0.1%	-0.23 [-1.24, 0.78]
Seo 2019	1.25	0.24	26	1.22	0.26	44	1.4%	0.03 [-0.09, 0.15]
Shalitin 2009	1.29	0.27	55	1.24	0.3	55	1.5%	0.05 [-0.06, 0.16]
Staiano 2017	1.26	0.18	20	1.33	0.22	18	1.3%	-0.07 [-0.20, 0.06]
Staiano 2018	1.22	0.19	22	1.31	0.19	23	1.4%	-0.09 [-0.20, 0.02]
Tas 2023	1.04	0.23	31	1.14	0.23	6	0.9%	-0.10 [-0.30, 0.10]
Tjonna 2009	1.24	0.18	20	1.14	0.19	22	1.4%	0.10 [-0.01, 0.21]
Tsang 2009	0.96	0.21	11	0.91	0.23	8	0.9%	0.05 [-0.15, 0.25]
Wong 2008	1.3	0.3	12	1.2	0.2	12	0.9%	0.10 [-0.10, 0.30]
**Subtotal (95% CI)**			**977**			**759**	**37.0%**	**0.05 [0.01, 0.09]**
Heterogeneity: Tau^2 = 0.01; Chi^2 = 72.25, df = 34 (P = 0.0001); I^2 = 53%								
Test for overall effect: Z = 2.55 (P = 0.01)								
**Total (95% CI)**			**2308**			**1811**	**100.0%**	**0.08 [0.05, 0.10]**
Heterogeneity: Tau^2 = 0.01; Chi^2 = 306.87, df = 87 (P < 0.00001); I^2 = 72%								
Test for overall effect: Z = 5.95 (P < 0.00001)								
Test for subgroup differences: Chi^2 = 4.08, df = 2 (P = 0.13), I^2 = 51.0%								

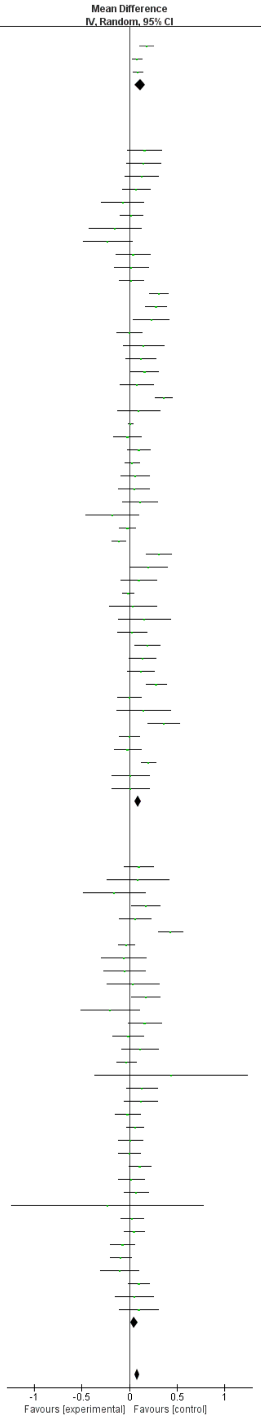

Figure S13.9. Forest plot of subgroup analyses by weight status

Study or Subgroup	Experimental Mean	Experimental SD	Experimental Total	Control Mean	Control SD	Control Total	Weight	Mean Difference IV, Random, 95% CI
**2.9.1 Overweight**								
Bezerra 2023	1.19	0.31	20	1.12	0.12	21	1.6%	0.07 [-0.08, 0.22]
Boer 2014(1)	1.54	0.29	17	1.45	0.4	7	0.6%	0.09 [-0.24, 0.42]
Boer 2014(2)	1.29	0.27	15	1.45	0.4	7	0.6%	-0.16 [-0.49, 0.17]
DeBar 2012	1.12	0.22	74	1.15	0.22	42	2.2%	-0.03 [-0.11, 0.05]
Faria 2020(1)	1.34	0.26	24	1.3	0.27	13	1.3%	0.04 [-0.14, 0.22]
Faria 2020(2)	1.32	0.27	26	1.3	0.27	13	1.3%	0.02 [-0.16, 0.20]
Gulijianati 2022	1.66	0.28	47	1.38	0.27	46	1.9%	0.28 [0.17, 0.39]
Harder-Lauridsen 2014	1.4	0.4	14	1.6	0.43	14	0.7%	-0.20 [-0.51, 0.11]
Kelly 2004	1.1	0.13	10	0.99	0.28	10	1.2%	0.11 [-0.08, 0.30]
Lopes 2016	1.37	0.32	17	1.55	0.47	16	0.8%	-0.18 [-0.46, 0.10]
Mohammadi 2019	1.1	0.09	10	1.11	0.04	10	2.3%	-0.01 [-0.07, 0.05]
Murphy 2009	0.93	0.21	23	0.92	0.17	12	1.7%	0.01 [-0.12, 0.14]
Sun 2011	1.1	0.2	25	1.1	0.2	17	1.8%	0.00 [-0.12, 0.12]
**Subtotal (95% CI)**			**322**			**228**	**17.9%**	**0.03 [-0.04, 0.09]**
Heterogeneity: Tau^2 = 0.01; Chi^2 = 29.85, df = 12 (P = 0.003); I^2 = 60%								
Test for overall effect: Z = 0.81 (P = 0.42)								
**2.9.2 Obesity**								
Bruyndonckx 2015	1.25	0.26	27	1.08	0.27	21	1.5%	0.17 [0.02, 0.32]
Chae 2010	1.37	0.25	19	1.31	0.28	19	1.4%	0.06 [-0.11, 0.23]
Chuensiri 2018(1)	1.19	0.26	11	1.34	0.26	5	0.8%	-0.15 [-0.42, 0.12]
Chuensiri 2018(2)	1.11	0.3	15	1.34	0.26	6	0.8%	-0.23 [-0.49, 0.03]
Dias 2018(1)	1.24	0.27	19	1.3	0.31	9	0.9%	-0.06 [-0.30, 0.18]
Dias 2018(2)	1.25	0.26	22	1.3	0.31	10	1.0%	-0.05 [-0.27, 0.17]
Farpour-Lambert 2009	1.14	0.19	22	0.97	0.31	22	1.5%	0.17 [0.02, 0.32]
Guijun 2009	1.22	0.16	16	0.91	0.1	10	2.0%	0.31 [0.21, 0.41]
Hongling 2012	1.2	0.2	30	1.2	0.3	30	1.7%	0.00 [-0.13, 0.13]
Horner 2015(1)	1.19	0.31	27	1.03	0.19	8	1.3%	0.16 [-0.02, 0.34]
Horner 2015(2)	1.02	0.23	23	1.03	0.19	8	1.4%	-0.01 [-0.17, 0.15]
Jian 2014	1.59	0.45	44	1.44	0.55	44	1.1%	0.15 [-0.06, 0.36]
Jianjun 2012	1.3	0.3	20	1.18	0.18	15	1.5%	0.12 [-0.04, 0.28]
Jun 2006(1)	1.61	0.27	33	1.45	0.32	31	1.6%	0.16 [0.01, 0.31]
Jun 2006(2)	1.61	0.26	35	1.53	0.44	32	1.3%	0.08 [-0.10, 0.26]
Karacabey 2009	1.53	0.19	20	1.17	0.09	20	2.1%	0.36 [0.27, 0.45]
Ke 2024	1.4	0.2	10	1.3	0.3	10	1.0%	0.10 [-0.12, 0.32]
Kelishadi 2008	1.01	0.06	45	1	0.06	47	2.6%	0.01 [-0.01, 0.03]
Kim 2007	1.12	0.14	14	1.15	0.13	12	2.0%	-0.03 [-0.13, 0.07]
Lee 2010(1)	1.28	0.24	20	1.15	0.19	9	1.4%	0.13 [-0.03, 0.29]
Lee 2010(2)	1.27	0.26	16	1.15	0.19	9	1.3%	0.12 [-0.06, 0.30]
Liangmei 2015	1.2	0.18	12	1.22	0.18	12	1.6%	-0.02 [-0.16, 0.12]
Lira 2017(1)	1.18	0.22	26	1.12	0.26	16	1.5%	0.06 [-0.09, 0.21]
Lira 2017(2)	1.17	0.27	25	1.12	0.26	17	1.4%	0.05 [-0.11, 0.21]
Liu 2018	1.21	0.18	30	1.1	0.4	20	1.2%	0.11 [-0.08, 0.30]
Luo 2013(1)	1.15	0.21	43	1.17	0.17	34	2.1%	-0.02 [-0.10, 0.06]
Luo 2013(2)	1.09	0.18	52	1.2	0.16	38	2.3%	-0.11 [-0.18, -0.04]
Meng 2022(1)	1.5	0.2	12	1.3	0.2	6	1.2%	0.20 [0.00, 0.40]
Meng 2022(2)	1.4	0.2	11	1.3	0.2	7	1.2%	0.10 [-0.09, 0.29]
Meyer 2006	1.09	0.24	33	1.11	0.31	34	1.7%	-0.02 [-0.15, 0.11]
Monteiro 2015(1)	0.98	0.26	18	0.94	0.31	8	0.9%	0.04 [-0.21, 0.29]
Monteiro 2015(2)	1.1	0.32	14	0.94	0.31	8	0.8%	0.16 [-0.11, 0.43]
Morgan 2021	1.3	0.08	15	1.12	0.11	15	2.3%	0.18 [0.11, 0.25]
Park 2007	1.26	0.16	19	1.26	0.2	21	1.9%	0.00 [-0.11, 0.11]
Plavsic 2020	1.33	0.27	22	1.3	0.26	22	1.5%	0.03 [-0.13, 0.19]
Racil 2013(1)	1.08	0.08	11	1	0.03	6	2.4%	0.08 [0.03, 0.13]
Racil 2013(2)	1.09	0.07	11	1	0.03	6	2.4%	0.09 [0.04, 0.14]
Ramezani 2017(1)	1.26	0.11	15	1.07	0.14	5	1.7%	0.19 [0.06, 0.32]
Ramezani 2017(2)	1.21	0.14	15	1.07	0.14	5	1.6%	0.14 [-0.00, 0.28]
Ramezani 2017(3)	1.19	0.14	15	1.07	0.14	5	1.6%	0.12 [-0.02, 0.26]
Salus(2) 2022	1.07	0.19	14	0.96	0.11	14	1.9%	0.11 [-0.01, 0.23]
Savoye 2007	1.17	0.15	75	1.15	0.45	44	1.6%	0.02 [-0.12, 0.16]
Savoye 2011	1.19	0.18	75	1.12	0.41	44	1.7%	0.07 [-0.06, 0.20]
Saygın 2011	1.36	0.21	20	1.08	0.12	19	1.9%	0.28 [0.17, 0.39]
Seo 2012	0.72	1.15	10	0.95	1.15	10	0.1%	-0.23 [-1.24, 0.78]
Seo 2019	1.25	0.24	26	1.22	0.26	44	1.8%	0.03 [-0.09, 0.15]
Shalitin 2009	1.29	0.27	55	1.24	0.3	55	1.9%	0.05 [-0.06, 0.16]
Tas 2023	1.04	0.23	31	1.14	0.23	6	1.2%	-0.10 [-0.30, 0.10]
Vasconcellos 2016	1.15	0.13	10	1	0.44	10	0.7%	0.15 [-0.13, 0.43]
Wen 2008	1.31	0.18	24	0.95	0.27	13	1.4%	0.36 [0.20, 0.52]
Wong 2008	1.3	0.3	12	1.2	0.2	12	1.1%	0.10 [-0.10, 0.30]
Xinhui 2016	1.21	0.19	14	1.23	0.19	14	1.6%	-0.02 [-0.16, 0.12]
Yu 2020	1.5	0.3	99	1.3	0.2	72	2.2%	0.20 [0.12, 0.28]
Zehsaz 2016	1.17	0.31	16	1.16	0.26	16	1.2%	0.01 [-0.19, 0.21]
**Subtotal (95% CI)**			**1368**			**1035**	**82.1%**	**0.09 [0.05, 0.12]**
Heterogeneity: Tau^2 = 0.01; Chi^2 = 203.57, df = 53 (P < 0.00001); I^2 = 74%								
Test for overall effect: Z = 5.18 (P < 0.00001)								
**Total (95% CI)**			**1690**			**1263**	**100.0%**	**0.07 [0.05, 0.10]**
Heterogeneity: Tau^2 = 0.01; Chi^2 = 238.21, df = 66 (P < 0.00001); I^2 = 72%								
Test for overall effect: Z = 5.08 (P < 0.00001)								
Test for subgroup differences: Chi^2 = 2.47, df = 1 (P = 0.12), I^2 = 59.5%								

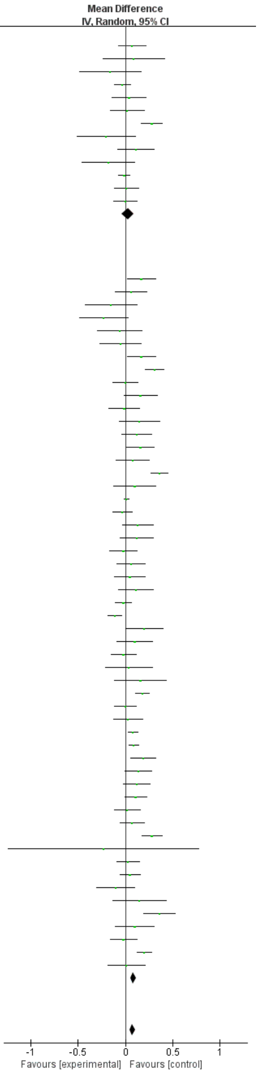

Figure S13.10. Forest plot of subgroup analyses by sex

Study or Subgroup	Experimental Mean	Experimental SD	Experimental Total	Control Mean	Control SD	Control Total	Weight	Mean Difference IV, Random, 95% CI
**2.10.1 Boys**								
Chuensiri 2018(1)	1.19	0.26	11	1.34	0.26	5	1.7%	-0.15 [-0.42, 0.12]
Chuensiri 2018(2)	1.11	0.3	15	1.34	0.26	6	1.8%	-0.23 [-0.49, 0.03]
Ghorbanian 2013	1.17	0.19	15	1.15	0.16	15	3.6%	0.02 [-0.11, 0.15]
Hongling 2012	1.2	0.2	30	1.2	0.3	30	3.5%	0.00 [-0.13, 0.13]
Jianjun 2012	1.3	0.3	20	1.18	0.18	15	3.0%	0.12 [-0.04, 0.28]
Karacabey 2009	1.53	0.19	20	1.17	0.09	20	4.1%	0.36 [0.27, 0.45]
Kim 2007	1.12	0.14	14	1.15	0.13	12	3.9%	-0.03 [-0.13, 0.07]
Lingling 2021	1.07	0.16	20	0.97	0.22	20	3.7%	0.10 [-0.02, 0.22]
Luo 2013(2)	1.09	0.18	52	1.2	0.16	38	4.5%	-0.11 [-0.18, -0.04]
Meng 2022(1)	1.5	0.2	12	1.3	0.2	6	2.5%	0.20 [0.00, 0.40]
Meng 2022(2)	1.4	0.2	11	1.3	0.2	7	2.6%	0.10 [-0.09, 0.29]
Ramezani 2017(1)	1.26	0.11	15	1.07	0.14	5	3.4%	0.19 [0.06, 0.32]
Ramezani 2017(2)	1.21	0.14	15	1.07	0.14	5	3.3%	0.14 [-0.00, 0.28]
Ramezani 2017(3)	1.19	0.14	15	1.07	0.14	5	3.3%	0.12 [-0.02, 0.26]
Salus(2) 2022	1.07	0.19	14	0.96	0.11	14	3.7%	0.11 [-0.01, 0.23]
Seo 2012	0.72	1.15	10	0.95	1.15	10	0.2%	-0.23 [-1.24, 0.78]
Wong 2008	1.3	0.3	12	1.2	0.2	12	2.4%	0.10 [-0.10, 0.30]
Zehsaz 2016	1.17	0.31	16	1.16	0.26	16	2.5%	0.01 [-0.19, 0.21]
Zehsaz 2017	1.17	0.31	16	1.16	0.26	16	2.5%	0.01 [-0.19, 0.21]
**Subtotal (95% CI)**			**333**			**257**	**56.2%**	**0.07 [-0.01, 0.14]**
Heterogeneity: Tau^2 = 0.02; Chi^2 = 84.39, df = 18 (P < 0.00001); I^2 = 79%								
Test for overall effect: Z = 1.80 (P = 0.07)								
**2.10.2 Girls**								
DeBar 2012	1.12	0.22	74	1.15	0.22	42	4.3%	-0.03 [-0.11, 0.05]
Jian 2014	1.59	0.45	44	1.44	0.55	44	2.3%	0.15 [-0.06, 0.36]
Kim 2022	2.26	1.08	13	1.82	1	13	0.3%	0.44 [-0.36, 1.24]
Liu 2018	1.21	0.18	30	1.1	0.4	20	2.6%	0.11 [-0.08, 0.30]
Lopes 2016	1.37	0.32	17	1.55	0.47	16	1.7%	-0.18 [-0.46, 0.10]
Luo 2013(1)	1.15	0.21	43	1.17	0.17	34	4.2%	-0.02 [-0.10, 0.06]
Mohammadi 2019	1.1	0.09	10	1.11	0.04	10	4.6%	-0.01 [-0.07, 0.05]
Park 2007	1.26	0.16	19	1.26	0.2	21	3.8%	0.00 [-0.11, 0.11]
Plavsic 2020	1.33	0.27	22	1.3	0.26	22	3.1%	0.03 [-0.13, 0.19]
Racil 2013(1)	1.08	0.08	11	1	0.03	6	4.7%	0.08 [0.03, 0.13]
Racil 2013(2)	1.09	0.07	11	1	0.03	6	4.8%	0.09 [0.04, 0.14]
Saygın 2011	1.36	0.21	20	1.08	0.12	19	3.9%	0.28 [0.17, 0.39]
Staiano 2017	1.26	0.18	20	1.33	0.22	18	3.5%	-0.07 [-0.20, 0.06]
**Subtotal (95% CI)**			**334**			**271**	**43.8%**	**0.04 [-0.01, 0.10]**
Heterogeneity: Tau^2 = 0.01; Chi^2 = 40.66, df = 12 (P < 0.0001); I^2 = 70%								
Test for overall effect: Z = 1.65 (P = 0.10)								
**Total (95% CI)**			**667**			**528**	**100.0%**	**0.06 [0.02, 0.10]**
Heterogeneity: Tau^2 = 0.01; Chi^2 = 125.60, df = 31 (P < 0.00001); I^2 = 75%								
Test for overall effect: Z = 2.64 (P = 0.008)								
Test for subgroup differences: Chi^2 = 0.24, df = 1 (P = 0.63), I^2 = 0%								

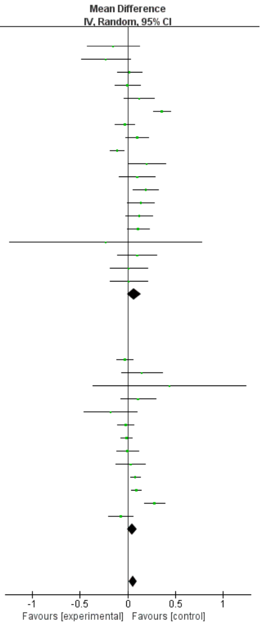

Figure S14. Forest plots of subgroup analyses for LDL-C in children and adolescents with overweight or obesity

Figure S14.1. Forest plot of subgroup analyses by type of exercise

Study or Subgroup	Experimental Mean	Experimental SD	Experimental Total	Control Mean	Control SD	Control Total	Weight	Mean Difference IV, Random, 95% CI
**2.1.1 Aerobic exercise**								
Ahmadi 2020(1)	2.37	0.45	28	2.3	0.72	9	1.0%	0.07 [-0.43, 0.57]
Bezerra 2023	2.45	0.41	20	2.82	0.62	21	1.3%	-0.37 [-0.69, -0.05]
Boer 2014(2)	2.52	0.74	15	2.48	0.55	7	0.9%	0.04 [-0.51, 0.59]
Boff 2020	2.25	0.74	34	2.08	0.75	31	1.2%	0.17 [-0.19, 0.53]
Bowen 2010	3	0.68	22	3.29	0.59	18	1.2%	-0.29 [-0.68, 0.10]
Davis 2020	2.44	0.86	90	2.57	0.8	85	1.5%	-0.13 [-0.38, 0.12]
DeBar 2012	2.48	0.69	74	2.45	0.53	42	1.5%	0.03 [-0.19, 0.25]
Dias 2018(2)	2.55	0.84	22	2.36	0.7	10	0.9%	0.19 [-0.37, 0.75]
Ghorbanian 2013	3.12	0.57	15	3.88	0.72	15	1.0%	-0.76 [-1.22, -0.30]
Guijun 2009	2.35	0.06	16	3.29	0.25	10	1.6%	-0.94 [-1.10, -0.78]
Gulijianati 2022	2.17	0.29	47	2.98	0.35	46	1.7%	-0.81 [-0.94, -0.68]
Guo 2015	2.74	0.48	23	3.02	0.87	12	0.9%	-0.28 [-0.81, 0.25]
Horner 2015(1)	2.2	0.71	27	2.27	0.34	8	1.2%	-0.07 [-0.43, 0.29]
Jian 2014	2.33	0.21	44	2.63	0.24	44	1.7%	-0.30 [-0.39, -0.21]
Jianjun 2012	1.71	0.48	20	2.92	0.67	15	1.1%	-1.21 [-1.61, -0.81]
Jun 2006(1)	1.91	0.41	33	2.14	0.64	31	1.4%	-0.23 [-0.50, 0.04]
Jun 2006(2)	1.8	0.43	35	2.13	1.04	32	1.2%	-0.33 [-0.72, 0.06]
Karacabey 2009	1.75	0.24	20	2.32	0.28	20	1.6%	-0.57 [-0.73, -0.41]
Kelishadi 2008	3.1	0.36	45	3.16	0.33	47	1.7%	-0.06 [-0.20, 0.08]
Kelly 2004	2.36	0.76	10	2.32	0.73	10	0.7%	0.04 [-0.61, 0.69]
Kim 2007	2.54	0.74	14	2.96	0.85	12	0.8%	-0.42 [-1.04, 0.20]
Lee 2010(2)	2.68	0.71	16	2.63	0.61	9	0.9%	0.05 [-0.48, 0.58]
Liqun 2014	2.39	0.38	76	2.49	0.4	97	1.7%	-0.10 [-0.22, 0.02]
Lira 2017(1)	2.47	0.93	26	2.89	0.69	16	1.0%	-0.42 [-0.91, 0.07]
Lira 2017(2)	2.48	0.72	25	2.89	0.69	17	1.1%	-0.41 [-0.84, 0.02]
Liu 2018	1.98	0.52	30	2.67	0.59	20	1.3%	-0.69 [-1.01, -0.37]
Luo 2013(1)	1.93	0.46	43	2.54	0.33	34	1.6%	-0.61 [-0.79, -0.43]
Luo 2013(2)	1.97	0.38	52	2.63	0.33	38	1.7%	-0.66 [-0.81, -0.51]
Machado 2022	2.15	0.6	20	2.13	0.56	15	1.2%	0.02 [-0.37, 0.41]
Meng 2022(2)	2	0.2	11	2.2	0.5	7	1.2%	-0.20 [-0.59, 0.19]
Meyer 2006	2.57	0.66	33	3.01	0.79	34	1.3%	-0.44 [-0.79, -0.09]
Monteiro 2015(1)	1.3	0.72	18	1.79	0.71	8	0.8%	-0.49 [-1.08, 0.10]
Morgan 2021	2.37	0.24	15	3.12	0.33	15	1.6%	-0.75 [-0.96, -0.54]
Murphy 2009	2.74	0.72	23	2.74	0.43	12	1.2%	0.00 [-0.38, 0.38]
Park 2007	2.35	0.49	19	2.54	0.66	21	1.2%	-0.19 [-0.55, 0.17]
Plavsic 2020	2.28	0.69	22	2.18	0.55	22	1.2%	0.10 [-0.27, 0.47]
Racil 2013(2)	2.55	0.32	11	2.62	0.18	6	1.5%	-0.07 [-0.31, 0.17]
Ramezani 2017(1)	1.89	1.02	15	3.8	1.17	5	0.3%	-1.91 [-3.06, -0.76]
Savoye 2007	2.52	0.92	75	2.52	0.68	44	1.4%	0.00 [-0.29, 0.29]
Savoye 2011	2.54	0.75	75	2.53	0.65	44	1.5%	0.01 [-0.25, 0.27]
Saygın 2011	2.4	0.37	20	2.51	0.28	19	1.6%	-0.11 [-0.32, 0.10]
Seo 2012	2.7	2	10	2.34	1.23	10	0.2%	0.36 [-1.10, 1.82]
Seo 2019	2.69	0.64	26	2.9	0.58	44	1.4%	-0.21 [-0.51, 0.09]
Staiano 2017	2.34	0.4	20	2.46	0.57	18	1.3%	-0.12 [-0.44, 0.20]
Staiano 2018	2.19	0.8	22	2.76	0.74	23	1.0%	-0.57 [-1.02, -0.12]
Sun 2011	2.3	0.5	25	2.4	0.5	17	1.3%	-0.10 [-0.41, 0.21]
Tsang 2009	2.62	0.75	11	2.33	0.57	8	0.8%	0.29 [-0.30, 0.88]
Woo 2004	2.6	0.8	41	2.7	0.7	41	1.3%	-0.10 [-0.43, 0.23]
Xianbo 2011	2.3	0.5	25	2.4	0.5	17	1.3%	-0.10 [-0.41, 0.21]
Xinhui 2016	2.21	0.61	14	2.68	0.75	14	0.9%	-0.47 [-0.98, 0.04]
**Subtotal (95% CI)**			**1473**			**1200**	**60.9%**	**-0.28 [-0.38, -0.19]**
Heterogeneity: Tau^2 = 0.09; Chi^2 = 307.36, df = 49 (P < 0.00001); I^2 = 84%								
Test for overall effect: Z = 5.66 (P < 0.00001)								
**2.1.2 Anaerobic exercise**								
Boer 2014(1)	2.47	0.24	17	2.48	0.55	7	1.1%	-0.01 [-0.43, 0.41]
Chuensiri 2018(1)	2.28	0.51	11	2.69	0.51	5	0.9%	-0.41 [-0.95, 0.13]
Chuensiri 2018(2)	2.09	0.5	15	2.69	0.51	6	1.0%	-0.60 [-1.08, -0.12]
Dias 2018(1)	2.1	0.5	19	2.36	0.7	9	0.9%	-0.26 [-0.77, 0.25]
Ke 2024	2.6	0.4	10	3.4	0.7	10	1.0%	-0.80 [-1.30, -0.30]
Lingling 2021	4	1.05	20	4.38	1	20	0.7%	-0.38 [-1.02, 0.26]
Meng 2022(1)	1.9	0.3	12	2.2	0.5	6	1.1%	-0.30 [-0.73, 0.13]
Racil 2013(1)	2.18	0.4	11	2.62	0.18	6	1.4%	-0.44 [-0.72, -0.16]
Salus(2) 2022	2.05	0.37	14	2.6	0.37	14	1.4%	-0.55 [-0.82, -0.28]
Tas 2023	2.37	0.55	31	2.41	0.55	6	1.0%	-0.04 [-0.52, 0.44]
Vasconcellos 2016	2.61	0.33	10	3.03	0.82	10	0.9%	-0.42 [-0.97, 0.13]
**Subtotal (95% CI)**			**170**			**99**	**11.4%**	**-0.40 [-0.53, -0.27]**
Heterogeneity: Tau^2 = 0.00; Chi^2 = 10.28, df = 10 (P = 0.42); I^2 = 3%								
Test for overall effect: Z = 6.14 (P < 0.00001)								
**2.1.3 Resistance exercise**								
Ahmadi 2020(2)	2.1	0.52	29	2.3	0.72	9	0.9%	-0.20 [-0.71, 0.31]
Benson 2008	2.3	0.9	32	2.4	0.6	35	1.2%	-0.10 [-0.47, 0.27]
Horner 2015(2)	1.86	0.43	23	2.27	0.34	8	1.4%	-0.41 [-0.70, -0.12]
Ramezani 2017(2)	2.28	0.93	15	3.8	1.17	5	0.3%	-1.52 [-2.65, -0.39]
**Subtotal (95% CI)**			**99**			**57**	**3.8%**	**-0.35 [-0.68, -0.01]**
Heterogeneity: Tau^2 = 0.06; Chi^2 = 6.28, df = 3 (P = 0.10); I^2 = 52%								
Test for overall effect: Z = 2.02 (P = 0.04)								
**2.1.4 Combined aerobic and resistance exercise**								
Ahmadi 2020(3)	2.49	0.68	29	2.3	0.72	9	0.9%	0.19 [-0.34, 0.72]
Bruyndonckx 2015	1.84	0.33	27	2.24	0.36	21	1.6%	-0.40 [-0.60, -0.20]
Chae 2010	2.33	0.73	19	2.54	0.57	19	1.1%	-0.21 [-0.63, 0.21]
Elmahgoub 2009	2.26	0.72	15	2.41	0.54	15	1.0%	-0.15 [-0.61, 0.31]
Faria 2020(1)	1.85	0.49	24	2.07	0.5	13	1.3%	-0.22 [-0.56, 0.12]
Faria 2020(2)	2.03	0.5	26	2.07	0.5	13	1.3%	-0.04 [-0.37, 0.29]
Farpour-Lambert 2009	2.72	0.76	22	2.69	0.55	22	1.2%	0.03 [-0.36, 0.42]
Harder-Lauridsen 2014	2.3	0.75	14	2.6	0.25	14	1.1%	-0.30 [-0.71, 0.11]
Hongling 2012	2.6	0.7	30	2.6	1	30	1.1%	0.00 [-0.44, 0.44]
Kim 2022	2.23	0.71	13	1.85	0.56	13	1.0%	0.38 [-0.11, 0.87]
Lee 2010(1)	2.73	0.42	20	2.63	0.61	9	1.1%	0.10 [-0.34, 0.54]
Liangmei 2015	2.21	0.59	12	2.68	0.74	12	0.9%	-0.47 [-1.01, 0.07]
Lopes 2016	2.41	0.8	17	2.39	0.96	16	0.8%	0.02 [-0.58, 0.62]
Migueles 2023	2.34	0.39	38	2.52	0.39	33	1.6%	-0.18 [-0.36, 0.00]
Mohammadi 2019	2.52	0.32	10	2.57	0.34	10	1.4%	-0.05 [-0.34, 0.24]
Monteiro 2015(2)	1.05	0.54	14	1.79	0.71	8	0.8%	-0.74 [-1.31, -0.17]
Ramezani 2017(3)	2.13	1.09	15	3.8	1.17	5	0.3%	-1.67 [-2.83, -0.51]
Shalitin 2009	2.55	0.67	55	2.53	0.73	55	1.4%	0.02 [-0.24, 0.28]
Wen 2008	2.21	0.47	24	3.29	0.87	13	0.9%	-1.08 [-1.59, -0.57]
Wong 2008	2.7	0.9	12	2.8	0.7	12	0.7%	-0.10 [-0.75, 0.55]
Zehsaz 2016	1.87	0.25	16	2.24	0.19	16	1.7%	-0.37 [-0.52, -0.22]
Zehsaz 2017	2.54	0.92	16	2.47	0.87	16	0.8%	0.07 [-0.55, 0.69]
**Subtotal (95% CI)**			**468**			**374**	**23.9%**	**-0.19 [-0.31, -0.07]**
Heterogeneity: Tau^2 = 0.04; Chi^2 = 48.07, df = 21 (P = 0.0007); I^2 = 56%								
Test for overall effect: Z = 3.06 (P = 0.002)								
**Total (95% CI)**			**2210**			**1730**	**100.0%**	**-0.28 [-0.35, -0.20]**
Heterogeneity: Tau^2 = 0.07; Chi^2 = 383.87, df = 86 (P < 0.00001); I^2 = 78%								
Test for overall effect: Z = 7.58 (P < 0.00001)								
Test for subgroup differences: Chi^2 = 5.87, df = 3 (P = 0.12), I^2 = 48.9%								

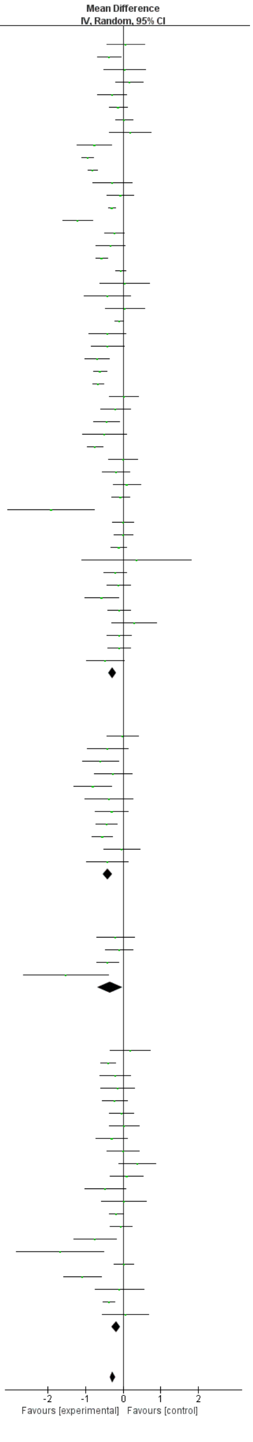

Figure S14.2. Forest plot of subgroup analyses by exercise intensity

Study or Subgroup	Experimental Mean	Experimental SD	Experimental Total	Control Mean	Control SD	Control Total	Weight	Mean Difference IV, Random, 95% CI
**2.2.1 Moderate-to-low intensity**								
Boer 2014(2)	2.52	0.74	15	2.48	0.55	7	0.9%	0.04 [-0.51, 0.59]
Boff 2020	2.25	0.74	34	2.08	0.75	31	1.2%	0.17 [-0.19, 0.53]
Bowen 2010	3	0.68	22	3.29	0.59	18	1.2%	-0.29 [-0.68, 0.10]
Bruyndonckx 2015	1.84	0.33	27	2.24	0.36	21	1.6%	-0.40 [-0.60, -0.20]
Davis 2020	2.44	0.86	90	2.57	0.8	85	1.5%	-0.13 [-0.38, 0.12]
DeBar 2012	2.48	0.69	74	2.45	0.53	42	1.5%	0.03 [-0.19, 0.25]
Dias 2018(2)	2.55	0.84	22	2.36	0.7	10	0.9%	0.19 [-0.37, 0.75]
Elmahgoub 2009	2.26	0.72	15	2.41	0.54	15	1.0%	-0.15 [-0.61, 0.31]
Faria 2020(1)	1.85	0.49	24	2.07	0.5	13	1.3%	-0.22 [-0.56, 0.12]
Farpour-Lambert 2009	2.72	0.76	22	2.69	0.55	22	1.2%	0.03 [-0.36, 0.42]
Ghorbanian 2013	3.12	0.57	15	3.88	0.72	15	1.0%	-0.76 [-1.22, -0.30]
Guijun 2009	2.35	0.06	16	3.29	0.25	10	1.6%	-0.94 [-1.10, -0.78]
Guo 2015	2.74	0.48	23	3.02	0.87	12	0.9%	-0.28 [-0.81, 0.25]
Harder-Lauridsen 2014	2.3	0.75	14	2.6	0.25	14	1.1%	-0.30 [-0.71, 0.11]
Horner 2015(1)	2.2	0.71	27	2.27	0.34	8	1.2%	-0.07 [-0.43, 0.29]
Jian 2014	2.33	0.21	44	2.63	0.24	44	1.7%	-0.30 [-0.39, -0.21]
Jianjun 2012	1.71	0.48	20	2.92	0.67	15	1.1%	-1.21 [-1.61, -0.81]
Jun 2006(1)	1.91	0.41	33	2.14	0.64	31	1.4%	-0.23 [-0.50, 0.04]
Jun 2006(2)	1.8	0.43	35	2.13	1.04	32	1.2%	-0.33 [-0.72, 0.06]
Karacabey 2009	1.75	0.24	20	2.32	0.28	20	1.6%	-0.57 [-0.73, -0.41]
Kelishadi 2008	3.1	0.36	45	3.16	0.33	47	1.7%	-0.06 [-0.20, 0.08]
Kelly 2004	2.36	0.76	10	2.32	0.73	10	0.7%	0.04 [-0.61, 0.69]
Kim 2007	2.54	0.74	14	2.96	0.85	12	0.8%	-0.42 [-1.04, 0.20]
Kim 2022	2.23	0.71	13	1.85	0.56	13	1.0%	0.38 [-0.11, 0.87]
Lee 2010(1)	2.73	0.42	20	2.63	0.61	9	1.1%	0.10 [-0.34, 0.54]
Lee 2010(2)	2.68	0.71	16	2.63	0.61	9	0.9%	0.05 [-0.48, 0.58]
Liangmei 2015	2.21	0.59	12	2.68	0.74	12	0.9%	-0.47 [-1.01, 0.07]
Liqun 2014	2.39	0.38	76	2.49	0.4	97	1.7%	-0.10 [-0.22, 0.02]
Lira 2017(2)	2.48	0.72	25	2.89	0.69	17	1.1%	-0.41 [-0.84, 0.02]
Liu 2018	1.98	0.52	30	2.67	0.59	20	1.3%	-0.69 [-1.01, -0.37]
Luo 2013(1)	1.93	0.46	43	2.54	0.33	34	1.6%	-0.61 [-0.79, -0.43]
Luo 2013(2)	1.97	0.38	52	2.63	0.33	38	1.7%	-0.66 [-0.81, -0.51]
Machado 2022	2.15	0.6	20	2.13	0.56	15	1.2%	0.02 [-0.37, 0.41]
Meng 2022(2)	2	0.2	11	2.2	0.5	7	1.2%	-0.20 [-0.59, 0.19]
Meyer 2006	2.57	0.66	33	3.01	0.79	34	1.3%	-0.44 [-0.79, -0.09]
Mohammadi 2019	2.52	0.32	10	2.57	0.34	10	1.4%	-0.05 [-0.34, 0.24]
Morgan 2021	2.37	0.24	15	3.12	0.33	15	1.6%	-0.75 [-0.96, -0.54]
Murphy 2009	2.74	0.72	23	2.74	0.43	12	1.2%	0.00 [-0.38, 0.38]
Park 2007	2.35	0.49	19	2.54	0.66	21	1.2%	-0.19 [-0.55, 0.17]
Racil 2013(2)	2.55	0.32	11	2.62	0.18	6	1.5%	-0.07 [-0.31, 0.17]
Ramezani 2017(1)	1.89	1.02	15	3.8	1.17	5	0.3%	-1.91 [-3.06, -0.76]
Ramezani 2017(2)	2.28	0.93	15	3.8	1.17	5	0.3%	-1.52 [-2.65, -0.39]
Ramezani 2017(3)	2.13	1.09	15	3.8	1.17	5	0.3%	-1.67 [-2.83, -0.51]
Saygın 2011	2.4	0.37	20	2.51	0.28	19	1.6%	-0.11 [-0.32, 0.10]
Seo 2012	2.7	2	10	2.34	1.23	10	0.2%	0.36 [-1.10, 1.82]
Seo 2019	2.69	0.64	26	2.9	0.58	44	1.4%	-0.21 [-0.51, 0.09]
Shalitin 2009	2.55	0.67	55	2.53	0.73	55	1.4%	0.02 [-0.24, 0.28]
Staiano 2017	2.34	0.4	20	2.46	0.57	18	1.3%	-0.12 [-0.44, 0.20]
Staiano 2018	2.19	0.8	22	2.76	0.74	23	1.0%	-0.57 [-1.02, -0.12]
Sun 2011	2.3	0.5	25	2.4	0.5	17	1.3%	-0.10 [-0.41, 0.21]
Tsang 2009	2.62	0.75	11	2.33	0.57	8	0.8%	0.29 [-0.30, 0.88]
Wen 2008	2.21	0.47	24	3.29	0.87	13	0.9%	-1.08 [-1.59, -0.57]
Woo 2004	2.6	0.8	41	2.7	0.7	41	1.3%	-0.10 [-0.43, 0.23]
Xianbo 2011	2.3	0.5	25	2.4	0.5	17	1.3%	-0.10 [-0.41, 0.21]
Xinhui 2016	2.21	0.61	14	2.68	0.75	14	0.9%	-0.47 [-0.98, 0.04]
Zehsaz 2016	1.87	0.25	16	2.24	0.19	16	1.7%	-0.37 [-0.52, -0.22]
**Subtotal (95% CI)**			**1444**			**1213**	**66.3%**	**-0.28 [-0.37, -0.20]**
Heterogeneity: Tau^2 = 0.08; Chi^2 = 280.97, df = 55 (P < 0.00001); I^2 = 80%								
Test for overall effect: Z = 6.32 (P < 0.00001)								
**2.2.2 High intensity**								
Ahmadi 2020(1)	2.37	0.45	28	2.3	0.72	9	1.0%	0.07 [-0.43, 0.57]
Ahmadi 2020(2)	2.1	0.52	29	2.3	0.72	9	0.9%	-0.20 [-0.71, 0.31]
Ahmadi 2020(3)	2.49	0.68	29	2.3	0.72	9	0.9%	0.19 [-0.34, 0.72]
Benson 2008	2.3	0.9	32	2.4	0.6	35	1.2%	-0.10 [-0.47, 0.27]
Bezerra 2023	2.45	0.41	20	2.82	0.62	21	1.3%	-0.37 [-0.69, -0.05]
Boer 2014(1)	2.47	0.24	17	2.48	0.55	7	1.1%	-0.01 [-0.43, 0.41]
Chae 2010	2.33	0.73	19	2.54	0.57	19	1.1%	-0.21 [-0.63, 0.21]
Chuensiri 2018(1)	2.28	0.51	11	2.69	0.51	5	0.9%	-0.41 [-0.95, 0.13]
Chuensiri 2018(2)	2.09	0.5	15	2.69	0.51	6	1.0%	-0.60 [-1.08, -0.12]
Dias 2018(1)	2.1	0.5	19	2.36	0.7	9	0.9%	-0.26 [-0.77, 0.25]
Faria 2020(2)	2.03	0.5	26	2.07	0.5	13	1.3%	-0.04 [-0.37, 0.29]
Gulijianati 2022	2.17	0.29	47	2.98	0.35	46	1.7%	-0.81 [-0.94, -0.68]
Hongling 2012	2.6	0.7	30	2.6	1	30	1.1%	0.00 [-0.44, 0.44]
Horner 2015(2)	1.86	0.43	23	2.27	0.34	8	1.4%	-0.41 [-0.70, -0.12]
Ke 2024	2.6	0.4	10	3.4	0.7	10	1.0%	-0.80 [-1.30, -0.30]
Lingling 2021	4	1.05	20	4.38	1	20	0.7%	-0.38 [-1.02, 0.26]
Lira 2017(1)	2.47	0.93	26	2.89	0.69	16	1.0%	-0.42 [-0.91, 0.07]
Lopes 2016	2.41	0.8	17	2.39	0.96	16	0.8%	0.02 [-0.58, 0.62]
Meng 2022(1)	1.9	0.3	12	2.2	0.5	6	1.1%	-0.30 [-0.73, 0.13]
Migueles 2023	2.34	0.39	38	2.52	0.39	33	1.6%	-0.18 [-0.36, 0.00]
Monteiro 2015(1)	1.3	0.72	18	1.79	0.71	8	0.8%	-0.49 [-1.08, 0.10]
Monteiro 2015(2)	1.05	0.54	14	1.79	0.71	8	0.8%	-0.74 [-1.31, -0.17]
Plavsic 2020	2.28	0.69	22	2.18	0.55	22	1.2%	0.10 [-0.27, 0.47]
Racil 2013(1)	2.18	0.4	11	2.62	0.18	6	1.4%	-0.44 [-0.72, -0.16]
Salus(2) 2022	2.05	0.37	14	2.6	0.37	14	1.4%	-0.55 [-0.82, -0.28]
Savoye 2007	2.52	0.92	75	2.52	0.68	44	1.4%	0.00 [-0.29, 0.29]
Savoye 2011	2.54	0.75	75	2.53	0.65	44	1.5%	0.01 [-0.25, 0.27]
Tas 2023	2.37	0.55	31	2.41	0.55	6	1.0%	-0.04 [-0.52, 0.44]
Vasconcellos 2016	2.61	0.33	10	3.03	0.82	10	0.9%	-0.42 [-0.97, 0.13]
Wong 2008	2.7	0.9	12	2.8	0.7	12	0.7%	-0.10 [-0.75, 0.55]
Zehsaz 2017	2.54	0.92	16	2.47	0.87	16	0.8%	0.07 [-0.55, 0.69]
**Subtotal (95% CI)**			**766**			**517**	**33.7%**	**-0.26 [-0.38, -0.13]**
Heterogeneity: Tau^2 = 0.08; Chi^2 = 101.83, df = 30 (P < 0.00001); I^2 = 71%								
Test for overall effect: Z = 4.07 (P < 0.0001)								
**Total (95% CI)**			**2210**			**1730**	**100.0%**	**-0.28 [-0.35, -0.20]**
Heterogeneity: Tau^2 = 0.07; Chi^2 = 383.87, df = 86 (P < 0.00001); I^2 = 78%								
Test for overall effect: Z = 7.58 (P < 0.00001)								
Test for subgroup differences: Chi^2 = 0.11, df = 1 (P = 0.74), I^2 = 0%								

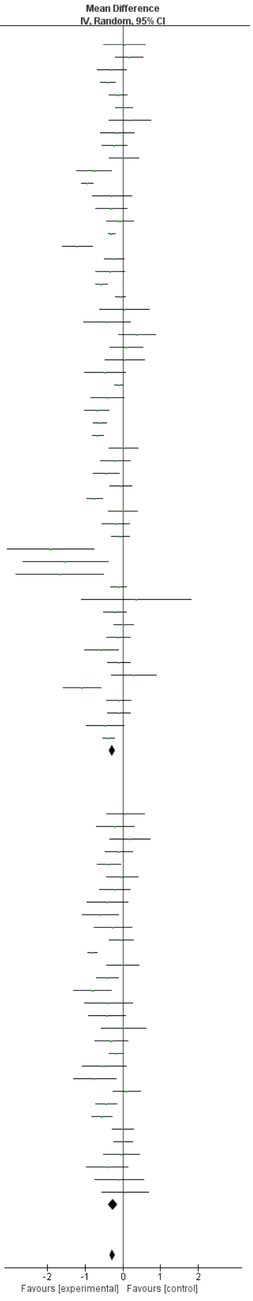

Figure S14.3. Forest plot of subgroup analyses by duration per session

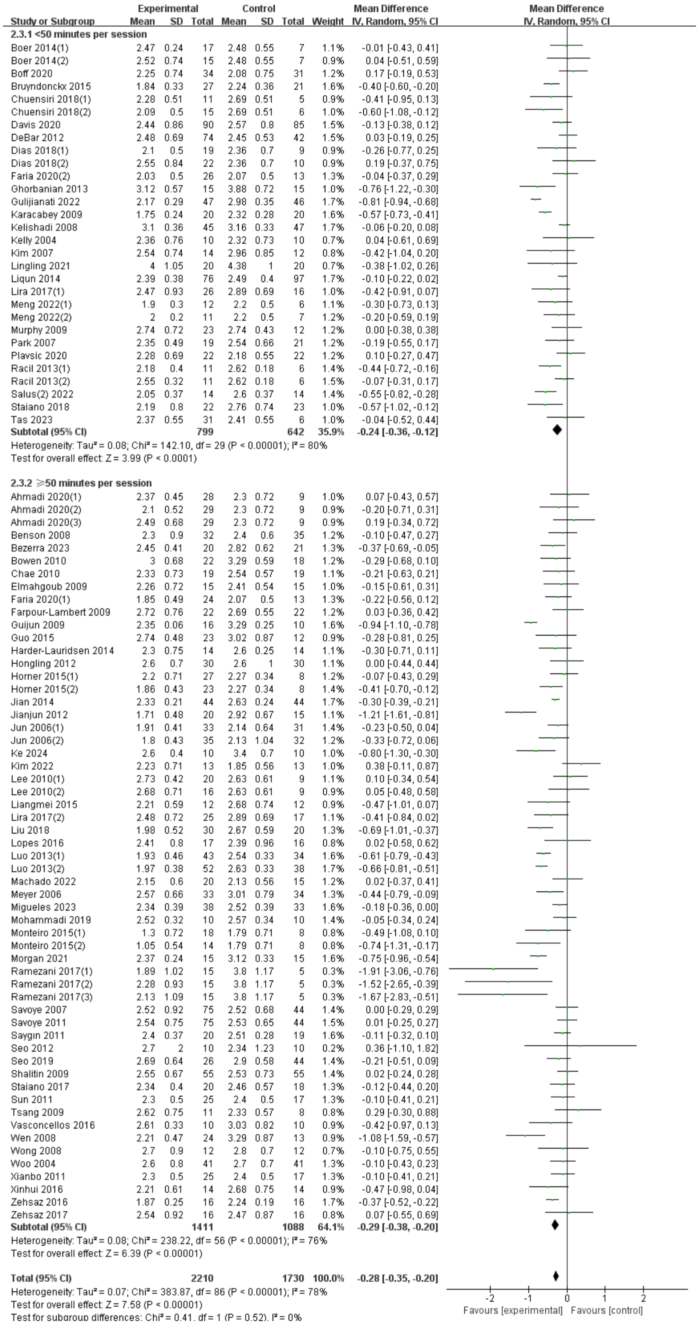

Figure S14.4. Forest plot of subgroup analyses by exercise frequency

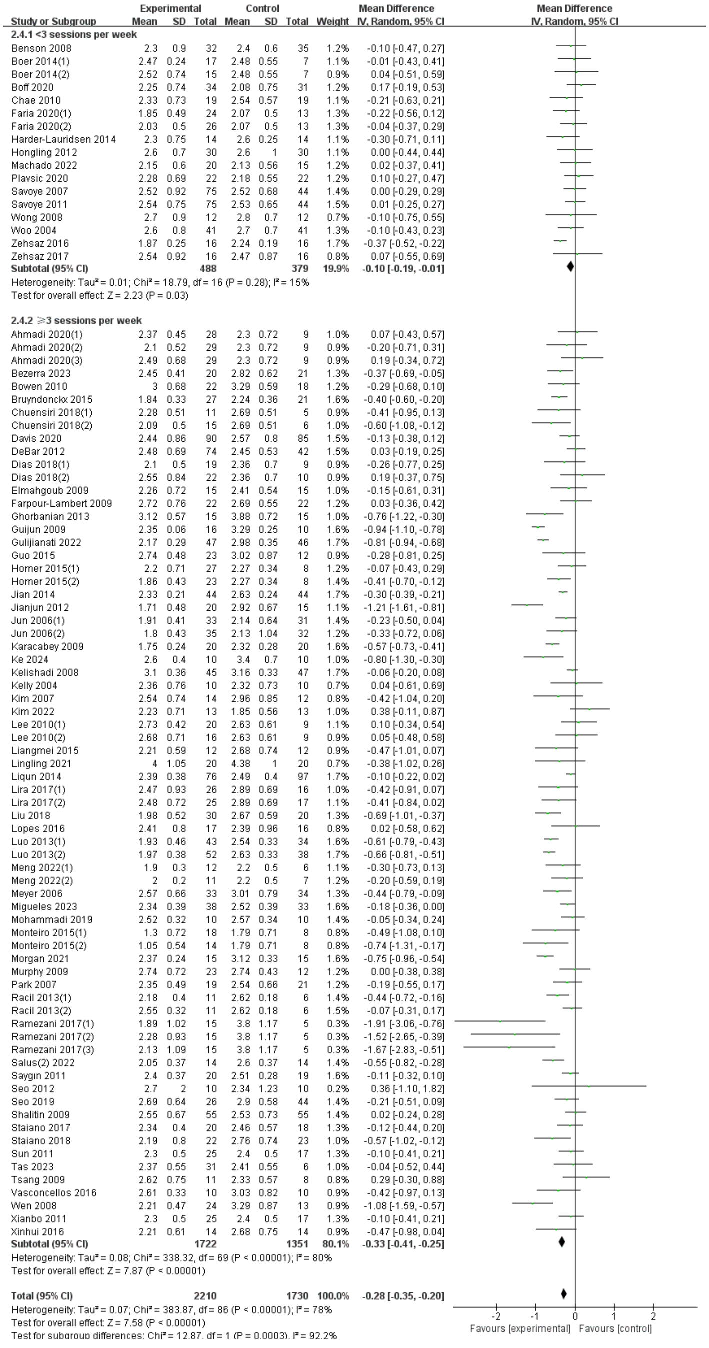

Figure S14.5. Forest plot of subgroup analyses by weekly exercise duration

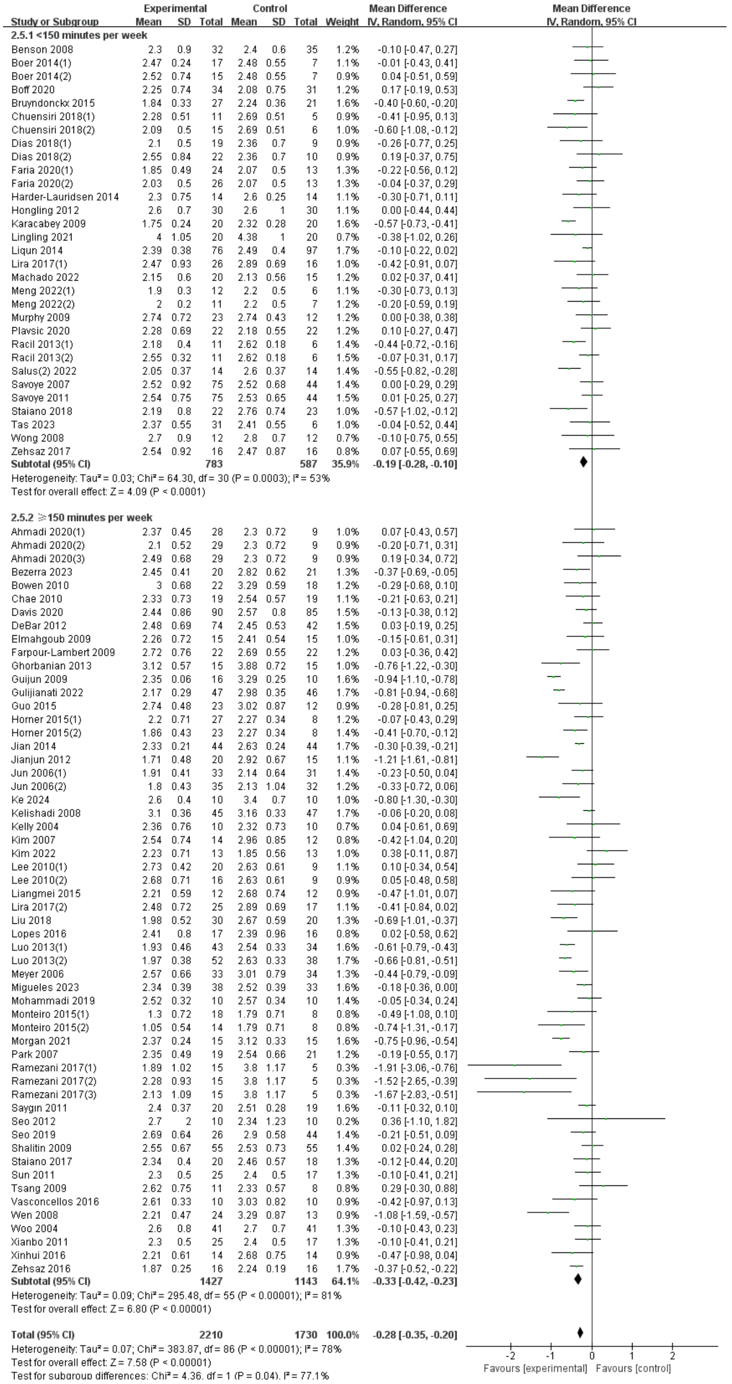

Figure S14.6. Forest plot of subgroup analyses by intervention duration

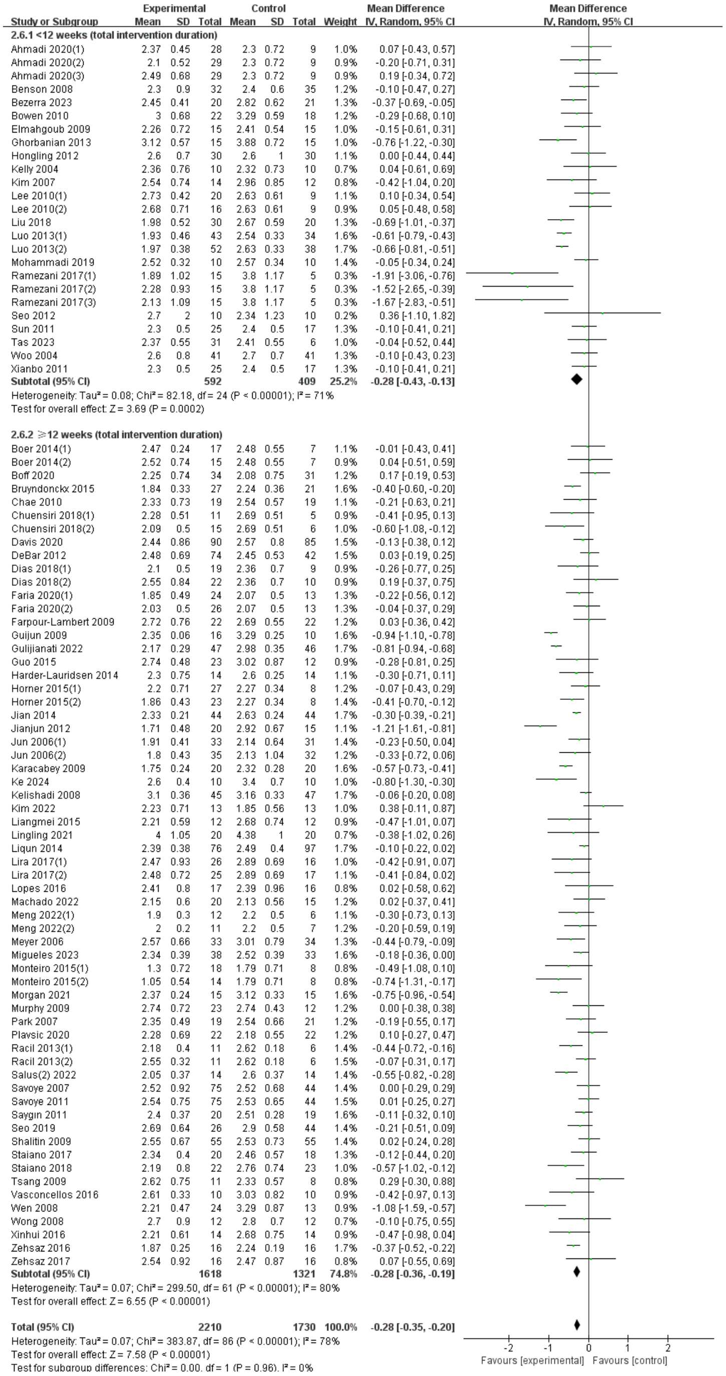

Figure S14.7. Forest plot of subgroup analyses by region

Study or Subgroup	Experimental Mean	Experimental SD	Experimental Total	Control Mean	Control SD	Control Total	Weight	Mean Difference IV, Random, 95% CI
**2.7.1 Asia**								
Ahmadi 2020(1)	2.37	0.45	28	2.3	0.72	9	1.0%	0.07 [-0.43, 0.57]
Ahmadi 2020(2)	2.1	0.52	29	2.3	0.72	9	0.9%	-0.20 [-0.71, 0.31]
Ahmadi 2020(3)	2.49	0.68	29	2.3	0.72	9	0.9%	0.19 [-0.34, 0.72]
Bowen 2010	3	0.68	22	3.29	0.59	18	1.2%	-0.29 [-0.68, 0.10]
Chae 2010	2.33	0.73	19	2.54	0.57	19	1.1%	-0.21 [-0.63, 0.21]
Chuensiri 2018(1)	2.28	0.51	11	2.69	0.51	5	0.9%	-0.41 [-0.95, 0.13]
Chuensiri 2018(2)	2.09	0.5	15	2.69	0.51	6	1.0%	-0.60 [-1.08, -0.12]
Ghorbanian 2013	3.12	0.57	15	3.88	0.72	15	1.0%	-0.76 [-1.22, -0.30]
Guijun 2009	2.35	0.06	16	3.29	0.25	10	1.6%	-0.94 [-1.10, -0.78]
Gulijianati 2022	2.17	0.29	47	2.98	0.35	46	1.7%	-0.81 [-0.94, -0.68]
Guo 2015	2.74	0.48	23	3.02	0.87	12	0.9%	-0.28 [-0.81, 0.25]
Hongling 2012	2.6	0.7	30	2.6	1	30	1.1%	0.00 [-0.44, 0.44]
Jian 2014	2.33	0.21	44	2.63	0.24	44	1.7%	-0.30 [-0.39, -0.21]
Jianjun 2012	1.71	0.48	20	2.92	0.67	15	1.1%	-1.21 [-1.61, -0.81]
Jun 2006(1)	1.91	0.41	33	2.14	0.64	31	1.4%	-0.23 [-0.50, 0.04]
Jun 2006(2)	1.8	0.43	35	2.13	1.04	32	1.2%	-0.33 [-0.72, 0.06]
Karacabey 2009	1.75	0.24	20	2.32	0.28	20	1.6%	-0.57 [-0.73, -0.41]
Ke 2024	2.6	0.4	10	3.4	0.7	10	1.0%	-0.80 [-1.30, -0.30]
Kelishadi 2008	3.1	0.36	45	3.16	0.33	47	1.7%	-0.06 [-0.20, 0.08]
Kim 2007	2.54	0.74	14	2.96	0.85	12	0.8%	-0.42 [-1.04, 0.20]
Kim 2022	2.23	0.71	13	1.85	0.56	13	1.0%	0.38 [-0.11, 0.87]
Lee 2010(1)	2.73	0.42	20	2.63	0.61	9	1.1%	0.10 [-0.34, 0.54]
Lee 2010(2)	2.68	0.71	16	2.63	0.61	9	0.9%	0.05 [-0.48, 0.58]
Liangmei 2015	2.21	0.59	12	2.68	0.74	12	0.9%	-0.47 [-1.01, 0.07]
Lingling 2021	4	1.05	20	4.38	1	20	0.7%	-0.38 [-1.02, 0.26]
Liqun 2014	2.39	0.38	76	2.49	0.4	97	1.7%	-0.10 [-0.22, 0.02]
Liu 2018	1.98	0.52	30	2.67	0.59	20	1.3%	-0.69 [-1.01, -0.37]
Luo 2013(1)	1.93	0.46	43	2.54	0.33	34	1.6%	-0.61 [-0.79, -0.43]
Luo 2013(2)	1.97	0.38	52	2.63	0.33	38	1.7%	-0.66 [-0.81, -0.51]
Meng 2022(1)	1.9	0.3	12	2.2	0.5	6	1.1%	-0.30 [-0.73, 0.13]
Meng 2022(2)	2	0.2	11	2.2	0.5	7	1.2%	-0.20 [-0.59, 0.19]
Mohammadi 2019	2.52	0.32	10	2.57	0.34	10	1.4%	-0.05 [-0.34, 0.24]
Park 2007	2.35	0.49	19	2.54	0.66	21	1.2%	-0.19 [-0.55, 0.17]
Ramezani 2017(1)	1.89	1.02	15	3.8	1.17	5	0.3%	-1.91 [-3.06, -0.76]
Ramezani 2017(2)	2.28	0.93	15	3.8	1.17	5	0.3%	-1.52 [-2.65, -0.39]
Ramezani 2017(3)	2.13	1.09	15	3.8	1.17	5	0.3%	-1.67 [-2.83, -0.51]
Saygın 2011	2.4	0.37	20	2.51	0.28	19	1.6%	-0.11 [-0.32, 0.10]
Seo 2012	2.7	2	10	2.34	1.23	10	0.2%	0.36 [-1.10, 1.82]
Seo 2019	2.69	0.64	26	2.9	0.58	44	1.4%	-0.21 [-0.51, 0.09]
Shalitin 2009	2.55	0.67	55	2.53	0.73	55	1.4%	0.02 [-0.24, 0.28]
Sun 2011	2.3	0.5	25	2.4	0.5	17	1.3%	-0.10 [-0.41, 0.21]
Wen 2008	2.21	0.47	24	3.29	0.87	13	0.9%	-1.08 [-1.59, -0.57]
Wong 2008	2.7	0.9	12	2.8	0.7	12	0.7%	-0.10 [-0.75, 0.55]
Woo 2004	2.6	0.8	41	2.7	0.7	41	1.3%	-0.10 [-0.43, 0.23]
Xianbo 2011	2.3	0.5	25	2.4	0.5	17	1.3%	-0.10 [-0.41, 0.21]
Xinhui 2016	2.21	0.61	14	2.68	0.75	14	0.9%	-0.47 [-0.98, 0.04]
Zehsaz 2016	1.87	0.25	16	2.24	0.19	16	1.7%	-0.37 [-0.52, -0.22]
Zehsaz 2017	2.54	0.92	16	2.47	0.87	16	0.8%	0.07 [-0.55, 0.69]
**Subtotal (95% CI)**			**1168**			**984**	**54.1%**	**-0.35 [-0.45, -0.25]**
Heterogeneity: Tau^2 = 0.09; Chi^2 = 275.89, df = 47 (P < 0.00001); I^2 = 83%								
Test for overall effect: Z = 6.63 (P < 0.00001)								
**2.7.2 Europe**								
Boer 2014(1)	2.47	0.24	17	2.48	0.55	7	1.1%	-0.01 [-0.43, 0.41]
Boer 2014(2)	2.52	0.74	15	2.48	0.55	7	0.9%	0.04 [-0.51, 0.59]
Bruyndonckx 2015	1.84	0.33	27	2.24	0.36	21	1.6%	-0.40 [-0.60, -0.20]
Elmahgoub 2009	2.26	0.72	15	2.41	0.54	15	1.0%	-0.15 [-0.61, 0.31]
Farpour-Lambert 2009	2.72	0.76	22	2.69	0.55	22	1.2%	0.03 [-0.36, 0.42]
Harder-Lauridsen 2014	2.3	0.75	14	2.6	0.25	14	1.1%	-0.30 [-0.71, 0.11]
Meyer 2006	2.57	0.66	33	3.01	0.79	34	1.3%	-0.44 [-0.79, -0.09]
Migueles 2023	2.34	0.39	38	2.52	0.39	33	1.6%	-0.18 [-0.36, 0.00]
Plavsic 2020	2.28	0.69	22	2.18	0.55	22	1.2%	0.10 [-0.27, 0.47]
Salus(2) 2022	2.05	0.37	14	2.6	0.37	14	1.4%	-0.55 [-0.82, -0.28]
**Subtotal (95% CI)**			**217**			**189**	**12.3%**	**-0.23 [-0.37, -0.09]**
Heterogeneity: Tau^2 = 0.02; Chi^2 = 16.47, df = 9 (P = 0.06); I^2 = 45%								
Test for overall effect: Z = 3.20 (P = 0.001)								
**2.7.3 Africa**								
Morgan 2021	2.37	0.24	15	3.12	0.33	15	1.6%	-0.75 [-0.96, -0.54]
Racil 2013(1)	2.18	0.4	11	2.62	0.18	6	1.4%	-0.44 [-0.72, -0.16]
Racil 2013(2)	2.55	0.32	11	2.62	0.18	6	1.5%	-0.07 [-0.31, 0.17]
**Subtotal (95% CI)**			**37**			**27**	**4.5%**	**-0.42 [-0.84, -0.01]**
Heterogeneity: Tau^2 = 0.12; Chi^2 = 17.93, df = 2 (P = 0.0001); I^2 = 89%								
Test for overall effect: Z = 2.01 (P = 0.04)								
**2.7.4 North America**								
Davis 2020	2.44	0.86	90	2.57	0.8	85	1.5%	-0.13 [-0.38, 0.12]
DeBar 2012	2.48	0.69	74	2.45	0.53	42	1.5%	0.03 [-0.19, 0.25]
Horner 2015(1)	2.2	0.71	27	2.27	0.34	8	1.2%	-0.07 [-0.43, 0.29]
Horner 2015(2)	1.86	0.43	23	2.27	0.34	8	1.4%	-0.41 [-0.70, -0.12]
Kelly 2004	2.36	0.76	10	2.32	0.73	10	0.7%	0.04 [-0.61, 0.69]
Murphy 2009	2.74	0.72	23	2.74	0.43	12	1.2%	0.00 [-0.38, 0.38]
Savoye 2007	2.52	0.92	75	2.52	0.68	44	1.4%	0.00 [-0.29, 0.29]
Savoye 2011	2.54	0.75	75	2.53	0.65	44	1.5%	0.01 [-0.25, 0.27]
Staiano 2017	2.34	0.4	20	2.46	0.57	18	1.3%	-0.12 [-0.44, 0.20]
Staiano 2018	2.19	0.8	22	2.76	0.74	23	1.0%	-0.57 [-1.02, -0.12]
Tas 2023	2.37	0.55	31	2.41	0.55	6	1.0%	-0.04 [-0.52, 0.44]
**Subtotal (95% CI)**			**470**			**300**	**13.7%**	**-0.10 [-0.20, 0.00]**
Heterogeneity: Tau^2 = 0.00; Chi^2 = 11.50, df = 10 (P = 0.32); I^2 = 13%								
Test for overall effect: Z = 1.90 (P = 0.06)								
**2.7.5 South America**								
Bezerra 2023	2.45	0.41	20	2.82	0.62	21	1.3%	-0.37 [-0.69, -0.05]
Boff 2020	2.25	0.74	34	2.08	0.75	31	1.2%	0.17 [-0.19, 0.53]
Faria 2020(1)	1.85	0.49	24	2.07	0.5	13	1.3%	-0.22 [-0.56, 0.12]
Faria 2020(2)	2.03	0.5	26	2.07	0.5	13	1.3%	-0.04 [-0.37, 0.29]
Lira 2017(1)	2.47	0.93	26	2.89	0.69	16	1.0%	-0.42 [-0.91, 0.07]
Lira 2017(2)	2.48	0.72	25	2.89	0.69	17	1.1%	-0.41 [-0.84, 0.02]
Lopes 2016	2.41	0.8	17	2.39	0.96	16	0.8%	0.02 [-0.58, 0.62]
Machado 2022	2.15	0.6	20	2.13	0.56	15	1.2%	0.02 [-0.37, 0.41]
Monteiro 2015(1)	1.3	0.72	18	1.79	0.71	8	0.8%	-0.49 [-1.08, 0.10]
Monteiro 2015(2)	1.05	0.54	14	1.79	0.71	8	0.8%	-0.74 [-1.31, -0.17]
Vasconcellos 2016	2.61	0.33	10	3.03	0.82	10	0.9%	-0.42 [-0.97, 0.13]
**Subtotal (95% CI)**			**234**			**168**	**11.6%**	**-0.23 [-0.38, -0.07]**
Heterogeneity: Tau^2 = 0.02; Chi^2 = 14.38, df = 10 (P = 0.16); I^2 = 30%								
Test for overall effect: Z = 2.86 (P = 0.004)								
**2.7.6 Oceania**								
Benson 2008	2.3	0.9	32	2.4	0.6	35	1.2%	-0.10 [-0.47, 0.27]
Dias 2018(1)	2.1	0.5	19	2.36	0.7	9	0.9%	-0.26 [-0.77, 0.25]
Dias 2018(2)	2.55	0.84	22	2.36	0.7	10	0.9%	0.19 [-0.37, 0.75]
Tsang 2009	2.62	0.75	11	2.33	0.57	8	0.8%	0.29 [-0.30, 0.88]
**Subtotal (95% CI)**			**84**			**62**	**3.8%**	**-0.02 [-0.26, 0.22]**
Heterogeneity: Tau^2 = 0.00; Chi^2 = 2.62, df = 3 (P = 0.45); I^2 = 0%								
Test for overall effect: Z = 0.14 (P = 0.89)								
**Total (95% CI)**			**2210**			**1730**	**100.0%**	**-0.28 [-0.35, -0.20]**
Heterogeneity: Tau^2 = 0.07; Chi^2 = 383.87, df = 86 (P < 0.00001); I^2 = 78%								
Test for overall effect: Z = 7.58 (P < 0.00001)								
Test for subgroup differences: Chi^2 = 14.80, df = 5 (P = 0.01), I^2 = 66.2%								

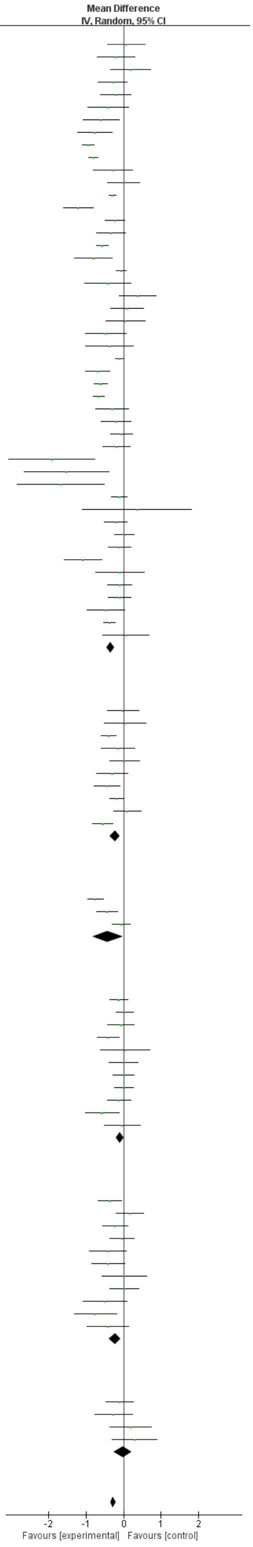

Figure S14.8. Forest plot of subgroup analyses by economic status

Study or Subgroup	Experimental Mean	Experimental SD	Experimental Total	Control Mean	Control SD	Control Total	Weight	Mean Difference IV, Random, 95% CI
**2.8.1 Lower-middle-income countries**								
Morgan 2021	2.37	0.24	15	3.12	0.33	15	1.6%	-0.75 [-0.96, -0.54]
Racil 2013(1)	2.18	0.4	11	2.62	0.18	6	1.4%	-0.44 [-0.72, -0.16]
Racil 2013(2)	2.55	0.32	11	2.62	0.18	6	1.5%	-0.07 [-0.31, 0.17]
**Subtotal (95% CI)**			**37**			**27**	**4.5%**	**-0.42 [-0.84, -0.01]**
Heterogeneity: Tau^2 = 0.12; Chi^2 = 17.93, df = 2 (P = 0.0001); I^2 = 89%								
Test for overall effect: Z = 2.01 (P = 0.04)								
**2.8.2 Upper-middle-income countries**								
Ahmadi 2020(1)	2.37	0.45	28	2.3	0.72	9	1.0%	0.07 [-0.43, 0.57]
Ahmadi 2020(2)	2.1	0.52	29	2.3	0.72	9	0.9%	-0.20 [-0.71, 0.31]
Ahmadi 2020(3)	2.49	0.68	29	2.3	0.72	9	0.9%	0.19 [-0.34, 0.72]
Bezerra 2023	2.45	0.41	20	2.82	0.62	21	1.3%	-0.37 [-0.69, -0.05]
Boff 2020	2.25	0.74	34	2.08	0.75	31	1.2%	0.17 [-0.19, 0.53]
Bowen 2010	3	0.68	22	3.29	0.59	18	1.2%	-0.29 [-0.68, 0.10]
Chuensiri 2018(1)	2.28	0.51	11	2.69	0.51	5	0.9%	-0.41 [-0.95, 0.13]
Chuensiri 2018(2)	2.09	0.5	15	2.69	0.51	6	1.0%	-0.60 [-1.08, -0.12]
Faria 2020(1)	1.85	0.49	24	2.07	0.5	13	1.3%	-0.22 [-0.56, 0.12]
Faria 2020(2)	2.03	0.5	26	2.07	0.5	13	1.3%	-0.04 [-0.37, 0.29]
Ghorbanian 2013	3.12	0.57	15	3.88	0.72	15	1.0%	-0.76 [-1.22, -0.30]
Guijun 2009	2.35	0.06	16	3.29	0.25	10	1.6%	-0.94 [-1.10, -0.78]
Gulijianati 2022	2.17	0.29	47	2.98	0.35	46	1.7%	-0.81 [-0.94, -0.68]
Guo 2015	2.74	0.48	23	3.02	0.87	12	0.9%	-0.28 [-0.81, 0.25]
Hongling 2012	2.6	0.7	30	2.6	1	30	1.1%	0.00 [-0.44, 0.44]
Jian 2014	2.33	0.21	44	2.63	0.24	44	1.7%	-0.30 [-0.39, -0.21]
Jianjun 2012	1.71	0.48	20	2.92	0.67	15	1.1%	-1.21 [-1.61, -0.81]
Jun 2006(1)	1.91	0.41	33	2.14	0.64	31	1.4%	-0.23 [-0.50, 0.04]
Jun 2006(2)	1.8	0.43	35	2.13	1.04	32	1.2%	-0.33 [-0.72, 0.06]
Karacabey 2009	1.75	0.24	20	2.32	0.28	20	1.6%	-0.57 [-0.73, -0.41]
Ke 2024	2.6	0.4	10	3.4	0.7	10	1.0%	-0.80 [-1.30, -0.30]
Kelishadi 2008	3.1	0.36	45	3.16	0.33	47	1.7%	-0.06 [-0.20, 0.08]
Liangmei 2015	2.21	0.59	12	2.68	0.74	12	0.9%	-0.47 [-1.01, 0.07]
Lingling 2021	4	1.05	20	4.38	1	20	0.7%	-0.38 [-1.02, 0.26]
Liqun 2014	2.39	0.38	76	2.49	0.4	97	1.7%	-0.10 [-0.22, 0.02]
Lira 2017(1)	2.47	0.93	26	2.89	0.69	16	1.0%	-0.42 [-0.91, 0.07]
Lira 2017(2)	2.48	0.72	25	2.89	0.69	17	1.1%	-0.41 [-0.84, 0.02]
Liu 2018	1.98	0.52	30	2.67	0.59	20	1.3%	-0.69 [-1.01, -0.37]
Lopes 2016	2.41	0.8	17	2.39	0.96	16	0.8%	0.02 [-0.58, 0.62]
Luo 2013(1)	1.93	0.46	43	2.54	0.33	34	1.6%	-0.61 [-0.79, -0.43]
Luo 2013(2)	1.97	0.38	52	2.63	0.33	38	1.7%	-0.66 [-0.81, -0.51]
Machado 2022	2.15	0.6	20	2.13	0.56	15	1.2%	0.02 [-0.37, 0.41]
Meng 2022(1)	1.9	0.3	12	2.2	0.5	6	1.1%	-0.30 [-0.73, 0.13]
Meng 2022(2)	2	0.2	11	2.2	0.5	7	1.2%	-0.20 [-0.59, 0.19]
Mohammadi 2019	2.52	0.32	10	2.57	0.34	10	1.4%	-0.05 [-0.34, 0.24]
Monteiro 2015(1)	1.3	0.72	18	1.79	0.71	8	0.8%	-0.49 [-1.08, 0.10]
Monteiro 2015(2)	1.05	0.54	14	1.79	0.71	8	0.8%	-0.74 [-1.31, -0.17]
Plavsic 2020	2.28	0.69	22	2.18	0.55	22	1.2%	0.10 [-0.27, 0.47]
Ramezani 2017(1)	1.89	1.02	15	3.8	1.17	5	0.3%	-1.91 [-3.06, -0.76]
Ramezani 2017(2)	2.28	0.93	15	3.8	1.17	5	0.3%	-1.52 [-2.65, -0.39]
Ramezani 2017(3)	2.13	1.09	15	3.8	1.17	5	0.3%	-1.67 [-2.83, -0.51]
Saygın 2011	2.4	0.37	20	2.51	0.28	19	1.6%	-0.11 [-0.32, 0.10]
Sun 2011	2.3	0.5	25	2.4	0.5	17	1.3%	-0.10 [-0.41, 0.21]
Vasconcellos 2016	2.61	0.33	10	3.03	0.82	10	0.9%	-0.42 [-0.97, 0.13]
Wen 2008	2.21	0.47	24	3.29	0.87	13	0.9%	-1.08 [-1.59, -0.57]
Woo 2004	2.6	0.8	41	2.7	0.7	41	1.3%	-0.10 [-0.43, 0.23]
Xianbo 2011	2.3	0.5	25	2.4	0.5	17	1.3%	-0.10 [-0.41, 0.21]
Xinhui 2016	2.21	0.61	14	2.68	0.75	14	0.9%	-0.47 [-0.98, 0.04]
Zehsaz 2016	1.87	0.25	16	2.24	0.19	16	1.7%	-0.37 [-0.52, -0.22]
Zehsaz 2017	2.54	0.92	16	2.47	0.87	16	0.8%	0.07 [-0.55, 0.69]
**Subtotal (95% CI)**			**1220**			**970**	**57.1%**	**-0.37 [-0.47, -0.27]**
Heterogeneity: Tau^2 = 0.08; Chi^2 = 273.66, df = 49 (P < 0.00001); I^2 = 82%								
Test for overall effect: Z = 7.31 (P < 0.00001)								
**2.8.3 High-income countries**								
Benson 2008	2.3	0.9	32	2.4	0.6	35	1.2%	-0.10 [-0.47, 0.27]
Boer 2014(1)	2.47	0.24	17	2.48	0.55	7	1.1%	-0.01 [-0.43, 0.41]
Boer 2014(2)	2.52	0.74	15	2.48	0.55	7	0.9%	0.04 [-0.51, 0.59]
Bruyndonckx 2015	1.84	0.33	27	2.24	0.36	21	1.6%	-0.40 [-0.60, -0.20]
Chae 2010	2.33	0.73	19	2.54	0.57	19	1.1%	-0.21 [-0.63, 0.21]
Davis 2020	2.44	0.86	90	2.57	0.8	85	1.5%	-0.13 [-0.38, 0.12]
DeBar 2012	2.48	0.69	74	2.45	0.53	42	1.5%	0.03 [-0.19, 0.25]
Dias 2018(1)	2.1	0.5	19	2.36	0.7	9	0.9%	-0.26 [-0.77, 0.25]
Dias 2018(2)	2.55	0.84	22	2.36	0.7	10	0.9%	0.19 [-0.37, 0.75]
Elmahgoub 2009	2.26	0.72	15	2.41	0.54	15	1.0%	-0.15 [-0.61, 0.31]
Farpour-Lambert 2009	2.72	0.76	22	2.69	0.55	22	1.2%	0.03 [-0.36, 0.42]
Harder-Lauridsen 2014	2.3	0.75	14	2.6	0.25	14	1.1%	-0.30 [-0.71, 0.11]
Horner 2015(1)	2.2	0.71	27	2.27	0.34	8	1.2%	-0.07 [-0.43, 0.29]
Horner 2015(2)	1.86	0.43	23	2.27	0.34	8	1.4%	-0.41 [-0.70, -0.12]
Kelly 2004	2.36	0.76	10	2.32	0.73	10	0.7%	0.04 [-0.61, 0.69]
Kim 2007	2.54	0.74	14	2.96	0.85	12	0.8%	-0.42 [-1.04, 0.20]
Kim 2022	2.23	0.71	13	1.85	0.56	13	1.0%	0.38 [-0.11, 0.87]
Lee 2010(1)	2.73	0.42	20	2.63	0.61	9	1.1%	0.10 [-0.34, 0.54]
Lee 2010(2)	2.68	0.71	16	2.63	0.61	9	0.9%	0.05 [-0.48, 0.58]
Meyer 2006	2.57	0.66	33	3.01	0.79	34	1.3%	-0.44 [-0.79, -0.09]
Migueles 2023	2.34	0.39	38	2.52	0.39	33	1.6%	-0.18 [-0.36, 0.00]
Murphy 2009	2.74	0.72	23	2.74	0.43	12	1.2%	0.00 [-0.38, 0.38]
Park 2007	2.35	0.49	19	2.54	0.66	21	1.2%	-0.19 [-0.55, 0.17]
Salus(2) 2022	2.05	0.37	14	2.6	0.37	14	1.4%	-0.55 [-0.82, -0.28]
Savoye 2007	2.52	0.92	75	2.52	0.68	44	1.4%	0.00 [-0.29, 0.29]
Savoye 2011	2.54	0.75	75	2.53	0.65	44	1.5%	0.01 [-0.25, 0.27]
Seo 2012	2.7	2	10	2.34	1.23	10	0.2%	0.36 [-1.10, 1.82]
Seo 2019	2.69	0.64	26	2.9	0.58	44	1.4%	-0.21 [-0.51, 0.09]
Shalitin 2009	2.55	0.67	55	2.53	0.73	55	1.4%	0.02 [-0.24, 0.28]
Staiano 2017	2.34	0.4	20	2.46	0.57	18	1.3%	-0.12 [-0.44, 0.20]
Staiano 2018	2.19	0.8	22	2.76	0.74	23	1.0%	-0.57 [-1.02, -0.12]
Tas 2023	2.37	0.55	31	2.41	0.55	6	1.0%	-0.04 [-0.52, 0.44]
Tsang 2009	2.62	0.75	11	2.33	0.57	8	0.8%	0.29 [-0.30, 0.88]
Wong 2008	2.7	0.9	12	2.8	0.7	12	0.7%	-0.10 [-0.75, 0.55]
**Subtotal (95% CI)**			**953**			**733**	**38.4%**	**-0.14 [-0.21, -0.07]**
Heterogeneity: Tau^2 = 0.01; Chi^2 = 45.15, df = 33 (P = 0.08); I^2 = 27%								
Test for overall effect: Z = 3.77 (P = 0.0002)								
**Total (95% CI)**			**2210**			**1730**	**100.0%**	**-0.28 [-0.35, -0.20]**
Heterogeneity: Tau^2 = 0.07; Chi^2 = 383.87, df = 86 (P < 0.00001); I^2 = 78%								
Test for overall effect: Z = 7.58 (P < 0.00001)								
Test for subgroup differences: Chi^2 = 14.29, df = 2 (P = 0.0008), I^2 = 86.0%								

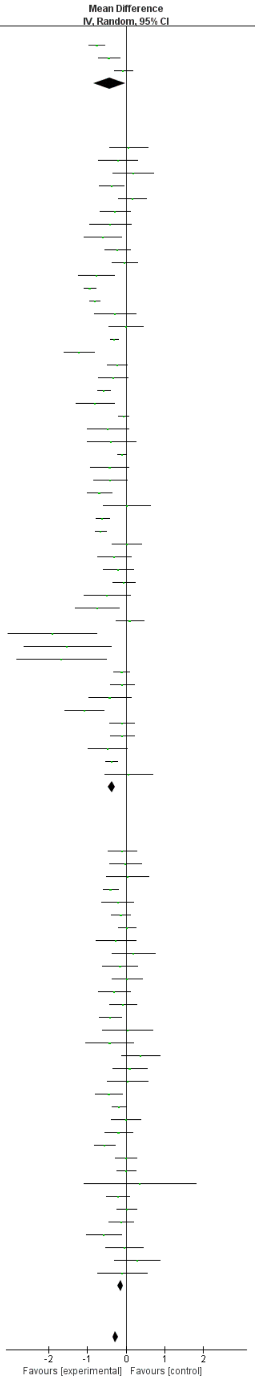

Figure S14.9. Forest plot of subgroup analyses by weight status

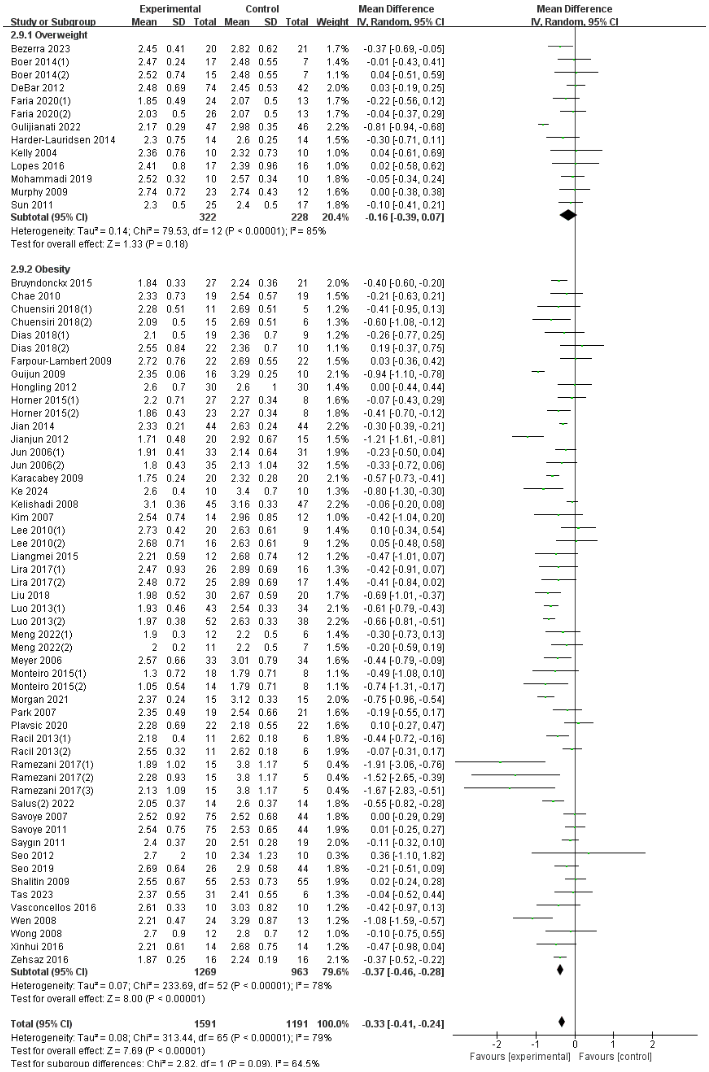

Figure S14.10. Forest plot of subgroup analyses by sex

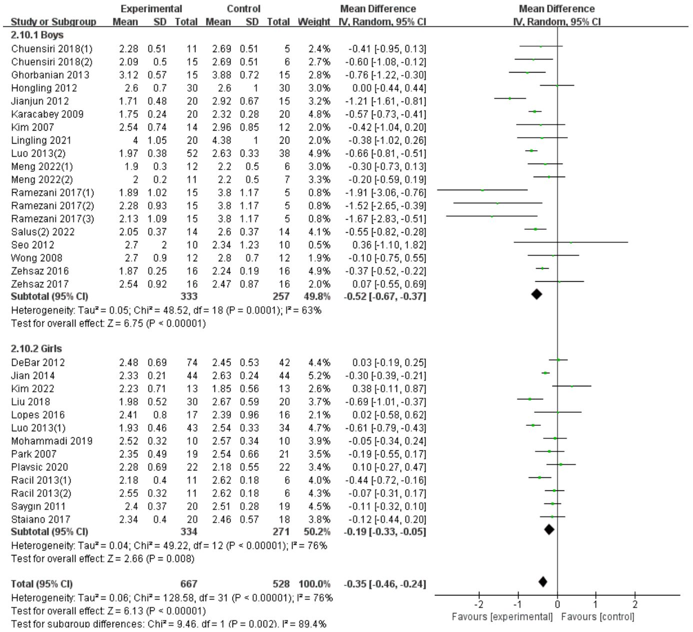

Figure S14.11. Forest plot of subgroup analyses by metabolic status

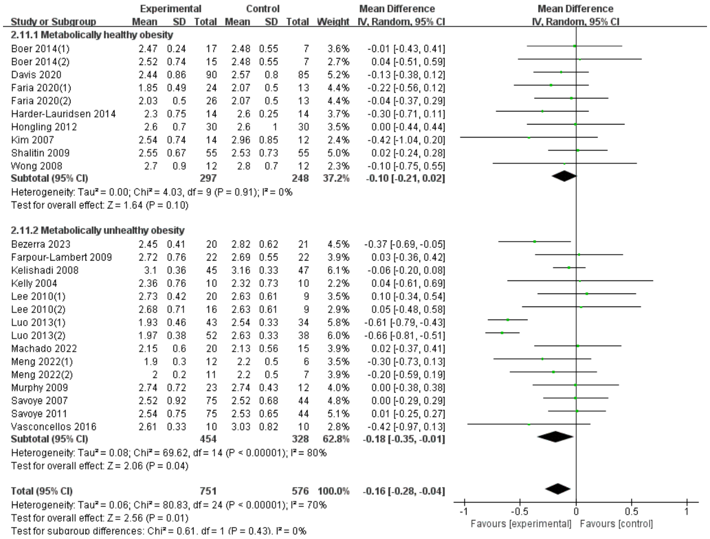

Figure S15. Forest plots of subgroup analyses for FPG in children and adolescents with overweight or obesity

Figure S15.1. Forest plot of subgroup analyses by type of exercise

Study or Subgroup	Experimental Mean	Experimental SD	Experimental Total	Control Mean	Control SD	Control Total	Weight	Mean Difference IV, Random, 95% CI
**2.1.1 Aerobic exercise**								
Abassi 2020(1)	4.88	0.4	8	5.2	0.21	4	1.0%	-0.32 [-0.67, 0.03]
Bezerra 2023	4.7	0.34	20	4.64	0.38	21	1.2%	0.06 [-0.16, 0.28]
Boer 2014(2)	4.72	0.28	15	4.72	0.23	7	1.2%	0.00 [-0.22, 0.22]
Bowen 2010	4.79	0.38	22	4.64	0.41	18	1.2%	0.15 [-0.10, 0.40]
Carrel 2005	4.83	0.28	27	5.16	0.39	23	1.3%	-0.33 [-0.52, -0.14]
Davis 2020	5.59	0.48	90	5.61	0.6	85	1.3%	-0.02 [-0.18, 0.14]
DeBar 2012	4.63	0.46	74	4.59	0.49	42	1.3%	0.04 [-0.14, 0.22]
Dias 2018(2)	5.19	0.37	22	5.08	0.39	9	1.1%	0.11 [-0.19, 0.41]
Ghorbanian 2013	5.42	0.52	15	5.34	0.53	15	1.0%	0.08 [-0.30, 0.46]
Gulijianati 2022	4.83	0.36	47	5.46	0.41	46	1.3%	-0.63 [-0.79, -0.47]
Guo 2015	4.92	0.4	23	5.06	0.59	12	1.0%	-0.14 [-0.51, 0.23]
Jiafen 2017	6.11	0.62	78	7.82	0.84	78	1.2%	-1.71 [-1.94, -1.48]
Jianjun 2012	3.78	0.79	20	5.62	0.74	15	0.8%	-1.84 [-2.35, -1.33]
Jun 2006(1)	3.92	0.45	33	4.18	0.46	31	1.2%	-0.26 [-0.48, -0.04]
Jun 2006(2)	3.97	0.27	35	4.03	0.37	32	1.3%	-0.06 [-0.22, 0.10]
Kelishadi 2008	4.51	0.46	45	4.5	0.44	47	1.3%	0.01 [-0.17, 0.19]
Kelly 2004	4.66	0.25	10	4.8	0.47	10	1.0%	-0.14 [-0.47, 0.19]
Kim 2007	4.72	0.33	14	4.72	0.37	12	1.1%	0.00 [-0.27, 0.27]
Kim 2020	4.82	0.12	24	5.22	0.15	24	1.4%	-0.40 [-0.48, -0.32]
Lee 2010(2)	4.95	0.33	16	5.11	0.51	9	1.0%	-0.16 [-0.53, 0.21]
Lee 2012(1)	5.31	0.22	15	5.37	0.27	5	1.1%	-0.06 [-0.32, 0.20]
Lee 2013(1)	5.04	0.61	14	5.47	0.21	4	1.0%	-0.43 [-0.81, -0.05]
Lidan 2012	4.18	0.72	30	4.78	1.01	30	0.9%	-0.60 [-1.04, -0.16]
Lira 2017(1)	4.54	0.53	26	4.4	0.3	16	1.2%	0.14 [-0.11, 0.39]
Lira 2017(2)	4.63	0.53	25	4.4	0.3	17	1.2%	0.23 [-0.02, 0.48]
Liu 2018	4.32	0.3	30	4.71	0.39	20	1.2%	-0.39 [-0.59, -0.19]
Luo 2013(1)	3.76	0.71	43	4.08	0.6	34	1.1%	-0.32 [-0.61, -0.03]
Luo 2013(2)	4.19	0.72	52	4.25	0.63	38	1.1%	-0.06 [-0.34, 0.22]
Machado 2022	5.06	0.29	20	5.39	0.51	15	1.1%	-0.33 [-0.62, -0.04]
Meng 2022(2)	4.9	0.4	11	5.4	0.4	7	1.0%	-0.50 [-0.88, -0.12]
Murphy 2009	5.07	0.24	23	5.08	0.16	12	1.3%	-0.01 [-0.14, 0.12]
Park 2007	4.77	0.52	19	5.28	0.63	21	1.0%	-0.51 [-0.87, -0.15]
Racil(1) 2016(2)	4.7	0.2	16	4.6	0.4	7	1.1%	0.10 [-0.21, 0.41]
Racil 2013(2)	4.4	0.34	11	4.6	0.32	6	1.0%	-0.20 [-0.53, 0.13]
Racil 2024(2)	4.05	0.4	12	4.3	0.4	6	0.9%	-0.25 [-0.64, 0.14]
Ramezani 2017(1)	4.89	0.61	15	5.86	0.92	5	0.4%	-0.97 [-1.83, -0.11]
Savoye 2007	4.92	0.36	75	4.9	0.48	44	1.3%	0.02 [-0.14, 0.18]
Savoye 2011	4.97	0.27	75	4.88	0.3	44	1.3%	0.09 [-0.02, 0.20]
Seo 2012	4.66	0.77	10	4.75	0.68	10	0.6%	-0.09 [-0.73, 0.55]
Staiano 2017	4.95	1.76	20	5.74	1.78	18	0.3%	-0.79 [-1.92, 0.34]
Staiano 2018	5.01	0.3	22	4.95	0.25	23	1.3%	0.06 [-0.10, 0.22]
Tjonna 2009	4.9	0.31	20	5	0.33	22	1.2%	-0.10 [-0.29, 0.09]
Tsang 2009	5.15	0.48	11	4.83	0.39	8	0.9%	0.32 [-0.07, 0.71]
Woo 2004	4.4	0.6	41	4.3	0.6	41	1.2%	0.10 [-0.16, 0.36]
Xianbo 2011	4.8	0.4	25	4.6	0.4	17	1.2%	0.20 [-0.05, 0.45]
Xishao 2009	4.18	0.72	34	4.78	1.01	33	0.9%	-0.60 [-1.02, -0.18]
Xishao 2010	4.18	0.72	100	4.78	1.01	100	1.2%	-0.60 [-0.84, -0.36]
Yu 2020	4.6	0.5	99	5.1	0.4	72	1.3%	-0.50 [-0.64, -0.36]
Zhaogang 2021	4.03	0.28	49	4.25	0.54	49	1.3%	-0.22 [-0.39, -0.05]
**Subtotal (95% CI)**			**1581**			**1264**	**53.6%**	**-0.21 [-0.31, -0.11]**
Heterogeneity: Tau² = 0.10; Chi² = 455.76, df = 48 (P < 0.00001); I² = 89%								
Test for overall effect: Z = 4.19 (P < 0.0001)								
**2.1.2 Anaerobic exercise**								
Abassi 2020(2)	5	0.39	8	5.2	0.21	4	1.0%	-0.20 [-0.54, 0.14]
Boer 2014(1)	4.72	0.39	17	4.72	0.23	7	1.2%	0.00 [-0.25, 0.25]
Dias 2018(1)	5.13	0.37	19	5.08	0.39	8	1.1%	0.05 [-0.27, 0.37]
Ke 2024	5.2	0.4	10	5.6	0.6	10	0.9%	-0.40 [-0.85, 0.05]
Meng 2022(1)	4.7	0.3	12	5.4	0.4	6	1.0%	-0.70 [-1.06, -0.34]
Pena 2022	5.49	0.49	79	5.88	1.37	38	0.9%	-0.39 [-0.84, 0.06]
Racil(1) 2016(1)	4.6	0.4	17	4.6	0.4	7	1.0%	0.00 [-0.35, 0.35]
Racil(2) 2016(1)	4.6	0.4	23	4.8	0.4	9	1.1%	-0.20 [-0.51, 0.11]
Racil(2) 2016(2)	4.1	0.4	26	4.8	0.4	10	1.1%	-0.70 [-0.99, -0.41]
Racil 2013(1)	4.5	0.47	11	4.6	0.32	6	1.0%	-0.10 [-0.48, 0.28]
Racil 2024(1)	4.35	0.4	12	4.3	0.4	5	0.9%	0.05 [-0.37, 0.47]
Salus(2) 2022	5.11	0.37	14	5.19	0.37	14	1.1%	-0.08 [-0.35, 0.19]
Tas 2023	5.09	0.49	31	5.05	0.49	6	0.9%	0.04 [-0.39, 0.47]
Vasconcellos 2016	5.1	0.36	10	5.23	0.46	10	1.0%	-0.13 [-0.49, 0.23]
**Subtotal (95% CI)**			**289**			**140**	**14.0%**	**-0.19 [-0.33, -0.05]**
Heterogeneity: Tau² = 0.04; Chi² = 29.80, df = 13 (P = 0.005); I² = 56%								
Test for overall effect: Z = 2.73 (P = 0.006)								
**2.1.3 Resistance exercise**								
Benson 2008	5.2	0.4	32	5	0.5	35	1.2%	0.20 [-0.02, 0.42]
Davis(1) 2009	4.91	0.48	17	5.07	0.36	21	1.1%	-0.16 [-0.44, 0.12]
DAVIS(2) 2009(1)	4.68	0.37	9	5.11	0.22	5	1.1%	-0.43 [-0.74, -0.12]
Kelly 2019	5.36	0.29	13	5.23	0.29	13	1.2%	0.13 [-0.09, 0.35]
Lee 2012(2)	5.27	0.23	16	5.37	0.27	6	1.2%	-0.10 [-0.34, 0.14]
Lee 2013(2)	5.26	0.6	14	5.47	0.21	4	1.0%	-0.21 [-0.59, 0.17]
Ramezani 2017(2)	5.26	0.77	15	5.86	0.92	5	0.4%	-0.60 [-1.50, 0.30]
Shaibi 2006	5.21	0.35	11	5.09	0.35	10	1.1%	0.12 [-0.18, 0.42]
**Subtotal (95% CI)**			**127**			**99**	**8.3%**	**-0.06 [-0.23, 0.10]**
Heterogeneity: Tau² = 0.03; Chi² = 17.32, df = 7 (P = 0.02); I² = 60%								
Test for overall effect: Z = 0.74 (P = 0.46)								
**2.1.4 Combined aerobic and resistance exercise**								
Bharath 2018	4.3	0.89	20	5.4	0.44	20	0.9%	-1.10 [-1.54, -0.66]
DAVIS(2) 2009(2)	4.82	0.34	15	5.11	0.22	5	1.2%	-0.29 [-0.55, -0.03]
Duft 2020	4.55	0.41	18	4.75	0.47	19	1.1%	-0.20 [-0.48, 0.08]
Faria 2020(1)	4.65	0.38	24	4.71	0.39	13	1.1%	-0.06 [-0.32, 0.20]
Faria 2020(2)	4.7	0.39	26	4.71	0.39	13	1.2%	-0.01 [-0.27, 0.25]
Farpour-Lambert 2009	4.95	0.26	22	4.79	0.31	22	1.3%	0.16 [-0.01, 0.33]
Harder-Lauridsen 2014	4.5	0.16	14	4.4	0.4	14	1.2%	0.10 [-0.13, 0.33]
Hongling 2012	4.1	0.8	30	4.2	0.4	30	1.1%	-0.10 [-0.42, 0.22]
Kim 2022	5	0.82	13	4.45	0.24	13	0.8%	0.55 [0.09, 1.01]
Lee 2010(1)	4.55	0.49	20	5.11	0.51	9	0.9%	-0.56 [-0.96, -0.16]
Lima 2020	4.58	0.34	198	4.71	0.38	100	1.4%	-0.13 [-0.22, -0.04]
Lopes 2016	4.52	0.34	17	4.88	0.31	16	1.2%	-0.36 [-0.58, -0.14]
McCormack 2014	4.38	0.18	10	4.24	0.27	8	1.2%	0.14 [-0.08, 0.36]
Migueles 2023	4.66	0.45	46	4.65	0.45	42	1.3%	0.01 [-0.18, 0.20]
Mohammadi 2019	4.3	0.29	10	4.57	0.37	10	1.1%	-0.27 [-0.56, 0.02]
Ramezani 2017(3)	5.06	0.63	15	5.86	0.92	5	0.4%	-0.80 [-1.67, 0.07]
Shalitin 2009	4.49	0.4	55	4.55	0.45	55	1.3%	-0.06 [-0.22, 0.10]
Son 2017	4.82	0.52	20	5.22	0.67	20	1.0%	-0.40 [-0.77, -0.03]
Wen 2008	3.97	0.31	24	5.23	0.69	13	0.9%	-1.26 [-1.66, -0.86]
Wong 2008	4.7	0.4	12	4.8	0.8	12	0.8%	-0.10 [-0.61, 0.41]
Wong 2018	4.3	0.77	15	5.4	0.39	15	0.9%	-1.10 [-1.54, -0.66]
Zehsaz 2016	4.8	0.5	16	4.8	0.5	16	1.0%	0.00 [-0.35, 0.35]
Zehsaz 2017	4.8	0.5	16	4.8	0.5	16	1.0%	0.00 [-0.35, 0.35]
**Subtotal (95% CI)**			**656**			**486**	**24.2%**	**-0.21 [-0.34, -0.08]**
Heterogeneity: Tau² = 0.07; Chi² = 119.47, df = 22 (P < 0.00001); I² = 82%								
Test for overall effect: Z = 3.28 (P = 0.001)								
**Total (95% CI)**			**2653**			**1989**	**100.0%**	**-0.20 [-0.26, -0.13]**
Heterogeneity: Tau² = 0.08; Chi² = 637.17, df = 93 (P < 0.00001); I² = 85%								
Test for overall effect: Z = 5.81 (P < 0.00001)								
Test for subgroup differences: Chi² = 2.58, df = 3 (P = 0.46), I² = 0%								

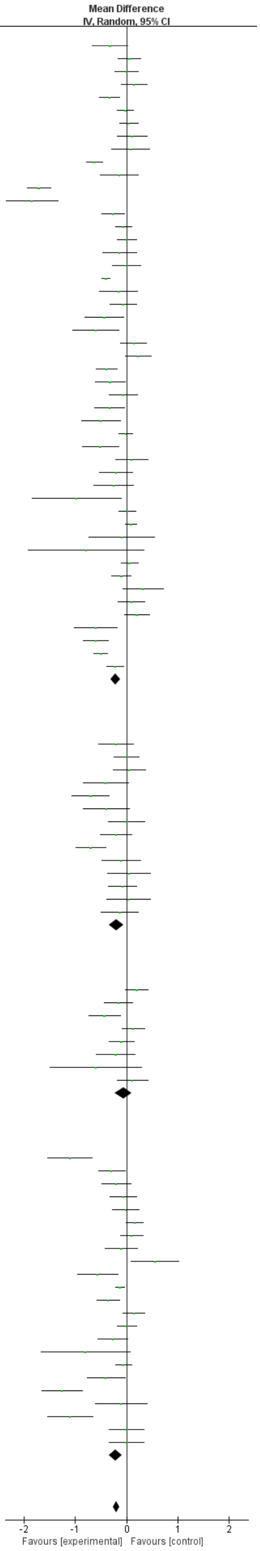

Figure S15.2. Forest plot of subgroup analyses by exercise intensity

Study or Subgroup	Experimental Mean	Experimental SD	Experimental Total	Control Mean	Control SD	Control Total	Weight	Mean Difference IV, Random, 95% CI
**2.2.1 Moderate-to-low intensity**								
Abassi 2020(1)	4.88	0.4	8	5.2	0.21	4	1.0%	-0.32 [-0.67, 0.03]
Bharath 2018	4.3	0.89	20	5.4	0.44	20	0.9%	-1.10 [-1.54, -0.66]
Boer 2014(2)	4.72	0.28	15	4.72	0.23	7	1.2%	0.00 [-0.22, 0.22]
Bowen 2010	4.79	0.38	22	4.64	0.41	18	1.2%	0.15 [-0.10, 0.40]
Carrel 2005	4.83	0.28	27	5.16	0.39	23	1.3%	-0.33 [-0.52, -0.14]
Davis 2020	5.59	0.48	90	5.61	0.6	85	1.3%	-0.02 [-0.18, 0.14]
DeBar 2012	4.63	0.46	74	4.59	0.49	42	1.3%	0.04 [-0.14, 0.22]
Dias 2018(2)	5.19	0.37	22	5.08	0.39	9	1.1%	0.11 [-0.19, 0.41]
Faria 2020(1)	4.65	0.38	24	4.71	0.39	13	1.1%	-0.06 [-0.32, 0.20]
Farpour-Lambert 2009	4.95	0.26	22	4.79	0.31	22	1.3%	0.16 [-0.01, 0.33]
Ghorbanian 2013	5.42	0.52	15	5.34	0.53	15	1.0%	0.08 [-0.30, 0.46]
Guo 2015	4.92	0.4	23	5.06	0.59	12	1.0%	-0.14 [-0.51, 0.23]
Harder-Lauridsen 2014	4.5	0.16	14	4.4	0.4	14	1.2%	0.10 [-0.13, 0.33]
Jiafen 2017	6.11	0.62	78	7.82	0.84	78	1.2%	-1.71 [-1.94, -1.48]
Jianjun 2012	3.78	0.79	20	5.62	0.74	15	0.8%	-1.84 [-2.35, -1.33]
Jun 2006(1)	3.92	0.45	33	4.18	0.46	31	1.2%	-0.26 [-0.48, -0.04]
Jun 2006(2)	3.97	0.27	35	4.03	0.37	32	1.3%	-0.06 [-0.22, 0.10]
Kelishadi 2008	4.51	0.46	45	4.5	0.44	47	1.3%	0.01 [-0.17, 0.19]
Kelly 2004	4.66	0.25	10	4.8	0.47	10	1.0%	-0.14 [-0.47, 0.19]
Kelly 2019	5.36	0.29	13	5.23	0.29	13	1.2%	0.13 [-0.09, 0.35]
Kim 2007	4.72	0.33	14	4.72	0.37	12	1.1%	0.00 [-0.27, 0.27]
Kim 2020	4.82	0.12	24	5.22	0.15	24	1.4%	-0.40 [-0.48, -0.32]
Kim 2022	5	0.82	13	4.45	0.24	13	0.8%	0.55 [0.09, 1.01]
Lee 2010(1)	4.55	0.49	20	5.11	0.51	9	0.9%	-0.56 [-0.96, -0.16]
Lee 2010(2)	4.95	0.33	16	5.11	0.51	9	1.0%	-0.16 [-0.53, 0.21]
Lee 2012(1)	5.31	0.22	15	5.37	0.27	5	1.1%	-0.06 [-0.32, 0.20]
Lee 2013(1)	5.04	0.61	14	5.47	0.21	4	1.0%	-0.43 [-0.81, -0.05]
Lidan 2012	4.18	0.72	30	4.78	1.01	30	0.9%	-0.60 [-1.04, -0.16]
Lima 2020	4.58	0.34	198	4.71	0.38	100	1.4%	-0.13 [-0.22, -0.04]
Lira 2017(2)	4.63	0.53	25	4.4	0.3	17	1.2%	0.23 [-0.02, 0.48]
Liu 2018	4.32	0.3	30	4.71	0.39	20	1.2%	-0.39 [-0.59, -0.19]
Luo 2013(1)	3.76	0.71	43	4.08	0.6	34	1.1%	-0.32 [-0.61, -0.03]
Luo 2013(2)	4.19	0.72	52	4.25	0.63	38	1.1%	-0.06 [-0.34, 0.22]
Machado 2022	5.06	0.29	20	5.39	0.51	15	1.1%	-0.33 [-0.62, -0.04]
Meng 2022(2)	4.9	0.4	11	5.4	0.4	7	1.0%	-0.50 [-0.88, -0.12]
Mohammadi 2019	4.3	0.29	10	4.57	0.37	10	1.1%	-0.27 [-0.56, 0.02]
Murphy 2009	5.07	0.24	23	5.08	0.16	12	1.3%	-0.01 [-0.14, 0.12]
Park 2007	4.77	0.52	19	5.28	0.63	21	1.0%	-0.51 [-0.87, -0.15]
Racil(1) 2016(2)	4.7	0.2	16	4.6	0.4	7	1.1%	0.10 [-0.21, 0.41]
Racil 2013(2)	4.4	0.34	11	4.6	0.32	6	1.0%	-0.20 [-0.53, 0.13]
Racil 2024(2)	4.05	0.4	12	4.3	0.4	6	0.9%	-0.25 [-0.64, 0.14]
Ramezani 2017(1)	4.89	0.61	15	5.86	0.92	5	0.4%	-0.97 [-1.83, -0.11]
Ramezani 2017(2)	5.26	0.77	15	5.86	0.92	5	0.4%	-0.60 [-1.50, 0.30]
Ramezani 2017(3)	5.06	0.63	15	5.86	0.92	5	0.4%	-0.80 [-1.67, 0.07]
Seo 2012	4.66	0.77	10	4.75	0.68	10	0.6%	-0.09 [-0.73, 0.55]
Shaibi 2006	5.21	0.35	11	5.09	0.35	10	1.1%	0.12 [-0.18, 0.42]
Shalitin 2009	4.49	0.4	55	4.55	0.45	55	1.3%	-0.06 [-0.22, 0.10]
Son 2017	4.82	0.52	20	5.22	0.67	20	1.0%	-0.40 [-0.77, -0.03]
Staiano 2017	4.95	1.76	20	5.74	1.78	18	0.3%	-0.79 [-1.92, 0.34]
Staiano 2018	5.01	0.3	22	4.95	0.25	23	1.3%	0.06 [-0.10, 0.22]
Tsang 2009	5.15	0.48	11	4.83	0.39	8	0.9%	0.32 [-0.07, 0.71]
Wen 2008	3.97	0.31	24	5.23	0.69	13	0.9%	-1.26 [-1.66, -0.86]
Wong 2018	4.3	0.77	15	5.4	0.39	15	0.9%	-1.10 [-1.54, -0.66]
Woo 2004	4.4	0.6	41	4.3	0.6	41	1.2%	0.10 [-0.16, 0.36]
Xianbo 2011	4.8	0.4	25	4.6	0.4	17	1.2%	0.20 [-0.05, 0.45]
Xishao 2009	4.18	0.72	34	4.78	1.01	33	0.9%	-0.60 [-1.02, -0.18]
Xishao 2010	4.18	0.72	100	4.78	1.01	100	1.2%	-0.60 [-0.84, -0.36]
Yu 2020	4.6	0.5	99	5.1	0.4	72	1.3%	-0.50 [-0.64, -0.36]
Zehsaz 2016	4.8	0.5	16	4.8	0.5	16	1.0%	0.00 [-0.35, 0.35]
Zhaogang 2021	4.03	0.28	49	4.25	0.54	49	1.3%	-0.22 [-0.39, -0.05]
**Subtotal (95% CI)**			**1823**			**1424**	**63.0%**	**-0.24 [-0.33, -0.15]**
Heterogeneity: Tau² = 0.10; Chi² = 501.04, df = 59 (P < 0.00001); I² = 88%								
Test for overall effect: Z = 5.14 (P < 0.00001)								
**2.2.2 High intensity**								
Abassi 2020(2)	5	0.39	8	5.2	0.21	4	1.0%	-0.20 [-0.54, 0.14]
Benson 2008	5.2	0.4	32	5	0.5	35	1.2%	0.20 [-0.02, 0.42]
Bezerra 2023	4.7	0.34	20	4.64	0.38	21	1.2%	0.06 [-0.16, 0.28]
Boer 2014(1)	4.72	0.39	17	4.72	0.23	7	1.2%	0.00 [-0.25, 0.25]
Davis(1) 2009	4.91	0.48	17	5.07	0.36	21	1.1%	-0.16 [-0.44, 0.12]
DAVIS(2) 2009(1)	4.68	0.37	9	5.11	0.22	5	1.1%	-0.43 [-0.74, -0.12]
DAVIS(2) 2009(2)	4.82	0.34	15	5.11	0.22	5	1.2%	-0.29 [-0.55, -0.03]
Dias 2018(1)	5.13	0.37	19	5.08	0.39	8	1.1%	0.05 [-0.27, 0.37]
Duft 2020	4.55	0.41	18	4.75	0.47	19	1.1%	-0.20 [-0.48, 0.08]
Faria 2020(2)	4.7	0.39	26	4.71	0.39	13	1.2%	-0.01 [-0.27, 0.25]
Gulijianati 2022	4.83	0.36	47	5.46	0.41	46	1.3%	-0.63 [-0.79, -0.47]
Hongling 2012	4.1	0.8	30	4.2	0.4	30	1.1%	-0.10 [-0.42, 0.22]
Ke 2024	5.2	0.4	10	5.6	0.6	10	0.9%	-0.40 [-0.85, 0.05]
Lee 2012(2)	5.27	0.23	16	5.37	0.27	6	1.2%	-0.10 [-0.34, 0.14]
Lee 2013(2)	5.26	0.6	14	5.47	0.21	4	1.0%	-0.21 [-0.59, 0.17]
Lira 2017(1)	4.54	0.53	26	4.4	0.3	16	1.2%	0.14 [-0.11, 0.39]
Lopes 2016	4.52	0.34	17	4.88	0.31	16	1.2%	-0.36 [-0.58, -0.14]
McCormack 2014	4.38	0.18	10	4.24	0.27	8	1.2%	0.14 [-0.08, 0.36]
Meng 2022(1)	4.7	0.3	12	5.4	0.4	6	1.0%	-0.70 [-1.06, -0.34]
Migueles 2023	4.66	0.45	46	4.65	0.45	42	1.3%	0.01 [-0.18, 0.20]
Pena 2022	5.49	0.49	79	5.88	1.37	38	0.9%	-0.39 [-0.84, 0.06]
Racil(1) 2016(1)	4.6	0.4	17	4.6	0.4	7	1.0%	0.00 [-0.35, 0.35]
Racil(2) 2016(1)	4.6	0.4	23	4.8	0.4	9	1.1%	-0.20 [-0.51, 0.11]
Racil(2) 2016(2)	4.1	0.4	26	4.8	0.4	10	1.1%	-0.70 [-0.99, -0.41]
Racil 2013(1)	4.5	0.47	11	4.6	0.32	6	1.0%	-0.10 [-0.48, 0.28]
Racil 2024(1)	4.35	0.4	12	4.3	0.4	5	0.9%	0.05 [-0.37, 0.47]
Salus(2) 2022	5.11	0.37	14	5.19	0.37	14	1.1%	-0.08 [-0.35, 0.19]
Savoye 2007	4.92	0.36	75	4.9	0.48	44	1.3%	0.02 [-0.14, 0.18]
Savoye 2011	4.97	0.27	75	4.88	0.3	44	1.3%	0.09 [-0.02, 0.20]
Tas 2023	5.09	0.49	31	5.05	0.49	6	0.9%	0.04 [-0.39, 0.47]
Tjonna 2009	4.9	0.31	20	5	0.33	22	1.2%	-0.10 [-0.29, 0.09]
Vasconcellos 2016	5.1	0.36	10	5.23	0.46	10	1.0%	-0.13 [-0.49, 0.23]
Wong 2008	4.7	0.4	12	4.8	0.8	12	0.8%	-0.10 [-0.61, 0.41]
Zehsaz 2017	4.8	0.5	16	4.8	0.5	16	1.0%	0.00 [-0.35, 0.35]
**Subtotal (95% CI)**			**830**			**565**	**37.0%**	**-0.13 [-0.22, -0.05]**
Heterogeneity: Tau² = 0.05; Chi² = 123.81, df = 33 (P < 0.00001); I² = 73%								
Test for overall effect: Z = 2.97 (P = 0.003)								
**Total (95% CI)**			**2653**			**1989**	**100.0%**	**-0.20 [-0.26, -0.13]**
Heterogeneity: Tau² = 0.08; Chi² = 637.17, df = 93 (P < 0.00001); I² = 85%								
Test for overall effect: Z = 5.81 (P < 0.00001)								
Test for subgroup differences: Chi² = 2.71, df = 1 (P = 0.10), I² = 63.1%								

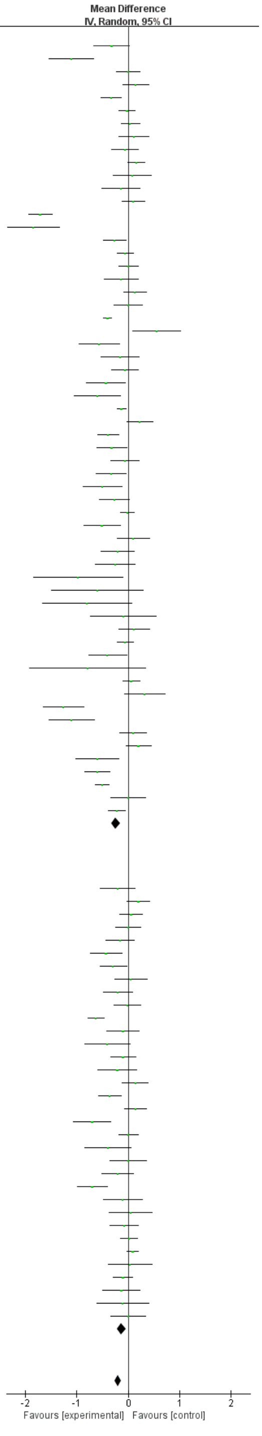

Figure S15.3. Forest plot of subgroup analyses by duration per session

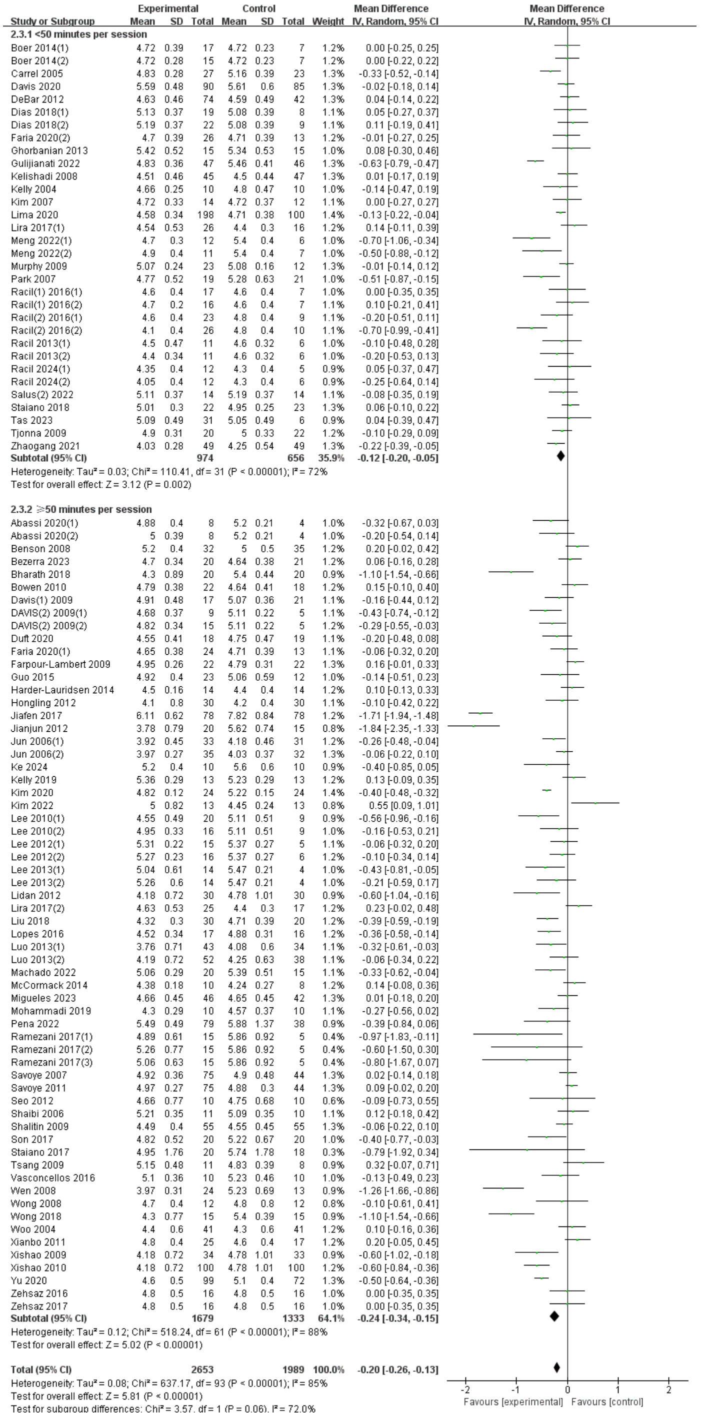

Figure S15.4. Forest plot of subgroup analyses by exercise frequency

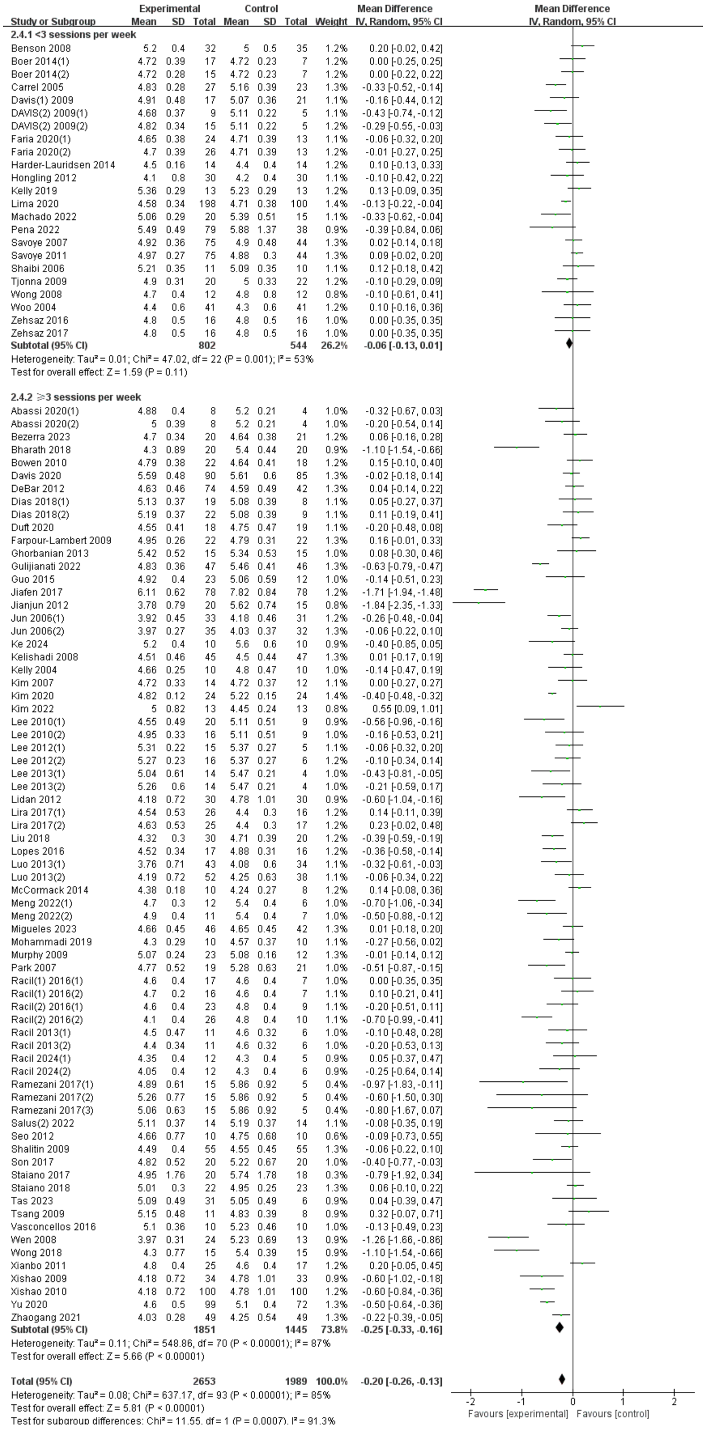

Figure S15.5. Forest plot of subgroup analyses by weekly exercise duration

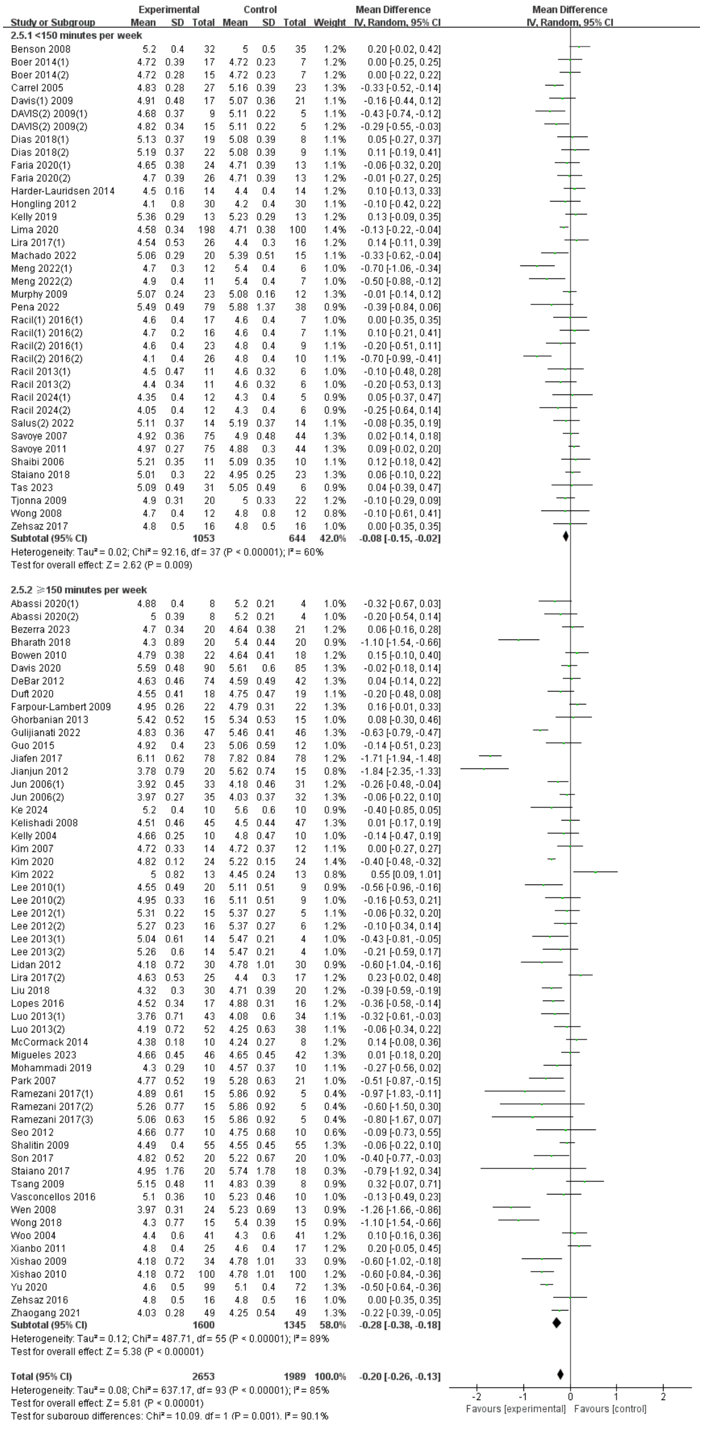

Figure S15.6. Forest plot of subgroup analyses by intervention duration

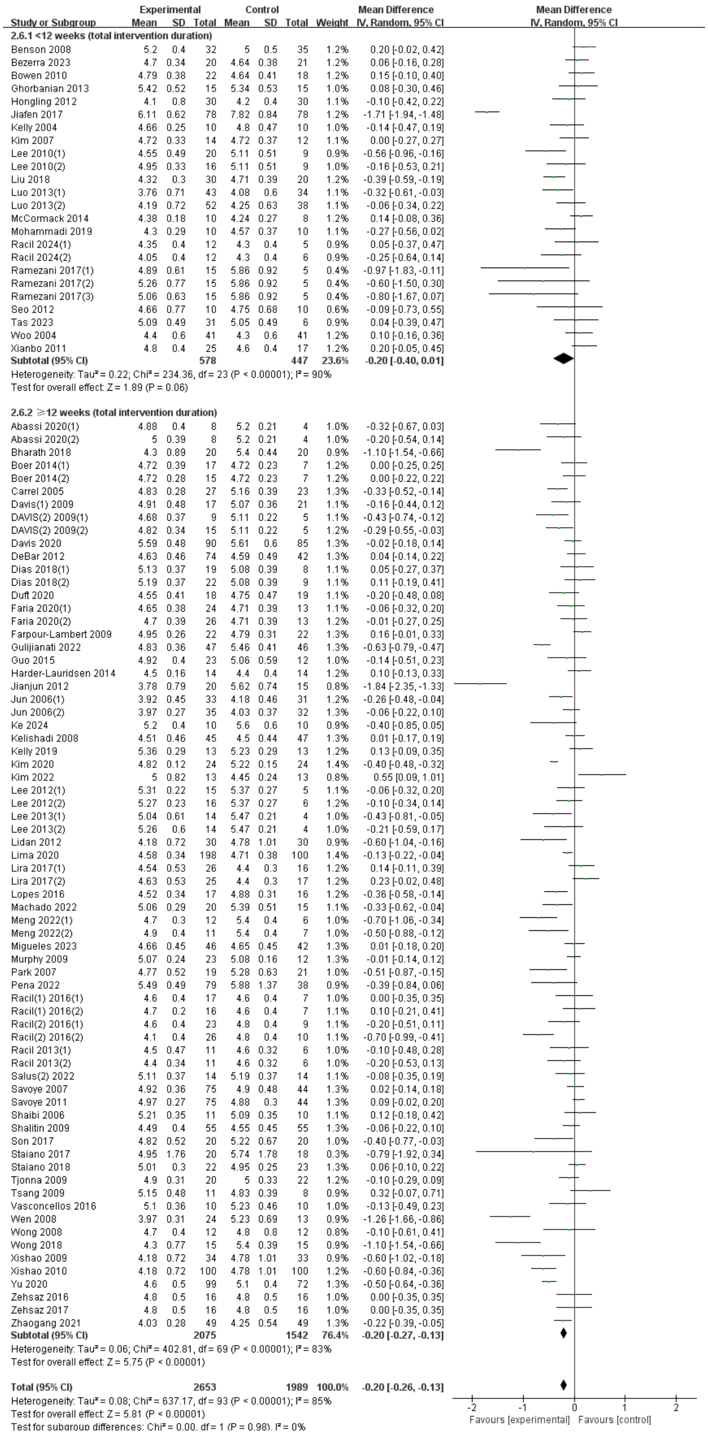

Figure S15.7. Forest plot of subgroup analyses by region

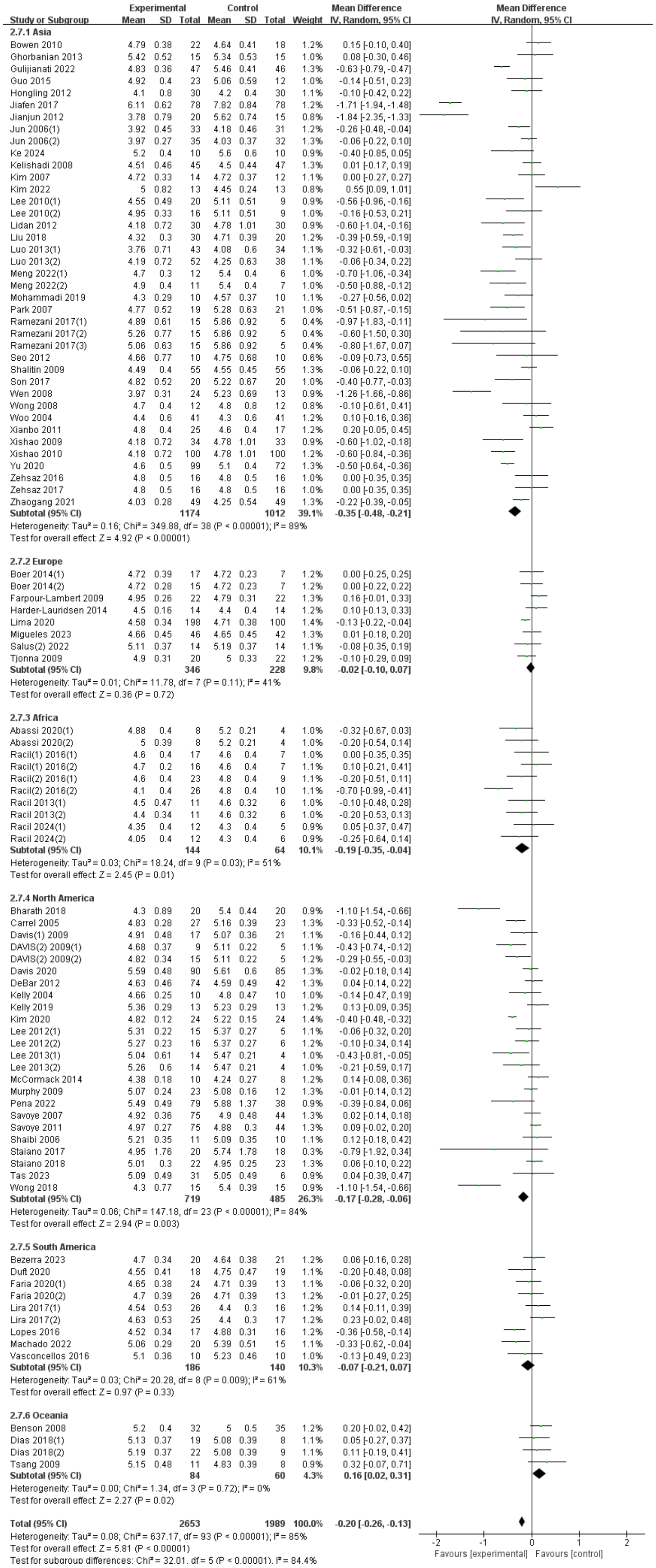

Figure S15.8. Forest plot of subgroup analyses by economic status

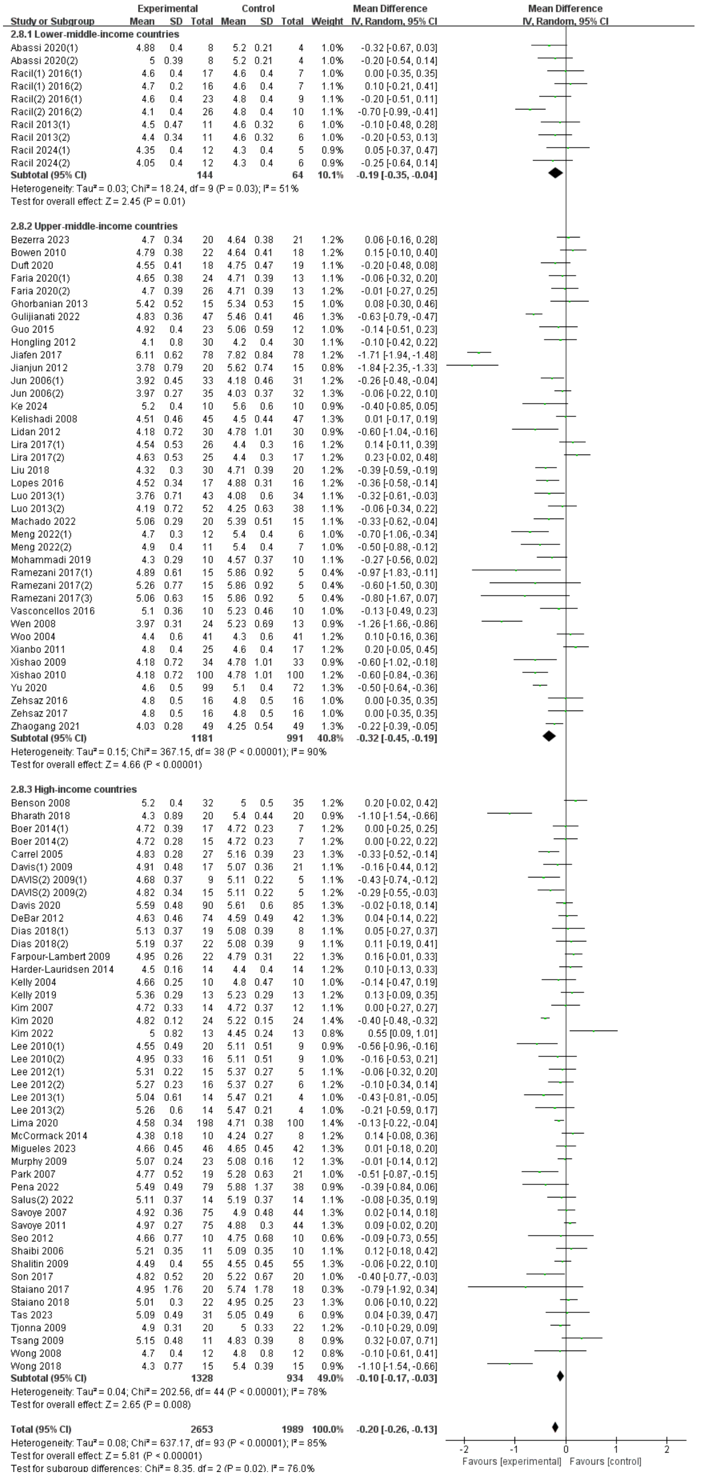

Figure S15.9. Forest plot of subgroup analyses by weight status

Study or Subgroup	Experimental Mean	Experimental SD	Experimental Total	Control Mean	Control SD	Control Total	Weight	Mean Difference IV, Random, 95% CI
**2.9.1 Overweight**								
Bezerra 2023	4.7	0.34	20	4.64	0.38	21	1.5%	0.06 [-0.16, 0.28]
Boer 2014(1)	4.72	0.39	17	4.72	0.23	7	1.5%	0.00 [-0.25, 0.25]
Boer 2014(2)	4.72	0.28	15	4.72	0.23	7	1.5%	0.00 [-0.22, 0.22]
Davis(1) 2009	4.91	0.48	17	5.07	0.36	21	1.4%	-0.16 [-0.44, 0.12]
DAVIS(2) 2009(1)	4.68	0.37	9	5.11	0.22	5	1.4%	-0.43 [-0.74, -0.12]
DAVIS(2) 2009(2)	4.82	0.34	15	5.11	0.22	5	1.4%	-0.29 [-0.55, -0.03]
DeBar 2012	4.63	0.46	74	4.59	0.49	42	1.6%	0.04 [-0.14, 0.22]
Faria 2020(1)	4.65	0.38	24	4.71	0.39	13	1.4%	-0.06 [-0.32, 0.20]
Faria 2020(2)	4.7	0.39	26	4.71	0.39	13	1.4%	-0.01 [-0.27, 0.25]
Gulijianati 2022	4.83	0.36	47	5.46	0.41	46	1.6%	-0.63 [-0.79, -0.47]
Harder-Lauridsen 2014	4.5	0.16	14	4.4	0.4	14	1.5%	0.10 [-0.13, 0.33]
Kelly 2004	4.66	0.25	10	4.8	0.47	10	1.3%	-0.14 [-0.47, 0.19]
Lopes 2016	4.52	0.34	17	4.88	0.31	16	1.5%	-0.36 [-0.58, -0.14]
Mohammadi 2019	4.3	0.29	10	4.57	0.37	10	1.4%	-0.27 [-0.56, 0.02]
Murphy 2009	5.07	0.24	23	5.08	0.16	12	1.6%	-0.01 [-0.14, 0.12]
Shaibi 2006	5.21	0.35	11	5.09	0.35	10	1.4%	0.12 [-0.18, 0.42]
**Subtotal (95% CI)**			**349**			**252**	**23.4%**	**-0.13 [-0.25, -0.01]**
Heterogeneity: Tau² = 0.05; Chi² = 68.53, df = 15 (P < 0.00001); I² = 78%								
Test for overall effect: Z = 2.04 (P = 0.04)								
**2.9.2 Obesity**								
Bharath 2018	4.3	0.89	20	5.4	0.44	20	1.1%	-1.10 [-1.54, -0.66]
Carrel 2005	4.83	0.28	27	5.16	0.39	23	1.5%	-0.33 [-0.52, -0.14]
Dias 2018(1)	5.13	0.37	19	5.08	0.39	8	1.3%	0.05 [-0.27, 0.37]
Dias 2018(2)	5.19	0.37	22	5.08	0.39	9	1.4%	0.11 [-0.19, 0.41]
Farpour-Lambert 2009	4.95	0.26	22	4.79	0.31	22	1.6%	0.16 [-0.01, 0.33]
Hongling 2012	4.1	0.8	30	4.2	0.4	30	1.3%	-0.10 [-0.42, 0.22]
Jiafen 2017	6.11	0.62	78	7.82	0.84	78	1.5%	-1.71 [-1.94, -1.48]
Jianjun 2012	3.78	0.79	20	5.62	0.74	15	1.0%	-1.84 [-2.35, -1.33]
Jun 2006(1)	3.92	0.45	33	4.18	0.46	31	1.5%	-0.26 [-0.48, -0.04]
Jun 2006(2)	3.97	0.27	35	4.03	0.37	32	1.6%	-0.06 [-0.22, 0.10]
Ke 2024	5.2	0.4	10	5.6	0.6	10	1.1%	-0.40 [-0.85, 0.05]
Kelishadi 2008	4.51	0.46	45	4.5	0.44	47	1.6%	0.01 [-0.17, 0.19]
Kelly 2019	5.36	0.29	13	5.23	0.29	13	1.5%	0.13 [-0.09, 0.35]
Kim 2007	4.72	0.33	14	4.72	0.37	12	1.4%	0.00 [-0.27, 0.27]
Kim 2020	4.82	0.12	24	5.22	0.15	24	1.7%	-0.40 [-0.48, -0.32]
Lee 2010(1)	4.55	0.49	20	5.11	0.51	9	1.2%	-0.56 [-0.96, -0.16]
Lee 2010(2)	4.95	0.33	16	5.11	0.51	9	1.2%	-0.16 [-0.53, 0.21]
Lee 2012(1)	5.31	0.22	15	5.37	0.27	5	1.4%	-0.06 [-0.32, 0.20]
Lee 2012(2)	5.27	0.23	16	5.37	0.27	6	1.5%	-0.10 [-0.34, 0.14]
Lee 2013(1)	5.04	0.61	14	5.47	0.21	4	1.2%	-0.43 [-0.81, -0.05]
Lee 2013(2)	5.26	0.6	14	5.47	0.21	4	1.2%	-0.21 [-0.59, 0.17]
Lidan 2012	4.18	0.72	30	4.78	1.01	30	1.1%	-0.60 [-1.04, -0.16]
Lira 2017(1)	4.54	0.53	26	4.4	0.3	16	1.5%	0.14 [-0.11, 0.39]
Lira 2017(2)	4.63	0.53	25	4.4	0.3	17	1.5%	0.23 [-0.02, 0.48]
Liu 2018	4.32	0.3	30	4.71	0.39	20	1.5%	-0.39 [-0.59, -0.19]
Luo 2013(1)	3.76	0.71	43	4.08	0.6	34	1.4%	-0.32 [-0.61, -0.03]
Luo 2013(2)	4.19	0.72	52	4.25	0.63	38	1.4%	-0.06 [-0.34, 0.22]
McCormack 2014	4.38	0.18	10	4.24	0.27	8	1.5%	0.14 [-0.08, 0.36]
Meng 2022(1)	4.7	0.3	12	5.4	0.4	6	1.3%	-0.70 [-1.06, -0.34]
Meng 2022(2)	4.9	0.4	11	5.4	0.4	7	1.2%	-0.50 [-0.88, -0.12]
Park 2007	4.77	0.52	19	5.28	0.63	21	1.3%	-0.51 [-0.87, -0.15]
Pena 2022	5.49	0.49	79	5.88	1.37	38	1.1%	-0.39 [-0.84, 0.06]
Racil(1) 2016(1)	4.6	0.4	17	4.6	0.4	7	1.3%	0.00 [-0.35, 0.35]
Racil(1) 2016(2)	4.7	0.2	16	4.6	0.4	7	1.3%	0.10 [-0.21, 0.41]
Racil(2) 2016(1)	4.6	0.4	23	4.8	0.4	9	1.4%	-0.20 [-0.51, 0.11]
Racil(2) 2016(2)	4.1	0.4	26	4.8	0.4	10	1.4%	-0.70 [-0.99, -0.41]
Racil 2013(1)	4.5	0.47	11	4.6	0.32	6	1.2%	-0.10 [-0.48, 0.28]
Racil 2013(2)	4.4	0.34	11	4.6	0.32	6	1.3%	-0.20 [-0.53, 0.13]
Racil 2024(1)	4.35	0.4	12	4.3	0.4	5	1.2%	0.05 [-0.37, 0.47]
Racil 2024(2)	4.05	0.4	12	4.3	0.4	6	1.2%	-0.25 [-0.64, 0.14]
Ramezani 2017(1)	4.89	0.61	15	5.86	0.92	5	0.6%	-0.97 [-1.83, -0.11]
Ramezani 2017(2)	5.26	0.77	15	5.86	0.92	5	0.5%	-0.60 [-1.50, 0.30]
Ramezani 2017(3)	5.06	0.63	15	5.86	0.92	5	0.6%	-0.80 [-1.67, 0.07]
Salus(2) 2022	5.11	0.37	14	5.19	0.37	14	1.4%	-0.08 [-0.35, 0.19]
Savoye 2007	4.92	0.36	75	4.9	0.48	44	1.6%	0.02 [-0.14, 0.18]
Savoye 2011	4.97	0.27	75	4.88	0.3	44	1.6%	0.09 [-0.02, 0.20]
Seo 2012	4.66	0.77	10	4.75	0.68	10	0.8%	-0.09 [-0.73, 0.55]
Shalitin 2009	4.49	0.4	55	4.55	0.45	55	1.6%	-0.06 [-0.22, 0.10]
Son 2017	4.82	0.52	20	5.22	0.67	20	1.2%	-0.40 [-0.77, -0.03]
Tas 2023	5.09	0.49	31	5.05	0.49	6	1.1%	0.04 [-0.39, 0.47]
Vasconcellos 2016	5.1	0.36	10	5.23	0.46	10	1.3%	-0.13 [-0.49, 0.23]
Wen 2008	3.97	0.31	24	5.23	0.69	13	1.2%	-1.26 [-1.66, -0.86]
Wong 2008	4.7	0.4	12	4.8	0.8	12	1.0%	-0.10 [-0.61, 0.41]
Wong 2018	4.3	0.77	15	5.4	0.39	15	1.1%	-1.10 [-1.54, -0.66]
Xishao 2009	4.18	0.72	34	4.78	1.01	33	1.2%	-0.60 [-1.02, -0.18]
Xishao 2010	4.18	0.72	100	4.78	1.01	100	1.5%	-0.60 [-0.84, -0.36]
Yu 2020	4.6	0.5	99	5.1	0.4	72	1.6%	-0.50 [-0.64, -0.36]
Zehsaz 2016	4.8	0.5	16	4.8	0.5	16	1.3%	0.00 [-0.35, 0.35]
Zhaogang 2021	4.03	0.28	49	4.25	0.54	49	1.6%	-0.22 [-0.39, -0.05]
**Subtotal (95% CI)**			**1656**			**1230**	**76.6%**	**-0.29 [-0.39, -0.19]**
Heterogeneity: Tau² = 0.11; Chi² = 485.80, df = 58 (P < 0.00001); I² = 88%								
Test for overall effect: Z = 5.85 (P < 0.00001)								
**Total (95% CI)**			**2005**			**1482**	**100.0%**	**-0.25 [-0.33, -0.17]**
Heterogeneity: Tau² = 0.10; Chi² = 562.60, df = 74 (P < 0.00001); I² = 87%								
Test for overall effect: Z = 6.14 (P < 0.00001)								
Test for subgroup differences: Chi² = 4.16, df = 1 (P = 0.04), I² = 76.0%								

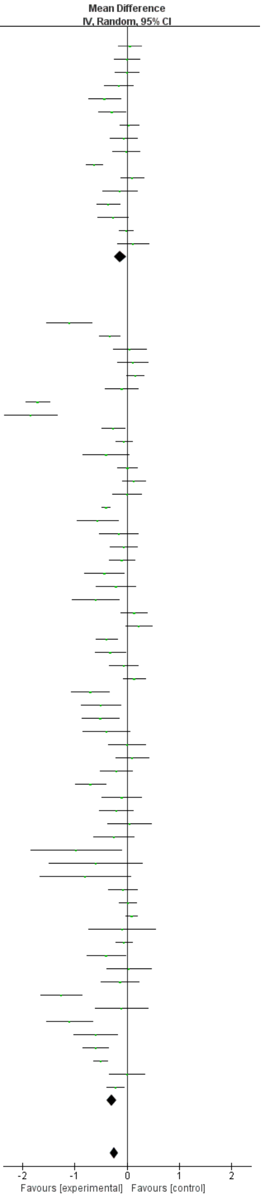

Figure S15.10. Forest plot of subgroup analyses by sex

Study or Subgroup	Experimental Mean	Experimental SD	Experimental Total	Control Mean	Control SD	Control Total	Weight	Mean Difference IV, Random, 95% CI
**2.10.1 Boys**								
Ghorbanian 2013	5.42	0.52	15	5.34	0.53	15	2.2%	0.08 [-0.30, 0.46]
Hongling 2012	4.1	0.8	30	4.2	0.4	30	2.4%	-0.10 [-0.42, 0.22]
Jianjun 2012	3.78	0.79	20	5.62	0.74	15	1.7%	-1.84 [-2.35, -1.33]
Kelly 2019	5.36	0.29	13	5.23	0.29	13	2.8%	0.13 [-0.09, 0.35]
Kim 2007	4.72	0.33	14	4.72	0.37	12	2.6%	0.00 [-0.27, 0.27]
Lee 2012(1)	5.31	0.22	15	5.37	0.27	5	2.7%	-0.06 [-0.32, 0.20]
Lee 2012(2)	5.27	0.23	16	5.37	0.27	6	2.8%	-0.10 [-0.34, 0.14]
Luo 2013(2)	4.19	0.72	52	4.25	0.63	38	2.6%	-0.06 [-0.34, 0.22]
Meng 2022(1)	4.7	0.3	12	5.4	0.4	6	2.2%	-0.70 [-1.06, -0.34]
Meng 2022(2)	4.9	0.4	11	5.4	0.4	7	2.2%	-0.50 [-0.88, -0.12]
Ramezani 2017(1)	4.89	0.61	15	5.86	0.92	5	0.9%	-0.97 [-1.83, -0.11]
Ramezani 2017(2)	5.26	0.77	15	5.86	0.92	5	0.8%	-0.60 [-1.50, 0.30]
Ramezani 2017(3)	5.06	0.63	15	5.86	0.92	5	0.9%	-0.80 [-1.67, 0.07]
Salus(2) 2022	5.11	0.37	14	5.19	0.37	14	2.6%	-0.08 [-0.35, 0.19]
Seo 2012	4.66	0.77	10	4.75	0.68	10	1.3%	-0.09 [-0.73, 0.55]
Shaibi 2006	5.21	0.35	11	5.09	0.35	10	2.5%	0.12 [-0.18, 0.42]
Wong 2008	4.7	0.4	12	4.8	0.8	12	1.7%	-0.10 [-0.61, 0.41]
Zehsaz 2016	4.8	0.5	16	4.8	0.5	16	2.3%	0.00 [-0.35, 0.35]
Zehsaz 2017	4.8	0.5	16	4.8	0.5	16	2.3%	0.00 [-0.35, 0.35]
**Subtotal (95% CI)**			**322**			**240**	**39.5%**	**-0.22 [-0.39, -0.05]**
Heterogeneity: Tau² = 0.10; Chi² = 75.14, df = 18 (P < 0.00001); I² = 76%								
Test for overall effect: Z = 2.58 (P = 0.010)								
**2.10.2 Girls**								
Abassi 2020(1)	4.88	0.4	8	5.2	0.21	4	2.3%	-0.32 [-0.67, 0.03]
Abassi 2020(2)	5	0.39	8	5.2	0.21	4	2.3%	-0.20 [-0.54, 0.14]
Bharath 2018	4.3	0.89	20	5.4	0.44	20	1.9%	-1.10 [-1.54, -0.66]
DAVIS(2) 2009(1)	4.68	0.37	9	5.11	0.22	5	2.5%	-0.43 [-0.74, -0.12]
DAVIS(2) 2009(2)	4.82	0.34	15	5.11	0.22	5	2.7%	-0.29 [-0.55, -0.03]
DeBar 2012	4.63	0.46	74	4.59	0.49	42	3.0%	0.04 [-0.14, 0.22]
Kim 2020	4.82	0.12	24	5.22	0.15	24	3.3%	-0.40 [-0.48, -0.32]
Kim 2022	5	0.82	13	4.45	0.24	13	1.8%	0.55 [0.09, 1.01]
Lee 2013(1)	5.04	0.61	14	5.47	0.21	4	2.2%	-0.43 [-0.81, -0.05]
Lee 2013(2)	5.26	0.6	14	5.47	0.21	4	2.2%	-0.21 [-0.59, 0.17]
Liu 2018	4.32	0.3	30	4.71	0.39	20	2.9%	-0.39 [-0.59, -0.19]
Lopes 2016	4.52	0.34	17	4.88	0.31	16	2.9%	-0.36 [-0.58, -0.14]
Luo 2013(1)	3.76	0.71	43	4.08	0.6	34	2.5%	-0.32 [-0.61, -0.03]
Mohammadi 2019	4.3	0.29	10	4.57	0.37	10	2.5%	-0.27 [-0.56, 0.02]
Park 2007	4.77	0.52	19	5.28	0.63	21	2.3%	-0.51 [-0.87, -0.15]
Racil(1) 2016(1)	4.6	0.4	17	4.6	0.4	7	2.3%	0.00 [-0.35, 0.35]
Racil(1) 2016(2)	4.7	0.2	16	4.6	0.4	7	2.5%	0.10 [-0.21, 0.41]
Racil(2) 2016(1)	4.6	0.4	23	4.8	0.4	9	2.5%	-0.20 [-0.51, 0.11]
Racil(2) 2016(2)	4.1	0.4	26	4.8	0.4	10	2.5%	-0.70 [-0.99, -0.41]
Racil 2013(1)	4.5	0.47	11	4.6	0.32	6	2.2%	-0.10 [-0.48, 0.28]
Racil 2013(2)	4.4	0.34	11	4.6	0.32	6	2.4%	-0.20 [-0.53, 0.13]
Racil 2024(1)	4.35	0.4	12	4.3	0.4	5	2.0%	0.05 [-0.37, 0.47]
Racil 2024(2)	4.05	0.4	12	4.3	0.4	6	2.1%	-0.25 [-0.64, 0.14]
Son 2017	4.82	0.52	20	5.22	0.67	20	2.2%	-0.40 [-0.77, -0.03]
Staiano 2017	4.95	1.76	20	5.74	1.78	18	0.6%	-0.79 [-1.92, 0.34]
Wong 2018	4.3	0.77	15	5.4	0.39	15	1.9%	-1.10 [-1.54, -0.66]
**Subtotal (95% CI)**			**501**			**335**	**60.5%**	**-0.30 [-0.40, -0.20]**
Heterogeneity: Tau² = 0.04; Chi² = 83.82, df = 25 (P < 0.00001); I² = 70%								
Test for overall effect: Z = 5.65 (P < 0.00001)								
**Total (95% CI)**			**823**			**575**	**100.0%**	**-0.26 [-0.36, -0.17]**
Heterogeneity: Tau² = 0.07; Chi² = 177.20, df = 44 (P < 0.00001); I² = 75%								
Test for overall effect: Z = 5.60 (P < 0.00001)								
Test for subgroup differences: Chi² = 0.56, df = 1 (P = 0.45), I² = 0%								

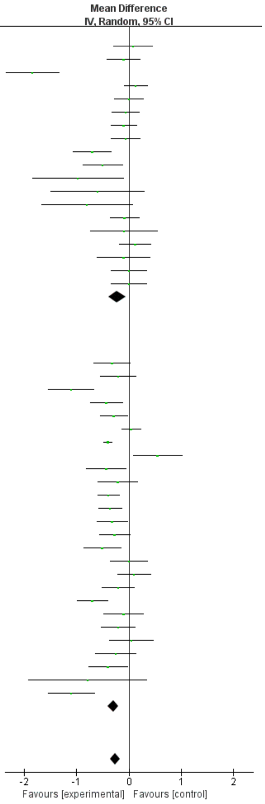

Figure S16. Forest plots of subgroup analyses for HbA1c in children and adolescents with overweight or obesity

Figure S16.1. Forest plot of subgroup analyses by type of exercise

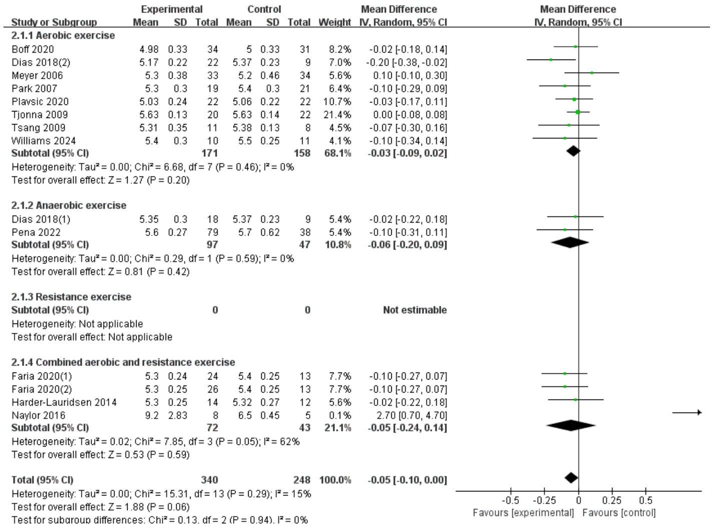

Figure S16.2. Forest plot of subgroup analyses by exercise intensity

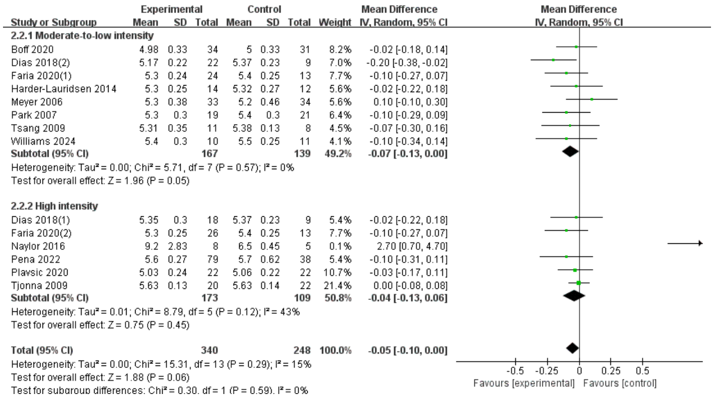

Figure S16.3. Forest plot of subgroup analyses by duration per session

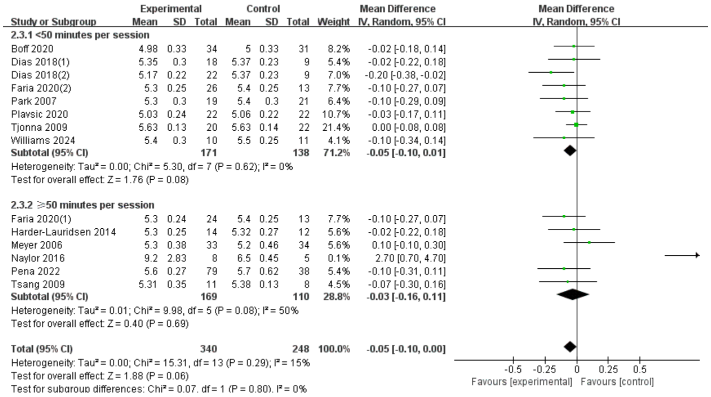

Figure S16.4. Forest plot of subgroup analyses by exercise frequency

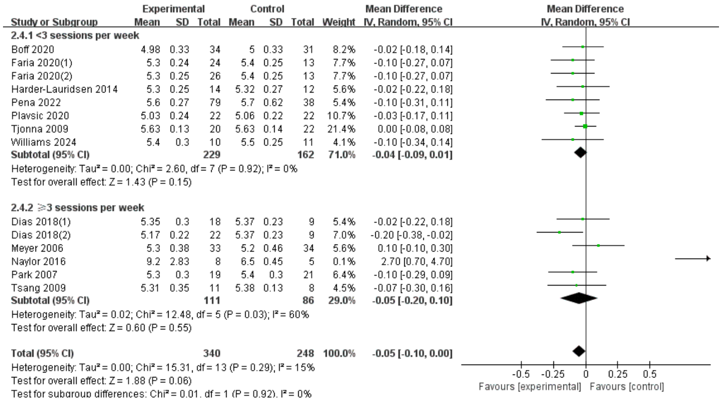

Figure S16.5. Forest plot of subgroup analyses by weekly exercise duration

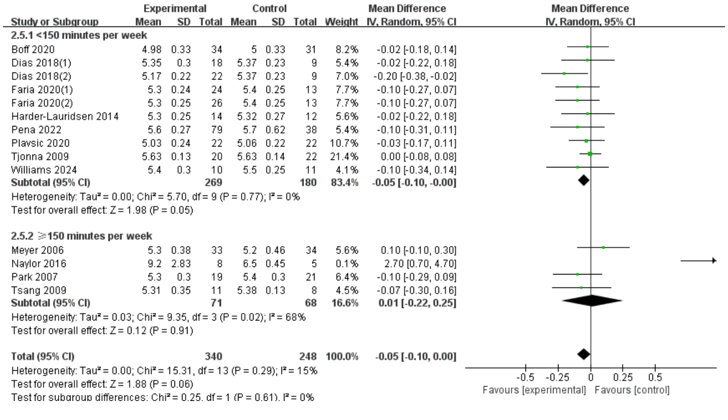

Figure S16.6. Forest plot of subgroup analyses by intervention duration

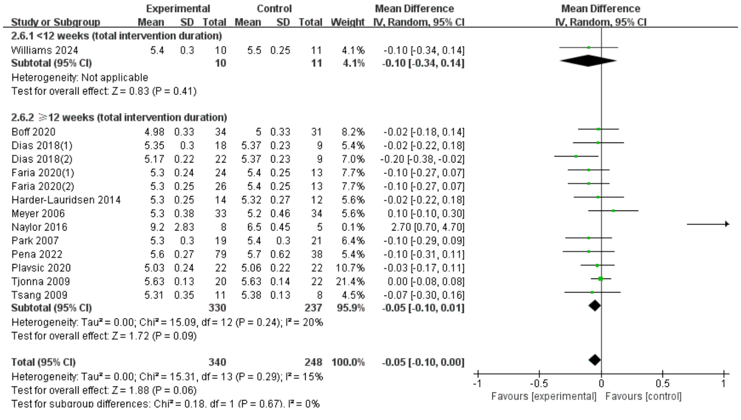

Figure S16.7. Forest plot of subgroup analyses by region

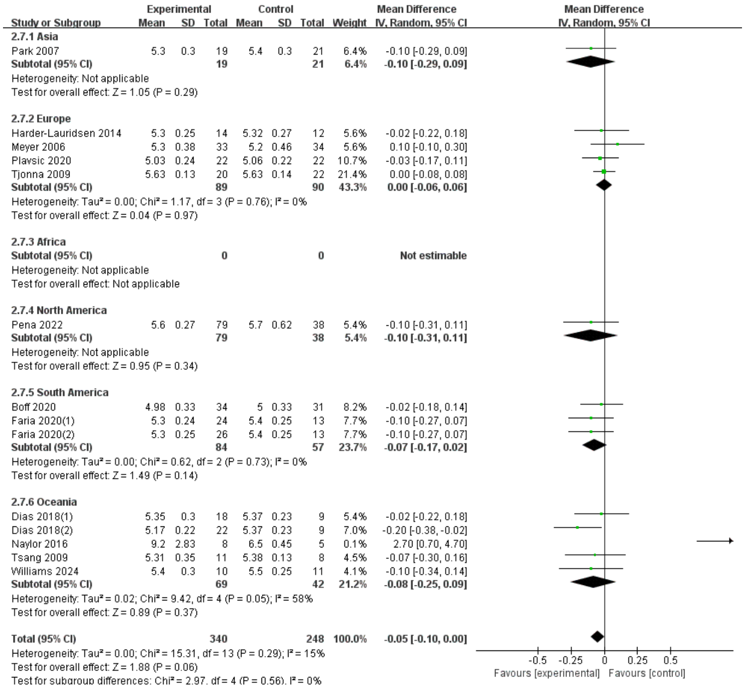

Figure S16.8. Forest plot of subgroup analyses by economic status

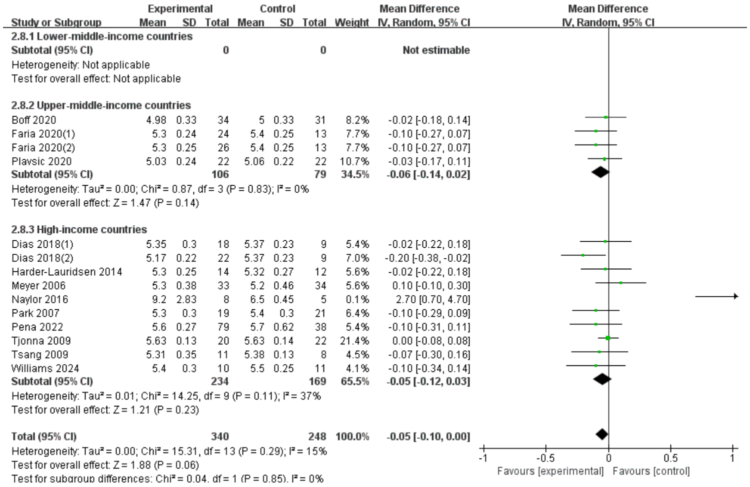

Figure S16.9. Forest plot of subgroup analyses by weight status

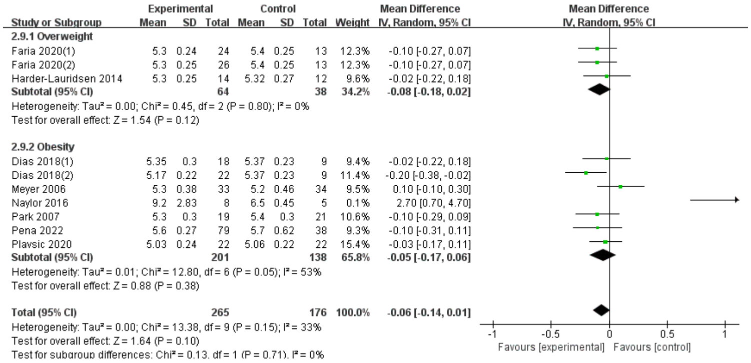

Figure S16.10. Forest plot of subgroup analyses by sex

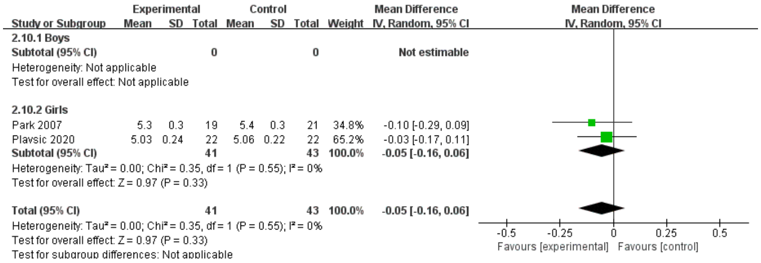

Figure S17. Forest plots of subgroup analyses for FINS in children and adolescents with overweight or obesity

Figure S17.1. Forest plot of subgroup analyses by type of exercise

Study or Subgroup	Experimental Mean	Experimental SD	Experimental Total	Control Mean	Control SD	Control Total	Weight	Mean Difference IV, Random, 95% CI
**2.1.1 Aerobic exercise**								
Abassi 2020(1)	9.46	2.08	8	12.3	5.31	4	1.5%	-2.84 [-8.24, 2.56]
Bowen 2010	15.05	4.7	22	16.29	12.2	18	1.4%	-1.24 [-7.21, 4.73]
Carrel 2005	21.9	14	27	25.8	10	23	1.2%	-3.90 [-10.58, 2.78]
Davis 2020	23.4	15.67	90	19.4	16.08	85	1.8%	4.00 [-0.71, 8.71]
Gulijianati 2022	5.18	1.23	47	7.89	1.29	46	3.7%	-2.71 [-3.22, -2.20]
Jianjun 2012	13.34	4.53	20	28.92	6.44	15	2.2%	-15.58 [-19.40, -11.76]
Kelly 2004	11.12	5	10	14.12	7.43	10	1.5%	-3.00 [-8.55, 2.55]
Kim 2007	8.9	4.98	14	10.8	4.5	12	2.3%	-1.90 [-5.55, 1.75]
Kim 2020	9.1	1.2	24	13.7	2.2	24	3.6%	-4.60 [-5.60, -3.60]
Lee 2012(1)	20.1	69.38	15	40	69.05	5	0.0%	-19.90 [-89.87, 50.07]
Lee 2013(1)	20.57	14.71	14	31.05	13.23	4	0.3%	-10.48 [-25.56, 4.60]
Lira 2017(1)	27.75	15.35	26	27.4	12.57	16	0.8%	0.35 [-8.18, 8.88]
Lira 2017(2)	25.48	10.3	25	27.4	12.57	17	1.1%	-1.92 [-9.13, 5.29]
Liu 2018	8.95	4.39	30	16.78	4.85	20	2.8%	-7.83 [-10.47, -5.19]
Luo 2013(1)	6.22	7.37	43	12.83	5.11	34	2.7%	-6.61 [-9.40, -3.82]
Luo 2013(2)	8.73	7.97	52	16.27	5.27	38	2.8%	-7.54 [-10.28, -4.80]
Machado 2022	14.7	7.6	20	29.9	6	15	1.9%	-15.20 [-19.71, -10.69]
Meng 2022(2)	6.8	3	11	12.3	2.4	7	2.9%	-5.50 [-8.01, -2.99]
Meyer 2006	11.16	4.61	33	14.6	7.65	34	2.6%	-3.44 [-6.45, -0.43]
Park 2007	8.3	3	19	14.4	8.2	21	2.2%	-6.10 [-9.86, -2.34]
Racil(1) 2016(2)	17.3	1.5	16	19.2	2	7	3.3%	-1.90 [-3.55, -0.25]
Racil 2013(2)	17.9	1.45	11	18.8	1.95	6	3.3%	-0.90 [-2.68, 0.88]
Savoye 2007	17.9	27.11	75	29.5	16.2	44	0.9%	-11.60 [-19.38, -3.82]
Savoye 2011	18.4	4.72	75	27.4	17.73	44	1.6%	-9.00 [-14.35, -3.65]
Seo 2012	6.63	8.66	10	9.93	25.11	10	0.3%	-3.30 [-19.76, 13.16]
Staiano 2017	26.1	13.37	20	23.47	7.63	18	1.1%	2.63 [-4.21, 9.47]
Tjonna 2009	22.02	12.82	20	23.33	13.6	22	0.9%	-1.31 [-9.30, 6.68]
Tsang 2009	19.95	12.57	11	20.89	5	8	0.9%	-0.94 [-9.14, 7.26]
**Subtotal (95% CI)**			**788**			**607**	**51.6%**	**-4.66 [-5.99, -3.33]**
Heterogeneity: Tau² = 6.89; Chi² = 142.69, df = 27 (P < 0.00001); I² = 81%								
Test for overall effect: Z = 6.88 (P < 0.00001)								
**2.1.2 Anaerobic exercise**								
Abassi 2020(2)	10.6	4.1	8	12.3	5.31	4	1.4%	-1.70 [-7.63, 4.23]
Meng 2022(1)	7.5	2.8	12	12.3	2.4	6	2.9%	-4.80 [-7.29, -2.31]
Pena 2022	19	8.83	78	21	12.33	38	1.9%	-2.00 [-6.38, 2.38]
Racil(1) 2016(1)	16.3	0.84	17	19.2	2	7	3.4%	-2.90 [-4.43, -1.37]
Racil(2) 2016(1)	15.6	1.2	23	18.6	1.6	9	3.6%	-3.00 [-4.15, -1.85]
Racil(2) 2016(2)	14.6	1	26	18.6	1.6	10	3.6%	-4.00 [-5.06, -2.94]
Racil 2013(1)	15.5	1.19	11	18.8	1.95	6	3.3%	-3.30 [-5.01, -1.59]
Salus(2) 2022	20.64	6.73	14	31.8	24.69	14	0.4%	-11.16 [-24.57, 2.25]
Tas 2023	35.85	22.41	31	28.86	22.41	6	0.2%	6.99 [-12.60, 26.58]
Vasconcellos 2016	31.5	9.2	10	37.1	18.6	10	0.4%	-5.60 [-18.46, 7.26]
**Subtotal (95% CI)**			**230**			**110**	**21.0%**	**-3.44 [-4.06, -2.83]**
Heterogeneity: Tau² = 0.00; Chi² = 6.49, df = 9 (P = 0.69); I² = 0%								
Test for overall effect: Z = 11.00 (P < 0.00001)								
**2.1.3 Resistance exercise**								
Benson 2008	15.33	9.6	32	16.03	20.85	35	1.0%	-0.70 [-8.37, 6.97]
Davis(1) 2009	27.8	12.5	17	24.3	14.5	21	0.8%	3.50 [-5.09, 12.09]
DAVIS(2) 2009(1)	28.3	8.01	9	28	17.5	5	0.3%	0.30 [-15.91, 16.51]
Lee 2012(2)	17	66.94	16	40	69.05	6	0.0%	-23.00 [-87.25, 41.25]
Lee 2013(2)	44.15	17.49	14	31.05	13.23	4	0.3%	13.10 [-2.78, 28.98]
Shaibi 2006	11.5	8.29	11	19.1	8.54	10	1.1%	-7.60 [-14.81, -0.39]
**Subtotal (95% CI)**			**99**			**81**	**3.4%**	**-0.28 [-6.03, 5.47]**
Heterogeneity: Tau² = 16.95; Chi² = 7.78, df = 5 (P = 0.17); I² = 36%								
Test for overall effect: Z = 0.10 (P = 0.92)								
**2.1.4 Combined aerobic and resistance exercise**								
Bharath 2018	10.3	9.39	20	27	10.73	20	1.3%	-16.70 [-22.95, -10.45]
DAVIS(2) 2009(2)	28.6	7.49	15	28	17.5	5	0.3%	0.60 [-15.20, 16.40]
Faria 2020(1)	10.8	4.14	24	11	4.21	13	2.7%	-0.20 [-3.03, 2.63]
Faria 2020(2)	11.4	4.33	26	11	4.21	13	2.7%	0.40 [-2.43, 3.23]
Farpour-Lambert 2009	16.13	6.77	22	13.5	5.27	22	2.3%	2.63 [-0.96, 6.22]
Lopes 2016	14.7	7.4	17	16.8	6.5	16	1.8%	-2.10 [-6.85, 2.65]
McCormack 2014	27.67	21.32	10	21.17	17.33	8	0.2%	6.50 [-11.36, 24.36]
Migueles 2023	13.36	6.32	39	13.91	6.33	37	2.7%	-0.55 [-3.40, 2.30]
Shalitin 2009	8.88	9.12	55	11.62	10.01	55	2.3%	-2.74 [-6.32, 0.84]
Son 2017	9.1	1.34	20	12.5	5.37	20	2.9%	-3.40 [-5.83, -0.97]
Wen 2008	22.08	12.64	24	51.23	26.09	13	0.3%	-29.15 [-44.21, -14.09]
Wong 2018	10.3	8.13	15	27	9.3	15	1.3%	-16.70 [-22.95, -10.45]
Zehsaz 2016	12.3	2.6	16	16.6	3.1	16	3.2%	-4.30 [-6.28, -2.32]
**Subtotal (95% CI)**			**303**			**253**	**24.0%**	**-4.03 [-6.77, -1.28]**
Heterogeneity: Tau² = 17.29; Chi² = 72.75, df = 12 (P < 0.00001); I² = 84%								
Test for overall effect: Z = 2.88 (P = 0.004)								
**Total (95% CI)**			**1420**			**1051**	**100.0%**	**-3.93 [-4.80, -3.06]**
Heterogeneity: Tau² = 5.23; Chi² = 233.37, df = 56 (P < 0.00001); I² = 76%								
Test for overall effect: Z = 8.83 (P < 0.00001)								
Test for subgroup differences: Chi² = 4.05, df = 3 (P = 0.26), I² = 25.9%								

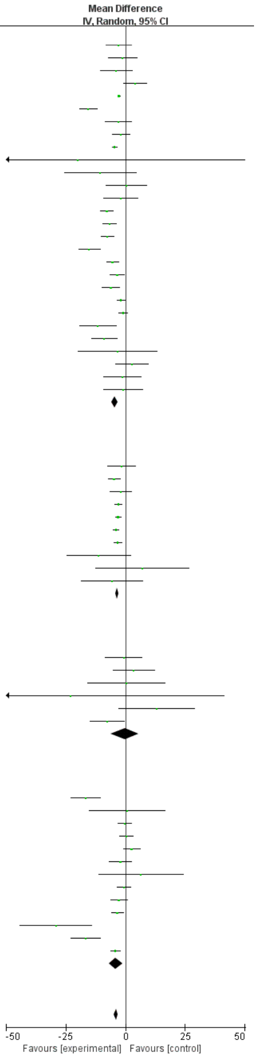

Figure S17.2. Forest plot of subgroup analyses by exercise intensity

Study or Subgroup	Experimental Mean	Experimental SD	Experimental Total	Control Mean	Control SD	Control Total	Weight	Mean Difference IV, Random, 95% CI
**2.2.1 Moderate-to-low intensity**								
Abassi 2020(1)	9.46	2.08	8	12.3	5.31	4	1.5%	-2.84 [-8.24, 2.56]
Bharath 2018	10.3	9.39	20	27	10.73	20	1.3%	-16.70 [-22.95, -10.45]
Bowen 2010	15.05	4.7	22	16.29	12.2	18	1.4%	-1.24 [-7.21, 4.73]
Carrel 2005	21.9	14	27	25.8	10	23	1.2%	-3.90 [-10.58, 2.78]
Davis 2020	23.4	15.67	90	19.4	16.08	85	1.8%	4.00 [-0.71, 8.71]
Faria 2020(1)	10.8	4.14	24	11	4.21	13	2.7%	-0.20 [-3.03, 2.63]
Farpour-Lambert 2009	16.13	6.77	22	13.5	5.27	22	2.3%	2.63 [-0.96, 6.22]
Jianjun 2012	13.34	4.53	20	28.92	6.44	15	2.2%	-15.58 [-19.40, -11.76]
Kelly 2004	11.12	5	10	14.12	7.43	10	1.5%	-3.00 [-8.55, 2.55]
Kim 2007	8.9	4.98	14	10.8	4.5	12	2.3%	-1.90 [-5.55, 1.75]
Kim 2020	9.1	1.2	24	13.7	2.2	24	3.6%	-4.60 [-5.60, -3.60]
Lee 2012(1)	20.1	69.38	15	40	69.05	5	0.0%	-19.90 [-89.87, 50.07]
Lee 2013(1)	20.57	14.71	14	31.05	13.23	4	0.3%	-10.48 [-25.56, 4.60]
Lira 2017(2)	25.48	10.3	25	27.4	12.57	17	1.1%	-1.92 [-9.13, 5.29]
Liu 2018	8.95	4.39	30	16.78	4.85	20	2.8%	-7.83 [-10.47, -5.19]
Luo 2013(1)	6.22	7.37	43	12.83	5.11	34	2.7%	-6.61 [-9.40, -3.82]
Luo 2013(2)	8.73	7.97	52	16.27	5.27	38	2.8%	-7.54 [-10.28, -4.80]
Machado 2022	14.7	7.6	20	29.9	6	15	1.9%	-15.20 [-19.71, -10.69]
Meng 2022(2)	6.8	3	11	12.3	2.4	7	2.9%	-5.50 [-8.01, -2.99]
Meyer 2006	11.16	4.61	33	14.6	7.65	34	2.6%	-3.44 [-6.45, -0.43]
Park 2007	8.3	3	19	14.4	8.2	21	2.2%	-6.10 [-9.86, -2.34]
Racil(1) 2016(2)	17.3	1.5	16	19.2	2	7	3.3%	-1.90 [-3.55, -0.25]
Racil 2013(2)	17.9	1.45	11	18.8	1.95	6	3.3%	-0.90 [-2.68, 0.88]
Seo 2012	6.63	8.66	10	9.93	25.11	10	0.3%	-3.30 [-19.76, 13.16]
Shaibi 2006	11.5	8.29	11	19.1	8.54	10	1.1%	-7.60 [-14.81, -0.39]
Shalitin 2009	8.88	9.12	55	11.62	10.01	55	2.3%	-2.74 [-6.32, 0.84]
Son 2017	9.1	1.34	20	12.5	5.37	20	2.9%	-3.40 [-5.83, -0.97]
Staiano 2017	26.1	13.37	20	23.47	7.63	18	1.1%	2.63 [-4.21, 9.47]
Tsang 2009	19.95	12.57	11	20.89	5	8	0.9%	-0.94 [-9.14, 7.26]
Wen 2008	22.08	12.64	24	51.23	26.09	13	0.3%	-29.15 [-44.21, -14.09]
Wong 2018	10.3	8.13	15	27	9.3	15	1.3%	-16.70 [-22.95, -10.45]
Zehsaz 2016	12.3	2.6	16	16.6	3.1	16	3.2%	-4.30 [-6.28, -2.32]
**Subtotal (95% CI)**			**752**			**619**	**61.0%**	**-4.92 [-6.42, -3.42]**
Heterogeneity: Tau² = 12.18; Chi² = 181.46, df = 31 (P < 0.00001); I² = 83%								
Test for overall effect: Z = 6.44 (P < 0.00001)								
**2.2.2 High intensity**								
Abassi 2020(2)	10.6	4.1	8	12.3	5.31	4	1.4%	-1.70 [-7.63, 4.23]
Benson 2008	15.33	9.6	32	16.03	20.85	35	1.0%	-0.70 [-8.37, 6.97]
Davis(1) 2009	27.8	12.5	17	24.3	14.5	21	0.8%	3.50 [-5.09, 12.09]
DAVIS(2) 2009(1)	28.3	8.01	9	28	17.5	5	0.3%	0.30 [-15.91, 16.51]
DAVIS(2) 2009(2)	28.6	7.49	15	28	17.5	5	0.3%	0.60 [-15.20, 16.40]
Faria 2020(2)	11.4	4.33	26	11	4.21	13	2.7%	0.40 [-2.43, 3.23]
Gulijianati 2022	5.18	1.23	47	7.89	1.29	46	3.7%	-2.71 [-3.22, -2.20]
Lee 2012(2)	17	66.94	16	40	69.05	6	0.0%	-23.00 [-87.25, 41.25]
Lee 2013(2)	44.15	17.49	14	31.05	13.23	4	0.3%	13.10 [-2.78, 28.98]
Lira 2017(1)	27.75	15.35	26	27.4	12.57	16	0.8%	0.35 [-8.18, 8.88]
Lopes 2016	14.7	7.4	17	16.8	6.5	16	1.8%	-2.10 [-6.85, 2.65]
McCormack 2014	27.67	21.32	10	21.17	17.33	8	0.2%	6.50 [-11.36, 24.36]
Meng 2022(1)	7.5	2.8	12	12.3	2.4	6	2.9%	-4.80 [-7.29, -2.31]
Migueles 2023	13.36	6.32	39	13.91	6.33	37	2.7%	-0.55 [-3.40, 2.30]
Pena 2022	19	8.83	78	21	12.33	38	1.9%	-2.00 [-6.38, 2.38]
Racil(1) 2016(1)	16.3	0.84	17	19.2	2	7	3.4%	-2.90 [-4.43, -1.37]
Racil(2) 2016(1)	15.6	1.2	23	18.6	1.6	9	3.6%	-3.00 [-4.15, -1.85]
Racil(2) 2016(2)	14.6	1	26	18.6	1.6	10	3.6%	-4.00 [-5.06, -2.94]
Racil 2013(1)	15.5	1.19	11	18.8	1.95	6	3.3%	-3.30 [-5.01, -1.59]
Salus(2) 2022	20.64	6.73	14	31.8	24.69	14	0.4%	-11.16 [-24.57, 2.25]
Savoye 2007	17.9	27.11	75	29.5	16.2	44	0.9%	-11.60 [-19.38, -3.82]
Savoye 2011	18.4	4.72	75	27.4	17.73	44	1.6%	-9.00 [-14.35, -3.65]
Tas 2023	35.85	22.41	31	28.86	22.41	6	0.2%	6.99 [-12.60, 26.58]
Tjonna 2009	22.02	12.82	20	23.33	13.6	22	0.9%	-1.31 [-9.30, 6.68]
Vasconcellos 2016	31.5	9.2	10	37.1	18.6	10	0.4%	-5.60 [-18.46, 7.26]
**Subtotal (95% CI)**			**668**			**432**	**39.0%**	**-2.88 [-3.64, -2.12]**
Heterogeneity: Tau² = 0.73; Chi² = 36.64, df = 24 (P = 0.05); I² = 34%								
Test for overall effect: Z = 7.44 (P < 0.00001)								
**Total (95% CI)**			**1420**			**1051**	**100.0%**	**-3.93 [-4.80, -3.06]**
Heterogeneity: Tau² = 5.23; Chi² = 233.37, df = 56 (P < 0.00001); I² = 76%								
Test for overall effect: Z = 8.83 (P < 0.00001)								
Test for subgroup differences: Chi² = 5.67, df = 1 (P = 0.02), I² = 82.3%								

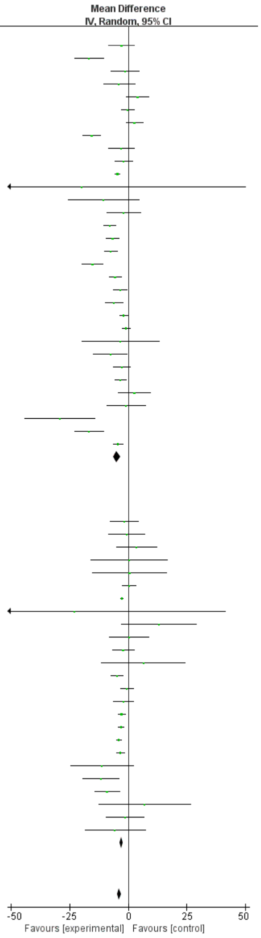

Figure S17.3. Forest plot of subgroup analyses by duration per session

Study or Subgroup	Experimental Mean	Experimental SD	Experimental Total	Control Mean	Control SD	Control Total	Weight	Mean Difference IV, Random, 95% CI
**2.3.1 <50 minutes per session**								
Carrel 2005	21.9	14	27	25.8	10	23	1.2%	-3.90 [-10.58, 2.78]
Davis 2020	23.4	15.67	90	19.4	16.08	85	1.8%	4.00 [-0.71, 8.71]
Faria 2020(2)	11.4	4.33	26	11	4.21	13	2.7%	0.40 [-2.43, 3.23]
Gulijianati 2022	5.18	1.23	47	7.89	1.29	46	3.7%	-2.71 [-3.22, -2.20]
Kelly 2004	11.12	5	10	14.12	7.43	10	1.5%	-3.00 [-8.55, 2.55]
Kim 2007	8.9	4.98	14	10.8	4.5	12	2.3%	-1.90 [-5.55, 1.75]
Lira 2017(1)	27.75	15.35	26	27.4	12.57	16	0.8%	0.35 [-8.18, 8.88]
Meng 2022(1)	7.5	2.8	12	12.3	2.4	6	2.9%	-4.80 [-7.29, -2.31]
Meng 2022(2)	6.8	3	11	12.3	2.4	7	2.9%	-5.50 [-8.01, -2.99]
Park 2007	8.3	3	19	14.4	8.2	21	2.2%	-6.10 [-9.86, -2.34]
Racil(1) 2016(1)	16.3	0.84	17	19.2	2	7	3.4%	-2.90 [-4.43, -1.37]
Racil(1) 2016(2)	17.3	1.5	16	19.2	2	7	3.3%	-1.90 [-3.55, -0.25]
Racil(2) 2016(1)	15.6	1.2	23	18.6	1.6	9	3.6%	-3.00 [-4.15, -1.85]
Racil(2) 2016(2)	14.6	1	26	18.6	1.6	10	3.6%	-4.00 [-5.06, -2.94]
Racil 2013(1)	15.5	1.19	11	18.8	1.95	6	3.3%	-3.30 [-5.01, -1.59]
Racil 2013(2)	17.9	1.45	11	18.8	1.95	6	3.3%	-0.90 [-2.68, 0.88]
Salus(2) 2022	20.64	6.73	14	31.8	24.69	14	0.4%	-11.16 [-24.57, 2.25]
Tas 2023	35.85	22.41	31	28.86	22.41	6	0.2%	6.99 [-12.60, 26.58]
Tjonna 2009	22.02	12.82	20	23.33	13.6	22	0.9%	-1.31 [-9.30, 6.68]
**Subtotal (95% CI)**			**451**			**326**	**43.9%**	**-2.80 [-3.53, -2.07]**
Heterogeneity: Tau² = 0.92; Chi² = 37.19, df = 18 (P = 0.005); I² = 52%								
Test for overall effect: Z = 7.47 (P < 0.00001)								
**2.3.2 ≥50 minutes per session**								
Abassi 2020(1)	9.46	2.08	8	12.3	5.31	4	1.5%	-2.84 [-8.24, 2.56]
Abassi 2020(2)	10.6	4.1	8	12.3	5.31	4	1.4%	-1.70 [-7.63, 4.23]
Benson 2008	15.33	9.6	32	16.03	20.85	35	1.0%	-0.70 [-8.37, 6.97]
Bharath 2018	10.3	9.39	20	27	10.73	20	1.3%	-16.70 [-22.95, -10.45]
Bowen 2010	15.05	4.7	22	16.29	12.2	18	1.4%	-1.24 [-7.21, 4.73]
Davis(1) 2009	27.8	12.5	17	24.3	14.5	21	0.8%	3.50 [-5.09, 12.09]
DAVIS(2) 2009(1)	28.3	8.01	9	28	17.5	5	0.3%	0.30 [-15.91, 16.51]
DAVIS(2) 2009(2)	28.6	7.49	15	28	17.5	5	0.3%	0.60 [-15.20, 16.40]
Faria 2020(1)	10.8	4.14	24	11	4.21	13	2.7%	-0.20 [-3.03, 2.63]
Farpour-Lambert 2009	16.13	6.77	22	13.5	5.27	22	2.3%	2.63 [-0.96, 6.22]
Jianjun 2012	13.34	4.53	20	28.92	6.44	15	2.2%	-15.58 [-19.40, -11.76]
Kim 2020	9.1	1.2	24	13.7	2.2	24	3.6%	-4.60 [-5.60, -3.60]
Lee 2012(1)	20.1	69.38	15	40	69.05	5	0.0%	-19.90 [-89.87, 50.07]
Lee 2012(2)	17	66.94	16	40	69.05	6	0.0%	-23.00 [-87.25, 41.25]
Lee 2013(1)	20.57	14.71	14	31.05	13.23	4	0.3%	-10.48 [-25.56, 4.60]
Lee 2013(2)	44.15	17.49	14	31.05	13.23	4	0.3%	13.10 [-2.78, 28.98]
Lira 2017(2)	25.48	10.3	25	27.4	12.57	17	1.1%	-1.92 [-9.13, 5.29]
Liu 2018	8.95	4.39	30	16.78	4.85	20	2.8%	-7.83 [-10.47, -5.19]
Lopes 2016	14.7	7.4	17	16.8	6.5	16	1.8%	-2.10 [-6.85, 2.65]
Luo 2013(1)	6.22	7.37	43	12.83	5.11	34	2.7%	-6.61 [-9.40, -3.82]
Luo 2013(2)	8.73	7.97	52	16.27	5.27	38	2.8%	-7.54 [-10.28, -4.80]
Machado 2022	14.7	7.6	20	29.9	6	15	1.9%	-15.20 [-19.71, -10.69]
McCormack 2014	27.67	21.32	10	21.17	17.33	8	0.2%	6.50 [-11.36, 24.36]
Meyer 2006	11.16	4.61	33	14.6	7.65	34	2.6%	-3.44 [-6.45, -0.43]
Migueles 2023	13.36	6.32	39	13.91	6.33	37	2.7%	-0.55 [-3.40, 2.30]
Pena 2022	19	8.83	78	21	12.33	38	1.9%	-2.00 [-6.38, 2.38]
Savoye 2007	17.9	27.11	75	29.5	16.2	44	0.9%	-11.60 [-19.38, -3.82]
Savoye 2011	18.4	4.72	75	27.4	17.73	44	1.6%	-9.00 [-14.35, -3.65]
Seo 2012	6.63	8.66	10	9.93	25.11	10	0.3%	-3.30 [-19.76, 13.16]
Shaibi 2006	11.5	8.29	11	19.1	8.54	10	1.1%	-7.60 [-14.81, -0.39]
Shalitin 2009	8.88	9.12	55	11.62	10.01	55	2.3%	-2.74 [-6.32, 0.84]
Son 2017	9.1	1.34	20	12.5	5.37	20	2.9%	-3.40 [-5.83, -0.97]
Staiano 2017	26.1	13.37	20	23.47	7.63	18	1.1%	2.63 [-4.21, 9.47]
Tsang 2009	19.95	12.57	11	20.89	5	8	0.9%	-0.94 [-9.14, 7.26]
Vasconcellos 2016	31.5	9.2	10	37.1	18.6	10	0.4%	-5.60 [-18.46, 7.26]
Wen 2008	22.08	12.64	24	51.23	26.09	13	0.3%	-29.15 [-44.21, -14.09]
Wong 2018	10.3	8.13	15	27	9.3	15	1.3%	-16.70 [-22.95, -10.45]
Zehsaz 2016	12.3	2.6	16	16.6	3.1	16	3.2%	-4.30 [-6.28, -2.32]
**Subtotal (95% CI)**			**969**			**725**	**56.1%**	**-4.94 [-6.54, -3.35]**
Heterogeneity: Tau² = 13.94; Chi² = 168.99, df = 37 (P < 0.00001); I² = 78%								
Test for overall effect: Z = 6.08 (P < 0.00001)								
**Total (95% CI)**			**1420**			**1051**	**100.0%**	**-3.93 [-4.80, -3.06]**
Heterogeneity: Tau² = 5.23; Chi² = 233.37, df = 56 (P < 0.00001); I² = 76%								
Test for overall effect: Z = 8.83 (P < 0.00001)								
Test for subgroup differences: Chi² = 5.74, df = 1 (P = 0.02), I² = 82.6%								

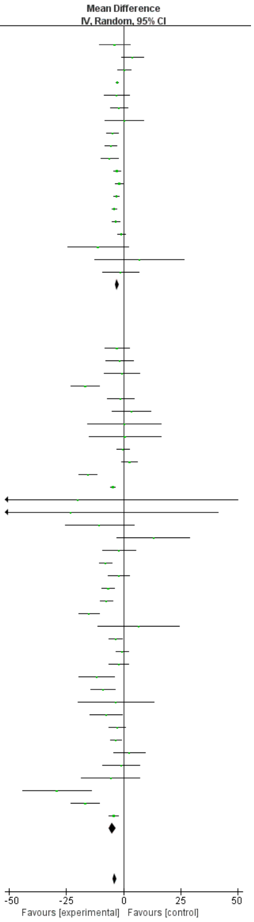

Figure S17.4. Forest plot of subgroup analyses by exercise frequency

Study or Subgroup	Experimental Mean	Experimental SD	Experimental Total	Control Mean	Control SD	Control Total	Weight	Mean Difference IV, Random, 95% CI
**2.4.1 <3 sessions per week**								
Benson 2008	15.33	9.6	32	16.03	20.85	35	1.0%	-0.70 [-8.37, 6.97]
Carrel 2005	21.9	14	27	25.8	10	23	1.2%	-3.90 [-10.58, 2.78]
Davis(1) 2009	27.8	12.5	17	24.3	14.5	21	0.8%	3.50 [-5.09, 12.09]
DAVIS(2) 2009(1)	28.3	8.01	9	28	17.5	5	0.3%	0.30 [-15.91, 16.51]
DAVIS(2) 2009(2)	28.6	7.49	15	28	17.5	5	0.3%	0.60 [-15.20, 16.40]
Faria 2020(1)	10.8	4.14	24	11	4.21	13	2.7%	-0.20 [-3.03, 2.63]
Faria 2020(2)	11.4	4.33	26	11	4.21	13	2.7%	0.40 [-2.43, 3.23]
Machado 2022	14.7	7.6	20	29.9	6	15	1.9%	-15.20 [-19.71, -10.69]
Pena 2022	19	8.83	78	21	12.33	38	1.9%	-2.00 [-6.38, 2.38]
Savoye 2007	17.9	27.11	75	29.5	16.2	44	0.9%	-11.60 [-19.38, -3.82]
Savoye 2011	18.4	4.72	75	27.4	17.73	44	1.6%	-9.00 [-14.35, -3.65]
Shaibi 2006	11.5	8.29	11	19.1	8.54	10	1.1%	-7.60 [-14.81, -0.39]
Tjonna 2009	22.02	12.82	20	23.33	13.6	22	0.9%	-1.31 [-9.30, 6.68]
Zehsaz 2016	12.3	2.6	16	16.6	3.1	16	3.2%	-4.30 [-6.28, -2.32]
**Subtotal (95% CI)**			**445**			**304**	**20.4%**	**-4.11 [-6.87, -1.35]**
Heterogeneity: Tau² = 16.86; Chi² = 52.81, df = 13 (P < 0.00001); I² = 75%								
Test for overall effect: Z = 2.92 (P = 0.004)								
**2.4.2 ≥3 sessions per week**								
Abassi 2020(1)	9.46	2.08	8	12.3	5.31	4	1.5%	-2.84 [-8.24, 2.56]
Abassi 2020(2)	10.6	4.1	8	12.3	5.31	4	1.4%	-1.70 [-7.63, 4.23]
Bharath 2018	10.3	9.39	20	27	10.73	20	1.3%	-16.70 [-22.95, -10.45]
Bowen 2010	15.05	4.7	22	16.29	12.2	18	1.4%	-1.24 [-7.21, 4.73]
Davis 2020	23.4	15.67	90	19.4	16.08	85	1.8%	4.00 [-0.71, 8.71]
Farpour-Lambert 2009	16.13	6.77	22	13.5	5.27	22	2.3%	2.63 [-0.96, 6.22]
Gulijianati 2022	5.18	1.23	47	7.89	1.29	46	3.7%	-2.71 [-3.22, -2.20]
Jianjun 2012	13.34	4.53	20	28.92	6.44	15	2.2%	-15.58 [-19.40, -11.76]
Kelly 2004	11.12	5	10	14.12	7.43	10	1.5%	-3.00 [-8.55, 2.55]
Kim 2007	8.9	4.98	14	10.8	4.5	12	2.3%	-1.90 [-5.55, 1.75]
Kim 2020	9.1	1.2	24	13.7	2.2	24	3.6%	-4.60 [-5.60, -3.60]
Lee 2012(1)	20.1	69.38	15	40	69.05	5	0.0%	-19.90 [-89.87, 50.07]
Lee 2012(2)	17	66.94	16	40	69.05	6	0.0%	-23.00 [-87.25, 41.25]
Lee 2013(1)	20.57	14.71	14	31.05	13.23	4	0.3%	-10.48 [-25.56, 4.60]
Lee 2013(2)	44.15	17.49	14	31.05	13.23	4	0.3%	13.10 [-2.78, 28.98]
Lira 2017(1)	27.75	15.35	26	27.4	12.57	16	0.8%	0.35 [-8.18, 8.88]
Lira 2017(2)	25.48	10.3	25	27.4	12.57	17	1.1%	-1.92 [-9.13, 5.29]
Liu 2018	8.95	4.39	30	16.78	4.85	20	2.8%	-7.83 [-10.47, -5.19]
Lopes 2016	14.7	7.4	17	16.8	6.5	16	1.8%	-2.10 [-6.85, 2.65]
Luo 2013(1)	6.22	7.37	43	12.83	5.11	34	2.7%	-6.61 [-9.40, -3.82]
Luo 2013(2)	8.73	7.97	52	16.27	5.27	38	2.8%	-7.54 [-10.28, -4.80]
McCormack 2014	27.67	21.32	10	21.17	17.33	8	0.2%	6.50 [-11.36, 24.36]
Meng 2022(1)	7.5	2.8	12	12.3	2.4	6	2.9%	-4.80 [-7.29, -2.31]
Meng 2022(2)	6.8	3	11	12.3	2.4	7	2.9%	-5.50 [-8.01, -2.99]
Meyer 2006	11.16	4.61	33	14.6	7.65	34	2.6%	-3.44 [-6.45, -0.43]
Migueles 2023	13.36	6.32	39	13.91	6.33	37	2.7%	-0.55 [-3.40, 2.30]
Park 2007	8.3	3	19	14.4	8.2	21	2.2%	-6.10 [-9.86, -2.34]
Racil(1) 2016(1)	16.3	0.84	17	19.2	2	7	3.4%	-2.90 [-4.43, -1.37]
Racil(1) 2016(2)	17.3	1.5	16	19.2	2	7	3.3%	-1.90 [-3.55, -0.25]
Racil(2) 2016(1)	15.6	1.2	23	18.6	1.6	9	3.6%	-3.00 [-4.15, -1.85]
Racil(2) 2016(2)	14.6	1	26	18.6	1.6	10	3.6%	-4.00 [-5.06, -2.94]
Racil 2013(1)	15.5	1.19	11	18.8	1.95	6	3.3%	-3.30 [-5.01, -1.59]
Racil 2013(2)	17.9	1.45	11	18.8	1.95	6	3.3%	-0.90 [-2.68, 0.88]
Salus(2) 2022	20.64	6.73	14	31.8	24.69	14	0.4%	-11.16 [-24.57, 2.25]
Seo 2012	6.63	8.66	10	9.93	25.11	10	0.3%	-3.30 [-19.76, 13.16]
Shalitin 2009	8.88	9.12	55	11.62	10.01	55	2.3%	-2.74 [-6.32, 0.84]
Son 2017	9.1	1.34	20	12.5	5.37	20	2.9%	-3.40 [-5.83, -0.97]
Staiano 2017	26.1	13.37	20	23.47	7.63	18	1.1%	2.63 [-4.21, 9.47]
Tas 2023	35.85	22.41	31	28.86	22.41	6	0.2%	6.99 [-12.60, 26.58]
Tsang 2009	19.95	12.57	11	20.89	5	8	0.9%	-0.94 [-9.14, 7.26]
Vasconcellos 2016	31.5	9.2	10	37.1	18.6	10	0.4%	-5.60 [-18.46, 7.26]
Wen 2008	22.08	12.64	24	51.23	26.09	13	0.3%	-29.15 [-44.21, -14.09]
Wong 2018	10.3	8.13	15	27	9.3	15	1.3%	-16.70 [-22.95, -10.45]
**Subtotal (95% CI)**			**975**			**747**	**79.6%**	**-3.89 [-4.82, -2.96]**
Heterogeneity: Tau² = 4.54; Chi² = 180.49, df = 42 (P < 0.00001); I² = 77%								
Test for overall effect: Z = 8.19 (P < 0.00001)								
**Total (95% CI)**			**1420**			**1051**	**100.0%**	**-3.93 [-4.80, -3.06]**
Heterogeneity: Tau² = 5.23; Chi² = 233.37, df = 56 (P < 0.00001); I² = 76%								
Test for overall effect: Z = 8.83 (P < 0.00001)								
Test for subgroup differences: Chi² = 0.02, df = 1 (P = 0.89), I² = 0%								

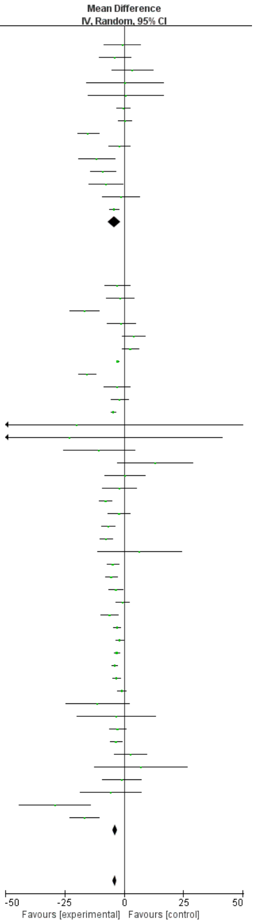

Figure S17.5. Forest plot of subgroup analyses by weekly exercise duration

Study or Subgroup	Experimental Mean	Experimental SD	Experimental Total	Control Mean	Control SD	Control Total	Weight	Mean Difference IV, Random, 95% CI
**2.5.1 <150 minutes per week**								
Benson 2008	15.33	9.6	32	16.03	20.85	35	1.0%	-0.70 [-8.37, 6.97]
Carrel 2005	21.9	14	27	25.8	10	23	1.2%	-3.90 [-10.58, 2.78]
Davis(1) 2009	27.8	12.5	17	24.3	14.5	21	0.8%	3.50 [-5.09, 12.09]
DAVIS(2) 2009(1)	28.3	8.01	9	28	17.5	5	0.3%	0.30 [-15.91, 16.51]
DAVIS(2) 2009(2)	28.6	7.49	15	28	17.5	5	0.3%	0.60 [-15.20, 16.40]
Faria 2020(1)	10.8	4.14	24	11	4.21	13	2.7%	-0.20 [-3.03, 2.63]
Faria 2020(2)	11.4	4.33	26	11	4.21	13	2.7%	0.40 [-2.43, 3.23]
Lira 2017(1)	27.75	15.35	26	27.4	12.57	16	0.8%	0.35 [-8.18, 8.88]
Machado 2022	14.7	7.6	20	29.9	6	15	1.9%	-15.20 [-19.71, -10.69]
Meng 2022(1)	7.5	2.8	12	12.3	2.4	6	2.9%	-4.80 [-7.29, -2.31]
Meng 2022(2)	6.8	3	11	12.3	2.4	7	2.9%	-5.50 [-8.01, -2.99]
Pena 2022	19	8.83	78	21	12.33	38	1.9%	-2.00 [-6.38, 2.38]
Racil(1) 2016(1)	16.3	0.84	17	19.2	2	7	3.4%	-2.90 [-4.43, -1.37]
Racil(1) 2016(2)	17.3	1.5	16	19.2	2	7	3.3%	-1.90 [-3.55, -0.25]
Racil(2) 2016(1)	15.6	1.2	23	18.6	1.6	9	3.6%	-3.00 [-4.15, -1.85]
Racil(2) 2016(2)	14.6	1	26	18.6	1.6	10	3.6%	-4.00 [-5.06, -2.94]
Racil 2013(1)	15.5	1.19	11	18.8	1.95	6	3.3%	-3.30 [-5.01, -1.59]
Racil 2013(2)	17.9	1.45	11	18.8	1.95	6	3.3%	-0.90 [-2.68, 0.88]
Salus(2) 2022	20.64	6.73	14	31.8	24.69	14	0.4%	-11.16 [-24.57, 2.25]
Savoye 2007	17.9	27.11	75	29.5	16.2	44	0.9%	-11.60 [-19.38, -3.82]
Savoye 2011	18.4	4.72	75	27.4	17.73	44	1.6%	-9.00 [-14.35, -3.65]
Shaibi 2006	11.5	8.29	11	19.1	8.54	10	1.1%	-7.60 [-14.81, -0.39]
Tas 2023	35.85	22.41	31	28.86	22.41	6	0.2%	6.99 [-12.60, 26.58]
Tjonna 2009	22.02	12.82	20	23.33	13.6	22	0.9%	-1.31 [-9.30, 6.68]
**Subtotal (95% CI)**			**627**			**382**	**44.8%**	**-3.42 [-4.57, -2.26]**
Heterogeneity: Tau² = 3.65; Chi² = 70.97, df = 23 (P < 0.00001); I² = 68%								
Test for overall effect: Z = 5.80 (P < 0.00001)								
**2.5.2 ⩾150 minutes per week**								
Abassi 2020(1)	9.46	2.08	8	12.3	5.31	4	1.5%	-2.84 [-8.24, 2.56]
Abassi 2020(2)	10.6	4.1	8	12.3	5.31	4	1.4%	-1.70 [-7.63, 4.23]
Bharath 2018	10.3	9.39	20	27	10.73	20	1.3%	-16.70 [-22.95, -10.45]
Bowen 2010	15.05	4.7	22	16.29	12.2	18	1.4%	-1.24 [-7.21, 4.73]
Davis 2020	23.4	15.67	90	19.4	16.08	85	1.8%	4.00 [-0.71, 8.71]
Farpour-Lambert 2009	16.13	6.77	22	13.5	5.27	22	2.3%	2.63 [-0.96, 6.22]
Gulijianati 2022	5.18	1.23	47	7.89	1.29	46	3.7%	-2.71 [-3.22, -2.20]
Jianjun 2012	13.34	4.53	20	28.92	6.44	15	2.2%	-15.58 [-19.40, -11.76]
Kelly 2004	11.12	5	10	14.12	7.43	10	1.5%	-3.00 [-8.55, 2.55]
Kim 2007	8.9	4.98	14	10.8	4.5	12	2.3%	-1.90 [-5.55, 1.75]
Kim 2020	9.1	1.2	24	13.7	2.2	24	3.6%	-4.60 [-5.60, -3.60]
Lee 2012(1)	20.1	69.38	15	40	69.05	5	0.0%	-19.90 [-89.87, 50.07]
Lee 2012(2)	17	66.94	16	40	69.05	6	0.0%	-23.00 [-87.25, 41.25]
Lee 2013(1)	20.57	14.71	14	31.05	13.23	4	0.3%	-10.48 [-25.56, 4.60]
Lee 2013(2)	44.15	17.49	14	31.05	13.23	4	0.3%	13.10 [-2.78, 28.98]
Lira 2017(2)	25.48	10.3	25	27.4	12.57	17	1.1%	-1.92 [-9.13, 5.29]
Liu 2018	8.95	4.39	30	16.78	4.85	20	2.8%	-7.83 [-10.47, -5.19]
Lopes 2016	14.7	7.4	17	16.8	6.5	16	1.8%	-2.10 [-6.85, 2.65]
Luo 2013(1)	6.22	7.37	43	12.83	5.11	34	2.7%	-6.61 [-9.40, -3.82]
Luo 2013(2)	8.73	7.97	52	16.27	5.27	38	2.8%	-7.54 [-10.28, -4.80]
McCormack 2014	27.67	21.32	10	21.17	17.33	8	0.2%	6.50 [-11.36, 24.36]
Meyer 2006	11.16	4.61	33	14.6	7.65	34	2.6%	-3.44 [-6.45, -0.43]
Migueles 2023	13.36	6.32	39	13.91	6.33	37	2.7%	-0.55 [-3.40, 2.30]
Park 2007	8.3	3	19	14.4	8.2	21	2.2%	-6.10 [-9.86, -2.34]
Seo 2012	6.63	8.66	10	9.93	25.11	10	0.3%	-3.30 [-19.76, 13.16]
Shalitin 2009	8.88	9.12	55	11.62	10.01	55	2.3%	-2.74 [-6.32, 0.84]
Son 2017	9.1	1.34	20	12.5	5.37	20	2.9%	-3.40 [-5.83, -0.97]
Staiano 2017	26.1	13.37	20	23.47	7.63	18	1.1%	2.63 [-4.21, 9.47]
Tsang 2009	19.95	12.57	11	20.89	5	8	0.9%	-0.94 [-9.14, 7.26]
Vasconcellos 2016	31.5	9.2	10	37.1	18.6	10	0.4%	-5.60 [-18.46, 7.26]
Wen 2008	22.08	12.64	24	51.23	26.09	13	0.3%	-29.15 [-44.21, -14.09]
Wong 2018	10.3	8.13	15	27	9.3	15	1.3%	-16.70 [-22.95, -10.45]
Zehsaz 2016	12.3	2.6	16	16.6	3.1	16	3.2%	-4.30 [-6.28, -2.32]
**Subtotal (95% CI)**			**793**			**669**	**55.2%**	**-4.31 [-5.66, -2.96]**
Heterogeneity: Tau² = 8.03; Chi² = 161.44, df = 32 (P < 0.00001); I² = 80%								
Test for overall effect: Z = 6.26 (P < 0.00001)								
**Total (95% CI)**			**1420**			**1051**	**100.0%**	**-3.93 [-4.80, -3.06]**
Heterogeneity: Tau² = 5.23; Chi² = 233.37, df = 56 (P < 0.00001); I² = 76%								
Test for overall effect: Z = 8.83 (P < 0.00001)								
Test for subgroup differences: Chi² = 0.98, df = 1 (P = 0.32), I² = 0%								

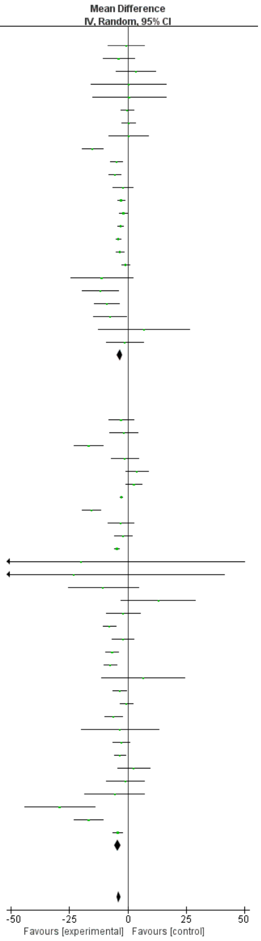

Figure S17.6. Forest plot of subgroup analyses by intervention duration

Study or Subgroup	Experimental Mean	Experimental SD	Experimental Total	Control Mean	Control SD	Control Total	Weight	Mean Difference IV, Random, 95% CI
**2.6.1 <12 weeks (total intervention duration)**								
Benson 2008	15.33	9.6	32	16.03	20.85	35	1.0%	-0.70 [-8.37, 6.97]
Bowen 2010	15.05	4.7	22	16.29	12.2	18	1.4%	-1.24 [-7.21, 4.73]
Kelly 2004	11.12	5	10	14.12	7.43	10	1.5%	-3.00 [-8.55, 2.55]
Kim 2007	8.9	4.98	14	10.8	4.5	12	2.3%	-1.90 [-5.55, 1.75]
Liu 2018	8.95	4.39	30	16.78	4.85	20	2.8%	-7.83 [-10.47, -5.19]
Luo 2013(1)	6.22	7.37	43	12.83	5.11	34	2.7%	-6.61 [-9.40, -3.82]
Luo 2013(2)	8.73	7.97	52	16.27	5.27	38	2.8%	-7.54 [-10.28, -4.80]
McCormack 2014	27.67	21.32	10	21.17	17.33	8	0.2%	6.50 [-11.36, 24.36]
Seo 2012	6.63	8.66	10	9.93	25.11	10	0.3%	-3.30 [-19.76, 13.16]
Tas 2023	35.85	22.41	31	28.86	22.41	6	0.2%	6.99 [-12.60, 26.58]
**Subtotal (95% CI)**			**254**			**191**	**15.1%**	**-4.73 [-6.89, -2.57]**
Heterogeneity: Tau² = 4.55; Chi² = 16.99, df = 9 (P = 0.05); I² = 47%								
Test for overall effect: Z = 4.29 (P < 0.0001)								
**2.6.2 ⩾12 weeks (total intervention duration)**								
Abassi 2020(1)	9.46	2.08	8	12.3	5.31	4	1.5%	-2.84 [-8.24, 2.56]
Abassi 2020(2)	10.6	4.1	8	12.3	5.31	4	1.4%	-1.70 [-7.63, 4.23]
Bharath 2018	10.3	9.39	20	27	10.73	20	1.3%	-16.70 [-22.95, -10.45]
Carrel 2005	21.9	14	27	25.8	10	23	1.2%	-3.90 [-10.58, 2.78]
Davis(1) 2009	27.8	12.5	17	24.3	14.5	21	0.8%	3.50 [-5.09, 12.09]
DAVIS(2) 2009(1)	28.3	8.01	9	28	17.5	5	0.3%	0.30 [-15.91, 16.51]
DAVIS(2) 2009(2)	28.6	7.49	15	28	17.5	5	0.3%	0.60 [-15.20, 16.40]
Davis 2020	23.4	15.67	90	19.4	16.08	85	1.8%	4.00 [-0.71, 8.71]
Faria 2020(1)	10.8	4.14	24	11	4.21	13	2.7%	-0.20 [-3.03, 2.63]
Faria 2020(2)	11.4	4.33	26	11	4.21	13	2.7%	0.40 [-2.43, 3.23]
Farpour-Lambert 2009	16.13	6.77	22	13.5	5.27	22	2.3%	2.63 [-0.96, 6.22]
Gulijianati 2022	5.18	1.23	47	7.89	1.29	46	3.7%	-2.71 [-3.22, -2.20]
Jianjun 2012	13.34	4.53	20	28.92	6.44	15	2.2%	-15.58 [-19.40, -11.76]
Kim 2020	9.1	1.2	24	13.7	2.2	24	3.6%	-4.60 [-5.60, -3.60]
Lee 2012(1)	20.1	69.38	15	40	69.05	5	0.0%	-19.90 [-89.87, 50.07]
Lee 2012(2)	17	66.94	16	40	69.05	6	0.0%	-23.00 [-87.25, 41.25]
Lee 2013(1)	20.57	14.71	14	31.05	13.23	4	0.3%	-10.48 [-25.56, 4.60]
Lee 2013(2)	44.15	17.49	14	31.05	13.23	4	0.3%	13.10 [-2.78, 28.98]
Lira 2017(1)	27.75	15.35	26	27.4	12.57	16	0.8%	0.35 [-8.18, 8.88]
Lira 2017(2)	25.48	10.3	25	27.4	12.57	17	1.1%	-1.92 [-9.13, 5.29]
Lopes 2016	14.7	7.4	17	16.8	6.5	16	1.8%	-2.10 [-6.85, 2.65]
Machado 2022	14.7	7.6	20	29.9	6	15	1.9%	-15.20 [-19.71, -10.69]
Meng 2022(1)	7.5	2.8	12	12.3	2.4	6	2.9%	-4.80 [-7.29, -2.31]
Meng 2022(2)	6.8	3	11	12.3	2.4	7	2.9%	-5.50 [-8.01, -2.99]
Meyer 2006	11.16	4.61	33	14.6	7.65	34	2.6%	-3.44 [-6.45, -0.43]
Migueles 2023	13.36	6.32	39	13.91	6.33	37	2.7%	-0.55 [-3.40, 2.30]
Park 2007	8.3	3	19	14.4	8.2	21	2.2%	-6.10 [-9.86, -2.34]
Pena 2022	19	8.83	78	21	12.33	38	1.9%	-2.00 [-6.38, 2.38]
Racil(1) 2016(1)	16.3	0.84	17	19.2	2	7	3.4%	-2.90 [-4.43, -1.37]
Racil(1) 2016(2)	17.3	1.5	16	19.2	2	7	3.3%	-1.90 [-3.55, -0.25]
Racil(2) 2016(1)	15.6	1.2	23	18.6	1.6	9	3.6%	-3.00 [-4.15, -1.85]
Racil(2) 2016(2)	14.6	1	26	18.6	1.6	10	3.6%	-4.00 [-5.06, -2.94]
Racil 2013(1)	15.5	1.19	11	18.8	1.95	6	3.3%	-3.30 [-5.01, -1.59]
Racil 2013(2)	17.9	1.45	11	18.8	1.95	6	3.3%	-0.90 [-2.68, 0.88]
Salus(2) 2022	20.64	6.73	14	31.8	24.69	14	0.4%	-11.16 [-24.57, 2.25]
Savoye 2007	17.9	27.11	75	29.5	16.2	44	0.9%	-11.60 [-19.38, -3.82]
Savoye 2011	18.4	4.72	75	27.4	17.73	44	1.6%	-9.00 [-14.35, -3.65]
Shaibi 2006	11.5	8.29	11	19.1	8.54	10	1.1%	-7.60 [-14.81, -0.39]
Shalitin 2009	8.88	9.12	55	11.62	10.01	55	2.3%	-2.74 [-6.32, 0.84]
Son 2017	9.1	1.34	20	12.5	5.37	20	2.9%	-3.40 [-5.83, -0.97]
Staiano 2017	26.1	13.37	20	23.47	7.63	18	1.1%	2.63 [-4.21, 9.47]
Tjonna 2009	22.02	12.82	20	23.33	13.6	22	0.9%	-1.31 [-9.30, 6.68]
Tsang 2009	19.95	12.57	11	20.89	5	8	0.9%	-0.94 [-9.14, 7.26]
Vasconcellos 2016	31.5	9.2	10	37.1	18.6	10	0.4%	-5.60 [-18.46, 7.26]
Wen 2008	22.08	12.64	24	51.23	26.09	13	0.3%	-29.15 [-44.21, -14.09]
Wong 2018	10.3	8.13	15	27	9.3	15	1.3%	-16.70 [-22.95, -10.45]
Zehsaz 2016	12.3	2.6	16	16.6	3.1	16	3.2%	-4.30 [-6.28, -2.32]
**Subtotal (95% CI)**			**1166**			**860**	**84.9%**	**-3.79 [-4.73, -2.86]**
Heterogeneity: Tau² = 5.03; Chi² = 203.63, df = 46 (P < 0.00001); I² = 77%								
Test for overall effect: Z = 7.96 (P < 0.00001)								
**Total (95% CI)**			**1420**			**1051**	**100.0%**	**-3.93 [-4.80, -3.06]**
Heterogeneity: Tau² = 5.23; Chi² = 233.37, df = 56 (P < 0.00001); I² = 76%								
Test for overall effect: Z = 8.83 (P < 0.00001)								
Test for subgroup differences: Chi² = 0.61, df = 1 (P = 0.44), I² = 0%								

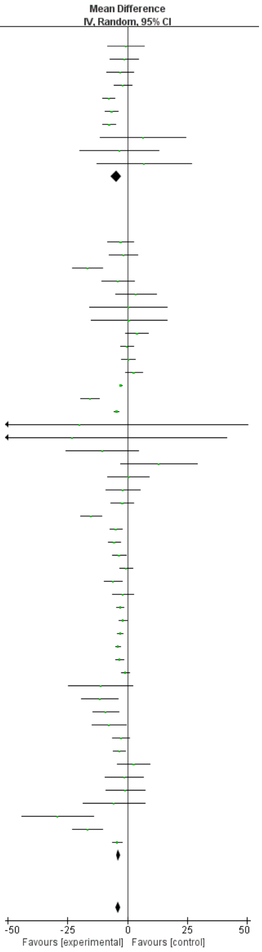

Figure S17.7. Forest plot of subgroup analyses by region

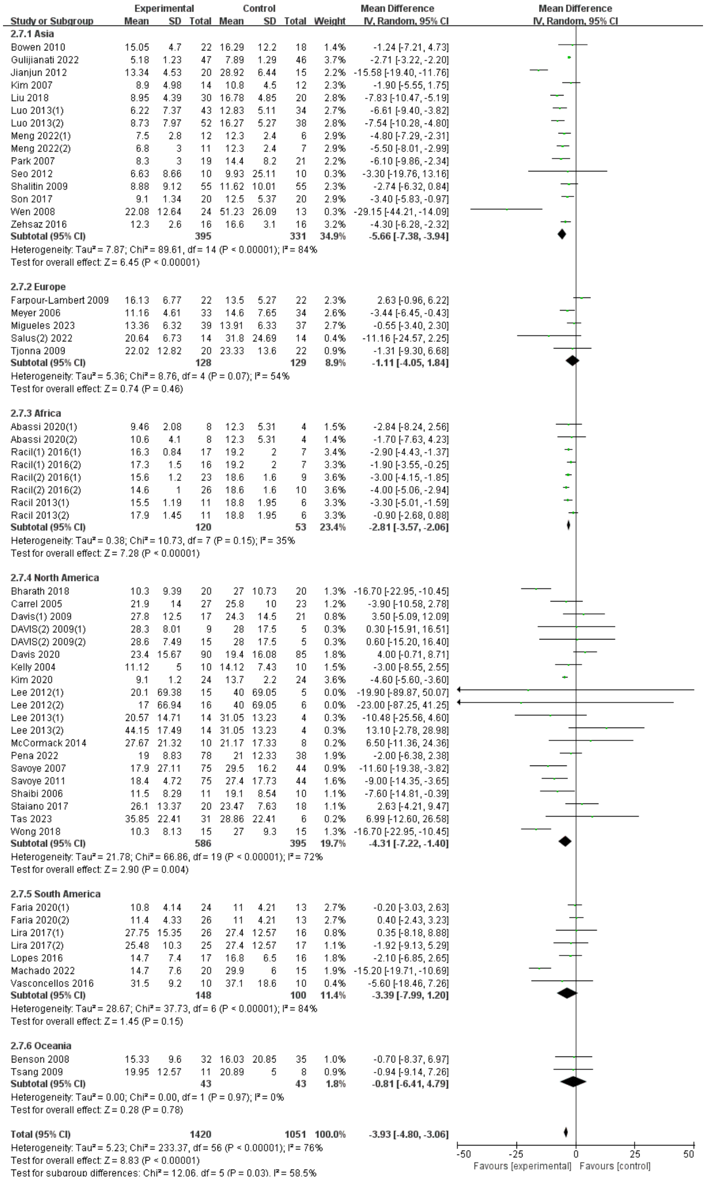

Figure S17.8. Forest plot of subgroup analyses by economic status

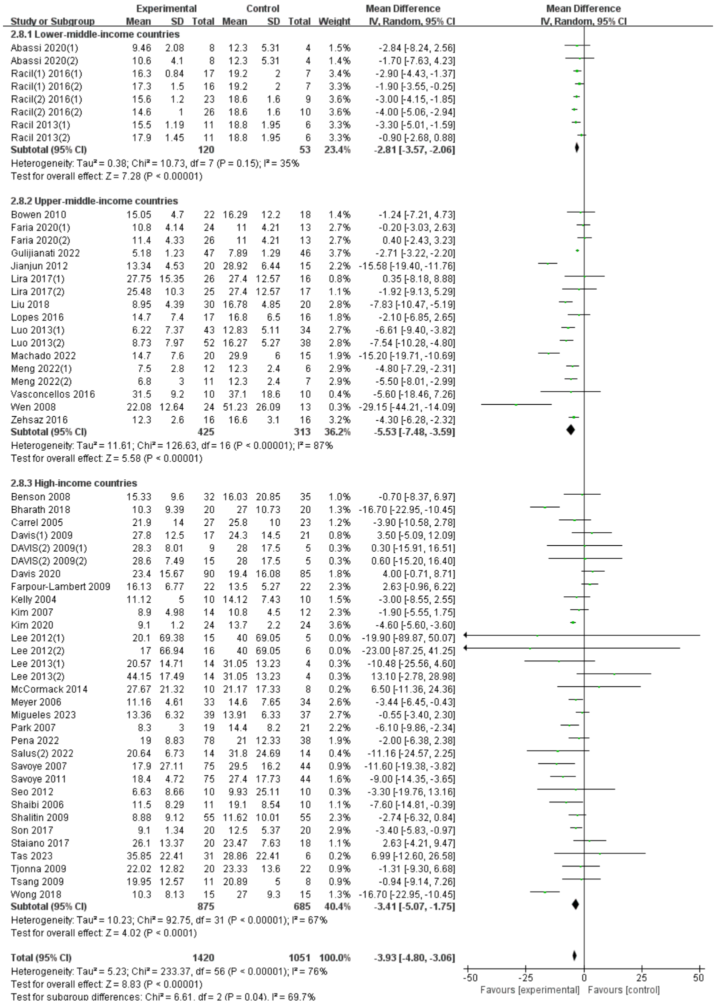

Figure S17.9. Forest plot of subgroup analyses by weight status

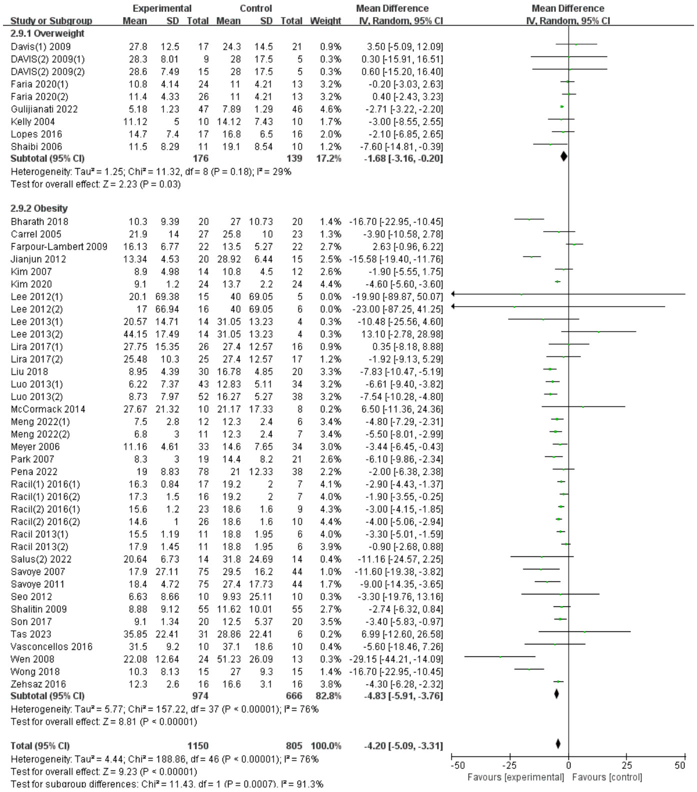

Figure S17.10. Forest plot of subgroup analyses by sex

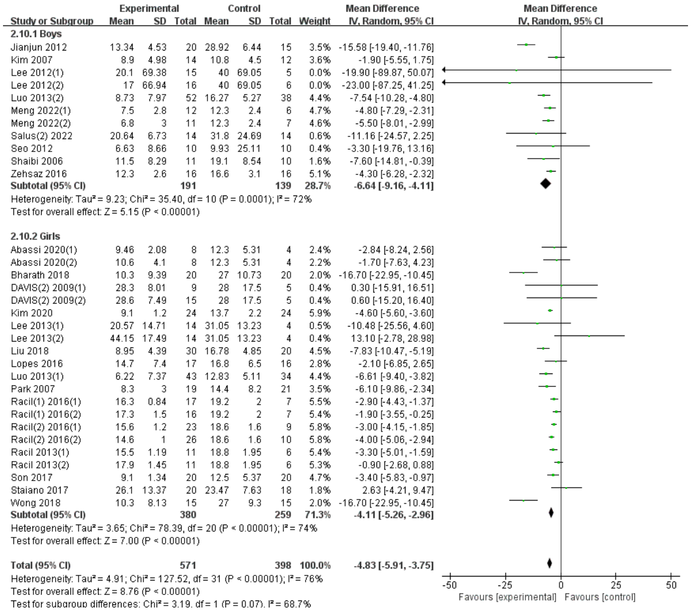

Figure S17.11. Forest plot of subgroup analyses by metabolic status

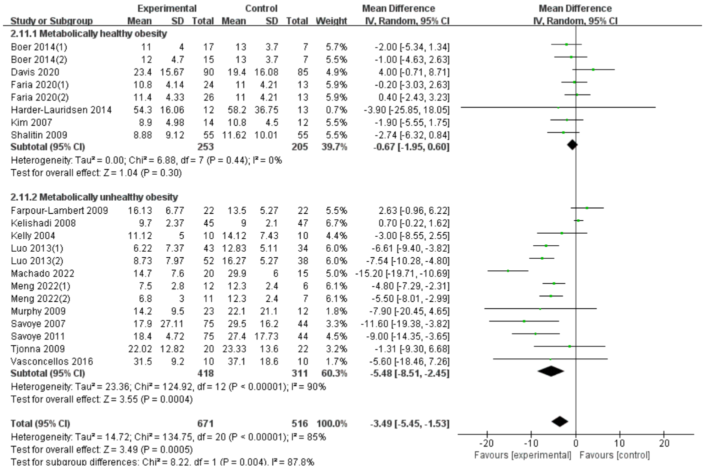

Figure S18. Forest plots of subgroup analyses for Depression in children and adolescents with overweight or obesity

Figure S18.1. Forest plot of subgroup analyses by type of exercise

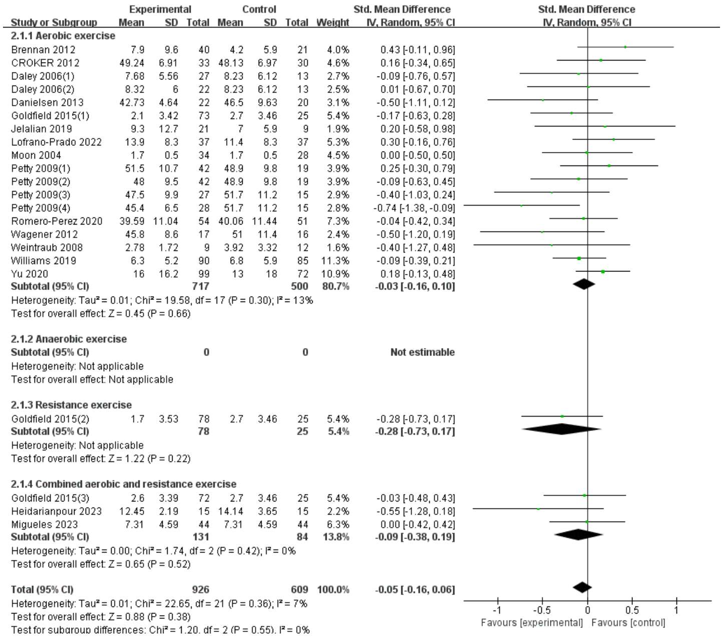

Figure S18.2. Forest plot of subgroup analyses by exercise intensity

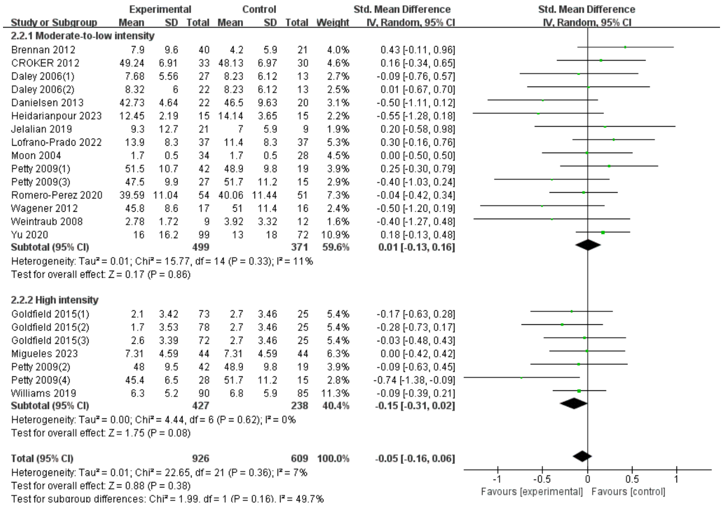

Figure S18.3. Forest plot of subgroup analyses by duration per session

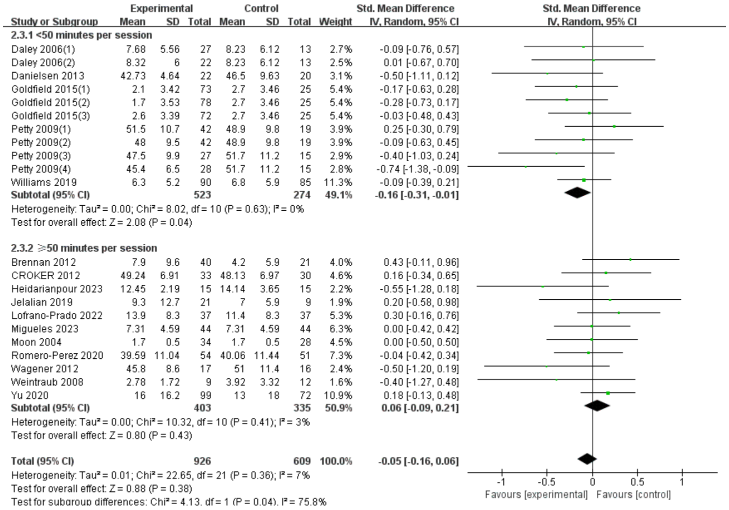

Figure S18.4. Forest plot of subgroup analyses by exercise frequency

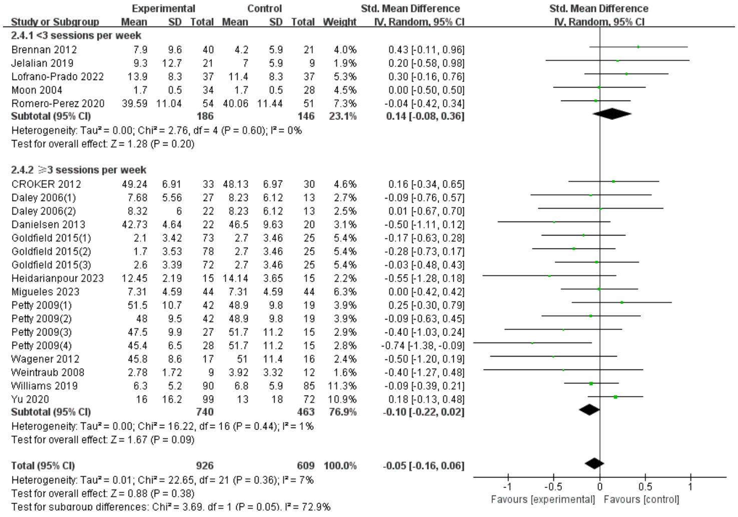

Figure S18.5. Forest plot of subgroup analyses by weekly exercise duration

Study or Subgroup	Experimental Mean	Experimental SD	Experimental Total	Control Mean	Control SD	Control Total	Weight	Std. Mean Difference IV, Random, 95% CI
**2.5.1 <150 minutes per week**								
Brennan 2012	7.9	9.6	40	4.2	5.9	21	4.0%	0.43 [-0.11, 0.96]
Daley 2006(1)	7.68	5.56	27	8.23	6.12	13	2.7%	-0.09 [-0.76, 0.57]
Daley 2006(2)	8.32	6	22	8.23	6.12	13	2.5%	0.01 [-0.67, 0.70]
Goldfield 2015(1)	2.1	3.42	73	2.7	3.46	25	5.4%	-0.17 [-0.63, 0.28]
Goldfield 2015(2)	1.7	3.53	78	2.7	3.46	25	5.4%	-0.28 [-0.73, 0.17]
Goldfield 2015(3)	2.6	3.39	72	2.7	3.46	25	5.4%	-0.03 [-0.48, 0.43]
Jelalian 2019	9.3	12.7	21	7	5.9	9	1.9%	0.20 [-0.58, 0.98]
Lofrano-Prado 2022	13.9	8.3	37	11.4	8.3	37	5.3%	0.30 [-0.16, 0.76]
Moon 2004	1.7	0.5	34	1.7	0.5	28	4.5%	0.00 [-0.50, 0.50]
Petty 2009(1)	51.5	10.7	42	48.9	9.8	19	3.9%	0.25 [-0.30, 0.79]
Petty 2009(3)	47.5	9.9	27	51.7	11.2	15	2.9%	-0.40 [-1.03, 0.24]
Romero-Perez 2020	39.59	11.04	54	40.06	11.44	51	7.3%	-0.04 [-0.42, 0.34]
**Subtotal (95% CI)**			**527**			**281**	**51.1%**	**0.01 [-0.14, 0.15]**
Heterogeneity: Tau² = 0.00; Chi² = 8.82, df = 11 (P = 0.64); I² = 0%								
Test for overall effect: Z = 0.08 (P = 0.94)								
**2.5.2 ≥150 minutes per week**								
CROKER 2012	49.24	6.91	33	48.13	6.97	30	4.6%	0.16 [-0.34, 0.65]
Danielsen 2013	42.73	4.64	22	46.5	9.63	20	3.1%	-0.50 [-1.11, 0.12]
Heidarianpour 2023	12.45	2.19	15	14.14	3.65	15	2.2%	-0.55 [-1.28, 0.18]
Migueles 2023	7.31	4.59	44	7.31	4.59	44	6.3%	0.00 [-0.42, 0.42]
Petty 2009(2)	48	9.5	42	48.9	9.8	19	3.9%	-0.09 [-0.63, 0.45]
Petty 2009(4)	45.4	6.5	28	51.7	11.2	15	2.8%	-0.74 [-1.38, -0.09]
Wagener 2012	45.8	8.6	17	51	11.4	16	2.4%	-0.50 [-1.20, 0.19]
Weintraub 2008	2.78	1.72	9	3.92	3.32	12	1.6%	-0.40 [-1.27, 0.48]
Williams 2019	6.3	5.2	90	6.8	5.9	85	11.3%	-0.09 [-0.39, 0.21]
Yu 2020	16	16.2	99	13	18	72	10.9%	0.18 [-0.13, 0.48]
**Subtotal (95% CI)**			**399**			**328**	**48.9%**	**-0.14 [-0.33, 0.05]**
Heterogeneity: Tau² = 0.03; Chi² = 12.90, df = 9 (P = 0.17); I² = 30%								
Test for overall effect: Z = 1.47 (P = 0.14)								
**Total (95% CI)**			**926**			**609**	**100.0%**	**-0.05 [-0.16, 0.06]**
Heterogeneity: Tau² = 0.01; Chi² = 22.65, df = 21 (P = 0.36); I² = 7%								
Test for overall effect: Z = 0.88 (P = 0.38)								
Test for subgroup differences: Chi² = 1.46, df = 1 (P = 0.23), I² = 31.6%								

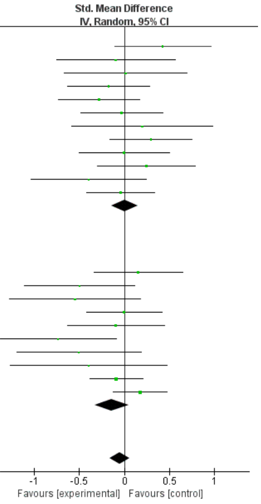

Figure S18.6. Forest plot of subgroup analyses by intervention duration

Study or Subgroup	Experimental Mean	Experimental SD	Experimental Total	Control Mean	Control SD	Control Total	Weight	Std. Mean Difference IV, Random, 95% CI
**2.6.1 <12 weeks (total intervention duration)**								
Daley 2006(1)	7.68	5.56	27	8.23	6.12	13	2.7%	-0.09 [-0.76, 0.57]
Daley 2006(2)	8.32	6	22	8.23	6.12	13	2.5%	0.01 [-0.67, 0.70]
Moon 2004	1.7	0.5	34	1.7	0.5	28	4.5%	0.00 [-0.50, 0.50]
Wagener 2012	45.8	8.6	17	51	11.4	16	2.4%	-0.50 [-1.20, 0.19]
**Subtotal (95% CI)**			**100**			**70**	**12.1%**	**-0.12 [-0.43, 0.19]**
Heterogeneity: Tau² = 0.00; Chi² = 1.55, df = 3 (P = 0.67); I² = 0%								
Test for overall effect: Z = 0.74 (P = 0.46)								
**2.6.2 ≥12 weeks (total intervention duration)**								
Brennan 2012	7.9	9.6	40	4.2	5.9	21	4.0%	0.43 [-0.11, 0.96]
CROKER 2012	49.24	6.91	33	48.13	6.97	30	4.6%	0.16 [-0.34, 0.65]
Danielsen 2013	42.73	4.64	22	46.5	9.63	20	3.1%	-0.50 [-1.11, 0.12]
Goldfield 2015(1)	2.1	3.42	73	2.7	3.46	25	5.4%	-0.17 [-0.63, 0.28]
Goldfield 2015(2)	1.7	3.53	78	2.7	3.46	25	5.4%	-0.28 [-0.73, 0.17]
Goldfield 2015(3)	2.6	3.39	72	2.7	3.46	25	5.4%	-0.03 [-0.48, 0.43]
Heidarianpour 2023	12.45	2.19	15	14.14	3.65	15	2.2%	-0.55 [-1.28, 0.18]
Jelalian 2019	9.3	12.7	21	7	5.9	9	1.9%	0.20 [-0.58, 0.98]
Lofrano-Prado 2022	13.9	8.3	37	11.4	8.3	37	5.3%	0.30 [-0.16, 0.76]
Migueles 2023	7.31	4.59	44	7.31	4.59	44	6.3%	0.00 [-0.42, 0.42]
Petty 2009(1)	51.5	10.7	42	48.9	9.8	19	3.9%	0.25 [-0.30, 0.79]
Petty 2009(2)	48	9.5	42	48.9	9.8	19	3.9%	-0.09 [-0.63, 0.45]
Petty 2009(3)	47.5	9.9	27	51.7	11.2	15	2.9%	-0.40 [-1.03, 0.24]
Petty 2009(4)	45.4	6.5	28	51.7	11.2	15	2.8%	-0.74 [-1.38, -0.09]
Romero-Perez 2020	39.59	11.04	54	40.06	11.44	51	7.3%	-0.04 [-0.42, 0.34]
Weintraub 2008	2.78	1.72	9	3.92	3.32	12	1.6%	-0.40 [-1.27, 0.48]
Williams 2019	6.3	5.2	90	6.8	5.9	85	11.3%	-0.09 [-0.39, 0.21]
Yu 2020	16	16.2	99	13	18	72	10.9%	0.18 [-0.13, 0.48]
**Subtotal (95% CI)**			**826**			**539**	**87.9%**	**-0.05 [-0.17, 0.08]**
Heterogeneity: Tau² = 0.01; Chi² = 20.86, df = 17 (P = 0.23); I² = 19%								
Test for overall effect: Z = 0.70 (P = 0.48)								
**Total (95% CI)**			**926**			**609**	**100.0%**	**-0.05 [-0.16, 0.06]**
Heterogeneity: Tau² = 0.01; Chi² = 22.65, df = 21 (P = 0.36); I² = 7%								
Test for overall effect: Z = 0.88 (P = 0.38)								
Test for subgroup differences: Chi² = 0.18, df = 1 (P = 0.67), I² = 0%								

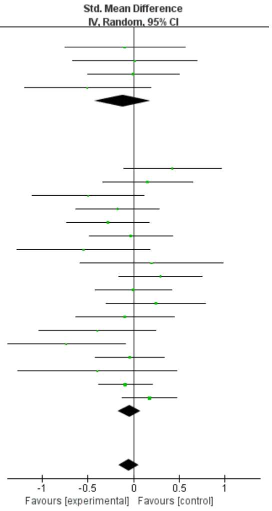

Figure S18.7. Forest plot of subgroup analyses by region

Study or Subgroup	Experimental Mean	Experimental SD	Experimental Total	Control Mean	Control SD	Control Total	Weight	Std. Mean Difference IV, Random, 95% CI
**2.7.1 Asia**								
Heidarianpour 2023	12.45	2.19	15	14.14	3.65	15	2.2%	-0.55 [-1.28, 0.18]
Moon 2004	1.7	0.5	34	1.7	0.5	28	4.5%	0.00 [-0.50, 0.50]
Yu 2020	16	16.2	99	13	18	72	10.9%	0.18 [-0.13, 0.48]
**Subtotal (95% CI)**			**148**			**115**	**17.6%**	**-0.01 [-0.36, 0.34]**
Heterogeneity: Tau² = 0.04; Chi² = 3.25, df = 2 (P = 0.20); I² = 39%								
Test for overall effect: Z = 0.05 (P = 0.96)								
**2.7.2 Europe**								
CROKER 2012	49.24	6.91	33	48.13	6.97	30	4.6%	0.16 [-0.34, 0.65]
Daley 2006(1)	7.68	5.56	27	8.23	6.12	13	2.7%	-0.09 [-0.76, 0.57]
Daley 2006(2)	8.32	6	22	8.23	6.12	13	2.5%	0.01 [-0.67, 0.70]
Danielsen 2013	42.73	4.64	22	46.5	9.63	20	3.1%	-0.50 [-1.11, 0.12]
Migueles 2023	7.31	4.59	44	7.31	4.59	44	6.3%	0.00 [-0.42, 0.42]
**Subtotal (95% CI)**			**148**			**120**	**19.1%**	**-0.05 [-0.29, 0.19]**
Heterogeneity: Tau² = 0.00; Chi² = 2.81, df = 4 (P = 0.59); I² = 0%								
Test for overall effect: Z = 0.41 (P = 0.68)								
**2.7.3 Africa**								
**Subtotal (95% CI)**			**0**			**0**		**Not estimable**
Heterogeneity: Not applicable								
Test for overall effect: Not applicable								
**2.7.4 North America**								
Goldfield 2015(1)	2.1	3.42	73	2.7	3.46	25	5.4%	-0.17 [-0.63, 0.28]
Goldfield 2015(2)	1.7	3.53	78	2.7	3.46	25	5.4%	-0.28 [-0.73, 0.17]
Goldfield 2015(3)	2.6	3.39	72	2.7	3.46	25	5.4%	-0.03 [-0.48, 0.43]
Jelalian 2019	9.3	12.7	21	7	5.9	9	1.9%	0.20 [-0.58, 0.98]
Petty 2009(1)	51.5	10.7	42	48.9	9.8	19	3.9%	0.25 [-0.30, 0.79]
Petty 2009(2)	48	9.5	42	48.9	9.8	19	3.9%	-0.09 [-0.63, 0.45]
Petty 2009(3)	47.5	9.9	27	51.7	11.2	15	2.9%	-0.40 [-1.03, 0.24]
Petty 2009(4)	45.4	6.5	28	51.7	11.2	15	2.8%	-0.74 [-1.38, -0.09]
Romero-Perez 2020	39.59	11.04	54	40.06	11.44	51	7.3%	-0.04 [-0.42, 0.34]
Wagener 2012	45.8	8.6	17	51	11.4	16	2.4%	-0.50 [-1.20, 0.19]
Weintraub 2008	2.78	1.72	9	3.92	3.32	12	1.6%	-0.40 [-1.27, 0.48]
Williams 2019	6.3	5.2	90	6.8	5.9	85	11.3%	-0.09 [-0.39, 0.21]
**Subtotal (95% CI)**			**553**			**316**	**54.1%**	**-0.14 [-0.29, -0.00]**
Heterogeneity: Tau² = 0.00; Chi² = 8.94, df = 11 (P = 0.63); I² = 0%								
Test for overall effect: Z = 1.98 (P = 0.05)								
**2.7.5 South America**								
Lofrano-Prado 2022	13.9	8.3	37	11.4	8.3	37	5.3%	0.30 [-0.16, 0.76]
**Subtotal (95% CI)**			**37**			**37**	**5.3%**	**0.30 [-0.16, 0.76]**
Heterogeneity: Not applicable								
Test for overall effect: Z = 1.27 (P = 0.20)								
**2.7.6 Oceania**								
Brennan 2012	7.9	9.6	40	4.2	5.9	21	4.0%	0.43 [-0.11, 0.96]
**Subtotal (95% CI)**			**40**			**21**	**4.0%**	**0.43 [-0.11, 0.96]**
Heterogeneity: Not applicable								
Test for overall effect: Z = 1.57 (P = 0.12)								
**Total (95% CI)**			**926**			**609**	**100.0%**	**-0.05 [-0.16, 0.06]**
Heterogeneity: Tau² = 0.01; Chi² = 22.65, df = 21 (P = 0.36); I² = 7%								
Test for overall effect: Z = 0.88 (P = 0.38)								
Test for subgroup differences: Chi² = 6.99, df = 4 (P = 0.14), I² = 42.7%								

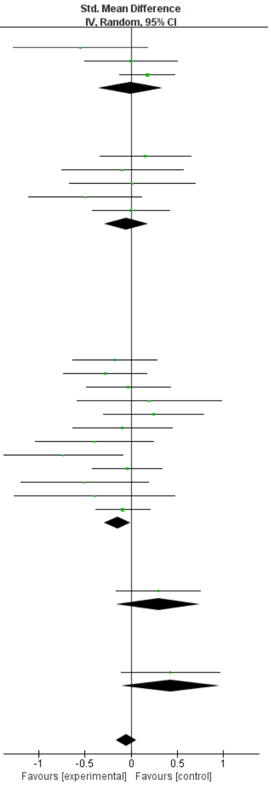

Figure S18.8. Forest plot of subgroup analyses by economic status

Study or Subgroup	Experimental Mean	Experimental SD	Experimental Total	Control Mean	Control SD	Control Total	Weight	Std. Mean Difference IV, Random, 95% CI
**2.8.1 Lower-middle-income countries**								
**Subtotal (95% CI)**			**0**			**0**		**Not estimable**
Heterogeneity: Not applicable								
Test for overall effect: Not applicable								
**2.8.2 Upper-middle-income countries**								
Heidarianpour 2023	12.45	2.19	15	14.14	3.65	15	2.2%	-0.55 [-1.28, 0.18]
Lofrano-Prado 2022	13.9	8.3	37	11.4	8.3	37	5.3%	0.30 [-0.16, 0.76]
Romero-Perez 2020	39.59	11.04	54	40.06	11.44	51	7.3%	-0.04 [-0.42, 0.34]
Yu 2020	16	16.2	99	13	18	72	10.9%	0.18 [-0.13, 0.48]
**Subtotal (95% CI)**			**205**			**175**	**25.7%**	**0.06 [-0.20, 0.32]**
Heterogeneity: Tau² = 0.02; Chi² = 4.46, df = 3 (P = 0.22); I² = 33%								
Test for overall effect: Z = 0.47 (P = 0.64)								
**2.8.3 High-income countries**								
Brennan 2012	7.9	9.6	40	4.2	5.9	21	4.0%	0.43 [-0.11, 0.96]
CROKER 2012	49.24	6.91	33	48.13	6.97	30	4.6%	0.16 [-0.34, 0.65]
Daley 2006(1)	7.68	5.56	27	8.23	6.12	13	2.7%	-0.09 [-0.76, 0.57]
Daley 2006(2)	8.32	6	22	8.23	6.12	13	2.5%	0.01 [-0.67, 0.70]
Danielsen 2013	42.73	4.64	22	46.5	9.63	20	3.1%	-0.50 [-1.11, 0.12]
Goldfield 2015(1)	2.1	3.42	73	2.7	3.46	25	5.4%	-0.17 [-0.63, 0.28]
Goldfield 2015(2)	1.7	3.53	78	2.7	3.46	25	5.4%	-0.28 [-0.73, 0.17]
Goldfield 2015(3)	2.6	3.39	72	2.7	3.46	25	5.4%	-0.03 [-0.48, 0.43]
Jelalian 2019	9.3	12.7	21	7	5.9	9	1.9%	0.20 [-0.58, 0.98]
Migueles 2023	7.31	4.59	44	7.31	4.59	44	6.3%	0.00 [-0.42, 0.42]
Moon 2004	1.7	0.5	34	1.7	0.5	28	4.5%	0.00 [-0.50, 0.50]
Petty 2009(1)	51.5	10.7	42	48.9	9.8	19	3.9%	0.25 [-0.30, 0.79]
Petty 2009(2)	48	9.5	42	48.9	9.8	19	3.9%	-0.09 [-0.63, 0.45]
Petty 2009(3)	47.5	9.9	27	51.7	11.2	15	2.9%	-0.40 [-1.03, 0.24]
Petty 2009(4)	45.4	6.5	28	51.7	11.2	15	2.8%	-0.74 [-1.38, -0.09]
Wagener 2012	45.8	8.6	17	51	11.4	16	2.4%	-0.50 [-1.20, 0.19]
Weintraub 2008	2.78	1.72	9	3.92	3.32	12	1.6%	-0.40 [-1.27, 0.48]
Williams 2019	6.3	5.2	90	6.8	5.9	85	11.3%	-0.09 [-0.39, 0.21]
**Subtotal (95% CI)**			**721**			**434**	**74.3%**	**-0.09 [-0.21, 0.03]**
Heterogeneity: Tau² = 0.00; Chi² = 16.09, df = 17 (P = 0.52); I² = 0%								
Test for overall effect: Z = 1.48 (P = 0.14)								
**Total (95% CI)**			**926**			**609**	**100.0%**	**-0.05 [-0.16, 0.06]**
Heterogeneity: Tau² = 0.01; Chi² = 22.65, df = 21 (P = 0.36); I² = 7%								
Test for overall effect: Z = 0.88 (P = 0.38)								
Test for subgroup differences: Chi² = 1.11, df = 1 (P = 0.29), I² = 9.6%								

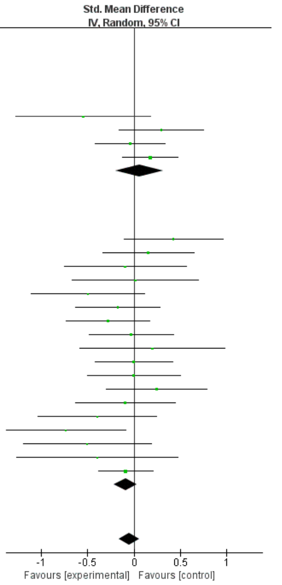

Figure S18.9. Forest plot of subgroup analyses by weight status

	Experimental			Control				Std. Mean Difference
Study or Subgroup	Mean	SD	Total	Mean	SD	Total	Weight	IV, Random, 95% CI
**2.9.1 Overweight**								
Petty 2009(1)	51.5	10.7	42	48.9	9.8	19	5.6%	0.25 [-0.30, 0.79]
Petty 2009(2)	48	9.5	42	48.9	9.8	19	5.6%	-0.09 [-0.63, 0.45]
Petty 2009(3)	47.5	9.9	27	51.7	11.2	15	4.2%	-0.40 [-1.03, 0.24]
Petty 2009(4)	45.4	6.5	28	51.7	11.2	15	4.0%	-0.74 [-1.38, -0.09]
Weintraub 2008	2.78	1.72	9	3.92	3.32	12	2.3%	-0.40 [-1.27, 0.48]
**Subtotal (95% CI)**			**148**			**80**	**21.6%**	**-0.23 [-0.58, 0.11]**
Heterogeneity: $Tau^2 = 0.05$; $Chi^2 = 5.92$, df = 4 (P = 0.21); $I^2 = 32\%$								
Test for overall effect: Z = 1.33 (P = 0.18)								
**2.9.2 Obesity**								
Daley 2006(1)	7.68	5.56	27	8.23	6.12	13	3.9%	-0.09 [-0.76, 0.57]
Daley 2006(2)	8.32	6	22	8.23	6.12	13	3.6%	0.01 [-0.67, 0.70]
Danielsen 2013	42.73	4.64	22	46.5	9.63	20	4.4%	-0.50 [-1.11, 0.12]
Goldfield 2015(1)	2.1	3.42	73	2.7	3.46	25	7.7%	-0.17 [-0.63, 0.28]
Goldfield 2015(2)	1.7	3.53	78	2.7	3.46	25	7.8%	-0.28 [-0.73, 0.17]
Goldfield 2015(3)	2.6	3.39	72	2.7	3.46	25	7.7%	-0.03 [-0.48, 0.43]
Lofrano-Prado 2022	13.9	8.3	37	11.4	8.3	37	7.6%	0.30 [-0.16, 0.76]
Moon 2004	1.7	0.5	34	1.7	0.5	28	6.5%	0.00 [-0.50, 0.50]
Romero-Perez 2020	39.59	11.04	54	40.06	11.44	51	10.4%	-0.04 [-0.42, 0.34]
Wagener 2012	45.8	8.6	17	51	11.4	16	3.5%	-0.50 [-1.20, 0.19]
Yu 2020	16	16.2	99	13	18	72	15.2%	0.18 [-0.13, 0.48]
**Subtotal (95% CI)**			**535**			**325**	**78.4%**	**-0.04 [-0.18, 0.10]**
Heterogeneity: $Tau^2 = 0.00$; $Chi^2 = 9.37$, df = 10 (P = 0.50); $I^2 = 0\%$								
Test for overall effect: Z = 0.52 (P = 0.61)								
**Total (95% CI)**			**683**			**405**	**100.0%**	**-0.08 [-0.22, 0.05]**
Heterogeneity: $Tau^2 = 0.01$; $Chi^2 = 16.48$, df = 15 (P = 0.35); $I^2 = 9\%$								
Test for overall effect: Z = 1.20 (P = 0.23)								
Test for subgroup differences: $Chi^2 = 1.06$, df = 1 (P = 0.30), $I^2 = 5.8\%$								

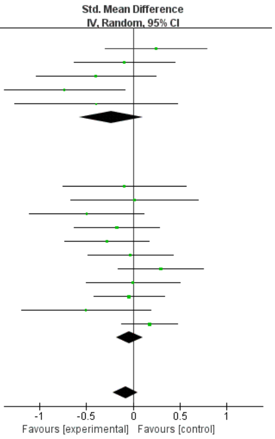

Figure S18.10. Forest plot of subgroup analyses by sex

	Experimental			Control				Std. Mean Difference
Study or Subgroup	Mean	SD	Total	Mean	SD	Total	Weight	IV, Random, 95% CI
**2.10.1 Boys**								
**Subtotal (95% CI)**			**0**			**0**		**Not estimable**
Heterogeneity: Not applicable								
Test for overall effect: Not applicable								
**2.10.2 Girls**								
Heidarianpour 2023	12.45	2.19	15	14.14	3.65	15	100.0%	-0.55 [-1.28, 0.18]
**Subtotal (95% CI)**			**15**			**15**	**100.0%**	**-0.55 [-1.28, 0.18]**
Heterogeneity: Not applicable								
Test for overall effect: Z = 1.47 (P = 0.14)								
**Total (95% CI)**			**15**			**15**	**100.0%**	**-0.55 [-1.28, 0.18]**
Heterogeneity: Not applicable								
Test for overall effect: Z = 1.47 (P = 0.14)								
Test for subgroup differences: Not applicable								

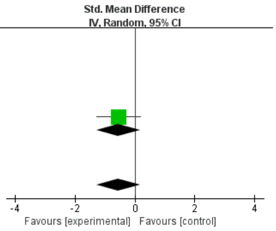

Figure S19. Forest plots of subgroup analyses for Anxiety in children and adolescents with overweight or obesity

Figure S19.1. Forest plot of subgroup analyses by type of exercise

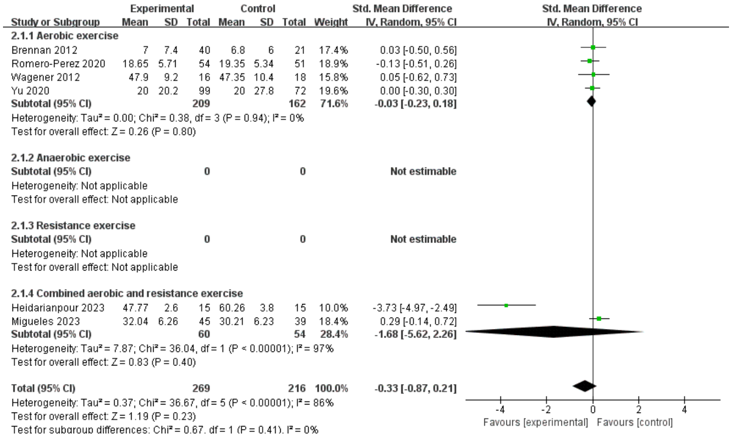

Figure S19.2. Forest plot of subgroup analyses by exercise intensity

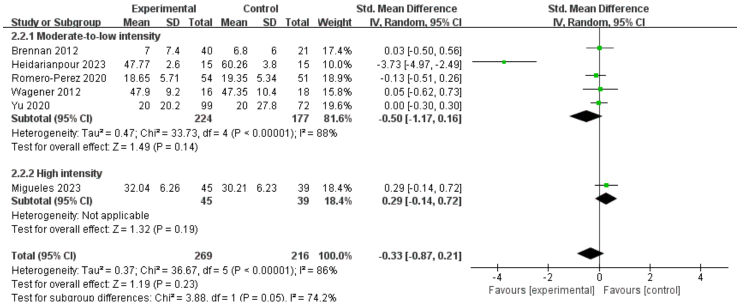

Figure S19.3. Forest plot of subgroup analyses by duration per session

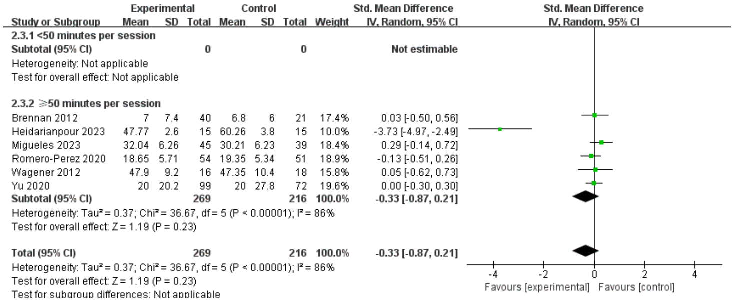

Figure S19.4. Forest plot of subgroup analyses by exercise frequency

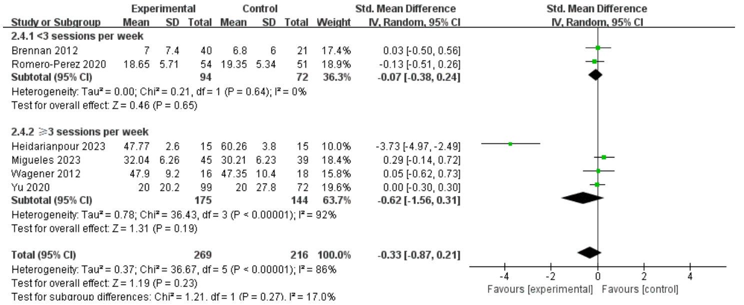

Figure S19.5. Forest plot of subgroup analyses by weekly exercise duration

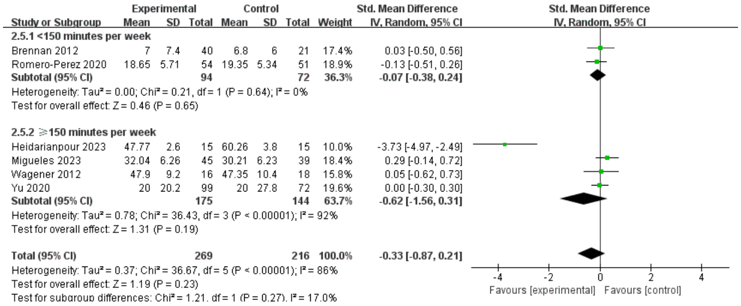

Figure S19.6. Forest plot of subgroup analyses by intervention duration

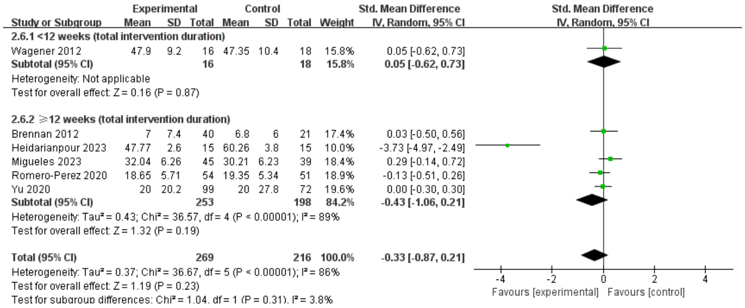

Figure S19.7. Forest plot of subgroup analyses by region

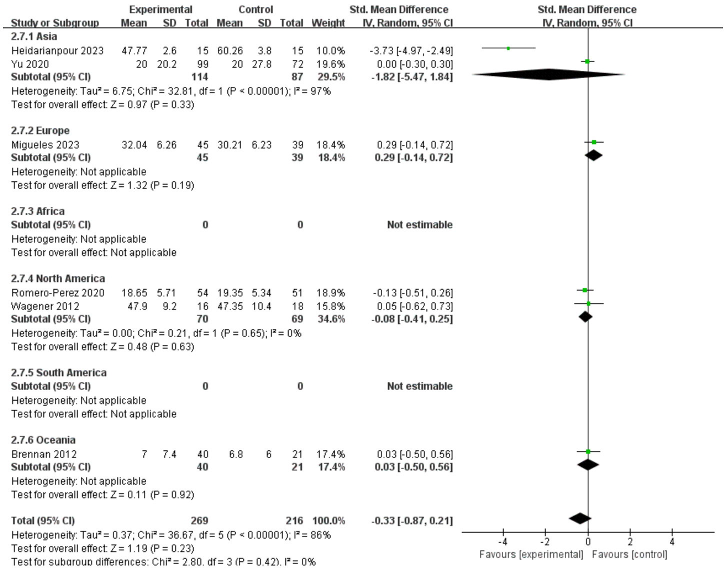

Study or Subgroup	Experimental Mean	Experimental SD	Experimental Total	Control Mean	Control SD	Control Total	Weight	Std. Mean Difference IV, Random, 95% CI
**2.7.1 Asia**								
Heidarianpour 2023	47.77	2.6	15	60.26	3.8	15	10.0%	-3.73 [-4.97, -2.49]
Yu 2020	20	20.2	99	20	27.8	72	19.6%	0.00 [-0.30, 0.30]
**Subtotal (95% CI)**			**114**			**87**	**29.5%**	**-1.82 [-5.47, 1.84]**
Heterogeneity: Tau² = 6.75; Chi² = 32.81, df = 1 (P < 0.00001); I² = 97%								
Test for overall effect: Z = 0.97 (P = 0.33)								
**2.7.2 Europe**								
Migueles 2023	32.04	6.26	45	30.21	6.23	39	18.4%	0.29 [-0.14, 0.72]
**Subtotal (95% CI)**			**45**			**39**	**18.4%**	**0.29 [-0.14, 0.72]**
Heterogeneity: Not applicable								
Test for overall effect: Z = 1.32 (P = 0.19)								
**2.7.3 Africa**								
**Subtotal (95% CI)**			**0**			**0**		**Not estimable**
Heterogeneity: Not applicable								
Test for overall effect: Not applicable								
**2.7.4 North America**								
Romero-Perez 2020	18.65	5.71	54	19.35	5.34	51	18.9%	-0.13 [-0.51, 0.26]
Wagener 2012	47.9	9.2	16	47.35	10.4	18	15.8%	0.05 [-0.62, 0.73]
**Subtotal (95% CI)**			**70**			**69**	**34.6%**	**-0.08 [-0.41, 0.25]**
Heterogeneity: Tau² = 0.00; Chi² = 0.21, df = 1 (P = 0.65); I² = 0%								
Test for overall effect: Z = 0.48 (P = 0.63)								
**2.7.5 South America**								
**Subtotal (95% CI)**			**0**			**0**		**Not estimable**
Heterogeneity: Not applicable								
Test for overall effect: Not applicable								
**2.7.6 Oceania**								
Brennan 2012	7	7.4	40	6.8	6	21	17.4%	0.03 [-0.50, 0.56]
**Subtotal (95% CI)**			**40**			**21**	**17.4%**	**0.03 [-0.50, 0.56]**
Heterogeneity: Not applicable								
Test for overall effect: Z = 0.11 (P = 0.92)								
**Total (95% CI)**			**269**			**216**	**100.0%**	**-0.33 [-0.87, 0.21]**
Heterogeneity: Tau² = 0.37; Chi² = 36.67, df = 5 (P < 0.00001); I² = 86%								
Test for overall effect: Z = 1.19 (P = 0.23)								
Test for subgroup differences: Chi² = 2.80, df = 3 (P = 0.42), I² = 0%								

Figure S19.8. Forest plot of subgroup analyses by economic status

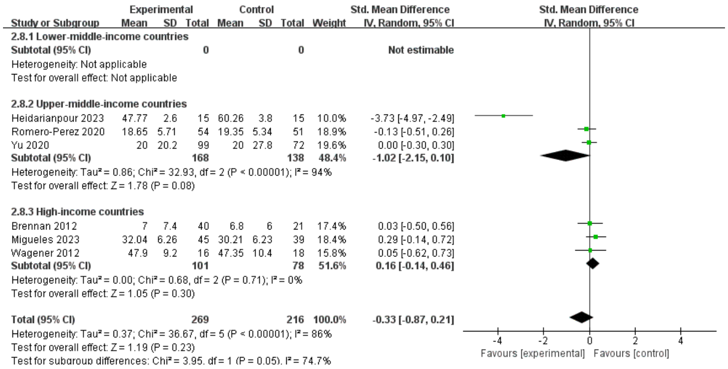

Study or Subgroup	Experimental Mean	Experimental SD	Experimental Total	Control Mean	Control SD	Control Total	Weight	Std. Mean Difference IV, Random, 95% CI
**2.8.1 Lower-middle-income countries**								
**Subtotal (95% CI)**			**0**			**0**		**Not estimable**
Heterogeneity: Not applicable								
Test for overall effect: Not applicable								
**2.8.2 Upper-middle-income countries**								
Heidarianpour 2023	47.77	2.6	15	60.26	3.8	15	10.0%	-3.73 [-4.97, -2.49]
Romero-Perez 2020	18.65	5.71	54	19.35	5.34	51	18.9%	-0.13 [-0.51, 0.26]
Yu 2020	20	20.2	99	20	27.8	72	19.6%	0.00 [-0.30, 0.30]
**Subtotal (95% CI)**			**168**			**138**	**48.4%**	**-1.02 [-2.15, 0.10]**
Heterogeneity: Tau² = 0.86; Chi² = 32.93, df = 2 (P < 0.00001); I² = 94%								
Test for overall effect: Z = 1.78 (P = 0.08)								
**2.8.3 High-income countries**								
Brennan 2012	7	7.4	40	6.8	6	21	17.4%	0.03 [-0.50, 0.56]
Migueles 2023	32.04	6.26	45	30.21	6.23	39	18.4%	0.29 [-0.14, 0.72]
Wagener 2012	47.9	9.2	16	47.35	10.4	18	15.8%	0.05 [-0.62, 0.73]
**Subtotal (95% CI)**			**101**			**78**	**51.6%**	**0.16 [-0.14, 0.46]**
Heterogeneity: Tau² = 0.00; Chi² = 0.68, df = 2 (P = 0.71); I² = 0%								
Test for overall effect: Z = 1.05 (P = 0.30)								
**Total (95% CI)**			**269**			**216**	**100.0%**	**-0.33 [-0.87, 0.21]**
Heterogeneity: Tau² = 0.37; Chi² = 36.67, df = 5 (P < 0.00001); I² = 86%								
Test for overall effect: Z = 1.19 (P = 0.23)								
Test for subgroup differences: Chi² = 3.95, df = 1 (P = 0.05), I² = 74.7%								

Figure S19.9. Forest plot of subgroup analyses by weight status

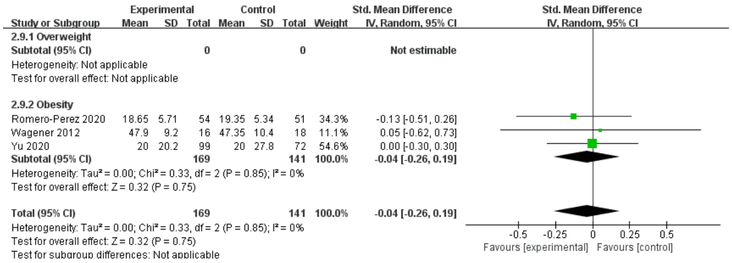

Study or Subgroup	Experimental Mean	Experimental SD	Experimental Total	Control Mean	Control SD	Control Total	Weight	Std. Mean Difference IV, Random, 95% CI
**2.9.1 Overweight**								
**Subtotal (95% CI)**			**0**			**0**		**Not estimable**
Heterogeneity: Not applicable								
Test for overall effect: Not applicable								
**2.9.2 Obesity**								
Romero-Perez 2020	18.65	5.71	54	19.35	5.34	51	34.3%	-0.13 [-0.51, 0.26]
Wagener 2012	47.9	9.2	16	47.35	10.4	18	11.1%	0.05 [-0.62, 0.73]
Yu 2020	20	20.2	99	20	27.8	72	54.6%	0.00 [-0.30, 0.30]
**Subtotal (95% CI)**			**169**			**141**	**100.0%**	**-0.04 [-0.26, 0.19]**
Heterogeneity: Tau² = 0.00; Chi² = 0.33, df = 2 (P = 0.85); I² = 0%								
Test for overall effect: Z = 0.32 (P = 0.75)								
**Total (95% CI)**			**169**			**141**	**100.0%**	**-0.04 [-0.26, 0.19]**
Heterogeneity: Tau² = 0.00; Chi² = 0.33, df = 2 (P = 0.85); I² = 0%								
Test for overall effect: Z = 0.32 (P = 0.75)								
Test for subgroup differences: Not applicable								

Figure S19.10. Forest plot of subgroup analyses by sex

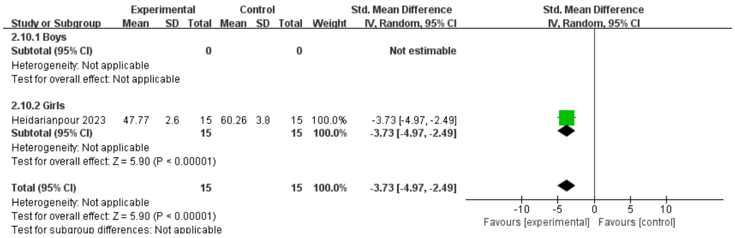

Study or Subgroup	Experimental Mean	Experimental SD	Experimental Total	Control Mean	Control SD	Control Total	Weight	Std. Mean Difference IV, Random, 95% CI
**2.10.1 Boys**								
**Subtotal (95% CI)**			**0**			**0**		**Not estimable**
Heterogeneity: Not applicable								
Test for overall effect: Not applicable								
**2.10.2 Girls**								
Heidarianpour 2023	47.77	2.6	15	60.26	3.8	15	100.0%	-3.73 [-4.97, -2.49]
**Subtotal (95% CI)**			**15**			**15**	**100.0%**	**-3.73 [-4.97, -2.49]**
Heterogeneity: Not applicable								
Test for overall effect: Z = 5.90 (P < 0.00001)								
**Total (95% CI)**			**15**			**15**	**100.0%**	**-3.73 [-4.97, -2.49]**
Heterogeneity: Not applicable								
Test for overall effect: Z = 5.90 (P < 0.00001)								
Test for subgroup differences: Not applicable								

Figure S20. Forest plots of subgroup analyses for Self-esteem in children and adolescents with overweight or obesity

Figure S20.1. Forest plot of subgroup analyses by type of exercise

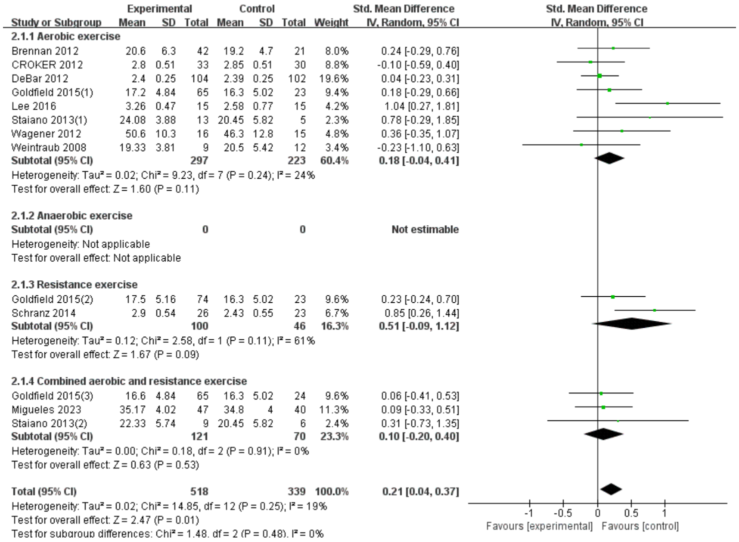

Figure S20.2. Forest plot of subgroup analyses by exercise intensity

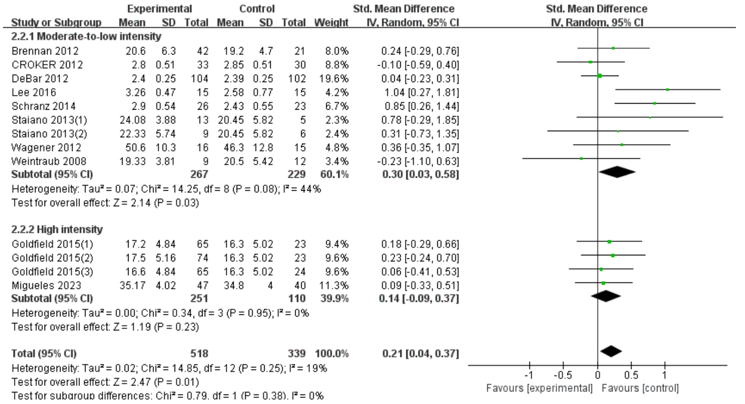

Figure S20.3. Forest plot of subgroup analyses by duration per session

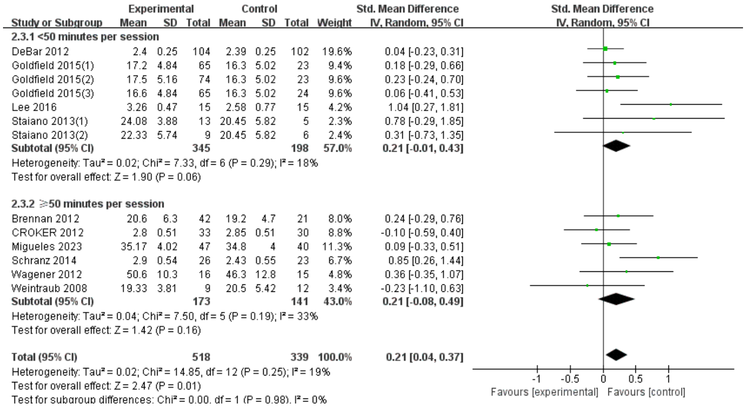

Figure S20.4. Forest plot of subgroup analyses by exercise frequency

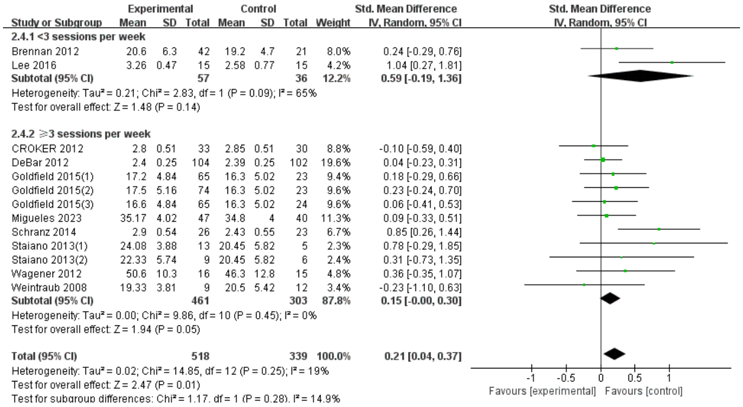

Figure S20.5. Forest plot of subgroup analyses by weekly exercise duration

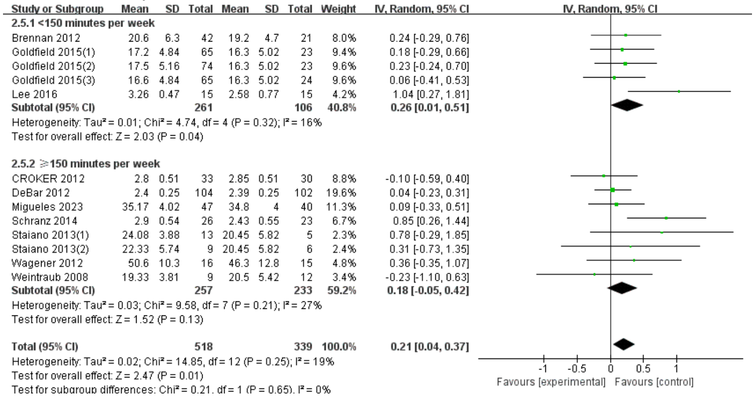

Figure S20.6. Forest plot of subgroup analyses by intervention duration

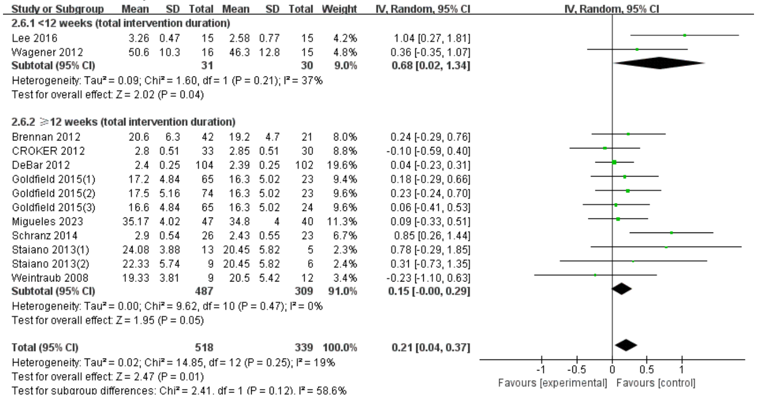

Figure S20.7. Forest plot of subgroup analyses by region

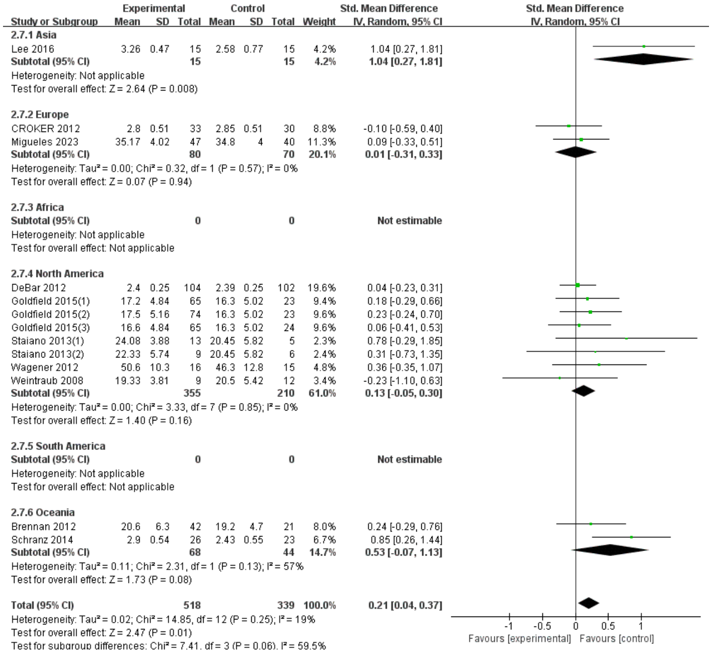

Figure S20.8. Forest plot of subgroup analyses by economic status

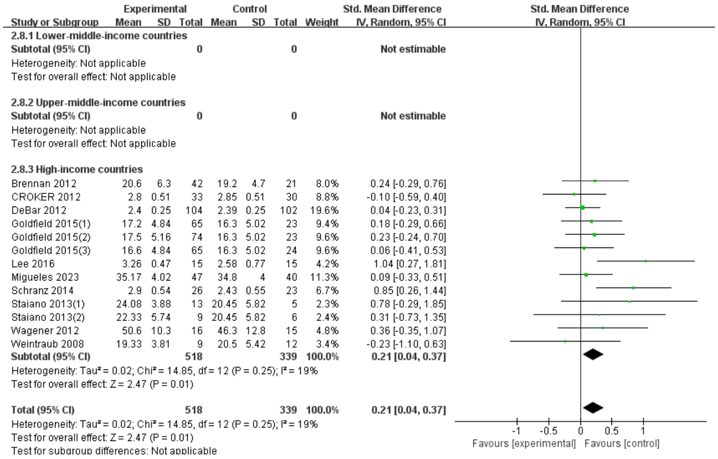

Figure S20.9. Forest plot of subgroup analyses by weight status

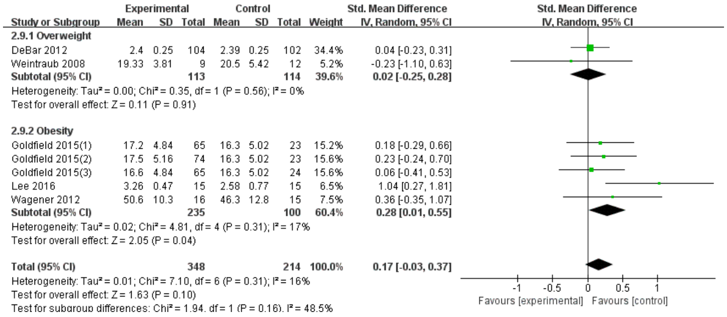

Figure S20.10. Forest plot of subgroup analyses by sex

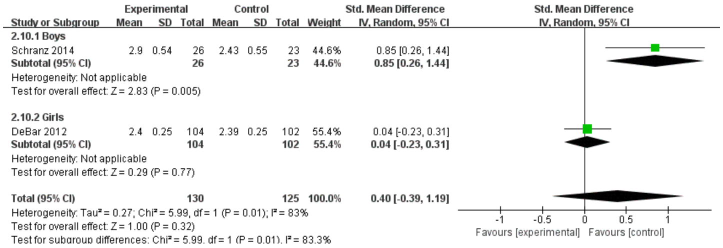

Figure S21. Forest plots of subgroup analyses for Self-worth in children and adolescents with overweight or obesity

Figure S21.1. Forest plot of subgroup analyses by type of exercise

Study or Subgroup	Experimental Mean	Experimental SD	Experimental Total	Control Mean	Control SD	Control Total	Weight	Std. Mean Difference IV, Random, 95% CI
**2.1.1 Aerobic exercise**								
Daley 2006(1)	2.14	0.36	27	1.94	0.25	13	4.7%	0.60 [-0.08, 1.27]
Daley 2006(2)	2.14	0.33	22	1.94	0.25	13	4.3%	0.64 [-0.06, 1.35]
Danielsen 2013	3.26	0.52	22	2.98	0.63	20	5.7%	0.48 [-0.14, 1.09]
Goldfield 2015(1)	13.3	4.03	65	13	4.18	23	9.5%	0.07 [-0.40, 0.55]
Petty 2009(1)	3.2	0.62	42	3.1	0.7	19	7.3%	0.15 [-0.39, 0.70]
Petty 2009(2)	3.1	0.7	42	3.1	0.7	19	7.3%	0.00 [-0.54, 0.54]
Petty 2009(3)	2.9	0.8	27	2.9	0.6	15	5.4%	0.00 [-0.63, 0.63]
Petty 2009(4)	3.4	0.5	28	2.9	0.6	15	5.0%	0.92 [0.26, 1.57]
Williams 2019	3.4	0.63	90	3.3	0.53	85	24.4%	0.17 [-0.13, 0.47]
**Subtotal (95% CI)**			**365**			**222**	**73.7%**	**0.27 [0.08, 0.45]**
Heterogeneity: Tau^2 = 0.01; Chi^2 = 8.99, df = 8 (P = 0.34); I^2 = 11%								
Test for overall effect: Z = 2.80 (P = 0.005)								
**2.1.2 Anaerobic exercise**								
**Subtotal (95% CI)**			**0**			**0**		**Not estimable**
Heterogeneity: Not applicable								
Test for overall effect: Not applicable								
**2.1.3 Resistance exercise**								
Goldfield 2015(2)	14.1	5.16	74	13	4.18	23	9.8%	0.22 [-0.25, 0.69]
Schranz 2014	2.51	0.66	26	2.29	0.57	23	6.7%	0.35 [-0.22, 0.92]
**Subtotal (95% CI)**			**100**			**46**	**16.5%**	**0.27 [-0.09, 0.63]**
Heterogeneity: Tau^2 = 0.00; Chi^2 = 0.12, df = 1 (P = 0.73); I^2 = 0%								
Test for overall effect: Z = 1.48 (P = 0.14)								
**2.1.4 Combined aerobic and resistance exercise**								
Goldfield 2015(3)	13.5	4.03	65	13	4.18	24	9.8%	0.12 [-0.35, 0.59]
**Subtotal (95% CI)**			**65**			**24**	**9.8%**	**0.12 [-0.35, 0.59]**
Heterogeneity: Not applicable								
Test for overall effect: Z = 0.51 (P = 0.61)								
**Total (95% CI)**			**530**			**292**	**100.0%**	**0.25 [0.10, 0.39]**
Heterogeneity: Tau^2 = 0.00; Chi^2 = 9.41, df = 11 (P = 0.58); I^2 = 0%								
Test for overall effect: Z = 3.28 (P = 0.001)								
Test for subgroup differences: Chi^2 = 0.33, df = 2 (P = 0.85), I^2 = 0%								

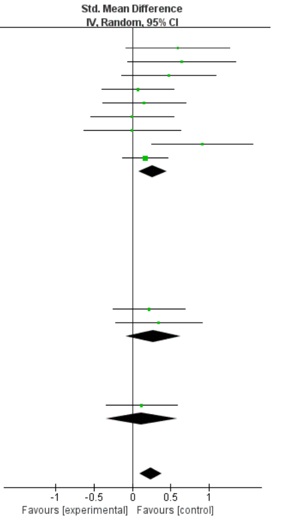

Figure S21.2. Forest plot of subgroup analyses by exercise intensity

Study or Subgroup	Experimental Mean	Experimental SD	Experimental Total	Control Mean	Control SD	Control Total	Weight	Std. Mean Difference IV, Random, 95% CI
**2.2.1 Moderate-to-low intensity**								
Daley 2006(1)	2.14	0.36	27	1.94	0.25	13	4.7%	0.60 [-0.08, 1.27]
Daley 2006(2)	2.14	0.33	22	1.94	0.25	13	4.3%	0.64 [-0.06, 1.35]
Danielsen 2013	3.26	0.52	22	2.98	0.63	20	5.7%	0.48 [-0.14, 1.09]
Petty 2009(1)	3.2	0.62	42	3.1	0.7	19	7.3%	0.15 [-0.39, 0.70]
Petty 2009(3)	2.9	0.8	27	2.9	0.6	15	5.4%	0.00 [-0.63, 0.63]
Schranz 2014	2.51	0.66	26	2.29	0.57	23	6.7%	0.35 [-0.22, 0.92]
**Subtotal (95% CI)**			**166**			**103**	**34.2%**	**0.34 [0.09, 0.60]**
Heterogeneity: Tau^2 = 0.00; Chi^2 = 3.03, df = 5 (P = 0.70); I^2 = 0%								
Test for overall effect: Z = 2.69 (P = 0.007)								
**2.2.2 High intensity**								
Goldfield 2015(1)	13.3	4.03	65	13	4.18	23	9.5%	0.07 [-0.40, 0.55]
Goldfield 2015(2)	14.1	5.16	74	13	4.18	23	9.8%	0.22 [-0.25, 0.69]
Goldfield 2015(3)	13.5	4.03	65	13	4.18	24	9.8%	0.12 [-0.35, 0.59]
Petty 2009(2)	3.1	0.7	42	3.1	0.7	19	7.3%	0.00 [-0.54, 0.54]
Petty 2009(4)	3.4	0.5	28	2.9	0.6	15	5.0%	0.92 [0.26, 1.57]
Williams 2019	3.4	0.63	90	3.3	0.53	85	24.4%	0.17 [-0.13, 0.47]
**Subtotal (95% CI)**			**364**			**189**	**65.8%**	**0.20 [0.00, 0.39]**
Heterogeneity: Tau^2 = 0.01; Chi^2 = 5.47, df = 5 (P = 0.36); I^2 = 9%								
Test for overall effect: Z = 2.00 (P = 0.04)								
**Total (95% CI)**			**530**			**292**	**100.0%**	**0.25 [0.10, 0.39]**
Heterogeneity: Tau^2 = 0.00; Chi^2 = 9.41, df = 11 (P = 0.58); I^2 = 0%								
Test for overall effect: Z = 3.28 (P = 0.001)								
Test for subgroup differences: Chi^2 = 0.85, df = 1 (P = 0.36), I^2 = 0%								

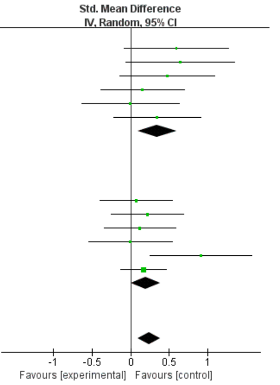

Figure S21.3. Forest plot of subgroup analyses by duration per session

Study or Subgroup	Experimental Mean	Experimental SD	Experimental Total	Control Mean	Control SD	Control Total	Weight	Std. Mean Difference IV, Random, 95% CI
**2.3.1 <50 minutes per session**								
Daley 2006(1)	2.14	0.36	27	1.94	0.25	13	4.7%	0.60 [-0.08, 1.27]
Daley 2006(2)	2.14	0.33	22	1.94	0.25	13	4.3%	0.64 [-0.06, 1.35]
Danielsen 2013	3.26	0.52	22	2.98	0.63	20	5.7%	0.48 [-0.14, 1.09]
Goldfield 2015(1)	13.3	4.03	65	13	4.18	23	9.5%	0.07 [-0.40, 0.55]
Goldfield 2015(2)	14.1	5.16	74	13	4.18	23	9.8%	0.22 [-0.25, 0.69]
Goldfield 2015(3)	13.5	4.03	65	13	4.18	24	9.8%	0.12 [-0.35, 0.59]
Petty 2009(1)	3.2	0.62	42	3.1	0.7	19	7.3%	0.15 [-0.39, 0.70]
Petty 2009(2)	3.1	0.7	42	3.1	0.7	19	7.3%	0.00 [-0.54, 0.54]
Petty 2009(3)	2.9	0.8	27	2.9	0.6	15	5.4%	0.00 [-0.63, 0.63]
Petty 2009(4)	3.4	0.5	28	2.9	0.6	15	5.0%	0.92 [0.26, 1.57]
Williams 2019	3.4	0.63	90	3.3	0.53	85	24.4%	0.17 [-0.13, 0.47]
**Subtotal (95% CI)**			**504**			**269**	**93.3%**	**0.24 [0.09, 0.39]**
Heterogeneity: Tau^2 = 0.00; Chi^2 = 9.28, df = 10 (P = 0.51); I^2 = 0%								
Test for overall effect: Z = 3.07 (P = 0.002)								
**2.3.2 ⩾50 minutes per session**								
Schranz 2014	2.51	0.66	26	2.29	0.57	23	6.7%	0.35 [-0.22, 0.92]
**Subtotal (95% CI)**			**26**			**23**	**6.7%**	**0.35 [-0.22, 0.92]**
Heterogeneity: Not applicable								
Test for overall effect: Z = 1.21 (P = 0.23)								
**Total (95% CI)**			**530**			**292**	**100.0%**	**0.25 [0.10, 0.39]**
Heterogeneity: Tau^2 = 0.00; Chi^2 = 9.41, df = 11 (P = 0.58); I^2 = 0%								
Test for overall effect: Z = 3.28 (P = 0.001)								
Test for subgroup differences: Chi^2 = 0.14, df = 1 (P = 0.71), I^2 = 0%								

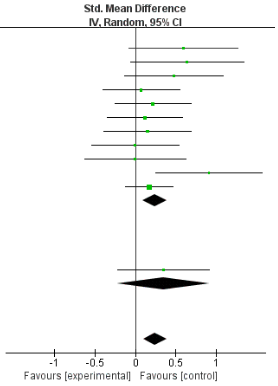

Figure S21.4. Forest plot of subgroup analyses by exercise frequency

Study or Subgroup	Experimental Mean	Experimental SD	Experimental Total	Control Mean	Control SD	Control Total	Weight	Std. Mean Difference IV, Random, 95% CI
**2.4.1 <3 sessions per week**								
**Subtotal (95% CI)**			**0**			**0**		**Not estimable**
Heterogeneity: Not applicable								
Test for overall effect: Not applicable								
**2.4.2 ⩾3 sessions per week**								
Daley 2006(1)	2.14	0.36	27	1.94	0.25	13	4.7%	0.60 [-0.08, 1.27]
Daley 2006(2)	2.14	0.33	22	1.94	0.25	13	4.3%	0.64 [-0.06, 1.35]
Danielsen 2013	3.26	0.52	22	2.98	0.63	20	5.7%	0.48 [-0.14, 1.09]
Goldfield 2015(1)	13.3	4.03	65	13	4.18	23	9.5%	0.07 [-0.40, 0.55]
Goldfield 2015(2)	14.1	5.16	74	13	4.18	23	9.8%	0.22 [-0.25, 0.69]
Goldfield 2015(3)	13.5	4.03	65	13	4.18	24	9.8%	0.12 [-0.35, 0.59]
Petty 2009(1)	3.2	0.62	42	3.1	0.7	19	7.3%	0.15 [-0.39, 0.70]
Petty 2009(2)	3.1	0.7	42	3.1	0.7	19	7.3%	0.00 [-0.54, 0.54]
Petty 2009(3)	2.9	0.8	27	2.9	0.6	15	5.4%	0.00 [-0.63, 0.63]
Petty 2009(4)	3.4	0.5	28	2.9	0.6	15	5.0%	0.92 [0.26, 1.57]
Schranz 2014	2.51	0.66	26	2.29	0.57	23	6.7%	0.35 [-0.22, 0.92]
Williams 2019	3.4	0.63	90	3.3	0.53	85	24.4%	0.17 [-0.13, 0.47]
**Subtotal (95% CI)**			**530**			**292**	**100.0%**	**0.25 [0.10, 0.39]**
Heterogeneity: Tau^2 = 0.00; Chi^2 = 9.41, df = 11 (P = 0.58); I^2 = 0%								
Test for overall effect: Z = 3.28 (P = 0.001)								
**Total (95% CI)**			**530**			**292**	**100.0%**	**0.25 [0.10, 0.39]**
Heterogeneity: Tau^2 = 0.00; Chi^2 = 9.41, df = 11 (P = 0.58); I^2 = 0%								
Test for overall effect: Z = 3.28 (P = 0.001)								
Test for subgroup differences: Not applicable								

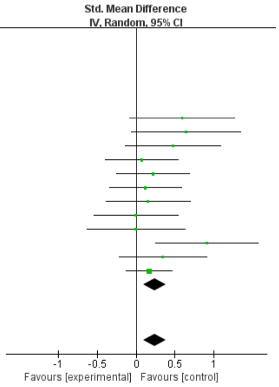

Figure S21.5. Forest plot of subgroup analyses by weekly exercise duration

Study or Subgroup	Experimental Mean	Experimental SD	Experimental Total	Control Mean	Control SD	Control Total	Weight	Std. Mean Difference IV, Random, 95% CI
**2.5.1 <150 minutes per week**								
Daley 2006(1)	2.14	0.36	27	1.94	0.25	13	4.7%	0.60 [-0.08, 1.27]
Daley 2006(2)	2.14	0.33	22	1.94	0.25	13	4.3%	0.64 [-0.06, 1.35]
Goldfield 2015(1)	13.3	4.03	65	13	4.18	23	9.5%	0.07 [-0.40, 0.55]
Goldfield 2015(2)	14.1	5.16	74	13	4.18	23	9.8%	0.22 [-0.25, 0.69]
Goldfield 2015(3)	13.5	4.03	65	13	4.18	24	9.8%	0.12 [-0.35, 0.59]
Petty 2009(1)	3.2	0.62	42	3.1	0.7	19	7.3%	0.15 [-0.39, 0.70]
Petty 2009(3)	2.9	0.8	27	2.9	0.6	15	5.4%	0.00 [-0.63, 0.63]
**Subtotal (95% CI)**			**322**			**130**	**50.9%**	**0.21 [0.01, 0.42]**
Heterogeneity: Tau^2 = 0.00; Chi^2 = 3.63, df = 6 (P = 0.73); I^2 = 0%								
Test for overall effect: Z = 2.02 (P = 0.04)								
**2.5.2 ⩾150 minutes per week**								
Danielsen 2013	3.26	0.52	22	2.98	0.63	20	5.7%	0.48 [-0.14, 1.09]
Petty 2009(2)	3.1	0.7	42	3.1	0.7	19	7.3%	0.00 [-0.54, 0.54]
Petty 2009(4)	3.4	0.5	28	2.9	0.6	15	5.0%	0.92 [0.26, 1.57]
Schranz 2014	2.51	0.66	26	2.29	0.57	23	6.7%	0.35 [-0.22, 0.92]
Williams 2019	3.4	0.63	90	3.3	0.53	85	24.4%	0.17 [-0.13, 0.47]
**Subtotal (95% CI)**			**208**			**162**	**49.1%**	**0.31 [0.05, 0.58]**
Heterogeneity: Tau^2 = 0.03; Chi^2 = 5.57, df = 4 (P = 0.23); I^2 = 28%								
Test for overall effect: Z = 2.31 (P = 0.02)								
**Total (95% CI)**			**530**			**292**	**100.0%**	**0.25 [0.10, 0.39]**
Heterogeneity: Tau^2 = 0.00; Chi^2 = 9.41, df = 11 (P = 0.58); I^2 = 0%								
Test for overall effect: Z = 3.28 (P = 0.001)								
Test for subgroup differences: Chi^2 = 0.35, df = 1 (P = 0.55), I^2 = 0%								

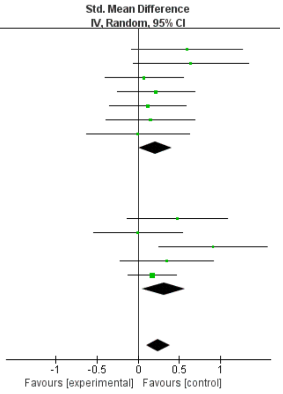

Figure S21.6. Forest plot of subgroup analyses by intervention duration

Study or Subgroup	Experimental Mean	Experimental SD	Experimental Total	Control Mean	Control SD	Control Total	Weight	Std. Mean Difference IV, Random, 95% CI
**2.6.1 <12 weeks (total intervention duration)**								
Daley 2006(1)	2.14	0.36	27	1.94	0.25	13	4.7%	0.60 [-0.08, 1.27]
Daley 2006(2)	2.14	0.33	22	1.94	0.25	13	4.3%	0.64 [-0.06, 1.35]
**Subtotal (95% CI)**			**49**			**26**	**9.1%**	**0.62 [0.13, 1.11]**
Heterogeneity: Tau^2 = 0.00; Chi^2 = 0.01, df = 1 (P = 0.92); I^2 = 0%								
Test for overall effect: Z = 2.49 (P = 0.01)								
**2.6.2 ⩾12 weeks (total intervention duration)**								
Danielsen 2013	3.26	0.52	22	2.98	0.63	20	5.7%	0.48 [-0.14, 1.09]
Goldfield 2015(1)	13.3	4.03	65	13	4.18	23	9.5%	0.07 [-0.40, 0.55]
Goldfield 2015(2)	14.1	5.16	74	13	4.18	23	9.8%	0.22 [-0.25, 0.69]
Goldfield 2015(3)	13.5	4.03	65	13	4.18	24	9.8%	0.12 [-0.35, 0.59]
Petty 2009(1)	3.2	0.62	42	3.1	0.7	19	7.3%	0.15 [-0.39, 0.70]
Petty 2009(2)	3.1	0.7	42	3.1	0.7	19	7.3%	0.00 [-0.54, 0.54]
Petty 2009(3)	2.9	0.8	27	2.9	0.6	15	5.4%	0.00 [-0.63, 0.63]
Petty 2009(4)	3.4	0.5	28	2.9	0.6	15	5.0%	0.92 [0.26, 1.57]
Schranz 2014	2.51	0.66	26	2.29	0.57	23	6.7%	0.35 [-0.22, 0.92]
Williams 2019	3.4	0.63	90	3.3	0.53	85	24.4%	0.17 [-0.13, 0.47]
**Subtotal (95% CI)**			**481**			**266**	**90.9%**	**0.21 [0.05, 0.36]**
Heterogeneity: Tau^2 = 0.00; Chi^2 = 6.93, df = 9 (P = 0.64); I^2 = 0%								
Test for overall effect: Z = 2.65 (P = 0.008)								
**Total (95% CI)**			**530**			**292**	**100.0%**	**0.25 [0.10, 0.39]**
Heterogeneity: Tau^2 = 0.00; Chi^2 = 9.41, df = 11 (P = 0.58); I^2 = 0%								
Test for overall effect: Z = 3.28 (P = 0.001)								
Test for subgroup differences: Chi^2 = 2.48, df = 1 (P = 0.12), I^2 = 59.6%								

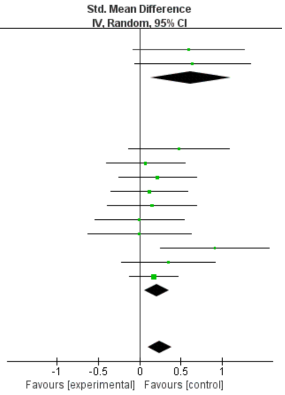

Figure S21.7. Forest plot of subgroup analyses by region

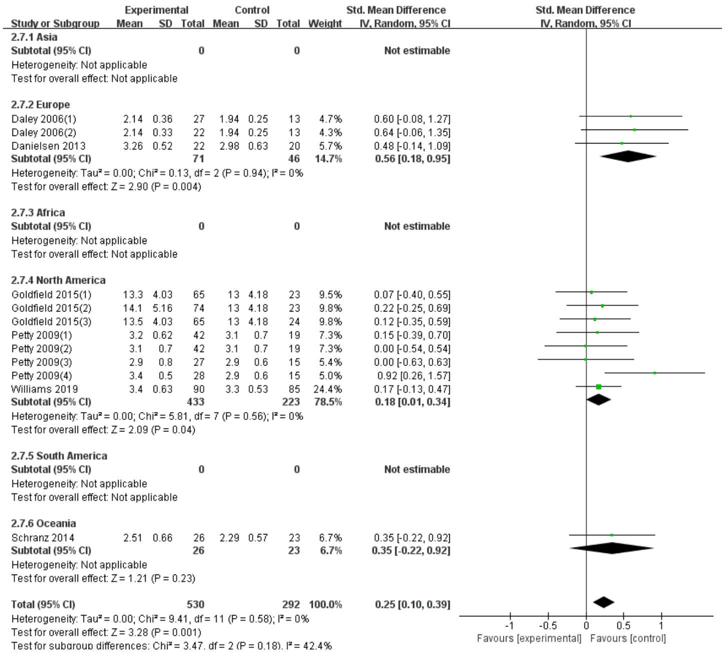

Figure S21.8. Forest plot of subgroup analyses by economic status

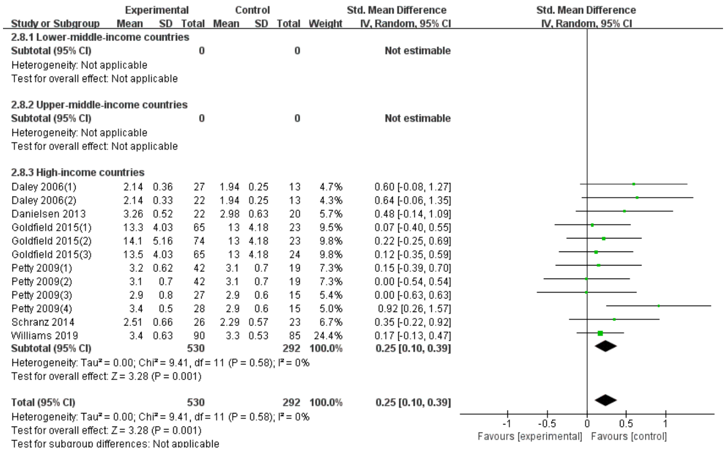

Figure S21.9. Forest plot of subgroup analyses by weight status

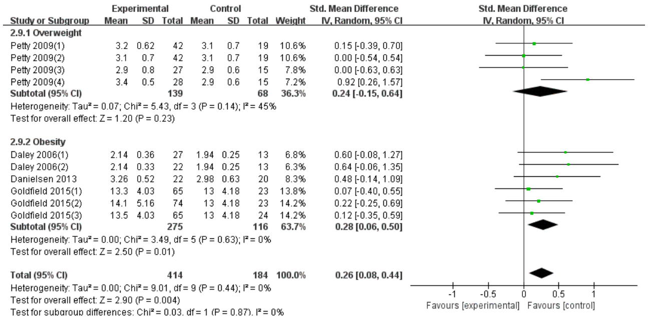

Figure S21.10. Forest plot of subgroup analyses by sex

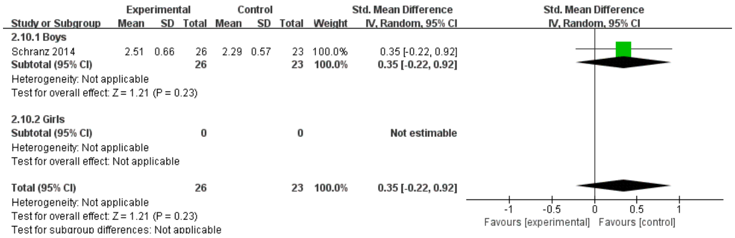

Figure S22. Funnel plots of meta-analysis results for 20 outcome indicators in children and adolescents with overweight or obesity

Figure S22.1. Funnel plot of the effect of exercise on BMI

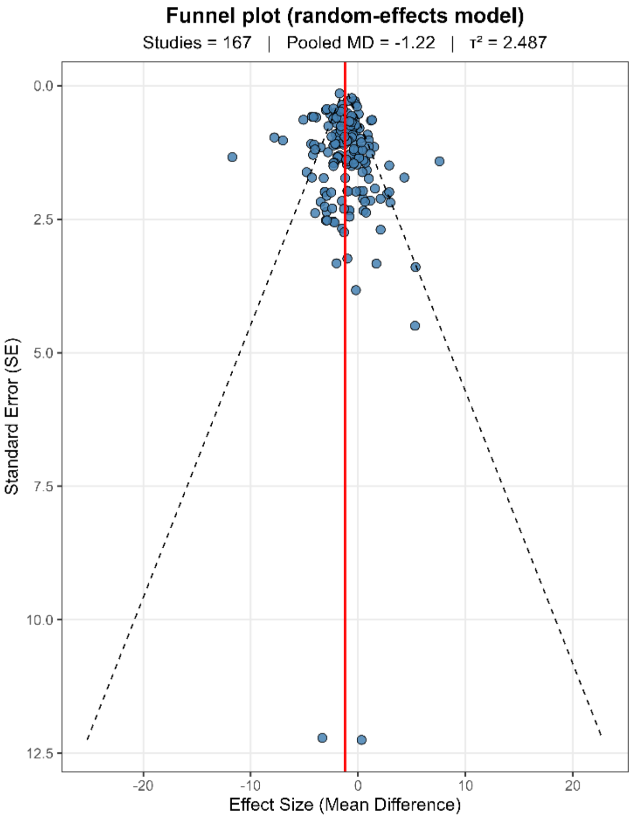

Figure S22.2. Funnel plot of the effect of exercise on WC

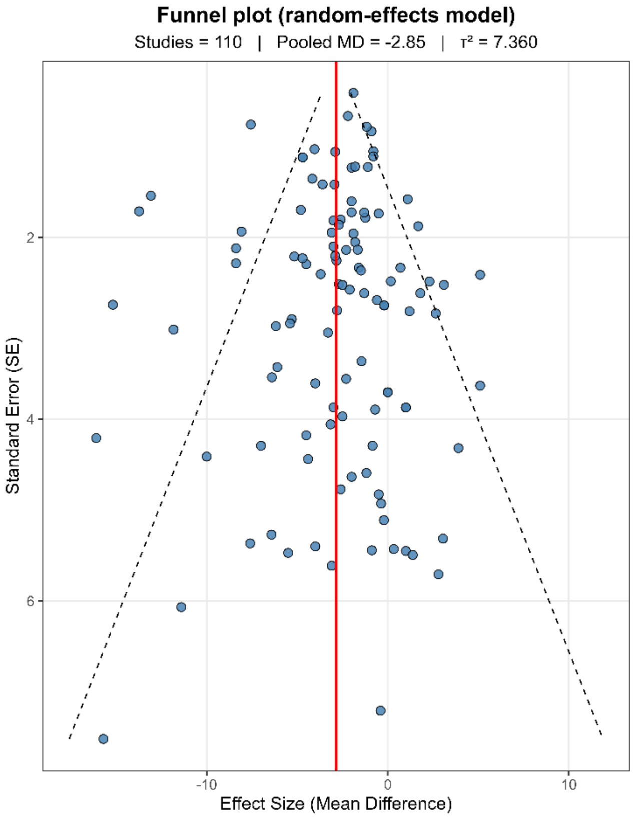

Figure S22.3. Funnel plot of the effect of exercise on BF%

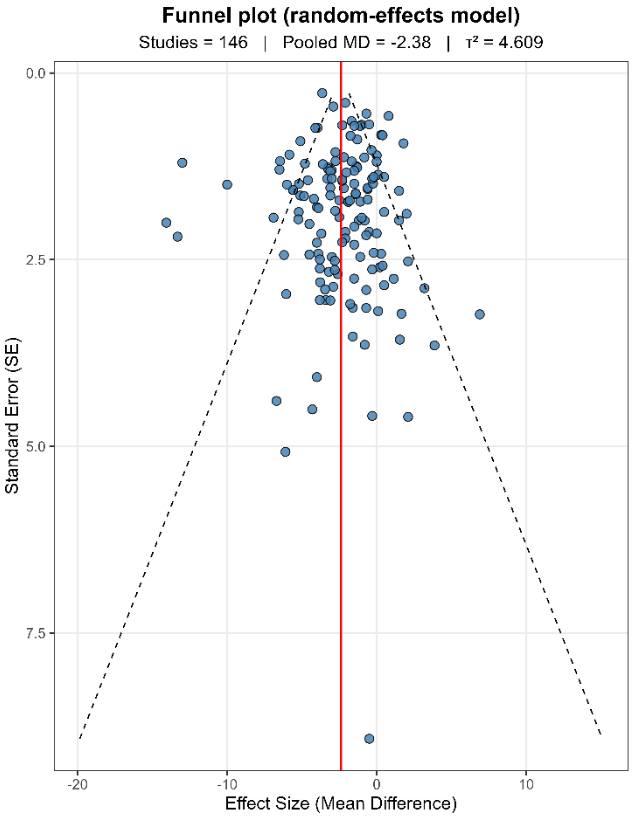

Figure S22.4. Funnel plot of the effect of exercise on VO_2max

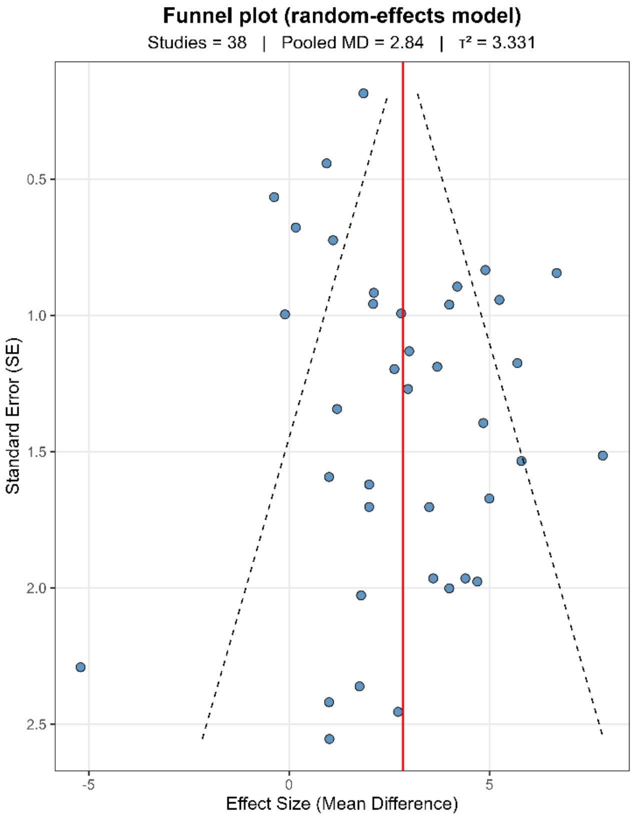

Figure S22.5. Funnel plot of the effect of exercise on VO_2peak

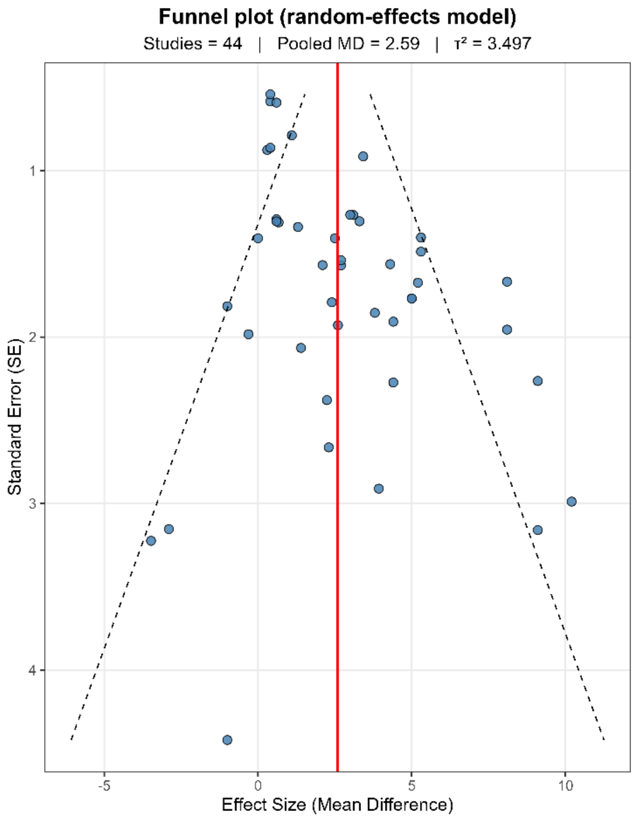

Figure S22.6. Funnel plot of the effect of exercise on HRmax

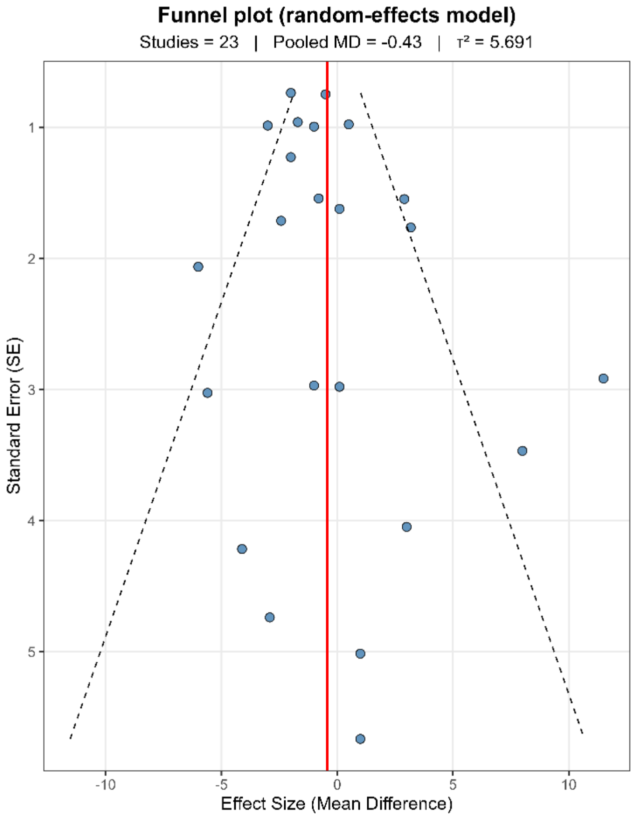

Figure S22.7. Funnel plot of the effect of exercise on HRrest

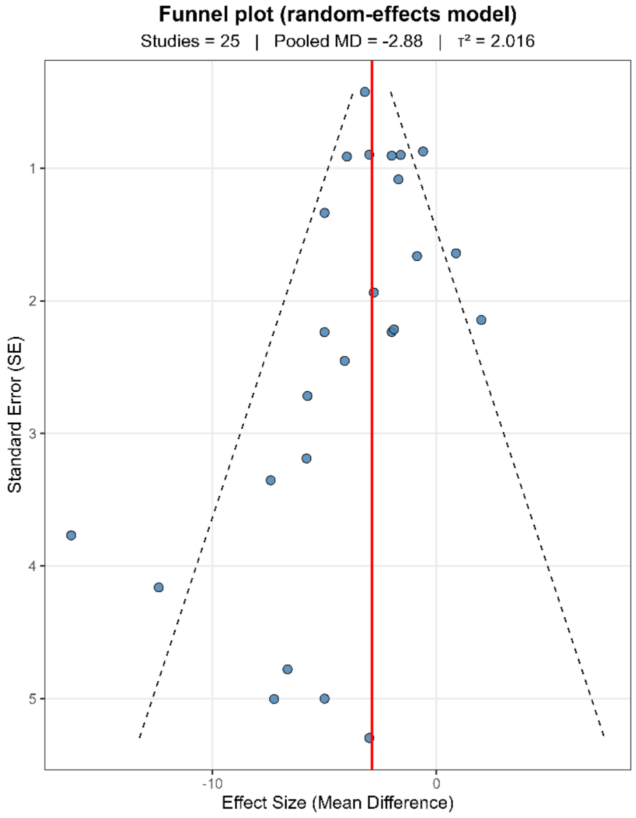

Figure S22.8. Funnel plot of the effect of exercise on SBP

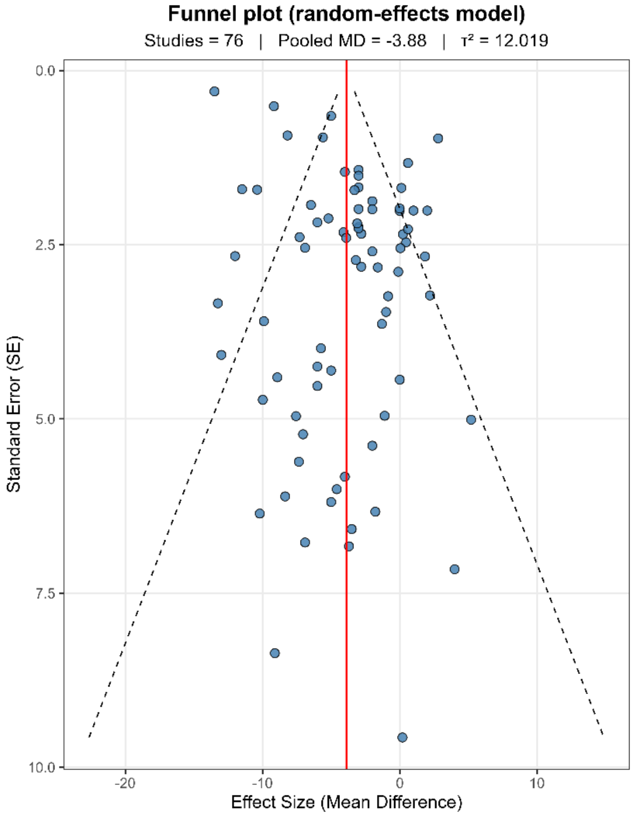

Figure S22.9. Funnel plot of the effect of exercise on DBP

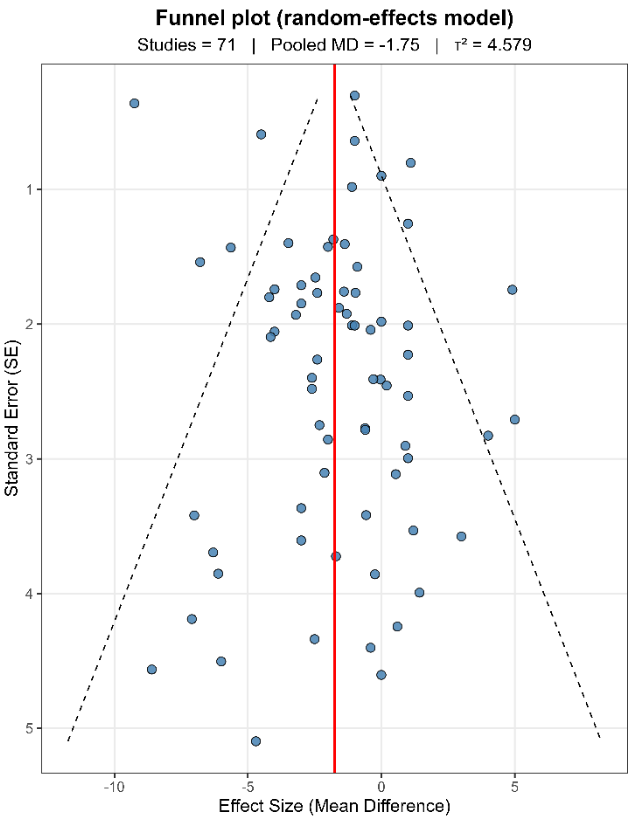

Figure S22.10. Funnel plot of the effect of exercise on TC

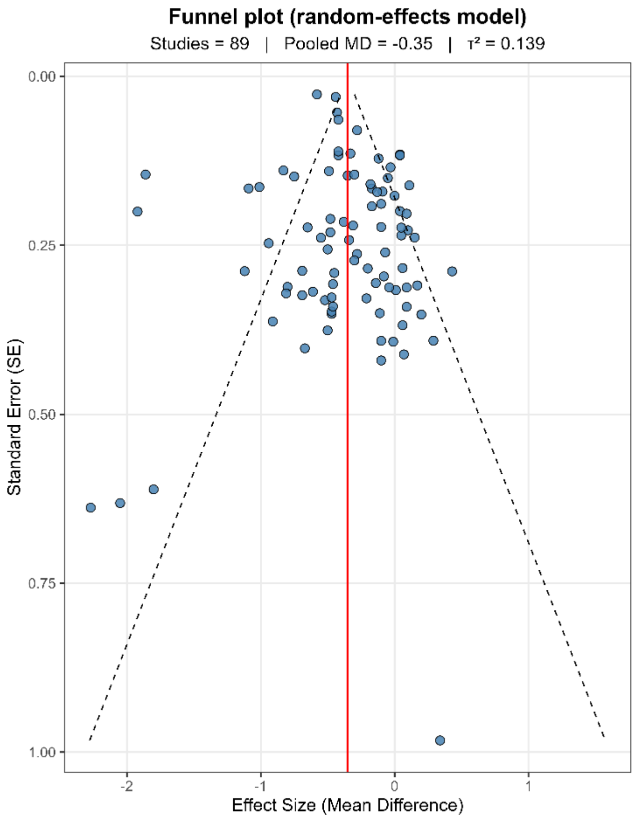

Figure S22.11. Funnel plot of the effect of exercise on TG

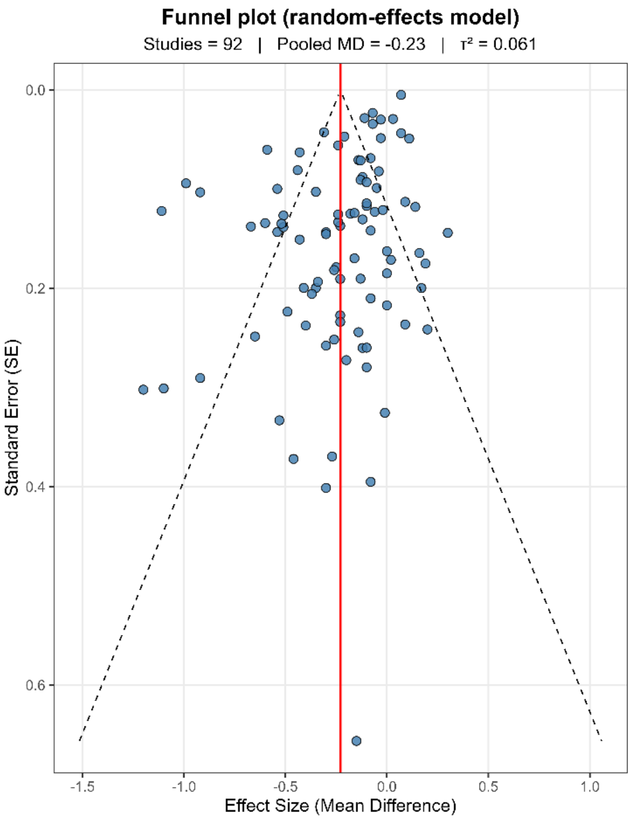

Figure S22.12. Funnel plot of the effect of exercise on HDL-C

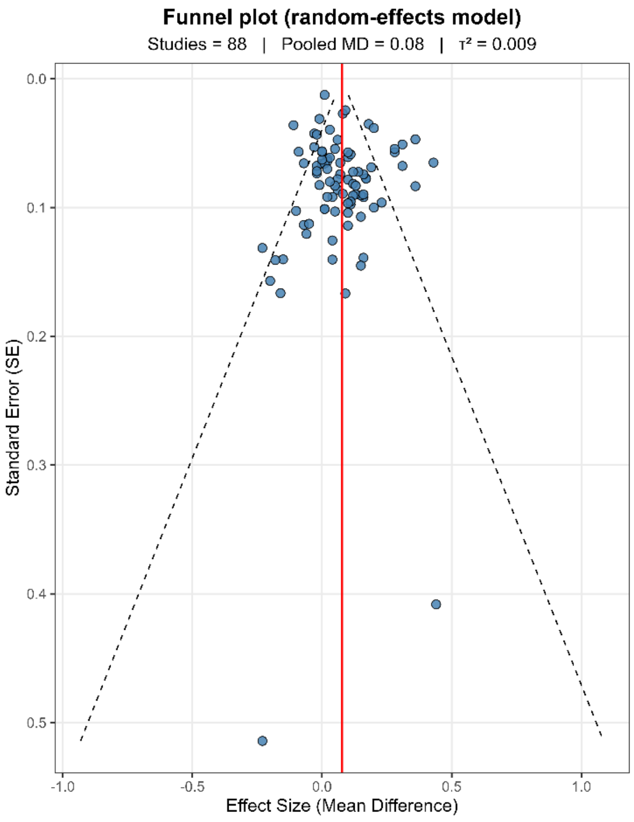

Figure S22.13. Funnel plot of the effect of exercise on LDL-C

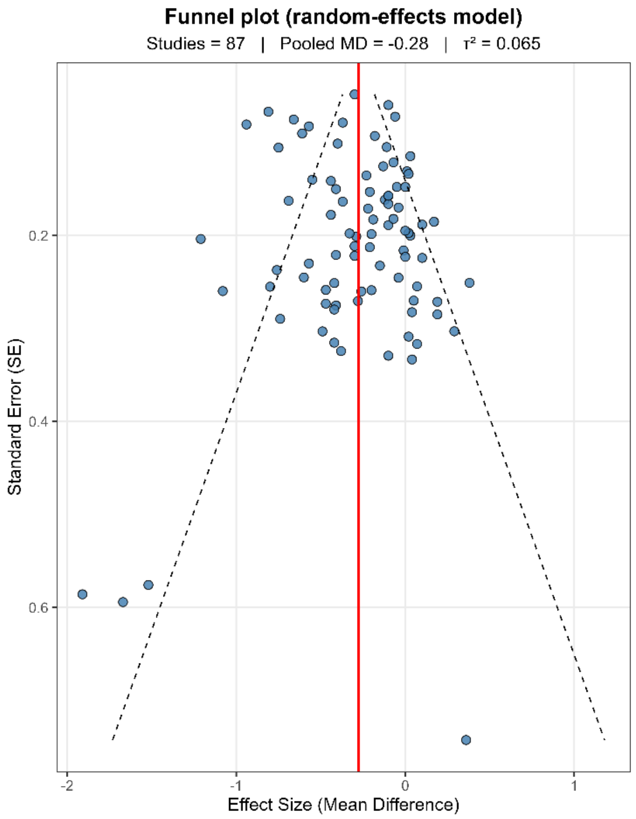

Figure S22.14. Funnel plot of the effect of exercise on FPG

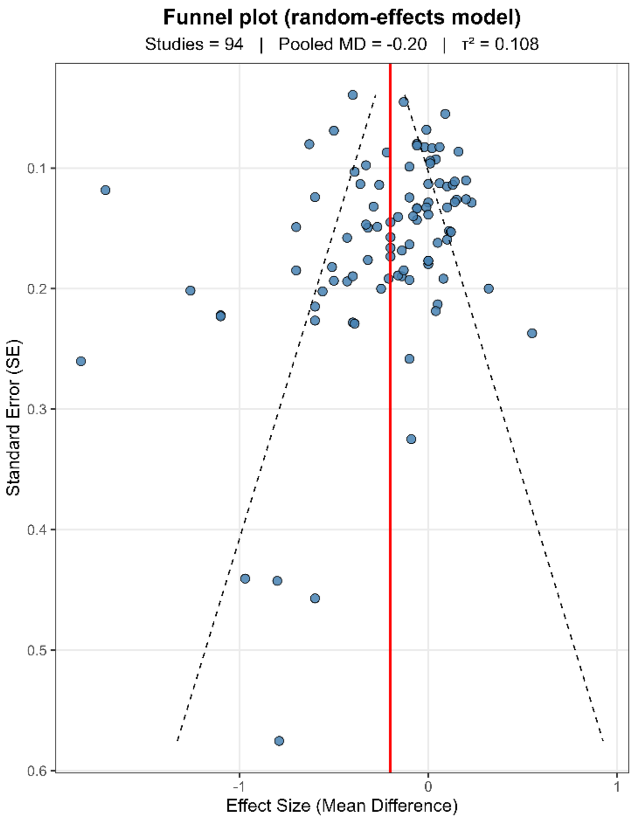

Figure S22.15. Funnel plot of the effect of exercise on HbA1c

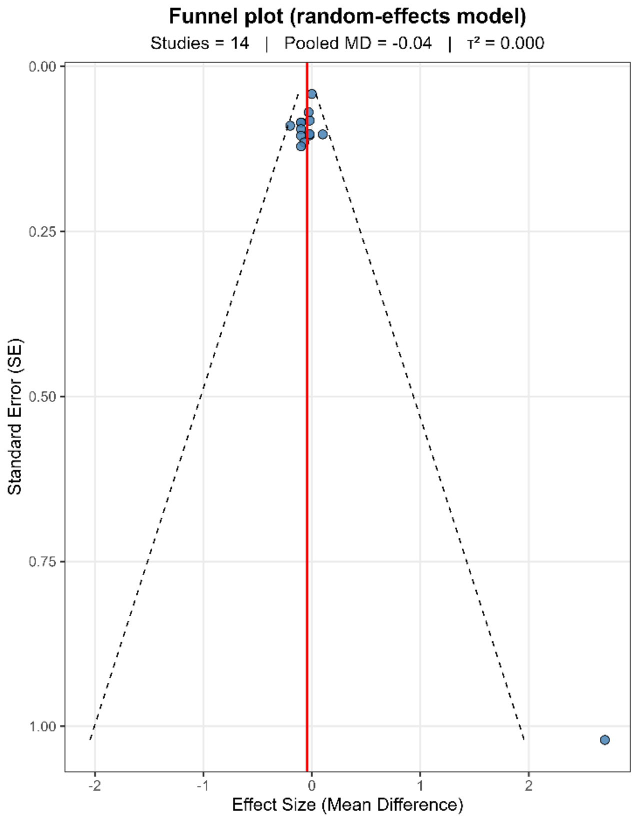

Figure S22.16. Funnel plot of the effect of exercise on FINS

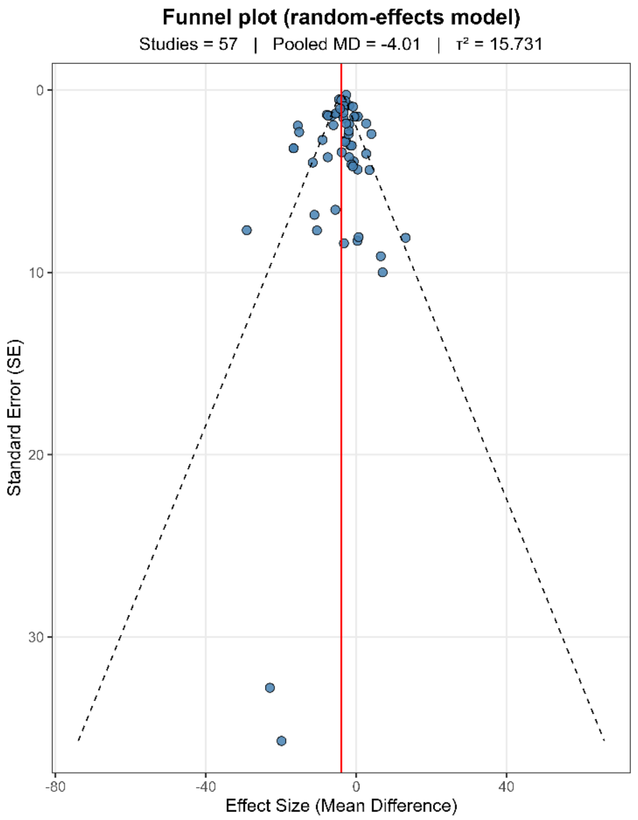

Figure S22.17. Funnel plot of the effect of exercise on Depression

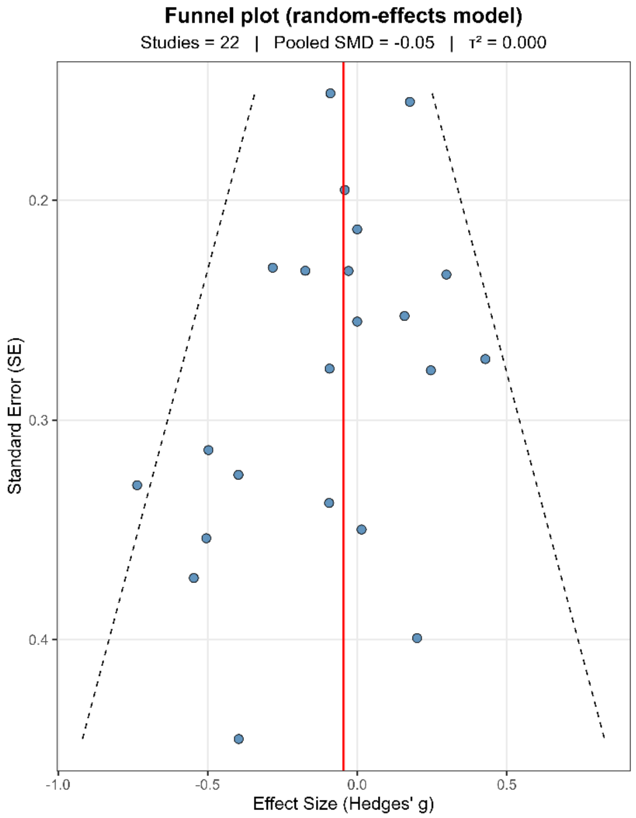

Figure S22.18. Funnel plot of the effect of exercise on Anxiety

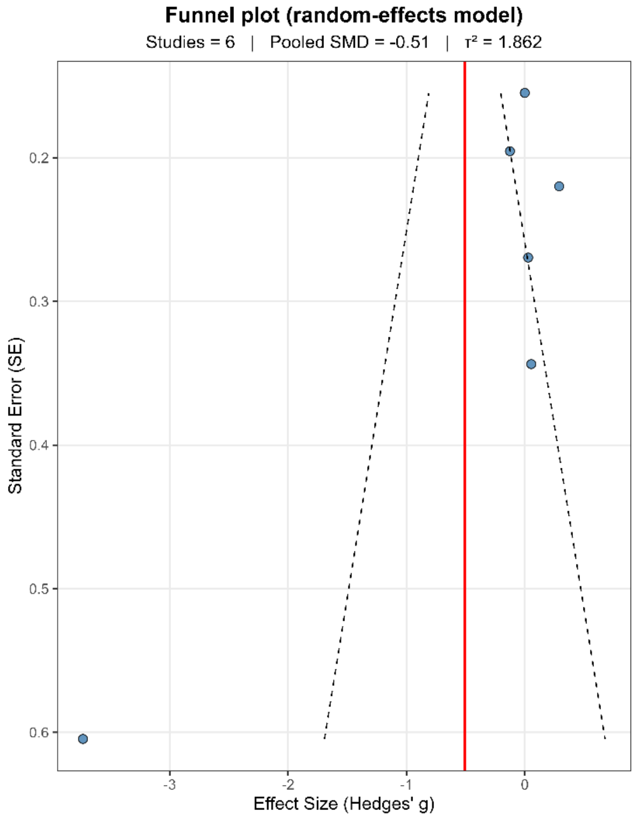

Figure S22.19. Funnel plot of the effect of exercise on Self-esteem

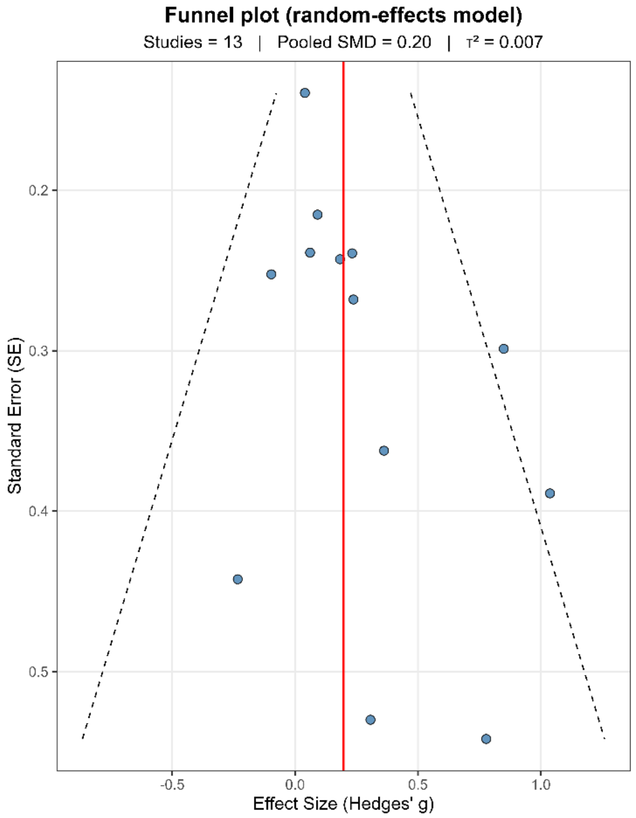

Figure S22.20. Funnel plot of the effect of exercise on Self-worth

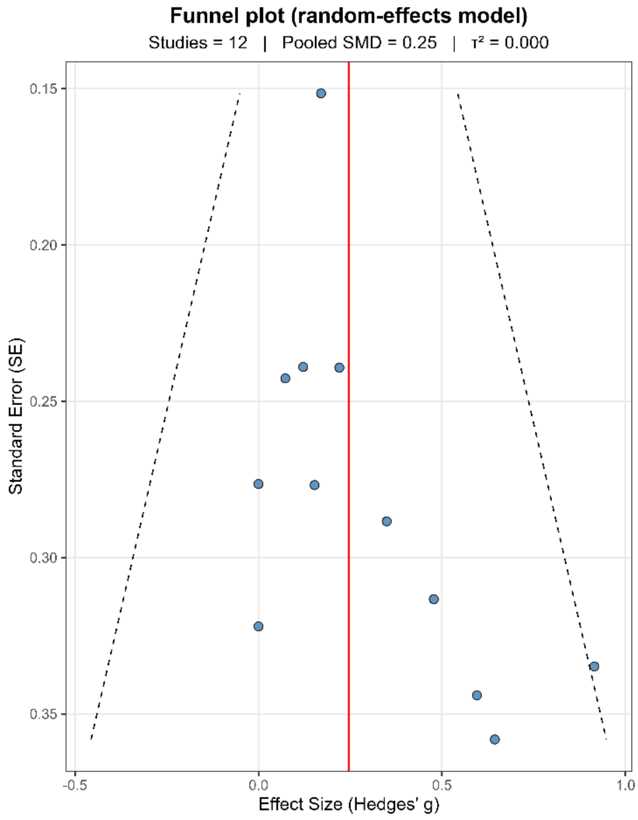

Table S3. Certainty of assessment using GRADE for RCTs

Outcome	Initial rating	Risk of bias	Inconsistency	Indirectness	Imprecision	Publication bias	Quality of the evidence
BMI	High	Serious concern [a]	No serious concern	No serious concern	No serious concern	No serious concern	Moderate
WC	High	Serious concern [a]	No serious concern	No serious concern	No serious concern	No serious concern	Moderate
BF%	High	Serious concern [a]	No serious concern	No serious concern	No serious concern	No serious concern	Moderate
VO_2max	High	No serious concern	Serious concern [b]	No serious concern	No serious concern	No serious concern	Moderate
VO_2peak	High	Serious concern [a]	No serious concern	No serious concern	No serious concern	No serious concern	Moderate
HRmax	High	No serious concern	Serious concern [b]	No serious concern	Serious concern [c]	No serious concern	Low
HRrest	High	No serious concern	No serious concern	No serious concern	No serious concern	No serious concern	High
SBP	High	No serious concern	No serious concern	No serious concern	No serious concern	No serious concern	High
DBP	High	No serious concern	Serious concern [b]	No serious concern	No serious concern	No serious concern	Moderate
TC	High	Serious concern [a]	No serious concern	No serious concern	No serious concern	No serious concern	Moderate
TG	High	Serious concern [a]	No serious concern	No serious concern	No serious concern	No serious concern	Moderate
HDL-C	High	Serious concern [a]	Serious concern [b]	No serious concern	No serious concern	No serious concern	Low
LDL-C	High	Serious concern [a]	No serious concern	No serious concern	No serious concern	No serious concern	Moderate
FPG	High	Serious concern [a]	No serious concern	No serious concern	No serious concern	No serious concern	Moderate
HbA1c	High	No serious concern	No serious concern	No serious concern	Serious concern [c]	No serious concern	Moderate
FINS	High	Serious concern [a]	No serious concern	No serious concern	No serious concern	No serious concern	Moderate
Depression	High	No serious concern	No serious concern	No serious concern	Serious concern [c]	No serious concern	Moderate
Anxiety	High	No serious concern	Serious concern [b]	No serious concern	Serious concern [c]	Serious concern [d]	Very Low
Self-esteem	High	Serious concern [a]	No serious concern	No serious concern	No serious concern	No serious concern	Moderate
Self-worth	High	No serious concern	No serious concern	No serious concern	No serious concern	No serious concern	High

Note: BMI, body mass index; WC, waist circumference; BF%, percentage of body fat; VO_2max, maximal oxygen consumption; VO_2peak, peak oxygen uptake; HRmax, max heart rate; HRrest, resting heart rate; SBP, systolic blood pressure; DBP, diastolic blood pressure; TC, total cholesterol; TG, triglyceride; HDL-C, high-density lipoprotein cholesterol; LDL-C, low-density lipoprotein cholesterol; FPG, fasting plasma glucose; HbA1c, hemoglobin A1c; FINS, fasting insulin

RCTs, randomised controlled trials; GRADE, the Grading of Recommendations, Assessment, Development and Evaluations.

a: Downgraded one level due to some concerns about risk of bias

b: Downgraded one level due to unexplained heterogeneity

c: Downgraded one level due to wide confidence interval crossing the line of no effect

d: Downgraded one level due to suspected publication bias

Figure S23. Revised Cochrane Risk-of-Bias Tool for Randomised Trials summary and author judgments of low, some concerns, and high risk of bias across all included RCTs (K=229)

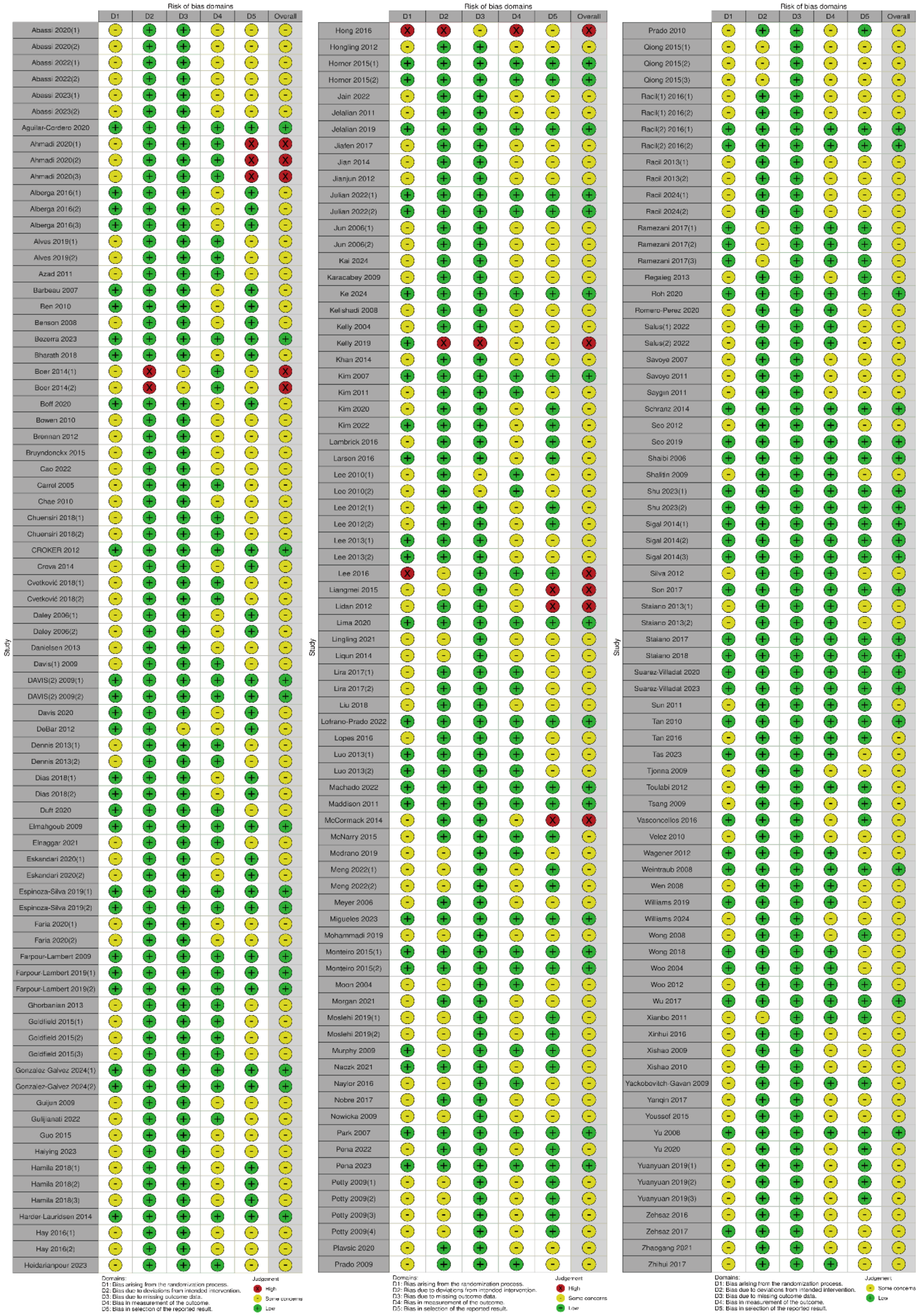

Note: RCT, randomised controlled trial. N indicated the number of the intervention groups.

Figure S24. Review judgment of risk bias for each domain: percentages across all included RCTs (K=229)

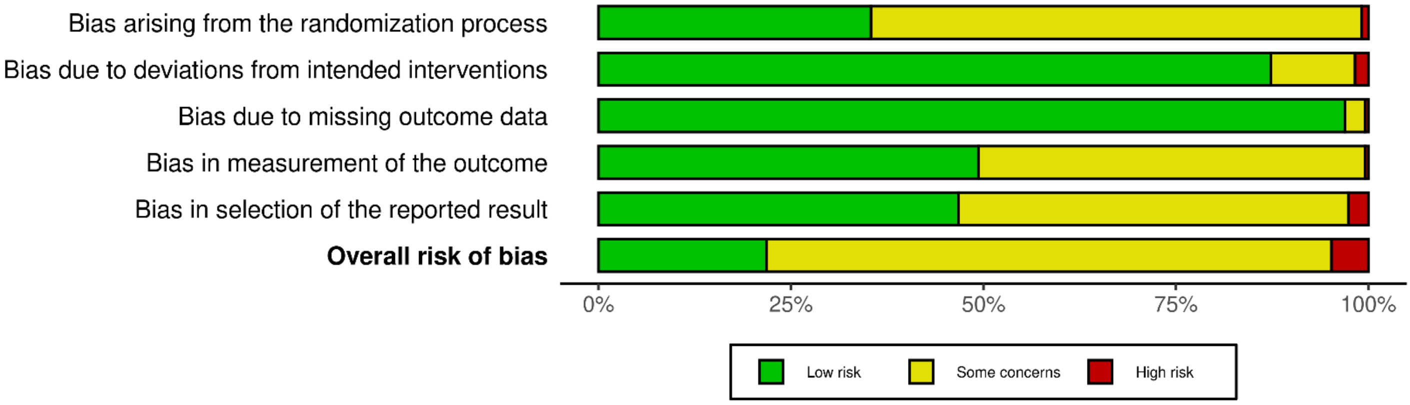

Note: RCTs, randomised controlled trials. N indicated the number of the intervention groups.

Table S4. Egger's test and Trim-and-Fill correction analyses for publication bias across outcomes

Outcomes	Egger's test p	No. of Imputed Studies (k_0)	Original Effect	Original 95% CI	Adjusted Effect	Adjusted 95% CI
BMI (kg/m^2)	0.4447	32	-1.220	-1.527 to -0.913	-1.715	-2.041 to -1.389
WC (cm)	0.7128	21	-2.854	-3.562 to -2.146	-3.727	-4.450 to -3.004
BF (%)	0.8769	31	-2.385	-2.842 to -1.928	-3.155	-3.620 to -2.691
VO_2max ($mL\ kg^{-1}\ min^{-1}$)	0.0429*	10	2.842	2.121 to 3.564	1.861	1.054 to 2.668
VO_2peak ($mL\ kg^{-1}\ min^{-1}$)	0.0002*	0	2.587	1.850 to 3.323	2.587	1.850 to 3.323
HRmax (bpm)	0.2298	0	-0.428	-1.728 to 0.872	-0.428	-1.728 to 0.872
HRrest (bpm)	0.1940	6	-2.882	-3.793 to -1.971	-2.379	-3.523 to -1.236
SBP (mmHg)	0.0000*	1	-3.884	-4.911 to -2.856	-3.952	-4.979 to -2.924
DBP (mmHg)	0.0241*	11	-1.752	-2.471 to -1.034	-2.403	-3.313 to -1.492
TC (mmol/l)	0.0540	0	-0.352	-0.445 to -0.258	-0.352	-0.445 to -0.258
TG (mmol/l)	0.0000*	0	-0.229	-0.289 to -0.169	-0.229	-0.289 to -0.169
HDL-C (mmol/l)	0.1617	13	0.077	0.051 to 0.103	0.100	0.073 to 0.126
LDL-C (mmol/l)	0.1289	0	-0.276	-0.344 to -0.208	-0.276	-0.344 to -0.208
FPG (mmol/l)	0.3146	0	-0.202	-0.276 to -0.127	-0.202	-0.276 to -0.127
HbA1c (%)	0.4798	0	-0.044	-0.088 to 0.001	-0.044	-0.088 to 0.001
FINS (μU/mL)	0.2372	0	-4.008	-5.299 to -2.717	-4.008	-5.299 to -2.717
Depression	0.0758	5	-0.046	-0.151 to 0.059	0.025	-0.092 to 0.141
Anxiety	0.1278	0	-0.507	-1.629 to 0.615	-0.507	-1.629 to 0.615
Self-esteem	0.0799	4	0.197	0.045 to 0.349	0.089	-0.116 to 0.295
Self-worth	0.0870	2	0.246	0.099 to 0.393	0.190	0.050 to 0.330

Note: BMI, body mass index; WC, waist circumference; BF%, percentage of body fat; VO_2max, maximal oxygen consumption; VO_2peak, peak oxygen uptake; HRmax, max heart rate; HRrest, resting heart rate; SBP, systolic blood pressure; DBP, diastolic blood pressure; TC, total cholesterol; TG, triglyceride; HDL-C, high-density lipoprotein cholesterol; LDL-C, low-density lipoprotein cholesterol; FPG, fasting plasma glucose; HbA1c, hemoglobin A1c; FINS, fasting insulin. To further explore and correct potential asymmetry, Duval and Tweedie's trim-and-fill procedure was applied under a random-effects model.

Egger's regression test (SND ~ 1/SE) was used to assess publication bias.

The Trim-and-Fill method by Duval and Tweedie was used to estimate the number of missing studies (k_0) and adjust the pooled effect size.

95% CI: 95% confidence interval.

P values < 0.05 were considered statistically significant. *$P < 0.05$

Figure S25. Trim-and-fill funnel plots of meta-analysis results for 20 outcome indicators in children and adolescents with overweight or obesity

Figure S25.1. Trim-and-fill funnel plot for BMI

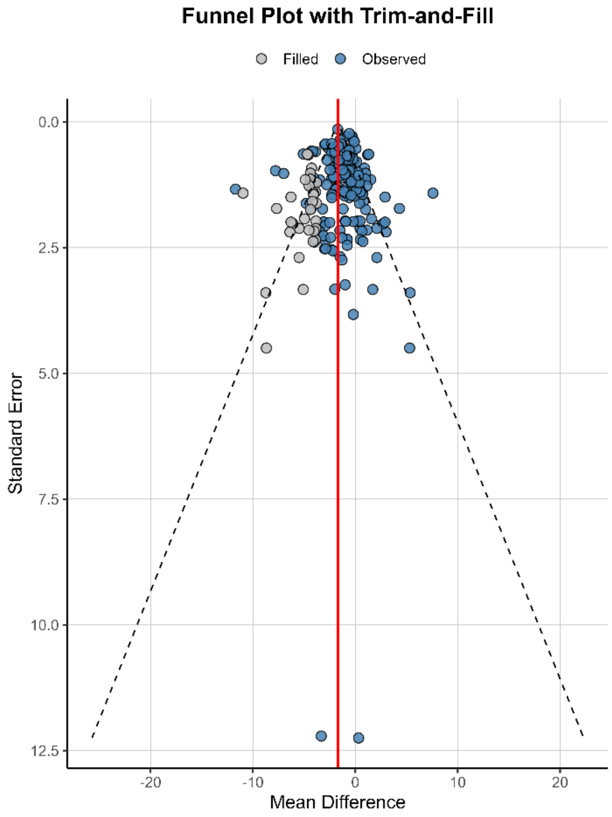

Figure S25.2. Trim-and-fill funnel plot for WC

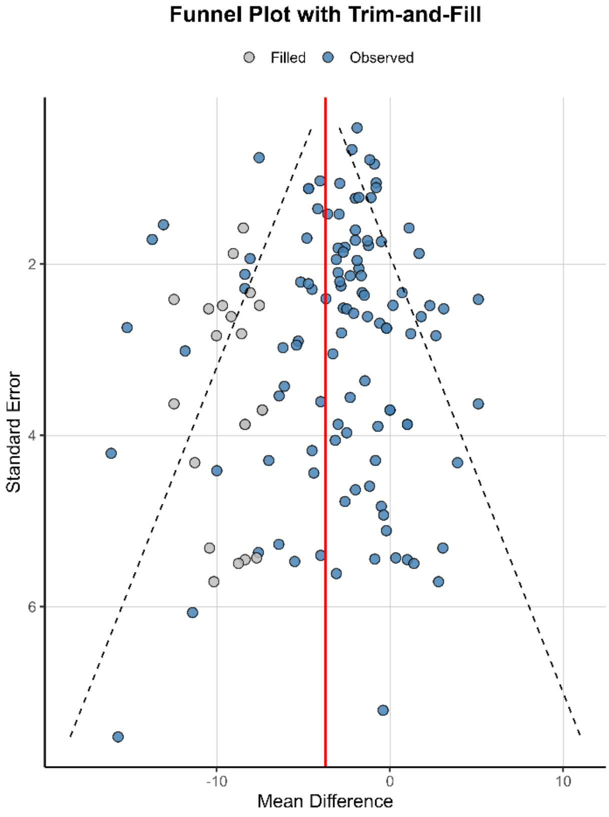

Figure S25.3. Trim-and-fill funnel plot for BF%

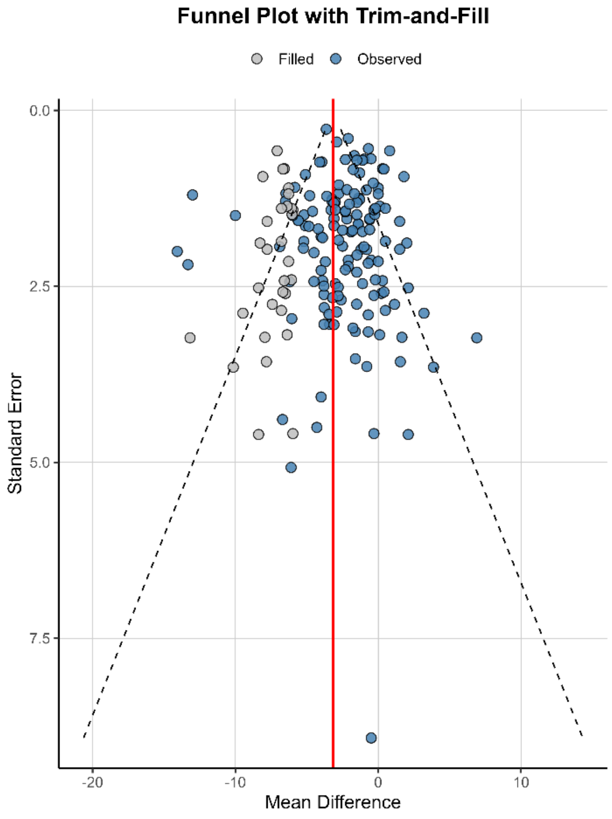

Figure S25.4. Trim-and-fill funnel plot for VO_2max

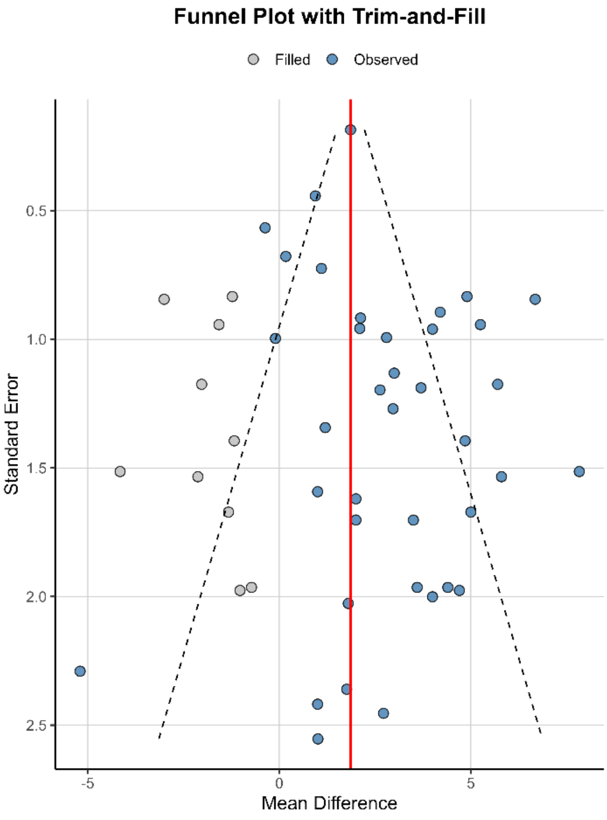

Figure S25.5. Trim-and-fill funnel plot for VO2peak

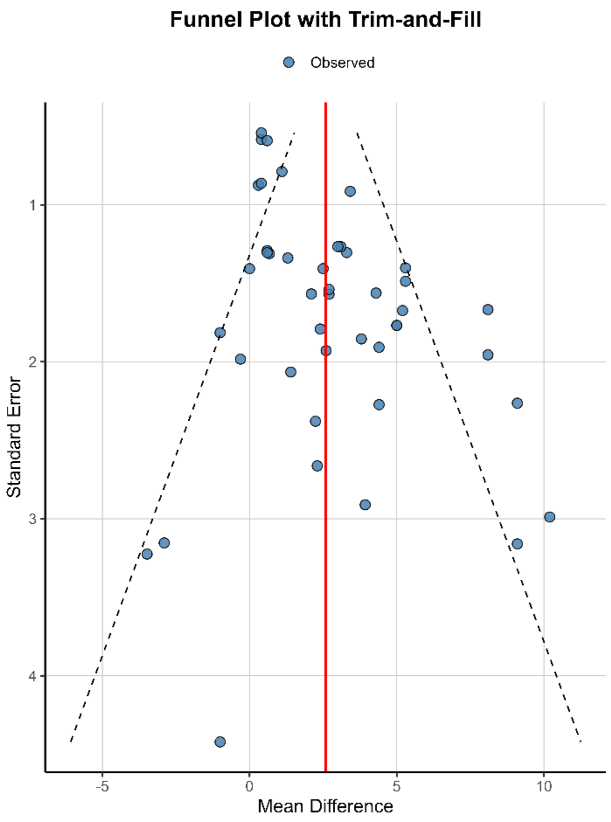

Figure S25.6. Trim-and-fill funnel plot for HRmax

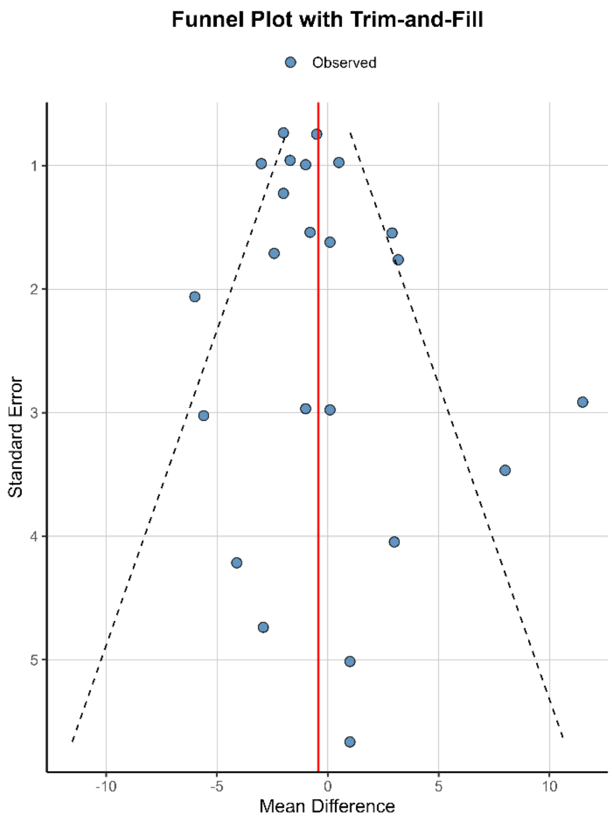

Figure S25.7. Trim-and-fill funnel plot for HRrest

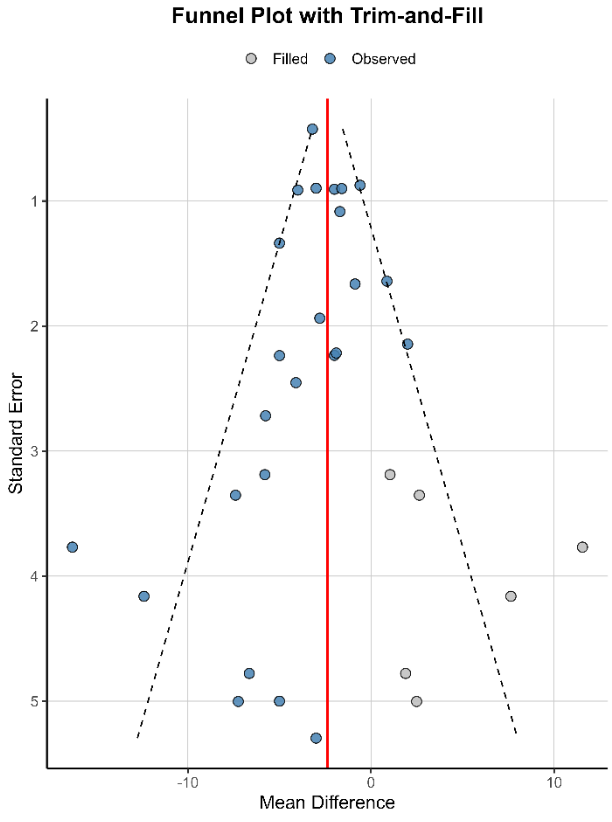

Figure S25.8. Trim-and-fill funnel plot for SBP

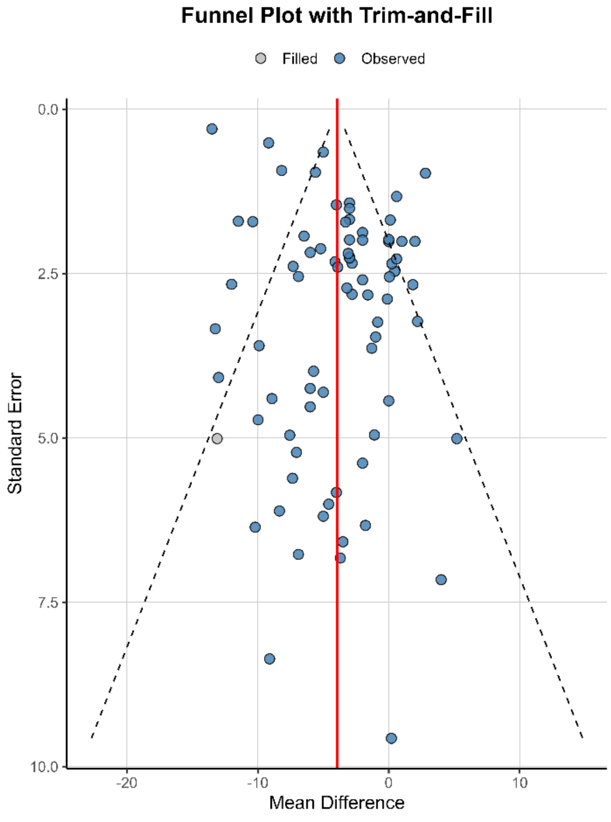

Figure S25.9. Trim-and-fill funnel plot for DBP

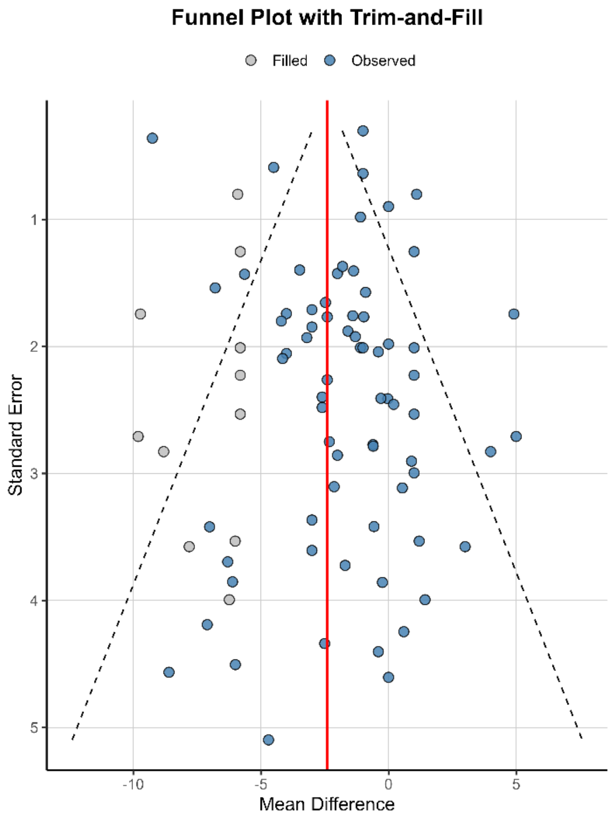

Figure S25.10. Trim-and-fill funnel plot for TC

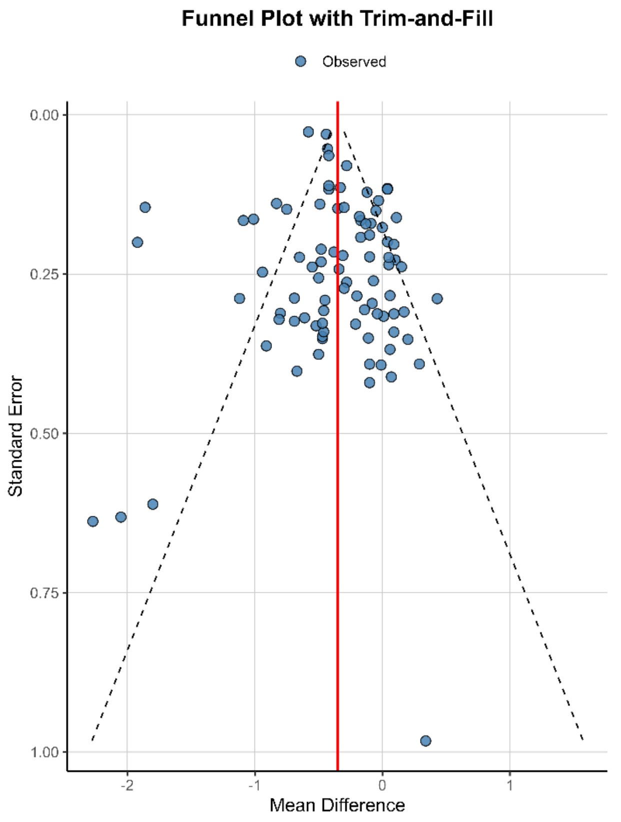

Figure S25.11. Trim-and-fill funnel plot for TG

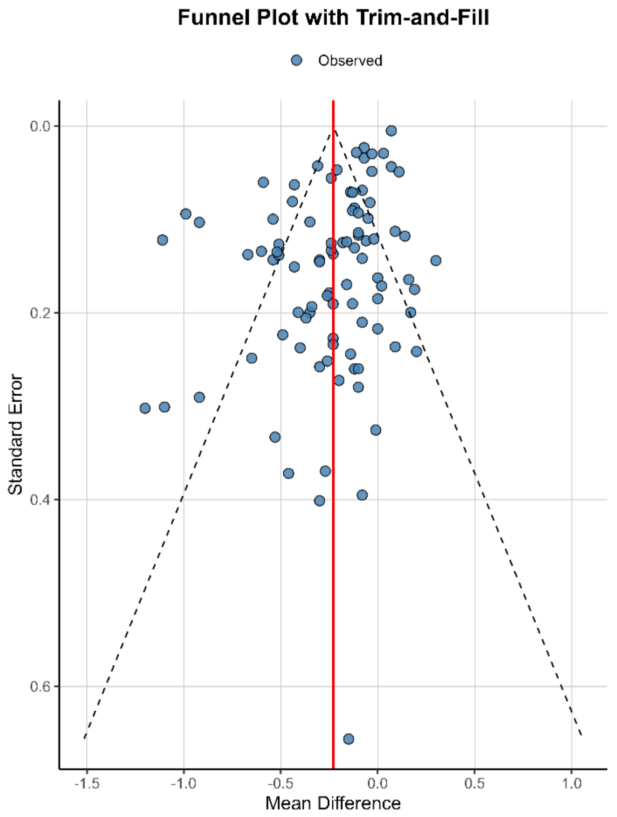

Figure S25.12. Trim-and-fill funnel plot for HDL-C

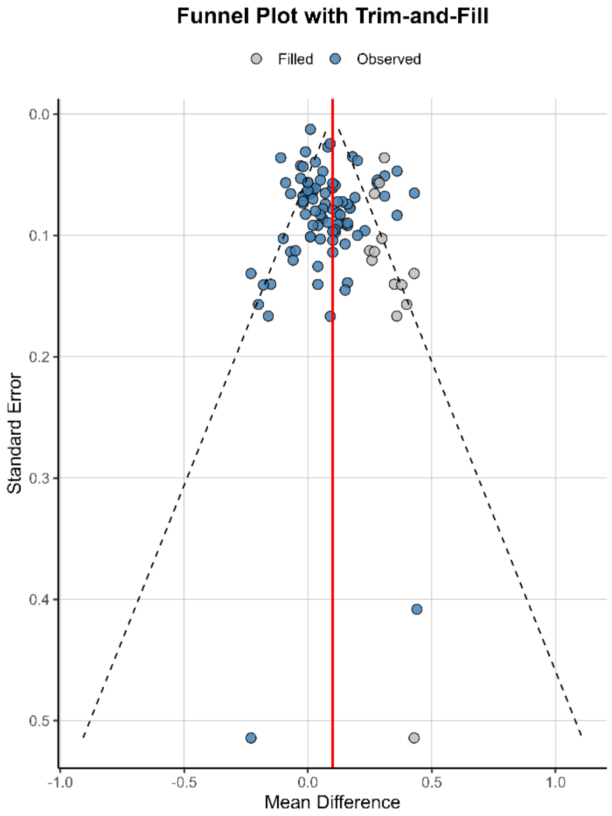

Figure S25.13. Trim-and-fill funnel plot for LDL-C

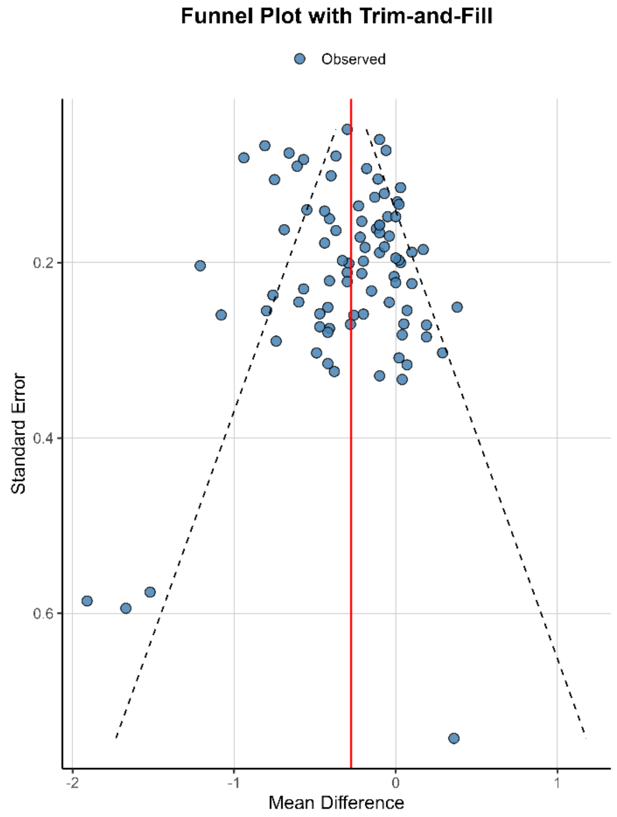

Figure S25.14. Trim-and-fill funnel plot for FPG

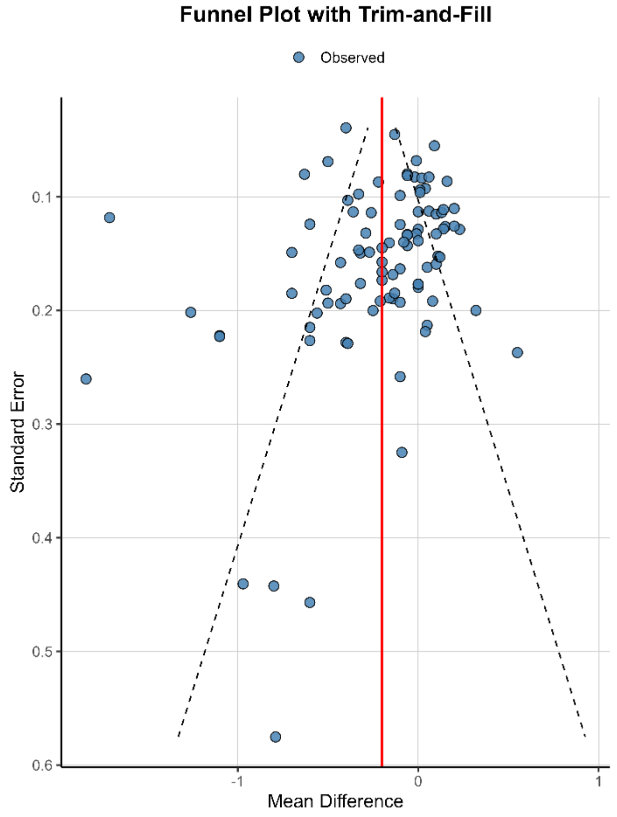

Figure S25.15. Trim-and-fill funnel plot for HbA1c

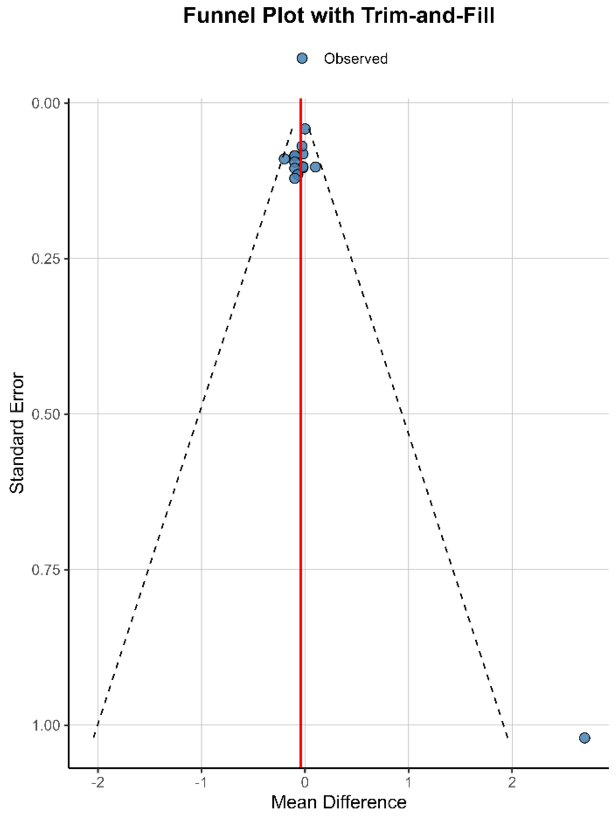

Figure S25.16. Trim-and-fill funnel plot for FINS

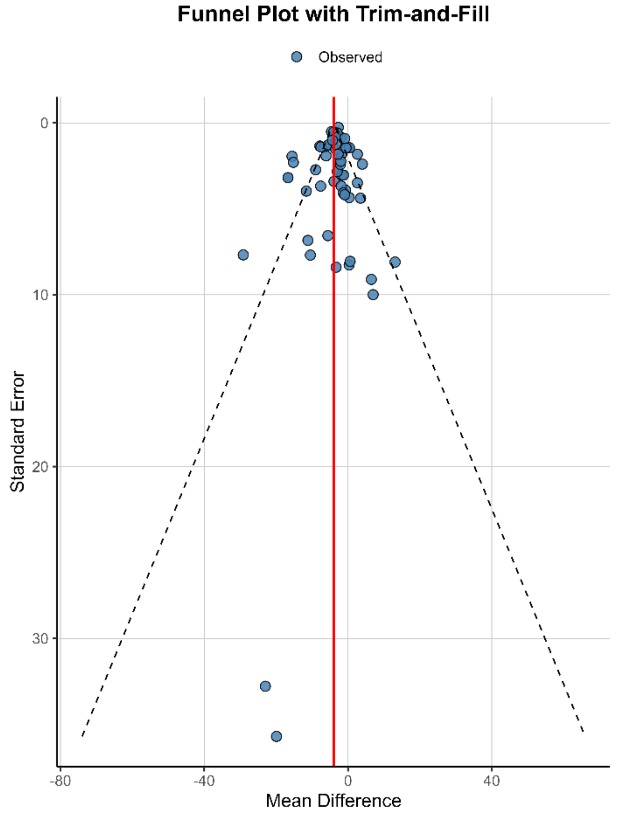

Figure S25.17. Trim-and-fill funnel plot for Depression

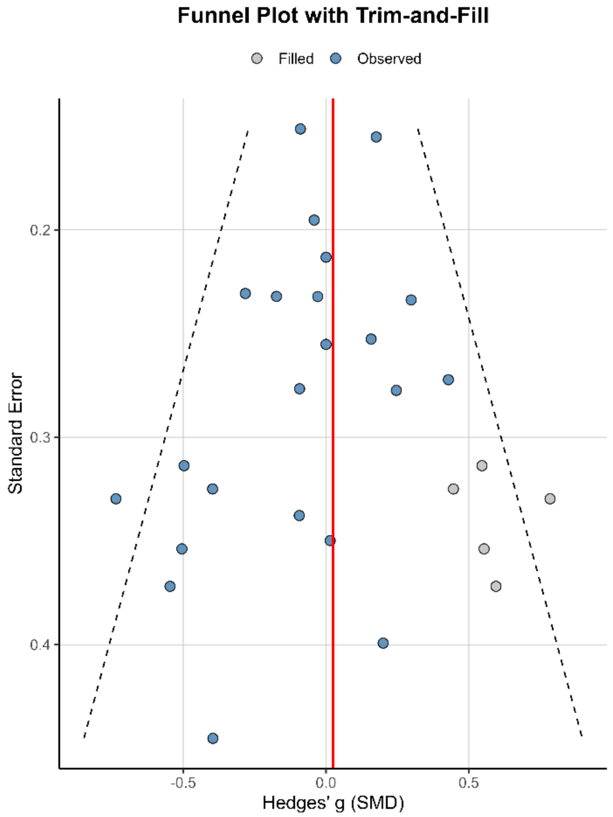

Figure S25.18. Trim-and-fill funnel plot for Anxiety

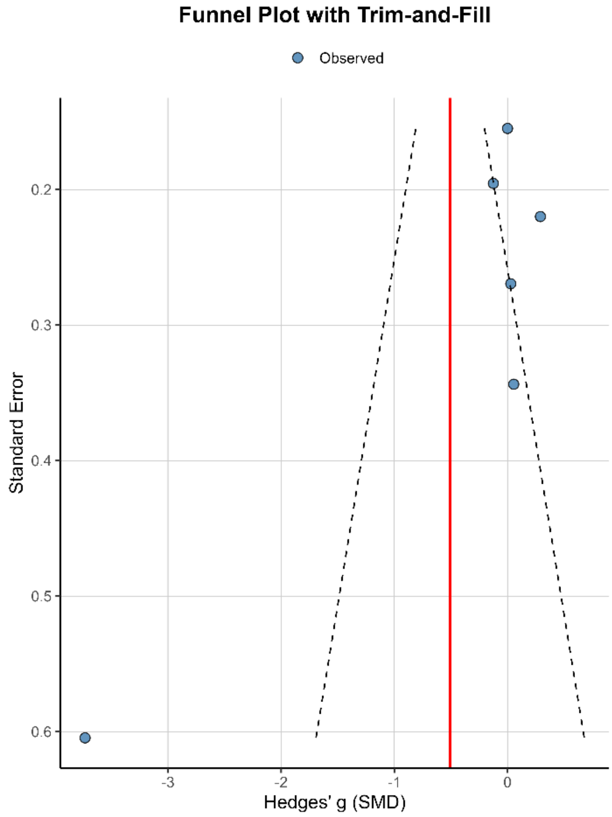

Figure S25.19. Trim-and-fill funnel plot for Self-esteem

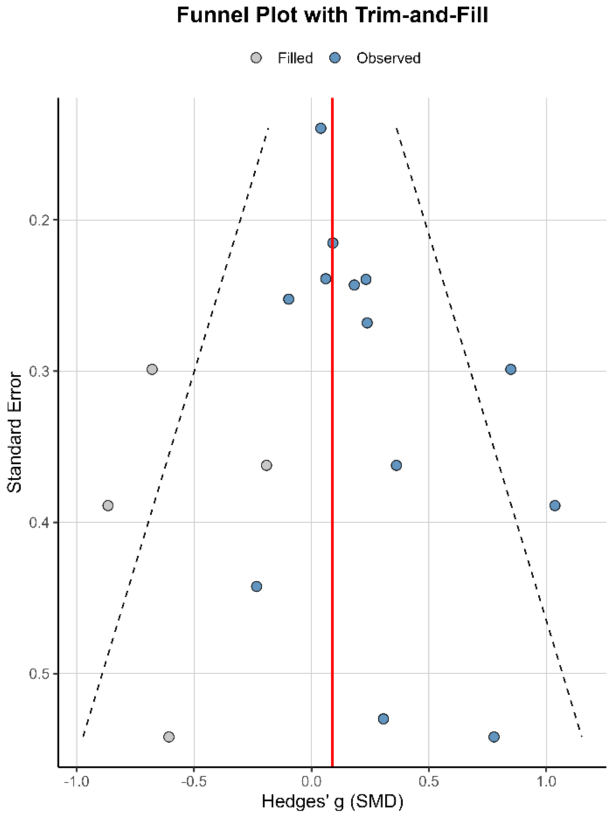

Figure S25.20. Trim-and-fill funnel plot for Self-worth

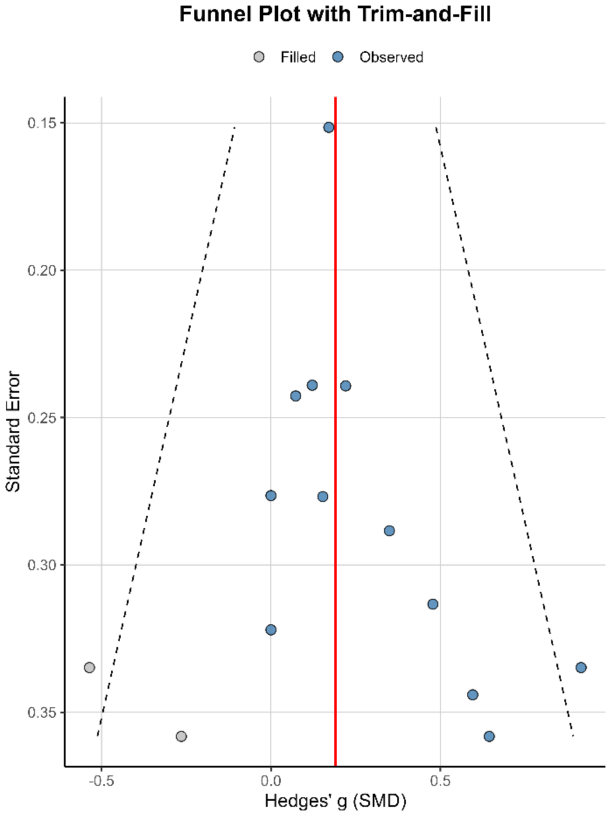

Appendix 5. List of all included studies in the meta-analysis

1. Abassi W, Ouerghi N and Feki M, et al. Effects of moderate- vs. High-intensity interval training on physical fitness, enjoyment, and affective valence in overweight/obese female adolescents: a pre-/post-test study. *Eur Rev Med Pharmacol Sci* 2023; 27: 3809-3822.
2. Abassi W, Ouerghi N and Ghouili H, et al. Greater effects of high- compared with moderate-intensity interval training on thyroid hormones in overweight/obese adolescent girls. *Horm Mol Biol Clin Investig* 2020; 41.
3. Abassi W, Ouerghi N and Nikolaidis PT, et al. Interval training with different intensities in overweight/obese adolescent females. *Int J Sports Med* 2022; 43: 434-443.
4. Ablakmu G, Minnan W and Yanhong H, et al. The effects of a low-energy balanced diet combined with aerobic exercise on body fat metabolism, insulin resistance, and oxidative stress in children with simple obesity. *Modern Biomedical Progress* 2022; 22: 3459-3462.
5. Aguilar-Cordero MJ, Rodriguez-Blanque R and Leon-Rios X, et al. Influence of physical activity on blood pressure in children with overweight/obesity: a randomized clinical trial. *Am J Hypertens* 2020; 33: 131-136.
6. Ahmadi A, Moheb-Mohammadi F and Navabi ZS, et al. The effects of aerobic training, resistance training, combined training, and healthy eating recommendations on lipid profile and body mass index in overweight and obese children and adolescents: a randomized clinical trial. *ARYA Atheroscler* 2020; 16: 226-234.
7. Alberga AS, Prud Homme D and Sigal RJ, et al. Effects of aerobic training, resistance training, or both on cardiorespiratory and musculoskeletal fitness in adolescents with obesity: the HEARTY trial. *Appl Physiol Nutr Metab* 2016; 41: 255-265.
8. Alves A, Venancio TL and Honorio S, et al. Multicomponent training with different frequencies on body composition and physical fitness in obese children. *An Acad Bras Cienc* 2019; 91: e20181264.
9. Azad A, Gharakhanlou R and Niknam A, et al. Effects of aerobic exercise on lung function in overweight and obese students. *Tanaffos* 2011; 10: 24-31.
10. Barbeau P, Johnson MH and Howe CA, et al. Ten months of exercise improves general and visceral adiposity, bone, and fitness in black girls. *Obesity (Silver Spring)* 2007; 15: 2077-2085.
11. Ben OO, Elloumi M and Zouhal H, et al. Effect of individualized exercise training combined with diet restriction on inflammatory markers and IGF-1/IGFBP-3 in obese children. *Ann Nutr Metab* 2010; 56: 260-266.
12. Benson AC, Torode ME and Fiatarone SM. The effect of high-intensity progressive resistance training on adiposity in children: a randomized controlled trial. *Int J Obes (Lond)* 2008; 32: 1016-1027.
13. Bezerra TA, Souza FA and Pessoa M, et al. Effects of a multicomponent intervention on cardiovascular risk factors in overweight children: a randomized clinical trial in light of complex systems. *Int J Environ Health Res* 2023; 33: 1368-1378.
14. Bharath LP, Choi WW and Cho J, et al. Combined resistance and aerobic exercise training reduces insulin resistance and central adiposity in adolescent girls who are obese: randomized clinical trial. *Eur J Appl Physiol* 2018; 118: 1653-1660.
15. Boer P, Meeus M and Terblanche E, et al. The influence of sprint interval training on body composition, physical and metabolic fitness in adolescents and young adults with intellectual disability: a randomized controlled trial. *Clin Rehabil* 2014; 28: 221-231.
16. Boff RM, Dornelles MA and Feoli A, et al. Transtheoretical model for change in obese adolescents: MERC randomized clinica trial. *J Health Psychol* 2020; 25: 2272-2285.
17. Bowen L and Zhengzhen W. The effect of short-term exercise and nutrition on certain metabolic indicators in obese adolescents.
. *Chinese Journal of Rehabilitation Theory and Practice* 2010; 16: 483-485.
18. Brennan L, Wilks R and Walkley J, et al. Treatment acceptability and psychosocial outcomes of a randomised controlled trial of a cognitive behavioural lifestyle intervention for overweight and obese adolescents. *Behav Change* 2012; 29: 36-62.
19. Bruyndonckx L, Hoymans VY and De Guchtenaere A, et al. Diet, exercise, and endothelial function in obese adolescents.

Pediatrics (Evanston) 2015; 135: e653.
20. Cao M, Tang Y and Zou Y. Integrating high-intensity interval training into a school setting improve body composition, cardiorespiratory fitness and physical activity in children with obesity: a randomized controlled trial. *J Clin Med* 2022; 11: 5436.
21. Carrel AL, Clark RR and Peterson SE, et al. Improvement of fitness, body composition, and insulin sensitivity in overweight children in a school-based exercise program: a randomized, controlled study. *Archives of pediatrics & adolescent medicine* 2005; 159: 963-968.
22. Chae H, Kwon Y and Rhie Y, et al. Effects of a structured exercise program on insulin resistance, inflammatory markers and physical fitness in obese korean children. *J Pediatr Endocrinol Metab* 2010; 23: 1065.
23. Chuensiri N, Suksom D and Tanaka H. Effects of high-intensity intermittent training on vascular function in obese preadolescent boys. *Child Obes* 2018; 14: 41-49.
24. Croker H, Viner RM and Nicholls D, et al. Family-based behavioural treatment of childhood obesity in a UK national health service setting: randomized controlled trial. *Int J Obes (Lond)* 2012; 36: 16-26.
25. Crova C, Struzzolino I and Marchetti R, et al. Cognitively challenging physical activity benefits executive function in overweight children. *J Sports Sci* 2014; 32: 201-211.
26. Cvetković N, Stojanović E and Stojiljković N, et al. Exercise training in overweight and obese children: recreational football and high-intensity interval training provide similar benefits to physical fitness. *Scand J Med Sci Sports* 2018; 28: 18-32.
27. Daley AJ, Copeland RJ and Wright NP, et al. Exercise therapy as a treatment for psychopathologic conditions in obese and morbidly obese adolescents: a randomized, controlled trial. *Pediatrics* 2006; 118: 2126-2134.
28. Danielsen YS, Nordhus IH and Juliusson PB, et al. Effect of a family-based cognitive behavioural intervention on body mass index, self-esteem and symptoms of depression in children with obesity (aged 7-13): a randomised waiting list controlled trial. *Obes Res Clin Pract* 2013; 7: e116-e128.
29. Davis CL, Litwin SE and Pollock NK, et al. Exercise effects on arterial stiffness and heart health in children with excess weight: the SMART RCT. *Int J Obes (Lond)* 2020; 44: 1152-1163.
30. Davis JN, Kelly LA and Lane CJ, et al. Randomized control trial to improve adiposity and insulin resistance in overweight latino adolescents. *Obesity (Silver Spring)* 2009; 17: 1542-1548.
31. Davis JN, Tung A and Chak SS, et al. Aerobic and strength training reduces adiposity in overweight latina adolescents. *Med Sci Sports Exerc* 2009; 41: 1494-1503.
32. de Lira CT, Dos SM and Gomes PP, et al. Aerobic training performed at ventilatory threshold improves liver enzymes and lipid profile related to non-alcoholic fatty liver disease in adolescents with obesity. *Nutr Health* 2017; 23: 281-288.
33. Debar LL, Stevens VJ and Perrin N, et al. A primary care-based, multicomponent lifestyle intervention for overweight adolescent females. *Pediatrics (Evanston)* 2012; 129: e611.
34. Dennis BA, Ergul A and Gower BA, et al. Oxidative stress and cardiovascular risk in overweight children in an exercise intervention program. *Child Obes* 2013; 9: 15-21.
35. Dias KA, Ingul CB and Tjønna AE, et al. Effect of high-intensity interval training on fitness, fat mass and cardiometabolic biomarkers in children with obesity: a randomised controlled trial. *Sports Med* 2018; 48: 733-746.
36. Duft RG, Castro A and Bonfante ILP, et al. Altered metabolomic profiling of overweight and obese adolescents after combined training is associated with reduced insulin resistance. *Sci Rep* 2020; 10: 16880.
37. Elmahgoub SM, Lambers S and Stegen S, et al. The influence of combined exercise training on indices of obesity, physical fitness and lipid profile in overweight and obese adolescents with mental retardation. *Eur J Pediatr* 2009; 168: 1327-1333.
38. Elnaggar RK, Shendy MA and Elfakharany MS. Effect of 8 weeks of incremental aerobic training on inflammatory mediators, cardiorespiratory indices, and functional capacity in obese children with bronchial asthma. *Pediatr Exerc Sci* 2021; 33: 23-31.
39. Eskandari M, Hooshmand MB and Bagheri R, et al. Effects of interval jump rope exercise combined with dark chocolate

supplementation on inflammatory adipokine, cytokine concentrations, and body composition in obese adolescent boys. *Nutrients* 2020; 12.

40. Espinoza-Silva M, Latorre-Román P and Párraga-Montilla J, et al. Response of obese schoolchildren to high-intensity interval training applied in the school context. *Endocrinología, Diabetes y Nutrición (English ed.)* 2019; 66: 611-619.
41. Faria WF, Mendonça FR and Santos GC, et al. Effects of 2 methods of combined training on cardiometabolic risk factors in adolescents: a randomized controlled trial. *Pediatr Exerc Sci* 2020; 32: 217-226.
42. Farpour-Lambert NJ, Aggoun Y and Marchand LM, et al. Physical activity reduces systemic blood pressure and improves early markers of atherosclerosis in pre-pubertal obese children. *J Am Coll Cardiol* 2009; 54: 2396-2406.
43. Farpour-Lambert NJ, Martin XE and Bucher DTS, et al. Effectiveness of individual and group programmes to treat obesity and reduce cardiovascular disease risk factors in pre-pubertal children. *Clin Obes* 2019; 9: e12335.
44. Ghorbanian B, Ravassi A and Reza M, et al. The effects of rope training on lymphocyte ABCA1 expression, plasma ApoA-i and HDL-c in boy adolescents. *Int J Endocrinol Metab* 2013; 11.
45. Goldfield GS, Kenny GP and Alberga AS, et al. Effects of aerobic training, resistance training, or both on psychological health in adolescents with obesity: the HEARTY randomized controlled trial. *J Consult Clin Psychol* 2015; 83: 1123-1135.
46. Gonzalez-Galvez N, Soler-Marin A and Abelleira-Lamela T, et al. Eight weeks of high-intensity interval vs. Sprint interval training effects on overweight and obese adolescents carried out during the cool-down period of physical education classes: randomized controlled trial. *Front Public Health* 2024; 12: 1394328.
47. Guijun D. Study on the effect of combined weight loss methods on blood pressure, blood lipids, and other indicators in adolescents with simple obesity.
. *Journal of Shandong Sport University* 2009; 25: 42-45.
48. Guo H, Zeng X and Zhuang Q, et al. Intervention of childhood and adolescents obesity in shantou city. *Obes Res Clin Pract* 2015; 9: 357-364.
49. Haiying J and Lirong Y. Impacts of aerobic exercise on the obesity of adolescents and their lipid metabolism. *Rev Bras Med Esporte* 2023; 29.
50. Hamila A, Younes M and Cottin F, et al. Effects of walking exercises on body composition, heart rate variability, and perceptual responses in overweight and obese adolescents. *Sci Sports* 2018; 33: e191-e202.
51. Harder-Lauridsen NM, Birk NM and Ried-Larsen M, et al. A randomized controlled trial on a multicomponent intervention for overweight school-aged children - copenhagen, denmark. *BMC Pediatr* 2014; 14: 273.
52. Hay J, Wittmeier K and Macintosh A, et al. Physical activity intensity and type 2 diabetes risk in overweight youth: a randomized trial. *Int J Obes (Lond)* 2016; 40: 607-614.
53. Heidarianpour A, Shokri E and Sadeghian E, et al. Combined training in addition to cortisol reduction can improve the mental health of girls with precocious puberty and obesity. *Front Pediatr* 2023; 11: 1241744.
54. Hong W and Ling-Ling Z. Curative efficacy and effects on the CRP and insulin level of comprehensive community intervention for children simple obesity. *Journal of Laboratory Medicine and Clinical Research* 2016: 381-382, 385.
55. Hongling C, Peng P and Zhu R, et al. The effect of 8-week exercise prescription intervention on aerobic capacity, body composition, blood lipids, and c-reactive protein in obese adolescents.
. *Journal of Jilin University (Medical Edition)* 2012.
56. Horner K, Kuk JL and Barinas-Mitchell E, et al. Effect of aerobic versus resistance exercise on pulse wave velocity, intima media thickness and left ventricular mass in obese adolescents. *Pediatr Exerc Sci* 2015; 27: 494-502.
57. Jain V, Kumar B and Sharma A, et al. A comprehensive yoga programme for weight reduction in children & adolescents with obesity: a randomized controlled trial. *Indian J Med Res* 2022; 155: 387-396.
58. Jelalian E, Jandasek B and Wolff JC, et al. Cognitive-behavioral therapy plus healthy lifestyle enhancement for depressed, overweight/obese adolescents: results of a pilot trial. *J Clin Child Adolesc Psychol* 2019; 48: S24-S33.
59. Jelalian E, Sato A and Hart CN. The effect of group-based weight control intervention on adolescent psychosocial outcomes:

perceived peer rejection, social anxiety and self-concept. *Child Health Care* 2011; 40: 197-211.

60. Jiafen L and Wenjun S. The effect of combined exercise and dietary intervention on lipid metabolism, insulin sensitivity, and low-grade inflammation in obese children. *Journal of Hainan Medical University* 2017; 23: 2240-2242.

61. Jian S and Fengzhen S. Evaluation of the weight loss effect of fitness belly dance prescription on obese female college students.
. *Chinese Journal of School Health* 2014; 35: 78-80.

62. Jianjun L, Shulin C and Haiyan W. Interuention effects of exercise on college obese adolescents. *Shandong Sports Science & Technology* 2012; 34: 22-26.

63. Julian V, Costa D and O'Malley G, et al. Bone response to high-intensity interval training versus moderate-intensity continuous training in adolescents with obesity. *Obes Facts* 2022; 15: 46-54.

64. Jun D, Zhen J and Bing Z. Exercise and nutrition therapy for simple obesity in children. *CHINESE JOURNAL OF CLINICAL REHABILITATION* 2006; 10: 20-22.

65. Kai W, Baichao X and Chaoxin W, et al. A study on the effects of combined exercise interventions on body composition, cardiovascular risk factors, and cardiorespiratory fitness in obese female adolescents. *Chinese General Practice* 2024; 27: 1109-1117.

66. Karacabey K. The effect of exercise on leptin, insulin, cortisol and lipid profiles in obese children. *J Int Med Res* 2009; 37: 1472-1478.

67. Ke LI, Ningchuan LI and Yifan D, et al. Analysis of the effect of 12-week high-intensity fitness exercises on improving metabolic disorders among obese adoles-cents. *Chinese Journal of School Health* 2024; 45: 788-793.

68. Kelishadi R, Hashemipour M and Mohammadifard N, et al. Short- and long-term relationships of serum ghrelin with changes in body composition and the metabolic syndrome in prepubescent obese children following two different weight loss programmes. *Clin Endocrinol (Oxf)* 2008; 69: 721-729.

69. Kelly AS, Wetzsteon RJ and Kaiser DR, et al. Inflammation, insulin, and endothelial function in overweight children and adolescents: the role of exercise. *J Pediatr* 2004; 145: 731-736.

70. Kelly L, Holmberg PM and Schroeder ET, et al. Effect of home-based strength training program on IGF-i, IGFBP-1 and IGFBP-3 in obese latino boys participating in a 16-week randomized controlled trial. *Journal of pediatric endocrinology & metabolism : JPEM* 2019; 32: 1121.

71. Khan NA, Raine LB and Drollette ES, et al. Impact of the FITKids physical activity intervention on adiposity in prepubertal children. *Pediatrics* 2014; 133: e875-e883.

72. Kim ES, Im JA and Kim KC, et al. Improved insulin sensitivity and adiponectin level after exercise training in obese korean youth. *Obesity (Silver Spring)* 2007; 15: 3023-3030.

73. Kim HJ, Tak YJ and Lee SY, et al. Effects of a 12-week diet versus diet plus aerobic and resistance exercise program on acylated and desacylated ghrelin, and ghrelin o-acyltransferase in adolescent girls with obesity. *Int J Environ Res Public Health* 2022; 19.

74. Kim H, Stebbins CL and Chai J, et al. Taekwondo training and fitness in female adolescents. *J Sports Sci* 2011; 29: 133-138.

75. Kim J, Son WM and Headid IR, et al. The effects of a 12-week jump rope exercise program on body composition, insulin sensitivity, and academic self-efficacy in obese adolescent girls. *J Pediatr Endocrinol Metab* 2020; 33: 129-137.

76. Lambrick D, Westrupp N and Kaufmann S, et al. The effectiveness of a high-intensity games intervention on improving indices of health in young children. *J Sports Sci* 2016; 34: 190-198.

77. Larsen KT, Huang T and Ried-Larsen M, et al. A multi-component day-camp weight-loss program is effective in reducing BMI in children after one year: a randomized controlled trial. *PLoS One* 2016; 11: e0157182.

78. Lee G and Choi Y. Effects of an obesity management mentoring program for korean children. *Appl Nurs Res* 2016; 31: 160-164.

79. Lee S, Bacha F and Hannon T, et al. Effects of aerobic versus resistance exercise without caloric restriction on abdominal

fat, intrahepatic lipid, and insulin sensitivity in obese adolescent boys: a randomized, controlled trial. *Diabetes* 2012; 61: 2787-2795.

80. Lee S, Deldin AR and White D, et al. Aerobic exercise but not resistance exercise reduces intrahepatic lipid content and visceral fat and improves insulin sensitivity in obese adolescent girls: a randomized controlled trial. *Am J Physiol Endocrinol Metab* 2013; 305: E1222-E1229.

81. Lee YH, Song YW and Kim HS, et al. The effects of an exercise program on anthropometric, metabolic, and cardiovascular parameters in obese children. *Korean Circ J* 2010; 40: 179-184.

82. Liangmei X, Xiangtian L and Wenjun W, et al. Effects of balanced diet and exercise on simple obesity among middle school students. *Chinese Journal of School Health* 2015; 36: 51-53.

83. Lidan X, Chuanmei W and Xishao L, et al. Evaluation of the effect of nutrition and exercise therapy on children with simple obesity.
. *Chinese Journal of Maternal and Child Health Care* 2012; 27: 66-68.

84. Lima RA, Andersen LB and Soares FC, et al. The causal pathway effects of a physical activity intervention on adiposity in children: the KISS study cluster randomized clinical trial. *Scand J Med Sci Sports* 2020; 30: 1685-1691.

85. Lingling Y. The effect of high-intensity interval training on cardiovascular fitness, body composition, and blood lipid levels in overweight or obese male adolescents. *Chinese Journal of Physical Medicine and Rehabilitation* 2021; 43: 251-253.

86. Li-Qun Z, Hong-Wei G and Kun X. Evaluation of the effect of comprehensive intervention and its cost-benefit analysis in children with obesity. *Acta Nutrimenta Sinica* 2014; 36: 426-429.

87. Liu M, Lin X and Wang X. Decrease in serum chemerin through aerobic exercise plus dieting and its association with mitigation of cardio-metabolic risk in obese female adolescents. *Journal of pediatric endocrinology & metabolism: JPEM* 2018; 31: 127.

88. Lofrano-Prado MC, Donato Junior J and Lambertucci AC, et al. Recreational physical activity improves adherence and dropout in a non-intensive behavioral intervention for adolescents with obesity. *Res Q Exerc Sport* 2022; 93: 659-669.

89. Lopes WA, Leite N and Da SL, et al. Effects of 12 weeks of combined training without caloric restriction on inflammatory markers in overweight girls. *J Sports Sci* 2016; 34: 1902-1912.

90. Luo B, Yang Y and Nieman DC, et al. A 6-week diet and exercise intervention alters metabolic syndrome risk factors in obese chinese children aged 11–13 years. *J Sport Health Sci* 2013; 2: 236-241.

91. Machado E, Jannuzzi F and Telles S, et al. A recreational swimming intervention during the whole school year improves fitness and cardiometabolic risk in children and adolescents with overweight and obesity. *Int J Environ Res Public Health* 2022; 19.

92. Maddison R, Foley L and Ni Mhurchu C, et al. Effects of active video games on body composition: a randomized controlled trial. *The American Journal of Clinical Nutrition* 2011; 94: 156-163.

93. Mccormack SE, Mccarthy MA and Harrington SG, et al. Effects of exercise and lifestyle modification on fitness, insulin resistance, skeletal muscle oxidative phosphorylation and intramyocellular lipid content in obese children and adolescents. *Pediatric Obesity* 2014; 9: 281-291.

94. Mcnarry MA, Lambrick D and Westrupp N, et al. The influence of a six-week, high-intensity games intervention on the pulmonary oxygen uptake kinetics in prepubertal obese and normal-weight children. *Appl Physiol Nutr Metab* 2015; 40: 1012-1018.

95. Medrano M, Arenaza L and Ramírez Vélez R, et al. Prevalence of responders for hepatic fat, adiposity and liver enzyme levels in response to a lifestyle intervention in children with overweight/obesity: EFIGRO randomized controlled trial. *Pediatr Diabetes* 2019; 21: 215-223.

96. Meng C, Yucheng T and Shu L, et al. Effects of school-based high-intensity interval training on body composition, cardiorespiratory fitness and cardiometabolic markers in adolescent boys with obesity: a randomized controlled trial. *BMC Pediatr* 2022; 22: 112.

97. Meyer AA, Kundt G and Lenschow U, et al. Improvement of early vascular changes and cardiovascular risk factors in obese children after a six-month exercise program. *J Am Coll Cardiol* 2006; 48: 1865-1870.
98. Migueles JH, Cadenas-Sanchez C and Lubans DR, et al. Effects of an exercise program on cardiometabolic and mental health in children with overweight or obesity. *JAMA Netw Open* 2023; 6: e2324839.
99. Mohammadi Sefat S, Shabani R and Nazari M. The effect of concurrent aerobic-resistance training on thyroid hormones, blood glucose hemostasis, and blood lipid indices in overweight girls with hypothyroidism. *Horm Mol Biol Clin Investig* 2019; 40.
100. Monteiro PA, Chen KY and Lira FS, et al. Concurrent and aerobic exercise training promote similar benefits in body composition and metabolic profiles in obese adolescents. *Lipids Health Dis* 2015; 14: 153.
101. Moon YI, Park HR and Koo HY, et al. Effects of behavior modification on body image, depression and body fat in obese korean elementary school children. *Yonsei Med J* 2004; 45: 61.
102. Moslehi E, Moslehi Z and Khalvati B. Playing in form of outdoor aerobic exercise is more effective than indoor treadmill exercise on serum orexin-a and weight loss in obese adolescent boys. *Obesity medicine* 2019; 15: 100104.
103. Murphy EC, Carson L and Neal W, et al. Effects of an exercise intervention using dance dance revolution on endothelial function and other risk factors in overweight children. *Int J Pediatr Obes* 2009; 4: 205-214.
104. Naczk A, Gajewska E and Naczk M. Effectiveness of swimming program in adolescents with down syndrome. *International journal of environmental research and public health* 2021; 18: 7441.
105. Naylor LH, Davis EA and Kalic RJ, et al. Exercise training improves vascular function in adolescents with type 2 diabetes. *Physiol Rep* 2016; 4.
106. Nobre GG, de Almeida MB and Nobre IG, et al. Twelve weeks of plyometric training improves motor performance of 7- to 9-year-old boys who were overweight/obese: a randomized controlled intervention. *J Strength Cond Res* 2017; 31: 2091-2099.
107. Nowicka P, Lanke J and Pietrobelli A, et al. Sports camp with six months of support from a local sports club as a treatment for childhood obesity. *Scand J Public Health* 2009; 37: 793-800.
108. Park TG, Hong HR and Lee J, et al. Lifestyle plus exercise intervention improves metabolic syndrome markers without change in adiponectin in obese girls. *Ann Nutr Metab* 2007; 51: 197-203.
109. Pena A, Olson ML and Ayers SL, et al. Inflammatory mediators and type 2 diabetes risk factors before and in response to lifestyle intervention among latino adolescents with obesity. *Nutrients* 2023; 15.
110. Peña A, Olson ML and Hooker E, et al. Effects of a diabetes prevention program on type 2 diabetes risk factors and quality of life among latino youths with prediabetes. *JAMA Netw Open* 2022; 5: e2231196.
111. Petty KH, Davis CL and Tkacz J, et al. Exercise effects on depressive symptoms and self-worth in overweight children: a randomized controlled trial. *J Pediatr Psychol* 2009; 34: 929-939.
112. Plavsic L, Knezevic OM and Sovtic A, et al. Effects of high-intensity interval training and nutrition advice on cardiometabolic markers and aerobic fitness in adolescent girls with obesity. *Appl Physiol Nutr Metab* 2020; 45: 294-300.
113. Prado DM, Silva AG and Trombetta IC, et al. Exercise training associated with diet improves heart rate recovery and cardiac autonomic nervous system activity in obese children. *Int J Sports Med* 2010; 31: 860-865.
114. Prado DM, Silva AG and Trombetta IC, et al. Weight loss associated with exercise training restores ventilatory efficiency in obese children. *Int J Sports Med* 2009; 30: 821-826.
115. Qiong C, Jie C and Lijun Z, et al. The effect of different exercise modalities on body composition, inflammatory factors, and physical fitness in obese adolescents.
. *Journal of Jilin University (Medical Edition)* 2015; 41: 1070-1075.
116. R. Morgan A, E. Ali F and G. Sedhom M. Effect of a diet program and aerobic exercise in class II and class III obese children with chronic kidney disease. *Physiotherapy Quarterly* 2021; 29: 35-39.
117. Racil G, Aouichaoui C and Hawani A, et al. The impact of interval training on adiponectin to leptin ratios and on blood

pressures in severely obese adolescent girls: a randomized controlled trial. *J Sports Sci* 2024: 1-9.
118. Racil G, Ben OO and Hammouda O, et al. Effects of high vs. Moderate exercise intensity during interval training on lipids and adiponectin levels in obese young females. *Eur J Appl Physiol* 2013; 113: 2531-2540.
119. Racil G, Coquart JB and Elmontassar W, et al. Greater effects of high- compared with moderate-intensity interval training on cardio-metabolic variables, blood leptin concentration and ratings of perceived exertion in obese adolescent females. *Biol Sport* 2016; 33: 145-152.
120. Racil G, Zouhal H and Elmontassar W, et al. Plyometric exercise combined with high-intensity interval training improves metabolic abnormalities in young obese females more so than interval training alone. *Appl Physiol Nutr Metab* 2016; 41: 103-109.
121. Ramezani A, Gaeini AA and Hosseini M, et al. Effects of three methods of exercise training on cardiovascular risk factors in obese boys. *Iran J Pediatr* 2017; 27.
122. Regaieg S, Charfi N and Kamoun M, et al. The effects of an exercise training program on body composition and aerobic capacity parameters in tunisian obese children. *Indian J Endocrinol Metab* 2013; 17: 1040-1045.
123. Roh HT, Cho SY and So WY. Effects of regular taekwondo intervention on oxidative stress biomarkers and myokines in overweight and obese adolescents. *Int J Environ Res Public Health* 2020; 17.
124. Romero-Perez EM, Gonzalez-Bernal JJ and Soto-Camara R, et al. Influence of a physical exercise program in the anxiety and depression in children with obesity. *Int J Environ Res Public Health* 2020; 17.
125. Salus M, Tillmann V and Remmel L, et al. Effect of supervised sprint interval training on cardiorespiratory fitness and body composition in adolescent boys with obesity. *J Sports Sci* 2022; 40: 2010-2017.
126. Salus M, Tillmann V and Remmel L, et al. Effect of sprint interval training on cardiometabolic biomarkers and adipokine levels in adolescent boys with obesity. *International journal of environmental research and public health* 2022; 19: 12672.
127. Savoye M, Nowicka P and Shaw M, et al. Long-term results of an obesity program in an ethnically diverse pediatric population. *Pediatrics (Evanston)* 2011; 127: 402.
128. Savoye M, Shaw M and Dziura J, et al. Effects of a weight management program on body composition and metabolic parameters in overweight children: a randomized controlled trial. *JAMA* 2007; 297: 2697-2704.
129. Saygın O and Zcan. The effect of twelve week aerobic exercise programme on health related physical fitness components and blood lipids in obese girls. *African journal of pharmacy and pharmacology* 2011; 5: 1441-1445.
130. Schranz N, Tomkinson G and Parletta N, et al. Can resistance training change the strength, body composition and self-concept of overweight and obese adolescent males? A randomised controlled trial. *Br J Sports Med* 2014; 48: 1482-1488.
131. Seo DY, Lee S and Figueroa A, et al. Yoga training improves metabolic parameters in obese boys. *Korean J Physiol Pharmacol* 2012; 16: 175-180.
132. Seo YG, Lim H and Kim Y, et al. The effect of a multidisciplinary lifestyle intervention on obesity status, body composition, physical fitness, and cardiometabolic risk markers in children and adolescents with obesity. *Nutrients* 2019; 11.
133. Shaibi GQ, Cruz ML and Ball G, et al. Effects of resistance training on insulin sensitivity in overweight latino adolescent males. *Med Sci Sports Exerc* 2006; 38: 1208-1215.
134. Shalitin S, Ashkenazi-Hoffnung L and Yackobovitch-Gavan M, et al. Effects of a twelve-week randomized intervention of exercise and/or diet on weight loss and weight maintenance, and other metabolic parameters in obese preadolescent children. *Horm Res Paediatr* 2009; 72: 287-301.
135. Shu LI, Meng C and Yu Z, et al. Effects of high-intensity interval training on visceral adipose tissue and cardiorespiratory fitness in children with obesity. *Journal of Physical Education Recreation and Dance* 2023; 30: 138-144.
136. Sigal RJ, Alberga AS and Goldfield GS, et al. Effects of aerobic training, resistance training, or both on percentage body fat and cardiometabolic risk markers in obese adolescents. *JAMA Pediatr* 2014; 168: 1006.
137. Silva DAS, Petroski EL and Pelegrini A, et al. Effect of physical exercise on the cardiorespiratory response in overweight adolescents. *Turk J Endocrinol Metab* 2012; 16: 14-18.

138. Son WM, Sung KD and Bharath LP, et al. Combined exercise training reduces blood pressure, arterial stiffness, and insulin resistance in obese prehypertensive adolescent girls. *Clin Exp Hypertens* 2017; 39: 546-552.
139. Staiano AE, Abraham AA and Calvert SL. Adolescent exergame play for weight loss and psychosocial improvement: a controlled physical activity intervention. *Obesity (Silver Spring)* 2013; 21: 598-601.
140. Staiano AE, Beyl RA and Guan W, et al. Home-based exergaming among children with overweight and obesity: a randomized clinical trial. *Pediatric obesity* 2018; 13: 724-733.
141. Staiano AE, Marker AM and Beyl RA, et al. A randomized controlled trial of dance exergaming for exercise training in overweight and obese adolescent girls. *Pediatric Obesity* 2017; 12: 120-128.
142. Suarez-Villadat B, Luna-Oliva L and Acebes C, et al. The effect of swimming program on body composition levels in adolescents with down syndrome. *Res Dev Disabil* 2020; 102: 103643.
143. Suarez-Villadat B, Sadarangani KP and Villagra A. Effectiveness of exergames programme to modify body composition and health-related physical fitness in adolescents with down syndrome after COVID-19 quarantine. *Eur J Sport Sci* 2023; 23: 2210-2220.
144. Sun M, Huang X and Yan Y, et al. One-hour after-school exercise ameliorates central adiposity and lipids in overweight chinese adolescents: a randomized controlled trial. *Chin Med J (Engl)* 2011; 124: 323-329.
145. Tan S, Wang J and Cao L. Exercise training at the intensity of maximal fat oxidation in obese boys. *Appl Physiol Nutr Metab* 2016; 41: 49-54.
146. Tan S, Yang C and Wang J. Physical training of 9- to 10-year-old children with obesity to lactate threshold intensity. *Pediatr Exerc Sci* 2010; 22: 477-485.
147. Tas E, Landes RD and Diaz EC, et al. Effects of short-term supervised exercise training on liver fat in adolescents with obesity: a randomized controlled trial. *Obesity (Silver Spring)* 2023; 31: 2740-2749.
148. Tjonna AE, Stolen TO and Bye A, et al. Aerobic interval training reduces cardiovascular risk factors more than a multitreatment approach in overweight adolescents. *Clin Sci (Lond)* 2009; 116: 317-326.
149. Toulabi T, Khosh NNM and Amini F, et al. The influence of a behavior modification interventional program on body mass index in obese adolescents. *J Formos Med Assoc* 2012; 111: 153-159.
150. Tsang TW, Kohn M and Chow CM, et al. A randomized controlled trial of kung fu training for metabolic health in overweight/obese adolescents: the "martial fitness" study. *J Pediatr Endocrinol Metab* 2009; 22: 595.
151. Vasconcellos F, Seabra A and Cunha F, et al. Health markers in obese adolescents improved by a 12-week recreational soccer program: a randomised controlled trial. *J Sports Sci* 2016; 34: 564-575.
152. Velez A, Golem DL and Arent SM. The impact of a 12-week resistance training program on strength, body composition, and self-concept of hispanic adolescents. *J Strength Cond Res* 2010; 24: 1065-1073.
153. Wagener TL, Fedele DA and Mignogna MR, et al. Psychological effects of dance-based group exergaming in obese adolescents. *Pediatric Obesity* 2012; 7.
154. Weintraub DL, Tirumalai EC and Haydel KF, et al. Team sports for overweight children. *Archives of Pediatrics & Adolescent Medicine* 2008; 162: 232.
155. Wen L, Cuiqing C and Xiaoqian Z, et al. The effect of school-based organized exercise intervention on BMI and glucose and lipid metabolism in obese adolescents.
. *Chinese Journal of Sports Medicine* 2008: 329-333.
156. Williams CF, Bustamante EE and Waller JL, et al. Exercise effects on quality of life, mood, and self-worth in overweight children: the SMART randomized controlled trial. *Transl Behav Med* 2019; 9: 451-459.
157. Williams TR, Walker RC and Dearing CG. Nurse facilitated 5000 m running at parkrun improves vulnerable adolescent health in a high deprivation area: a matched pair randomized control trial. *Public Health Nurs* 2024; 41: 458-465.
158. Wong A, Sanchez-Gonzalez MA and Son W, et al. The effects of a 12-week combined exercise training program on arterial stiffness, vasoactive substances, inflammatory markers, metabolic profile, and body composition in obese adolescent girls.

Pediatr Exerc Sci 2018; 30: 480-486.
159. Wong PC, Chia MY and Tsou IY, et al. Effects of a 12-week exercise training programme on aerobic fitness, body composition, blood lipids and c-reactive protein in adolescents with obesity. *Ann Acad Med Singap* 2008; 37: 286-293.
160. Woo J, Shin KO and Yoo JH, et al. The effects of detraining on blood adipokines and antioxidant enzyme in korean overweight children. *Eur J Pediatr* 2012; 171: 235-243.
161. Woo KS, Chook P and Yu CW, et al. Effects of diet and exercise on obesity-related vascular dysfunction in children. *Circulation* 2004; 109: 1981-1986.
162. Wu W, Yang Y and Chu I, et al. Effectiveness of a cross-circuit exercise training program in improving the fitness of overweight or obese adolescents with intellectual disability enrolled in special education schools. *Res Dev Disabil* 2017; 60: 83-95.
163. Xian-Bo Z, Xiu-Qing H and Ming-Xiao S, et al. Lifestyle intervention improves oxidant stress in overweight or obese adolescents. *Chinese Journal of Health Management* 2011; 05: 334-337.
164. Xinhui N and Lijun W. Evaluation of the effects of exercise and dietary intervention on obese uyghur middle school students in xinjiang.
. *Chinese Journal of School Health* 2016; 37: 589-591.
165. Xi-Shao L and Ju-Qing X. Intervention effects on obese children by nutrition control and sports. *CHINESE JOURNAL OF MISDIAGNOSTICS* 2009; 9: 8322-8324.
166. Xishao L, Pingxiang Y and Xiaofang W, et al. Development and evaluation of simple obesity children nutrition health education model. *UNRSING PRACTICE AND RESEARCH* 2010; 7: 3-4.
167. Yackobovitch-Gavan M, Nagelberg N and Phillip M, et al. The influence of diet and/or exercise and parental compliance on health-related quality of life in obese children. *Nutr Res* 2009; 29: 397-404.
168. Yanqin L, Huiyu Z and Jie L. The effect of comprehensive intervention based on aerobic exercise on non-alcoholic fatty liver in children with simple obesity.
. *Chinese Journal of Clinical Research* 2017; 30: 828-830.
169. Youssef H, Groussard C and Lemoine-Morel S, et al. Aerobic training suppresses exercise-induced lipid peroxidation and inflammation in overweight/obese adolescent girls. *Pediatr Exerc Sci* 2015; 27: 67-76.
170. Yu CC, Sung RY and Hau KT, et al. The effect of diet and strength training on obese children's physical self-concept. *J Sports Med Phys Fitness* 2008; 48: 76-82.
171. Yu HJ, Li F and Hu YF, et al. Improving the metabolic and mental health of children with obesity: a school-based nutrition education and physical activity intervention in wuhan, china. *Nutrients* 2020; 12.
172. Yuanyuan Q and Xiaochen W. Evaluation of the intervention effects of different intensity football exercises on body composition and blood biochemical indicators in obese zhuang primary school students. *Chinese Journal of School Health* 2019; 40: 1238-1241.
173. Zehsaz F, Farhangi N and Ghahramani M. Exercise training lowers serum chemerin concentration in obese children. *Sci Sports* 2017; 32: 39-45.
174. Zehsaz F, Farhangi N and Ghahramani M. The response of circulating omentin-1 concentration to 16-week exercise training in male children with obesity. *Phys Sportsmed* 2016; 44: 355-361.
175. Zhaogang M, Zixuan Z and Juan H. Exercise and nutrition interventions for adolescent obesity patients. *Medical Journal of National Defending Forces in Southwest China* 2021; 31: 523-524.
176. Zhi-Hui W. Core strength training to study the influence of obese adolescents serum antioxidant enzyme activity. *Science Technology and Engineering* 2017; 17: 166-171.
